# Improvement of the antimicrobial potency, pharmacokinetic and pharmacodynamic properties of albicidin by incorporation of nitrogen atoms†

**DOI:** 10.1039/d1sc04019g

**Published:** 2021-10-19

**Authors:** Lieby Zborovsky, Leonardo Kleebauer, Maria Seidel, Arseni Kostenko, Leonard von Eckardstein, Frank Otto Gombert, John Weston, Roderich D. Süssmuth

**Affiliations:** Institut für Organische Chemie, Technische Universität Berlin Straße des 17. Juni 124 10623 Berlin Germany suessmuth@chem.tu-berlin.de; Gombert Pharma Research Solutions (GPRS) Dornacherstrasse 120 CH 4053 Basel Switzerland

## Abstract

The worrisome development and spread of multidrug-resistant bacteria demands new antibacterial agents with strong bioactivities particularly against Gram-negative bacteria. Albicidins were recently structurally characterized as highly active antibacterial natural products from the bacterium *Xanthomonas albilineans*. Albicidin, which effectively targets the bacterial DNA-gyrase, is a lipophilic hexapeptide mostly consisting of *para* amino benzoic acid units and only one α-amino acid. In this study, we report on the design and synthesis of new albicidins, containing N-atoms on each of the 5 different phenyl rings. We systematically introduced N-atoms into the aromatic backbone to monitor intramolecular H-bonds and for one derivative correlated them with a significant enhancement of the antibacterial activity and activity spectrum, particularly also towards Gram-positive bacteria. In parallel we conducted DFT calculations to find the most stable conformation of each derivative. A drastic angle-change was observed for the lead compound and shows a preferred planarity through H-bonding with the introduced N-atom at the D-fragment of albicidin. Finally, we went to the next level and conducted the first *in vivo* experiments with an albicidin analogue. Our lead compound was evaluated in two different mouse experiments: In the first we show a promising PK profile and the absence of toxicity and in the second very good efficiency and reduction of the bacterial titre in an *E. coli* infection model with FQ-resistant clinically relevant strains. These results qualify albicidins as active antibacterial substances with the potential to be developed as a drug for treatment of infections caused by Gram-negative and Gram-positive bacteria.

## Abstract 2

A systematic pyridine-scan of the albicidin molecule provides a new lead structure with improved antimicrobial properties.

## Graphical abstract

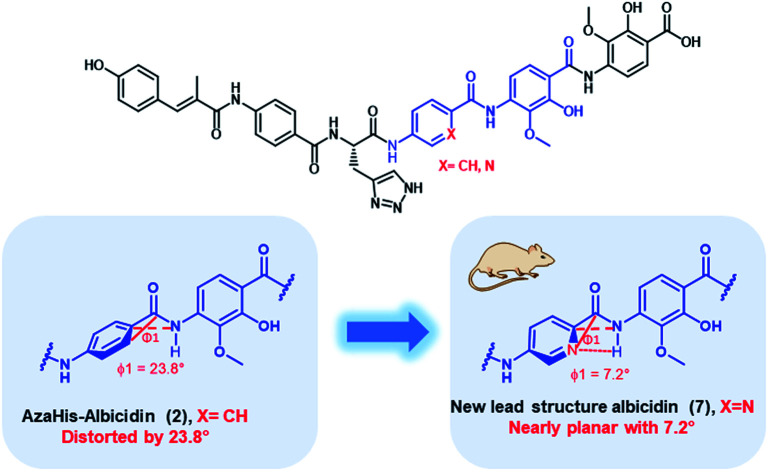

## Introduction

Antibiotic resistance is one of the biggest public health threats today.^1^ Worldwide, 17 million people die each year from bacterial infections. In the United States, approximately two million people are infected annually with bacteria resistant to antibiotics, and 23 000 of these infections are fatal. In Europe, 25 000 people die from drug-resistant bacteria every year.^2^

The current need for novel antibiotics with new modes of action is especially critical for drug-resistant Gram-negative pathogens.^3,4^ Gram-negative microorganisms have a highly restrictive permeability barrier, which limits the penetration of most antibiotics.^5,6^ Only few antibacterial molecule classes can cross this barrier. The quinolones developed in the 1960s are the last big class of antibiotics acting against Gram-negative bacteria.^4,7^ Since then, discovery has been largely limited to narrow-spectrum compounds.^4,8^ This lack of Gram-negative antibiotics is largely responsible for the current antimicrobial resistance crisis.^9^ Pathogens such as *Escherichia coli*, *Klebsiella pneumoniae*, *Pseudomonas aeruginosa* and *Acinetobacter baumannii* have acquired resistance to most, and in some cases to all, antibiotics currently available in clinics leading to a decreasing number of treatment options.^10^ The WHO has classified these drug resistant pathogens as a critical priority for global human health.^11^ Thus, there is an urgent need to provide new effective treatments to meet the increasing public health burden caused by multidrug-resistant Gram-negative bacteria. To meet this need, there is an ongoing search for novel antibiotics, involving novel molecular targets and modes of action. Many such compounds are derived from natural products made by soil microorganisms, primarily Actinomycetes, examples include aminoglycosides, tetracyclines and β-lactams.^12^ Exploration of natural products remains one of the most promising approaches for the discovery of novel antibiotics.

**Fig. 1:**
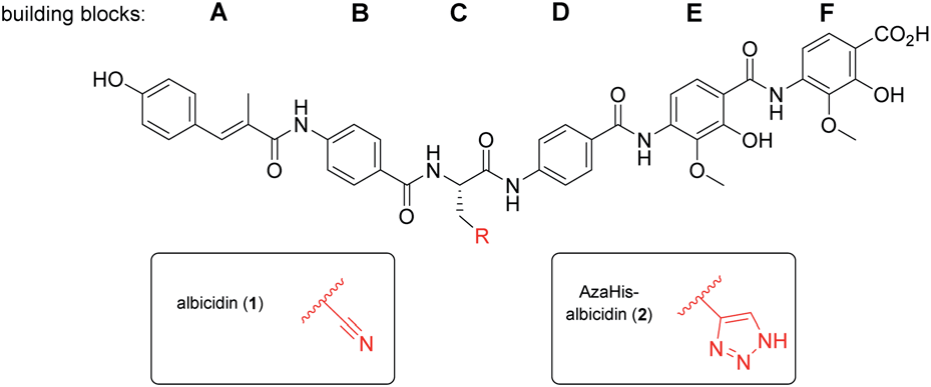
Molecular structure of albicidin (**1**) and AzaHis-albicidin (**2**).

Albicidin (Fig. 1) is a phytotoxic oligoaromatic peptide synthesized by the Gram-negative sugarcane pathogen *Xanthomonas albilineans* that causes leaf scald disease in sugarcane plants.^13^ Structurally related compounds, the cystobactamids and coralmycins have also been identified and are under ongoing development.^14,15^ Albicidin exhibits antibacterial activity at nanomolar concentrations against both Gram-positive and Gram-negative microorganisms and is active against resistant strains. The molecular target is DNA gyrase^16^ with a half maximum inhibitory concentration (IC_50_) of 40 nM (compared to 80 nM of cystobactamid),^17^ thus exhibiting an inhibitory activity similar to that of DNA gyrase-inhibiting quinolones and coumarins.^16^ Hence, albicidin represents a promising lead structure in the search for a new class of antibiotics.

With regard to its biosynthesis, albicidin is assembled by a hybrid polyketide synthase/non-ribosomal peptide synthetase (PKS/NRPS) complex.^18,19^ The structure of albicidin is composed of six building blocks, namely A–F. The N-terminal part is a cinnamoyl residue (building block A) and a *para*-aminobenzoic acid (*p*ABA; building block B). The C terminal part consists of a dipeptidic moiety of *p*ABA, each decorated with adjacent methoxy and hydroxy groups (building blocks E and F) and an undecorated *p*ABA moiety (building block D).^18^ Both, N-terminal and C-terminal parts are linked by a central l-cyanoalanine (building block C) which is the only stereo-centre of the molecule, forming a hinge-like structure (Fig. 1).

Previously, various resistance factors against albicidin have been characterized. The two main resistant factors are the albicidin binding protein AlbA^20,21^ and the serine protease AlbD.^22^

To maximize the bioactivity of albicidin in the quest for a potent novel antimicrobial drug, structure–activity relationship (SAR) studies are key. The first total synthesis of albicidin established by our group employed an allyl protecting group strategy^23^ and paved the way for initial SAR studies. This included many variations of the cinnamoyl residue (building block A)^24,25^ and the incorporation of various α-amino acids at building block C.^26,27^ A recent study demonstrated the significance and influence of the hydroxy and methoxy substituents on the E and F building blocks on the antibacterial activity.^28^ Furthermore, with an azahistidine we have established a new important modification in the central building block C, which resulted in improved antibacterial activities and prevented hydrolysis.^28^

In our ongoing studies we have systematically investigated the effect of the variation of the functional groups on each building block on the antibacterial activity. For the selection of a lead structure however, the compound further needs to display optimised balance between physicochemical, pharmacokinetic (PK) and target related pharmacodynamic (PD) properties. This includes the determination of solubility, plasma stability, plasma protein binding as key parameters and the subsequent *in vivo* profiling in an animal model as the ultimate proof of the drug ability of an antibacterial compound.

Heterocycles play an important role in medicinal chemistry optimisation studies. It is not surprising that the phenyl to pyridine exchange is among the most common and beneficial in medicinal chemistry and more than 60 pyridine-containing drugs have been approved by the FDA.^29^ Pyridine is an electron-deficient aromatic heterocycle containing a ring nitrogen atom, which is present in many natural products including vitamins, coenzymes and alkaloids.^29^ The ring nitrogen is more electronegative than the ring carbons, naturally making the remaining ring carbons more electropositive in comparison to the ring carbons of benzene. The replacement of a CH group with an N atom in aromatic ring systems can have many important effects on molecular and physicochemical properties such as increased basicity which leads to decreased lipophilicity and improved water solubility. Additionally, the introduction of the heterocyclic nitrogen can result in the formation of intra- and intermolecular interactions such as hydrogen bonding (acceptor) and more efficient π-stacking. This may lead to improved pharmacological properties such as active site binding, elevated cell permeability and improved antimicrobial activity. All these properties make pyridine an excellent bioisostere for the phenyl fragment.

As the albicidin antibiotic contains five phenyl rings in its core structure, a SAR study involving a phenyl to pyridyl exchange could lead to new and more potent and pharmacokinetically improved antibiotic compounds. This idea has been probed before by our group, when the hydroxy group of the E and/or F building blocks was replaced by a heterocyclic nitrogen.^28^ However, a systematic study describing the phenyl to pyridyl replacement (pyridine scan) on a full length albicidin as well as its cystobactamid and coralmycin analogues is lacking.

In this work, we report the systematic phenyl to pyridyl building block replacement along the albicidin sequence followed by a thorough SAR testing. We found that substituting the phenyl ring of the D building block with a pyridyl has the highest beneficial impact on the antimicrobial activity leading to a novel albicidin derivative with unprecedented potency. We show, using DFT quantum mechanical calculations that the phenyl to pyridyl replacement leads to preferable planarization of the albicidin backbone, caused by intramolecular hydrogen bonding, which we believe is beneficial for the albicidin's *in vivo* activity. In addition, we report the investigation of the pharmacokinetic and pharmacodynamic properties of this new albicidin derivative. The experimental outcome of an *E. coli* sepsis model proves exposure of the antibacterial by reducing the bacterial burden by several orders of magnitude thus turning albicidin into a promising lead structure for a future antibiotic agent.

## Results

### Synthesis of N-aryl substituted albicidins

**Fig. 2:**
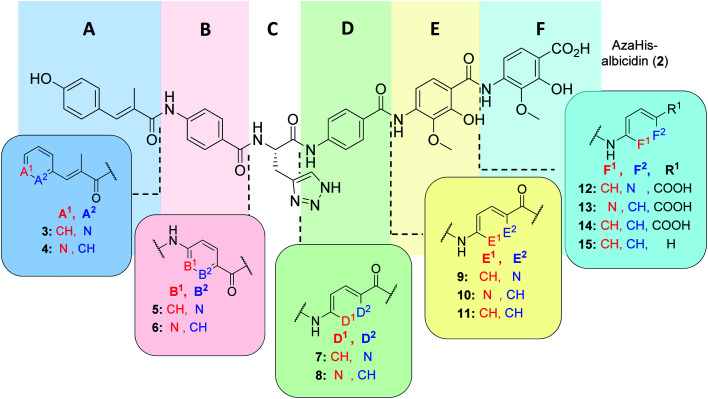
Molecular structure of albicidin derivatives **2**,^27^**11** & **14** (ref. 28) and aimed derivatives **3–10**, **12**, **13** and **15**.

As was previously reported, the substitution of the central cyanoalanine group in building block C with a 2-amino-3-(1*H*-1,2,3-triazol-4-yl)propanoic acid (azahistidine) moiety AzaHis-albicidin **2**, significantly improved the antibacterial activities against various host strains and overcame stability issues of the cyano group which is prone to hydrolysis and thus loss of activity.^28^ For this reason, we chose **2** as a template structure for our pyridine scan/SAR studies aiming for increased, bioactivity and solubility. Since we have previously found that it is possible to replace the methoxy and hydroxy substituted *p*ABA rings with unsubstituted phenyls, whilst retaining comparable antimicrobial activities,^28^ for the simplicity and ease of synthesis, we replaced the phenyl rings in E and F with an unsubstituted pyridyl. For each pyridyl building block we aimed for the synthesis of two isomers, the picolinic acid and nicotinic acid derivatives, thus yielding compounds **3–10**, **12** and **13** (Fig. 2) and compound **15** missing the C-terminal carboxy group.

**Scheme 1:**
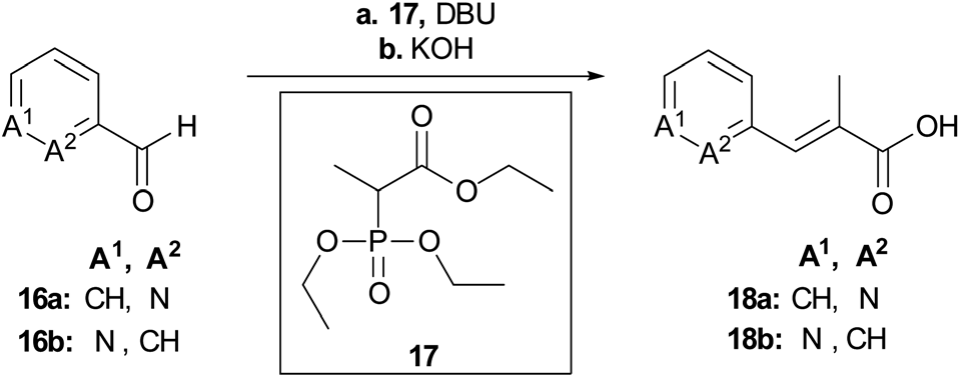
Synthesis of the key building blocks for A-derivatives. Conditions: **a** LiCl, DBU, MeCN, 25 °C, **b** aq. KOH, THF, 25 °C, 71% (**18a**), 80% (**18b**).

**Scheme 2:**
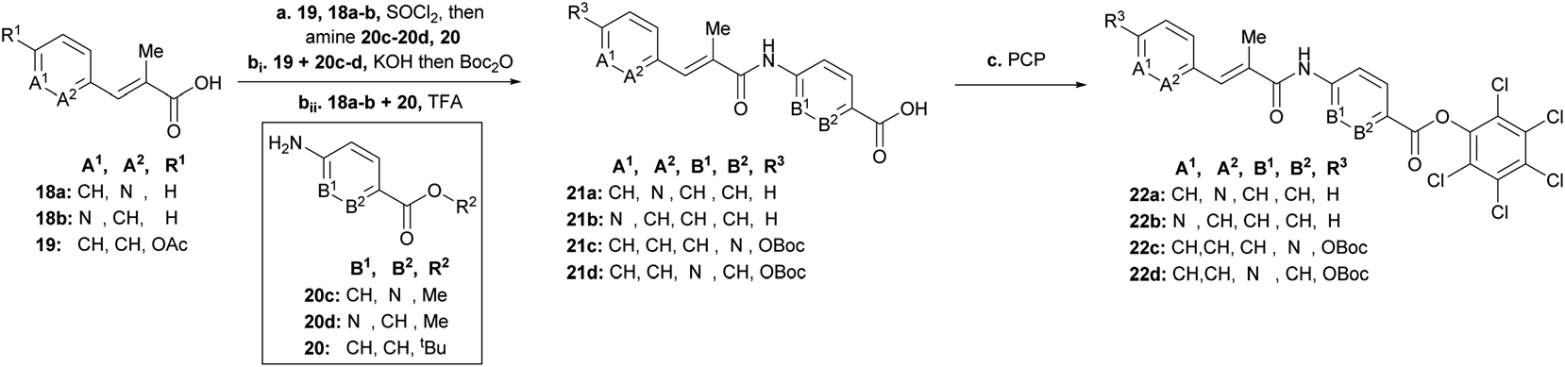
Synthesis of AB-fragments with modified A or B building block. Conditions: **a19**, **18a–b**, SOCl_2_, 95 °C, then (**20c** or **20d** to **19**) or (**20** to **18a** or **18b**) and NEt_3_ or Na_2_CO_3_ (for **20d**), THF, 0 °C → 25 °C; **bi** product of **19** and **20c** or **20d**, KOH, THF, 25 °C, then Boc_2_O, DMAP, THF, 25 °C, 75% (**21c**, 3 steps), 59% (**21d**, 3 steps); **bii** product of **20** and **19a** or **19b**, TFA/CH_2_Cl_2_ (1 : 1), 0 °C, 57% (**21a**), 68% (**21b**), **c** [(**21a** or **21b**), SOCl_2_, 95 °C] or [(**21c** or **21d**), DIPEA, DMAP, EDC, THF], then pentachlorophenol (PCP), THF, 25 °C, 51% (**22a**), 51% (**22b**), 12% (**22c**), 18% (**22d**).

To rapidly generate derivatives of albicidin, we adhered to our previously established convergent synthesis strategy^26^ and modified or improved it when necessary. We were able to modify each of the five different phenyl rings (Fig. 2). Starting with the A-building block from the commercially available aldehydes **16a** and **16b** (Scheme 1), we used a Horner–Wadsworth–Emmons reaction to give the desired coumaric acid ester isomers with exclusive (*E*)-selectivity, which were directly saponified to the acids **18a** and **18b** in good yields. The further steps towards the AB-PCP ester (**22a–b**, Scheme 2) were carried out in analogy with the synthesis variations of the B-fragment (see ESI†): there, acetyl-protected coumaric acid **19** (see ESI†) was coupled *via* the acid chlorides with the amine **20c** or **20d**. After removal of both protecting groups, the methyl ester and the acetyl group, the hydroxy group was again re-protected with Boc to obtain **21c** and **21d**, respectively. To avoid possible by-products in the further coupling which has been observed by using HATU as coupling reagent we prepared the active esters **22c** and **22d** instead.

**Scheme 3:**
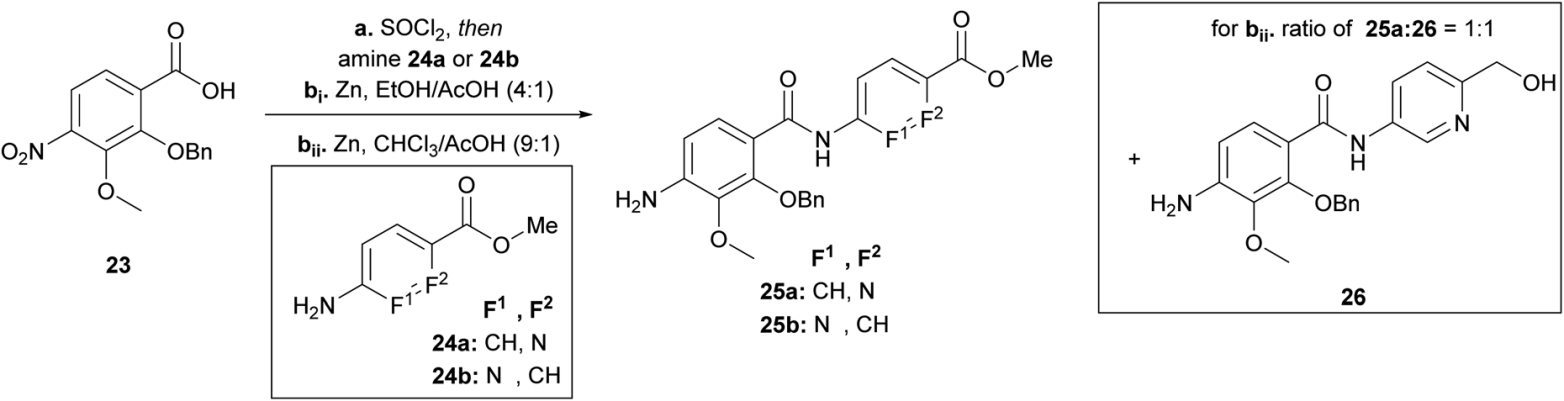
Synthesis of the EF-building block for F-variations. Conditions: **a** SOCl_2_, 95 °C, then amine **25a** (80%) or **25b** (79%), NEt_3_, THF, 0 °C → 25 °C; **bi** Zn, EtOH/AcOH (4 : 1), 0 °C → 25 °C, **25a** (30%), **25b** (96%), **26** (32%); **bii** Zn, CHCl_3_/AcOH (9 : 1), 0 °C → 25 °C, **25a** (96%).

Having the N-variations of the AB fragments in hand for the final coupling with an already synthetically established C–D–E–F fragment, we focused on preparing the N-substituted EF variations. One of our key building blocks, compound **23** (Scheme 3), was synthesized in 6 steps^23^ and then coupled *via* the acid chloride with heterocycles **24a** and **24b**, respectively, to obtain dipeptides with a nitroaryl group, which were subsequently reduced to **25a** and **25b** (Scheme 3). While the reduction to dipeptide **25b** was uncomplicated, a by-product was observed during reduction of the picolinic acid dipeptide: the methyl ester was reduced to the alcohol **26** yielding a 1 : 1 ratio, with a yield of 30% of the desired product (**25a**). We could enhance the yields by changing the solvent from ethanol to chloroform yielding **25a** with a yield of 96%.

**Scheme 4:**
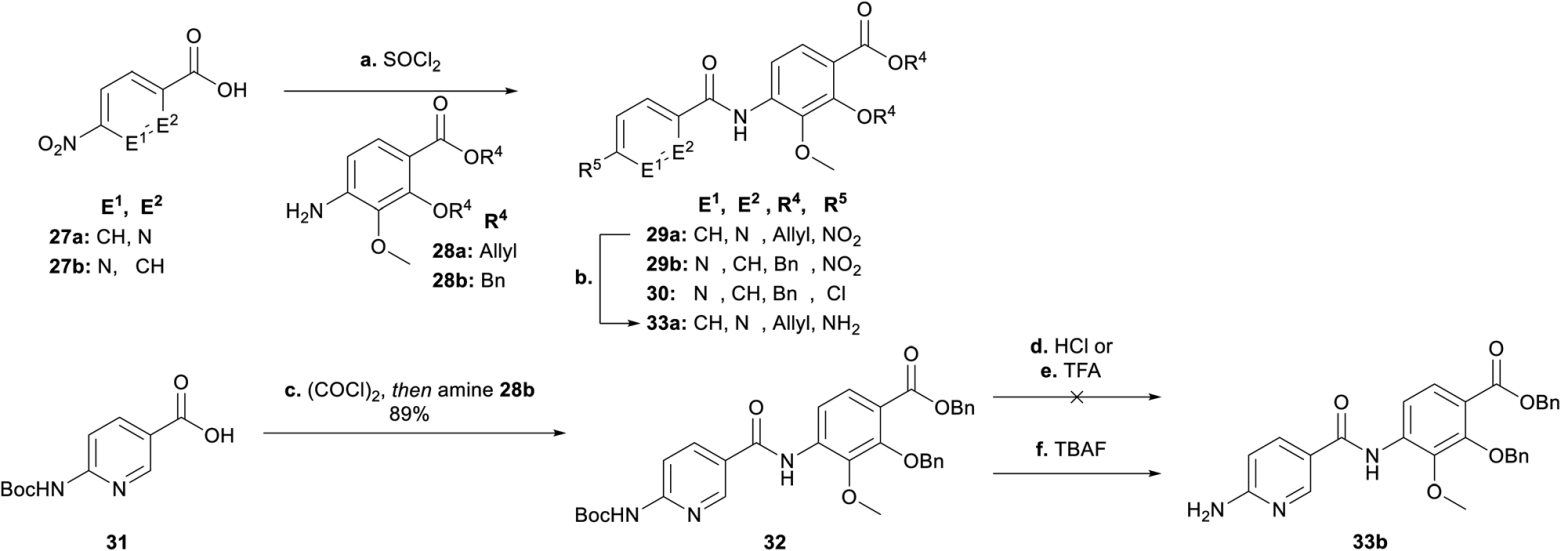
Synthesis of the EF-building block for E-variations. Conditions: **ai27a**, SOCl_2_, 95 °C, then **28a**, NEt_3_, THF, 0 °C → 25 °C, 89% (**29a**); **aii27b**, SOCl_2_, 95 °C, then **28b**, NEt_3_, THF, 0 °C → 25 °C, 11% (**29b**), 25% (**30**); **b** Zn, AcOH/EtOH (1 : 4), 0 °C, 92% (**33a**); **c** (COCl)_2_, THF, cat. DMF, 0 °C → 25 °C, then **28b**, NEt_3_, THF, 0 °C → 25 °C, 89%; **d** HCl 4 N in dioxane; 0 °C; **e** TFA/CH_2_Cl_2_ (1 : 4), 0 °C; **f** TBAF, THF, 120 °C, 79% (**33b**).

Next, we focused on the variation of the E-building block (Scheme 4). For the picolinic acid **27a** the transformation to the acid chloride worked well. This was followed by coupling with **28a** and reduction to **33a**. However, coupling with nicotinic acid **27b** decreased the yield of **29b** dramatically, due to the formation of the by-product **30** coming from a nucleophilic substitution of the nitro group by a chlorine. Previously, similar reactions have been reported in literature (see ESI†).^30^ We decided at this point for an alternative approach with a Boc-protected amine group (**31**) replacing the nitro group (**27b**). Knowing that the Boc group would be cleaved under harsh acidic activation conditions (SOCl_2_), we switched to oxalyl chloride in a ratio of 1 : 1.2 (**31** : (COCl)_2_) at 0 °C, to obtain dipeptide **32**. Removal of the Boc group with hydrochloric acid and trifluoro acetic acid also cleaved the benzyl ester to give corresponding carboxylic acid **34** (see ESI†), whereas TBAF rendered the desired product **33b**.

**Scheme 5:**
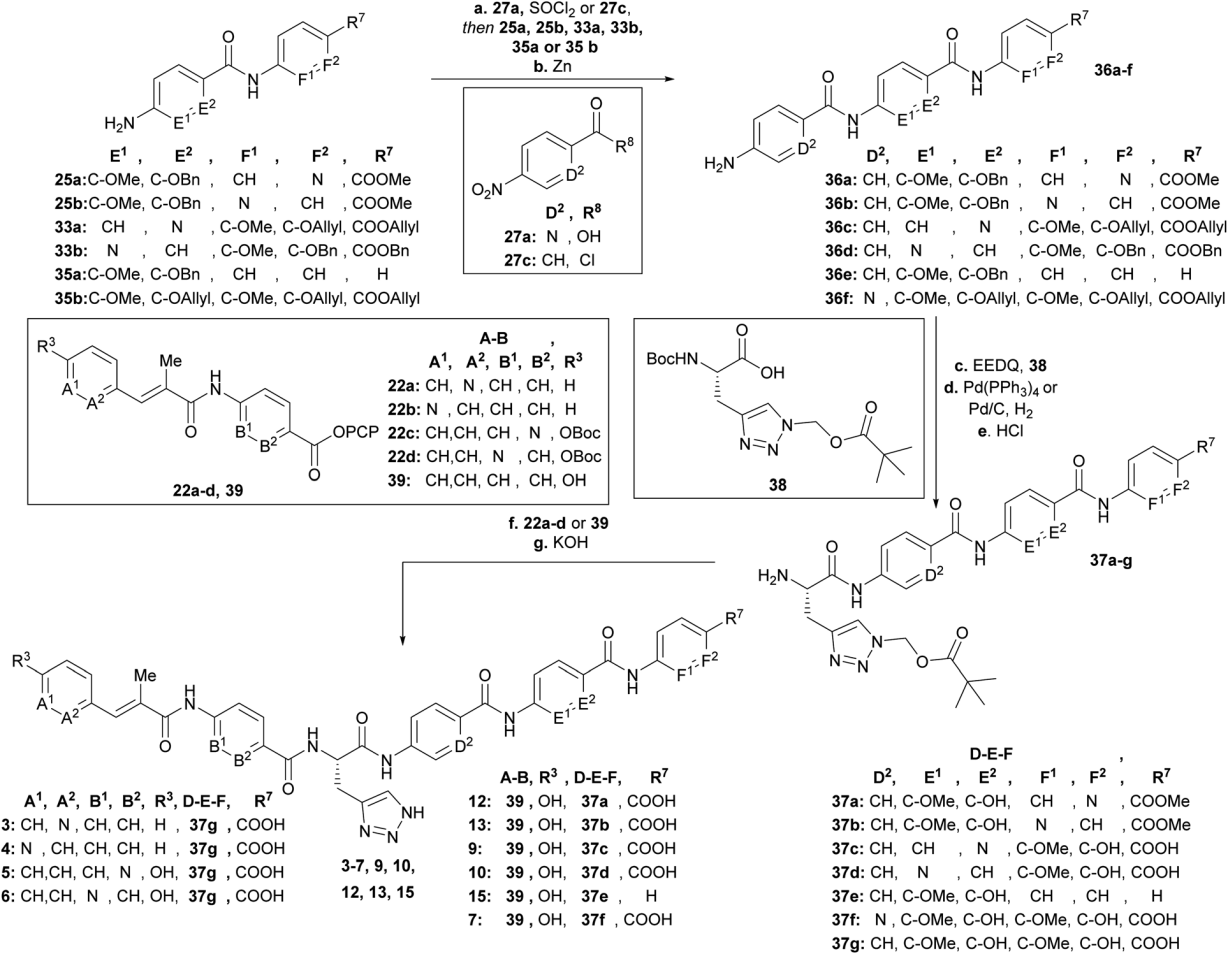
Synthesis of final derivatives by assembling of the building blocks. Conditions: **a** (SOCl_2_, 90 °C **27a** then **35b**, DIPEA) or [**27c** and (**25a**, **25b**, **33a**, **33b** or **35a**), NEt_3_], THF, 0 °C → 25 °C; **b** Zn, EtOH/AcOH (4 : 1) or CHCl_3_/AcOH (9 : 1), 0 °C, 2 steps: 82% (**36a**), 54% (**36b**), 41% (**36c**), 32% (**36d**), 76% (**36e**), 75% (**36f**); **c** EEDQ, **38**, THF, 25 °C; **d** Pd(PPh_3_)_4_, morpholine, THF or Pd/C, H_2_, EtOH/THF/MeOH (1 : 1 : 1); **e** 4N HCl, dioxane, 25 °C, 3 steps: 65% (**37a**), 66% (**37b**), 20% (**37c**), 53% (**37d**), 51% (**37e**), 37% (**37f**); **f** (**37a–f**, **39**) or (**37g**, **22a–b**) or (first **22a** or **22b**, TFA/CH_2_Cl_2_ (1 : 1), 0 °C, then **37g**), NEt_3_, DMF, 25 °C, g 3N KOH, DMF, 25 °C, 2–3 steps: 15% (**3**), 25% (**4**), 8% (**5**), 12% (**6**), 23% (**7**), 16% (**9**), 12% (**10**), 5% (**12**), 8% (**13**), 15% (**15**).

After having all variations of EF building blocks (**25a–b**, **33a–b**, **35a** (for synthesis of compound **15**, see ESI†), **35b** (ref. 23)) in hand (Scheme 5), the assembly of the tripeptides was carried out: this was performed either by coupling with benzoyl chloride (**27c**) or picolinic acid (**27a**)/SOCl_2_. The nitroaryl peptides were reduced with zinc to yield **36a–f**.

Next the AzaHis building block C (**38**)^27^ was coupled with the tripeptides **36a–f** (Scheme 5) and subsequently the allyl (**36c**, **36f**)− or benzyl (**36a**, **36b**, **36d**, **36e**) protecting groups were removed with tetrakis (triphenylphosphine)palladium(0), or Pd/C respectively. The remaining Boc groups of all tetrapeptides were then cleaved under acidic conditions to obtain the tetrapeptides **37a–f**, ready for the final coupling with the AB building block. Finally, we used either AB-PCP ester **39** (ref. 28) or PCP-esters **22a–d** (Scheme 2) for coupling with the tetrapeptides **36a–f**. Literature-known tetrapeptide **37g**,^28^ was prepared in analogy to the C–D–E–F fragments **37a–f**, for coupling with the different AB-PCP esters **22a–d**. The pivaloyloxymethyl (POM) protecting group on the C part was removed under basic conditions (KOH, DMF/H_2_O (1 : 1)) to obtain the full-length derivatives **3–7**, **9**, **10**, **12**, **13** and **15**.

Having managed to overcome several synthetic issues, we then tackled the synthesis of C-terminal tripeptide analogues of compound **8** (Fig. 2). The coupling with the *para*-amino nicotinic acid in building block D was extremely challenging. The formation of the corresponding tri- and tetra-peptide was achieved in only low yields. The coupling to give the final derivative was highly inefficient and additionally the final derivative (**8**) was found to be chemically unstable during the purification process. Thus, further attempts on the synthesis and antibacterial profiling of **8** were not continued.

### Antibacterial activity and physicochemical characterization of N-aryl substituted albicidins

Compounds **3** and **4** represent the N-terminal part of the pyridine-N-scan, namely building block A. As it has been previously shown for variations in building block A^24^ aromaticity is important to retain antibacterial activity, otherwise a number of structural variations were allowed. Results from the antibacterial assays showed that compounds **3** and **4** (Table 1) with pyridine replacing the hydroxy-phenyl in the cinnamoyl fragment, exhibited similar antimicrobial activities regardless of the position of the heterocyclic nitrogen. Both compounds however showed lower activities compared to the parent compound AzaHis-albicidin **2**. While the activities of **3** and **4** against Gram-negative strains are only an order of magnitude lower, there is extremely low activity against Gram-positive strains. This observation is in line with the previously shown importance of the para-hydroxy substitution on the building block A of albicidin and the observation that variations of A basically led to no increase in bioactivity.^24^ The phenyl to pyridyl exchange in building block B leading to compounds **5** (picolinic acid) and **6** (nicotinic acid), showed that both compounds have very good antibacterial activity, which is fairly comparable to the activity of compound **2** (Table 1).

**Table 1:** MIC data for selected strains for synthesized variations (**3–10**, **12**, **13**, **15**) and for reference compounds marked with* (**2**, **11**, **14**)

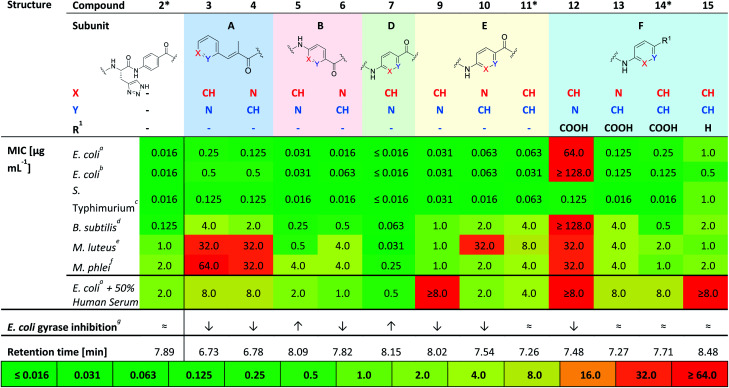

a DSM 1116.

b BW25113.

c TA100.

d DSM 10.

e DSM1790.

f DSM 750,

g Gyrase inhibition at a constant concentration of 45 nM of compounds **1** and **2−8**: ↑, for stronger inhibition; ≈, for equal inhibition; ↓, for diminished inhibition compared to albicidin **1** (see ESI Fig. S1).

The next test set of compounds comprises pyridyl variations in building blocks E and F: interestingly, compound **9**, with the picolinic acid in building block E shows very good bioactivity against all Gram-negative strains. This observation can be compared with activities for compound **10** (nicotinic acid), for which there is however very poor activity against one bacterial strain (*M. luteus* 32 μg mL^−1^). Comparison of **9** and **10** to compound **11** (ref. 28) (with *p*ABA in building block E) shows that the N-substitutions lead to a slight but measurable enhancement of antibacterial activity against Gram-positive bacteria, especially in the case of derivative **9**.

Next, we investigated variations in building block F. As we had found out in our previous work,^28^ replacing building block F with unsubstituted *p*ABA (compound **14**), resulted in an antibacterial activity that was comparable to compound **2**. Removal of the C-terminal carboxy group gave **15** which only displayed moderate antimicrobial activities against Gram-negative and Gram-positive organisms. This points to the significance of this functional group for potent inhibitory activity. Replacing the F building block with *para*-amino picolinic acid (compound **12**) and *para*-amino nicotinic acid (compound **13**) gives compounds, which show strikingly different antimicrobial properties (Table 1). For compound **12** the activity loss is nearly total with MIC values of >32 μg mL^−1^ for both Gram-negative (except for *S. typhimurium*) and Gram-positive strains. Compound **13** shows better MIC values (0.125 μg mL^−1^) against the Gram-negative strains and a slightly better activity (4 μg mL^−1^) against the Gram-positive strains. Overall, the bioactivity of **13** is comparable to that of the previously reported compound **14** (Table 1) with a loss of activity for Gram positive strains.^28^ Interestingly, it is noticeable that the change of the position of the heterocyclic nitrogen from position 2 to position 3 in building block E has a smaller impact on MICs than the analogous change in building block F.

Finally, with compound **7** building block D was assessed: This derivative with the pyridyl in building-block D (nitrogen *ortho* to carboxylic acid) demonstrates an excellent antimicrobial activity against all tested Gram-negative and also Gram-positive strains including *M. luteus* (0.031 μg mL^−1^) and *Mycobacterium phlei* (0.25 μg mL^−1^), which is more than two orders of magnitude superior to that of the parent compound **2** and all other tested derivatives. Interestingly, the MIC data against *E. coli* are mostly paralleled by *in vitro* inhibition data on *E. coli* gyrase (Table 1 and ESI Fig. S1;† relative comparison to albicidin **1**) which particularly confirms the better performance of compound **7** with regard to antibacterial activity.

Compound **2** was representatively probed for development of bacterial resistance on a narrow selection of bacterial strains. The formation of resistance (FoR) observed for the tested organisms were low (10^−10^ to 10^−11^ range at eight times MIC (see ESI Table S3†). The value of 10^−11^ for *E. coli* is lower compared to representative literature values obtained for the reference drug ciprofloxacin (10^−9^ at 16 times MIC), and points to a desirably low development of resistance by mutations.^31^

In summary, from this initial antibacterial profiling we have found that the phenyl to pyridyl exchange plays a more significant role for the C-terminal half of the molecule. Moreover, the position of the heterocyclic nitrogen in the C-terminal building blocks F, E and most significantly, building block D plays a key role in the bioactivity of the albicidin derivatives. Thus, we identified compound **7** as the most active in our primary screens.

Therefore, **7** was subjected to a follow-up antibacterial profiling against a panel of clinically relevant and ciprofloxacin (CIP)-resistant pathogens (Table 2).

**Table 2:** Extended panel for antimicrobial characterization of compound **7** in comparison to albicidin **1** and analogue **2**

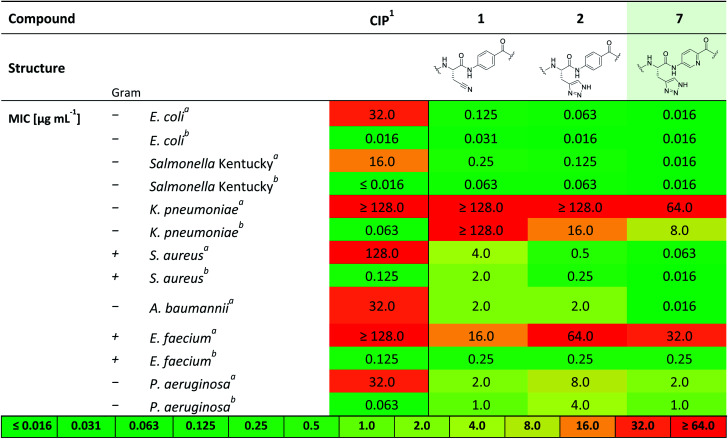

a CIP = ciprofloxacin.

b Ciprofloxacin resistant isolate.

c Ciprofloxacin susceptible isolate, additional strain information is given in ESI.

Compound **7** shows excellent activity against CIP-resistant strains of *E. coli* (4 times higher compared to **2**), *Salmonella* Kentucky (8 times higher compared to **2**), *S. aureus* (8 times higher compared to **2**) and *A. baumannii* (125 times higher compared to **2**), as well as the CIP-sensitive strains of these bacteria. Moreover, **7** shows improved, yet moderate activities against CIP sensitive strains of *K. pneumonia* and *P. aeruginosa* (8 and 4 times higher than **2**, respectively). We further investigated the biological activity of **7** against a panel of gyrase- and topoisomerase-deficient isolates of *E. coli* GK571 and against selected *E. coli* knockout mutants from the KEIO collection (Table S2†). The potent antimicrobial activity of compound **7** against *E. coli* was not affected by quinolone resistance-mediating mutations (0.031–0.016 μg mL^−1^, 10-fold higher than **1** and **2**). In comparison, the antimicrobial activity of cystobactamid analogue **CYS-22** (Fig. S1, SI†) was previously reported to be 0.5 μg mL^−1^ on gyrase-deficient *E. coli* WT-3-1 [*gyrA* (S83L, D87G)].^32^ In addition, we observed a weak effect of the TolC (efflux transporter) knockout mutation on the antimicrobial activity of **7**. The efficacy increased by a factor of 4 from 0.063 to ≤0.016 μg mL^−1^ presumably due to the impaired efflux mechanisms of the strain. In contrast, the knockout mutation of the *tsx* gene resulted in a 10-fold decrease in activity caused by a diminished compound uptake by the bacterial cells (Table S2†).^33,34^

This investigation was completed by assessment of the antibacterial activity in presence of human plasma (50%) (Table 1), since high plasma protein binding can severely impair antibacterial activity and thus efficacy. Remarkably, with an MIC 0.5 μg mL^−1^ against *E. coli* DSM 1116 compound **7** also showed the strongest activity in presence of plasma, which is superior to all other candidate molecules.

With this strong antibacterial profile in hand, we determined the kinetic solubility and logD values for **7** in comparison to albicidin **1** and AzaHis-albicidin **2**. Interestingly, with a solubility of 14.2 μg mL^−1^ for **7** and 14.8 μg mL^−1^ for **2**, both synthetic compounds are roughly three times more soluble in phosphate buffer (pH 7.5, DMSO f.c.: 5%) than albicidin (5.53 μg mL^−1^) (Table S3†). The logD_7.4_ 1.63 of compound **7** is higher than that for the parent compound **2** (logD_7.4_ 1.06) but still in a reasonable range <5. For the last step of *in vitro* profiling, we examined plasma half-life in both human and mouse plasma. We found a half-life (*t*_1/2_) for **1** of 60 min in human and 30 min in mouse plasma, respectively. Half-life was found increased to >240 min in human plasma and 95 min in mouse plasma for **2** and **7** which is sufficient for the subsequent progression into an animal model. Hemolysis and cell proliferation determined with HepG2 cells were in support of the conductance of *in vivo* experiments (see ESI, Table S3 and Fig. S2†).

### Computational analysis

Intrigued by the striking bioactivity differences observed in the various pyridine substituted derivatives, we performed a computational structural analysis with and without incorporation of heterocyclic nitrogen into the albicidin structure at various sites. Some molecular dynamics (MD) calculations have been previously applied to the truncated cystobactamids^35^ and to a full cystobactamid derivative.^36^ However, structural investigation of albicidin or its analogues using density functional theory (DFT) has never been reported. We aimed at conducting systematic structural investigations using state of the art DFT methods. For this purpose, the GFN-xTB tight-binding method was used for initial conformational searching in water employing the GBSA solvation model. The structures were fully optimized at the B3LYP CPCM (water) level of theory, with the BJ-damped DFT-D3 dispersion correction and 6-311+G (d,p) basis set (see ESI† for details). Analytical frequencies were calculated to verify the optimized geometries as minima (with no imaginary frequency).

Interestingly, the optimized geometry of **2** (ESI Fig. S3†) reveals a hairpin-like folded structure of the albicidin core. The molecule is folded *via* a twist around the sp^3^ carbon of the central AzaHis amino acid (building block C) and forms a *cis*-amide bond between C and D, resulting in the C-terminal and N-terminal “arms” facing each other. A similar structure was described for cystobactamid by Kirschning and co-workers using MD simulations.^36^ Hairpin-like structures of similar type have been reported in naturally derived (*e.g.* gramicidins^37^) and peptidomimetic drugs such as plantazolicin A^38^ and protegrin I^39^ and others.^40^ Furthermore, aromatic oligoamide foldamers have been constructed using a rigid turn unit into β-sheet hairpin-like structures stabilized by π-stacking interactions.^41^ For our part, we did not find experimental evidence by NOESY NMR spectroscopy for these structures, and conclude that the proposed hair-pin structure has to be taken with some reservation and should be studied in more detail in the future.

**Fig. 3:**
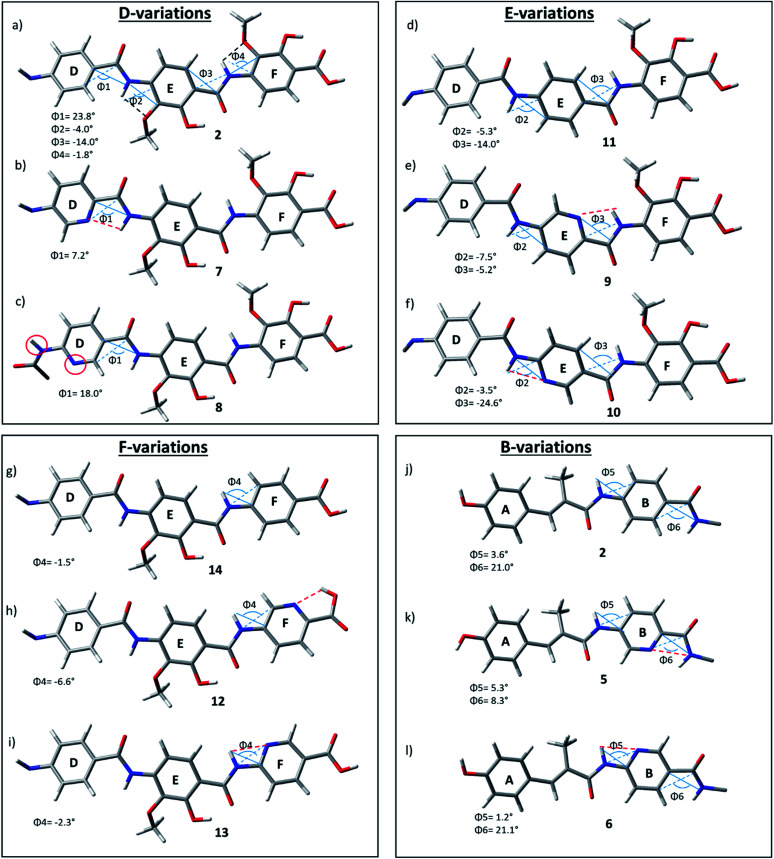
D–F fragments of derivatives **2 (a)**, **7–14** (b–i) and A–B fragments of derivatives **2 (j)**, **5 (k)**, **6 (l)**, extracted from optimized geometries of the corresponding full albicidin derivatives.

As the D–E–F fragment of the C-terminal part of albicidin shares its main structural features with aromatic oligoamides (AOA),^42^ it is to be expected that albicidin might show coordination patterns similar with AOA and its geometrical structure can be analysed in the same way. It has been shown that the incorporation of hydrogen acceptors, such as alkoxy substituents or a heterocyclic nitrogen into the aromatic rings of the AOA scaffold results in the formation of intramolecular hydrogen bonds (IMHB), which are responsible for conformational restrictions leading to a number of molecular architectures.^43^ Especially the ether oxygen of the lipophilic methoxy groups as H bond acceptor is important for the overall geometry. As expected, the calculated geometry of the D-E–F fragment of **2** (Fig. 3a reveals IMHB between the oxygen of the aryl methoxy substituents of building blocks F and E and the neighbouring amide hydrogens (shown with black dashed lines), with a distance of 2.1 Å which is in the common range of O–H hydrogen bonding. The corresponding MeO–C=C–N–H dihedral angles of **2** (Fig. 3a) are essentially planar with Φ2 = −4.0° and Φ4 = −1.8°.

In contrast to the N-terminal dihedral angles, the C-terminal angles of **2** (Fig. 3a, Φ1 and Φ3) are substantially twisted (23.8° and −14.0° respectively). Introduction of picolinic acid in building block D (derivative **7**) provides an additional IMHB between the ring nitrogen and the amide hydrogen (Fig. 3b, red dashed line), similar to the IMHB observed for the truncated (tri-peptide) cystobactamid 507 analogues.^35^ The formation of the additional N–H hydrogen bond in **7** results in a dihedral angle Φ1 = 7.2° (Fig. 3b) which is 16.6° smaller compared to the analogous angle in **2** (Φ1 = 23.8°) and leads to a planarization of the C-terminal part. In the resulting structure, the three aromatic rings of building blocks D, E and F are aligned in a slightly distorted linear structure (Fig. 3b). (Table 3)

**Table 3:** Calculated angles Φ1–Φ6 for compounds **2–14**, Φ color-code: reference compound **2** (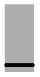), shown in Fig. 3 angle decrease/increase >5° (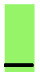)

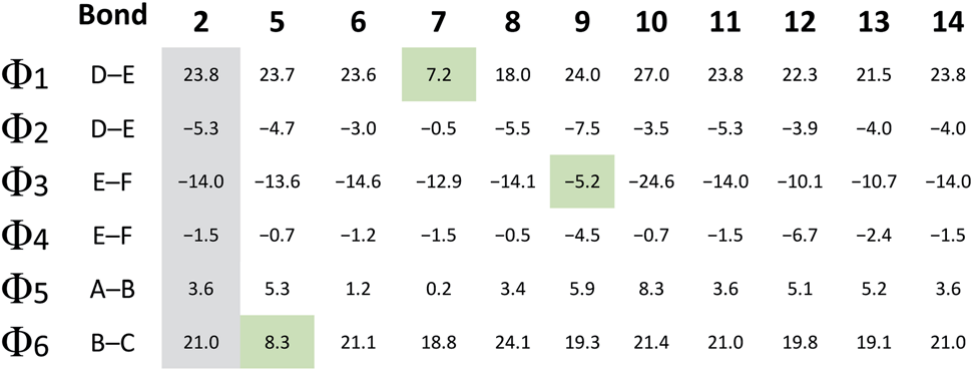

For the derivatives containing pyridine in building block B (**5**, **6**), we observe an adverse effect. For compound **5**, the formation of an additional IMHB results in a significantly smaller dihedral angle Φ6 = 8.3° (Fig. 3k) *versus* Φ6 = 21.0° (parent compound **2**, Fig. 3j). This local geometrical change, however, has no significant effect on the planarity of the entire molecule since it is located next to the sp^3^ amino acid of building block C and is not connected directly with the C-terminal arm. For compound **6** the change in angle is much smaller with Φ5 = 1.2° (Fig. 3l), which is only 2.4° narrower than the corresponding angle in **2** (Fig. 3j, Φ5).

Since albicidin (**1**) and AzaHis-albicidin **2** contain substituted *p*ABA in building blocks E and F, it would be more accurate to compare the geometry of the pyridine substituted compounds in E (**9**, **10**) and F (**12**, **13**) with the *p*ABA derivative in E (**11**) and *p*ABA derivative in F (**14**) respectively. Curiously, when replacing building block E with *p*ABA (compound **11**, Fig. 3) the dihedral angle Φ2 is only slightly affected (Φ2 = −5.3°) implying that the planarity of the DEF fragment is not determined by the MeO substitution in building block E. The same is true for the compound with *p*ABA in F (**14**, Fig. 3g) where angle Φ4 is essentially not changed upon removal of the MeO substituent (Φ4 = −1.5° in **14** (Fig. 3g) *vs.* Φ4 = −1.8° in **2** (Fig. 3a)). A similar trend is observed for compounds **12** and **13**, where the dihedral angle of parent compound **14** (Φ4 = −1.5) is already small (Fig. 3g–i). The picolinic acid derivative **12** stands out in our range of pyridine containing derivatives since the heterocyclic nitrogen located at the C-terminal position is not suitable for the formation of IMHB with the amide hydrogens. Compound **12** is even more intriguing concerning its complete loss of antimicrobial activity compared with compounds **2** and **14** (Table 1). The heterocyclic nitrogen in the picolinic acid unit coordinates with the acidic hydrogen of the carboxylic acid (Fig. 3h, red dashed line) with the distance of 1.94 Å between the heterocyclic nitrogen and the acidic hydrogen thus altering the C-terminal properties of the albicidin molecule. Picolinic acid is more acidic than benzoic acid by 3.2 p*K*_a_ units. The increased acidity might play an additional role in the loss of activity in **12** compared with **14**.

In summary, using DFT methods we have shown that the oligoaromatic albicidin scaffold is strongly impacted by IMHB through the introduction of heterocyclic nitrogens. In this context, the placement of picolinic acid renders a much stronger planarization effect through IMHB formation than in the nicotinic acid derivatives. This effect is the largest for building block D (compound **7**) resulting in the planar alignment of the C-terminal tripeptide fragment and thus possibly influencing the antimicrobial potency of **7**. This information is of high importance, since it enables us to rationally design further albicidin analogues with a local incorporation of IMHBs which should improve the SAR profile. Once the mode of action and the molecular binding site of albicidin are elucidated, these data can become a game changer in the design and synthesis of novel potent alibicidin derivatives.

### Pharmacokinetics and pharmacodynamics of albicidin analogues **2** and **7**

**Fig. 4:**
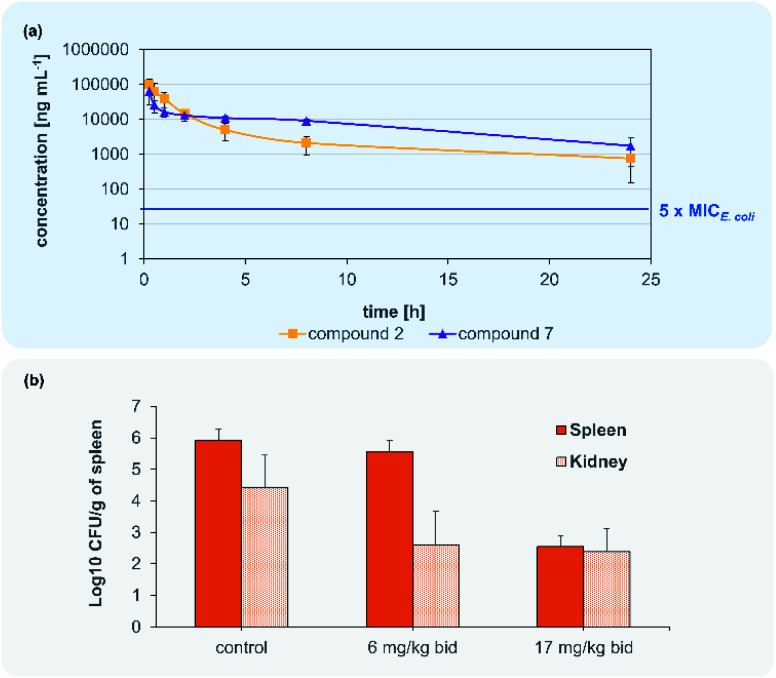
Blood concentration of **2** and **7** after 50 mg kg^−1^ i.v. administration (a). Murine septicaemia model for the i.v. administration of **7** to CD-1 mice infected with a fluoroquinolone-resistant (FQR) *E. coli* isolate infected (b).

Encouraged by the antimicrobial *in vitro* results, the *in vivo* profile of compound **2** and **7** was then examined. The optimized compound formulation for *in vivo* experiments was established: From this work, a clear solution of analogues **2** was observed for a formulation mixture of DMSO/PEG400 (10 : 90) (Table S3†), which later was also applied for compound **7**. In a first step, a tolerability study was conducted with albicidin analogue **2** in adult male CD-1 mice. Doses of 12.5, 25 and 50 mg kg^−1^ were administered twice at *t* = 0 h and *t* = 12 h by intravenous (i.v.) application route with a volume of 5 mL kg^−1^ (*n* = 3 per dose). No adverse effects and signs indicating pain were noticed throughout 24 hours. Blood cell count and macroscopic necropsy of inner organs did not show any pathological modifications (Table S4†). We then subjected both compounds **2** and **7** to a pharmacokinetic analysis. For each compound, a single dose of 50 mg kg^−1^ was administered i.v. in 5 mL kg^−1^ to three male CD-1 mice. Samples of plasma were collected at designated time points and analysed for the compound concentration levels by HPLC-MS/MS (Fig. 4, Table S7†). Subsequently, PK parameters were calculated using a two-compartment model (Table 4).

**Table 4:** Pharmacokinetic properties for **2** and **7** after i.v. administration of 50 mg kg^−1^ compared to the cystobactamid analogue **CYS-22**.^32^

PK parameter	**2**	**7**	**CYS-22**
	50 mg kg^−1^	50 mg kg^−1^	5 mg kg^−1^
AUC [ng mL h^−1^]	153 320.0 ± 47 239.0	224 852.0 ± 49 029.0	2087.0 ± 573.1
*T*_1/2_ [h]	1.2 ± 0.6	1.373 ± 1.5	0.98 ± 0.1
*V*_Z_ [L kg^−1^]	4.9 ± 5.2	2.7 ± 1.1	3.5 ± 1.2
CL [mL h^−1^ kg^−1^]	352.4 ± 128.6	229.2 ± 47.5	2490 ± 684

Accordingly, compounds **2** and **7** attained plasma levels in amounts higher than 5-times MIC for *E. coli* (=16 ng mL^−1^ for compound **7**) even after 24 h (Fig. 4). By comparing the plasma level after 8 h of our lead compound **7** (i.v. 50 mg kg^−1^) with the lead compound **CYS-22** from Testolin *et al.*^32^ (i.v. 5 mg kg^−1^, 8 h) to our delight we found a much higher (about 2000-fold) plasma concentrations of 100 μg mL^−1^ for 7 compared to 0.05 μg mL^−1^ for **CYS-22**), even taking into account a 10-fold higher dosing of **7** compared to **CYS-22**. This much higher plasma concentrations resulting mainly from the 7- to 10-fold lower clearance rate compared to compound **CYS-22** leads to >10-fold higher plasma concentration after 24 h above the microbiological important 5-fold MIC-value. This overcomes the need for repeated and complicated dosing regimen to achieve satisfactory plasma levels over the anticipated whole therapeutic treatment period. Importantly, no adverse effects were observed on the tested mice at the used dose of 50 mg kg^−1^. The full set of pharmacokinetic parameters are found in the ESI (Table S8†).

Following the encouraging pharmacokinetic results, we then proceeded to an *in vivo* efficacy study in a murine septicaemia model. We decided to further follow compound **7** in the *in vivo* infection model, due to the better antibacterial activity in presence of plasma and the pharmacological parameters allowing a bi-daily dosing of the test compound. From a panel of clinical ESKAPE isolates one fluoroquinolone (FQR)-resistant *E. coli* strain was selected for the preparation of the infection model (Table S3†). A solution of **7** was administered intravenously to 6 weeks old CD-1 male mice infected with a ciprofloxacin resistant *E. coli* clinical isolate (MIC = 0.031 μg kg^−1^) in two doses of 6 mg kg^−1^ (12 mg per kg per day) and 17 mg kg^−1^ (34 mg per kg per day) at *t* = 2 h and *t* = 12 h post infection. All mice infected with the lethal dose of FQR resistant bacteria survived after 24 h while treated with doses of 6 and 17 mg kg^−1^ injected after 2 h and 10 h. Bacterial payload (CFU) was measured from spleens and kidneys and compared to untreated control animals. Compound **7** showed significant reduction of bacterial counts (>3 log 10 CFU g^−1^) in kidneys in both tested doses (Fig. 4). The dose of 17 mg kg^−1^ of compound **7** indicates a main elimination of the unmetabolized compound *via* the kidney.

## Conclusion

In summary, we have performed a systematic synthesis and SAR study for the replacement of the phenyl by pyridines in the aromatic building blocks of albicidin. Such replacements commonly are performed in regions which are not necessarily pharmacophore regions of the molecule. Following our study, we succeeded in obtaining a novel albicidin derivative **7** with a pyridine as building block D, of unprecedented antibacterial activity against a microbial panel of clinically relevant multi-resistant bacteria clearly showing the broad spectrum of activity of this new analogue of albicidin. The best antibacterial potency was observed towards Gram-negative *E. coli* and Gram-positive *S. aureus* FQR isolates. We are delighted to report a breakthrough regarding the activity in presence of plasma, and *in vitro* plasma stability. The tolerability study in mice by intravenous injection of a dose of 50 mg kg^−1^ of compound (**7**) did not show any adverse effects or histopathological observations which provides evidence very promising safety profile. The pharmacokinetic data obtained after intravenous injection with a safe dose of 50 mg kg^−1^ in mice revealed a bi-phasic elimination of compound **2** and **7** suggesting a 2-compartmental distribution in the body. In the *in vivo* proof-of-concept experiment, treating animals infected with a lethal dose of FQR clinical isolate (BSL2) *E. coli* at a dose of 17 mg kg^−1^ compound **7** applied twice, all mice survived. The CFU reduction in the infected isolated organs was more than 3-log units in spleen and 2-log units in kidney. In summary, the pharmacokinetic and pharmacodynamics testing of albicidin analogue **7** has clearly demonstrated efficacy and revealed **7** as a lead structure for a novel antibiotic drug. After this successful target validation in a rodent animal model the broader anti-bacterial testing towards WHO priority 1 and 2 clinical isolates of these new albicidin analogues will set the stage for a full pre-clinical profiling. This will be paralleled by MOA studies and a further thorough understanding of albicidin resistance mechanisms as a prerequisite for the pre-clinical and clinical development of new antibiotic therapies addressing AMR.

## Data availability

Experimental procedures and analytical data are availiable in the ESI.†

## Author contributions

LZ and LK designed and synthesized albicidins analyzed the data and wrote the manuscript. LvE designed and synthesized albicidins (**5**, **6**). MS was responsible for biological testing and wrote the manuscript. AK developed the theory and performed the computations. FOG and JW aided in interpreting the results and worked on the manuscript. RDS supervised the project. All authors provided critical feedback and helped shape the research, analysis and manuscript.

## Conflicts of interest

FOG is employee of Gombert Pharma Research Solutions (GPRS) and shareholder of Selmod LLC. The other authors declare no conflict of interest. Patents are filed.

## Supplementary Material

SC-012-D1SC04019G-s001
