# Supplementary material for: Improvement of the antimicrobial potency, pharmacokinetic and pharmacodynamic properties of albicidin by incorporation of nitrogen atoms
Source: Chem Sci. 2021 Oct 19;12(43):14606–17. doi: 10.1039/d1sc04019g (PMC8580050; doi:10.1039/d1sc04019g)
Supplement: SC-012-D1SC04019G-s001 [file SC-012-D1SC04019G-s001.pdf]

Electronic Supplementary Material (ESI) for Chemical Science.
This journal is © The Royal Society of Chemistry 2021

Electronic Supplementary Material (ESI) for Chemical Science.
This journal is © The Royal Society of Chemistry 2021

Improvement of the antimicrobial potency, pharmacokinetic and pharmacodynamic properties of albicidin by incorporation of nitrogen atoms

Lieby Zborovsky[‡a], Leonardo Kleebauer[‡a], Maria Seidel[a], Arseni Kostenko[a], Leonard von Eckardstein[a], Frank Otto Gombert[b], John Weston[a], Roderich D. Süssmuth*[a]

[a] Institut für Organische Chemie, Technische Universität Berlin, Straße des 17. Juni 124, 10623 Berlin

[b] Gombert Pharma Research Solutions (GPRS), Dornacherstrasse 120, CH 4053 Basel

* To whom correspondence should be addressed. E-mail: suessmuth@chem.tu-berlin.de

[‡]These authors contributed equally to this work.

Table of Contents

1 EXPERIMENTAL PART 2

1.1 BIOLOGY 2
1.1.1 *In vitro* experiments 2
1.1.2 Tolerability assessment and pharmacokinetic study 7
1.1.3 Murine septicemia infection model 11

1.2 CHEMISTRY 13
1.2.1 Materials and methods 13
1.2.2 Experimental procedures 14
1.2.3 Synthesis of A-Variations (3, 4) 14
1.2.4 Synthesis of B-Variations (5, 6) 22
1.2.5 Synthesis of F-Variations (12, 13, 15) 30
1.2.6 Synthesis of E-Variations (9, 10) 51
1.2.7 Synthesis of D-Variations (7, 8) 70

1.3 PHYSICOCHEMICAL PROPERTIES ANALYSIS 86
1.3.1 Determination of kinetic solubility 86
1.3.2 Determination of logD values 86

2 COMPUTATIONAL METHODS 87

3 NMR SPECTRA 111
3.1 NMR spectra of A-Variations (3, 4) and reaction intermediates 111
3.2 NMR spectra of B-Variations (5, 6) and reaction intermediates 122
3.3 NMR spectra of F-Variations (12, 13, 15) and reaction intermediates 133
3.4 NMR spectra of E-Variations (9, 10) and reaction intermediates 161
3.5 NMR spectra of D-Variation (7) and reaction intermediates 184

4 REFERENCES 196

1 EXPERIMENTAL PART

1.1 BIOLOGY

1.1.1 *In vitro* experiments

Determination of Minimal inhibitory concentrations (MIC)

Minimal inhibitory concentration (MIC) values were determined according to the ninth edition of the Approved Standard M07-A9.[1]

20 µL of cryo stocks of bacterial strains were suspended in 20 mL LB medium (Lysogeny broth: 10 g L^{-1} peptone, 5 g L^{-1} yeast extract, 5 g L^{-1} NaCl). Following incubation overnight at 37°C, 200 rpm, the test inoculum was adjusted by 0.5 McFarland Standard in cation adjusted Mueller-Hinton broth (BBLTM Mueller-Hinton Broth II, Becton, Dickinson and Company, New Jersey, USA) to approximately $1x10^6$ CFU mL^{-1}. 95 µL of the bacterial inoculum were applied per well of a 96-well plate. Albicidin and albicidin derivatives **2** to **13** were dissolved in DMSO (100%) to an initial concentration of 2560 µg mL^{-1} and serially diluted in DMSO (100%). Initial solution and serial dilutions of Ciprofloxacin were prepared in 0.1 N HCL, respectively. 5 µL of each antibiotic dilution were added to the microdilution tray to reach final concentrations of 128 µg mL^{-1} to 0.016 µg mL^{-1} per well. One row of each well plate served as a growth control without antibiotic substances and another row of the microdilution tray served as sterility control (only MHB II-media). The antimicrobial effect of the solvent (DMSO) was tested by adding 5 µL DMSO to several wells. Purity check and cell titer control were performed according to International Standard M07-A9 on Mueller-Hinton II Agar (Mueller Hinton II Broth, 15 g/L agar-agar). Both microdilution trays and agar plates were incubated at 30-37°C for 16 h and subsequently analyzed for growth inhibition by naked eye.

The following primary panel of microorganisms were purchased from German Collection of Microorganisms and Cell Cultures (DSMZ):

Escherichia coli DSM 1116
Bacillus subtilis DSM 10
Micrococcus luteus DSM 1790
Mycobacterium phlei DSM 750
Salmonella typhimurium TA100 was kindly provided by Prof. Dr. Vera Meyer (Technische Universität Berlin).
Escherichia coli BW25113 was purchased as part of the single gene knockout library of *E. coli* K12 BW25113 (Keio collection) from Horizon Discovery group plc (Cambridge, UK).

The following extended panel of bacterial strains were and were kindly provided either by Prof. Dr. Brötz-Oesterheldt (Tübingen, Germany)/ Prof. Dr. Heisig (Hamburg, Germany)[2]

Escherichia coli WT (GK571)
Escherichia coli WT-3-1 (GK796) [*gyrA* (S83L, D87G)]
Escherichia coli WT-3-1-MB2 (GK927) [*gyrA* (S83L, D87G), *parC* (S80I)]
Escherichia coli MI (GK572) [gyrA (S83L)]
Escherichia coli MII (GK573) [*gyrA* (S83L), *marR* (Δ175bp)]
Escherichia coli MIII (GK574) [*gyrA* (S83L, D87G), *parC* (S80I), *marR* (Δ175bp)]
or by Prof. Dr. Werner (Robert-Koch-Institut, Wernigerode, Germany):
Escherichia coli (ciprofloxacin resistant clinical isolate)
Escherichia coli (ciprofloxacin susceptible clinical isolate)
Salmonella Kentucky (ciprofloxacin resistant clinical isolate)
Salmonella Kentucky (ciprofloxacin susceptible clinical isolate)
Klebsiella pneumoniae (ciprofloxacin resistant clinical isolate)
Klebsiella pneumoniae (ciprofloxacin susceptible clinical isolate)

Staphylococcus aureus (ciprofloxacin resistant clinical isolate)
Staphylococcus aureus (ciprofloxacin susceptible clinical isolate)
Acinetobacter baumannii (ciprofloxacin susceptible clinical isolate)
Enterococcus faecium (ciprofloxacin resistant clinical isolate)
Enterococcus faecium (ciprofloxacin susceptible clinical isolate)
Pseudomonas aeruginosa (ciprofloxacin resistant clinical isolate)
Pseudomonas aeruginosa (ciprofloxacin susceptible clinical isolate)
Escherichia coli deficient mutants were purchased as part of the single gene knockout library of *E. coli* K12 BW25113 (Keio collection) from Horizon Discovery group plc (Cambridge, UK).

In preparation for the *in vivo* infection study, susceptibility determination of compound **7** was carried out in cooperation with Atlantic Group for Research on Anti-Microbials (Atlangram, Nantes, France) for the following 11 pathogenic isolates:
Escherichia coli (ciprofloxacin resistant clinical isolate number 191950584)
Escherichia coli (ciprofloxacin resistant clinical isolate number 192132534)
Escherichia coli (ciprofloxacin susceptible clinical isolate ATCC 23716)
Klebsiella pneumoniae (ciprofloxacin resistant clinical isolate number 191830147)
Acinetobacter baumannii (ciprofloxacin resistant clinical isolate number 190022323)
Acinetobacter baumannii (ciprofloxacin resistant clinical isolate number 190550628)
Pseudomonas aeruginosa (ciprofloxacin resistant clinical isolate number 191261152)
Pseudomonas aeruginosa (ciprofloxacin resistant clinical isolate number 192392023)
Staphylococcus aureus (ciprofloxacin resistant clinical isolate number 192322258)
Staphylococcus aureus (ciprofloxacin resistant clinical isolate number 192190337)
Clinical and Laboratory Standards Institute (CLSI) microdilution technique was used for measurement of these MICs by using Ciprofloxacin and Ofloxacin as reference antibiotics.

Escherichia coli **Gyrase Supercoiling Inhibition Assay**

DNA supercoiling experiments with *E. coli* DNA gyrase were performed in a total volume of 30 µL. All components and protocol were purchased from inspiralis Limited, Norwich, UK. Each reaction mixture contained 0.5 µg relaxed pBR322 plasmid DNA, 1 U DNA-gyrase (6 U μL^{-1}) and gyrase buffer. The albicidins **1** to **15** were added to the mixture at a final concentration of 45 nM. The final DMSO concentration was 3%. Samples were incubated at 37°C (water bath) for 30 min and subsequently loaded on an agarose gel. Electrophoretic analysis was performed using a 1% agarose gel (100 V, 90 min). Subsequently, DNA bands were stained with ethidium bromide and visually analyzed.[3]

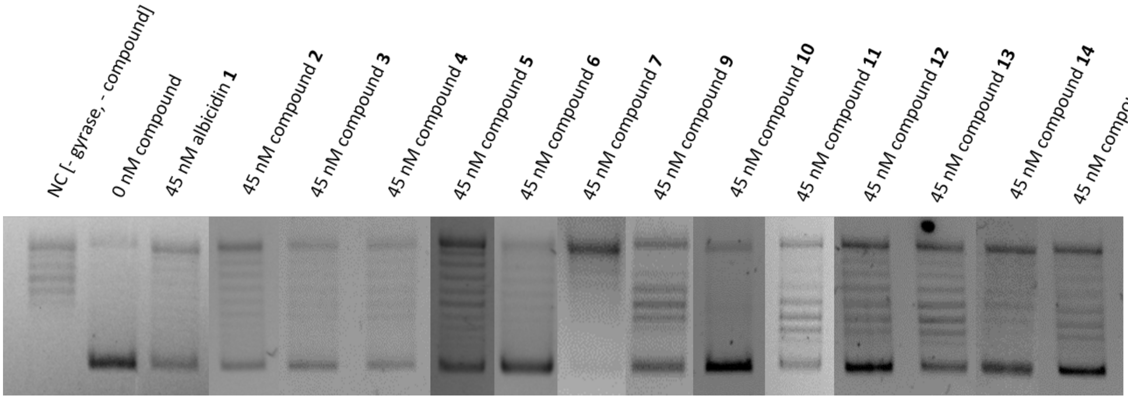

Figure S 1: Inhibition assay of the supercoiling activity of **E. coli** ***DNA gyrase for albicidin (1), and compounds 2-15. The control experiment without enzyme and drug (left lane) shows relaxed DNA. Addition of DNA gyrase results in supercoiled DNA (second lane from left). All derivatives were tested at a concentration of 45 nM.***

Supplementary antibacterial activity data

Table S 1. Biological characterization of compound 7 on extended panel of ESKAPE organisms prior to in vivo efficacy studies.

**Compound**			**CIP**[1]	**OFL**[2]	**7**
**Structure**					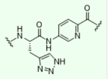
**MIC [µg mL⁻¹]**	*E. coli*[a]	191950584	≤ 0.016	0.031	0.031
	E. coli[b]	192132534	4.0	16.0	0.063
	E. coli[b]	ATCC 23716	16.0	16.0	0.031
	K. pneumoniae[b]	191830147	32.0	32.0	0.25
	S. aureus[b]	192322258	64.0	32.0	1.0
	S. aureus[b]	192190337	128.0	64.0	0.25
	A. baumannii[b]	190022323	64.0	64.0	≥ 128.0
	A. baumannii[b]	190550628	64.0	64.0	≥ 128.0
	E. cloacae[b]	192180419	4.0	4.0	8.0
	P. aeruginosa[b]	191261152	4.0	32.0	16.0
	P. aeruginosa[b]	192392023	4.0	32.0	16.0

[1] CIP = ciprofloxacin, [2] OFL = Ofloxacin, [a] ciprofloxacin resistant isolate,
[b] ciprofloxacin susceptible isolate, additional strain information is given in supplementary information

≤ 0.016	0.031	0.063	0.125	0.25	0.5	1.0	2.0	4.0	8.0	16.0	32.0	≥ 64.0

Table S 2. Biological activities of albicidin, analogs 2 and 7 on a panel of gyrase or topoisomerase IV deficient E. coli isolates and E. coli BW25113 deletion mutants.

**Compound**		**CIP**[1]	**1**	**2**	**7**
**Structure**			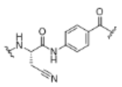	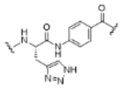	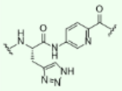
**MIC [µg mL⁻¹]**	*E. coli* WT[2] (GK571)[a]	0.031	0.063	0.063	≤ 0.016
	E. coli WT-3-1 (GK796)[b]	0.5	0.125	0.063	≤ 0.016
	E. coli WT-3-1-MB2 (GK927)[c]	32.0	0.125	0.25	0.031
	E. coli MI (GK572)[d]	1.0	0.25	0.25	≤ 0.016
	E. coli MII (GK573)[e]	2.0	0.5	0.25	≤ 0.016
	E. coli MIII (GK574)[f]	64.0	1.0	0.25	0.031
	E. coli BW25113	0.016	0.063	0.016	0.063
	E. coli BW25113 Δ*tolC*	0.063	0.063	0.031	≤ 0.016
	E. coli BW25113 Δ*acrA*	0.031	0.063	0.031	≤ 0.016
	E. coli BW25113 Δ*acrB*	0.031	0.063	0.031	≤ 0.016
	E. coli BW25113 Δ*recA*	≤ 0.016	0.016	≤ 0.016	≤ 0.016
	E. coli BW25113 Δ*tsx*	0.063	0.25	0.25	0.125
	E. coli BW25113 Δ*parC*	0.063	0.063	0.031	≤ 0.016

[1] CIP = ciprofloxacin, [2] WT = wild type,
[a] *E. coli* clinical wild type isolate , [b] *E. coli* [*gyrA*(S83L, D87G)], [c] *E. coli* [*gyrA*(S83L, D87G), *parC*(S80I)], [d] *E.coli* [*gyrA*(S83L)], [e] *E. coli* [*gyrA*(S83L), *marR*(Δ175bp)], [f] *E. coli* [*gyrA*(S83L, D87G), *parC*(S80I), *marR* (Δ175bp)], additional strain information is given in supplementary information

≤ 0.016	0.031	0.063	0.125	0.25	0.5	1.0	2.0	4.0	8.0	16.0	32.0	≥ 64.0

Formation of Resistance

To determine the mutation frequency the individual strain was grown to an approximately concentration of 10^{12} cfu ml^{-1}. Next a dilution-sequence was built up on a MHB-II medium with a factor of 1:10 each, by using the 0.5-McFarland-Standard. From the culture dilutions were taken 100 µL each (10^{12} – 10^{6} cfu mL^{-1}) and plated on MHB-II-Agar plates, with the eight times MIC-concentration of the compound to be studied. The number of colonies appearing after 18 h at 37°C was counted and the frequency of resistance was calculated by dividing this number by the inoculum.

Table S 3. Frequency of resistance values for albicidin (1) and compound 2

	FoR (8x MIC)	
Strain	**albicdin (1)**	**2**
E.coli **DSM1116**	< 1.22×10^{-11}	< 1.22×10^{-11}
E.coli **ATCC 25992**	< 1.43×10^{-11}	< 1.43×10^{-11}
P.aeruginosa **ATCC 27853**	< 1.16×10^{-11}	< 1.16×10^{-10}
A.baumannii **PEG 10-57-24**	n.d	< 1.11×10^{-10}
S.aureus **ATCC29213**	n.d	< 1.11×10^{-11}

Cell proliferation assay (HepG2 cells)

The assay was performed by the company Eurofins (Taiwan). Cytotoxicity was representatively determined for compound **2** (concentration 16 µM – close to maximal buffer solubility) on HepG2 cells according to a protocol by Ansar et al.[27] This is in line with previously published cytotoxicity data on cystobactamid analogs.[4-6]

Table S 4. Cell proliferation of HepG2 cells for compound 2.

	**Compound 2**	**Reference Staurosporine**
**GI_{50}**	>16 µM	5.23 nM
**TGI**	>16 µM	<0.14 µM
**LC_{50}**	>16 µM	5.81 µM

GI = growth inhibition; TGI = total growth inhibition; LC = lethal concentration

Hemolysis Assay

Sterile, defibrinated sheep blood (Merck KGaA, Darmstadt/Germany) was washed four times with 0.9% NaCl solution at 300 x g, 10 min to remove blood plasma. Subsequently, an erythrocyte suspension of 1x10^8 cells/mL was freshly prepared in 0.9% NaCl. Final concentrations of 140 µM compound **7** (f.c. DMSO: 5%) were added to the erythrocytes followed by an incubation for 1 h at 37°C, 300 rpm. After centrifugation at 600 x g, 10 min, supernatant was photometrically analyzed at 410 nm (Tecan i-control, Infinite-200). Hypotonically lysed erythrocytes (1% Triton X-100) were used as a normalization standard of 100% hemolysis. The experiment was repeated twice. For compound **7** hemolysis was ≈3.7 % (140 µM).

Table S 5. Experimental data of hemolysis experiments on defibrinated sheep blood for compound 7 at 140 µM.

**Experiment 1**	**1**	**2**	**3**	**Average**	**Factor 3**	**corr NaCl**	**% hemolysis**
1% Triton X	2,9482	2,9442	2,9165	2,9363	8,8089	8,4177	100
0.9% NaCl	0,1488	0,1389	0,1035	0,1304	0,3912	0	0
5% DMSO	0,583	0,2895	0,5060	0,4595	1,3785	0,9873	11,7289
1% Triton X + 5% DMSO	2,7988	2,8877	2,8640	2,8502	8,5505	8,1593	96,9297
Compound **7**	0,4702	0,4006	0,4062	0,4257	1,2770	0,2897	**3,4412**
**Experiment 2**	**1**	**2**	**3**	**Average**	**Factor 15**	**corr NaCl**	**% hemolysis**
Triton X	0,4202	0,4102	0,4184	0,4163	6,2440	5,4080	100
5% DMSO	0,1959	0,1670	0,1495	0,1708	2,5620	1,7260	31,9157
0,9% NaCl	0,0552	0,0555	0,0565	0,0557	0,836	0	0
1% Triton 5% DMSO	0,4276	0,4010	0,4118	0,4135	6,2020	5,3660	99,2233
Compound **7**	0,1416	0,1257	0,1199	0,1291	1,9360	0,2100	**3,8831**
Average	-	-	-	-	-	-	**3,6622**

Determination of plasma stability

Human and murine plasma aliquots (Sigma-Aldrich, Taufkirchen, Germany) were thawed and subsequently centrifuged at 3000 rpm for 10 min at 4°C. Resulting supernatant was diluted in the same volume of phosphate buffer (pH 7.4) to obtain a plasma concentration of 50% (v/v). Stock solutions of 200 µM Albicidin **1**, compounds **2** or **7** were diluted in 50% human or murine plasma to a final concentration of 20 µM (5% DMSO). Following incubation at 37°C ice-cold methanol containing 30% (v/v) THF was added to the samples at timepoints 0, 60, 120, 180 and 240 min. Subsequently, samples were centrifuged at 4000 rpm for 20 min at 4°C and 100 µL of supernatant were directly transferred to a fresh MS-vial. Samples were analyzed by LC/MS-MS experiments. Quantification of remaining compounds was performed by an external calibration curve of standard solutions in the range of 100 µM to 0,5 µM.

Formulation studies

In preparation to *in vivo* experiments, solubility of compound **2** was evaluated in nine different types of preclinical formulations in a final concentration of 10 mg ml^{-1}. All experimental procedures were carried out in cooperation with Pharmacelsus GmbH (Saarbrucken, Germany) according to Table S6.

Table S 6. Evaluation of solubility of compound 2 in nine different preclinical formulations.

		1% HPMC in water/ Tween 80 (99:1)	10-20% SBECD	DMSO/ 10% 2-HPßCD in water (10:90)	1% HPMC in water/ PEG 400 (25/75)	DMSO/ PEG (20:80)	Labrafac™ lipophile WL 1349	Capryol 90/ 10% Tween 80 (25:75)	DMSO/ 31.6% 2-HPßCD (5:95)	DMSO/ homologous plasma (10:90)
Principle		surfactant	complexing agent		co-solvent		solution or suspension			
**Compound 2**	mg	4.095	4.033	4.005	4.051	4.016	4.461	4.474	3.006	4.011
**Reagents**										
HPMC (1%)	µL	405	-	-	101	-	-	-	-	-
Tween 80	µL	4	-	-	-	-	-	336 (10%)	-	-
Captisol (SBECD 10-20%)	µL	-	403	-	-	-	-	-	-	-
DMSO (100%)	µL	-	-	40	-	80	-	-	15	40
2-HPßCD 10%	µL	-	-	360	-	-	-	-	286 (31,6%)	-
homologoues plasma	µL	-	-	-	-	-	-	-	-	361
PEG 400	µL	-	-	-	304	321	-	-	-	-
Labrafac™ lipophile Wl 1349	µL	-	-	-	-	-	446	-	-	-
Capryol 90	µL	-	-	-	-	-	-	112	-	-
**Procedures**										
1st homogenization step						vortex vigirously				
observations following first homogenization step		opaque solution with particles	opaque solution with small particles	opaque solution with small particles	opaque solution with particles	opaque solution with particles	opaque solution with particles	opaque solution with particles	opaque solution with particles	opaque solution with small particles
2nd homogenization step						ultrasonic bath, 37°C				
observations final formulation		opaque solution with particles	insoluble large particles	insoluble large particles	insoluble large particles	clear solution without particles	insoluble large particles	insoluble large particles	opaque solution with particles	opaque solution with particles
Microscopial findings		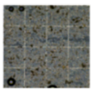	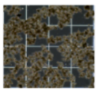	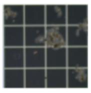	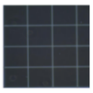	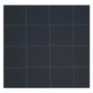	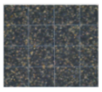	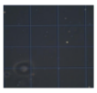	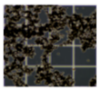	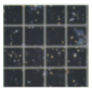

1.1.2 Tolerability assessment and pharmacokinetic study

Cooperation partner

In vivo tolerability studies as well as determinations of pharmacokinetic parameters were carried out in cooperation with Pharmacelsus GmbH (Saarbrucken, Germany).

Animals and housing

For tolerability assessment adult male CD-1 mice (RjOrl:SWISS, 7 weeks old) were purchased from Janvier Labs (France). For pharmacokinetic studies 6 weeks old, male CD-1 mice (RjOrl:SWISS, Janvier Labs, France) were used. The animals were allowed to acclimatize for at least seven days before the start of experiments. Housing was realized in a temperature-controlled room (20-24°C) and mice were exposed to a 12h light/dark cycle. Mice had access to food and water *ad libitum* throughout the study. All experimental procedures were approved by and conducted in accordance with the regulations of local Animal Welfare authorities (Landesamt für Gesundheit und Verbraucherschutz, Abteilung Lebensmittel- und Veterinärwesen, Saarbrücken, Germany).

Tolerability Assessment

Compound **2** was dissolved in DMSO/PEG (10:90) and was immediately administered at doses of 12.5 mg kg^{-1}, 25 mg kg^{-1} or 50 mg kg^{-1} intravenously (i.v.) to mice (n=3 for each dose) at t=0 h and t=12 h. Application volume of 5 ml kg^{-1} was used. Samples of K-EDTA blood were collected from each mouse before (predose) and 24 h after the first injection of compound **2**. An individual blood cell count was performed for each sample. Mice were weighed at t=0 h and t=12 h and were observed for clinical signs of intolerability post administration at t=0 h and t=12 h, respectively. Pain assessment was performed with help of the grimace scale to monitor any changes in facial expression. After 24 h mice were sacrificed, and a macroscopic necropsy was performed to search for damaged organs. Data of blood cell count is given in Table S7 and reference values for measured blood cell parameters are given in Table S8.

Table S 7. Blood cell count predose and 24 hours after first injection of compound 2.

		LEU [x10^9 L^{-1}]	LYM [x10^9 L^{-1}]	MON [x10^9 L^{-1}]	NEU [x10^9 L^{-1}]	LYM [%]	MON [%]	NEU [%]	ERY [x10^{12} L^{-1}]	HGB [g dL^{-1}]	HCT [%]	MCV [fl]	MCHC [g dL^{-1}]
12.5 mg kg^{-1} TU-AF-C-014	**predose**												
	#352	6.70	5.95	0.20	0.55	88.8	2.9	8.2	9.25	13.6	41.92	45.00	32.4
	#353	8.17	6.83	0.28	1.06	83.6	3.5	13.0	9.35	13.6	42.55	46.00	32.0
	#354	5.81	5.25	0.04	0.53	90.3	0.6	9.1	9.30	14.4	44.40	48.00	32.4
	Mean	6.89	6.01	0.17	0.71	87.57	2.33	10.10	9.30	13.87	42.96	46.33	32.27
	SD	1.19	0.79	0.12	0.30	3.52	1.53	2.55	0.05	0.46	1.29	1.53	0.23
	**24 h**												
	#352	11.14	8.75	0.07	2.32	78.6	0.6	20.8	8.79	13.0	40.98	47.00	31.6
	#353	10.73	4.32	0.21	6.21	40.2	1.9	57.9	8.76	13.00	40.22	46.00	32.4
	#354	12.70	8.36	0.50	3.84	65.8	3.9	30.2	8.34	12.8	39.70	48.00	32.3
	Mean	11.52	7.14	0.26	4.12	61.53	2.13	36.30	8.63	12.93	40.30	47.00	32.10
	SD	1.04	2.45	0.22	1.96	19.55	1.66	19.29	0.25	0.12	0.64	1.00	0.44
25 mg kg^{-1} TU-AF-C-014	**predose**												
	#355	9.34	8.57	0.68	0.09	91.8	7.2	1.0	8.95	13.0	41.29	46.00	31.6
	#356	2.70	2.35	0.08	0.27	87.2	2.9	9.9	14.96	9.2	71.30	48.00	12.9
	#357	5.10	4.54	0.17	0.39	89.0	3.3	7.6	11.80	12.5	55.32	47.00	22.6
	Mean	5.71	5.15	0.31	0.25	89.33	4.47	6.17	11.90	11.57	55.97	47.00	22.37
	SD	3.36	3.16	0.32	0.15	2.32	2.38	4.62	3.01	2.06	15.02	1.00	9.35
	**24 h**												
	#355	4.61	2.59	0.17	1.86	56.1	3.6	40.3	7.63	11.4	35.43	46.00	32.1
	#356	13.33	10.17	0.13	3.03	76.3	1.0	22.7	8.58	13.1	39.73	46.00	33.0
	#357	8.19	5.28	0.13	2.78	64.5	1.5	34.0	8.45	12.4	38.90	46.00	31.9
	Mean	8.71	6.01	0.14	2.56	65.63	2.03	32.33	8.22	12.30	38.02	46.00	32.33
	SD	4.38	3.84	0.02	0.62	10.15	1.38	8.92	0.52	0.85	2.28	0.00	0.59
50 mg kg^{-1} TU-AF-C-014	**predose**												
	#358	5.16	4.40	0.41	0.35	85.3	7.9	6.8	11.02	15.0	55.69	51.00	26.9
	#359	6.40	5.26	0.10	1.04	82.2	1.6	16.3	11.53	15.2	53.90	47.00	28.1
	#360	5.87	4.87	0.28	0.73	82.9	4.7	12.4	10.34	14.3	52.31	51.00	27.3
	Mean	5.81	4.84	0.26	0.71	83.47	4.73	11.83	10.96	14.83	53.97	49.67	27.43
	SD	0.62	0.43	0.16	0.35	1.63	3.15	4.78	0.60	0.47	1.69	2.31	0.61
	**24 h**												
	#358	8.31	5.37	0.06	2.89	64.6	0.7	34.7	8.60	13.7	43.74	51.00	31.3
	#359	12.42	8.43	0.43	3.56	67.9	3.4	28.7	9.22	12.9	43.64	47.00	29.5
	#360	9.61	6.82	0.36	2.44	70.9	3.7	25.4	8.07	12.3	40.96	51.00	29.9
	Mean	10.11	5.05	0.26	1.51	67.4	3.7	17.6	8.79	12.3	43.24	43.75	25.1
	SD	2.10	1.53	0.20	0.56	3.15	1.65	4.71	0.58	0.70	1.58	2.31	0.95
10%/90% DMSO/PEG Vehicle	**predose**												
	#361	3.87	2.98	0.12	0.77	77.1	3.1	19.8	9.75	14.1	46.46	48.00	30.4
	#362	5.06	4.06	0.20	0.80	80.3	3.9	15.7	9.06	13.9	42.89	47.00	32.4
	#363	8.05	5.55	0.64	1.87	68.9	7.9	32.2	9.29	13.7	44.96	48.00	30.4
	Mean	5.66	4.20	0.32	1.15	75.43	4.97	22.57	9.37	13.90	44.77	47.67	31.07
	SD	2.15	1.29	0.28	0.63	5.88	2.57	8.59	0.35	0.20	1.79	0.58	1.15
	**24 h**												
	#361	9.67	5.27	0.53	3.86	54.5	5.5	39.9	7.91	12.1	38.07	48.00	31.8
	#362	9.16	6.94	0.06	2.16	75.8	0.7	23.6	8.97	13.5	42.81	48.00	31.4
	#363	5.42	3.79	0.14	1.49	69.9	2.6	27.4	9.16	13.3	44.57	49.00	29.8
	Mean	8.08	5.33	0.24	2.50	66.73	2.93	30.30	8.68	12.97	41.82	48.33	31.00
	SD	2.32	1.58	0.25	1.22	11.00	2.42	8.53	0.67	0.76	3.36	0.58	1.06

Table S 8. Reference values for determined blood cell count parameters.

**Parameter**	**abbrev./unit**	**normal range**
Leucocytes	LEU [x10^9 L^{-1}]	6.00-15.00
Lymphocytes	LYM [x10^9 L^{-1}]	3.40-7.44
Monocytes	MON [x10^9 L^{-1}]	0.00-0.60
Neutrophiles	NEU [x10^9 L^{-1}]	0.50-3.80
% Lymphocytes	LYM [%]	57-93
% Monocytes	MON [%]	0-7
% Neutrophiles	NEU [%]	8-48
Erythrocytes	ERY [x10^{12} L^{-1}]	7.00-12.00
Haemoglobin	HGB [g dL^{-1}]	12.2-16.2
Haematocrit	HCT [%]	35.00-45.00
Mean corpuscular volume	MCV [fl]	45-55
Mean corpuscular haemoglobin concentration	MCHC [g dL^{-1}]	22.3-32.0

Pharmacokinetic evaluation

For single dosing PK studies solutions of both compounds **2** or **7** in DMSO/PEG (10:90) were prepared.10.40 mg compound **2** were dissolved in 900 µL DMSO/PEG (10:90) or 6.4 mg of compound **7** were dissolved in 640 µL of DMSO/PEG (10:90), respectively. Compound solutions were mixed by vortexing and subsequently administered at doses of 50 mg kg^{-1} to mice (n=3 for each compound) at t=0 h. Animals were monitored for clinical signs after administration. 20 µL blood was collected from the tail vein into K-EDTA tubes at time points 0.25, 0.5, 1, 2, 4, 8, 24 h post administration. Blood samples were immediately frozen on dry ice and afterwards stored at -80°C until Liquid chromatography – mass spectrometry (LC-MS) analysis.
PK samples were finally analyzed by HPLC-MS. HPLC system consists of Surveyor MS Plus pump and an AS Plus autosampler. For mass spectrometry an ESI-TSQ Quantum Discovery Max Triple Quadrupole MS was used. MS data was analyzed via Xcalibur 2.0.7.

Preparation of blood samples, calibration standards and quality controls

Quality control samples (QCs) and calibration standards were prepared in duplicates by spiking 20 µL of drug free plasma from CD-1 mice with 2.4 µL of various compound concentrations (compound **2** or **7** in 100% DMSO starting from a concentration of 200 µg mL^{-1}). Accordingly, blood samples, zero samples and blanks were spiked with 2.4 µL DMSO (100%). The concentration of calibration standards was chosen in the range of expected blood sample concentrations.
Following sample equilibration (1 min, room temperature) 40 µL acetonitrile (100%) containing a concentration of 600 ng mL^{-1} Griseofulvin (internal standard) was added to blood samples, zero samples, calibration standards and QCs. Acetonitrile (100%) was added to blank samples. All samples were vigorously vortexed and were afterwards spun down (6000 g, 10 min, room temperature). Subsequently, a 1 to 1 dilution of the particle free supernatant in water was prepared and an aliquot was transferred to 200 µL HPLC sampler vials. 12 µL of each sample was injected to LC-MS system.

LC-MS measurements

For separation of compound **2** or **7** and internal standard an analytical column Kinetex Phenyl-Hexyl, 2.6 µm, 50 × 2.1 mm with a precolumn (Gemini C6-Phenyl 4.0 × 2.0 mm, Phenomenex, Germany) was

used. Compound separation was performed in gradient mode using acetonitrile + 0.1% formic acid as organic phase (A) and water + 0.1% formic acid as aqueous phase (B). HPLC gradient was chosen as follows: 95% B from 0.0 min to 0.1 min, 3% B from 0.6 to 1.7 min and 95% B from 1.8 min to 2.5 min. The HPLC flow rate was set to 600 µL min^{-1}.
Protonated molecular ions $[M+H]^+$ were measured in positive ion mode using syringe pump infusion. Auto-tuning to maximize ion abundance was performed, followed by identification of characteristic fragment ions using a generic parameter set: ion transfer capillary temperature 350°C, capillary voltage 3.8 kV, collision gas 0.8 mbar argon, sheath gas, ion sweep gas and auxiliary gas pressure were 20, 2 and 8 (arbitrary units), respectively. Ions with highest signal to noise ratio were used as monitoring ions for compound quantification in selected reaction monitoring mode (Table S9). For final quantification of compound **2** or **7** in blood samples a calibration curve was used (accurate best-fit calibration). The lower limit of quantification was set to 21.6 ng mL^{-1} blood. With-in run accuracy of calibration curve was determined by independently prepared QC samples.

Table S 9. Mass spectrometry conditions and instrument parameters for separation of compound 2 and 7.

compound	Molecular weight	$[M+H]^+$ [m/z]	Monitoring ion [m/z]	Qualifier ion [m/z]	Scan time [s]	Collision energy [V]	RT [min]
**2**	884.86	885.0	280.0	-	0.01	20	1.15
**7**	885.9	886.4	703.0	161.0	0.01/0.01	20/50	1.15
Griseofulvin (ISTD)	352.8	353.0	215.0	-	0.01	20	1.15

Mean concentrations of compound **2** and **7** in blood for all measured time points are given in Table S10.

Table S 10. Blood concentrations of compound 2 and 7 after i.v. dosing at 50 mg kg^{-1} after selected time points.

	**2** [µg mL^{-1}]			**7** [µg mL^{-1}]		
**Time [h]**	Mean	SD	CV [%]	Mean	SD	CV [%]
**0.25**	101.88	39.99	0.04	63.16	37.37	0.59
**0.5**	62.64	47.80	0.08	26.46	7.19	0.27
**1**	40.05	18.78	0.05	16.56	5.23	0.32
**2**	14.61	3.63	0.03	13.07	4.37	0.33
**4**	5.03	2.65	0.05	10.98	1.33	0.12
**8**	2.13	1.15	0.05	9.15	1.07	0.12
**24**	0.76	0.61	0.08	1.75	1.29	0.74

Pharmacokinetic calculations

Pharmacokinetic analysis of blood samples was based on a 2-compartment model using Kinetica 5.0 software (Thermo Scientific, Waltham, USA) and 1 x Y_{obs}^{-2} weighting. Results of pharmacokinetic determinations are summarized in Table S11.

Table S 11. Pharmacokinetic parameters based on 2-compartment analysis for compound 2 and 7 administered i.v. at 50 mg kg^{-1}

	**2**		**7**	
**PK parameter**	**Mean**	**SD**	**Mean**	**SD**
**AUC** [ng mLh^{-1}]	153320.0	47239.0	224852.0	49029.0
**λ_z** [h^{-1}]	0.1	0.0	0.1	0.06
**$C_{max\ calc}$** [ng mLh^{-1}]	111906.0	88454.0	267370.0	224221.0
**$T_{max\ calc}$** [h]	0.0	0.0	0.0	0.0
**k_{el}** [h^{-1}]	0.7	0.4	1.2	1.0
**k_{12}** [h^{-1}]	0.2	0.1	3.9	1.8
**k_{21}** [h^{-1}]	0.1	0.04	0.7	0.4
**$T_{1/2}\lambda_z$** [h]	10.1	2.5	8.3	3.8
**$T_{1/2}$** k_{el} [h]	1.2	0.6	1.4	1.5
**V_Z** [ml kg^{-1}]	4852.4	518.5	2664.2	1080.2
**CL** [mL /h /kg]	352.4	128.6	229.2	47.5

For comparison purposes the structure of **CYS-22** is shown in Figure S2.[4]

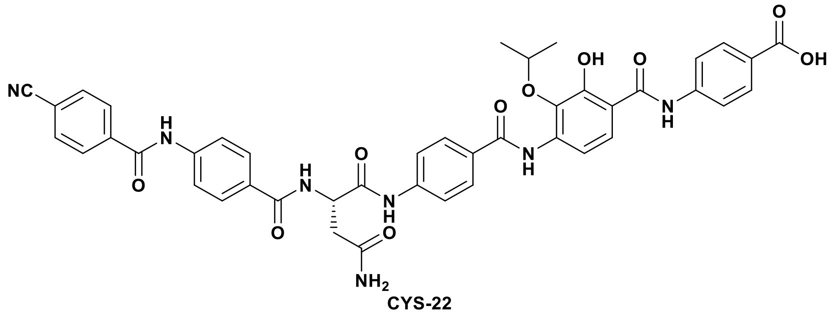

CYS-22

Figure S 2. Structure of Cystobactamid derivative CYS-22, which was evaluated in vivo.[4]

1.1.3 Murine septicemia infection model

Collaboration partner

In vivo E. coli induced septicemia infection model was carried out in cooperation with the Atlangram (Nantes, France).

Animals and housing

For *in vivo* infections study female CD-1 mice (RjOrl:SWISS, 6 weeks old) were purchased from Janvier Labs (Le Genest Saint Isle, France). The animals were allowed to acclimatize for at least seven days before the start of experiments. Group-Housing was realized in a pathogen-free, temperature-controlled room (20-24°C) and mice were exposed to a 12h light/dark cycle. Mice had access to food and water *ad libitum* throughout the study. All experimental procedures were approved by and conducted in accordance to the institutional guidelines.

Bacterial strain

Clinical fluoroquinolone resistant isolate of *E. coli* isolate number 191950584 was used for murine septicemia infection model. Following overnight incubation in Brain Heart infusion at 37°C, the strain was spin down at 2.500 rpm for 10 min. Immediately before use, bacteria pellet was resuspended in sterile saline and the inoculum was calibrated to 10^9 CFU mL^{-1} by nephelometry.

In vivo infection study

For infection procedure, mice were briefly anesthetized with continuous inhalation of isoflurane. 100 µl of bacterial suspension were intravenously administered. At least nine animals were used for each of the following therapeutic groups:

- Untreated but infected animals: control group (PBS)
- Drug candidate, dose 1 group (6 mg kg^{-1});
- Drug candidate, dose 2 group (17 mg kg^{-1}).

Compound **7** or PBS were administered by intravenous infection route 2 h and 12 h post infection, respectively. Animals were monitored for clinical signs throughout the whole study using a 4-point body condition score analyzing weight loss, respiratory distress, hampered mobility and hunched posture. Animals were euthanized after 24 hours of infection. Spleen and kidneys from each animal were removed, weighed, and homogenized in 2 mL of PBS and used for quantitative cultures on agar for 24 h at 37°C. Viable counts, after 48 h of incubation, were expressed as the mean$\pm$SD $\log_{10}$ CFU per gram of organ.

1.2 CHEMISTRY

1.2.1 Materials and methods

Commercially available reagents (Carl Roth GmbH and Co. KG, Karlsruhe, Germany; Sigma-Aldrich, Taufkirchen, Germany; Iris Biotech GmbH, Marktredwitz, Germany; Orpegen, Heidelberg, Germany; ABCR, Karlsruhe, Germany; Alfa Aesar, Karlsruhe, Germany; Merk, Darmstadt, Germany; TCI, Eschborn, Germany; VWR International GmBH, Darmstadt, Germany; Acros, Geel, Belgium) and solvents (Fisher Scientific-Acros, Schwerte, Germany) were used without further purification. If necessary, reactions were carried out under an atmosphere of argon or nitrogen and in dry solvents. Analytical thin layer chromatography was carried out using aluminium backed plates coated with Macherey-Nagel silica gel (60, F254). Analysis was performed by visualizing under UV light (λ = 254 nm), by staining with $KMnO_4$-solution ($KMnO_4$ (3 g), K_2CO_3 (20 g, H2O (300 mL), 5% NaOH(aq) (5 mL)) and with ninhydrin-solution (ninhydrin (0.3 g), AcOH (3 mL), nBuOH (100 mL)). Column chromatography was carried out with silica gel (particle size 40-63 µm, VWR Chemicals, Darmstadt, Germany). Preparative HPLC was carried out on a 1260 Infinity (Agilent Technologies, Waldbronn, Germany) system with a polymeric reversed phase column (PLRP-S 100A) 300 x 50 mm, particle size 10 µm, Agilent Technologies, Waldbronn, Germany). ^{1}H and ^{13}C NMR spectra were recorded at 298 K using Bruker Avance-II 400 MHz, Bruker Avance-III 500 MHz or Bruker Avance-III 700 MHz instruments (Bruker, Karlsruhe, Germany). The chemical shifts are reported in ppm using the residual solvent peak as an internal reference (DMSO-d_6, $CDCl_3$ or THF-d_8). Multiplicity (br. s = broad singlet, s = singlet, d = doublet, dd = doublet of doublet, t = triplet, q = quartet, m = multiplet) and coupling constants (J = Hz) are quoted where possible. HPLC-HRMS spectra were recorded on a QTrap LTQ XL (Thermo Fisher Scientific, Waltham, Massachusetts, USA) with an Agilent 1200 Series HPLC-System (Agilent) Technologies, Waldbronn, Germany) with a C18 column (50 x 2 mm, particle size 3 µm).

1.2.2 Experimental procedures

Compounds which have been synthesized before have a citation in the name of the molecule, indicating that the procedure used for the reaction was adapted from the cited document.

1.2.3 Synthesis of A-Variations (3, 4)

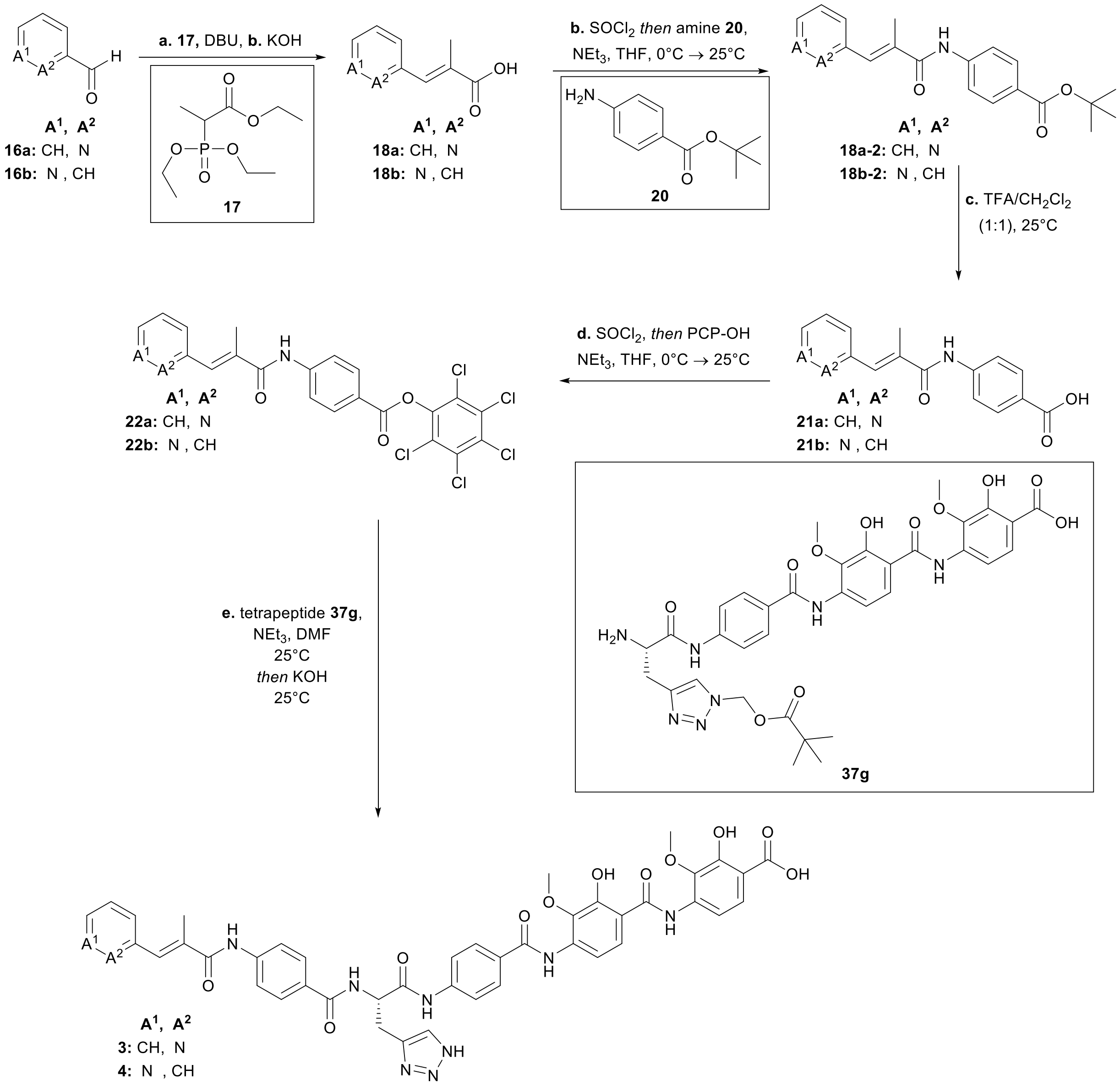

Scheme S 1. *General synthesis of A variations **3** and **4**.*

(*E*)-2-methyl-3-(pyridin-2-yl)acrylic acid (18a)[7]

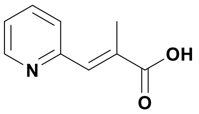

Chemical Formula: $C_9H_9NO_2$
Exact Mass: 163,0633

Lithium chloride (4.06 g, 95.6 mmol) was stirred for 15 min in acetonitrile (250 mL). To the cloudy solution was added picolinaldehyde (5.0 g, 46.7 mmol), ethyl 2-(diethoxyphosphoryl) propanoate (12.6 mL, 58.4 mmol), and 1,8-diazabicyclo- [5.4.0]undec-7-ene (6.97 mL, 46.7 mmol). The reaction mixture was stirred at room temperature for 12 h. To the reaction was added saturated sodium bicarbonate solution and extracted with EtOAc three times. The combined organic layers were washed with brine and dried on sodium sulfate, filtered, and concentrated. The crude material was purified by column chromatography with a gradient of 27−50% cyclohexane/ ethyl acetate to give ethyl (E)-2-methyl-3-(pyridin-2-yl)acrylate (**18a-1**) as a colorless oil. HRMS (ESI) calculated for $C_{11}H_{13}NO_2$ (M+H$^+$) 192.1019, found 192.1018. The compound **18a-1** was directly used in the next step. The residue was dissolved in THF (50 mL) and KOH 3 N (50 mL) was added. The reaction stirred at 25°C for 30 min. Next, the solvent was partially evaporated under reduced pressure, the aqueous residue was acidified with HCl 1 N (155 mL) to pH ~ 5 and cooled to 0°C. The precipitate was filtered and dried in vacuo to obtain 5.42 g of a colorless solid (33.3 mmol, 71%, over 2 steps).

^{1}H NMR (500 MHz, DMSO-d_6) δ 13.02 (s, 1H), 8.87 (d, *J* = 5.6 Hz, 1H), 8.53 (t, *J* = 7.9 Hz, 1H), 8.12 (d, *J* = 8.1 Hz, 1H), 7.94 (t, *J* = 6.7 Hz, 1H), 7.75 (t, *J* = 1.7 Hz, 1H), 2.14 (s, 3H).
^{13}C NMR (126 MHz, DMSO) δ 168.3, 148.8, 145.3, 143.4, 138.8, 128.8, 127.9, 126.3, 15.1.
HRMS (ESI) calculated for $C_9H_{10}NO_2$ (M+H$^+$) 164.0706, found 164.0704.

Tert-butyl (*E*)-4-(2-methyl-3-(pyridin-2-yl)acrylamido)benzoate (18a-2)

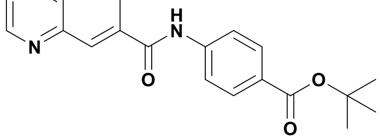

Chemical Formula: $C_{20}H_{22}N_2O_3$
Exact Mass: 338,1630

(*E*)-2-methyl-3-(pyridin-2-yl)acrylic acid (**18a**, 2.00 g, 12.3 mmol) was refluxed in $SOCl_2$ (8.89 mL, 123 mmol) at 80°C for 1.5 h. All volatiles were removed under reduced pressure, the residue was dissolved in THF (10 mL), the solution was dropwise added to a mixture of NEt_3 (3.42 mL, 24.5 mmol) and tert-butyl 4-aminobenzoate (**20**, 2.25 g, 11.6 mmol) in THF (20 mL) at 0°C. The solvent was partially evaporated under reduced pressure, the residue was dissolved in dichloromethane (100 mL). Organic phase washed with a saturated solution of $NaHCO_3$ (100 mL), HCl 1N (100 mL), brine (100 mL) dried over sodium sulphate and evaporated under vacuum. The residue thus obtained was triturated with CH_2Cl_2 and chromatographed on silica gel with a gradient 0.4-3.0% acetone in CH_2Cl_2 with 0.1% NEt_3, in 0.6% steps to give 2.37 g of a colorless solid (7.00 mmol, 60%).

^{1}H NMR (500 MHz, DMSO-d_6) δ 10.31 (s, 1H), 8.69 (dd, *J* = 5.0, 1.7 Hz, 1H), 7.91 – 7.83 (m, 5H), 7.56 (d, *J* = 7.8 Hz, 1H), 7.34 (dd, *J* = 7.6, 4.8 Hz, 1H), 7.26 (s, 1H), 2.39 (d, *J* = 1.5 Hz, 3H), 1.55 (s, 9H).
^{13}C NMR (126 MHz, DMSO) δ 169.3, 165.1, 155.4, 149.8, 143.8, 137.2, 136.9, 132.3, 130.4, 126.4, 126.4, 123.1, 119.8, 80.8, 28.3, 15.2.
HRMS (ESI) calculated for $C_{20}H_{23}N_2O_3$ (M+H$^+$) 361.1521, found 361.1523.

(*E*)-4-(2-methyl-3-(pyridin-2-yl)acrylamido)benzoic acid (21a)

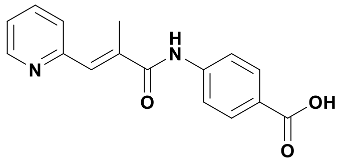

Chemical Formula: $C_{16}H_{14}N_2O_3$
Exact Mass: 282,1004

To a solution of tert-butyl (E)-4-(2-methyl-3-(pyridin-2-yl)acrylamido)benzoate (**18a-2**, 1.78 g, 5.25 mmol) in CH_2Cl_2 (20 mL) was added TFA (20 mL). The reaction mixture was stirred for 1 h at 25°C. All volatiles were removed under reduced pressure, to the residue was added HCl 1 N (20 mL) and lyophilized to give 1.41 g of a colorless solid (4.99 mmol, 95%).

^{1}H NMR (400 MHz, DMSO-d_6) δ 10.64 (s, 1H), 10.15 (s, 1H), 8.88 (dd, *J* = 5.7, 1.6 Hz, 1H), 8.44 (td, *J* = 7.9, 1.6 Hz, 1H), 8.05 (d, *J* = 8.0 Hz, 1H), 7.97 – 7.87 (m, 4H), 7.85 (ddd, *J* = 7.8, 5.5, 1.2 Hz, 1H), 7.49 (q, *J* = 1.5 Hz, 1H), 2.28 (d, *J* = 1.5 Hz, 3H).
^{13}C NMR (101 MHz, DMSO) δ 167.3, 166.9, 158.6, 149.4, 143.8, 142.9, 141.5, 130.2, 127.1, 125.8, 125.7, 125.1, 119.5, 15.1.
HRMS (ESI) calculated for $C_{16}H_{15}N_2O_3$ (M+H$^+$) 283.1077, found 283.1075.

Perchlorophenyl (*E*)-4-(2-methyl-3-(pyridin-2-yl)acrylamido)benzoate (22a)

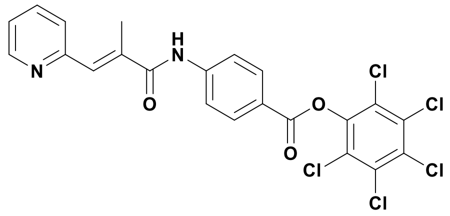

Chemical Formula: $C_{22}H_{13}Cl_5N_2O_3$
Exact Mass: 527,9369

(*E*)-4-(2-methyl-3-(pyridin-2-yl)acrylamido)benzoic acid (**21a**, 500 mg, 1.27 mmol) was refluxed at 80°C in $SOCl_2$ (0.909 mL, 12.7 mmol) for 1.5 h. All volatiles were removed under reduced pressure, the residue was dissolved in THF (10 mL), the solution was dropwise added to a mixture of NEt_3 (0.442 mL, 3.17 mmol) and pentachlorophenol (371 mg, 1.39 mmol) in THF (20 mL) at 0°C. The reaction mixture was stirred 2 d at 25°C. The solvent was evaporated under reduced pressure, the residue was triturated with dichloromethane and chromatographed on silica gel with a gradient 17-40% ethyl acetate in cyclohexane with 0.1% NEt_3, in 4.6% steps to give 344 mg of a light brown solid (0.648 mmol, 51%).

^{1}H NMR (500 MHz, DMSO-d_6) δ 10.53 (s, 1H), 8.70 (d, *J* = 4.1 Hz, 1H), 8.20 (d, *J* = 8.9 Hz, 2H), 8.04 (d, *J* = 8.9 Hz, 2H), 7.88 (td, *J* = 7.7, 1.9 Hz, 1H), 7.58 (d, *J* = 7.9 Hz, 1H), 7.35 (ddd, *J* = 7.6, 4.8, 1.1 Hz, 1H), 7.31 (q, *J* = 1.4 Hz, 1H), 2.41 (d, *J* = 1.4 Hz, 3H).
HRMS (ESI) calculated for $C_{22}H_{14}Cl_5N_2O_3$ (M+H$^+$) 528.9442, found 528.9446.

(S)-4-(4-(4-(2-amino-3-(1-((pivaloyloxy)methyl)-1H-1,2,3-triazol-4-yl)propanamido)benzamido)-2-hydroxy-3-methoxybenzamido)-2-hydroxy-3-methoxybenzoic acid (37g)[8]

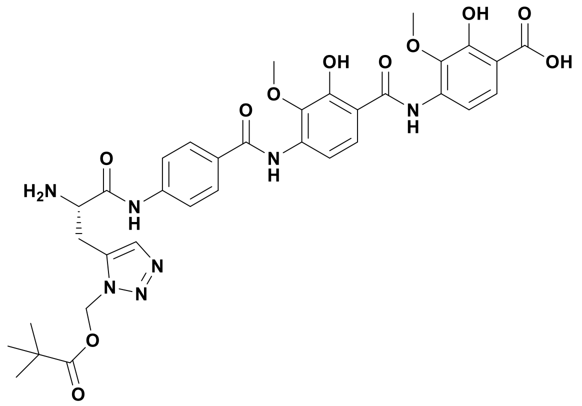

Chemical Formula: $C_{34}H_{37}N_7O_{11}$
Exact Mass: 719,2551

Synthesized according to exp. procedure reported.

^{1}H NMR (400 MHz, DMSO-d_6) δ 11.56 (s, 1H), 11.31 (s, 1H), 11.20 (s, 1H), 9.73 (s, 1H), 8.63 (d, *J* = 5.0 Hz, 2H), 8.17 (s, 1H), 8.06 (d, *J* = 8.9 Hz, 1H), 7.99 (d, *J* = 8.8 Hz, 2H), 7.82 (d, *J* = 8.8 Hz, 1H), 7.77 (d, *J* = 8.8 Hz, 2H), 7.58 (dd, *J* = 12.2, 8.8 Hz, 2H), 6.28 (s, 2H), 4.43 (d, *J* = 5.9 Hz, 1H), 3.92 (s, 3H), 3.78 (s, 3H), 3.42 – 3.32 (m, 2H), 1.07 (s, 9H).
^{13}C NMR (101 MHz, DMSO) δ 176.4, 172.0, 166.8, 164.8, 163.3, 154.4, 149.7, 141.4, 140.9, 140.3, 137.9, 136.1, 135.9, 129.3, 128.8, 125.7, 125.5, 125.0, 119.0, 116.3, 115.0, 110.3, 109.0, 69.9, 66.4, 60.5, 60.3, 52.7, 38.2, 26.5.
HRMS (ESI) calculated for $C_{34}H_{38}N_7O_{11}$ (M+H$^+$) 720.2624, found 720.2626.

(S,*E*)-2-hydroxy-4-(2-hydroxy-3-methoxy-4-(4-(2-(4-(2-methyl-3-(pyridin-2-yl)acrylamido)benzamido)-3-(1-((pivaloyloxy)methyl)-1H-1,2,3-triazol-4-yl)propanamido)benzamido)benzamido)-3-methoxybenzoic acid (3)

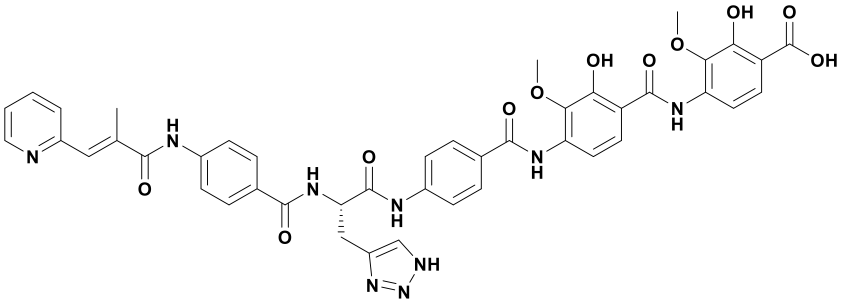

Chemical Formula: $C_{44}H_{39}N_9O_{11}$
Exact Mass: 869,2769

To a solution of tetrapeptide **37g** (60 mg, 79.6 µmol) in DMF (2 mL) was added perchlorophenyl (*E*)-4-(2-methyl-3-(pyridin-2-yl)acrylamido)benzoate (**22a**, 50.7 mg, 95.5 µmol) and NEt_3 (0.111 mL, 0.795 mmol). The reaction mixture was stirred for 2 d at 25°C and monitored by LC-MS (POM protected product: HRMS (ESI) calculated for $C_{50}H_{49}N_9O_{13}$ (M+H$^+$) 984.3523, found 984.3536). After full conversion KOH 3 N (1 mL) was added and stirred for 20 min. Next, the mixture was neutralized with HCl 6 N (0.5 mL) to a pH ~ 7. All volatiles were removed under reduced pressure, the residue was dissolved with DMSO (1 mL) and the salts were separated by centrifugation. The supernatant was loaded on the RP-HPLC system and purified under the following conditions. The collected fractions were lyophilized after their identity and purity was verified by LCMS to obtain 12 mg as a white solid (12.2 µmol, 15% over 2 steps).
RP-HPLC: 38-48% MeCN (+0.1% TFA) in milliQ H_2O (+0.1% TFA); 30 min; 70 mL/min.

^{1}H NMR (700 MHz, DMSO-d_6) δ 11.58 (s, 1H), 11.53 (s, 1H), 11.18 (s, 1H), 10.52 (s, 1H), 10.26 (s, 1H), 9.67 (s, 1H), 8.72 (d, *J* = 7.7 Hz, 1H), 8.70 (ddd, *J* = 4.9, 1.8, 0.9 Hz, 1H), 8.06 (d, *J* = 8.8 Hz, 1H), 7.97 (d, *J* = 8.8 Hz, 2H), 7.91 (td, *J* = 7.7, 1.9 Hz, 2H), 7.89 (d, *J* = 8.8 Hz, 2H), 7.85 – 7.77 (m, 5H), 7.69 (s, 1H), 7.59 (t, *J* = 8.1 Hz, 3H), 7.40 – 7.36 (m, 1H), 7.27 (q, *J* = 1.5 Hz, 1H), 4.92 (q, *J* = 7.5 Hz, 1H), 3.92 (s, 3H), 3.78 (s, 3H), 3.30 (dd, *J* = 14.8, 5.7 Hz, 1H), 3.24 (dd, *J* = 14.8, 9.3 Hz, 1H), 2.38 (d, *J* = 1.5 Hz, 3H).
^{13}C NMR (176 MHz, DMSO - from HSQC_ed) δ 149.3, 110.7, 129.3, 137.8, 128.7, 119.6, 119.6, 126.0, 119.2, 126.3, 115.2, 123.2, 131.8, 54.6, 60.7, 61.0, 27.8, 27.8, 54.6, 40.3, 15.2.
HRMS (ESI) calculated for $C_{44}H_{40}N_9O_{11}$ (M+H$^+$) 870.2842, found 870.2847, t_R = 6.73 min.

(*E*)-2-methyl-3-(pyridin-3-yl)acrylic acid (18b)[7]

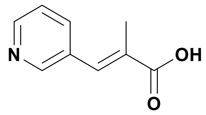

Chemical Formula: $C_9H_9NO_2$
Exact Mass: 163,0633

Lithium chloride (4.06 g, 95.6 mmol) was stirred for 15 min in acetonitrile (250 mL). To the cloudy solution was added nicotinaldehyde (**16b**, 5.0 g, 46.7 mmol), ethyl 2-(diethoxyphosphoryl) propanoate (**17**, 12.6 mL, 58.4 mmol), and 1,8-diazabicyclo- [5.4.0]undec-7-ene (6.97 mL, 46.7 mmol). The reaction mixture was stirred at room temperature for 12 h. To the reaction was added saturated sodium bicarbonate solution and extracted with EtOAc three times. The combined organic layers were washed with brine and dried on sodium sulfate, filtered, and concentrated. The crude material was purified by column chromatography with a gradient of 27-50% cyclohexane/ ethyl acetate to give ethyl (E)-2-methyl-3-(pyridin-3-yl)acrylate (**18a-2**) as a colorless oil. HRMS (ESI) calculated for $C_{11}H_{13}NO_2$ (M+H$^+$) 192.1019, found 192.1013. The compound **18a-2** was directly used in the next step. The residue was dissolved in THF (50 mL) and KOH 3 N (50 mL) was added. The reaction stirred at 25°C for 30 min. Next, the solvent was partially evaporated under reduced pressure, the aqueous residue was acidified with HCl 1 N (155 mL) to pH ~ 5 and cooled to 0°C. The precipitate was filtered and dried in vacuo to obtain 6.08 g of a colorless solid (37.3 mmol, 80%, over 2 steps).

^{1}H NMR (400 MHz, DMSO-d_6) δ 12.73 (s, 1H), 8.66 (d, *J* = 2.2 Hz, 1H), 8.54 (dd, *J* = 4.8, 1.7 Hz, 1H), 7.90 (dt, *J* = 8.0, 2.1 Hz, 1H), 7.59 (d, *J* = 1.7 Hz, 1H), 7.46 (ddd, *J* = 7.8, 4.7, 0.8 Hz, 1H), 2.03 (d, *J* = 1.5 Hz, 3H).
^{13}C NMR (101 MHz, DMSO) δ 168.9, 150.3, 149.0, 136.5, 134.1, 131.4, 130.9, 123.5, 13.9.
HRMS (ESI) calculated for $C_9H_{10}NO_2$ (M+H$^+$) 164.0706, found 164.0700.

Tert-butyl (*E*)-4-(2-methyl-3-(pyridin-3-yl)acrylamido)benzoate (18b-2)

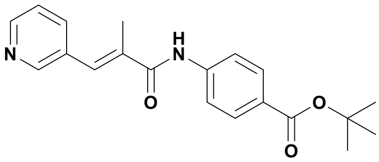

Chemical Formula: $C_{20}H_{22}N_2O_3$
Exact Mass: 338,1630

(*E*)-2-methyl-3-(pyridin-3-yl)acrylic acid (**18b**, 3.55 g, 21.7 mmol) was refluxed in $SOCl_2$ (14.8 mL, 207 mmol) at 80°C for 1.5 h. All volatiles were removed under reduced pressure, the residue was dissolved in THF (10 mL), the solution was added dropwise to a mixture of NEt_3 (5.77 mL, 41.5 mmol) and tert-butyl 4-aminobenzoate (**20**, 4.00 g, 20.7 mmol) in THF (20 mL) at 0°C. The solvent was partially evaporated under reduced pressure, the residue was dissolved in dichloromethane (100 mL). Organic phase washed with $NaHCO_3$ saturated solution (100 mL), HCl 1N (100 mL), brine (100 mL) dried over sodium sulphate and evaporated under vacuum. The residue thus obtained was triturated with CH_2Cl_2 and chromatographed on silica gel with a gradient 0.4-4.0% acetone in CH_2Cl_2 with 0.1% NEt_3, in 0.6% steps to give 6.29 g of a colorless solid (7.00 mmol; 86%).

^{1}H NMR (400 MHz, DMSO-d_6) δ 10.43 (s, 1H), 8.69 (d, *J* = 2.2 Hz, 1H), 8.54 (dd, *J* = 4.8, 1.6 Hz, 1H), 7.97 – 7.86 (m, 5H), 7.48 (ddd, *J* = 7.9, 4.9, 0.9 Hz, 1H), 7.39 (d, *J* = 2.1 Hz, 1H), 2.13 (d, *J* = 1.4 Hz, 3H), 1.54 (s, 9H).
^{13}C NMR (101 MHz, DMSO) δ 168.2, 164.6, 150.1, 148.7, 143.4, 136.3, 134.7, 131.6, 130.3, 129.8, 125.8, 123.5, 119.3, 80.3, 27.8, 14.5.

HRMS (ESI) calculated for $C_{20}H_{23}N_2O_3$ (M+H$^+$) 339.1703, found 339.1709.

(*E*)-4-(2-methyl-3-(pyridin-3-yl)acrylamido)benzoic acid (21b)

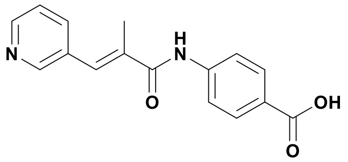

Chemical Formula: $C_{16}H_{14}N_2O_3$
Exact Mass: 282,1004

To a solution of tert-butyl (E)-4-(2-methyl-3-(pyridin-3-yl)acrylamido)benzoate (**18b-2**, 4.00 g, 11.8 mmol) in CH_2Cl_2 (20 mL) was added TFA (20 mL). The reaction mixture was stirred for 1 h at 25°C. All volatiles were removed under reduced pressure, to the residue was added HCl 1N (20 mL) and lyophilized to give 2.65 g of a colorless solid (9.39 mmol, 79%).

^{1}H NMR (500 MHz, DMSO-d_6) δ 10.63 (s, 1H), 8.98 (d, *J* = 2.0 Hz, 1H), 8.83 (dd, *J* = 5.6, 1.4 Hz, 1H), 8.54 (dt, *J* = 8.3, 1.8 Hz, 1H), 8.01 (dd, *J* = 8.1, 5.5 Hz, 1H), 7.93 (s, 4H), 7.50 (s, 1H), 2.15 (d, *J* = 1.4 Hz, 3H).
^{13}C NMR (126 MHz, DMSO) δ 168.3, 167.4, 143.9, 143.9, 143.7, 142.7, 137.9, 134.9, 130.6, 128.4, 126.8, 126.0, 119.9, 14.9.
HRMS (ESI) calculated for $C_{16}H_{15}N_2O_3$ (M+H$^+$) 283.1077, found 283.1071.

Perchlorophenyl (E)-4-(2-methyl-3-(pyridin-3-yl)acrylamido)benzoate (22b)

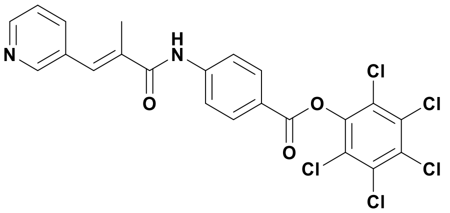

Chemical Formula: $C_{22}H_{13}Cl_5N_2O_3$
Exact Mass: 527,9369

(E)-4-(2-methyl-3-(pyridin-3-yl)acrylamido)benzoic acid (**21b**, 500 mg, 1.77 mmol) was refluxed at 80°C in $SOCl_2$ (0.909 mL, 12.7 mmol) for 1.5 h. All volatiles were removed under reduced pressure, the residue was dissolved in THF (10 mL), the solution was dropwise added to a mixture of NEt_3 (0.442 mL, 3.17 mmol) and pentachlorophenol (371 mg, 1.39 mmol) in THF (20 mL) at 0°C. The reaction mixture was stirred 2 d at 25°C. The solvent was evaporated under reduced pressure, the residue was triturated with dichloromethane and chromatographed on silica gel with a gradient 17-40% ethyl acetate in cyclohexane with 0.1% NEt_3, in 4.6% steps give 477 mg of a light brown solid (0.899 mmol; 51%).

^{1}H NMR (700 MHz, DMSO-d_6) δ 10.51 (s, 1H), 8.70 (d, *J* = 2.3 Hz, 1H), 8.56 (dd, *J* = 4.8, 1.6 Hz, 1H), 8.19 (d, *J* = 8.8 Hz, 2H), 8.03 (d, *J* = 8.8 Hz, 2H), 7.92 (dt, *J* = 8.1, 2.1 Hz, 1H), 7.49 (ddd, *J* = 7.9, 4.8, 0.9 Hz, 1H), 7.38 (d, *J* = 2.0 Hz, 1H), 2.15 (d, *J* = 1.4 Hz, 3H).
^{13}C NMR (176 MHz, DMSO) δ 169.0, 162.3, 150.7, 149.3, 146.0, 144.6, 136.8, 135.2, 132.0, 132.0, 131.8, 131.3, 131.2, 127.9, 124.0, 121.0, 120.3, 15.0.
HRMS (ESI) calculated for $C_{22}H_{14}Cl_5N_2O_3$ (M+H$^+$) 528.9442, found 528.9454.

(S,*E*)-2-hydroxy-4-(2-hydroxy-3-methoxy-4-(4-(2-(4-(2-methyl-3-(pyridin-3-yl)acrylamido)benzamido)-3-(1H-1,2,3-triazol-4-yl)propanamido)benzamido)benzamido)-3-methoxybenzoic acid (4)

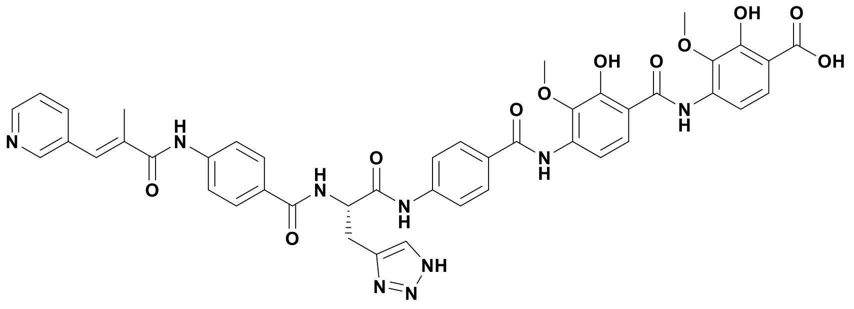

Chemical Formula: $C_{44}H_{39}N_9O_{11}$
Exact Mass: 869,2769

To a solution of tetrapeptide **37g** (80 mg, 111 µmol) in DMF (2 mL) was added perchlorophenyl (*E*)-4-(2-methyl-3-(pyridin -3-yl)acrylamido)benzoate (**22b**, 70.8 mg, 133 µmol) and NEt_3 (0.156 mL, 1.11 mmol). The reaction mixture was stirred for 2 d at 25°C and monitored by LC-MS (POM protected product: HRMS (ESI) calculated for $C_{50}H_{49}N_9O_{13}$ (M+H$^+$) 984.3523, found 984.3523). After full conversion KOH 3N (1 mL) was added and stirred for 20 min. Next, the mixture was neutralized with HCl 6N (0.5 mL) to a pH ~ 7. All volatiles were removed under reduced pressure, the residue was dissolved with DMSO (1 mL) and the salts were separated by centrifugation. The supernatant was loaded on the RP-HPLC system and purified under the following conditions. The collected fractions were lyophilized after their identity and purity was verified by LCMS to obtain 25 mg as a white solid (28.7 µmol, 25% over 2 steps).
RP-HPLC: 37-47% MeCN (+0.1% TFA) in milliQ H_2O (+0.1% TFA); 30 min; 70 mL/min.

^{1}H NMR (700 MHz, DMSO-d_6) δ 11.53 (s, 1H), 11.18 (s, 1H), 10.53 (s, 1H), 10.26 (s, 1H), 9.67 (s, 1H), 8.79 – 8.76 (m, 1H), 8.73 (d, *J* = 7.7 Hz, 1H), 8.66 – 8.62 (m, 1H), 8.11 (d, *J* = 7.9 Hz, 1H), 8.06 (d, *J* = 8.9 Hz, 1H), 7.99 – 7.95 (m, 2H), 7.92 – 7.87 (m, 2H), 7.85 – 7.79 (m, 4H), 7.81 – 7.77 (m, 2H), 7.67 (dd, *J* = 16.1, 9.2 Hz, 2H), 7.62 – 7.56 (m, 2H), 7.36 (d, *J* = 1.7 Hz, 1H), 4.92 (ddd, *J* = 9.1, 7.5, 5.7 Hz, 1H), 3.92 (s, 3H), 3.78 (s, 3H), 3.30 (dd, *J* = 14.8, 5.7 Hz, 1H), 3.24 (dd, *J* = 14.8, 9.2 Hz, 1H), 2.15 (d, *J* = 1.5 Hz, 3H);
^{13}C NMR (176 MHz, DMSO – from HSQC_ed) δ 148.5, 147.1, 139.2, 110.7, 119.0, 129.3, 128.8, 119.6, 119.7, 126.0, 119.2, 124.9, 126.2, 115.3, 129.7, 54.7, 60.6, 61.0, 27.8, 15.0;
HRMS (ESI) calculated for $C_{44}H_{40}N_9O_{11}$ (M+H$^+$) 870.2842, found 870.2833, t_R = 6.78 min.

1.2.4 Synthesis of B-Variations (5, 6)

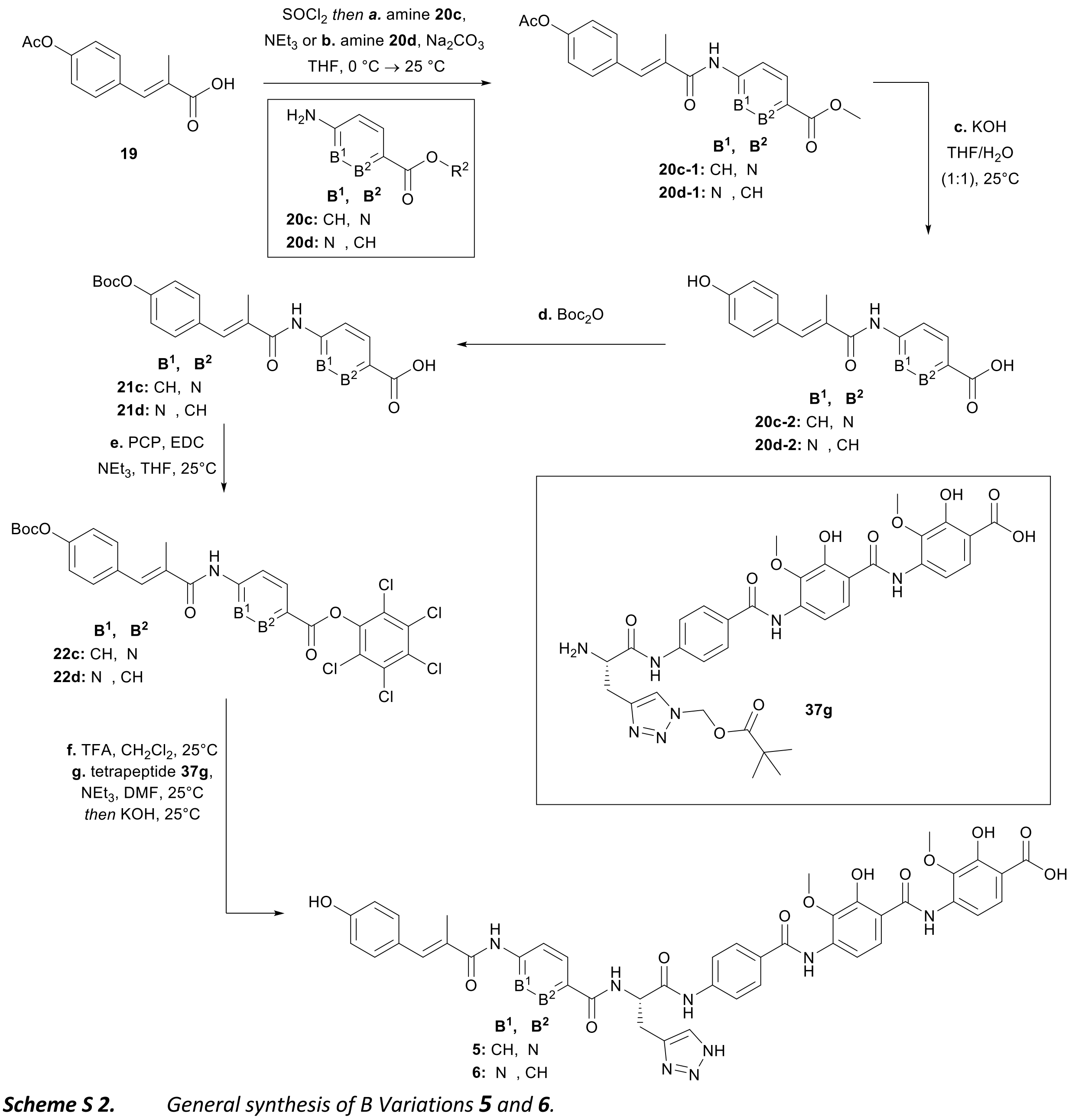

Scheme S 2. *General synthesis of B Variations **5** and **6**.*

(*E*)-3-(4-acetoxyphenyl)-2-methylacrylic acid (19)[9]

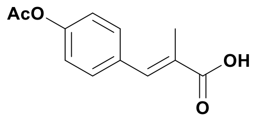

Chemical Formula: $C_{12}H_{12}O_4$
Exact Mass: 220,0736

Synthesized according to exp. procedure reported.

^{1}H NMR (400 MHz, DMSO-*d*$_6$) δ 12.51 (s, 1H), 7.59 (d, *J* = 1.7 Hz, 1H), 7.52 (d, *J* = 8.4 Hz, 2H), 7.19 (d, *J* = 8.6 Hz, 2H), 2.28 (s, 3H), 2.03 (d, *J* = 1.5 Hz, 3H).
^{13}C NMR (101 MHz, DMSO) δ 169.3, 169.1, 150.3, 136.7, 133.1, 130.8, 128.7, 121.9, 20.8, 13.9.
HRMS (ESI) calculated for $C_{12}H_{13}O_4$ (M−H$^+$) 219.0663, found 219.0664.

Methyl (E)-5-(3-(4-acetoxyphenyl)-2-methylacrylamido)picolinate (20c-1)

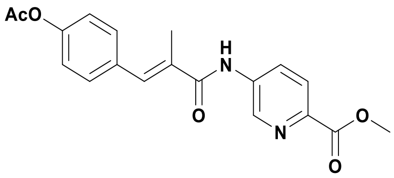

Chemical Formula: $C_{19}H_{18}N_2O_5$
Exact Mass: 354,1216

To a solution of compound **19** (395 mg, 1.79 mmol) in toluene (4 mL) was added $SOCl_2$ (0.39 mL, 5.38 mmol) and the resulting solution was refluxed for 3 h. All volatiles were removed under reduced pressure and co-evaporated twice with n-hexane. The residue was dissolved in THF (1.5 mL), the solution was dropwise added to a mixture of DIPEA (0.61 mL, 3.59 mmol) and 5-amino-picolinic acid methyl ester (**20c**, 210 mg, 1.38 mmol) in THF (1.5 mL) at 0°C. The solution was allowed to room temperature and stirred for 1 h. The crude product was precipitated by the addition of Et_2O (15 mL) at 0 °C, filtered and washed with Et_2O (15 mL) to give a 470 mg of a light-yellow solid (1.32 mmol, 96 %) that was directly used in the next step.

^{1}H NMR (500 MHz, DMSO-*d*$_6$) δ 10.46 (s, 1H), 9.00 (dd, *J* = 2.5, 0.7 Hz, 1H), 8.37 (dd, *J* = 8.6, 2.5 Hz, 1H), 8.07 (dd, *J* = 8.7, 0.6 Hz, 1H), 7.54 (d, *J* = 8.6 Hz, 2H), 7.41 (d, *J* = 1.7 Hz, 1H), 7.22 (d, *J* = 8.6 Hz, 2H), 3.86 (s, 3H), 2.29 (s, 3H), 2.14 (d, *J* = 1.5 Hz, 3H).
^{13}C NMR (126 MHz, DMSO) δ 169.1, 168.9, 164.8, 150.2, 141.5, 141.2, 139.0, 133.5, 133.1, 132.3, 130.6, 126.5, 125.4, 122.0, 52.1, 20.9, 14.3.
HRMS (ESI) calculated for $C_{19}H_{19}N_2O_5$ (M+H$^+$) 355.1288, found 355.1278.

(E)-5-(3-(4-hydroxyphenyl)-2-methylacrylamido)picolinic acid (20c-2)

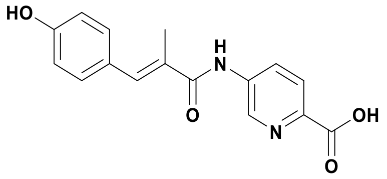

Chemical Formula: $C_{16}H_{14}N_2O_4$
Exact Mass: 298,0954

To a solution of compound **20c-1** (470, 1.33 mmol) in THF (2 mL) and MeOH (4 mL) was added aqueous KOH 5N (1.3 mL, 6.63 mmol). The resulting mixture was stirred at room temperature for 2 h. All volatiles were removed under reduced pressure, to the residue was added water (50 mL) and the resulting mixture was acidified to pH 4 by the addition of HCl 1N and extracted with EtOAc (3 x 30 mL). The combined organic phases were washed with brine (30 mL), dried over sodium sulphate and evaporated under vacuum to give 333 mg of a colorless solid (1.12 mmol, 84 %).

^{1}H NMR (400 MHz, DMSO-d_6) δ 10.55 (s, 1H), 9.08 (d, J = 2.4 Hz, 1H), 8.44 (dd, J = 8.7, 2.4 Hz, 1H), 8.11 (d, J = 8.6 Hz, 1H), 7.40 – 7.33 (m, 3H), 6.86 (d, J = 8.2 Hz, 2H), 2.12 (s, 3H).
^{13}C NMR (101 MHz, DMSO) δ 169.3, 164.9, 157.9, 140.8, 139.6, 135.2, 131.8, 131.5, 128.7, 127.9, 126.4, 125.7, 115.5, 14.5.
HRMS (ESI) calculated for $C_{16}H_{15}N_2O_4$ (M-H$^+$) 297.0881, found 297.0884.

(E)-5-(3-(4-((tert-butoxycarbonyl)oxy)phenyl)-2-methylacrylamido)picolinic acid (21c)

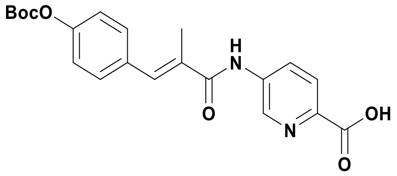

Chemical Formula: $C_{21}H_{22}N_2O_6$
Exact Mass: 398,1478

To a solution of compound **20c-2** (300 mg, 1.01 mmol) and DMAP (12 mg, 0.10 mmol) in THF (3 mL) was added Boc_2O (0.24 mL, 1.11 mmol) consecutively. The resulting mixture was stirred at room temperature for 3 h. The solvent was partially evaporated under reduced pressure, the residue was dissolved in EtOAc (50 mL). The organic phase was washed with aqueous $KHSO_4$ 10 % (50 mL) and brine (50 mL), dried over sodium sulphate and evaporated under vacuum to give 369 mg of a colorless solid (0.93 mmol, 93 %).

^{1}H NMR (400 MHz, DMSO-d_6) δ 10.47 (s, 1H), 8.98 (d, J = 2.5 Hz, 1H), 8.33 (dd, J = 8.6, 2.5 Hz, 1H), 8.05 (d, J = 8.6 Hz, 1H), 7.54 (d, J = 8.7 Hz, 2H), 7.39 (d, J = 1.7 Hz, 1H), 7.28 (d, J = 8.7 Hz, 2H), 2.12 (d, J = 1.5 Hz, 3H), 1.49 (s, 9H).
^{13}C NMR (101 MHz, DMSO) δ 140.8, 133.3, 131.3, 130.6, 126.6, 125.2, 121.5, 115.3, 27.1, 14.2.
HRMS (ESI) calculated for $C_{21}H_{23}N_2O_6$ (M+H$^+$) 297.0881, found 297.0883.

Perchlorophenyl (E)-5-(3-(4-((tert-butoxycarbonyl)oxy)phenyl)-2-methylacrylamido)picolinate (22c)

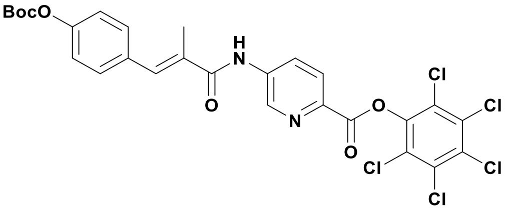

Chemical Formula: $C_{27}H_{21}Cl_5N_2O_6$
Exact Mass: 643,9842

To a solution of compound **21c** (340 mg 0.85 mmol), DIPEA (0.4 mL, 2.35 mmol) and DMAP (10 mg, 0.09 mmol) in THF (4 mL) was added EDC (196 mg, 1.02 mmol). The resulting mixture was stirred at room temperature for 5 min before pentachlorophenol 250 mg, 0.94 mmol) was added and the mixture was stirred at room temperature for 18 h. The solvent was evaporated under reduced pressure, the residue was triturated with dichloromethane and chromatographed on silica gel with a gradient of 5-25 % ethyl acetate in dichloromethane to give 71 mg of a light brown solid (0.11 mmol, 12 %).

^{1}H NMR (400 MHz, Chloroform-*d*) δ 8.83 (d, *J* = 2.6 Hz, 1H), 8.61 (dd, *J* = 8.7, 2.5 Hz, 1H), 8.34 (d, *J* = 8.7 Hz, 1H), 8.05 (s, 1H), 7.47 (s, 1H), 7.44 – 7.36 (m, 2H), 7.24 (d, *J* = 8.6 Hz, 2H), 2.25 (d, *J* = 1.3 Hz, 3H).
^{13}C NMR (101 MHz, $CDCl_3$) δ 168.2, 160.8, 151.8, 151.2, 144.4, 141.5, 140.3, 139.1, 135.4, 132.8, 132.3, 132.0, 130.8, 127.9, 127.8, 126.9, 121.7, 84.2, 27.8, 14.6.
HRMS (ESI) calculated for $C_{27}H_{22}Cl_5N_2O_6$ (M+H$^+$) 646.9886, found 646.9886.

(S,*E*)-2-hydroxy-4-(2-hydroxy-4-(4-(2-(5-(3-(4-hydroxyphenyl)-2-methylacrylamido)picolinamido)-3-(1H-1,2,3-triazol-4-yl)propanamido)benzamido)-3-methoxybenzamido)-3-methoxybenzoic acid (5)

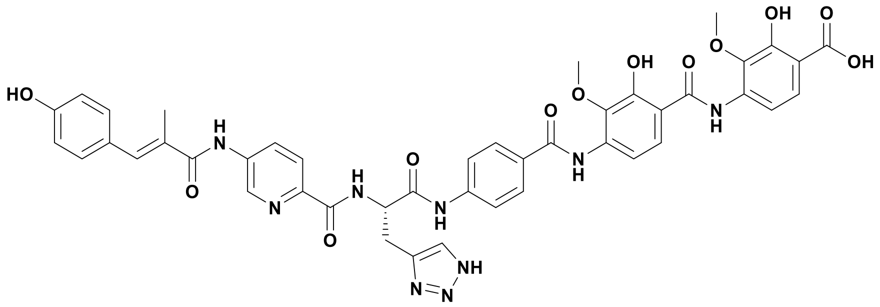

Chemical Formula: $C_{44}H_{39}N_9O_{12}$
Exact Mass: 885.2718

Compound **22c** (65 mg, 102 µmol) was dissolved in mixture of TFA and dichloromethane (1:4, 2 mL) and stirred at 25°C for 1 h. All volatiles were removed under reduced pressure and co-evaporated twice with n-hexane. The residue was dissolved in DMF (1 mL), the solution was dropwise added to a mixture of DIPEA (79 µL, 0.46 mmol) and compound **37g** (70 mg, 97 µmol) in DMF (1 mL) at 0°C. The resulting mixture was stirred at room temperature for 18 h. Afterwards KOH 5N (1 mL) was added and stirred for 1 h. The solution was diluted with water (20 mL) acidified to pH 7 by the addition of HCl 3N and lyophilized. The resulting residue was loaded on the RP-HPLC system and purified under the following conditions. The collected fractions were lyophilized after their identity and purity was verified by LCMS to obtain 7 mg as a white solid (7.9 µmol, 8 % over 3 steps).
RP-HPLC: 36-52% MeCN (+0.1% TFA) in milliQ H_2O (+0.1% TFA); 30 min; 50 mL/min.

^{1}H NMR (500 MHz, DMSO-*d*$_6$) δ 11.52 (s, 1H), 11.18 (s, 1H), 10.55 (s, 1H), 10.36 (s, 1H), 9.80 (s, 1H), 9.68 (s, 1H), 8.98 (t, *J* = 2.7 Hz, 1H), 8.79 (d, *J* = 8.1 Hz, 1H), 8.34 (dt, *J* = 8.6, 2.6 Hz, 1H), 8.06 (dd, *J* = 8.8, 3.8 Hz, 1H), 8.01 (d, *J* = 8.5 Hz, 1H), 7.98 (d, *J* = 8.6 Hz, 2H), 7.81 (d, *J* = 8.9 Hz, 1H), 7.75 (d, *J* = 8.6 Hz, 2H), 7.59 (dd, *J* = 9.4, 7.5 Hz, 3H), 7.40 – 7.35 (m, 2H), 7.33 (d, *J* = 1.6 Hz, 1H), 6.88 – 6.82 (m, 2H), 4.99 (q, *J* = 7.1 Hz, 1H), 3.92 (s, 3H), 3.78 (d, *J* = 1.5 Hz, 3H), 3.33 (d, *J* = 6.6 Hz, 2H), 2.13 (d, *J* = 1.4 Hz, 3H).
^{13}C NMR (126 MHz, DMSO – from HSQC_ed) δ 140.5, 127.7, 110.7, 122.9, 129.3, 125.9, 119.3, 125.9, 115.9, 131.9, 135.2, 115.9, 53.9, 60.7, 60.9, 28.3, 14.9.
HRMS (ESI) calculated for $C_{44}H_{40}N_9O_{12}$ (M+H$^+$) 886.2791, found 886.2778, t_R = 8.09 min.

Methyl (*E*)-6-(3-(4-acetoxyphenyl)-2-methylacrylamido)nicotinate (20d-1)

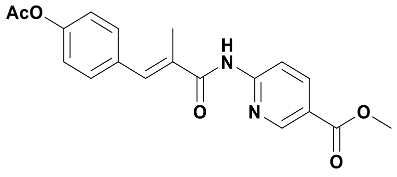

Chemical Formula: $C_{19}H_{18}N_2O_5$
Exact Mass: 354,1216

To a solution of compound **19** (1.74 g, 7.89 mmol) was added $SOCl_2$ (4.77 mL, 65.7 mmol) and the resulting solution was refluxed for 2 h. All volatiles were removed under reduced pressure and co-evaporated twice with n-hexane. The residue was dissolved in THF (5 mL), the solution was dropwise added to a mixture of Na_2CO_3 (1.39 g, 13.1 mmol) and methyl 6-aminonicotinate (**20d**, 1.00 g, 6.57 mmol) in THF (5 mL) at 0°C. The solution was allowed to room temperature and stirred for 1 h. The crude product was precipitated by the addition of Et_2O (15 mL) at 0 °C, filtered and washed with Et_2O (15 mL). The residue was dissolved in CH_2Cl_2 (50 mL), washed with water (2 x 50 mL), dried over sodium sulfate, filtered, and concentrated under reduced pressure to give 1.72 g of a light-yellow solid (4.85 mmol, 74 %) that was directly used in the next step.

^{1}H NMR (400 MHz, Chloroform-*d*) δ 8.92 (d, *J* = 2.2 Hz, 1H), 8.62 (s, 1H), 8.41 (dd, *J* = 8.8, 1.0 Hz, 1H), 8.33 (ddd, *J* = 8.8, 2.2, 1.0 Hz, 1H), 7.49 (s, 1H), 7.47 – 7.37 (m, 2H), 7.14 (d, *J* = 8.7 Hz, 2H), 3.93 (s, 3H), 2.32 (s, 3H), 2.23 (s, 3H).
^{13}C NMR (101 MHz, $CDCl_3$) δ 169.4, 167.8, 165.5, 154.6, 150.7, 150.1, 140.0, 135.3, 133.2, 132.0, 130.8, 122.2, 121.9, 113.2, 52.4, 21.3, 14.4.
HRMS (ESI) calculated for $C_{19}H_{19}N_2O_5$ $(M+H^+)$ 355.1288, found 355.1285.

(*E*)-6-(3-(4-hydroxyphenyl)-2-methylacrylamido)nicotinic acid (20d-2)

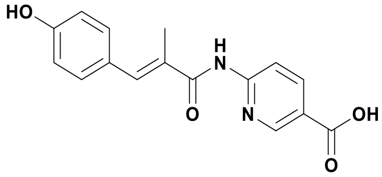

Chemical Formula: $C_{16}H_{14}N_2O_4$
Exact Mass: 298,0954

To a solution of compound **20d-1** (400 mg, 1.13 mmol) in THF (5 mL) and water (3.7 mL) was added aqueous KOH 5 N (1.3 mL, 6.63 mmol). The resulting mixture was stirred at room temperature for 2 h. All volatiles were removed under reduced pressure, to the residue was added water (20 mL) and the resulting mixture was acidified to pH 4 by the addition of HCl and extracted with EtOAc (3 x 30 mL). The combined organic phases were washed with brine (30 mL), dried over sodium sulphate and evaporated under vacuum to give 310 mg of a colorless solid (1.13 mmol, 92%).

^{1}H NMR (400 MHz, DMSO-d_6) δ 10.64 (s, 1H), 9.86 (s, 1H), 8.85 (d, *J* = 2.3 Hz, 1H), 8.30 – 8.18 (m, 2H), 7.42 – 7.32 (m, 3H), 6.84 (d, *J* = 8.2 Hz, 2H), 2.11 (s, 3H).
^{13}C NMR (101 MHz, DMSO) δ 169.4, 166.0, 157.8, 155.5, 149.6, 139.2, 135.4, 131.5, 128.4, 126.5, 121.9, 115.4, 113.1, 14.4.
HRMS (ESI) calculated for $C_{16}H_{15}N_2O_4$ $(M+H^+)$ 299.1026, found 299.1028.

(E)-6-(3-(4-((tert-butoxycarbonyl)oxy)phenyl)-2-methylacrylamido)nicotinic (21d)

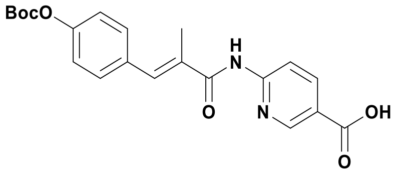

Chemical Formula: $C_{21}H_{22}N_2O_6$
Exact Mass: 398,1478

To a solution of compound **20d-2** (305 mg, 1.02 mmol) and DMAP (12 mg, 0.10 mmol) in THF (3 mL) was added Boc_2O (0.258 mL, 1.12 mmol) consecutively. The resulting mixture was stirred at room temperature for 3 h. The solvent was partially evaporated under reduced pressure, the residue was dissolved in EtOAc (50 mL). The organic phase was washed with aqueous $KHSO_4$ 10 % (50 mL) and brine (50 mL), dried over sodium sulphate and evaporated under vacuum to give 352 mg of a colorless solid (0.88 mmol, 86%).

^{1}H NMR (400 MHz, DMSO-d_6) δ 10.79 (s, 1H), 8.86 (dd, J = 2.3, 0.8 Hz, 1H), 8.33 – 8.20 (m, 2H), 7.52 (d, J = 8.7 Hz, 2H), 7.45 (d, J = 1.7 Hz, 1H), 7.27 (d, J = 8.6 Hz, 2H), 2.11 (d, J = 1.4 Hz, 3H), 1.50 (s, 9H).
^{13}C NMR (101 MHz, DMSO) δ 169.1, 166.1, 155.2, 151.1, 150.2, 149.6, 139.3, 133.9, 133.4, 131.9, 130.7, 121.5, 113.2, 83.5, 59.8, 27.3, 14.3.
HRMS (ESI) calculated for $C_{21}H_{23}N_2O_6$ (M+H$^+$) 399.1551, found 399.1557.

Perchlorophenyl (E)-6-(3-(4-((tert-butoxycarbonyl)oxy)phenyl)-2-methylacrylamido)nicotinate (22d)

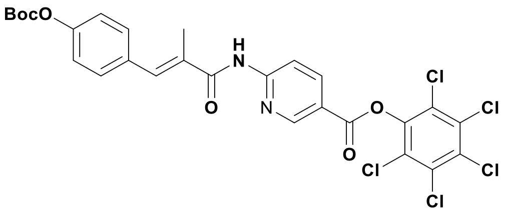

Chemical Formula: $C_{27}H_{21}Cl_5N_2O_6$
Exact Mass: 643.9842

To a solution of compound **21d** (325 mg 0.816 mmol), DIPEA (355 mL, 2.04 mmol) and DMAP (10 mg, 82 µmol) in THF (4 mL) was added EDC (152 mg, 0.979 mmol). The resulting mixture was stirred at room temperature for 5 min before pentachlorophenol (230 mg, 0.865 mmol) was added and the mixture was stirred at room temperature for 18 h. The solvent was evaporated under reduced pressure, the residue was triturated with dichloromethane and chromatographed on silica gel with a gradient of 5-25 % ethyl acetate in dichloromethane to give 93 mg of a light brown solid (816 µmol, 18%).

^{1}H NMR (400 MHz, Chloroform-d) δ 9.11 (t, J = 1.6 Hz, 1H), 8.68 (s, 1H), 8.52 (d, J = 1.8 Hz, 2H), 7.55 – 7.50 (m, 1H), 7.42 (d, J = 8.8 Hz, 2H), 7.25 (s, 1H), 7.23 (s, 1H), 2.25 (d, J = 1.4 Hz, 3H), 1.58 (s, 9H).
^{13}C NMR (101 MHz, $CDCl_3$) δ 167.9, 161.1, 155.8, 151.8, 151.2, 144.1, 141.0, 135.8, 133.0, 132.3, 132.0, 131.8, 130.8, 128.0, 121.6, 119.5, 113.5, 84.1, 27.8, 14.4.

(S,*E*)-2-hydroxy-4-(2-hydroxy-4-(4-(2-(6-(3-(4-hydroxyphenyl)-2-methylacrylamido)nicotinamido)-3-(1H-1,2,3-triazol-4-yl)propanamido)benzamido)-3-methoxybenzamido)-3-methoxybenzoic acid (6)

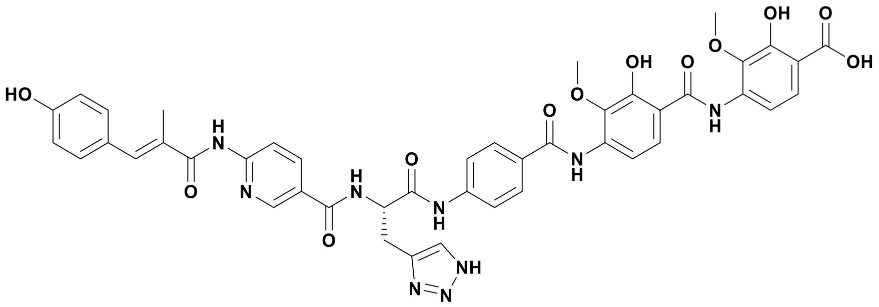

Chemical Formula: $C_{44}H_{39}N_9O_{12}$
Exact Mass: 885.2718

Compound **22d** (54 mg, 84 µmol) was dissolved in mixture of TFA and dichloromethane (1:4, 2 mL) and stirred at 25°C for 1 h. All volatiles were removed under reduced pressure and co-evaporated twice with n-hexane. The residue was dissolved in DMF (1 mL), the solution was dropwise added to a mixture of DIPEA (68 µL, 0.38 mmol) and compound **37g** (55 mg, 76.4 µmol) in DMF (1 mL) at 0°C. The resulting mixture was stirred at room temperature for 18 h. Afterwards KOH 5 N (1 mL) was added and stirred for 1 h. The solution was diluted with water (20 mL) acidified to pH 7 by the addition of HCl and lyophilized. The resulting residue was loaded on the RP-HPLC system and purified under the following conditions. The collected fractions were lyophilized after their identity and purity was verified by LCMS to obtain 8 mg as a white solid (9.03 µmol, 12% over 3 steps).
RP-HPLC: 36-52% MeCN (+0.1% TFA) in milliQ H_2O (+0.1% TFA); 30 min; 50 mL/min.

^{1}H NMR (500 MHz, DMSO-d_6) δ 11.52 (s, 1H), 11.15 (d, J = 28.8 Hz, 1H), 10.59 (s, 1H), 10.53 (s, 1H), 9.78 (s, 1H), 9.67 (s, 1H), 8.96 (d, J = 7.6 Hz, 1H), 8.85 (d, J = 2.4 Hz, 1H), 8.26 (dd, J = 8.7, 2.4 Hz, 1H), 8.20 (d, J = 8.8 Hz, 1H), 8.06 (dd, J = 8.9, 3.9 Hz, 1H), 7.97 (d, J = 8.6 Hz, 2H), 7.80 (dd, J = 12.1, 8.7 Hz, 3H), 7.69 (s, 1H), 7.63 – 7.53 (m, 2H), 7.41 (s, 1H), 7.38 – 7.33 (m, 2H), 6.86 – 6.81 (m, 2H), 4.95 (q, J = 7.5 Hz, 1H), 3.92 (s, 3H), 3.78 (s, 3H), 3.32 (dd, J = 14.8, 5.7 Hz, 1H), 3.23 (dd, J = 14.7, 9.1 Hz, 1H), 2.11 (d, J = 1.3 Hz, 3H).
^{13}C NMR (126 MHz, DMSO – from HSQC_ed) δ 147.6, 137.5, 112.9, 110.4, 128.9, 125.8, 118.9, 115.5, 125.8, 135.4, 131.6, 115.5, 60.6, 60.9, 14.8.
HRMS (ESI) calculated for $C_{44}H_{40}N_9O_{12}$ (M+H$^+$) 886.2791, found 886.2772, t_R = 7.82 min.

1.2.5 Synthesis of F-Variations (12, 13, 15)

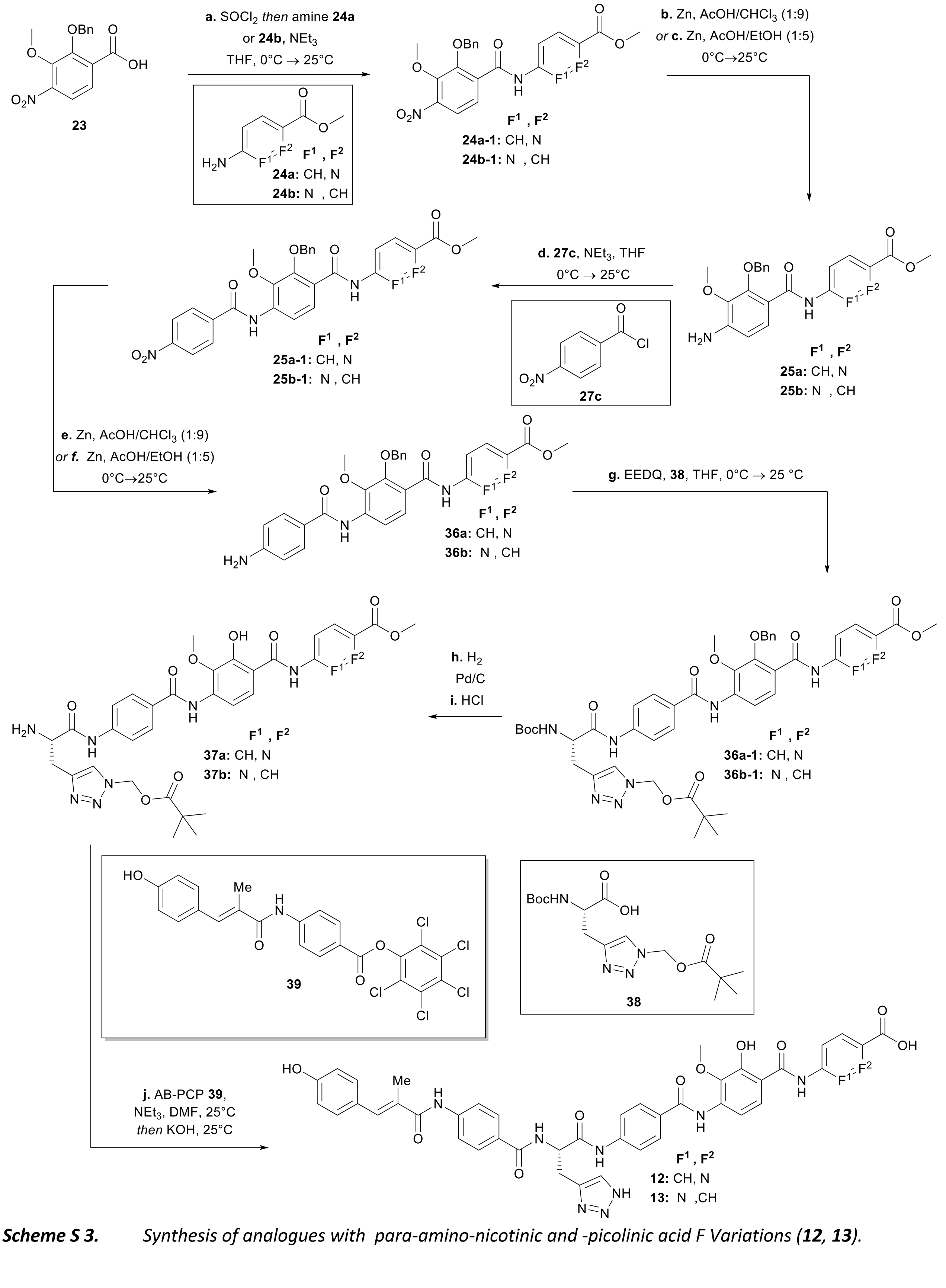

Scheme S 3. *Synthesis of analogues with para-amino-nicotinic and -picolinic acid F Variations (**12, 13**).*

2-(Benzyloxy)-3-methoxy-4-nitrobenzoic acid (23)

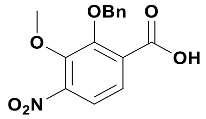

Chemical Formula: $C_{15}H_{13}NO_6$
Exact Mass: 303,0743

To a solution of benzyl 2-(benzyloxy)-3-methoxy-4-nitrobenzoate (**31-2**, 26.0 g, 68.0 mmol) in THF (100 mL) was added a solution of lithium hydroxide (11.1 g, 462 mmol) in water (100 mL). The reaction mixture was stirred for 3.5 h at 25°C. The organic solvent was removed under reduced pressure and the aqueous residue phase was acidified with HCl 1 N. The precipitate was filtered and dried in vacuo to obtain 18.1 g of a colorless solid (59.3 mmol, 90%).

^{1}H NMR (500 MHz, DMSO-d_6) δ 7.72 (d, J = 8.6 Hz, 1H), 7.57 (d, J = 8.5 Hz, 1H), 7.51 – 7.48 (m, 2H), 7.42 – 7.32 (m, 3H), 5.07 (s, 2H), 3.93 (s, 3H).
^{13}C NMR (126 MHz, DMSO) δ 166.0, 151.5, 146.7, 146.1, 136.4, 132.5, 128.4, 128.3, 128.0, 124.9, 119.3, 75.8, 62.3.
HRMS (ESI) calculated for $C_{15}H_{14}NO_6$ (M-H$^-$) 302.0670, found 302.0662.

Methyl 5-(2-(benzyloxy)-3-methoxy-4-nitrobenzamido)picolinate (24a-1)

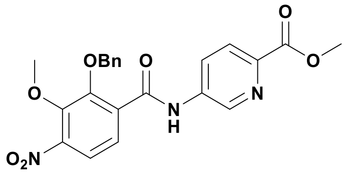

Chemical Formula: $C_{22}H_{19}N_3O_7$
Exact Mass: 437,1223

2-(Benzyloxy)-3-methoxy-4-nitrobenzoic acid (**23**, 2.63 g, 8.68 mmol) was dissolved in thionyl chloride (6.22 mL, 86.8 mmol) and refluxed for 2 h at 90°C. The thionyl chloride was removed under reduced pressure and the resulting acid chloride was dissolved in THF (10 mL). In parallel was prepared a mixture of methyl 5-aminopicolinate (**24a**, 1.10 g, 8.68 mmol) and triethylamine (2.02 mL, 14.5 mmol) in THF (10 mL). To the mixture was dropwise added the acid chloride solution at 0°C. The solution was allowed to warm to ambient temperature and stirred for 16 h. Diethylether (75 mL) was added to the reaction mixture and cooled to 0°C. The precipitate was collected by filtration and dissolved in dichloromethane (100 mL), water (100 mL) was added. The organic phase was washed with a saturated solution of $NaHCO_3$ (2 x 75 mL), HCl 1 N (3 x 75 mL) and brine (75 mL), dried over sodium sulfate and evaporated under reduced pressure to give 2.54 g of a white solid (5.81 mmol, 80%) without further purification.

^{1}H NMR (500 MHz, DMSO-d_6) δ 11.08 (s, 1H), 10.35 (s, 1H), 8.89 – 8.85 (m, 1H), 8.31 (dd, J = 8.6, 2.6 Hz, 1H), 8.10 (d, J = 8.6 Hz, 1H), 7.79 (d, J = 8.4 Hz, 1H), 7.53 (d, J = 8.4 Hz, 1H), 7.40 – 7.33 (m, 2H), 7.33 – 7.25 (m, 3H), 5.14 (s, 2H), 4.01 (s, 3H), 3.87 (s, 3H).
^{13}C NMR (126 MHz, DMSO) δ 164.7, 164.1, 150.1, 146.1, 145.8, 142.1, 140.8, 138.2, 136.0, 135.8, 128.4, 128.3, 126.3, 125.6, 123.8, 119.5, 76.1, 62.3, 52.2.
HRMS (ESI) calculated for $C_{22}H_{20}N_3O_7$ (M+H$^+$) 438.1296, found 438.1295.

Methyl 5-(4-amino-2-(benzyloxy)-3-methoxybenzamido)picolinate (25a)

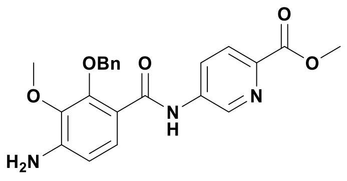

Chemical Formula: $C_{22}H_{21}N_3O_5$
Exact Mass: 407,1481

To methyl 5-(2-(benzyloxy)-3-methoxy-4-nitrobenzamido)picolinate (**24-1a**, 2.24 g, 5.11 mmol) in a mixture of chloroform (90 mL) and acetic acid (10 mL) at 0°C was added zinc dust (6.69 g, 102 mmol) portion wise. The ice bath was removed after 10 min and the reaction mixture was stirred for further 30 min. The reaction was monitored by TLC, after full conversion the suspension was filtered over celite® and washed with dichloromethane (100 mL). The filtrate was washed with a saturated solution of $NaHCO_3$ (3 x 100 mL) and brine (100 mL), dried over sodium sulfate and evaporated under reduced pressure to give 2.08 g of a yellow solid (4.94 mol, 96%) without further purification.

^{1}H NMR (400 MHz, DMSO-d_6) δ 10.24 (s, 1H), 8.43 (ddd, J = 14.5, 2.6, 0.7 Hz, 1H), 8.14 (dd, J = 8.7, 2.6 Hz, 1H), 7.98 (dd, J = 8.7, 1.9 Hz, 1H), 7.47 (ddd, J = 4.7, 2.3, 1.3 Hz, 1H), 7.46 – 7.37 (m, 2H), 7.37 – 7.29 (m, 3H), 6.57 (d, J = 8.7 Hz, 1H), 5.84 (s, 2H), 5.17 (s, 2H), 3.85 (s, 3H), 3.81 (s, 3H).
^{13}C NMR (101 MHz, DMSO) δ 140.1, 128.4, 128.3, 128.3, 126.2, 125.3, 125.3, 109.7, 75.6, 59.5, 51.9.
HRMS (ESI) calculated for $C_{22}H_{22}N_3O_5$ (M+H$^+$) 408.1554, found 408.1546.

4-Amino-2-(benzyloxy)-N-(6-(hydroxymethyl)pyridin-3-yl)-3-methoxybenzamide (26)

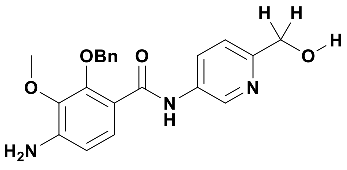

Chemical Formula: $C_{21}H_{21}N_3O_4$
Exact Mass: 379,1532

The compound 4-amino-2-(benzyloxy)-N-(6-(hydroxymethyl)pyridin-3-yl)-3-methoxybenzamide (**26**) was found as byproduct in the reaction towards methyl 5-(4-amino-2-(benzyloxy)-3-methoxybenzamido)picolinate (**25a**), by using the following procedure:
In a mixture of ethanol (250 mL) and acetic acid (50 mL) was dissolved methyl 5-(2-(benzyloxy)-3-methoxy-4-nitrobenzamido)picolinate (**24-1a**, 4.00 g, 9.14 mmol). At 0°C was added Zn (12.0 g, 183 mmol) in portions. The mixture was stirred for 20 min and allowed to warm to 25°C. Next, the suspension was filtered through celite® and washed with EtOAc. The filtrate was evaporated under reduced pressure. To the residue were added EtOAc (200 mL) and a saturated solution of $NaHCO_3$ (200 mL), the organic phase was washed with $NaHCO_3$ (3 x 200 mL) and brine (200 mL), dried over sodium sulphate and evaporated under reduced pressure. The residue was purified by column chromatography (1.5→4% MeOH in dichloromethane) to give 1.10 g of a yellow solid (2.90 mmol, 32%) of the described byproduct (**26**) and 1.09 g of a yellow solid of the desired methyl 5-(4-amino-2-(benzyloxy)-3-methoxybenzamido)picolinate (**25a**, 2.70 mmol, 30%).

^{1}H NMR (400 MHz, DMSO-d_6) δ 9.94 (s, 1H), 8.31 (d, J = 2.5 Hz, 1H), 7.84 (dd, J = 8.5, 2.6 Hz, 1H), 7.50 (dd, J = 3.5, 1.5 Hz, 1H), 7.49 – 7.30 (m, 7H), 6.57 (d, J = 8.6 Hz, 1H), 5.76 (s, 2H), 5.34 (t, J = 5.8 Hz, 1H), 5.18 (s, 2H), 4.49 (d, J = 5.8 Hz, 2H), 3.81 (s, 3H).
^{13}C NMR (101 MHz, DMSO) δ 139.4, 128.4, 128.3, 128.3, 126.5, 126.1, 119.7, 109.7, 75.4, 63.7, 59.5.
HRMS (ESI) calculated for $C_{21}H_{22}N_3O_4$ (M+H$^+$) 380.1605, found 380.1596.

Methyl 5-(2-(benzyloxy)-3-methoxy-4-(4-nitrobenzamido)benzamido)picolinate (25a-1)

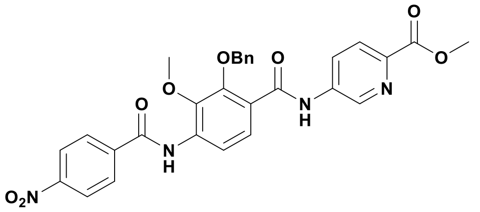

Chemical Formula: $C_{29}H_{24}N_4O_8$
Exact Mass: 556,1594

To a mixture of methyl 5-(4-amino-2-(benzyloxy)-3-methoxybenzamido)picolinate (**25a**, 1.10 g, 2.70 mmol) and triethylamine (0.752 mL, 5.40 mmol) was added dropwise a solution of 4-nitrobenzoyl chloride (**27c**, 1.50 g, 8.10 mmol) in THF (10 mL) at 0°C. The reaction mixture was allowed to warm to ambient temperature and stirred for 12 h. Diethylether (100 mL) was added to the reaction mixture and cooled to 0°C. The precipitate was collected by filtration and dissolved in dichloromethane (100 mL), the organic phase was washed with a saturated solution of $NaHCO_3$ (3 x 100 mL), HCl 1N (1 x 100 mL) and brine (1 x 100 mL), dried over sodium sulfate and concentrated under reduced pressure to give 1.38 g of a yellow solid (2.28 mmol, 92%) without further purification.

^{1}H NMR (400 MHz, DMSO-d_6) δ 10.78 (s, 1H), 10.31 (s, 1H), 8.81 (dd, *J* = 4.7, 2.5 Hz, 1H), 8.45 – 8.35 (m, 2H), 8.31 (dd, *J* = 8.7, 2.5 Hz, 1H), 8.27 – 8.16 (m, 2H), 8.08 (d, *J* = 8.6 Hz, 1H), 7.74 (d, *J* = 8.4 Hz, 1H), 7.49 – 7.32 (m, 4H), 7.29 (ddt, *J* = 5.4, 3.6, 2.2 Hz, 3H), 5.13 (s, 2H), 3.93 (s, 3H), 3.87 (s, 3H).
^{13}C NMR (101 MHz, DMSO) δ 164.9, 164.7, 164.3, 149.3, 145.9, 141.7, 140.7, 139.9, 138.5, 136.5, 134.4, 129.4, 128.6, 128.5, 128.4, 128.3, 127.7, 126.1, 125.6, 123.6, 120.2, 75.7, 60.9, 52.2.
HRMS (ESI) calculated for $C_{29}H_{25}N_4O_8$ (M+H$^+$) 557.1667, found 557.1675.

Methyl 5-(4-(4-aminobenzamido)-2-(benzyloxy)-3-methoxybenzamido)picolinate (36a)

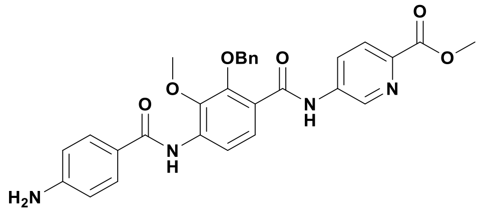

Chemical Formula: $C_{29}H_{26}N_4O_6$
Exact Mass: 526,1852

To methyl 5-(2-(benzyloxy)-3-methoxy-4-(4-nitrobenzamido)benzamido)picolinate (**25a-1**, 1.24 g, 2.23 mmol) in a mixture of chloroform (225 mL) and acetic acid (25 mL) at 0°C was added zinc dust (2.92 g, 44.7 mmol) portion wise. The ice bath was removed after 10 min and the reaction mixture was stirred for further 30 min. The reaction was monitored by TLC, after full conversion the suspension was filtered over celite® and washed with dichloromethane (100 mL). The filtrate was washed with a saturated solution of $NaHCO_3$ (3 x 100 mL) and brine (100 mL), dried over sodium sulfate and evaporated under reduced pressure to give to give 1.04 g of yellow solid (1.98 mmol, 89%) without further purification.

^{1}H NMR (400 MHz, DMSO-d_6) δ 10.68 (s, 1H), 9.25 (s, 1H), 8.77 (t, *J* = 4.5 Hz, 1H), 8.35 – 8.26 (m, 2H), 8.07 (d, *J* = 8.6 Hz, 1H), 7.91 (d, *J* = 8.6 Hz, 1H), 7.74 (dd, *J* = 9.1, 2.3 Hz, 2H), 7.50 – 7.38 (m, 4H), 7.34 – 7.27 (m, 3H), 6.64 (dd, *J* = 9.0, 2.3 Hz, 2H), 5.90 (s, 2H), 5.13 (s, 2H), 3.95 (s, 3H), 3.87 (s, 3H).
^{13}C NMR (101 MHz, DMSO) δ 165.0, 164.8, 164.7, 152.5, 149.3, 144.1, 141.6, 140.7, 138.5, 136.5, 135.9, 129.4, 128.7, 128.4, 128.3, 126.0, 125.6, 125.4, 124.0, 120.2, 118.0, 112.8, 79.2, 75.8, 60.9.
HRMS (ESI) calculated for $C_{29}H_{27}N_4O_6$ (M+H$^+$) 527.1925, found 527.1921.

Methyl (S)-5-(2-(benzyloxy)-4-(4-(2-((tert-butoxycarbonyl)amino)-3-(1-((pivaloyloxy)methyl)-1H-1,2,3-triazol-4-yl)propanamido)benzamido)-3-methoxybenzamido)picolinate (36a-1)

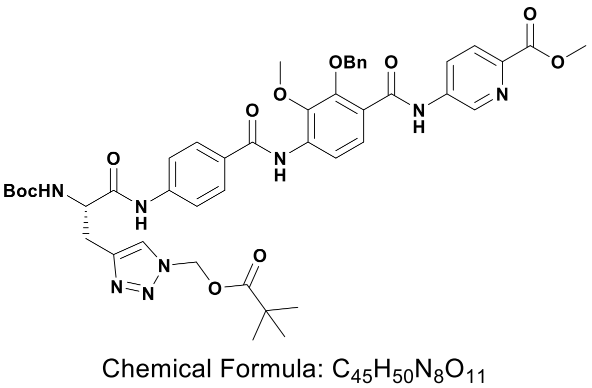

Chemical Formula: $C_{45}H_{50}N_8O_{11}$
Exact Mass: 878,3599

To a solution of EEDQ (704 mg, 2.85 mmol) in THF (10 mL) was added (S)-2-((tert-butoxycarbonyl)amino)-3-(1-((pivaloyloxy)methyl)-1H-1,2,3-triazol-4-yl)propanoic acid (**38**, 703 mg, 1.90 mmol) at 0°C and stirred for 15 min. Next a solution of - methyl 5-(4-(4-aminobenzamido)-2-(benzyloxy)-3-methoxybenzamido)picolinate (**36a**, 500 mg, 950 µmol) in THF (5 mL) was added at 0°C, the reaction mixture was allowed to warm to ambient temperature and stirred for 18 h. All volatiles were removed under reduced pressure and the residue was dissolved in dichloromethane, the organic phase was washed with HCl 1 N (50 mL), $NaHCO_3$ saturated solution (50 mL) and brine (50 mL), dried over sodium sulfate and evaporated. The residue was purified by column chromatography on silica gel (eluent: 0.3→3% MeOH in dichloromethane) to give 638 mg of a colorless solid (726 µmol, 76%).

^{1}H NMR (400 MHz, DMSO-d_6) δ 10.73 (s, 1H), 10.44 (s, 1H), 9.70 (s, 1H), 8.79 (d, *J* = 2.6 Hz, 1H), 8.31 (dd, *J* = 8.7, 2.5 Hz, 1H), 8.08 (d, *J* = 8.6 Hz, 1H), 8.02 – 7.96 (m, 3H), 7.82 (d, *J* = 8.5 Hz, 1H), 7.80 – 7.74 (m, 2H), 7.49 – 7.37 (m, 3H), 7.31 (d, *J* = 1.8 Hz, 1H), 7.30 – 7.20 (m, 3H), 6.30 (s, 2H), 5.13 (s, 2H), 4.41 (q, *J* = 8.1 Hz, 1H), 3.95 (s, 3H), 3.87 (s, 3H), 3.13 (dd, *J* = 14.7, 5.0 Hz, 1H), 3.00 (dd, *J* = 14.6, 9.4 Hz, 1H), 1.37 (s, 9H), 1.10 (s, 9H).
^{13}C NMR (101 MHz, DMSO) δ 176.9, 176.5, 164.9, 164.8, 155.4, 149.5, 145.2, 143.5, 142.2, 141.7, 140.7, 138.6, 136.5, 135.2, 128.7, 128.6, 128.4, 128.3, 126.6, 126.1, 125.6, 124.2, 123.8, 119.3, 118.7, 78.3, 75.8, 69.9, 60.9, 54.9, 52.2, 38.2, 28.2, 26.5.
HRMS (ESI) calculated for $C_{45}H_{51}N_8O_{11}$ (M+H$^+$) 879.3672, found 879.3685.

Methyl (S)-5-(4-(4-(2-((tert-butoxycarbonyl)amino)-3-(1-((pivaloyloxy)methyl)-1H-1,2,3-triazol-4-yl)propanamido)benzamido)-2-hydroxy-3-methoxybenzamido)picolinate (36a-2)

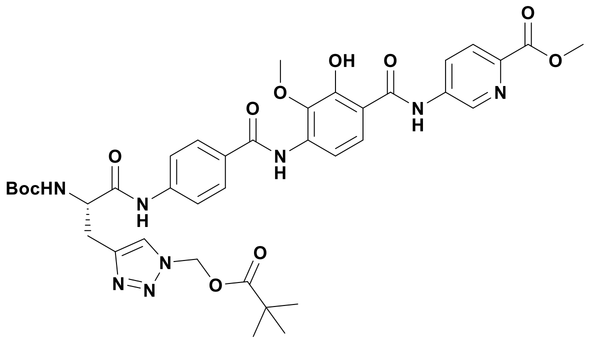

Chemical Formula: $C_{38}H_{44}N_8O_{11}$
Exact Mass: 788,3130

A mixture of Methyl (S)-5-(2-(benzyloxy)-4-(4-(2-((tert -butoxycarbonyl)amino)-3-(1-((pivaloyloxy)methyl)-1H-1,2,3-triazol-4-yl)propanamido)benzamido)-3-methoxybenzamido)picolinate (**36a-1**, 570 mg, 649 µmol) and 10% Pd/C (57 mg), in 1:1:1 mixture of EtOAc/MeOH/THF (60 mL), was stirred for 1 h under H_2 atmosphere. After the catalyst was filtered and washed with MeOH, the filtrate was evaporated to give 454 mg of a colorless solid (575 µmol, 89%).

^{1}H NMR (500 MHz, DMSO-d_6) δ 11.95 (s, 1H), 10.79 (s, 1H), 10.41 (s, 1H), 9.53 (s, 1H), 9.01 (dd, *J* = 2.5, 0.7 Hz, 1H), 8.39 (dd, *J* = 8.7, 2.5 Hz, 1H), 8.12 (dt, *J* = 8.6, 1.4 Hz, 1H), 7.99 – 7.92 (m, 3H), 7.85 – 7.79 (m, 1H), 7.79 – 7.74 (m, 2H), 7.68 (d, *J* = 8.8 Hz, 1H), 7.19 (d, *J* = 8.0 Hz, 1H), 6.29 (s, 2H), 4.38 (dq, *J* = 31.7, 7.8, 7.2 Hz, 1H), 3.88 (s, 3H), 3.87 (s, 3H), 3.12 (dd, *J* = 14.7, 5.1 Hz, 1H), 3.05 – 2.96 (m, 1H), 1.36 (s, 9H), 1.09 (s, 9H).
^{13}C NMR (126 MHz, DMSO) δ 176.4, 168.3, 164.7, 155.3, 153.3, 143.4, 142.3, 142.1, 140.7, 138.9, 137.9, 136.4, 134.9, 132.4, 128.7, 128.5, 127.8, 125.4, 124.1, 123.3, 118.7, 113.0, 112.7, 78.3, 69.8, 67.0, 59.7, 52.2, 48.6, 38.2, 28.1, 26.4.
HRMS (ESI) calculated for $C_{38}H_{45}N_8O_{11}$ (M+H$^+$) 789.3202, found 789.3215.

Methyl (S)-5-(4-(4-(2-amino-3-(1-((pivaloyloxy)methyl)-1H-1,2,3-triazol-4-yl)propanamido)benzamido)-2-hydroxy-3-methoxybenzamido)picolinate (37a)

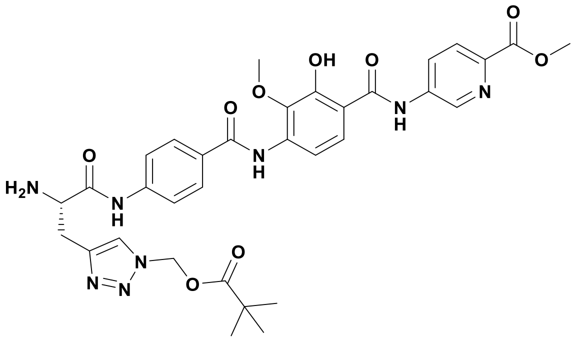

Chemical Formula: $C_{33}H_{36}N_8O_9$
Exact Mass: 688,2605

Methyl (S)-5-(4-(4-(2-((tert-butoxycarbonyl)amino)-3-(1-((pivaloyloxy)methyl)-1H-1,2,3-triazol-4-yl)propanamido)benzamido)-2-hydroxy-3-methoxybenzamido)picolinate (**36a-2**, 439 mg, 557 µmol) was dissolved in HCl 4N in dioxane (10 mL) and stirred for 1 h at 25°C. All volatiles were removed under reduced pressure, the residue was dissolved in 2:1 mixture of water/MeCN and lyophilized to give 392 mg of a white fluffy solid (540 µmol, 97%).

^{1}H NMR (500 MHz, DMSO-d_6) δ 11.26 (s, 1H), 10.94 (s, 1H), 9.57 (s, 1H), 9.06 (dd, *J* = 2.5, 0.7 Hz, 1H), 8.60 (d, *J* = 5.4 Hz, 3H), 8.42 (dd, *J* = 8.7, 2.5 Hz, 1H), 8.15 (s, 1H), 8.12 (dt, *J* = 8.8, 1.1 Hz, 1H), 8.00 – 7.94 (m, 2H), 7.91 (d, *J* = 9.0 Hz, 1H), 7.79 – 7.73 (m, 2H), 7.65 (d, *J* = 8.9 Hz, 1H), 6.28 (s, 2H), 4.42 (q, *J* = 6.0 Hz, 1H), 3.88 (s, 3H), 3.86 (s, 3H), 3.36 (d, *J* = 6.6 Hz, 2H), 1.07 (s, 9H).
^{13}C NMR (126 MHz, DMSO) δ 176.4, 168.4, 166.8, 164.7, 164.7, 153.4, 142.1, 142.0, 141.4, 140.9, 139.0, 138.0, 136.4, 129.3, 128.7, 128.1, 125.5, 125.0, 123.5, 119.0, 113.0, 112.8, 69.9, 60.3, 52.7, 52.3, 38.1, 26.4.
HRMS (ESI) calculated for $C_{33}H_{37}N_8O_9^+$ (M+H$^+$) 689.2678, found 689.2684.

Perchlorophenyl (E)-4-(3-(4-hydroxyphenyl)-2-methylacrylamido)benzoate (39)[8]

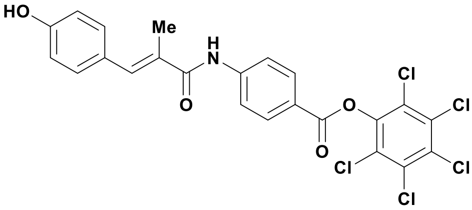

Chemical Formula: $C_{23}H_{14}Cl_5NO_4$
Exact Mass: 542,9365

Synthesized according to exp. procedure reported.

^{1}H NMR (500 MHz, DMF-d_7) δ 10.40 (s, 1H), 10.00 (s, 1H), 8.26 (d, *J* = 8.8 Hz, 2H), 8.16 (d, *J* = 8.9 Hz, 2H), 7.42 (d, *J* = 8.7 Hz, 3H), 6.95 (d, *J* = 8.4 Hz, 2H), 2.22 (s, 3H).
^{13}C NMR (126 MHz, DMF) δ 169.7, 162.5, 162.4, 158.6, 146.8, 145.1, 135.0, 131.9, 131.8, 131.3, 130.0, 128.2, 127.3, 121.0, 120.0, 115.8, 14.4.
HRMS (ESI) calculated for $C_{23}H_{15}Cl_5NO_4$ (M+H$^+$) 545.9409, found 545.9392.

(S,E)-5-(2-hydroxy-4-(4-(2-(4-(3-(4-hydroxyphenyl)-2-methylacrylamido)benzamido)-3-(1H-1,2,3-triazol-4-yl)propanamido)benzamido)-3-methoxybenzamido)picolinic acid (12)

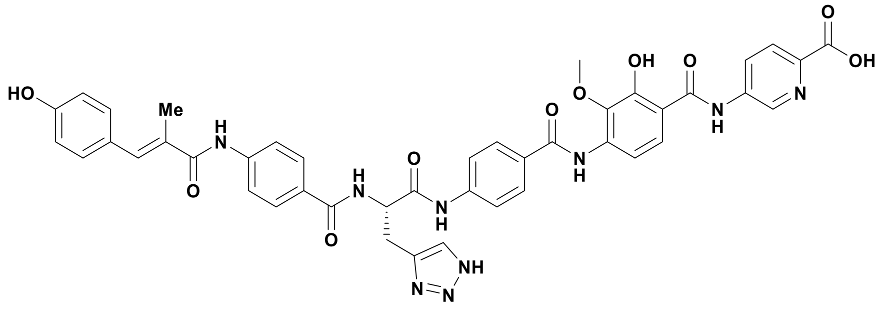

Chemical Formula: $C_{43}H_{37}N_9O_{10}$
Exact Mass: 839,2663

To a solution of tetrapeptide (**37a**, 200 mg, 275 µmol) in DMF (2 mL) was added perchlorophenyl (E)-4-(3-(4-hydroxyphenyl)-2-methylacrylamido) benzoate (**39**, 153 mg, 281 µmol) and NEt_3 (268 µL, 1.93 mmol). The reaction mixture was stirred for 1 d at 25°C and monitored by LC-MS (POM and methyl ester protected product: HRMS (ESI) calculated for $C_{50}H_{49}N_9O_{12}$ (M+H$^+$) 968.3573, found 968.3574). After full conversion KOH 3 N (1 mL) was added and stirred for 2.5 h. Next, the mixture was neutralized with HCl 6 N (0.5 mL) to a pH ~ 7. All volatiles were removed under reduced pressure, the residue was dissolved with DMSO (1 mL) and the salts were separated by centrifugation. The supernatant was loaded on the RP-HPLC system and purified under the following conditions. The collected fractions were lyophilized after their identity and purity was verified by LCMS to obtain 11 mg as a white solid (13 µmol, 5% over 2 steps).
RP-HPLC: 32-42% MeCN (+0.1% TFA) in milliQ H_2O (+0.1% TFA); 30 min; 50 mL/min.

^{1}H NMR (400 MHz, DMSO-d_6) δ 11.98 (s, 1H), 10.78 (s, 1H), 10.53 (s, 1H), 10.09 (s, 1H), 9.57 (s, 1H), 9.00 (d, J = 2.5 Hz, 1H), 8.71 (d, J = 7.3 Hz, 1H), 8.36 (dd, J = 8.7, 2.6 Hz, 1H), 8.10 (d, J = 8.6 Hz, 1H), 7.96 (d, J = 8.8 Hz, 2H), 7.87 (d, J = 8.9 Hz, 2H), 7.84 – 7.76 (m, 5H), 7.68 (d, J = 8.9 Hz, 2H), 7.35 (d, J = 8.6 Hz, 2H), 7.26 (s, 1H), 6.84 (d, J = 8.7 Hz, 2H), 3.86 (s, 3H), 2.11 (d, J = 1.3 Hz, 3H).
^{13}C NMR (126 MHz, DMSO) δ 142.2, 128.5, 125.7, 119.2, 129.2, 119.6, 128.7, 128.7, 123.9, 119.3, 113.1, 131.8, 134.4, 115.8, 54.7, 60.7, 27.6, 15.0.
HRMS (ESI) calculated for $C_{43}H_{38}N_9O_{10}$ (M+H$^+$) 840.2736, found: 840.2732, t_R = 7.48 min.

Methyl 6-(2-(benzyloxy)-3-methoxy-4-nitrobenzamido)nicotinate (24b-1)

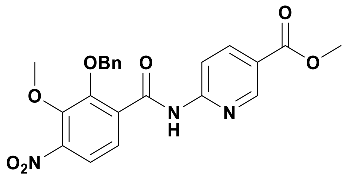

Chemical Formula: $C_{22}H_{19}N_3O_7$
Exact Mass: 437,1223

2-(Benzyloxy)-3-methoxy-4-nitrobenzoic acid (**23**, 2.19 g, 7.23 mmol) was dissolved in thionyl chloride (5.72 mL, 78.9 mmol) and refluxed for 2 h at 90°C. The thionyl chloride was removed under reduced pressure and the resulting acid chloride was dissolved in THF (10 mL). In parallel was prepared a mixture of methyl 6-aminonicotinate (**24b**, 1.00 g, 6.57 mmol) and triethylamine (1.83 mL, 13.1 mmol) in THF (10 mL). To the mixture was dropwise added the acid chloride solution at 0°C. The solution was allowed to warm to ambient temperature and stirred for 16 h. Diethylether (75 mL) was added to the reaction mixture and cooled to 0°C. The precipitate was collected by filtration and dissolved in dichloromethane (100 mL), water (100 mL) was added. The organic phase was washed with $NaHCO_3$ saturated solution (2 x 75 mL), HCl 1N (3 x 75 mL) and brine (75 mL), dried over sodium sulfate and evaporated under reduced pressure to give 2.28 g of a white solid (5.22 mmol, 79%) without further purification.

^{1}H NMR (500 MHz, DMSO-d_6) δ 11.26 (s, 1H), 8.88 (dd, *J* = 2.3, 0.8 Hz, 1H), 8.37 (dd, *J* = 8.7, 2.3 Hz, 1H), 8.29 (d, *J* = 8.8 Hz, 1H), 7.76 (d, *J* = 8.5 Hz, 1H), 7.54 (d, *J* = 8.4 Hz, 1H), 7.41 – 7.31 (m, 2H), 7.32 – 7.18 (m, 3H), 5.13 (s, 2H), 4.00 (s, 3H), 3.88 (s, 3H).
^{13}C NMR (126 MHz, DMSO) δ 164.8, 164.1, 154.6, 150.2, 149.5, 146.0, 145.9, 139.5, 135.8, 135.2, 128.5, 128.4, 128.3, 123.9, 121.5, 119.4, 113.1, 76.1, 62.2, 52.2.
HRMS (ESI) calculated for $C_{22}H_{20}N_3O_7$ (M+H$^+$) 438.1296, found 438.1290.

Methyl 6-(4-amino-2-(benzyloxy)-3-methoxybenzamido)nicotinate (25b)

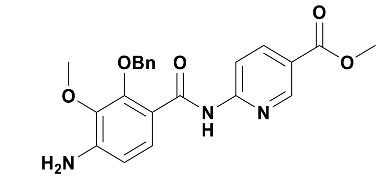

Chemical Formula: $C_{22}H_{21}N_3O_5$
Exact Mass: 407,1481

In a mixture of ethanol (250 mL) and acetic acid (50 mL) was dissolved methyl 6-(2-(benzyloxy)-3-methoxy-4-nitrobenzamido)nicotinate (**24b-1**, 2.24 g, 5.11 mmol). At 0°C was added Zn (6.69 g, 102 mmol) in portions. The mixture was stirred for 20 min and allowed to warm to 25°C. Next, the suspension was filtered through celite® and washed with EtOAc. The filtrate was evaporated under reduced pressure. To the residue were added EtOAc (200 mL) and a saturated solution of $NaHCO_3$ (200 mL), the organic phase was washed with $NaHCO_3$ (3 x 200 mL) and brine (200 mL), dried over sodium sulphate, evaporated under reduced pressure to give 2.01 g of an orange solid (4.93 mmol, 96%).

^{1}H NMR (400 MHz, DMSO-*d*$_6$) δ 10.68 (s, 1H), 8.79 (dt, *J* = 2.1, 1.0 Hz, 1H), 8.35 – 8.25 (m, 2H), 7.54 (d, *J* = 8.8 Hz, 1H), 7.52 – 7.45 (m, 2H), 7.39 – 7.30 (m, 3H), 6.59 (dd, *J* = 8.8, 0.7 Hz, 1H), 5.97 (s, 2H), 5.18 (s, 2H), 3.87 (s, 3H), 3.78 (s, 3H).
^{13}C NMR (101 MHz, DMSO) δ 164.9, 163.2, 154.9, 150.9, 149.6, 147.9, 139.3, 137.8, 135.6, 129.3, 128.6, 128.4, 127.0, 120.6, 112.5, 112.3, 110.3, 76.2, 59.8, 52.1.
HRMS (ESI) calculated for $C_{22}H_{22}N_3O_5$ (M+H$^+$) 408.1554, found 408.1548.

Methyl 6-(2-(benzyloxy)-3-methoxy-4-(4-nitrobenzamido)benzamido)nicotinate (25b-1)

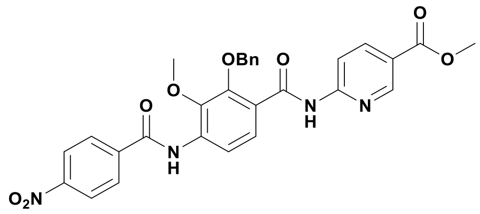

Chemical Formula: $C_{29}H_{24}N_4O_8$
Exact Mass: 556,1594

To a mixture of methyl 6-(4-amino-2-(benzyloxy)-3-methoxybenzamido)nicotinate (**25b**, 2.10 g, 4.94 mmol) and triethylamine (1.37 mL, 9.88 mmol) was added dropwise a solution of 4-nitrobenzoyl chloride (**27c**, 1.37 g, 7.41 mmol) in THF (10 mL) at 0°C. The reaction mixture was allowed to warm to ambient temperature and stirred for 12 h. Diethylether (100 mL) was added to the reaction mixture and cooled to 0°C. The precipitate was collected by filtration and dissolved in dichloromethane (100 mL), the organic phase was washed with a saturated solution of $NaHCO_3$ (3 x 100 mL), HCl 1N (1 x 100 mL) and brine (1 x 100 mL), dried over sodium sulfate and concentrated under reduced pressure to give 2.22 g of a yellow solid (3.99 mmol, 81%) without further purification.

^{1}H NMR (500 MHz, DMSO-*d*$_6$) δ 10.93 (s, 1H), 10.24 (s, 1H), 8.86 (dd, *J* = 2.3, 0.9 Hz, 1H), 8.42 – 8.29 (m, 4H), 8.21 (d, *J* = 8.9 Hz, 2H), 7.82 (d, *J* = 8.6 Hz, 1H), 7.60 (d, *J* = 8.6 Hz, 1H), 7.47 – 7.38 (m, 2H), 7.34 – 7.24 (m, 3H), 5.17 (s, 2H), 3.94 (s, 3H), 3.89 (s, 3H).
^{13}C NMR (126 MHz, DMSO) δ 164.8, 164.4, 163.9, 154.7, 149.8, 149.5, 149.3, 145.4, 140.0, 139.4, 135.8, 135.3, 129.4, 128.9, 128.5, 128.3, 125.4, 124.6, 123.6, 121.2, 119.6, 112.9, 76.1, 61.0, 52.2.
HRMS (ESI) calculated for $C_{29}H_{25}N_4O_8$ (M+H$^+$) 557.1667, found 557.1658.

Methyl 6-(4-(4-aminobenzamido)-2-(benzyloxy)-3-methoxybenzamido)nicotinate (36b)

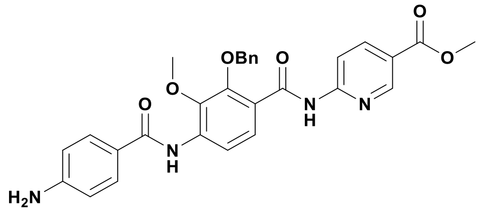

Chemical Formula: $C_{29}H_{26}N_4O_6$
Exact Mass: 526,1852

In a mixture of ethanol (250 mL) and acetic acid (50 mL) was dissolved methyl 6-(2-(benzyloxy)-3-methoxy-4-(4-nitrobenzamido)benzamido)nicotinate (**25b-1**, 2.19 g, 5.01 mmol). At 0°C was added Zn (6.55 g, 100 mmol) in portions. The mixture was stirred for 20 min and allowed to warm to 25°C. Next, the suspension was filtered through celite® and washed with EtOAc. The filtrate was evaporated under reduced pressure. To the residue were added EtOAc (200 mL) and $NaHCO_3$ saturated solution (200 mL), the organic phase was washed with $NaHCO_3$ (3 x 200 mL) and brine (200 mL), dried over sodium sulphate, evaporated under reduced pressure to give 1.36 g of yellow solid (3.34 mmol, 67%).

^{1}H NMR (500 MHz, DMSO-d_6) δ 10.85 (s, 1H), 9.21 (s, 1H), 8.85 (dd, *J* = 2.2, 1.0 Hz, 1H), 8.39 – 8.29 (m, 2H), 8.00 (d, *J* = 8.7 Hz, 1H), 7.76 – 7.68 (m, 2H), 7.61 (d, *J* = 8.7 Hz, 1H), 7.48 – 7.40 (m, 2H), 7.35 – 7.25 (m, 3H), 6.67 – 6.60 (m, 2H), 5.88 (s, 2H), 5.18 (s, 2H), 3.95 (s, 3H), 3.88 (s, 3H).
^{13}C NMR (126 MHz, DMSO) δ 165.0, 164.8, 163.7, 154.7, 152.7, 149.6, 149.5, 143.6, 139.4, 136.8, 135.7, 129.4, 129.0, 128.5, 128.3, 125.0, 123.0, 121.2, 120.1, 117.6, 112.9, 112.7, 76.1, 60.9, 52.1.
HRMS (ESI) calculated for $C_{29}H_{27}N_4O_6$ (M+H$^+$) 527.1925, found 527.1921.

(S)-2-((tert-butoxycarbonyl)amino)-3-(1-((pivaloyloxy)methyl)-1H-1,2,3-triazol-4-yl)propanoic acid (38)[8]

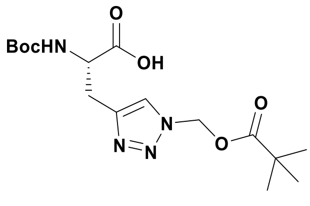

Chemical Formula: $C_{16}H_{26}N_4O_6$
Exact Mass: 370,1852

Synthesized according to exp. procedure reported.

^{1}H NMR (500 MHz, DMSO-d_6) δ 12.57 (s, 1H), 7.94 (s, 1H), 7.08 (d, *J* = 8.3 Hz, 1H), 6.28 (s, 2H), 4.16 (td, *J* = 9.2, 4.7 Hz, 1H), 3.09 (dd, *J* = 14.8, 4.7 Hz, 1H), 2.94 (dd, *J* = 14.8, 9.8 Hz, 1H), 1.34 (s, 9H), 1.11 (s, 9H).
^{13}C NMR (126 MHz, DMSO) δ 176.5, 173.1, 155.4, 143.8, 124.1, 78.2, 69.9, 53.4, 38.2, 28.2, 28.2, 26.5.
HRMS (ESI) calculated for $C_{16}H_{27}N_4O_6$ (M+H$^+$) 371.1917, found 371.1925.

Methyl (S)-6-(2-(benzyloxy)-4-(4-(2-((tert-butoxycarbonyl)amino)-3-(1-((pivaloyloxy)methyl)-1H-1,2,3-triazol-4-yl)propanamido)benzamido)-3-methoxybenzamido)nicotinate (36b-1)

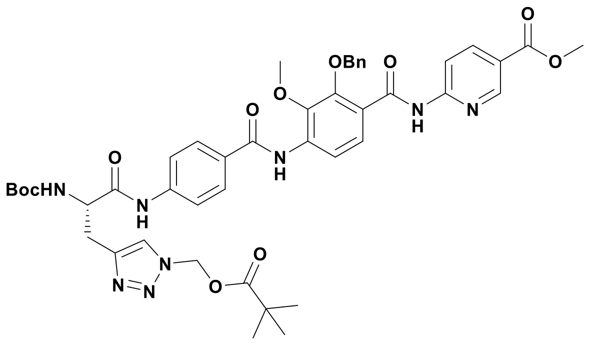

Chemical Formula: $C_{45}H_{50}N_8O_{11}$
Exact Mass: 878,3599

To a solution of EEDQ (845 mg, 3.42 mmol) in THF (10 mL) was added (S)-2-((tert-butoxycarbonyl)amino)-3-(1-((pivaloyloxy)methyl)-1H-1,2,3-triazol-4-yl)propanoic acid (**38**, 844 mg, 2.28 mmol) at 0°C and stirred for 15 min. Next a solution of methyl 6-(4-(4-aminobenzamido)-2-(benzyloxy)-3-methoxybenzamido)nicotinate (**36b**, 600 mg, 1.14 mmol) in THF (5 mL) was added at 0°C, the reaction mixture was allowed to warm to ambient temperature and stirred for 18 h. All volatiles were removed under reduced pressure and the residue was dissolved in dichloromethane, the organic phase was washed with HCl 1 N (50 mL), a saturated solution of $NaHCO_3$ (50 mL) and brine (50 mL), dried over sodium sulfate and evaporated. The residue was purified by column chromatography on silica gel (eluent: 0.3→1% MeOH in dichloromethane) to give 757 mg of a colorless solid (861 µmol, 76%).

^{1}H NMR (500 MHz, DMSO-d_6) δ 10.90 (s, 1H), 10.41 (s, 1H), 9.66 (s, 1H), 8.85 (dd, *J* = 2.2, 1.0 Hz, 1H), 8.38 – 8.29 (m, 2H), 8.01 – 7.95 (m, 3H), 7.90 (d, *J* = 8.7 Hz, 1H), 7.80 – 7.74 (m, 2H), 7.61 (d, *J* = 8.6 Hz, 1H), 7.47 – 7.40 (m, 2H), 7.34 – 7.26 (m, 3H), 7.20 (d, *J* = 8.0 Hz, 1H), 6.29 (s, 2H), 5.18 (s, 2H), 4.41 (q, *J* = 8.3 Hz, 1H), 3.95 (s, 3H), 3.88 (s, 3H), 3.13 (dd, *J* = 14.7, 5.1 Hz, 1H), 3.01 (dd, *J* = 14.7, 9.4 Hz, 1H), 1.37 (s, 9H), 1.10 (s, 9H).
^{13}C NMR (126 MHz, DMSO) δ 176.4, 170.7, 164.8, 163.8, 154.7, 149.7, 149.5, 144.6, 142.2, 139.4, 136.1, 135.7, 129.0, 128.7, 128.5, 128.3, 124.7, 124.3, 121.2, 118.7, 112.9, 78.3, 76.1, 69.8, 60.9, 54.9, 52.2, 38.2, 28.1, 26.4.
HRMS (ESI) calculated for $C_{45}H_{51}N_8O_{11}$ (M+H$^+$) 879.3672, found 879.3669.

Methyl (S)-6-(4-(4-(2-((tert-butoxycarbonyl)amino)-3-(1-((pivaloyloxy)methyl)-1H-1,2,3-triazol-4-yl)propanamido)benzamido)-2-hydroxy-3-methoxybenzamido)nicotinate (36b-2)

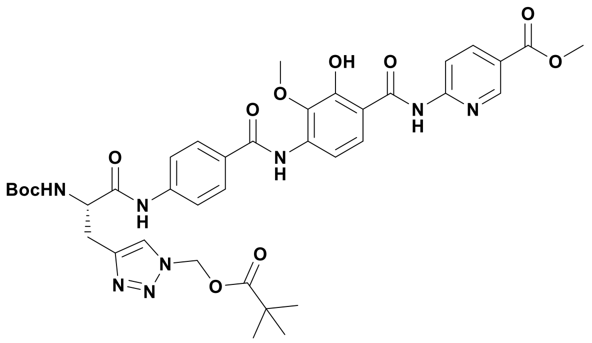

Chemical Formula: $C_{38}H_{44}N_8O_{11}$
Exact Mass: 788,3130

A mixture of Methyl (S)-6-(2-(benzyloxy)-4-(4-(2-((tert-butoxycarbonyl)amino)-3-(1-((pivaloyloxy)methyl)-1H-1,2,3-triazol-4-yl)propanamido)benzamido)-3-methoxybenzamido) nicotinate (**36b-1**, 684 mg, 778 µmol) and 10% Pd/C (68 mg), in 1:1:1 mixture of EtOAc/MeOH/THF (60 mL), was stirred for 1 h under H_2 atmosphere. After the catalyst was filtered and washed with MeOH, the filtrate was evaporated to give 577 mg of a colorless solid (731 µmol, 94%).

^{1}H NMR (400 MHz, DMSO-d_6) δ 11.73 (s, 1H), 11.25 (s, 1H), 10.43 (s, 1H), 9.62 (s, 1H), 8.90 (t, *J* = 1.6 Hz, 1H), 8.36 (d, *J* = 1.6 Hz, 2H), 8.00 – 7.90 (m, 3H), 7.87 (d, *J* = 8.9 Hz, 1H), 7.80 – 7.71 (m, 2H), 7.62 (d, *J* = 8.9 Hz, 1H), 7.29 – 7.13 (m, 1H), 6.29 (s, 2H), 4.40 (q, *J* = 8.3 Hz, 1H), 3.88 (s, 3H), 3.80 (s, 3H), 3.12 (dd, *J* = 14.7, 5.1 Hz, 1H), 3.00 (dd, *J* = 14.6, 9.4 Hz, 1H), 1.35 (d, *J* = 3.1 Hz, 9H), 1.09 (s, 9H).
^{13}C NMR (126 MHz, DMSO) δ 176.4, 170.7, 164.8, 163.8, 154.7, 149.7, 149.5, 144.6, 142.2, 139.4, 136.1, 135.7, 129.0, 128.7, 128.5, 128.3, 124.7, 124.3, 121.2, 118.7, 112.9, 78.3, 76.1, 69.8, 60.9, 54.9, 52.2, 38.2, 28.1, 26.4.
HRMS (ESI) calculated for $C_{38}H_{45}N_8O_{11}$ (M+H$^+$) 789.3202, found 789.3208.

Methyl (S)-6-(4-(4-(2-amino-3-(1-((pivaloyloxy)methyl)-1H-1,2,3-triazol-4-yl)propanamido)benzamido)-2-hydroxy-3-methoxybenzamido)nicotinate (37b)

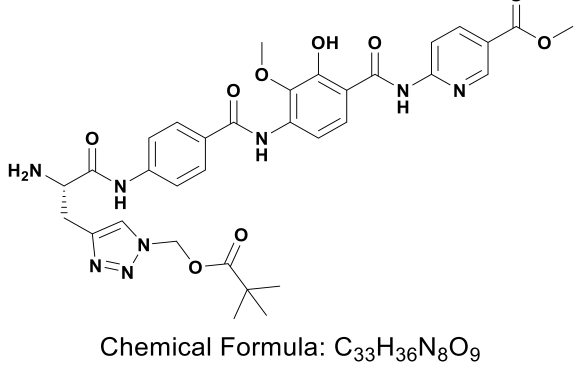

Chemical Formula: $C_{33}H_{36}N_8O_9$
Exact Mass: 688,2605

Methyl (S)-6-(4-(4-(2-((tert-butoxycarbonyl)amino)-3-(1-((pivaloyloxy)methyl)-1H-1,2,3-triazol-4-yl)propanamido)benzamido)-2-hydroxy-3-methoxybenzamido)nicotinate (**36b-2,** 548 mg, 695 µmol) was dissolved in HCl 4N in dioxane (10 mL) and stirred for 1 h at 25°C. All volatiles were removed under reduced pressure, the residue was dissolved in 2:1 mixture of water/MeCN and lyophilized to give 462 mg of a white fluffy solid (637 µmol, 92%).

^{1}H NMR (400 MHz, DMSO-d_6) δ 11.33 (s, 1H), 11.27 (s, 1H), 9.68 (s, 1H), 8.89 (t, *J* = 1.6 Hz, 1H), 8.63 (d, *J* = 5.4 Hz, 3H), 8.36 (d, *J* = 1.6 Hz, 2H), 8.16 (s, 1H), 8.01 – 7.92 (m, 2H), 7.86 (d, *J* = 8.9 Hz, 1H), 7.80 – 7.72 (m, 2H), 7.59 (d, *J* = 8.9 Hz, 1H), 6.27 (s, 2H), 4.46 – 4.38 (m, 1H), 3.87 (s, 3H), 3.79 (s, 3H), 3.36 (d, *J* = 6.6 Hz, 2H), 1.07 (s, 10H).
^{13}C NMR (101 MHz, DMSO) δ 176.4, 166.7, 165.1, 164.8, 154.6, 151.0, 149.5, 141.4, 140z.8, 139.8, 139.6, 136.4, 129.2, 128.8, 125.1, 125.0, 121.3, 118.9, 114.7, 114.2, 113.5, 69.8, 66.3, 60.4, 52.6, 52.2, 26.4.
^{13}C NMR (101 MHz, DMSO) δ 176.4, 166.8, 165.1, 164.8, 154.7, 151.1, 149.6, 141.5, 140.9, 139.8, 139.6, 136.4, 129.3, 128.8, 125.1, 125.0, 121.4, 119.0, 114.8, 114.3, 113.5, 69.9, 66.4, 60.5, 52.7, 52.2, 38.2, 26.5.
HRMS (ESI) calculated for $C_{33}H_{37}N_8O_9$ ($M+H^+$) 689.2678, found 689.2680.

(S,*E*)-6-(2-hydroxy-4-(4-(2-(4-(3-(4-hydroxyphenyl)-2-methylacrylamido)benzamido)-3-(1H-1,2,3-triazol-4-yl)propanamido)benzamido)-3-methoxybenzamido)nicotinic acid (13)

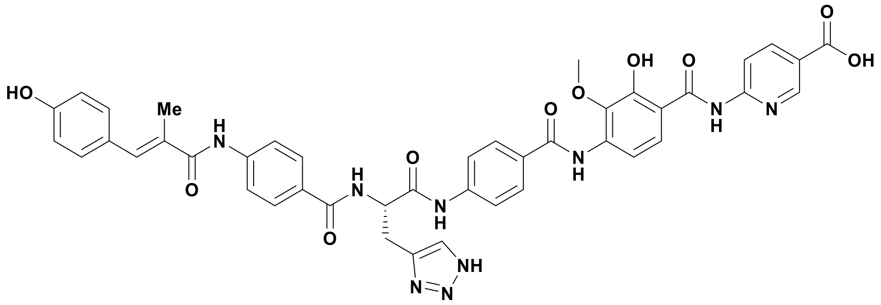

Chemical Formula: $C_{43}H_{37}N_9O_{10}$
Exact Mass: 839,2663

To a solution of tetrapeptide **37b** (246 mg, 357 µmol) in DMF (2 mL) was added perchlorophenyl (E)-4-(3-(4-hydroxyphenyl)-2-methylacrylamido)benzoate (**39**, 194 mg, 357 µmol) and NEt_3 (0.111 mL, 0.795 mmol). The reaction mixture was stirred for 1 d at 25°C and monitored by LC-MS (POM protected product: HRMS (ESI) calculated for $C_{50}H_{49}N_9O_{12}$ (M+H$^+$) 968.3573, found 968.3582). After full conversion KOH 3 N (1 mL) was added and stirred for 20 min. Next, the mixture was neutralized with HCl 6 N (0.5 mL) to a pH ~ 7. All volatiles were removed under reduced pressure, the residue was dissolved with DMSO (1 mL) and the salts were separated by centrifugation. The supernatant was loaded on the RP-HPLC system and purified under the following conditions. The collected fractions were lyophilized after their identity and purity was verified by LCMS to obtain 23.6 mg as a white solid (28 µmol, 8% over 2 steps).
RP-HPLC: 38-55% MeCN (+0.1% TFA) in milliQ H_2O (+0.1% TFA); 30 min; 50 mL/min.

^{1}H NMR (400 MHz, DMSO-*d*$_6$) δ 11.74 (s, 1H), 11.23 (s, 1H), 10.53 (s, 1H), 10.09 (s, 1H), 9.64 (s, 1H), 8.88 (t, *J* = 1.6 Hz, 1H), 8.71 (d, *J* = 7.6 Hz, 1H), 8.34 (d, *J* = 1.5 Hz, 2H), 7.96 (d, *J* = 8.8 Hz, 2H), 7.91 – 7.75 (m, 7H), 7.69 (s, 1H), 7.62 (d, *J* = 9.0 Hz, 1H), 7.38 – 7.32 (m, 2H), 7.28 – 7.24 (m, 1H), 6.87 – 6.80 (m, 2H), 4.95 – 4.88 (m, 1H), 3.80 (s, 3H), 3.32 – 3.22 (m, 2H), 2.11 (d, *J* = 1.4 Hz, 3H).
^{13}C NMR (126 MHz, DMSO) δ 150.2, 113.8, 140.1, 119.1, 129.3, 119.5, 128.6, 119.5, 128.6, 119.4, 114.4, 131.8, 134.4, 115.9, 54.7, 60.9, 27.6, 15.0.
HRMS (ESI) calculated for $C_{43}H_{38}N_9O_{10}$ (M+H$^+$) 840.2736, found 840.2733, t_R= 7.27 min.

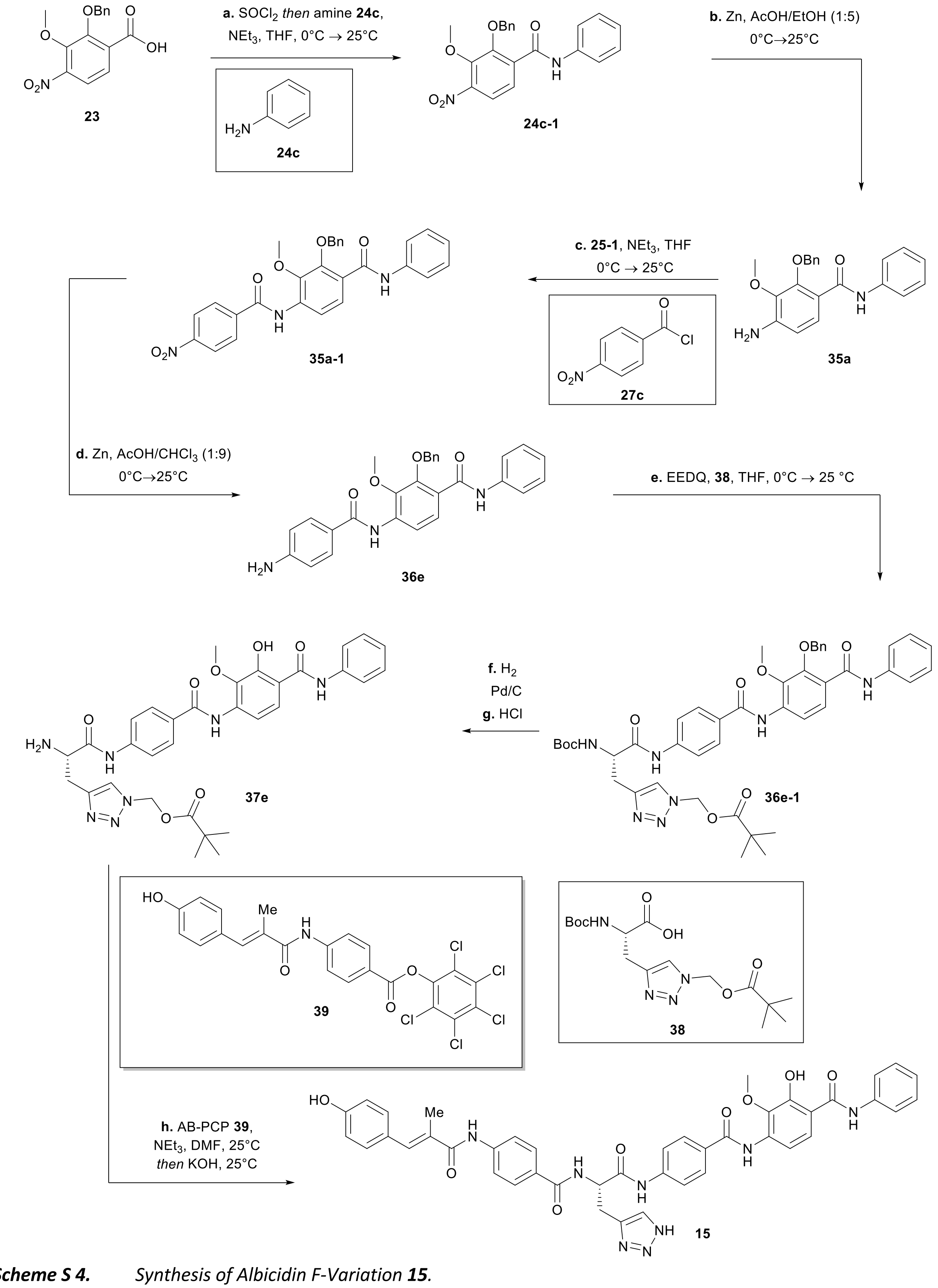

Scheme S 4. *Synthesis of Albicidin F-Variation* ***15.***

2-(Benzyloxy)-3-methoxy-4-nitro-N-phenylbenzamide (24c-1)

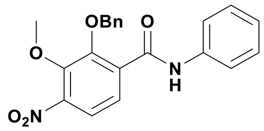

Chemical Formula: $C_{21}H_{18}N_2O_5$
Exact Mass: 378.1216

2-(Benzyloxy)-3-methoxy-4-nitrobenzoic acid (**23**, 1.97 g, 6.50 mmol) was dissolved in thionyl chloride (11.3 mL, 156 mmol) and refluxed for 2 h at 90°C. The thionyl chloride was removed under reduced pressure and the resulting acid chloride was dissolved in THF (10 mL). In parallel was prepared a mixture of aniline (**24c**, 1.78 mL, 19.5 mmol) and triethylamine (1.81 mL, 13 mmol) in THF (10 mL). To the mixture was added dropwise the acid chloride solution at 0°C. The solution was allowed to warm to ambient temperature and stirred for 16 h. Diethylether (75 mL) was added to the reaction mixture and cooled to 0°C. The precipitate was collected by filtration and dissolved in dichloromethane (100 mL). Water (100 mL) was then added. The organic phase was washed with a saturated solution of $NaHCO_3$ (2 x 75 mL), HCl 1 N (3 x 75 mL) and brine (75 mL), dried over sodium sulfate and evaporated under reduced pressure. The crude product was purified by recrystallisation (CH_2Cl_2/cyclohexane) to give 1.67 g of a light-yellow solid (4.41 mmol, 68%).

^{1}H NMR (400 MHz, DMSO-d_6) δ 10.48 (s, 1H), 7.77 (d, *J* = 8.5 Hz, 1H), 7.74 – 7.61 (m, 2H), 7.49 (d, *J* = 8.4 Hz, 1H), 7.44 – 7.24 (m, 7H), 7.17 – 7.08 (m, 1H), 5.12 (s, 2H), 3.99 (s, 3H).
^{13}C NMR (101 MHz, DMSO) δ 163.2, 149.9, 146.1, 145.4, 138.6, 136.8, 136.2, 128.8, 128.4, 128.4, 124.0, 123.7, 119.7, 119.5, 76.0, 62.3.
HRMS (ESI) calculated for $C_{21}H_{19}N_2O_5$ (M+H$^+$) 379.1288, found 379.1285.

4-Amino-2-(benzyloxy)-3-methoxy-N-phenylbenzamide (35a)

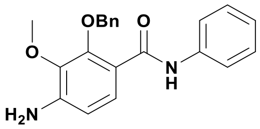

Chemical Formula: $C_{21}H_{20}N_2O_3$
Exact Mass: 348.1474

To 2-(benzyloxy)-3-methoxy-4-nitro-N-phenylbenzamide (**24c-1**, 1.16 g, 3.07 mmol) in a mixture of chloroform (90 mL) and acetic acid (10 mL) at 0°C was added zinc dust (11.6 g, 177 mmol) portion wise. The ice bath was removed after 10 min and the reaction mixture was stirred for further 30 min. The reaction was monitored by TLC, after full conversion the suspension was filtered over celite® and washed with dichloromethane (100 mL). The filtrate was washed with a saturated solution of $NaHCO_3$ (3 x 100 mL) and brine (100 mL), dried over sodium sulfate and evaporated under reduced pressure to give 1.06 g of a brownish oil (3.07 mmol, 99%) which was used without further purification.

TLC (2% MeOH in CH_2Cl_2) R_f = 0.40;
^{1}H NMR (400 MHz, DMSO-d_6) δ 9.89 (s, 1H), 7.49 (ddd, *J* = 5.0, 3.9, 2.1 Hz, 2H), 7.45 – 7.28 (m, 7H), 7.26 – 7.17 (m, 2H), 7.04 – 6.95 (m, 1H), 6.56 (d, *J* = 8.6 Hz, 1H), 5.72 (s, 2H), 5.17 (s, 2H), 3.80 (s, 3H);
^{13}C NMR (101 MHz, DMSO) δ 163.4, 150.7, 146.5, 139.0, 137.9, 136.6, 128.7, 128.6, 128.6, 128.5, 126.3, 123.0, 119.2, 114.5, 109.9, 75.6, 59.7;
HRMS (ESI) calculated for $C_{21}H_{21}N_2O_3$ (M+H$^+$) 349.1547, found 349.1539.

2-(Benzyloxy)-3-methoxy-4-(4-nitrobenzamido)-N-phenylbenzamide (35a-1)

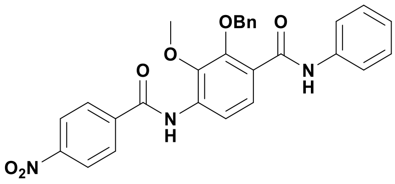

Chemical Formula: $C_{28}H_{23}N_3O_6$
Exact Mass: 497.1587

To a mixture of 4-amino-2-(benzyloxy)-3-methoxy-N-phenylbenzamide (**35a**, 1.04 g, 2.97 mmol) and triethylamine (826 µL, 5.94 mmol) was added dropwise a solution of 4-nitrobenzoyl chloride (**27c**, 0.827 g, 4.46 mmol) in THF (10 mL) at 0°C. The reaction mixture was allowed to warm to ambient temperature and stirred for 12 h. Diethylether (100 mL) was added to the reaction mixture and cooled to 0°C. The precipitate was collected by filtration and dissolved in dichloromethane (100 mL), the organic phase was washed with $NaHCO_3$ saturated solution (3 x 100 mL), HCl 1N (1 x 100 mL) and brine (1 x 100 mL), dried over sodium sulfate and concentrated under reduced pressure to give 1.29 g of a light-yellow solid (2.60 mmol, 87%) which was used without further purification.

^1H NMR (400 MHz, DMSO-d_6) δ 10.28 (d, *J* = 2.9 Hz, 2H), 8.39 (d, *J* = 8.8 Hz, 2H), 8.23 (d, *J* = 8.8 Hz, 2H), 7.70 (d, *J* = 8.5 Hz, 1H), 7.67 – 7.58 (m, 2H), 7.49 – 7.40 (m, 3H), 7.38 – 7.27 (m, 5H), 7.09 (ddt, *J* = 8.6, 7.3, 1.2 Hz, 1H), 5.13 (s, 2H), 3.92 (s, 3H);
^{13}C NMR (101 MHz, DMSO) δ 164.4, 164.0, 149.4, 149.3, 146.0, 140.0, 138.9, 136.6, 133.9, 130.7, 129.4, 128.7, 128.5, 128.4, 128.4, 128.3, 123.8, 123.6, 120.2, 119.6, 75.7, 60.9;
HRMS (ESI) calculated for $C_{28}H_{24}N_3O_6$ ($M+H^+$) 498.1660, found 498.1647.

4-(4-Aminobenzamido)-2-(benzyloxy)-3-methoxy-N-phenylbenzamide (36e)

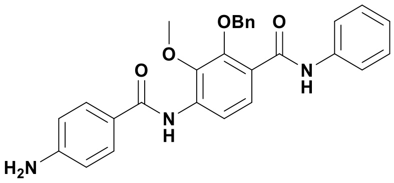

Chemical Formula: $C_{28}H_{25}N_3O_4$
Exact Mass: 467.1845

To 2-(benzyloxy)-3-methoxy-4-(4-nitrobenzamido)-N-phenylbenzamide (**35a-1**, 1.29 g, 2.60 mmol) in a mixture of chloroform (90 mL) and acetic acid (10 mL) at 0°C was added zinc dust (12.0 g, 183 mmol) portion wise. The ice bath was removed after 10 min and the reaction mixture was stirred for further 30 min. The reaction was monitored by TLC, after full conversion the suspension was filtered over celite® and washed with dichloromethane (100 mL). The filtrate was washed with a saturated solution of $NaHCO_3$ (3 x 100 mL) and brine (100 mL), dried over sodium sulfate and evaporated under reduced pressure to give 1.02 g of a grey solid (2.49 mmol, 87%) which was used without further purification.

^1H NMR (400 MHz, DMSO-d_6) δ 10.21 (s, 1H), 9.22 (s, 1H), 7.88 (d, *J* = 8.6 Hz, 1H), 7.77 – 7.68 (m, 2H), 7.62 – 7.54 (m, 2H), 7.50 – 7.39 (m, 3H), 7.39 – 7.26 (m, 5H), 7.12 – 7.03 (m, 1H), 6.67 – 6.59 (m, 2H), 5.87 (s, 2H), 5.13 (s, 2H), 3.93 (s, 3H);
^{13}C NMR (101 MHz, DMSO) δ 165.0, 163.9, 152.6, 149.2, 144.2, 138.9, 136.6, 135.3, 129.4, 128.7, 128.4, 128.3, 126.2, 123.9, 123.5, 120.2, 119.6, 118.1, 112.7, 75.6, 60.9;
HRMS (ESI) calculated for $C_{28}H_{26}N_3O_4$ ($M+H^+$) 468.1918, found 468.1904.

(S)-(4-(3-((4-((3-(benzyloxy)-2-methoxy-4-(phenylcarbamoyl)phenyl)carbamoyl)phenyl)amino)-2-((tert-butoxycarbonyl)amino)-3-oxopropyl)-1H-1,2,3-triazol-1-yl)methyl pivalate (36e-1)

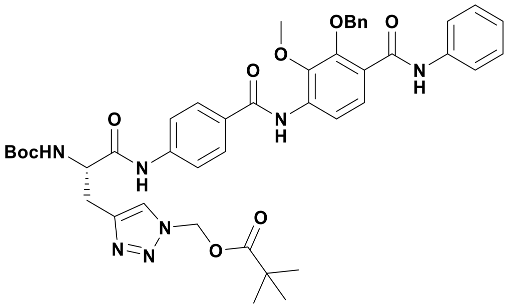

Chemical Formula: $C_{44}H_{49}N_7O_9$
Exact Mass: 819.3592

To a solution of EEDQ (1.52 g, 6.15 mmol) in THF (10 mL) was added (S)-2-((tert-butoxycarbonyl)amino)-3-(1-((pivaloyloxy)methyl)-1H-1,2,3-triazol-4-yl)propanoic acid (**38**, 1.52 g, 4.10 mmol) at 0°C and stirred for 15 min. Next a solution of 4-(4-aminobenzamido)-2-(benzyloxy)-3-methoxy-N-phenylbenzamide (**36e**, 0.760 g, 1.63 mmol) in THF (5 mL) was added at 0°C, the reaction mixture was allowed to warm to ambient temperature and stirred for 18 h. All volatiles were removed under reduced pressure and the residue was dissolved in dichloromethane, the organic phase was washed with HCl 1 N (50 mL), a saturated solution of $NaHCO_3$ (50 mL) and brine (50 mL), dried over sodium sulfate and evaporated. The residue was purified by column chromatography on silica gel (eluent: 0.3→3% MeOH in CH_2Cl_2) to give 826 mg of a colorless solid (1.01 mmol, 62%).

^{1}H NMR (400 MHz, DMSO-d_6) δ 10.43 (s, 1H), 10.25 (s, 1H), 9.67 (s, 1H), 8.06 – 7.95 (m, 3H), 7.78 (dd, *J* = 8.6, 5.0 Hz, 3H), 7.64 – 7.57 (m, 2H), 7.50 – 7.40 (m, 3H), 7.37 – 7.27 (m, 5H), 7.23 (d, *J* = 8.0 Hz, 1H), 7.12 – 7.04 (m, 1H), 6.29 (s, 2H), 5.76 (s, 1H), 5.13 (s, 2H), 4.44 – 4.36 (m, 1H), 3.92 (s, 3H), 3.13 (dd, *J* = 14.7, 5.1 Hz, 1H), 3.00 (dd, *J* = 14.6, 9.5 Hz, 1H), 1.36 (s, 9H), 1.09 (s, 9H);
^{13}C NMR (101 MHz, DMSO) δ 176.5, 170.8, 164.9, 164.0, 155.4, 149.3, 145.2, 143.5, 142.2, 138.9, 136.6, 134.6, 128.7, 128.6, 128.4, 128.3, 127.5, 124.2, 123.7, 123.6, 119.6, 119.3, 118.7, 78.3, 75.7, 69.9, 60.9, 54.9, 28.2, 27.8, 26.5;
HRMS (ESI) calculated for $C_{44}H_{50}N_7O_9$ (M+H$^+$) 820.3665, found 820.3664.

(S)-(4-(2-((tert-butoxycarbonyl)amino)-3-((4-((3-hydroxy-2-methoxy-4-(phenylcarbamoyl)phenyl)carbamoyl)phenyl)amino)-3-oxopropyl)-1H-1,2,3-triazol-1-yl)methyl pivalate (36e-2)

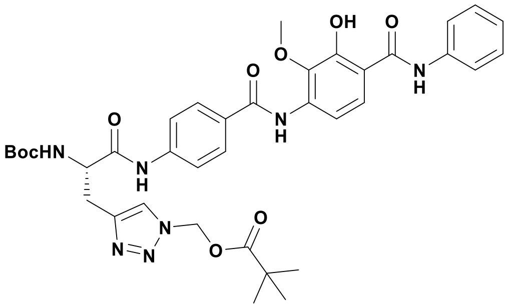

Chemical Formula: $C_{37}H_{43}N_7O_9$
Exact Mass: 729.3122

A mixture of Methyl (S)-(4-(3-((4-((3-(benzyloxy)-2-methoxy-4-(phenylcarbamoyl)phenyl)carbamoyl)phenyl)amino)-2-((tert-butoxycarbonyl)amino)-3-oxopropyl)-1H-1,2,3-triazol-1-yl)methyl pivalate (**36e-1**, 642 mg, 783 µmol) and 10% Pd/C (64 mg), in 1:1:1

mixture of EtOAc/MeOH/THF (60 mL), was stirred for 1 h under H_2 atmosphere. After the catalyst was filtered and washed with MeOH, the filtrate was evaporated to give 505 mg of a colorless solid (692 µmol, 88%).

^{1}H NMR (400 MHz, DMSO-d_6) δ 12.56 (s, 1H), 10.42 (d, *J* = 15.6 Hz, 2H), 9.50 (s, 1H), 8.00 – 7.92 (m, 3H), 7.85 (d, *J* = 9.1 Hz, 1H), 7.76 (d, *J* = 8.7 Hz, 2H), 7.73 – 7.66 (m, 2H), 7.65 (d, *J* = 8.9 Hz, 1H), 7.40 (t, *J* = 7.9 Hz, 2H), 7.26 – 7.13 (m, 2H), 6.29 (s, 2H), 4.40 (q, *J* = 7.9, 7.4 Hz, 1H), 3.87 (s, 3H), 3.12 (dd, *J* = 14.6, 5.1 Hz, 1H), 3.00 (dd, *J* = 14.7, 9.4 Hz, 1H), 1.36 (s, 9H), 1.09 (s, 9H).
^{13}C NMR (101 MHz, DMSO) δ 176.5, 170.8, 168.1, 164.8, 155.4, 153.9, 143.5, 142.3, 138.8, 137.7, 136.0, 128.7, 128.7, 124.2, 122.8, 121.7, 118.7, 112.6, 112.3, 78.3, 69.9, 60.2, 59.8, 54.9, 48.6, 38.2, 28.2, 26.5, 25.1.
HRMS (ESI) calculated for $C_{37}H_{44}N_7O_9$ (M+H$^+$) 730.3195, found 730.3197.

(S)-(4-(2-amino-3-((4-((3-hydroxy-2-methoxy-4-(phenylcarbamoyl)phenyl)carbamoyl)phenyl)amino)-3-oxopropyl)-1H-1,2,3-triazol-1-yl)methyl pivalate (37e)

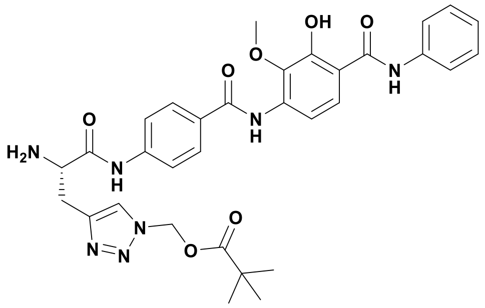

Chemical Formula: $C_{32}H_{35}N_7O_7$
Exact Mass: 629.2598

(S)-(4-(2-((tert-butoxycarbonyl)amino)-3-((4-((3-hydroxy-2-methoxy-4-(phenylcarbamoyl)phenyl)carbamoyl)phenyl)amino)-3-oxopropyl)-1H-1,2,3-triazol-1-yl)methyl pivalate (**36e-2**, 489 mg, 670 µmol) was dissolved in HCl 4 N in dioxane (10 mL) and stirred for 1 h at 25°C. All volatiles were removed under reduced pressure. The residue was dissolved in 2:1 mixture of water/MeCN and lyophilized to give 414 mg of a white fluffy solid (621 µmol, 93%).

^{1}H NMR (500 MHz, DMSO-d_6) δ 12.58 (s, 1H), 11.23 (s, 1H), 10.46 (s, 1H), 9.52 (s, 1H), 8.61 – 8.57 (m, 3H), 8.15 (s, 1H), 8.00 – 7.94 (m, 2H), 7.90 (d, *J* = 9.0 Hz, 1H), 7.79 – 7.73 (m, 2H), 7.74 – 7.68 (m, 2H), 7.63 (d, *J* = 8.8 Hz, 1H), 7.43 – 7.35 (m, 2H), 7.21 – 7.14 (m, 1H), 6.28 (s, 2H), 4.42 (q, *J* = 6.1 Hz, 1H), 3.87 (s, 3H), 3.36 (d, *J* = 6.6 Hz, 2H), 1.07 (s, 9H).
^{13}C NMR (126 MHz, DMSO) δ 176.4, 168.1, 166.8, 164.7, 154.0, 141.4, 140.9, 138.9, 137.7, 135.9, 129.3, 128.7, 128.7, 125.0, 124.6, 122.9, 121.7, 119.0, 112.7, 112.3, 69.9, 60.2, 52.7, 38.1, 27.1, 26.4.
HRMS (ESI) calculated for $C_{32}H_{36}N_7O_7$ (M+H$^+$) 630.2671, found 630.2667.

(S,E)-2-hydroxy-4-(4-(2-(4-(3-(4-hydroxyphenyl)-2-methylacrylamido)benzamido)-3-(1H-1,2,3-triazol-4-yl)propanamido)benzamido)-3-methoxy-N-phenylbenzamide (15)

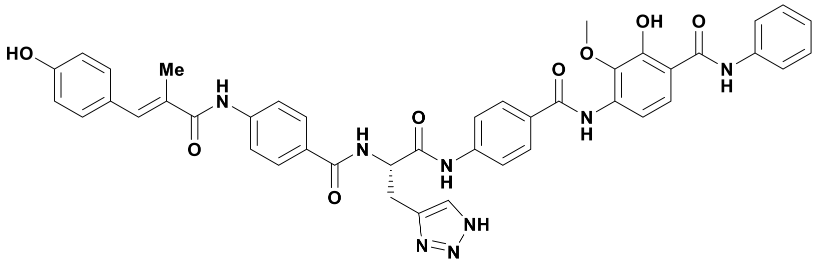

Chemical Formula: $C_{43}H_{38}N_8O_8$
Exact Mass: 794.2813

To a solution of tetrapeptide (**37e**, 374 mg, 561 µmol) in DMF (2 mL) was added perchlorophenyl (E)-4-(3-(4-hydroxyphenyl)-2-methylacrylamido)benzoate (**39**, 312 mg, 573 µmol) and NEt_3 (546 µL, 3.93 mmol). The reaction mixture was stirred for 2 d at 25°C and monitored by LC-MS (POM protected product: HRMS (ESI) calculated for $C_{49}H_{48}N_8O_{10}$ (M+H$^+$) 909.3566, found 909.3569). After full conversion KOH 3 N (1 mL) was added and stirred for 20 min. Next, the mixture was neutralized with HCl 6 N (0.5 mL) to a pH ~ 7. All volatiles were removed under reduced pressure, the residue was dissolved with DMSO (1 mL) and the salts were separated by centrifugation. The supernatant was loaded on the RP-HPLC system and purified under the following conditions. The collected fractions were lyophilized after their identity and purity was verified by LCMS to obtain 66.4 mg as a white solid (84 µmol, 15% over 2 steps).
RP-HPLC: 40-60% MeCN (+0.1% TFA) in milliQ H_2O (+0.1% TFA); 30 min; 50 mL/min.

^{1}H NMR (500 MHz, DMSO-*d*$_6$) δ 12.54 (s, 1H), 10.51 (s, 1H), 10.38 (s, 1H), 10.07 (s, 1H), 9.75 (s, 1H), 9.50 (s, 1H), 8.69 (d, *J* = 7.7 Hz, 1H), 7.96 (d, *J* = 8.8 Hz, 2H), 7.89 – 7.76 (m, 7H), 7.72 – 7.68 (m, 2H), 7.65 (d, *J* = 8.9 Hz, 1H), 7.40 (dd, *J* = 8.5, 7.4 Hz, 2H), 7.35 (d, *J* = 8.7 Hz, 2H), 7.26 (d, *J* = 1.6 Hz, 1H), 7.17 (tt, *J* = 7.3, 1.2 Hz, 1H), 6.84 (d, *J* = 8.7 Hz, 2H), 4.92 (q, *J* = 7.6 Hz, 1H), 3.87 (s, 3H), 3.28 – 3.16 (m, 2H), 2.11 (d, *J* = 1.3 Hz, 3H);
^{13}C NMR – from HSQC_ed (126 MHz, DMSO) δ 129.1, 129.1, 128.7, 128.6, 123.1, 123.3, 119.5, 119.4, 122.2, 122.2, 112.7, 112.8, 129.1, 129.2, 131.7, 131.8, 134.4, 125.1, 115.8, 115.8, 55.0, 60.6, 28.3, 14.9;
HRMS (ESI) calculated for $C_{43}H_{39}N_8O_8$ (M+H$^+$) 795.2885, found 795.2886, t_R = 8.48 min

1.2.6 Synthesis of E-Variations (9, 10)

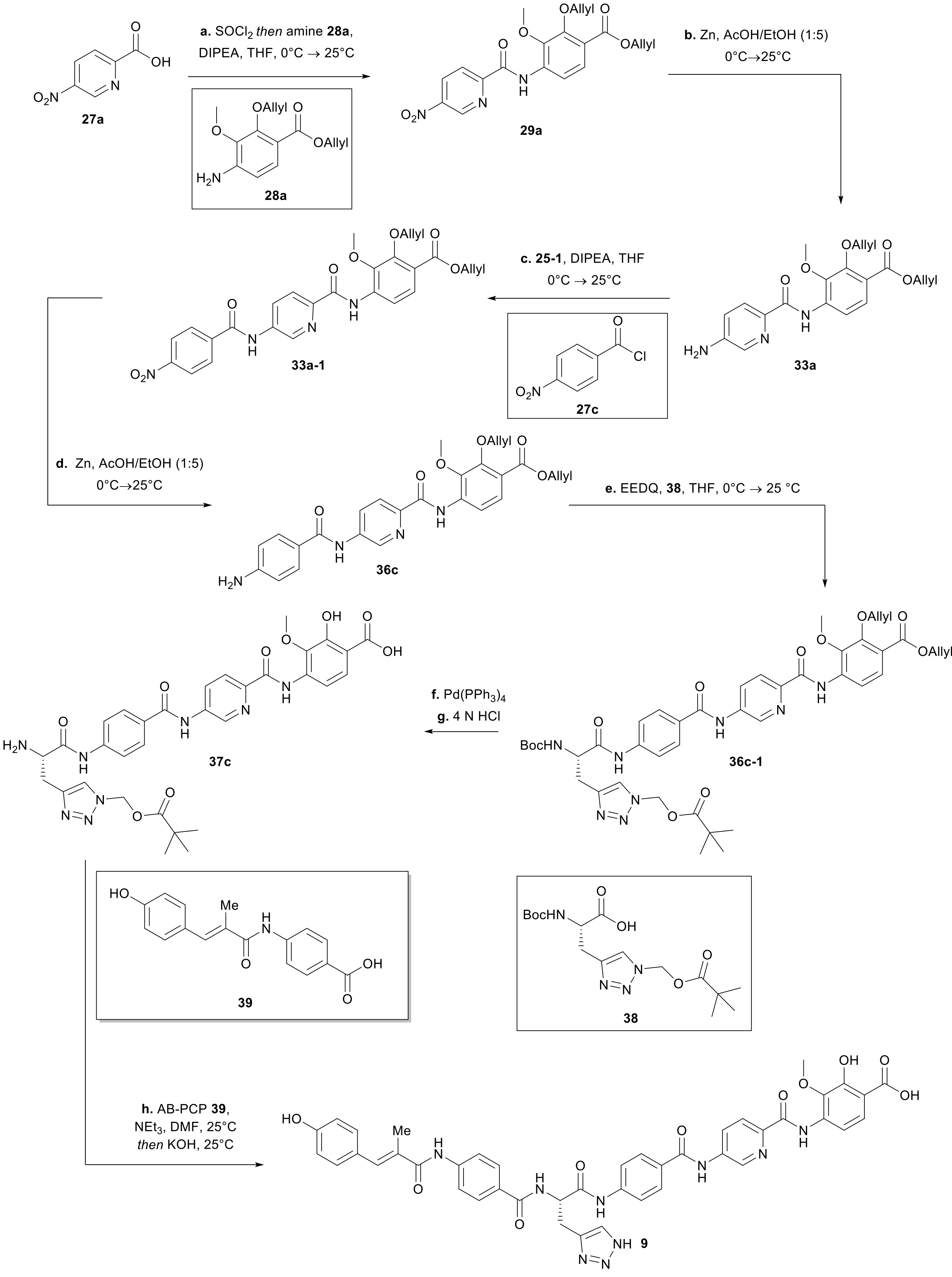

Scheme S 5. *Synthesis of E variation I to afford derivative **9**.*

Allyl 2-(allyloxy)-4-amino-3-methoxybenzoate (28a)[10]

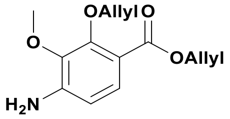

Chemical Formula: $C_{14}H_{17}NO_4$
Exact Mass: 263,1158

Synthesized according to exp. procedure reported.

i^{1}H NMR (500 MHz, DMSO-d_6) δ 7.34 (d, J = 8.6 Hz, 1H), 6.46 (d, J = 8.7 Hz, 1H), 6.03 (dddt, J = 38.7, 17.2, 10.7, 5.5 Hz, 2H), 5.76 (s, 2H), 5.35 (ddq, J = 17.2, 3.6, 1.8 Hz, 2H), 5.21 (ddq, J = 18.0, 10.4, 1.5 Hz, 2H), 4.66 (dt, J = 5.4, 1.5 Hz, 2H), 4.45 (dt, J = 5.6, 1.5 Hz, 2H), 3.69 (s, 3H).
^{13}C NMR (126 MHz, DMSO) δ 164.5, 153.1, 147.5, 138.9, 134.7, 133.2, 127.5, 117.4, 116.9, 110.8, 109.3, 74.2, 64.1, 59.6.
HRMS (ESI) calculated for $C_{14}H_{18}NO_4$ (M+H$^+$) 262.1085, found 262.1090.

Allyl 2-(allyloxy)-3-methoxy-4-(5-nitropicolinamido)benzoate (29a)

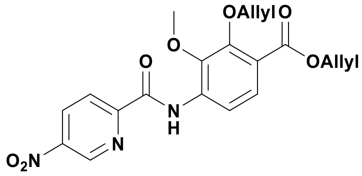

Chemical Formula: $C_{20}H_{19}N_3O_7$
Exact Mass: 413,1223

5-Nitropicolinic acid (**27a**, 479 mg, 2.85 mmol) were put in a 50 ml round bottom flask equipped with a condenser. $SOCl_2$ (2.75 mL, 38.0 mmol) was added, and the reaction mixture was heated to reflux and stirred under reflux for 2 h. Then, the $SOCl_2$ was evaporated under reduced pressure and the resulted acid chloride was used without further purification. The residue was dissolved in dry THF (3 mL) and added to a solution of allyl 2-(allyloxy)-4-amino-3-methoxybenzoate (**28a**, 500 mg, 1.90 mmol) and DIPEA (0.676 mL, 3.80 mmol) in dry THF (3 mL) at 0℃. The reaction mixture was kept under N_2 atmosphere, allowed to reach room temperature, and stirred at room temperature overnight. The reaction mixture was quenched with 1N HCl (5 mL) and extracted with three portions of EtOAc (20 mL). The combined organic layers were washed with $NaHCO_3$ saturated solution (20 mL), brine (20 mL) and concentrated under reduced pressure. The crude mixture was purified on a silica column (product eluted at 1:2 EtOAc:Hex) to give 711 mg of grey solid (1.72 mmol, 89%).

^{1}H NMR (500 MHz, DMSO-d_6) δ 10.55 (s, 1H), 9.49 (dd, J = 2.5, 0.7 Hz, 1H), 8.83 (dd, J = 8.6, 2.6 Hz, 1H), 8.39 (dd, J = 8.6, 0.7 Hz, 1H), 8.24 (d, J = 8.7 Hz, 1H), 7.57 (d, J = 8.7 Hz, 1H), 6.06 (dddt, J = 30.9, 17.2, 10.8, 5.6 Hz, 2H), 5.40 (ddq, J = 17.2, 11.3, 1.6 Hz, 2H), 5.26 (ddq, J = 16.9, 10.4, 1.4 Hz, 2H), 4.77 (dt, J = 5.5, 1.5 Hz, 2H), 4.55 (dt, J = 5.8, 1.5 Hz, 2H), 3.96 (s, 3H).
^{13}C NMR (126 MHz, DMSO) δ 164.3, 160.0, 152.6, 151.1, 146.1, 144.2, 142.9, 135.1, 134.0, 133.8, 132.5, 126.3, 123.1, 121.2, 118.1, 117.9, 114.1, 74.6, 65.2, 61.2.

Allyl 2-(allyloxy)-4-(5-aminopicolinamido)-3-methoxybenzoate (33a)

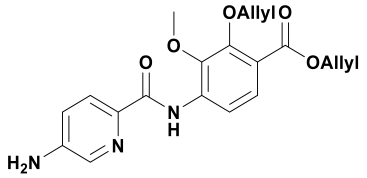

Chemical Formula: $C_{20}H_{21}N_3O_5$
Exact Mass: 383,1481

Allyl 2-(allyloxy)-3-methoxy-4-(5-nitropicolinamido)benzoate (**29a**, 700 mg, 1.69 mmol) was dissolved in a mixture of EtOH (8 mL) and AcOH (2 mL). The mixture was cooled to 0℃ and Zn powder (4.42 g, 67.7 mmol) was added. The reaction was stirred at 0℃ for 30 minutes, filtered over celite® and the residue was washed with three portions of CH_2Cl_2. The filtrate was concentrated under reduced pressure, redissolved in EtOAc (20 mL) and washed with three portions of a saturated solution of $NaHCO_3$ (10 mL) and one portion of brine (10 m). Then, the organic phase was concentrated under reduced pressure to yield 591 mg of yellow solid (1.54 mmol, 92%) that was used without further purification.

^{1}H NMR (500 MHz, DMSO-d_6) δ 10.42 (s, 1H), 8.31 (d, *J* = 8.8 Hz, 1H), 7.99 (d, *J* = 2.6 Hz, 1H), 7.86 (d, *J* = 8.5 Hz, 1H), 7.57 (d, *J* = 8.8 Hz, 1H), 7.05 (dd, *J* = 8.5, 2.6 Hz, 1H), 6.24 (s, 2H), 6.12 – 5.98 (m, 2H), 5.40 (ddq, *J* = 17.3, 12.4, 1.7 Hz, 2H), 5.25 (ddq, *J* = 18.2, 10.4, 1.4 Hz, 3H), 4.76 (dt, *J* = 5.5, 1.5 Hz, 2H), 4.54 (dt, *J* = 5.7, 1.4 Hz, 2H), 3.91 (s, 3H).
^{13}C NMR (126 MHz, DMSO) δ 164.4, 162.4, 151.3, 148.5, 142.1, 136.4, 136.0, 134.5, 134.0, 132.6, 126.5, 123.6, 119.6, 119.3, 118.1, 117.8, 113.4, 74.6, 65.0, 61.0.

Allyl 2-(allyloxy)-3-methoxy-4-(5-(4-nitrobenzamido)picolinamido)benzoate (33a-1)

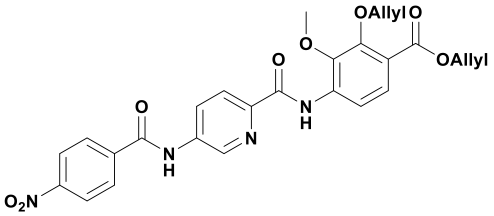

Chemical Formula: $C_{27}H_{24}N_4O_8$
Exact Mass: 532,1594

Allyl 2-(allyloxy)-4-(5-aminopicolinamido)-3-methoxybenzoate (**33a**, 585 mg, 1.53 mmol) and DIPEA (532 mL, 3.05 mmol) were dissolved in dry THF (5 mL) and cooled to 0℃. *Para*-nitro benzoylchloride (**27c**, 340 mg, 1.83 mmol) in THF (5 mL) was added and the reaction mixture was stirred overnight at room temperature under N_2 atmosphere. Then, the reaction mixture was quenched with 1N HCl (10 mL) and washed with three portions EtOAc (20 mL). The combined organic fractions were washed with a saturated solution of $NaHCO_3$ (20 mL), dried over Na_2SO_4, filtered, and concentrated under reduced pressure. The crude mixture was purified on a silica column (20-60% EtOAc in hexane). The product eluted at 60% EtOAc in hexane to yield 582 mg (1.09 mmol, 72%).

^{1}H NMR (500 MHz, DMSO-d_6) δ 11.12 (s, 1H), 10.58 (s, 1H), 9.12 (dd, *J* = 2.4, 0.7 Hz, 1H), 8.49 (dd, *J* = 8.6, 2.4 Hz, 1H), 8.45 – 8.38 (m, 2H), 8.32 (d, *J* = 8.8 Hz, 1H), 8.27 – 8.20 (m, 3H), 7.60 (d, *J* = 8.7 Hz, 1H), 6.16 – 5.98 (m, 2H), 5.41 (ddq, *J* = 17.2, 10.8, 1.7 Hz, 2H), 5.27 (ddq, *J* = 16.4, 10.4, 1.5 Hz, 2H), 4.77 (dt, *J* = 5.5, 1.5 Hz, 2H), 4.56 (dt, *J* = 5.7, 1.4 Hz, 2H), 3.96 (s, 3H).
^{13}C NMR (126 MHz, DMSO) δ 164.7, 164.4, 161.4, 151.2, 149.5, 143.8, 142.5, 140.4, 139.6, 138.7, 135.8, 133.9, 132.6, 129.5, 128.2, 126.5, 123.7, 122.9, 120.5, 119.6, 118.1, 117.9, 113.8, 74.6, 65.1, 61.1.

Allyl 2-(allyloxy)-4-(5-(4-aminobenzamido)picolinamido)-3-methoxybenzoate (36c)

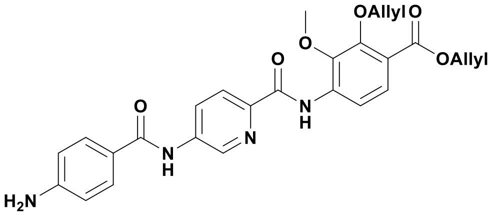

Chemical Formula: $C_{27}H_{26}N_4O_6$
Exact Mass: 502,1852

Allyl 2-(allyloxy)-3-methoxy-4-(5-(4-nitrobenzamido)picolinamido)benzoate (**33a-1**, 400 mg, 751 µmol) was dissolved in a mixture of EtOH (8 mL) and AcOH (2 mL). The mixture was cooled to 0℃ and zinc dust (1.96 g, 30.1 mmol) was added. The reaction was stirred at 0℃ for 30 minutes, filtered over celite® and the residue was washed with three portions of CH_2Cl_2. The filtrate was concentrated under reduced pressure, redissolved in EtOAc (20 mL) and washed with three portions of a saturated solution of $NaHCO_3$ (10 mL) and one portion of brine (10 m). Then, the organic phase was concentrated under reduced pressure to give 320 mg of a brown solid (637 µmol, 57%) that was used without further purification.

^{1}H NMR (500 MHz, DMSO-d_6) δ 10.58 (s, 1H), 10.32 (s, 1H), 9.10 (d, *J* = 2.4 Hz, 1H), 8.46 (dd, *J* = 8.6, 2.4 Hz, 1H), 8.33 (d, *J* = 8.8 Hz, 1H), 8.18 (d, *J* = 8.6 Hz, 1H), 7.80 – 7.74 (m, 2H), 7.60 (d, *J* = 8.7 Hz, 1H), 6.66 – 6.61 (m, 2H), 6.16 – 5.98 (m, 2H), 5.90 (s, 2H), 5.46 – 5.35 (m, 2H), 5.27 (ddq, *J* = 17.2, 10.4, 1.5 Hz, 2H), 4.78 (dt, *J* = 5.5, 1.5 Hz, 2H), 4.56 (dt, *J* = 5.8, 1.5 Hz, 2H), 3.96 (s, 3H).

Allyl (S)-2-(allyloxy)-4-(5-(4-(2-((tert-butoxycarbonyl)amino)-3-(1-((pivaloyloxy)methyl)-1H-1,2,3-triazol-4-yl)propanamido)benzamido)picolinamido)-3-methoxybenzoate (36c-1)

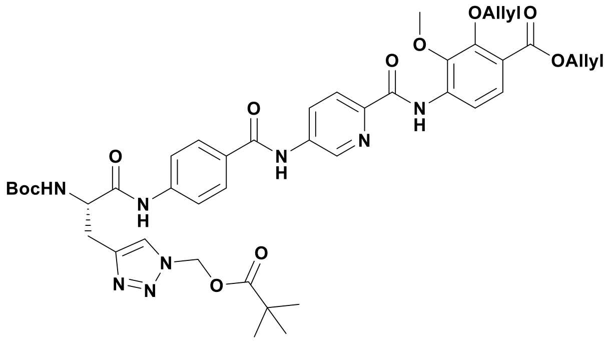

Chemical Formula: $C_{43}H_{50}N_8O_{11}$
Exact Mass: 854,3599

To a solution of EEDQ (393 mg, 1.59 mmol) in THF (10 mL) was added (S)-2-((tert-butoxycarbonyl)amino)-3-(1-((pivaloyloxy)methyl)-1H-1,2,3-triazol-4-yl)propanoic acid (**38**, 470 mg, 1.27 mmol) at 0°C and stirred for 15 min. Next a solution of allyl 2-(allyloxy)-4-(5-(4-aminobenzamido)picolinamido)-3-methoxybenzoate (**36c**, 320 mg, 636 µmol) in THF (5 mL) was added at 0°C, the reaction mixture was allowed to warm to ambient temperature and stirred for 18 h. All volatiles were removed under reduced pressure and the residue was dissolved in dichloromethane, the organic phase was washed with HCl 1 N (50 mL), a saturated solution of $NaHCO_3$ (50 mL) and brine (50 mL), dried over sodium sulfate and evaporated. The residue was purified by column chromatography on silica gel (eluent: 0→5% Acetone in CH_2Cl_2) to give 315 mg of a colorless solid (368 µmol, 55%).

^{1}H NMR (500 MHz, DMSO-d_6) δ 10.70 (s, 1H), 10.59 (s, 1H), 10.43 (s, 1H), 9.13 (d, *J* = 2.5 Hz, 1H), 8.49 (dd, *J* = 8.6, 2.4 Hz, 1H), 8.33 (d, *J* = 8.7 Hz, 1H), 8.25 – 8.20 (m, 1H), 8.03 – 7.93 (m, 3H), 7.81 – 7.75 (m, 2H), 7.61 (d, *J* = 8.7 Hz, 1H), 7.20 (d, *J* = 8.1 Hz, 1H), 6.29 (s, 2H), 6.16 – 5.98 (m, 2H), 5.41 (ddq, *J* = 17.2, 11.2, 1.7 Hz, 2H), 5.27 (ddq, *J* = 17.0, 10.5, 1.4 Hz, 2H), 4.78 (dt, *J* = 5.5, 1.5 Hz, 2H), 4.56 (dt, *J* = 5.7, 1.5 Hz, 2H), 4.41 (q, *J* = 8.5, 8.1 Hz, 1H), 3.96 (s, 3H), 3.13 (dd, *J* = 14.8, 5.0 Hz, 1H), 3.00 (dd, *J* = 14.7, 9.5 Hz, 1H), 1.36 (s, 9H), 1.10 (s, 9H).
^{13}C NMR (126 MHz, DMSO – from HSQC_ed) δ 140.7, 128.3, 114.3, 123.4, 129.4, 119.1, 124.5, 119.1, 129.4, 127.0, 73.3, 134.4, 134.1, 133.2, 118.7, 118.4, 118.7, 118.4, 118.7, 118.5, 118.4, 65.5, 75.2, 55.5, 61.6, 28.3, 28.3, 28.7, 27.0.

(S)-4-(5-(4-(2-((tert-butoxycarbonyl)amino)-3-(1-((pivaloyloxy)methyl)-1H-1,2,3-triazol-4-yl)propanamido)benzamido)picolinamido)-2-hydroxy-3-methoxybenzoic acid (36c-2)

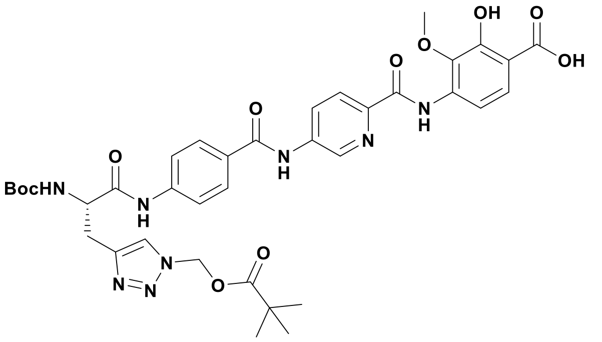

Chemical Formula: $C_{43}H_{50}N_8O_{11}$
Exact Mass: 854,3599

Allyl (S)-2-(allyloxy)-4-(5-(4-(2-((tert-butoxycarbonyl)amino)-3-(1-((pivaloyloxy)methyl)-1H-1,2,3-triazol-4-yl)propanamido)benzamido)picolinamido)-3-methoxybenzoate (**36c-1**, 300 mg, 350 µmol) and morpholine (0.605 mL, 7.02 mmol) were dissolved in dry THF (5 ml). $Pd(PPh_3)_4$ (162 mg, 140 µmol) was added. The reaction mixture was kept under N_2 atmosphere and protected from light and stirred at room temperature for 24 h. After removing all volatiles under reduced pressure, the residue was taken up in EtOAc and washed with 10% HCl(aq.) (3× 20 mL), a saturated solution of $NaHCO_3$ (3× 20 mL) and brine (20 mL). The organic phase was dried over Na_2SO_4, filtered, and concentrated under reduced pressure. Purification of the crude product by column chromatography (5-20% MeOH in CH_2Cl_2) to give 102 mg as a yellowish solid (132 µmol, 36%).

^{1}H NMR (500 MHz, DMSO-d_6) δ 10.69 (s, 1H), 10.45 (d, *J* = 6.6 Hz, 2H), 9.10 (d, *J* = 2.4 Hz, 1H), 8.48 (dd, *J* = 8.5, 2.4 Hz, 1H), 8.19 (d, *J* = 8.6 Hz, 1H), 8.03 – 7.96 (m, 3H), 7.78 (d, *J* = 8.6 Hz, 3H), 7.47 (d, *J* = 8.6 Hz, 1H), 7.20 (d, *J* = 8.2 Hz, 1H), 6.29 (s, 2H), 4.41 (s, 1H), 3.92 (s, 3H), 3.13 (d, *J* = 14.9 Hz, 1H), 3.05 – 2.96 (m, 1H), 1.36 (s, 9H), 1.10 (s, 9H).
^{13}C NMR (126 MHz, DMSO – from HSQC_ed) δ 140.6, 128.3, 123.2, 123.3, 129.4, 124.5, 119.1, 125.2, 70.3, 55.3, 60.1, 28.6, 28.7, 29.4, 27.0.

(S)-4-(5-(4-(2-amino-3-(1-((pivaloyloxy)methyl)-1H-1,2,3-triazol-4-yl)propanamido)benzamido)picolinamido)-2-hydroxy-3-methoxybenzoic acid (37c)

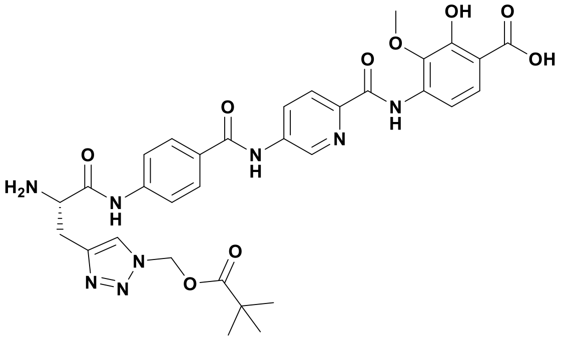

Chemical Formula: $C_{32}H_{34}N_8O_9$
Exact Mass: 674,2449

The Boc-protected tetrapeptide **36c-2** (100 mg, 0.13 mmol) was suspended in 4N HCl in 1,4-dioxane (5 mL) and the reaction mixture was stirred at room temperature for 1 h. All volatiles were subsequently removed under reduced pressure and the residue was taken up in a mixture of acetonitrile and little water and freeze-dried to afford compound **37c** (87 mg, 0.13 mmol, quant.) as a yellowish powder.

HRMS (ESI) calculated for $C_{32}H_{35}N_8O_9$ ($M+H^+$) 675.2522, found 675.2528.

(S,*E*)-2-hydroxy-4-(5-(4-(2-(4-(3-(4-hydroxyphenyl)-2-methylacrylamido)benzamido)-3-(1H-1,2,3-triazol-4-yl)propanamido)benzamido)picolinamido)-3-methoxybenzoic acid (9)

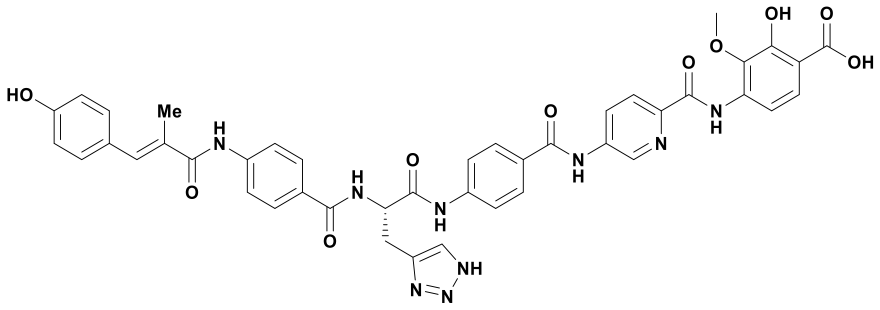

Chemical Formula: $C_{43}H_{37}N_9O_{10}$
Exact Mass: 839,2663

To a solution of tetrapeptide (**37c**, 50.0 mg, 74.1 µmol) in DMF (2 mL) was added perchlorophenyl (E)-4-(3-(4-hydroxyphenyl)-2-methylacrylamido)benzoate (**39**, 44.5 mg, 81.5 µmol) and NEt_3 (110 µL, 741 µmol). The reaction mixture was stirred for 2 d at 25°C and monitored by LC-MS. After full conversion KOH 3 N (1 mL) was added and stirred for 20 min. Next, the mixture was neutralized with HCl 6 N (0.5 mL) to a pH ~ 7. All volatiles were removed under reduced pressure, the residue was dissolved with DMSO (1 mL) and the salts were separated by centrifugation. The supernatant was loaded on the RP-HPLC system and purified under the following conditions. The collected fractions were lyophilized after their identity and purity was verified by LCMS to obtain 10 mg of a yellowish powder (11.9 µmol, 16% (over 2 steps)).
RP-HPLC: 35-50% MeCN (+0.1% TFA) in milliQ H_2O (+0.1% TFA); 30 min; 50 mL/min.

^{1}H NMR (500 MHz, DMSO-d_6) δ 10.72 (s, 1H), 10.58 (s, 1H), 10.54 (s, 1H), 10.07 (s, 1H), 9.76 (s, 1H), 9.53 (s, 1H), 9.12 (d, J = 2.4 Hz, 1H), 8.69 (s, 1H), 8.49 (dd, J = 8.6, 2.4 Hz, 1H), 8.22 (d, J = 8.6 Hz, 1H), 8.01 (d, J = 8.5 Hz, 3H), 7.90 – 7.79 (m, 7H), 7.67 – 7.56 (m, 2H), 7.35 (d, J = 8.3 Hz, 2H), 7.26 (s, 1H), 6.86 – 6.78 (m, 3H), 4.91 (d, J = 7.8 Hz, 1H), 3.95 (s, 3H), 3.27 – 3.20 (m, 3H), 2.11 (s, 3H).
^{13}C NMR (126 MHz, DMSO – from HSQC_ed) δ 140.7, 128.4, 123.2, 129.5, 128.7, 129.0, 119.4, 126.0, 131.8, 134.4, 115.8, 107.7, 54.8, 28.1, 15.0, 29.5.
HRMS (ESI) calculated for $C_{44}H_{38}N_8O_{10}$ (M+H$^+$) 840.2736, found 840.2758, t_R = 8.02 min.

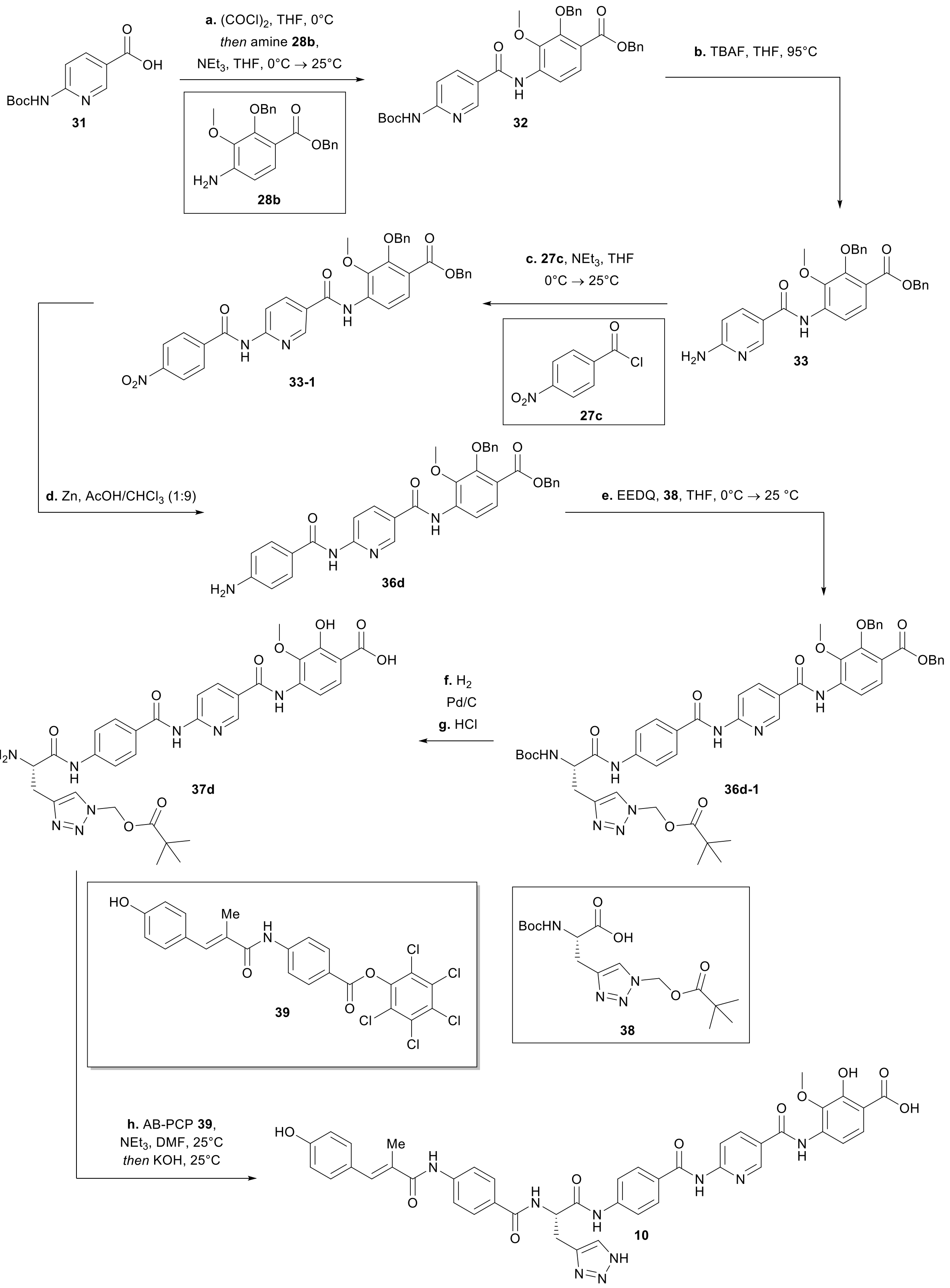

Scheme S 6. *Synthesis of E variation II to afford derivative* ***10****.*

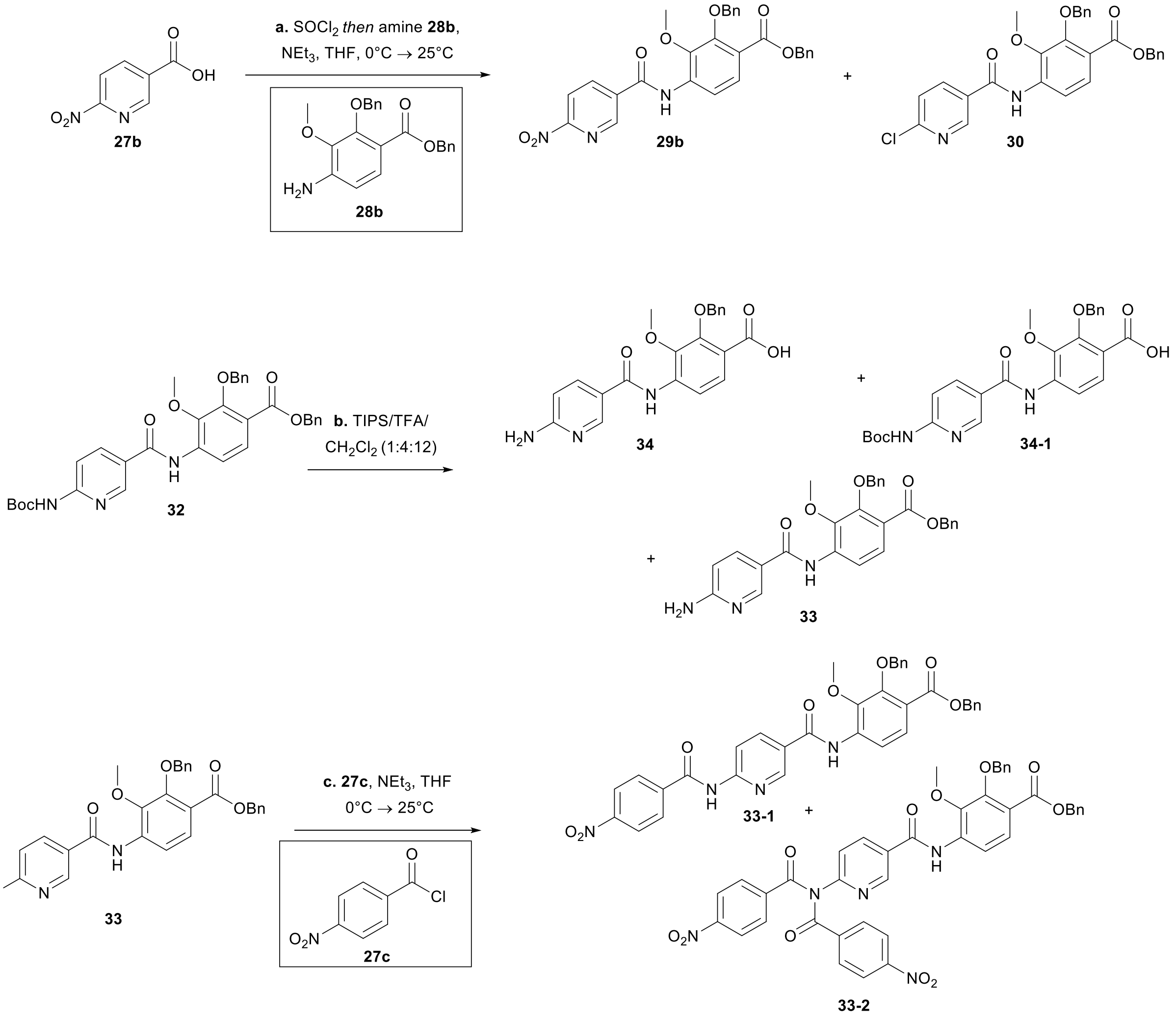

Scheme S 7. Observed side reactions during the synthesis of derivative 10.

6-((Tert-butoxycarbonyl)amino)nicotinic acid (31)

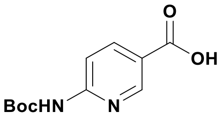

Chemical Formula: $C_{11}H_{14}N_2O_4$
Exact Mass: 238,0954

Methyl 6-((tert-butoxycarbonyl)amino)nicotinate (3.55 g, 14.1 mmol) was dissolved in EtOH (20 mL) and THF (20 mL). Next 3N KOH (20 mL) was added, and the reaction mixture was stirred for 16 h at 25°C. The organic solvents were removed under reduced pressure and the residue from the aqueous phase was acidified with 1N HCl. The precipitate was filtered and dried in vacuo to obtain 2.81 g of a colorless solid (11.8 mmol, 84%).

^{1}H NMR (500 MHz, DMSO-d_6) δ 13.18 (s, 1H), 10.21 (s, 1H), 8.74 (d, *J* = 2.3 Hz, 1H), 8.19 (dd, *J* = 8.7, 2.4 Hz, 1H), 7.91 (d, *J* = 8.8 Hz, 1H), 1.48 (s, 9H).
^{13}C NMR (126 MHz, DMSO) δ 166.1, 155.5, 152.5, 149.6, 139.1, 111.2, 80.2, 27.9.
HRMS (ESI) calculated for $C_{11}H_{15}N_2O_4$ (M+H$^+$) 239.1026, found 239.1032.

2-Hydroxy-3-methoxy-4-nitrobenzoic acid (31-1)[11]

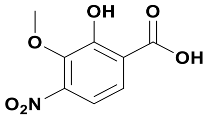

Chemical Formula: $C_8H_7NO_6$
Exact Mass: 213,0273

Synthesized according to exp. procedure reported.

^{1}H NMR (500 MHz, DMSO-d_6) δ 12.17 (s, 1H), 7.69 (d, *J* = 8.6 Hz, 1H), 7.32 (d, *J* = 8.6 Hz, 1H), 3.92 (s, 3H).
^{13}C NMR (126 MHz, DMSO) δ 170.8, 155.9, 147.3, 140.5, 125.2, 117.8, 112.8, 61.5.
HRMS (ESI) calculated for $C_8H_8NO_6$ (M−H$^+$) 212.0201, found 212.0201.

Benzyl 2-(benzyloxy)-3-methoxy-4-nitrobenzoate (31-2)

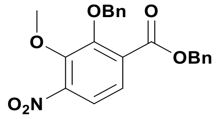

Chemical Formula: $C_{22}H_{19}NO_6$
Exact Mass: 393,1212

To a solution of 2-hydroxy-3-methoxy-4-nitrobenzoic acid (**31-1**, 20.0 g, 93.8 mmol) in DMF (120 mL) was added K_2CO_3 (38.9 g, 282 mmol), followed by dropwise addition of benzyl bromide (23.4 mL, 197 mmol). The reaction mixture was stirred for 48 h at 25°C. Et_2O (500 mL) and water (500 mL) were added, the organic phase washed then with a saturated solution of $NaHCO_3$ twice (400 mL) and once with brine (400 mL), dried over sodium sulphate, evaporated under reduced pressure. The residue was chromatographed on silica gel with a gradient 10-20% ethyl acetate in cyclohexane to give 28.4 g of a colorless solid (72.2 mmol, 77%).

^{1}H NMR (500 MHz, DMSO-d_6) δ 7.74 (d, *J* = 8.6 Hz, 1H), 7.62 (d, *J* = 8.6 Hz, 1H), 7.45 – 7.34 (m, 10H), 5.33 (s, 2H), 5.04 (s, 2H), 3.93 (s, 3H).

^{13}C NMR (126 MHz, DMSO) δ 164.1, 151.8, 146.7, 146.6, 136.0, 135.4, 130.7, 128.5, 128.4, 128.4, 128.3, 128.1, 125.3, 119.4, 76.0, 67.1, 62.4.
HRMS (ESI) calculated for $C_{22}H_{20}NO_6$ (M+H$^+$) 394.1285, found 394.1282.

Benzyl 4-amino-2-(benzyloxy)-3-methoxybenzoate (28b)

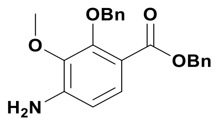

Chemical Formula: $C_{22}H_{21}NO_4$
Exact Mass: 363,1471

In a mixture of ethanol (250 mL) and acetic acid (50 mL) was dissolved benzyl 2-(benzyloxy)-3-methoxy-4-nitrobenzoate (**31-2**, 6.10 g, 15.5 mmol). At 0°C was added zinc (20.3 g, 310 mmol) in portions. The mixture was stirred for 20 min and allowed to warm to 25°C. Next, the suspension was filtered through celite® and washed with EtOAc. The filtrate was evaporated under reduced pressure. To the residue were added EtOAc (200 mL) and a saturated solution of $NaHCO_3$ (200 mL), the organic phase was washed with $NaHCO_3$ (3 x 200 mL) and brine (200 mL), dried over sodium sulphate, evaporated under reduced pressure to give 5.15 g of an orange solid (14.2 mmol, 91%).

^{1}H NMR (500 MHz, DMSO-d_6) δ 7.44 – 7.28 (m, 11H), 6.50 (d, *J* = 8.7 Hz, 1H), 5.82 (s, 2H), 5.22 (s, 2H), 4.93 (s, 2H), 3.69 (s, 3H).
^{13}C NMR (101 MHz, DMSO) δ 164.7, 153.2, 147.7, 139.0, 137.6, 136.7, 128.4, 128.2, 128.1, 128.0, 127.9, 127.8, 110.8, 109.5, 75.0, 65.3, 59.8.
HRMS (ESI) calculated for $C_{22}H_{22}NO_4$ (M+H$^+$) 364.1543, found 364.1535.

Benzyl 2-(benzyloxy)-3-methoxy-4-(6-nitronicotinamido)benzoate (29b)

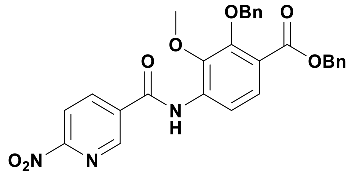

Chemical Formula: $C_{28}H_{23}N_3O_7$
Exact Mass: 513,1536

6-Nitronicotinic acid (**27b**, 1.00 g, 5.95 mmol) was dissolved in thionyl chloride (4.31 mL, 59.5 mmol) and refluxed for 2 h at 90°C. The thionyl chloride was removed under reduced pressure and the resulting acid chloride was dissolved in THF (10 mL). In parallel was prepared a mixture of benzyl 4-amino-2-(benzyloxy)-3-methoxybenzoate (**28b**, 2.16 g, 5.95 mmol) and triethylamine (1.66 mL, 11.9 mmol) in THF (10 mL). To the mixture was dropwise added the acid chloride solution at 0°C. The solution was allowed to warm to ambient temperature and stirred for 16 h. Diethylether (75 mL) was added to the reaction mixture and cooled to 0°C. The precipitate was collected by filtration and dissolved in dichloromethane (100 mL), water (100 mL) was added. The organic phase was washed with a saturated solution of $NaHCO_3$ (2 x 75 mL), HCl 1 N (3 x 75 mL) and brine (75 mL), dried over sodium sulfate and evaporated under reduced pressure. The residue was chromatographed on silica gel (gradient 10-20% ethyl acetate in cyclohexane, 0.1% triethylamine) to give 335 mg of a yellow solid (652 µmol, 11%) and the byproduct (see below).

^{1}H NMR (400 MHz, DMSO-d_6) δ 10.44 (s, 1H), 9.12 (dd, *J* = 2.3, 0.7 Hz, 1H), 8.67 (dd, *J* = 8.4, 2.3 Hz, 1H), 8.46 (dd, *J* = 8.4, 0.7 Hz, 1H), 7.87 (d, *J* = 8.7 Hz, 1H), 7.59 (d, *J* = 8.7 Hz, 1H), 7.45 – 7.33 (m, 10H), 5.31 (s, 2H), 5.00 (s, 2H), 3.85 (s, 3H).

^{13}C NMR (101 MHz, DMSO) δ 164.7, 163.2, 157.3, 151.6, 148.4, 145.7, 140.5, 136.8, 135.9, 135.7, 135.4, 128.5, 128.3, 128.2, 128.2, 128.1, 128.0, 125.6, 122.5, 118.9, 118.0, 75.5, 66.4, 61.1.
HRMS (ESI) calculated for $C_{28}H_{24}N_3O_7$ (M+H$^+$) 514.1609, found 514.1598.

Benzyl 2-(benzyloxy)-4-(6-chloronicotinamido)-3-methoxybenzoate (30)

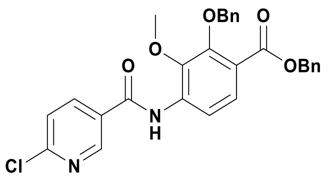

Chemical Formula: $C_{28}H_{23}ClN_2O_5$
Exact Mass: 502,1295

By-product of the reaction obtained during the preparation of **32**.
It was obtained as 741 mg of yellow solid (1.47 mmol, 25%).

^1H NMR (400 MHz, DMSO-d_6) δ 10.19 (s, 1H), 8.93 (dd, *J* = 2.6, 0.7 Hz, 1H), 8.33 (dd, *J* = 8.4, 2.5 Hz, 1H), 7.83 (d, *J* = 8.7 Hz, 1H), 7.71 (dd, *J* = 8.3, 0.7 Hz, 1H), 7.57 (d, *J* = 8.7 Hz, 1H), 7.43 – 7.34 (m, 10H), 5.31 (s, 2H), 4.99 (s, 2H), 3.84 (s, 3H).
^{13}C NMR (101 MHz, DMSO) δ 164.7, 163.6, 152.9, 151.5, 149.5, 145.7, 139.3, 136.8, 135.9, 129.5, 128.5, 128.4, 128.3, 128.2, 128.1, 128.1, 128.0, 128.0, 125.6, 124.1, 118.9, 75.5, 66.4, 61.0.
HRMS (ESI) calculated for $C_{28}H_{24}ClN_2O_5$ (M+H$^+$) 503.1368, found 503.1360.

Benzyl 2-(benzyloxy)-4-(6-((tert-butoxycarbonyl)amino)nicotinamido)-3-methoxybenzoate (32)

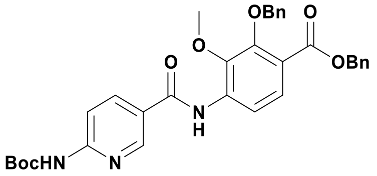

Chemical Formula: $C_{33}H_{33}N_3O_7$
Exact Mass: 583,2319

Oxalyl chloride (1.13 mL, 13.2 mmol) as a solution in THF (10 mL) was added to a stirred solution of 6-((tert-butoxycarbonyl)amino)nicotinic acid (**31**, 3.15 g, 13.2 mmol) and DMF (0.2 mL) in THF (40 mL) at 0°C. The reaction mixture was allowed to warm to ambient temperature over 3 hours. Next, triethylamine (3.07 mL, 22.0 mmol) and benzyl 4-amino-2-(benzyloxy)-3-methoxybenzoate (**28b**, 4.00 g, 11.0 mmol) as solution in THF (20 mL) were added at 0°C to the reaction mixture. The reaction mixture was allowed to warm to ambient temperature over 16 h. The solvent was removed under reduced pressure. Dichloromethane (100 mL) and water (100 mL) were added to the residue. The organic phase was washed with $NaHCO_3$ saturated solution (2 x 100 mL) and brine (100 mL), dried over sodium sulfate, concentrated under reduced pressure. The residue thus obtained was triturated with CH_2Cl_2 and chromatographed on silica gel with a gradient 0.4-4.0% acetone in CH_2Cl_2 with 0.1% NEt_3, in 0.4% steps to give 5.71 g of a colorless solid (9.78 mmol; 89%).

^1H NMR (500 MHz, DMSO-d_6) δ 10.20 (s, 1H), 9.87 (s, 1H), 8.83 (d, *J* = 2.4 Hz, 1H), 8.28 (dd, *J* = 8.9, 2.5 Hz, 1H), 7.93 (d, *J* = 8.8 Hz, 1H), 7.83 (d, *J* = 8.7 Hz, 1H), 7.56 (d, *J* = 8.7 Hz, 1H), 7.47 – 7.31 (m, 10H), 5.31 (s, 2H), 5.00 (s, 2H), 3.85 (s, 3H), 1.50 (s, 9H).
^{13}C NMR (101 MHz, DMSO) δ 164.7, 163.9, 154.9, 152.5, 151.5, 148.1, 145.6, 137.8, 136.8, 136.3, 136.0, 128.5, 128.3, 128.2, 128.1, 128.0, 125.6, 124.1, 121.9, 118.8, 111.0, 80.1, 75.5, 66.3, 61.0, 27.9.
HRMS (ESI) calculated for $C_{33}H_{34}N_3O_7$ (M+H$^+$) 584.2391, found 584.2380.

Benzyl 4-(6-aminonicotinamido)-2-(benzyloxy)-3-methoxybenzoate (34)

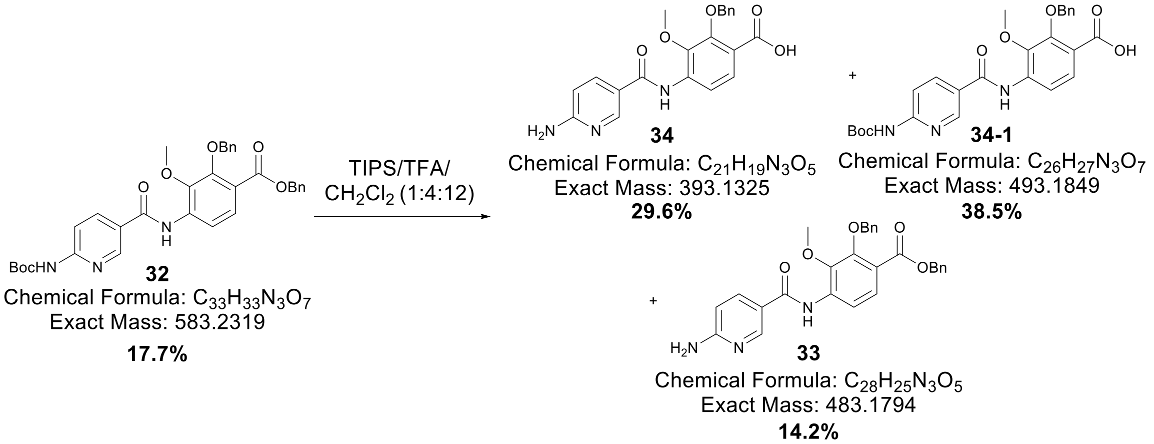

Benzyl 2-(benzyloxy)-4-(6-((tert-butoxycarbonyl)amino)nicotinamido)-3-methoxybenzoate (**32**, 30.0 mg, 51.4 µmol) was dissolved in mixture of triisopropylsilane (TIPS), trifluoroacetic acid (TFA) and CH_2Cl_2 (5 mL, 1:4:12) at 0°C. The reaction mixture was stirred for 1 h at room temperature and monitored by LC-MS. Then quenched with ice (5 mL), neutralized with $NaHCO_3$ saturated solution, and extracted with CH_2Cl_2 (2 x 20 mL). The organic extract was washed with water (10 mL), dried over Na_2SO_4 and filtered. The residue was purified by column chromatography (0.5−5.0% MeOH in CH_2Cl_2 with 0.1% NEt_3, in 0.5% steps) to separate one of the products, identified by MS, benzyl 4-(6-aminonicotinamido)-2-(benzyloxy)-3-methoxybenzoate (**34**, conversion= 29.6%) to give 3.5 mg of a white solid (8.90 µmol, isolated yield: 17%).

^{1}H NMR (500 MHz, DMSO-d_6) δ 10.76 (s, 1H), 9.36 (s, 1H), 8.55 (d, *J* = 2.5 Hz, 1H), 7.92 (dd, *J* = 8.8, 2.5 Hz, 1H), 7.67 (d, *J* = 8.9 Hz, 1H), 7.59 (d, *J* = 8.9 Hz, 1H), 7.52 – 7.47 (m, 2H), 7.42 (t, *J* = 7.4 Hz, 2H), 7.40 – 7.33 (m, 1H), 6.81 (s, 2H), 6.53 (d, *J* = 8.7 Hz, 1H), 5.41 (s, 2H), 3.85 (s, 3H);
^{13}C NMR (126 MHz, DMSO - from HSQC_ed) δ 149.1, 137.5, 125.2, 113.2, 125.2, 113.4, 128.8, 129.0, 107.8, 67.1, 60.7;

Number of detected peaks: 4

Apex RT	Start RT	End RT	Area	%Area	Found HRMS (ESI)	compounds	Calculated
6.33	6.2	6.51	297686.637	29.64	394.1394	**34**	394.1397
6.95	6.82	7.09	142337.624	14.17	484.1862	**33**	484.1867
10.15	9.99	10.23	177793.271	17.71	584.2390	**32**	584.2391
10.38	10.24	10.52	386376.758	38.48	494.1917	**34-1**	494.1922

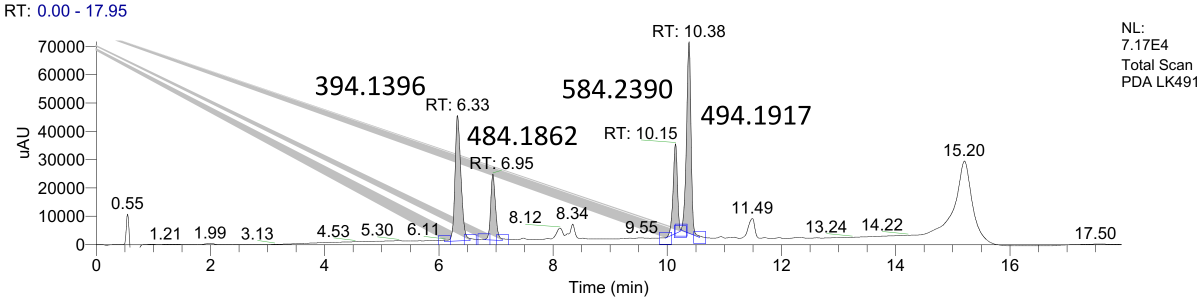

Benzyl 4-(6-aminonicotinamido)-2-(benzyloxy)-3-methoxybenzoate (34)

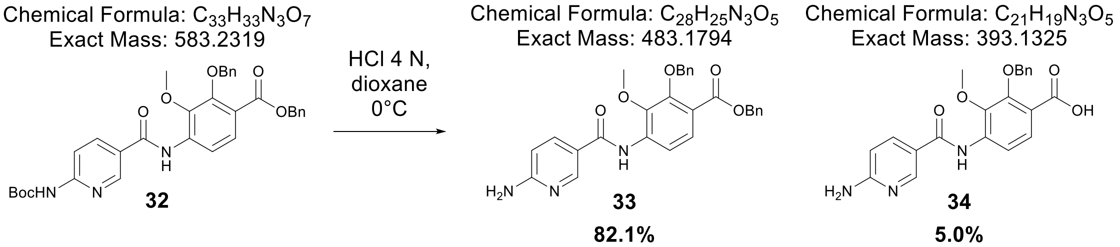

Benzyl 2-(benzyloxy)-4-(6-((tert-butoxycarbonyl)amino)nicotinamido)-3-methoxybenzoate (**32**, 3.0 mg, 5.14 µmol) was dissolved in a solution of HCl 4 N in dioxane at 0°C. The reaction mixture was stirred for 1 h at 0°C and monitored by LC-MS. Then quenched with ice (5 mL), neutralized with $NaHCO_3$ saturated solution, and extracted with CH_2Cl_2 (2 x 20 mL). The organic extract was washed with water (10 mL), dried over Na_2SO_4 and filtered. The residue was analyzed by LC-MS.

Number of detected peaks: 3

Apex RT	Start RT	End RT	Area	%Area	Found HRMS (ESI)	compounds	Calculated
6.75	6.41	7.00	2201599.717	82.10	394.1394	**34**	394.1394
7.16	7.06	7.46	346478.707	12.92	n.d.	-	n.d.
8.77	8.43	8.97	133508.105	4.98	484.1862	**33**	484.1866

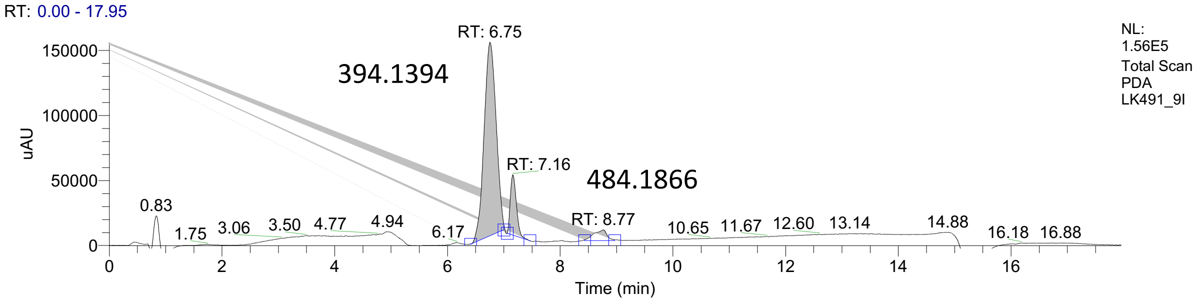

Benzyl 4-(6-aminonicotinamido)-2-(benzyloxy)-3-methoxybenzoate (33b)

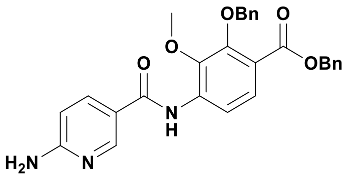

Chemical Formula: $C_{28}H_{25}N_3O_5$
Exact Mass: 483,1794

Benzyl 2-(benzyloxy)-4-(6-((tert-butoxycarbonyl)amino)nicotinamido)-3-methoxybenzoate (**32**, 1.15 g, 1.97 mmol) dissolved in dry THF (20 mL) and treated with tetrabutylammonium fluoride (19.7 mmol, 1 M solution in THF) at 120°C for 6 h. After completion of reaction, monitored by TLC (eluent cyclohexane /ethyl acetate 60:40), the reaction was quenched with saturated $NaHCO_3$ aqueous solution and extracted with dichloromethane (3 x 50 mL). Anhydrous Na_2SO_4 was added to the combined organic phases. After filtration of the salt, the organic phase was evaporated under reduced pressure. The residue was purified by silica gel chromatography (eluting with cyclohexane /ethyl acetate 2:1 → 1:2) to give 751 mg of a colorless solid (1.55 mmol, 79%).

^{1}H NMR (500 MHz, DMSO-d_6) δ 9.48 (s, 1H), 8.58 (d, J = 2.6 Hz, 1H), 7.98 – 7.91 (m, 1H), 7.86 (d, J = 8.7 Hz, 1H), 7.55 (d, J = 8.7 Hz, 1H), 7.45 – 7.33 (m, 10H), 6.83 (s, 1H), 6.54 (d, J = 8.8 Hz, 1H), 5.30 (s, 2H), 4.98 (s, 2H), 3.84 (s, 3H).
^{13}C NMR (126 MHz, DMSO) δ 165.2, 164.7, 152.0, 151.6, 145.5, 137.4, 137.2, 136.5, 129.0, 129.0, 128.8, 128.7, 128.7, 128.6, 128.5, 126.2, 121.6, 118.6, 118.0, 107.9, 75.9, 66.8, 61.4.
^{13}C NMR (126 MHz, DMSO) δ 164.8, 164.2, 151.5, 151.1, 145.0, 136.9, 136.8, 136.1, 128.5, 128.5, 128.3, 128.3, 128.2, 128.1, 128.0, 125.7, 121.2, 118.1, 117.6, 107.4, 75.5, 66.3, 61.0.
HRMS (ESI) calculated for $C_{28}H_{26}N_3O_5$ (M+H$^+$) 484.1867, found 484.1855.

Benzyl 2-(benzyloxy)-3-methoxy-4-(6-(4-nitro-N-(4-nitrobenzoyl)benzamido)nicotinamido)benzoate (33b-1)

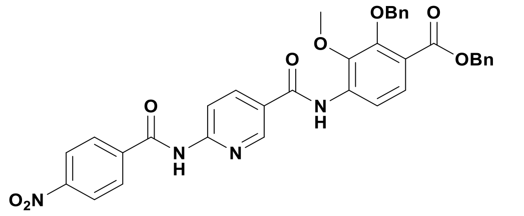

Chemical Formula: $C_{35}H_{28}N_4O_8$
Exact Mass: 632,1907

To a mixture of benzyl 4-(6-aminonicotinamido)-2-(benzyloxy)-3-methoxybenzoate (**33b**, 600 mg, 1.24 mmol) and triethylamine (0.208 mL, 1.49 mmol) in THF (5 mL) at 0°C was added a solution of 4-nitrobenzoyl chloride (276 mg, 1.49 mmol) in THF (5 mL). The solution was allowed to warm to 25°C and stirred for 3 d. All volatiles were removed under reduced pressure, the residue was diluted with dichloromethane (100 mL) and washed with $NaHCO_3$ saturated solution (3 x 100 mL) and brine (100 mL), dried over sodium sulfate and concentrated under reduced pressure. The residue was purified by column chromatography on silica gel (eluent: 0.5→5% acetone in dichloromethane) to give 307 mg of a white solid (485 µmol, 39%).

^{1}H NMR (500 MHz, DMSO-d_6) δ 11.56 (s, 1H), 10.03 (s, 1H), 8.99 (dd, J = 2.4, 0.8 Hz, 1H), 8.42 (dd, J = 8.7, 2.4 Hz, 1H), 8.39 – 8.32 (m, 3H), 8.29 – 8.23 (m, 2H), 7.84 (d, J = 8.6 Hz, 1H), 7.58 (d, J = 8.7 Hz, 1H), 7.46 – 7.38 (m, 5H), 7.38 – 7.31 (m, 5H), 5.31 (s, 2H), 5.01 (s, 2H), 3.86 (s, 3H).
^{13}C NMR (126 MHz, DMSO) δ 165.0, 164.7, 163.9, 154.2, 151.6, 149.5, 148.1, 145.7, 139.5, 138.2, 136.8, 136.2, 136.0, 129.8, 128.5, 128.4, 128.3, 128.2, 128.1, 128.0, 126.0, 125.6, 123.4, 122.1, 118.9, 113.6, 75.5, 66.4, 61.0.
HRMS (ESI) calculated for $C_{35}H_{29}N_4O_8$ (M+H$^+$) 633.1980, found 633.1976.

Benzyl 2-(benzyloxy)-3-methoxy-4-(6-(4-nitro-N-(4-nitrobenzoyl)benzamido)nicotinamido)benzoate (33b-2)

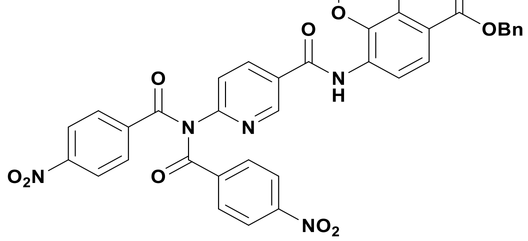

Chemical Formula: $C_{42}H_{31}N_5O_{11}$
Exact Mass: 781,2020

The compound **33b-2** was obtained as byproduct of the reaction towards benzyl 2-(benzyloxy)-3-methoxy-4-(6-(4-nitro-N-(4-nitrobenzoyl)benzamido)nicotinamido)benzoate (**33b-1**). This byproduct could be separated by the above-described column chromatography to give 309 mg of a white foam (395 µmol, 32%).

^{1}H NMR (500 MHz, DMSO-d_6) δ 10.15 (s, 1H), 8.76 (d, *J* = 2.5 Hz, 1H), 8.47 (dd, *J* = 8.4, 2.4 Hz, 1H), 8.34 – 8.25 (m, 4H), 8.12 – 8.06 (m, 4H), 7.98 (d, *J* = 8.4 Hz, 1H), 7.77 (d, *J* = 8.7 Hz, 1H), 7.55 (d, *J* = 8.7 Hz, 1H), 7.46 – 7.40 (m, 2H), 7.40 – 7.32 (m, 8H), 5.75 (s, 2H), 5.30 (s, 2H), 4.98 (s, 2H), 3.82 (s, 3H).
HRMS (ESI) calculated for $C_{42}H_{32}N_5O_{11}$ (M+H$^+$) 782.2093, found 782.2085.

Benzyl 4-(6-(4-aminobenzamido)nicotinamido)-2-(benzyloxy)-3-methoxybenzoate (36d)

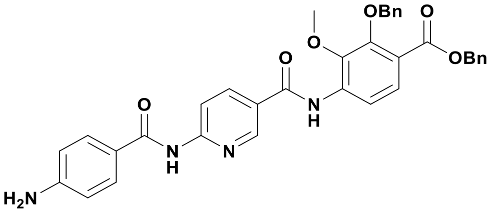

Chemical Formula: $C_{35}H_{30}N_4O_6$
Exact Mass: 602,2165

To Benzyl 2-(benzyloxy)-3-methoxy-4-(6-(4-nitro-N-(4-nitrobenzoyl)benzamido)nicotinamido)benzoate (**33b-1**, 300 mg, 474 µmol) in a mixture of chloroform (45 mL) and acetic acid (5 mL) at 0°C was added zinc dust (620 mg, 9.48 mmol) portion wise. The ice bath was removed after 10 min and the reaction mixture was stirred for further 30 min. The reaction was monitored by TLC, after full conversion the suspension was filtered over celite® and washed with dichloromethane (100 mL). The filtrate was washed with a saturated solution of $NaHCO_3$ (3 x 100 mL) and brine (100 mL), dried over sodium sulfate and evaporated under reduced pressure to give 236 mg of an orange solid (392 µmol, 83%) which was used without further purification.

^{1}H NMR (500 MHz, DMSO-d_6) δ 10.57 (s, 1H), 9.94 (s, 1H), 8.92 (dd, *J* = 2.3, 1.1 Hz, 1H), 8.38 – 8.28 (m, 2H), 7.84 (dd, *J* = 8.7, 6.8 Hz, 3H), 7.57 (d, *J* = 8.7 Hz, 1H), 7.46 – 7.32 (m, 10H), 6.59 (d, *J* = 8.7 Hz, 2H), 5.89 (s, 2H), 5.31 (s, 2H), 5.00 (s, 2H), 3.86 (s, 3H).
^{13}C NMR (126 MHz, DMSO) δ 166.3, 165.2, 164.5, 153.3, 152.4, 152.0, 148.5, 146.7, 146.1, 138.2, 137.3, 137.3, 136.8, 136.5, 130.6, 130.5, 129.0, 128.8, 128.7, 128.6, 128.5, 126.9, 126.1, 122.4, 120.1, 113.5, 113.0, 76.0, 66.8, 61.5.
^{13}C NMR (126 MHz, DMSO) δ 165.8, 164.7, 164.0, 152.9, 151.9, 151.5, 148.0, 146.2, 145.6, 137.7, 136.8, 136.8, 136.3, 136.0, 130.1, 130.1, 128.5, 128.3, 128.2, 128.1, 128.0, 126.4, 125.6, 121.9, 119.6, 113.0, 112.5, 75.5, 66.4, 61.0.
HRMS (ESI) calculated for $C_{35}H_{31}N_4O_6$ (M+H$^+$) 603.2238, found 603.2238.

Benzyl (S)-2-(benzyloxy)-4-(6-(4-(2-((tert-butoxycarbonyl)amino)-3-(1-((pivaloyloxy)methyl)-1H-1,2,3-triazol-4-yl)propanamido)benzamido)nicotinamido)-3-methoxybenzoate (36d-1)

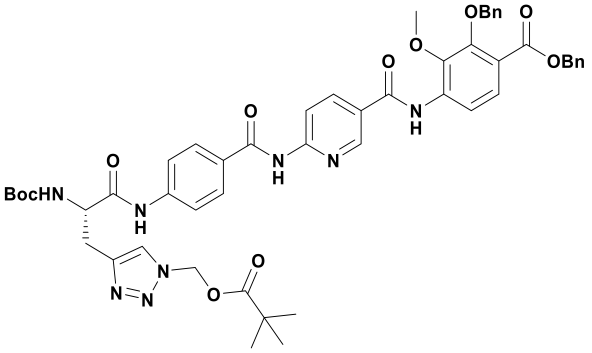

Chemical Formula: $C_{51}H_{54}N_8O_{11}$
Exact Mass: 954,3912

To a solution of EEDQ (212 mg, 861 µmmol) in THF (10 mL) was added (S)-2-((tert-butoxycarbonyl)amino)-3-(1-((pivaloyloxy)methyl)-1H-1,2,3-triazol-4-yl)propanoic acid (**38**, 212 mg, 574 µmol) at 0°C and stirred for 15 min. Next a solution of benzyl 4-(6-(4-aminobenzamido)nicotinamido)-2-(benzyloxy)-3-methoxybenzoate (**36d**, 173 mg, 287 µmol) in THF (5 mL) was added at 0°C, the reaction mixture was allowed to warm to ambient temperature and stirred for 9 d. All volatiles were removed under reduced pressure and the residue was dissolved in dichloromethane, the organic phase was washed with HCl 1 N (50 mL), a saturated solution of $NaHCO_3$ (50 mL) and brine (50 mL), dried over sodium sulfate and evaporated. The residue was purified by column chromatography on silica gel (eluent: 1→3% MeOH in dichloromethane) to give 166 mg of a colorless solid (174 µmol, 61%).

^{1}H NMR (500 MHz, DMSO-d_6) δ 11.03 (s, 1H), 10.41 (s, 1H), 9.99 (s, 1H), 8.99 – 8.95 (m, 1H), 8.42 – 8.32 (m, 2H), 8.09 – 8.01 (m, 2H), 7.98 (d, *J* = 8.0 Hz, 1H), 7.85 (d, *J* = 8.7 Hz, 1H), 7.74 (d, *J* = 8.6 Hz, 2H), 7.59 (d, *J* = 8.7 Hz, 1H), 7.45 (dd, *J* = 7.9, 1.8 Hz, 2H), 7.44 – 7.32 (m, 8H), 7.20 (d, *J* = 7.8 Hz, 1H), 6.29 (s, 2H), 5.32 (s, 2H), 5.01 (s, 2H), 4.41 (s, 1H), 3.87 (s, 3H), 3.11 (s, 1H), 3.05 – 2.96 (m, 1H), 1.37 (s, 9H), 1.10 (s, 9H).
^{13}C NMR (126 MHz, DMSO - from HSQC_ed) δ 148.5, 138.6, 113.7, 129.7, 124.3, 119.5, 118.9, 125.9, 128.7, 128.8, 128.8, 128.7, 70.2, 66.8, 76.0, 61.5, 40.3, 28.7, 27.0
HRMS (ESI) calculated for $C_{51}H_{55}N_8O_{11}$ (M+H$^+$) 955.3985, found 955.4004.

(S)-4-(6-(4-(2-((tert-butoxycarbonyl)amino)-3-(1-((pivaloyloxy)methyl)-1H-1,2,3-triazol-4-yl)propanamido)benzamido)nicotinamido)-2-hydroxy-3-methoxybenzoic acid (36d-2)

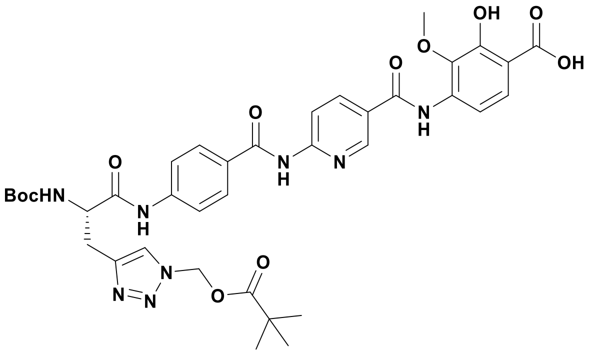

Chemical Formula: $C_{37}H_{42}N_8O_{11}$
Exact Mass: 774,2973

A mixture of benzyl (S)-2-(benzyloxy)-4-(6-(4-(2-((tert-butoxycarbonyl)amino)-3-(1-((pivaloyloxy)methyl)-1H-1,2,3-triazol-4-yl)propanamido)benzamido)nicotinamido)-3-methoxybenzoate (**36d-1**, 160 mg, 168 µmol) and 10% Pd/C (32 mg), in 1:1:1 mixture of CH_2Cl_2/MeOH/THF (60 mL), was stirred for 1 h under H_2 atmosphere. After the catalyst was filtered and washed with MeOH, the filtrate was evaporated to give 120 mg of a white solid (155 µmol, 92%).

HRMS (ESI) calculated for $C_{37}H_{43}N_8O_{11}$ (M+H$^+$) 775.3046, found 775.3047.

(S)-4-(6-(4-(2-amino-3-(1-((pivaloyloxy)methyl)-1H-1,2,3-triazol-4-yl)propanamido)benzamido)nicotinamido)-2-hydroxy-3-methoxybenzoic acid (37d)

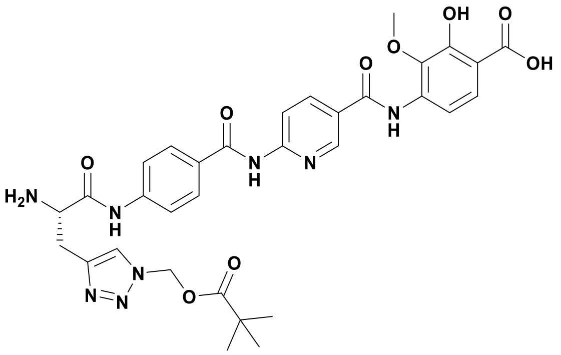

Chemical Formula: $C_{32}H_{34}N_8O_9$
Exact Mass: 674,2449

(S)-4-(6-(4-(2-((tert-butoxycarbonyl)amino)-3-(1-((pivaloyloxy)methyl)-1H-1,2,3-triazol-4-yl)propanamido)benzamido)nicotinamido)-2-hydroxy-3-methoxybenzoic acid (**36d-2**, 111 mg, 143 µmol) was dissolved in HCl 4 N in dioxane (10 mL) and stirred for 1 h at 25°C. All volatiles were removed under reduced pressure, the residue was dissolved in 2:1 mixture of water/MeCN and lyophilized to give 92 mg of fluffy white solid (136 mmol, 95%).

^{1}H NMR (400 MHz, DMSO-d_6) δ 11.27 (s, 1H), 11.11 (s, 1H), 9.88 (s, 1H), 8.94 (dd, *J* = 2.4, 0.9 Hz, 1H), 8.59 (d, *J* = 5.6 Hz, 2H), 8.42 – 8.29 (m, 2H), 8.15 (d, *J* = 8.8 Hz, 1H), 8.07 (dd, *J* = 8.9, 2.0 Hz, 2H), 7.74 (d, *J* = 8.9 Hz, 2H), 7.58 (s, 2H), 6.28 (s, 2H), 4.42 (d, *J* = 5.9 Hz, 1H), 3.87 (s, 3H), 3.40 – 3.31 (m, 2H), 1.07 (s, 9H).
^{13}C NMR (101 MHz, DMSO) δ 176.4, 172.0, 166.8, 165.5, 163.9, 154.8, 154.6, 147.9, 141.6, 140.9, 138.8, 138.2, 137.0, 129.3, 128.8, 125.6, 125.0, 124.8, 118.8, 113.4, 113.2, 110.1, 69.9, 60.2, 52.7, 38.2, 27.1, 26.5.
HRMS (ESI) calculated for $C_{32}H_{35}N_8O_9$ (M+H$^+$) 675.2522, found 675.2527.

(S,E)-2-Hydroxy-4-(6-(4-(2-(4-(3-(4-hydroxyphenyl)-2-methylacrylamido)benzamido)-3-(1H-1,2,3-triazol-4-yl)propanamido)benzamido)nicotinamido)-3-methoxybenzoic acid (10)

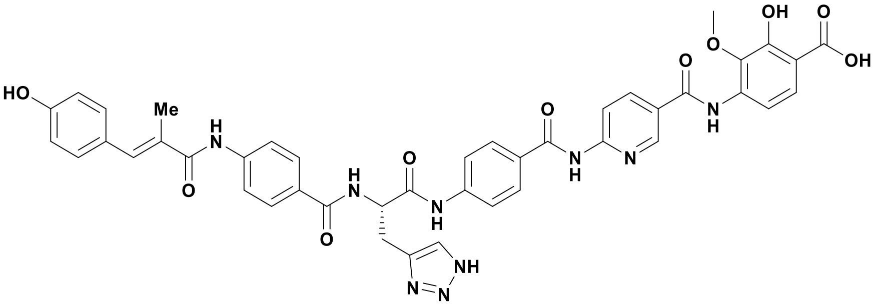

Chemical Formula: $C_{43}H_{37}N_9O_{10}$
Exact Mass: 839,2663

To a solution of tetrapeptide(**37d**, 83.0 mg, 117 µmol) in DMF (2 mL) was added perchlorophenyl (E)-4-(3-(4-hydroxyphenyl)-2-methylacrylamido)benzoate (**39**, 82.3 mg, 140 µmol) and NEt_3 (113 µL, 817 µmol)). The reaction mixture was stirred for 4 d at 25°C and monitored by LC-MS (POM protected product: HRMS (ESI) calculated for $C_{50}H_{49}N_9O_{12}$ (M+H$^+$) 968.3573, found 968.3574). After full conversion KOH 3 N (1 mL) was added and stirred for 20 min. Next, the mixture was neutralized with HCl 6 N (0.5 mL) to a pH ~ 7. All volatiles were removed under reduced pressure, the residue was dissolved with DMSO (1 mL) and the salts were separated by centrifugation. The supernatant was loaded on the RP-HPLC system and purified under the following conditions. The collected fractions were lyophilized after their identity and purity was verified by LCMS to obtain 12 mg of a fluffy white solid (14 µmol, 12% (over 2 steps)).
RP-HPLC: 40-57% MeCN (+0.1% TFA) in milliQ H_2O (+0.1% TFA); 30 min; 50 mL/min.

^{1}H NMR (500 MHz, DMSO-d_6) δ 11.61 (s, 1H), 11.03 (s, 1H), 10.50 (s, 1H), 10.07 (s, 1H), 9.84 (s, 1H), 9.75 (s, 1H), 8.93 (dd, *J* = 2.3, 1.1 Hz, 1H), 8.69 (d, *J* = 7.6 Hz, 1H), 8.39 – 8.31 (m, 2H), 8.06 (d, *J* = 8.8 Hz, 2H), 7.89 – 7.75 (m, 6H), 7.68 (s, 1H), 7.58 (s, 2H), 7.36 – 7.32 (m, 2H), 7.26 (d, *J* = 1.6 Hz, 1H), 6.87 – 6.83 (m, 2H), 4.93 – 4.88 (m, 1H), 3.87 (s, 3H), 3.30 – 3.19 (m, 2H), 2.11 (d, *J* = 1.4 Hz, 3H).
^{13}C NMR (176 MHz, DMSO – from HSQC_ed) δ 148.5, 138.4, 113.6, 129.8, 128.7, 119.3, 119.5, 119.0, 113.5, 125.3, 131.8, 134.4, 115.8, 54.6, 60.6, 66.8, 15.0.
HRMS (ESI) calculated for $C_{43}H_{38}N_9O_{10}$ (M+H$^+$) 840.2736, found 840.2740, t_R= 7.54 min.

1.2.7 Synthesis of D-Variations (7, 8)

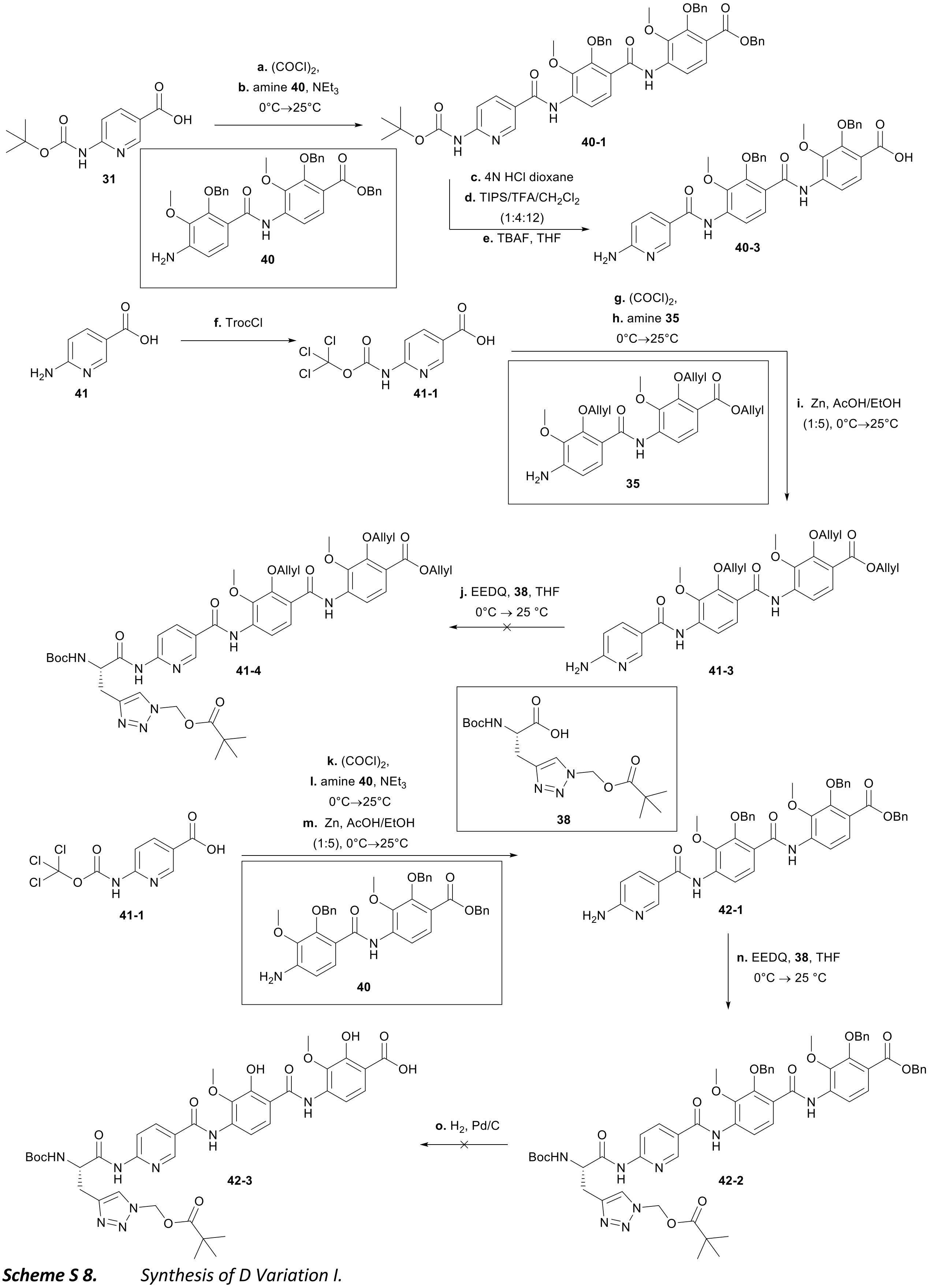

Scheme S 8. *Synthesis of D Variation I.*

Benzyl 4-(4-amino-2-(benzyloxy)-3-methoxybenzamido)-2-(benzyloxy)-3-methoxybenzoate (40)[8]

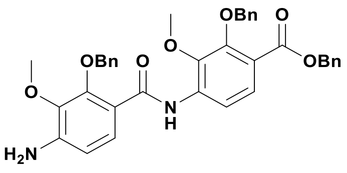

Chemical Formula: $C_{37}H_{34}N_2O_7$
Exact Mass: 618.2366

Synthesized according to exp. procedure reported.

^{1}H NMR (500 MHz, DMSO-d_6) δ 10.58 (s, 1H), 8.30 (d, *J* = 8.8 Hz, 1H), 7.55 (dd, *J* = 8.8, 6.2 Hz, 2H), 7.44 (td, *J* = 7.9, 1.8 Hz, 4H), 7.41 – 7.30 (m, 12H), 6.58 (d, *J* = 8.8 Hz, 1H), 5.88 (s, 2H), 5.29 (s, 2H), 5.25 (s, 2H), 4.91 (s, 2H), 3.80 (s, 3H), 3.50 (s, 3H).
^{13}C NMR (126 MHz, DMSO) δ 164.6, 162.9, 151.1, 150.8, 142.1, 137.7, 137.4, 136.8, 136.1, 136.1, 128.7, 128.4, 128.3, 128.2, 128.2, 128.1, 127.1, 126.4, 119.3, 114.5, 112.9, 110.3, 76.0, 75.3, 66.1, 60.4, 59.8.
HRMS (ESI) calculated for $C_{37}H_{35}N_2O_7$ (M+H$^+$) 619.2439, found 619.2442.

Benzyl 2-(benzyloxy)-4-(2-(benzyloxy)-4-(6-((tert-butoxycarbonyl)amino)nicotinamido)-3-methoxybenzamido)-3-methoxybenzoate (40-1)

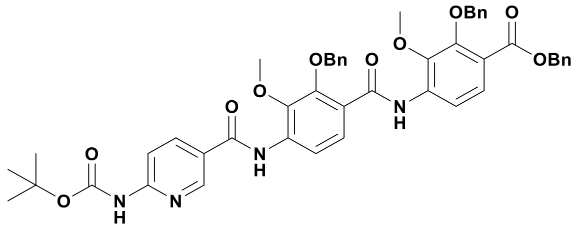

Chemical Formula: $C_{48}H_{46}N_4O_{10}$
Exact Mass: 838.3214

Oxalyl chloride (58 µL, 679 µL) as a solution in THF (3 mL) was added to a stirred solution of 6-((tert-butoxycarbonyl)amino)nicotinic acid (**31**, 162 mg, 679 µmol) and DMF (0.2 mL) in THF (10 mL) at 0°C. The reaction mixture was allowed to warm to ambient temperature over 3 hours. Next, triethylamine (158 µL, 1.13 mmol) and benzyl 4-amino-2-(benzyloxy)-3-methoxybenzoate (**40**, 350 mg, 566 µmol) as solution in THF (20 mL) were added at 0°C to the reaction mixture. The reaction mixture was allowed to warm to ambient temperature over 16 h. The solvent was removed under reduced pressure. Dichloromethane (100 mL) and water (100 mL) were added to the residue. The organic phase was washed with a saturated solution of $NaHCO_3$ (2 x 100 mL) and brine (100 mL), dried over sodium sulfate, concentrated under reduced pressure. The residue thus obtained was triturated with CH_2Cl_2 and chromatographed on silica gel with a gradient 0.4-5.0% acetone in CH_2Cl_2 with 0.1% NEt_3, in 0.4% steps to give 376 mg of a white solid (566 µmol; 79%).

^{1}H NMR (500 MHz, DMSO-d_6) δ 10.57 (s, 1H), 10.22 (s, 1H), 9.96 (s, 1H), 8.86 (d, *J* = 2.4 Hz, 1H), 8.34 – 8.25 (m, 2H), 7.96 (d, *J* = 8.8 Hz, 1H), 7.87 (d, *J* = 8.7 Hz, 1H), 7.76 (d, *J* = 8.8 Hz, 1H), 7.58 (d, *J* = 8.8 Hz, 1H), 7.47 – 7.41 (m, 4H), 7.41 – 7.30 (m, 10H), 7.30 (d, *J* = 2.6 Hz, 2H), 5.30 (s, 2H), 5.28 (s, 2H), 4.94 (s, 2H), 3.96 (s, 3H), 3.61 (s, 3H), 1.50 (s, 9H).
^{13}C NMR (126 MHz, DMSO) δ 164.6, 164.0, 162.4, 154.9, 152.5, 151.0, 149.8, 148.1, 145.0, 142.6, 137.8, 136.7, 136.6, 136.2, 136.0, 135.6, 128.8, 128.5, 128.4, 128.3, 128.2, 128.2, 128.1, 128.0, 126.3, 125.3, 124.1, 123.5, 120.3, 119.4, 115.0, 111.1, 80.1, 76.2, 75.3, 66.2, 61.0, 60.6, 27.9.
HRMS (ESI) calculated for $C_{48}H_{47}N_4O_{10}$ (M+H$^+$) 839.3287, found 839.3282.

4-(4-(6-Aminonicotinamido)-2-(benzyloxy)-3-methoxybenzamido)-2-(benzyloxy)-3-methoxybenzoic acid (40-2)

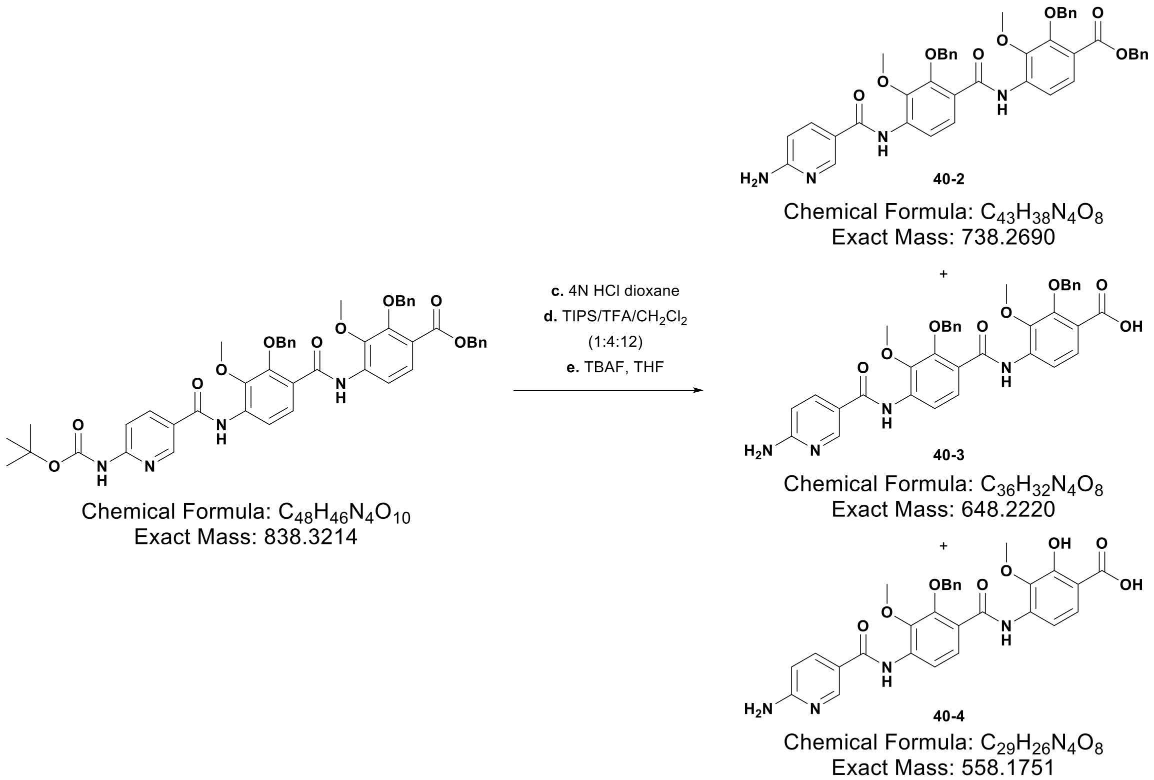

Benzyl 2-(benzyloxy)-4-(2-(benzyloxy)-4-(6-((tert-butoxycarbonyl)amino)nicotinamido)-3-methoxybenzamido)-3-methoxybenzoate (**40-1**, 7.5 mg, 8.94 µmol) dissolved in **a.**, **b.** or **c.** and the reaction mixtures were analyzed by LC-MS.

- **c.** mixture of 4N HCl in dioxane and acetonitrile (4 mL; 1:1) and stirred at 25°C for 16 h. LC-MS showed conversion to **40-3** (63%) and **40-4** (37%)
- **d.** a mixture of TIPS, TFA and CH_2Cl_2 (4 mL, 1:4:12) at 0°C and stirred for 2 h at 25°C. LC-MS showed conversion to **40-2** (5%), **40-3** (26%) and **40-4** (69%).
- **e.** dry THF (2 mL) and tetrabutylammonium fluoride (89.4 µmol, 1 M solution in THF) and stirred at 70°C for 3 h. LC-MS showed solely conversion to **40-3**.

c. HPLC-MS

Number of detected peaks: 2

Apex RT	Start RT	End RT	Area	%Area	Found HRMS (ESI)	compounds	Calculated
8.12	8.00	8.35	113800.355	36.90	559.1824	**40-4**	559.1823
9.44	9.33	9.72	194624.533	63.10	649.2288	**40-3**	649.2293

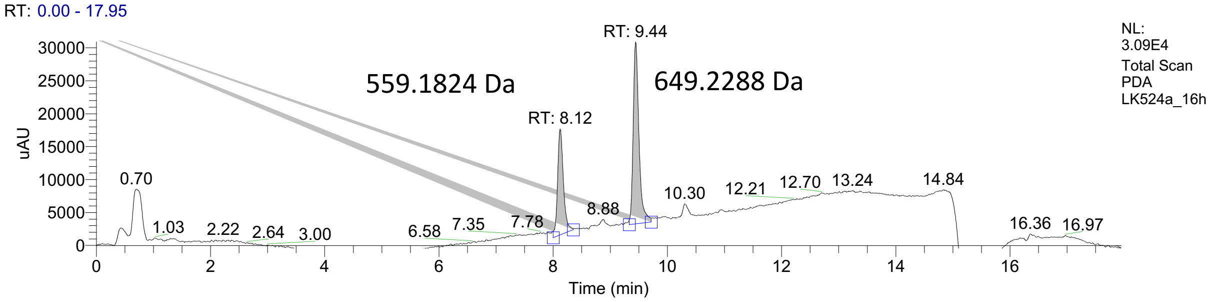

d. HPCL-MS

Number of detected peaks: 3

Apex RT	Start RT	End RT	Area	%Area	Found HRMS (ESI)	compounds	Calculated
8.15	8.03	8.30	29948.925	25.53	559.1824	**40-4**	559.1823
8.71	8.57	8.85	80868.419	68.95	649.2288	**40-3**	649.2293
8.91	8.87	9.01	6475.005	5.52	739.2769	**40-2**	739.2762

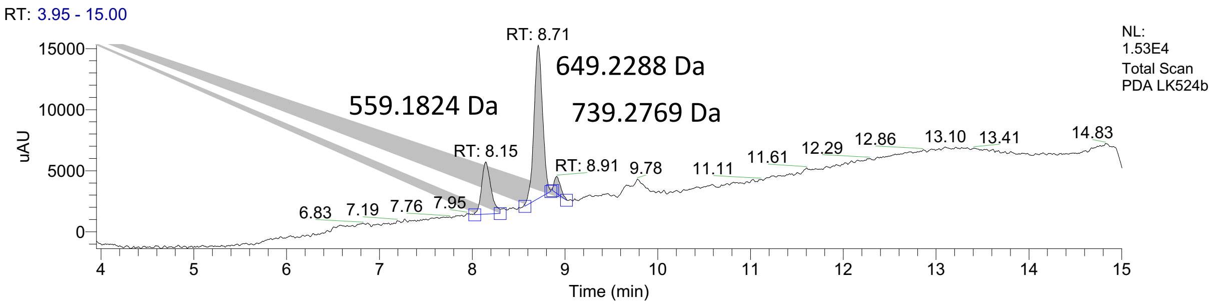

e. HPLC - MS

Number of detected peaks: 1

Apex RT	Start RT	End RT	Area	%Area	Found HRMS (ESI)	compounds	Calculated
7.60	7.41	7.82	122780.088	100.00	649.2288	**40-3**	649.2293

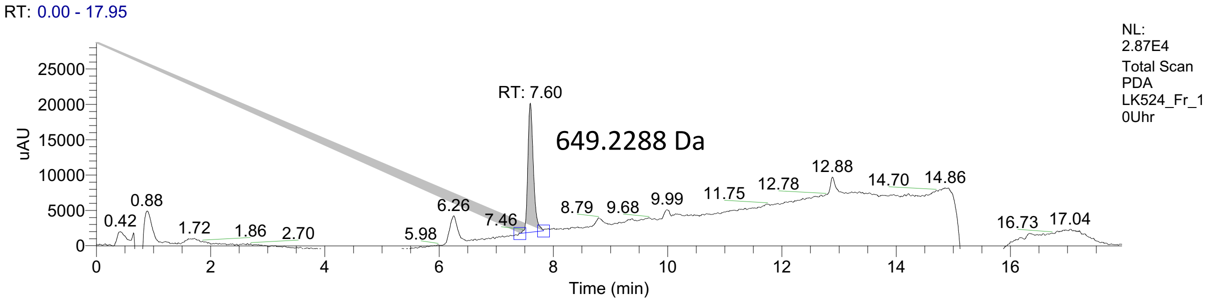

6-(((Trichloromethoxy)carbonyl)amino)nicotinic acid (41-1)

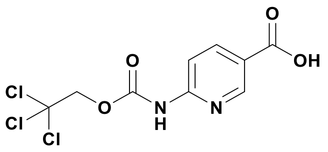

Chemical Formula: $C_9H_7Cl_3N_2O_4$
Exact Mass: 311.9471

To a solution of 6-aminonicotinic acid (**41**, 500 mg, 3.62 mmol) and pyridine (641 µL, 7.96 mmol) in THF (10 mL) was added 2,2,2-trichloroethyl chloroformate (1.10 mL, 7.96 mmol) at 0°C. The reaction mixture was stirred for 1.5 h at 0°C, then quenched with water and extracted with EtOAc (3x). The combined organic layers were washed with water, dried over Na_2SO_4, filtered, and concentrated under reduced pressure. The residue was purified by column chromatography (2-50% EtOAc in cyclohexane) to give 678 mg of a white solid (2.16 mmol, 60%).

^{1}H NMR (500 MHz, DMSO-d_6) δ 13.14 (s, 1H), 11.11 (s, 1H), 8.81 (dd, *J* = 2.3, 0.8 Hz, 1H), 8.27 (dd, *J* = 8.7, 2.3 Hz, 1H), 7.92 (dd, *J* = 8.8, 0.8 Hz, 1H), 4.99 (s, 2H).
^{13}C NMR (126 MHz, DMSO) δ 165.9, 154.8, 151.9, 149.7, 139.5, 121.8, 111.8, 95.6, 73.5.
HRMS (ESI) calculated for $C_9H_8Cl_3N_2O_4$ (M+H$^+$) 312.9544, found 312.9539.

Allyl 2-(allyloxy)-4-(2-(allyloxy)-4-amino-3-methoxybenzamido)-3-methoxybenzoate (35b)[10]

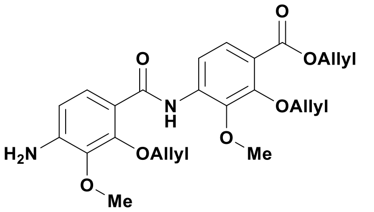

Chemical Formula: $C_{25}H_{28}N_2O_7$
Exact Mass: 468,1897

Synthesized according to exp. procedure reported.

^{1}H NMR (400 MHz, DMSO-d_6) δ 10.62 (s, 1H), 8.35 (d, J = 8.8 Hz, 1H), 7.56 (dd, J = 20.3, 8.8 Hz, 2H), 6.57 (d, J = 8.8 Hz, 1H), 6.18 – 5.96 (m, 3H), 5.85 (s, 2H), 5.39 (ddq, J = 17.2, 11.2, 1.6 Hz, 3H), 5.25 (ddq, J = 14.8, 10.4, 1.4 Hz, 3H), 4.81 – 4.72 (m, 4H), 4.53 (dt, J = 5.8, 1.4 Hz, 2H), 3.90 (s, 3H), 3.75 (s, 3H).
^{13}C NMR (101 MHz, DMSO) δ 164.4, 163.0, 151.1, 150.6, 147.5, 142.0, 137.6, 137.3, 134.0, 133.1, 132.7, 127.0, 126.3, 119.6, 119.3, 118.0, 117.7, 114.4, 112.7, 110.2, 74.8, 74.5, 65.0, 60.8, 59.6.
HRMS (ESI) calculated for $C_{25}H_{29}N_2O_7$ (M+H$^+$) 469.1969, found 469.1960.

Allyl 2-(allyloxy)-4-(2-(allyloxy)-3-methoxy-4-(6-(((2,2,2-trichloroethoxy)carbonyl)amino)nicotinamido)benzamido)-3-methoxybenzoate (41-2)

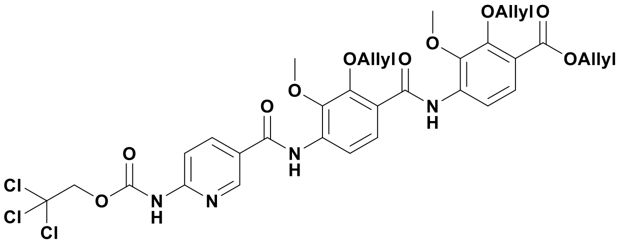

Chemical Formula: $C_{34}H_{33}Cl_3N_4O_{10}$
Exact Mass: 762.1262

6-(((Trichloromethoxy)carbonyl)amino)nicotinic acid (**41-1**, 1.20 g, 3.84 mmol) was suspended in $SOCl_2$ (2.58 mL, 38.4 mmol) and refluxed for 3 h at 90°C. Then, the $SOCl_2$ was evaporated under reduced pressure and the resulted acid chloride was used without further purification. The residue was dissolved in dry THF (3 ml) and added to a solution of allyl 2-(allyloxy)-4-(2-(allyloxy)-4-amino-3-methoxybenzamido)-3-methoxybenzoate (**35b**, 600 mg, 1.28 mmol) and NEt_3 (0.357 mL, 2.56 mmol) in dry THF (3 ml) at 0℃. The reaction mixture was kept under N_2 atmosphere, allowed to reach room temperature, and stirred at room temperature overnight. The reaction mixture was quenched with 1N HCl (5 ml) and extracted with three portions of EtOAc (20 ml). The combined organic layers were washed with a saturated solution of $NaHCO_3$ (20 ml), brine (20 ml) and concentrated under reduced pressure. The crude mixture was purified on a silica column (product eluted at 1:5 EtOAc : cyclohexane) to give 911 mg of a grey solid (1.19 mmol, 93%).

HRMS (ESI) calculated for $C_{34}H_{34}Cl_3N_4O_{10}$ (M+H$^+$) 763.1335, found 763.1334.

Allyl 2-(allyloxy)-4-(2-(allyloxy)-4-(6-aminonicotinamido)-3-methoxybenzamido)-3-methoxybenzoate (41-3)

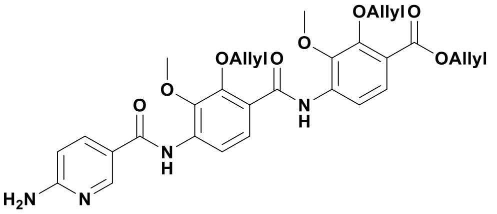

Chemical Formula: $C_{31}H_{32}N_4O_8$
Exact Mass: 588.2220

Allyl 2-(allyloxy)-4-(2-(allyloxy)-3-methoxy-4-(6-(((2,2,2-trichloroethoxy)carbonyl)amino)nicotinamido)benzamido)-3-methoxybenzoate (**41-2**, 900 mg, 1.18 mmol) was dissolved in a mixture of EtOH/EtOAc/AcOH (8:6:1, 15 mL). The mixture was cooled to 0℃ and zinc dust (1.54 g, 23.6 mmol) was added. The reaction was stirred at 0℃ for 30 minutes, filtered over celite and the residue was washed with three portions of CH_2Cl_2. The filtrate was concentrated under reduced pressure, redissolved in EtOAc (20 ml) and washed with three portions of a saturated solution of $NaHCO_3$ (10 mL) and one portion of brine (10 ml). Then, the organic phase was concentrated under reduced pressure to yield 651 mg of light-yellow solid (1.11 mmol, 94%) that was used without further purification.

^{1}H NMR (500 MHz, DMSO-d_6) δ 10.64 (s, 1H), 9.46 (s, 1H), 8.59 (d, *J* = 2.4 Hz, 1H), 8.33 (d, *J* = 8.8 Hz, 1H), 7.96 – 7.87 (m, 2H), 7.78 (d, *J* = 8.8 Hz, 1H), 7.57 (d, *J* = 8.9 Hz, 1H), 6.70 (s, 2H), 6.50 (d, *J* = 8.8 Hz, 1H), 6.07 (ddt, *J* = 33.6, 16.9, 5.5 Hz, 3H), 5.45 – 5.36 (m, 3H), 5.29 (dd, *J* = 10.5, 4.9 Hz, 2H), 5.24 (d, *J* = 10.3 Hz, 1H), 4.78 (dd, *J* = 12.4, 5.9 Hz, 4H), 4.54 (d, *J* = 5.7 Hz, 2H), 3.93 (s, 3H), 3.90 (s, 3H).
HRMS (ESI) calculated for $C_{31}H_{33}N_4O_8$ (M+H$^+$) 589.2293, found 589.2288.

Allyl (S)-2-(allyloxy)-4-(2-(allyloxy)-4-(6-(2-((tert-butoxycarbonyl)amino)-3-(1-((pivaloyloxy)methyl)-1H-1,2,3-triazol-4-yl)propanamido)nicotinamido)-3-methoxybenzamido)-3-methoxybenzoate (41-4)

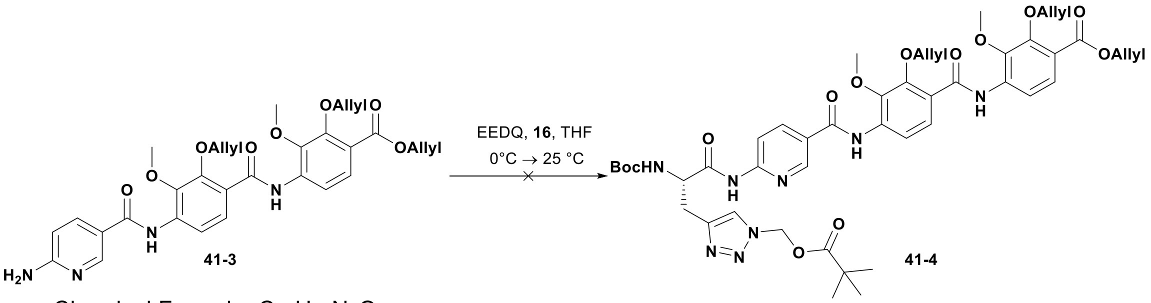

Chemical Formula: $C_{31}H_{32}N_4O_8$
Exact Mass: 588.2220

Chemical Formula: $C_{47}H_{56}N_8O_{13}$
Exact Mass: 940.3967

To a solution of EEDQ (818 mg, 3.31 mmol) in THF (10 mL) was added (S)-2-((tert-butoxycarbonyl)amino)-3-(1-((pivaloyloxy)methyl)-1H-1,2,3-triazol-4-yl)propanoic acid (**38**, 819 mg, 2.21 mmol) at 0°C and stirred for 15 min. Next a solution of benzyl 4-(6-(4-aminobenzamido)nicotinamido)-2-(benzyloxy)-3-methoxybenzoate (**41-3**, 650 mg, 1.10 mmol) in THF (5 mL) was added at 0°C, the reaction mixture was allowed to warm to ambient temperature and stirred for 2 d. The conversion was monitored by LC-MS. A portion of EEDQ was added after 2 d and after 5 d (EEDQ, each time: 818 mg, 3.31 mmol) and (S)-2-((tert-butoxycarbonyl)amino)-3-(1-((pivaloyloxy)methyl)-1H-1,2,3-triazol-4-yl)propanoic acid (**38**, each time: 819 mg, 2.21 mmol). All volatiles were removed under reduced pressure and the residue was dissolved in dichloromethane, the organic phase was washed with HCl 1N (50 mL), a saturated solution of $NaHCO_3$ (50 mL) and brine

(50 mL), dried over sodium sulfate and evaporated. The residue was purified by column chromatography on silica gel (eluent: 1→3% MeOH in dichloromethane), to give 512 mg of **41-3** (s.m., 870 µmol, 79%) as a light-yellow solid back, the desired product could not be isolated.

Number of detected peaks: 2

Apex RT	Start RT	End RT	Area	%Area	Found HRMS (ESI)	compounds	Calculated
8.46	8.28	8.76	555596.951	78.41	589.2296	**41-3**	589.2293
11.64	11.48	11.92	152967.313	21.59	941.4049	**41-4**	941.4040

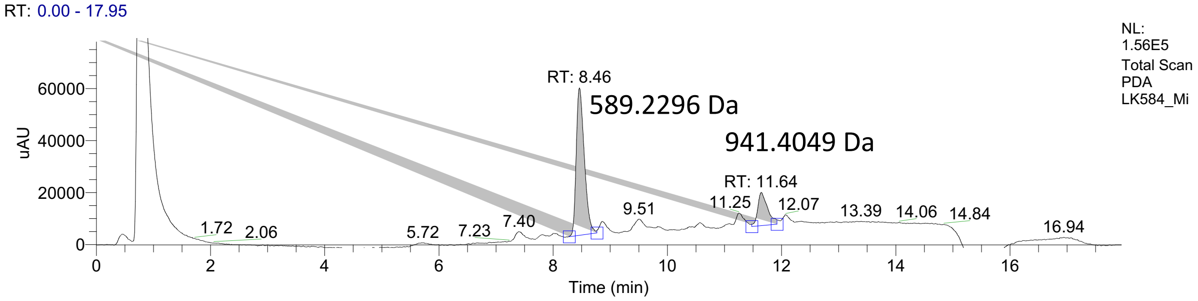

Benzyl 2-(benzyloxy)-4-(2-(benzyloxy)-3-methoxy-4-(6-(((trichloromethoxy)carbonyl)amino)nicotinamido)benzamido)-3-methoxybenzoate (42)

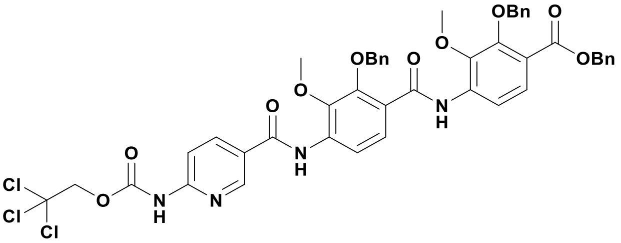

Chemical Formula: $C_{46}H_{39}Cl_3N_4O_{10}$
Exact Mass: 912.1732

6-(((Trichloromethoxy)carbonyl)amino)nicotinic acid (**41-1**, 293 mg, 937 µmol) was suspended in $SOCl_2$ (566 µL, 7.81 mmol) and refluxed for 12 h at 80°C. Then, the $SOCl_2$ was evaporated under reduced pressure and the resulted acid chloride was used without further purification. The residue was dissolved in dry THF (3 ml) and added to a solution of benzyl 4-(4-amino-2-(benzyloxy)-3-methoxybenzamido)-2-(benzyloxy)-3-methoxybenzoate (**40**, 483 mg, 781 µmol) and NEt_3 (218 µL, 1.56 mmol) in dry THF (3 ml) at 0℃. The reaction mixture was kept under N_2 atmosphere, allowed to reach room temperature, and stirred at room temperature overnight. The reaction mixture was quenched with 1N HCl (5 ml) and extracted with three portions of EtOAc (20 ml). The combined organic layers were washed with a saturated solution of $NaHCO_3$ (20 ml), brine (20 ml) and concentrated under reduced pressure. The crude mixture was purified on a silica column (0.8% acetone, 0.4% MeOH in CH_2Cl_2) to give 402 mg of a grey solid (440 µmol, 56%).

HRMS (ESI) calculated for $C_{46}H_{40}Cl_3N_4O_{10}$ $(M+H^+)$ 913.1805, found 913.1796.

Benzyl 4-(4-(6-aminonicotinamido)-2-(benzyloxy)-3-methoxybenzamido)-2-(benzyloxy)-3-methoxybenzoate (42-1)

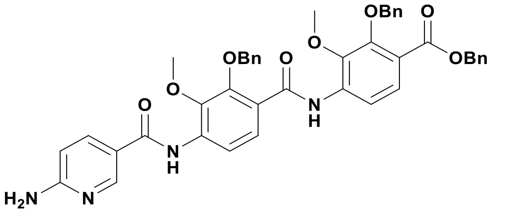

Chemical Formula: $C_{43}H_{38}N_4O_8$
Exact Mass: 738.2690

Benzyl 2-(benzyloxy)-4-(2-(benzyloxy)-3-methoxy-4-(6-(((trichloromethoxy)carbonyl)amino)nicotinamido)benzamido)-3-methoxybenzoate (**42**, 387 mg, 423 µmol) was dissolved in a mixture of EtOH/EtOAc/AcOH (8:6:1, 75 mL). The mixture was cooled to 0℃ and Zn powder (553 mg, 8.47 mmol) was added. The reaction was stirred at 0℃ for 30 minutes, filtered over celite and the residue was washed with three portions of CH_2Cl_2. The filtrate was concentrated under reduced pressure, redissolved in EtOAc (20 mL) and washed with three portions of $NaHCO_3$ saturated solution (10 mL) and one portion of brine (10 mL). Then, the organic phase was concentrated under reduced pressure to yield 303 mg of light-yellow solid (410 µmol, 97%) that was used without further purification.

^{1}H NMR (500 MHz, DMSO-d_6) δ 10.56 (s, 1H), 9.51 (s, 1H), 8.61 (d, *J* = 2.4 Hz, 1H), 8.27 (d, *J* = 8.8 Hz, 1H), 7.96 – 7.90 (m, 1H), 7.74 (d, *J* = 8.8 Hz, 1H), 7.58 (d, *J* = 8.7 Hz, 1H), 7.47 – 7.42 (m, 4H), 7.39 – 7.29 (m, 11H), 6.70 (s, 1H), 6.51 (d, *J* = 8.8 Hz, 1H), 5.29 (d, *J* = 11.6 Hz, 4H), 4.94 (s, 2H), 3.95 (s, 3H), 3.60 (s, 3H).
HRMS (ESI) calculated for $C_{43}H_{39}N_4O_8$ (M+H$^+$) 739.2762, found 739.2757.

Benzyl (S)-2-(benzyloxy)-4-(2-(benzyloxy)-4-(6-(2-((tert-butoxycarbonyl)amino)-3-(1-((pivaloyloxy)methyl)-1H-1,2,3-triazol-4-yl)propanamido)nicotinamido)-3-methoxybenzamido)-3-methoxybenzoate (42-2)

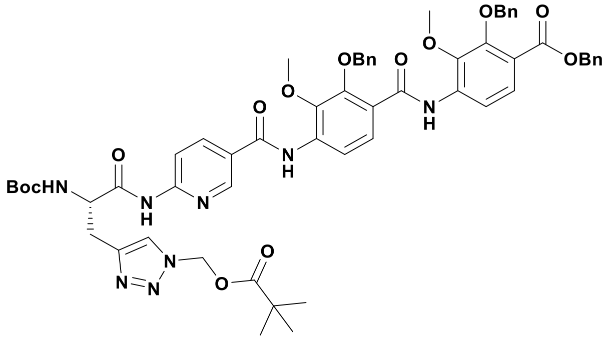

Chemical Formula: $C_{59}H_{62}N_8O_{13}$
Exact Mass: 1090.4436

To a solution of EEDQ (301 mg, 1.22 mmol) in THF (10 mL) was added (S)-2-((tert-butoxycarbonyl)amino)-3-(1-((pivaloyloxy)methyl)-1H-1,2,3-triazol-4-yl)propanoic acid (**38**, 301 mg, 812 mmol) at 0°C and stirred for 15 min. Next a solution of benzyl 4-(6-(4-aminobenzamido)nicotinamido)-2-(benzyloxy)-3-methoxybenzoate (**42-1**, 300 mg, 406 µmol) in THF (5 mL) was added at 0°C, the reaction mixture was allowed to warm to ambient temperature and stirred for 9 d. All volatiles were removed under reduced pressure and the residue was dissolved in dichloromethane, the organic phase was washed with HCl 1 N (50 mL), a saturated solution of $NaHCO_3$ (50 mL) and brine (50 mL), dried over sodium sulfate and evaporated. The residue was purified by

column chromatography on silica gel (eluent: 1→3% MeOH in dichloromethane) to give 152 mg of a colorless solid (139 µmol, 34%).

^{1}H NMR (500 MHz, DMSO-d_6) δ 10.56 (s, 1H), 9.51 (s, 1H), 8.61 (d, *J* = 2.5 Hz, 1H), 8.27 (d, *J* = 8.8 Hz, 1H), 7.97 – 7.88 (m, 3H), 7.73 (d, *J* = 8.8 Hz, 1H), 7.58 (d, *J* = 8.8 Hz, 1H), 7.47 – 7.36 (m, 9H), 7.36 (d, *J* = 3.4 Hz, 8H), 7.33 – 7.28 (m, 3H), 7.08 (d, *J* = 8.4 Hz, 1H), 6.69 (s, 2H), 6.51 (d, *J* = 8.8 Hz, 1H), 6.26 (d, *J* = 15.1 Hz, 3H), 5.29 (d, *J* = 11.0 Hz, 4H), 4.94 (s, 2H), 4.14 (d, *J* = 12.0 Hz, 1H), 3.95 (s, 3H), 3.60 (s, 3H), 3.07 (d, *J* = 4.7 Hz, 1H), 2.99 – 2.92 (m, 1H), 1.34 (s, 9H), 1.11 (s, 14H).
^{13}C NMR (126 MHz, DMSO) δ 155.3, 151.1, 149.3, 144.3, 143.7, 142.6, 136.8, 136.7, 136.1, 135.6, 128.9, 128.5, 128.5, 128.3, 128.3, 128.3, 128.2, 128.1, 128.0, 126.4, 125.4, 124.0, 122.7, 120.6, 120.2, 118.7, 117.5, 114.9, 106.9, 78.1, 76.2, 75.3, 69.8, 66.2, 61.0, 60.6, 53.3, 38.2, 28.1, 27.0, 26.5.
HRMS (ESI) calculated for $C_{59}H_{63}N_8O_{13}$ (M+H$^+$) 1091.4509, found 1091.4520.

(S)-4-(4-(6-(2-((tert-butoxycarbonyl)amino)-3-(1-((pivaloyloxy)methyl)-1H-1,2,3-triazol-4-yl)propanamido)nicotinamido)-2-hydroxy-3-methoxybenzamido)-2-hydroxy-3-methoxybenzoic acid (42-3)

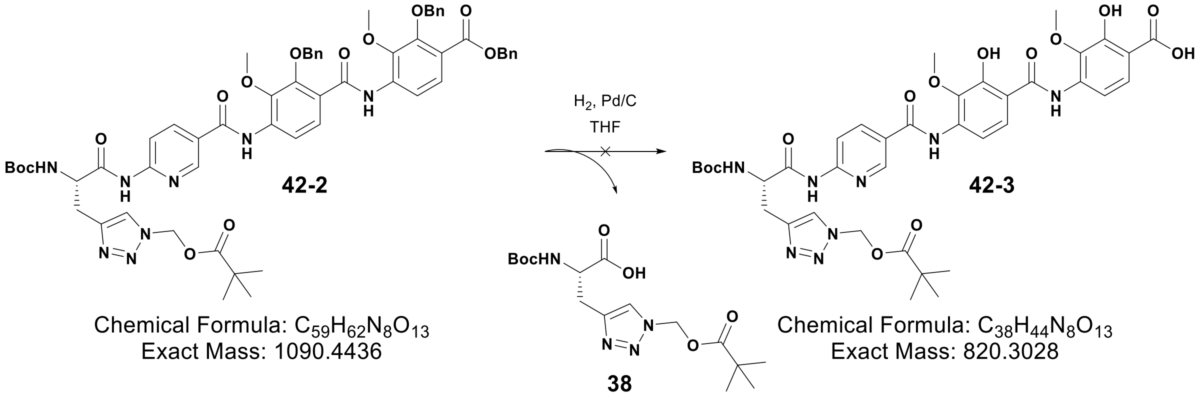

Chemical Formula: $C_{59}H_{62}N_8O_{13}$
Exact Mass: 1090.4436

Chemical Formula: $C_{38}H_{44}N_8O_{13}$
Exact Mass: 820.3028

Chemical Formula: $C_{16}H_{26}N_4O_6$
Exact Mass: 370.1852

A mixture of benzyl (S)-2-(benzyloxy)-4-(2-(benzyloxy)-4-(6-(2-((tert-butoxycarbonyl)amino)-3-(1-((pivaloyloxy)methyl)-1H-1,2,3-triazol-4-yl)propanamido) nicotinamido)-3-methoxybenzamido)-3-methoxybenzoate (**42-2**, 150 mg, 137 µmol) and 10% Pd/C (15 mg), in 1:1:1 mixture of EtOAc/THF (30 mL), was stirred for 12 h under H_2 atmosphere. After the catalyst was filtered and washed with MeOH, the filtrate was evaporated. The desired product was not observed, instead three not assignable peaks were found in the LC-MS and one peak was identified as the C fragment (**38**).

Number of detected peaks: 4

Apex RT	Start RT	End RT	Area	%Area	Found HRMS (ESI)	compounds	Calculated
7.29	7.15	7.52	57697.543	37.75	371.1921	**38**	371.1925
7.68	7.53	7.88	18612.987	12.18	413.2028	**n.i.**[1]	-
8.32	8.17	8.60	67720.247	44.31	559.1807	**n.i.**	-
8.70	8.63	8.88	8811.456	5.77	649.2277	**n.i.**	-

[1] Not identified

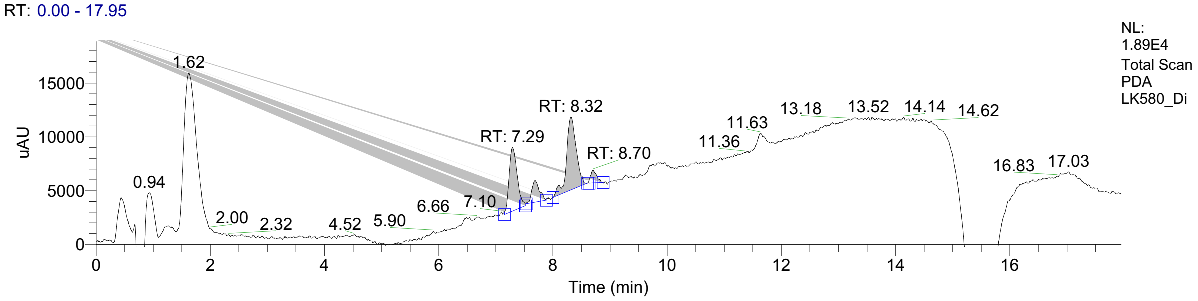
RT: 0.00 - 17.95
NL:
1.89E4
Total Scan
PDA
LK580_Di
uAU
Time (min)
1.62
0.94
2.00
2.32
4.52
5.90
6.66
7.10
RT: 7.29
RT: 8.32
RT: 8.70
11.36
11.63
13.18
13.52
14.14
14.62
16.83
17.03

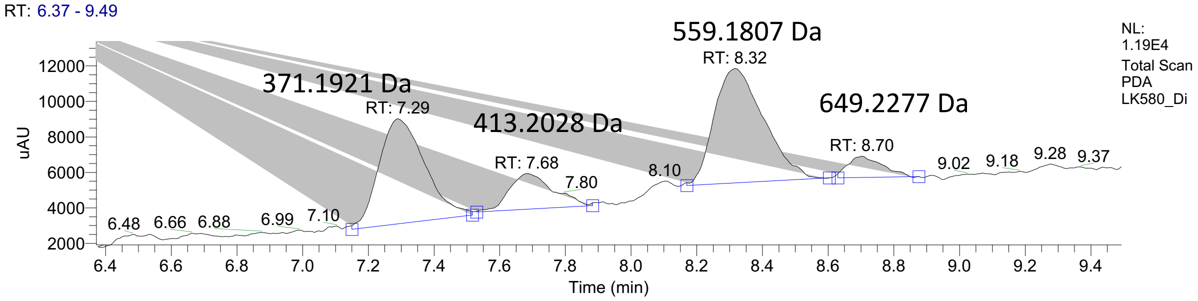
RT: 6.37 - 9.49
NL:
1.19E4
Total Scan
PDA
LK580_Di
559.1807 Da
371.1921 Da
413.2028 Da
649.2277 Da
RT: 7.29
RT: 7.68
RT: 8.32
RT: 8.70
6.48
6.66
6.88
6.99
7.10
7.80
8.10
9.02
9.18
9.28
9.37
uAU
Time (min)

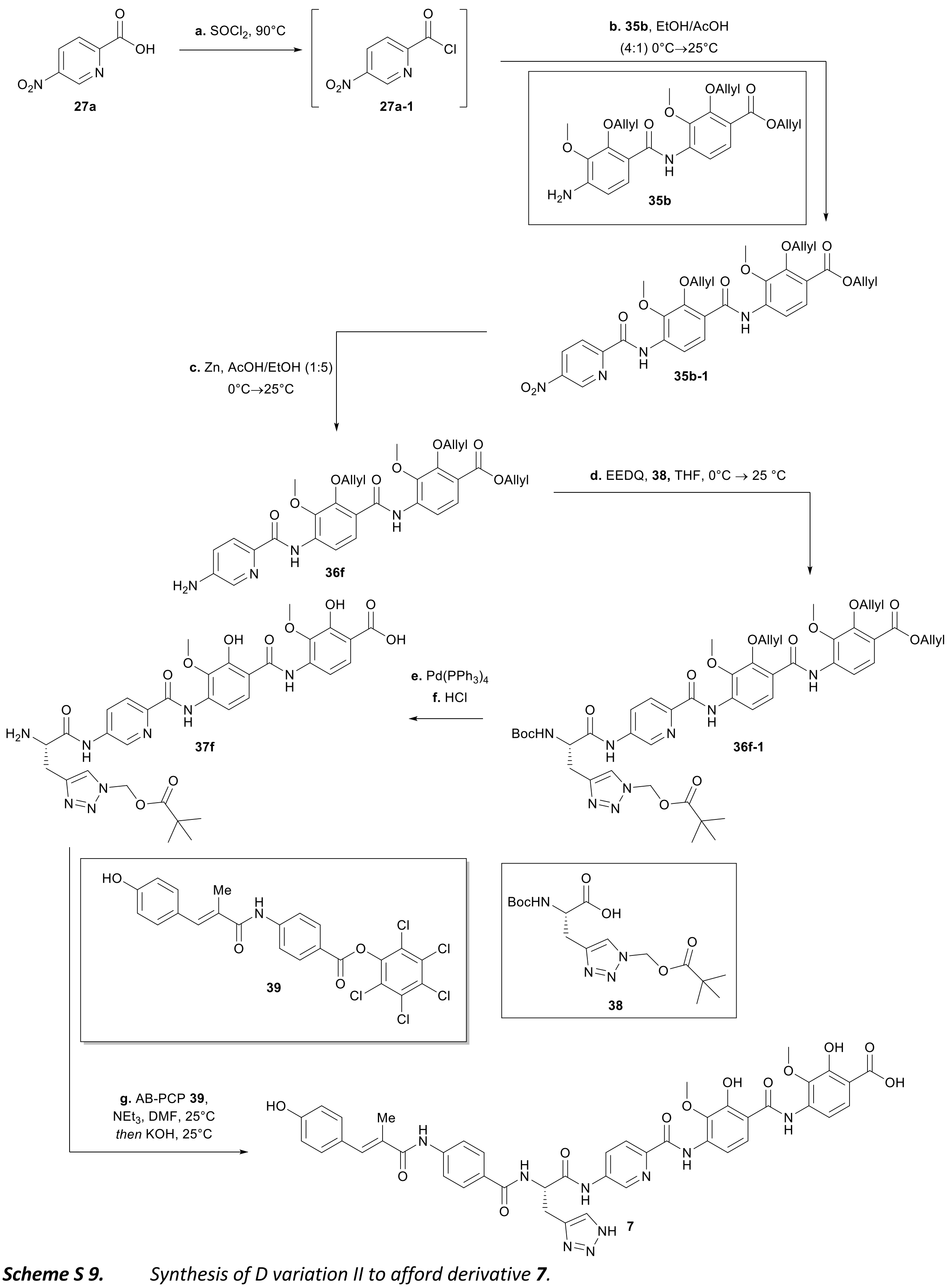

Scheme S 9. *Synthesis of D variation II to afford derivative **7**.*

Allyl 2-(allyloxy)-4-(2-(allyloxy)-3-methoxy-4-(5-nitropicolinamido)benzamido)-3-methoxybenzoate (35b-1)

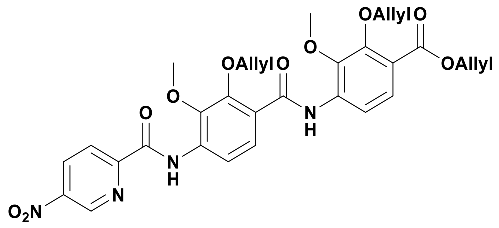

Chemical Formula: $C_{31}H_{30}N_4O_{10}$
Exact Mass: 618,1962

Para-nitro pyridine-2 carboxylic acid (**27a**, 1.08 mg, 6.40 mmol) was put in a 50 ml round bottom flask equipped with a condenser. $SOCl_2$ (6.19 mL, 85.4 mmol) were added, and the reaction mixture was stirred at 90°C for 2 h. Then, the $SOCl_2$ was evaporated under reduced pressure and the resulted acid chloride was used without further purification. The residue was dissolved in dry THF (3 ml) and added to a solution of allyl 2-(allyloxy)-4-(2-(allyloxy)-4-amino-3-methoxybenzamido)-3-methoxybenzoate (**35b**, 2.00 g, 4.27 mmol) and DIPEA (1.49 mL, 8.54 mmol) in dry THF (3 ml) at 0℃. The reaction mixture was kept under N_2 atmosphere, allowed to reach room temperature, and stirred at room temperature overnight. Then, the reaction mixture was quenched with 1N HCl (20 ml) and washed with three portions EtOAc (30 ml). The combined organic fractions were washed with a saturated solution of $NaHCO_3$ (30 mL). The organic phase was dried over Na_2SO_4, filtered, and concentrated under reduced pressure. The crude mixture was purified on a silica column 20% to 60% EtOAc in hexane. The product eluted at 60% EtOAc in hexane to yield 2.30 g (3.72 mmol, 87%).

^{1}H NMR (500 MHz, DMSO-d_6) δ 10.62 (d, *J* = 2.8 Hz, 2H), 9.56 – 9.51 (m, 1H), 8.87 (dd, *J* = 8.6, 2.6 Hz, 1H), 8.44 (d, *J* = 8.6 Hz, 1H), 8.33 (dd, *J* = 18.8, 8.8 Hz, 2H), 7.88 (d, *J* = 8.8 Hz, 1H), 7.56 (d, *J* = 8.8 Hz, 1H), 6.18 – 5.98 (m, 3H), 5.43 (q, *J* = 1.6 Hz, 1H), 5.43 – 5.34 (m, 2H), 5.29 (ddd, *J* = 10.5, 9.0, 1.5 Hz, 3H), 5.25 (dt, *J* = 10.4, 1.6 Hz, 2H), 4.84 (dt, *J* = 6.3, 1.2 Hz, 2H), 4.77 (dt, *J* = 5.5, 1.5 Hz, 2H), 4.54 (dt, *J* = 5.7, 1.4 Hz, 2H), 4.01 (s, 3H), 3.94 (s, 3H).
HRMS (ESI) calculated for $C_{31}H_{31}N_4O_{10}$ ($M+H^+$) 619.2035, found 619.2043.

Allyl 2-(allyloxy)-4-(2-(allyloxy)-4-(5-aminopicolinamido)-3-methoxybenzamido)-3-methoxybenzoate (36f)

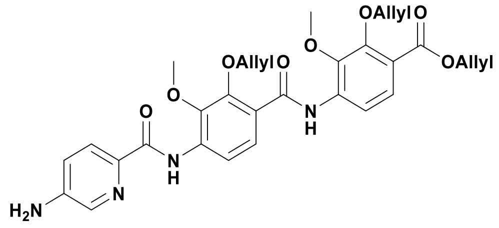

Chemical Formula: $C_{31}H_{32}N_4O_8$
Exact Mass: 588,2220

Allyl 2-(allyloxy)-4-(2-(allyloxy)-3-methoxy-4-(5-nitropicolinamido)benzamido)-3-methoxybenzoate (**35b-1**, 2.30 g, 3.72 mmol) was dissolved in a mixture of EtOH (16 mL) and AcOH (4 mL). The mixture was cooled to 0℃ and zinc dust (4.86 g, 74.4 mmol) was added. The reaction was stirred at 0℃ for 30 minutes, filtered over celite® and the residue was washed with three portions of CH_2Cl_2. The filtrate was concentrated under reduced pressure, redissolved in EtOAc (20 mL) and washed with three portions of a saturated solution of $NaHCO_3$ (10 mL) and one portion of brine (10 m). Then, the organic phase was concentrated under reduced pressure to yield 1.87 g of a brown solid (3.18 mmol, 86%) that was used without further purification.

^{1}H NMR (400 MHz, DMSO-d_6) δ 10.65 (s, 1H), 10.45 (s, 1H), 8.36 (dd, *J* = 23.4, 8.8 Hz, 2H), 8.01 (d, *J* = 2.6 Hz, 1H), 7.86 (dd, *J* = 13.0, 8.7 Hz, 2H), 7.56 (d, *J* = 8.8 Hz, 1H), 7.06 (dd, *J* = 8.6, 2.6 Hz, 1H), 6.28 (s, 2H), 6.19 – 5.96 (m, 3H), 5.40 (ddq, *J* = 17.2, 10.4, 1.6 Hz, 3H), 5.33 – 5.20 (m, 3H), 4.87 – 4.80 (m, 2H), 4.76 (dt, *J* = 5.6, 1.5 Hz, 2H), 4.54 (dt, *J* = 5.9, 1.4 Hz, 2H), 3.97 (s, 3H), 3.93 (s, 3H).
^{13}C NMR (101 MHz, DMSO) δ 164.4, 162.4, 162.3, 151.1, 149.3, 148.5, 142.4, 141.3, 136.6, 136.6, 136.0, 134.6, 134.0, 132.6, 126.6, 126.3, 123.7, 120.9, 120.4, 120.1, 119.3, 118.1, 117.8, 114.7, 114.0, 75.2, 74.6, 65.1, 61.0.
HRMS (ESI) calculated for $C_{31}H_{33}N_4O_8$ (M+H$^+$) 589.2297, found 589.2293.

Allyl (S)-2-(allyloxy)-4-(2-(allyloxy)-4-(5-(2-((tert-butoxycarbonyl)amino)-3-(1-((pivaloyloxy)methyl)-1H-1,2,3-triazol-4-yl)propanamido)picolinamido)-3-methoxybenzamido)-3-methoxybenzoate (36f-1)

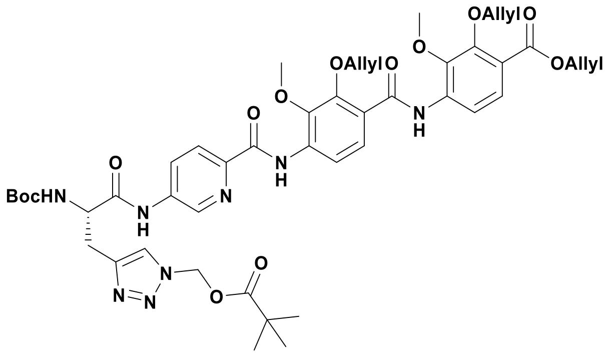

Chemical Formula: $C_{47}H_{56}N_8O_{13}$
Exact Mass: 940,3967

To a solution of EEDQ (1.05 g. 4.25 mmol) in THF (10 mL) was added (S)-2-((tert-butoxycarbonyl)amino)-3-(1-((pivaloyloxy)methyl)-1H-1,2,3-triazol-4-yl)propanoic acid (**38**, 1.26 g, 3.40 mmol) at 0°C and stirred for 15 min. Next a solution of allyl 2-(allyloxy)-4-(2-(allyloxy)-4-(5-aminopicolinamido)-3-methoxybenzamido)-3-methoxybenzoate (**36f**, 1.00 g, 1.70 mmol) in THF (5 mL) was added at 0°C, the reaction mixture was allowed to warm to ambient temperature and stirred for 18 h. All volatiles were removed under reduced pressure and the residue was dissolved in dichloromethane, the organic phase was washed with HCl 1 N (50 mL), a saturated solution of $NaHCO_3$ (50 mL) and brine (50 mL), dried over sodium sulfate and evaporated. The residue was purified by column chromatography on silica gel (eluent: 0→20% Acetone in CH_2Cl_2) to give 1.10 g of a yellowish solid (1.17 mmol, 69%).

^{1}H NMR (400 MHz, DMSO-d_6) δ 10.76 (s, 1H), 10.65 (s, 1H), 10.58 (s, 1H), 8.92 (d, *J* = 2.4 Hz, 1H), 8.38 (d, *J* = 8.8 Hz, 1H), 8.36 – 8.28 (m, 2H), 8.18 (d, *J* = 8.7 Hz, 1H), 8.00 (s, 1H), 7.87 (d, *J* = 8.8 Hz, 1H), 7.56 (d, *J* = 8.8 Hz, 1H), 7.32 (d, *J* = 7.8 Hz, 1H), 6.29 (s, 2H), 6.20 – 5.96 (m, 3H), 5.40 (ddq, *J* = 17.2, 9.5, 1.6 Hz, 3H), 5.34 – 5.20 (m, 3H), 4.84 (d, *J* = 6.3 Hz, 2H), 4.76 (dt, *J* = 5.4, 1.5 Hz, 2H), 4.54 (dt, *J* = 5.7, 1.5 Hz, 2H), 4.43 (q, *J* = 7.8 Hz, 1H), 4.00 (s, 3H), 3.94 (s, 3H), 3.15 (dd, *J* = 14.7, 5.4 Hz, 1H), 3.02 (dd, *J* = 14.7, 9.1 Hz, 1H), 1.36 (s, 9H), 1.08 (s, 9H).
^{13}C NMR (101 MHz, DMSO) δ 176.4, 171.4, 164.4, 162.3, 161.5, 155.4, 151.1, 149.3, 143.3, 143.2, 142.4, 141.6, 139.5, 138.8, 136.6, 136.0, 134.0, 132.6, 132.6, 127.1, 126.5, 126.3, 124.3, 123.0, 121.7, 120.4, 120.2, 118.1, 117.8, 114.8, 114.3, 78.4, 75.2, 74.6, 69.9, 65.1, 61.1, 61.0, 59.8, 55.0, 38.2, 28.1, 26.4, 20.8, 14.1.
HRMS (ESI) calculated for $C_{47}H_{57}N_8O_{13}$ (M+H$^+$) 941.4040, found 941.4059.

(S)-4-(4-(5-(2-((tert-butoxycarbonyl)amino)-3-(1-((pivaloyloxy)methyl)-1H-1,2,3-triazol-4-yl)propanamido)picolinamido)-2-hydroxy-3-methoxybenzamido)-2-hydroxy-3-methoxybenzoic acid (36f-2)

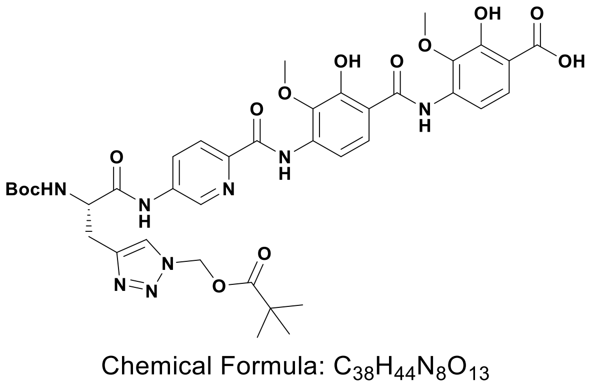

Chemical Formula: $C_{38}H_{44}N_8O_{13}$
Exact Mass: 820,3028

Allyl (S)-2-(allyloxy)-4-(2-(allyloxy)-4-(5-(2-((tert-butoxycarbonyl)amino)-3-(1-((pivaloyloxy)methyl)-1H-1,2,3-triazol-4-yl)propanamido)picolinamido)-3-methoxybenzamido)-3-methoxybenzoate (**36f-1**, 1.10 g, 1.17 mmol) and morpholine (2.02 mL, 23.4 mmol) were dissolved in dry THF (5 ml). $Pd(PPh_3)_4$ (540 mg, 468 µmol) was added. The reaction mixture was kept under N_2 atmosphere and protected from light and stirred at room temperature for 24 h. After removing all volatiles under reduced pressure, the residue was taken up in EtOAc and washed with 10% HCl(aq.) (3× 20 mL), $NaHCO_3$ saturated solution (3× 20 mL) and brine (20 mL). The organic phase was dried over Na_2SO_4, filtered, and concentrated under reduced pressure. Purification of the crude product by column chromatography (5-25% Acetone in CH_2Cl_2) gave 520 mg of the title compound (1.17 mmol, 54%) as a yellowish solid.

^{1}H NMR (400 MHz, DMSO-d_6) δ 10.90 (s, 1H), 10.82 (s, 1H), 10.48 (s, 1H), 8.94 (d, J = 2.4 Hz, 1H), 8.31 (dd, J = 8.6, 2.4 Hz, 1H), 8.19 (d, J = 8.6 Hz, 1H), 8.09 (d, J = 8.9 Hz, 1H), 8.00 (s, 1H), 7.87 (d, J = 8.9 Hz, 1H), 7.72 (d, J = 8.7 Hz, 1H), 7.50 (d, J = 8.6 Hz, 1H), 7.31 (d, J = 7.7 Hz, 1H), 6.28 (s, 2H), 4.44 (q, J = 7.5 Hz, 1H), 3.88 (s, 3H), 3.88 (s, 3H), 3.15 (d, J = 9.0 Hz, 1H), 3.03 (dd, J = 14.7, 9.0 Hz, 1H), 2.08 (s, 9H), 1.36 (s, 9H), 1.07 (s, 9H).
^{13}C NMR (101 MHz, DMSO) δ 176.4, 171.9, 171.4, 166.8, 163.8, 161.4, 155.7, 155.4, 143.3, 140.8, 139.5, 138.8, 137.3, 136.9, 135.3, 131.1, 130.6, 129.8, 127.2, 126.1, 125.0, 124.3, 123.0, 115.4, 108.8, 78.4, 69.9, 60.8, 59.7, 38.2, 30.7, 28.1, 27.7, 26.4.
HRMS (ESI) calculated for $C_{38}H_{45}N_8O_{13}$ (M+H$^+$) 819.2955, found 819.2953.

(S)-4-(4-(5-(2-amino-3-(1-((pivaloyloxy)methyl)-1H-1,2,3-triazol-4-yl)propanamido)picolinamido)-2-hydroxy-3-methoxybenzamido)-2-hydroxy-3-methoxybenzoic acid (37)

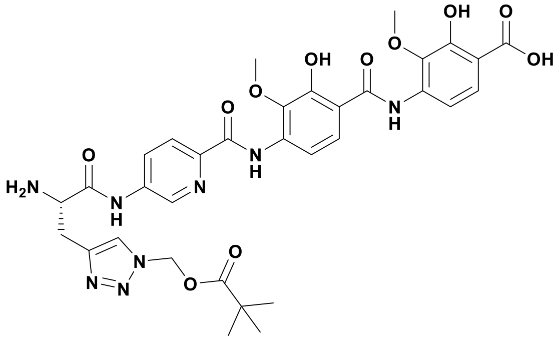

Chemical Formula: $C_{33}H_{36}N_8O_{11}$
Exact Mass: 720,2504

The Boc-protected tetrapeptide **36f-2** (500 mg, 0.69 mmol) was suspended in 4 N HCl in 1,4-dioxane (5 mL) and the reaction mixture was stirred at room temperature for 1 h. All volatiles were subsequently removed under reduced pressure and the residue was taken up in a mixture of acetonitrile and little water and freeze-dried to afford 439 mg of a yellowish powder (0.69 mmol, quant.).

^{1}H NMR (400 MHz, DMSO-d_6) δ 12.01 (s, 1H), 11.76 (s, 1H), 11.15 (s, 1H), 10.49 (s, 1H), 9.00 (d, *J* = 2.4 Hz, 1H), 8.74 (s, 2H), 8.34 (dd, *J* = 8.6, 2.4 Hz, 1H), 8.20 (t, *J* = 4.3 Hz, 2H), 8.10 (d, *J* = 8.9 Hz, 1H), 8.02 (d, *J* = 8.9 Hz, 1H), 7.88 (d, *J* = 8.9 Hz, 1H), 7.58 (d, *J* = 8.9 Hz, 1H), 6.27 (s, 2H), 4.51 (s, 1H), 3.92 (s, 3H), 3.87 (s, 3H), 3.47 (dd, *J* = 15.3, 6.1 Hz, 1H), 3.39 (dd, *J* = 15.0, 6.8 Hz, 1H), 1.05 (s, 9H).
^{13}C NMR (101 MHz, DMSO) δ 176.4, 172.0, 167.4, 163.4, 161.3, 154.4, 149.6, 143.9, 140.7, 139.6, 138.2, 137.8, 137.4, 136.3, 135.5, 131.5, 128.9, 127.4, 126.6, 125.7, 125.1, 123.0, 115.6, 110.4, 110.2, 109.1, 69.9, 60.9, 60.3, 52.6, 38.1, 27.0, 26.4.
HRMS (ESI) calculated for $C_{33}H_{37}N_8O_{11}$ (M+H$^+$) 721.2576, found 721.2581.

(S,*E*)-2-hydroxy-4-(2-hydroxy-4-(5-(2-(4-(3-(4-hydroxyphenyl)-2-methylacrylamido)benzamido)-3-(1H-1,2,3-triazol-4-yl)propanamido)picolinamido)-3-methoxybenzamido)-3-methoxybenzoic acid (7)

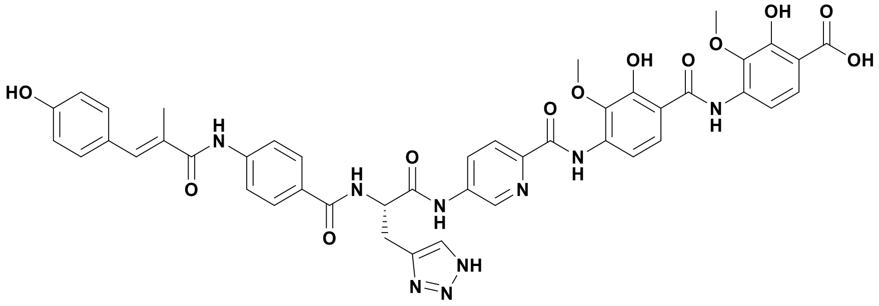

Chemical Formula: $C_{44}H_{39}N_9O_{12}$
Exact Mass: 885.2718

To a solution of tetrapeptide **37f** (50 mg, 69.4 µmol) in DMF (2 mL) was added perchlorophenyl (E)-4-(3-(4-hydroxyphenyl)-2-methylacrylamido)benzoate (**39**, 37.9 mg, 69.4 µmol) and NEt_3 (96.7 µL, 694 µmol). The reaction mixture was stirred for 2 d at 25°C and monitored by LC-MS (POM protected product: HRMS (ESI) calculated for $C_{50}H_{49}N_9O_{12}$ (M+H$^+$) 968.3573, found 968.3582). After full conversion KOH 3 N (1 mL) was added and stirred for 20 min. Next, the mixture was neutralized with HCl 6 N (0.5 mL) to a pH ~ 7. All volatiles were removed under reduced pressure, the residue was dissolved with DMSO (1 mL) and the salts were separated by centrifugation. The supernatant was loaded on the RP-HPLC system and purified under the following conditions. The collected fractions were lyophilized after their identity and purity was verified by LCMS to obtain 14.0 mg as a white solid (15.8 µmol, 23% over 2 steps).
RP-HPLC: 35-50% MeCN (+0.1% TFA) in milliQ H_2O (+0.1% TFA); 30 min; 50 mL/min.

^{1}H NMR (700 MHz, DMSO-d_6) δ 11.72 (s, 1H), 11.64 (s, 1H), 11.13 (s, 1H), 10.88 (s, 1H), 10.83 (s, 1H), 10.49 (s, 1H), 8.98 (d, *J* = 2.4 Hz, 1H), 8.83 (d, *J* = 7.6 Hz, 1H), 8.75 (d, *J* = 2.9 Hz, 1H), 8.34 (dd, *J* = 8.6, 2.4 Hz, 1H), 8.26 (dd, *J* = 8.7, 4.6 Hz, 1H), 8.20 (d, *J* = 8.5 Hz, 1H), 8.11 (d, *J* = 8.9 Hz, 1H), 8.01 (qd, *J* = 8.8, 3.1 Hz, 4H), 7.89 (dd, *J* = 22.8, 8.8 Hz, 3H), 7.67 (dt, *J* = 23.4, 1.7 Hz, 1H), 7.59 (d, *J* = 8.8 Hz, 1H), 4.93 (q, *J* = 7.5 Hz, 1H), 3.92 (s, 3H), 3.87 (s, 3H), 3.27 (dd, *J* = 14.8, 9.4 Hz, 3H), 2.54 (s, 3H).
^{13}C NMR (176 MHz, DMSO – from HSQC_ed) δ 140.0, 140.0, 137.3, 137.4, 127.7, 125.4, 127.7, 123.5, 127.0, 110.7, 110.8, 128.8, 120.0, 125.4, 130.6, 120.0, 128.8, 128.8, 127.0, 123.4, 126.1, 121.2, 54.8, 60.7, 61.3, 42.2, 43.3, 34.7.
HRMS (ESI) calculated for $C_{44}H_{40}N_9O_{12}$ (M+H$^+$) 886.2791, found 886.2786, t_R = 8.15 min.

1.3 PHYSICOCHEMICAL PROPERTIES ANALYSIS

1.3.1 Determination of kinetic solubility

20 mM stock solutions of albicidin, compounds **2** or **7** were freshly prepared in 100% DMSO. Stock solutions were diluted in *dd*H_2O or PBS (pH 7.4) to obtain final concentrations of 1 mM compound in 5% DMSO. Subsequently, samples were incubated at 20°C, 100 rpm for 20 h. After centrifugation at 15.000 rpm for 20 min, 100 µL of the supernatant was transferred to a 96-well plate and analyzed via UV spectrometry at appropriate absorbance maxima. Quantification of dissolved compound in the supernatants was performed by an external calibration curve of standard solutions in the range of 1 mM to 0.0010 mM.

1.3.2 Determination of logD values

A 10 mM stock solution of compounds **2** or **7** were prepared in DMSO. From the stock solution was added 10 µL to a mixture (1 mL, 1:1) of octanol and buffer (pH 7.4, 25 mM citric acid, 25 mM HCl, 25 mM phosphoric acid, 20 mM NaCl, 30 mM boric acid and 3N KOH for pH adjustment). Subsequently, samples were incubated at 20°C, 150 rpm for 16 h. After centrifugation at 15.000 rpm for 5 min, the phases were separated and analyzed via the UV-trace at appropriate absorbance maxima of the LC-MS measurement.[12] For log D calculation (clog D) of compounds **1**, **2** and **7**, the calculator plugins of ChemAxon were used with MarvinSketch.[13]

Table S 10. *Kinetic solubilities and logD values.*

Compound	**1**	**2**	**7**
Solubility in H_2O with 5% DMSO [µg/mL]	nd	5.22	6.78
Solubility in PBS with 5% DMSO [µg/mL]	5.53	14.8	14.2
Log D	nd	1.06	1.63
clog D	1.63	1.82	0.64

2 COMPUTATIONAL METHODS

The Conformer–Rotamer Ensemble Sampling Tool (CREST)[14] using the GFN2-xTB tight-binding method[15] as implemented in the xtb program[16] was used for initial conformational search of **2** in water employing the GBSA solvation model. The geometry of **2** was fully optimized at the B3LYP[17-20] /level of theory with 6-311+G(d,p)[21, 22] basis set, BJ-damped DFT-D3 dispersion correction[23, 24] and conductor-like polarizable continuum model (CPCM(water))[25] to account for the solvation effect of water (Dielectric constant (ε) = 80.4; Refractive index = 1.33) using the Gaussian 16.A03 program.[26] Analytical frequencies were calculated to verify the obtained geometry as minimum. The obtained geometry of **2** was used as a reference structure for all other molecules (**5-14**) that were fully optimized using the same DFT method and the minima were verified by analytical frequencies calculations.

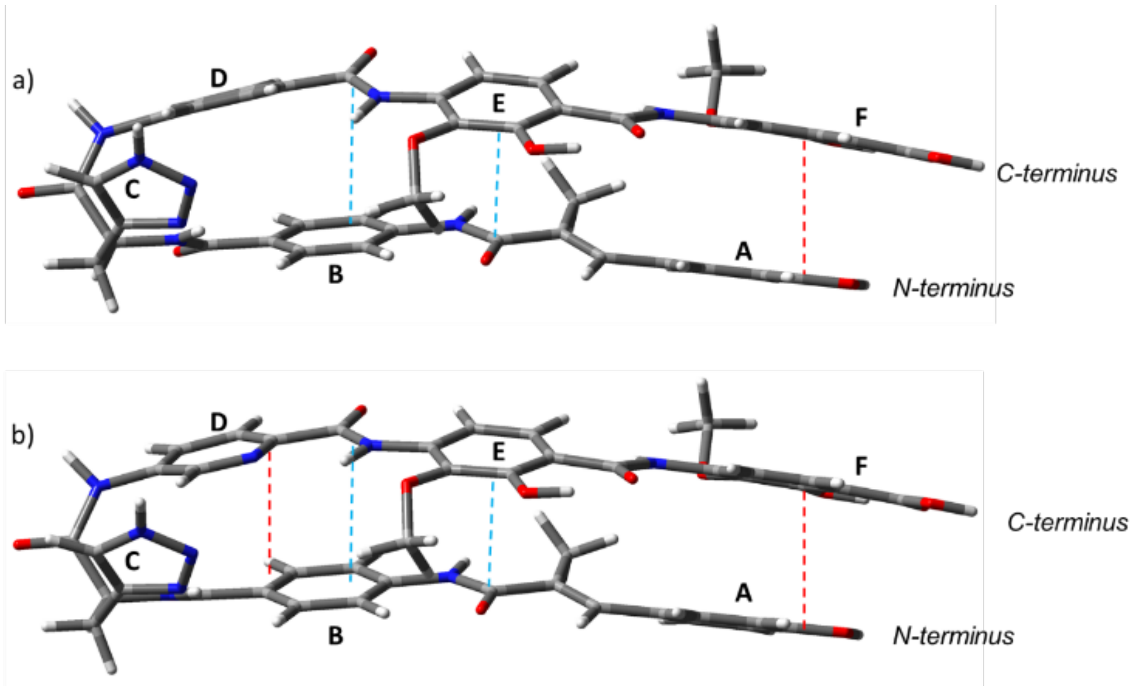

Figure S3. *Optimized geometries at the B3LYP CPCM(water) level of theory, with the BJ-damped DFT-D3 dispersion correction and 6-311+G(d,p) basis set of a)* ***2****, b)* ***7****. π-stacking interactions are shown in dashed lines (red color for aryl-aryl and blue color for aryl-amide interactions).*

The hairpin-like conformation of **2** (Figure S3, a) is stabilized by the favourable π-stacking interactions between the aromatic rings of the parallel branches. In particular, there is an off-centre parallel π-stacking between the phenyl rings of building blocks F and A (dashed red line). The distance between the two phenyls is around 3.5 Å which is in the range of classical parallel aromatic π-stacking.[20] In addition, there are phenyl-amide π-stacking interactions between the phenyl ring of building block E and the amide bond between building blocks A and B and between the phenyl ring of building block B and the amide bond between building blocks D and E (dashed blue lines). The distances between the phenyl ring and the amide carbonyl are 3.5Å as expected for aryl-amide π-stacking.[21] The phenyl rings of building blocks D and B are slightly rotated in such manner that the longest distance between the two aryls is 3.81Å and the shortest distance is 3.33Å which makes the π-stacking less efficient (as the two ring are not exactly parallel to each other).

In derivative **7**, the twisting of the aromatic ring of building block D results in the parallel alignment of the aryl rings of building blocks D and B, with the distance between the rings of 3.5Å resulting in a much more efficient π-stacking and an overall tightly packed molecule (Figure S3, b).

Cartesian coordinates of the optimized geometry of **2**:

C	6.41730	1.90681	-0.75095
C	7.77523	1.77065	-0.55209
C	8.40732	0.51874	-0.61385
C	7.63555	-0.63072	-0.90505
C	6.26440	-0.49295	-1.13408
C	5.64951	0.76395	-1.03003
N	4.27068	0.76011	-1.22854
C	3.35612	1.73871	-0.97059
O	3.68282	2.83786	-0.48626
C	1.94990	1.40791	-1.26561
C	1.57092	0.29770	-2.03591
C	0.24673	-0.03068	-2.24230
C	-0.75402	0.75465	-1.64934
C	-0.40719	1.89072	-0.89996
C	0.93742	2.24280	-0.73468
O	1.21771	3.37476	-0.05873
H	2.20444	3.46282	-0.07210
O	-1.44316	2.64486	-0.41805
C	-1.41865	3.05170	0.97138
H	-2.46218	3.12589	1.27470
H	-0.92541	4.01713	1.06967
H	-0.90102	2.30473	1.57128
N	-2.11966	0.48868	-1.73476
C	-2.75979	-0.54486	-2.36401
O	-2.18120	-1.40476	-3.02191
C	-4.24001	-0.57882	-2.15100
C	-4.87470	-1.81861	-2.26200
C	-6.22177	-1.95057	-1.95676
C	-6.95166	-0.83275	-1.55330
C	-6.35270	0.42533	-1.53940
C	-4.99738	0.54891	-1.81370
H	-6.94428	1.29522	-1.29395
N	-8.32205	-0.98766	-1.18601
C	-8.92484	-0.92489	0.03223
O	-10.14081	-1.07676	0.12990
C	-8.13736	-0.69718	1.34139
N	-6.69555	-0.76480	1.28520
C	-6.00116	-1.92858	1.25055
O	-6.56474	-3.02622	1.22818
C	-4.51491	-1.79178	1.20377
C	-3.76324	-2.85927	0.70077
C	-2.38605	-2.77146	0.59964
C	-1.70978	-1.61451	1.02277
C	-2.45412	-0.54341	1.53507
C	-3.83819	-0.63912	1.61376
H	-4.37775	0.20600	2.02431
H	-1.94955	0.34481	1.87173
N	-0.31405	-1.60952	0.90291
C	0.59559	-0.70818	1.40803

O	0.24494	0.30850	2.00609
C	2.03223	-1.08600	1.20810
C	2.92290	-0.32670	1.88084
C	4.36949	-0.46329	2.01499
C	5.06031	-1.68477	1.93792
C	6.43582	-1.74607	2.11251
C	7.16041	-0.57951	2.36417
C	6.49628	0.64333	2.46873
C	5.12176	0.69029	2.30665
H	4.61271	1.64400	2.38586
H	7.06628	1.54255	2.66420
O	8.51645	-0.57783	2.52595
H	8.86468	-1.47146	2.41909
H	6.94895	-2.69918	2.04928
H	4.52187	-2.60632	1.77080
H	2.49874	0.52546	2.40342
C	2.38635	-2.23910	0.30464
H	2.26462	-3.21128	0.79527
H	1.76436	-2.23936	-0.59432
H	3.42084	-2.16819	-0.02869
H	0.07021	-2.43216	0.46354
H	-1.82149	-3.60321	0.19340
H	-4.27574	-3.75371	0.37219
H	-6.19840	0.11432	1.22190
H	-8.48808	-1.50926	1.98068
C	-8.56974	0.63830	1.99214
C	-8.03296	1.83918	1.28325
C	-8.66034	2.75387	0.46510
N	-7.66757	3.58531	0.07416
N	-6.48722	3.23081	0.60019
N	-6.70537	2.17192	1.33462
H	-7.72884	4.39842	-0.52427
H	-9.68187	2.87084	0.14797
H	-9.65763	0.67286	2.01488
H	-8.21189	0.63536	3.02365
H	-8.95791	-1.27300	-1.92215
H	-6.70111	-2.92064	-1.98244
H	-4.28836	-2.68420	-2.53903
H	-2.67665	1.11280	-1.16640
H	-0.02948	-0.88799	-2.83202
H	2.32130	-0.32113	-2.51067
H	3.91427	-0.16059	-1.44547
O	5.46091	-1.57995	-1.37594
C	5.66564	-2.24438	-2.64306
H	4.90759	-3.02341	-2.69448
H	5.52840	-1.53662	-3.46440
H	6.66161	-2.68383	-2.68670
O	8.16073	-1.86921	-0.95395
H	9.12218	-1.77995	-0.75755
C	9.82943	0.35631	-0.33576
O	10.42045	-0.72409	-0.36867

O	10.47739	1.49057	-0.02993
H	11.40648	1.26961	0.13904
H	8.37108	2.64376	-0.32518
H	5.93683	2.86718	-0.67287
H	-4.55230	1.53571	-1.78166

Cartesian coordinates of the optimized geometry of **5**:

C	-6.56125	1.76566	0.85225
C	-7.90388	1.58687	0.59197
C	-8.47508	0.30653	0.51382
C	-7.65701	-0.82883	0.72227
C	-6.30309	-0.64997	1.01692
C	-5.74828	0.63792	1.05400
N	-4.37770	0.67838	1.30002
C	-3.50163	1.71197	1.14373
O	-3.86592	2.83765	0.75805
C	-2.08574	1.40646	1.41814
C	-1.67018	0.24852	2.09251
C	-0.33615	-0.05765	2.26787
C	0.63814	0.80505	1.74190
C	0.25360	1.98868	1.09211
C	-1.10098	2.31080	0.95368
O	-1.41633	3.48108	0.36377
H	-2.40460	3.54185	0.38851
O	1.25876	2.81930	0.66972
C	1.27689	3.19647	-0.72946
H	0.58852	4.02121	-0.90626
H	1.00454	2.34053	-1.34641
H	2.30058	3.50459	-0.93532
N	2.01170	0.57483	1.79827
C	2.68661	-0.49030	2.33170
O	2.13187	-1.41774	2.91386
C	4.16758	-0.47577	2.11298
C	4.88909	0.65685	1.71690
C	6.24642	0.56439	1.44301
C	6.89070	-0.66813	1.52917
C	6.19797	-1.78766	1.98862
C	4.84479	-1.68743	2.27907
H	4.28660	-2.55745	2.59679
H	6.71059	-2.73743	2.06880
N	8.27129	-0.78999	1.18589
C	8.89078	-0.77602	-0.02519
O	10.11512	-0.85991	-0.09598
C	8.11265	-0.69376	-1.35686
N	6.68005	-0.86212	-1.30557
C	6.08395	-2.06328	-1.15645
O	6.70348	-3.12389	-1.04913
C	4.58629	-1.99993	-1.08229
C	3.85606	-3.12491	-0.70386
C	2.48098	-3.01726	-0.59575

C	1.85844	-1.79510	-0.88286
C	2.68048	-0.71302	-1.24946
N	4.00410	-0.82343	-1.33848
H	2.24660	0.24804	-1.47166
N	0.46712	-1.72591	-0.77939
C	-0.37831	-0.75048	-1.26730
O	0.05513	0.27532	-1.78783
C	-1.83699	-1.06510	-1.15260
C	-2.65890	-0.20676	-1.79327
C	-4.10253	-0.25951	-2.00010
C	-4.84854	-1.45025	-2.07626
C	-6.21213	-1.43071	-2.32470
C	-6.87263	-0.21298	-2.49785
C	-6.15420	0.98247	-2.44281
C	-4.78757	0.95012	-2.21045
H	-4.23718	1.88295	-2.17064
H	-6.66392	1.92985	-2.57528
O	-8.21758	-0.25182	-2.73105
H	-8.56303	0.64487	-2.81984
H	-6.77776	-2.35217	-2.38344
H	-4.35723	-2.40703	-1.97302
H	-2.17199	0.66338	-2.22337
C	-2.28545	-2.26525	-0.35942
H	-2.16885	-3.20372	-0.91299
H	-3.33431	-2.17734	-0.07969
H	-1.71821	-2.35378	0.57104
H	0.03035	-2.56112	-0.41877
H	1.88489	-3.87131	-0.29517
H	4.36706	-4.05297	-0.48863
H	6.07280	-0.05066	-1.37143
C	8.47043	0.61470	-2.09714
C	7.88879	1.83298	-1.45866
C	8.47468	2.78854	-0.65606
N	7.45716	3.61868	-0.33235
N	6.30136	3.22491	-0.88691
N	6.56040	2.14170	-1.57058
H	7.48518	4.45560	0.23486
H	9.48274	2.93350	-0.30869
H	9.55552	0.69567	-2.13715
H	8.10190	0.52938	-3.12125
H	8.52940	-1.52392	-1.93108
H	8.91094	-0.97598	1.95002
H	6.80581	1.43937	1.14631
H	4.41940	1.62843	1.62854
H	2.54474	1.28291	1.31198
H	-0.03324	-0.95270	2.78353
H	-2.40000	-0.42943	2.51598
H	-3.98373	-0.24255	1.43718
O	-5.45584	-1.71719	1.18553
C	-5.66888	-2.50728	2.37686
H	-4.88047	-3.25733	2.37920

H	-6.64619	-2.98774	2.34617
H	-5.58481	-1.87641	3.26530
O	-8.12011	-2.08904	0.63286
H	-9.07788	-2.02696	0.41067
C	-9.88169	0.10573	0.18460
O	-10.41935	-0.99869	0.09658
O	-10.57744	1.23358	-0.02689
H	-11.49159	0.98809	-0.23857
H	-8.53506	2.44918	0.42795
H	-6.12610	2.75008	0.88191

Cartesian coordinates of the optimized geometry of **6**:

C	-6.43219	1.94590	0.61545
C	-7.78800	1.78895	0.41611
C	-8.41347	0.53953	0.55111
C	-7.63720	-0.58531	0.91751
C	-6.26840	-0.42548	1.14496
C	-5.66031	0.82686	0.96948
N	-4.28313	0.84055	1.17602
C	-3.37214	1.81612	0.89729
O	-3.69942	2.89812	0.37599
C	-1.96722	1.50140	1.21618
C	-1.59092	0.41162	2.01619
C	-0.26771	0.09225	2.24078
C	0.73529	0.86536	1.63576
C	0.39128	1.98249	0.85766
C	-0.95332	2.32680	0.67385
O	-1.23149	3.44083	-0.03171
H	-2.21883	3.52332	-0.03309
O	1.42895	2.72457	0.36130
C	1.40878	3.09725	-1.03769
H	2.45292	3.15724	-1.34183
H	0.92151	4.06284	-1.16051
H	0.88668	2.33956	-1.62004
N	2.10106	0.59869	1.73092
C	2.73473	-0.41833	2.39351
O	2.15012	-1.25033	3.08068
C	4.21380	-0.48033	2.17479
C	4.98807	0.62024	1.78951
C	6.34142	0.46414	1.52014
C	6.92142	-0.80112	1.58848
C	6.17323	-1.89070	2.03337
C	4.82860	-1.72538	2.33311
H	4.22945	-2.56924	2.64781
H	6.63783	-2.86593	2.10026
N	8.29262	-0.99235	1.24003
C	8.90489	-1.01820	0.02540
O	10.11643	-1.20871	-0.05371
C	8.13491	-0.83776	-1.30082

N	6.69030	-0.84542	-1.25287
C	5.94801	-1.97509	-1.17681
O	6.45444	-3.09648	-1.09917
C	4.46993	-1.76744	-1.15609
C	3.65949	-2.76826	-0.61628
N	2.33514	-2.67912	-0.52175
C	1.73583	-1.57061	-0.98684
C	2.45266	-0.50359	-1.54568
C	3.83079	-0.61372	-1.62072
H	4.39058	0.20372	-2.05934
H	1.93417	0.36497	-1.91077
N	0.34949	-1.60079	-0.85401
C	-0.59047	-0.72147	-1.35502
O	-0.26437	0.29797	-1.95913
C	-2.01261	-1.13221	-1.13850
C	-2.92317	-0.42411	-1.84137
C	-4.36442	-0.60079	-1.97296
C	-5.03310	-1.82763	-1.81826
C	-6.40598	-1.92583	-1.99411
C	-7.15055	-0.79167	-2.32467
C	-6.50872	0.43409	-2.50726
C	-5.13655	0.51716	-2.34308
H	-4.64466	1.47281	-2.48401
H	-7.09424	1.30753	-2.76384
O	-8.50517	-0.82516	-2.48907
H	-8.83757	-1.71691	-2.32946
H	-6.90218	-2.88191	-1.86960
H	-4.47966	-2.72607	-1.58779
H	-2.52061	0.41696	-2.39781
C	-2.33183	-2.25839	-0.18950
H	-2.21192	-3.24520	-0.65035
H	-3.35948	-2.18548	0.16507
H	-1.68869	-2.22212	0.69317
H	0.02137	-2.44705	-0.41023
H	4.11542	-3.67327	-0.23215
H	6.23151	0.05733	-1.24367
C	8.61957	0.44563	-2.01654
C	8.12350	1.69792	-1.36938
C	8.78108	2.63324	-0.59987
N	7.81534	3.51362	-0.25059
N	6.62307	3.16937	-0.75543
N	6.80641	2.06813	-1.43512
H	7.90331	4.35415	0.30517
H	9.80666	2.73487	-0.29070
H	9.70792	0.43887	-2.03399
H	8.26587	0.40387	-3.04861
H	8.45725	-1.69586	-1.89304
H	8.91731	-1.24803	1.99646
H	6.94532	1.31449	1.23817
H	4.55905	1.61197	1.71635
H	2.66177	1.21355	1.15602

H	0.00570	-0.74955	2.85348
H	-2.34225	-0.19884	2.49999
H	-3.92438	-0.06798	1.43613
O	-5.46055	-1.49110	1.45812
C	-5.65842	-2.06253	2.77091
H	-4.90730	-2.84359	2.87043
H	-6.65795	-2.48813	2.85470
H	-5.50658	-1.29988	3.53882
O	-8.15597	-1.82107	1.04124
H	-9.11756	-1.74917	0.83856
C	-9.83357	0.35205	0.27793
O	-10.42016	-0.72615	0.38165
O	-10.48477	1.45996	-0.10739
H	-11.41239	1.22399	-0.26379
H	-8.38734	2.64285	0.13234
H	-5.95654	2.90269	0.48279

Cartesian coordinates of the optimized geometry of **7**:

C	6.36533	1.78247	-1.05273
C	7.72424	1.70993	-0.82847
C	8.37975	0.47950	-0.66456
C	7.63082	-0.71785	-0.74847
C	6.25890	-0.64948	-1.00313
C	5.62021	0.59368	-1.12713
N	4.24326	0.52826	-1.32892
C	3.31079	1.52166	-1.26226
O	3.61663	2.69602	-0.98438
C	1.91269	1.12295	-1.50775
C	1.55413	-0.12240	-2.04808
C	0.23637	-0.49562	-2.21204
C	-0.78161	0.38617	-1.81375
C	-0.45587	1.65078	-1.29995
C	0.88418	2.03654	-1.17309
O	1.14132	3.27979	-0.72147
H	2.12781	3.37299	-0.72778
O	-1.49707	2.48662	-1.00068
C	-1.53102	3.08339	0.31797
H	-2.58538	3.20261	0.56433
H	-1.03266	4.05153	0.30294
H	-1.04617	2.42601	1.03842
N	-2.13873	0.09278	-1.88686
C	-2.74936	-1.06463	-2.26053
O	-2.18240	-2.06333	-2.69267
C	-4.23883	-1.02709	-2.05746
C	-4.97752	-2.19617	-2.20318
C	-6.32963	-2.16831	-1.88641
C	-6.89155	-0.96899	-1.45964
C	-6.08793	0.17432	-1.42955
N	-4.78680	0.14171	-1.69279

H	-6.51538	1.13129	-1.15664
N	-8.26609	-0.91052	-1.10630
C	-8.88646	-0.72707	0.09641
O	-10.11116	-0.77289	0.16113
C	-8.11953	-0.48338	1.41326
N	-6.67809	-0.57201	1.38511
C	-5.99359	-1.74332	1.40177
O	-6.56906	-2.83448	1.39540
C	-4.50604	-1.61978	1.37535
C	-3.75194	-2.72343	0.96054
C	-2.37279	-2.64877	0.87584
C	-1.69721	-1.46580	1.22149
C	-2.44408	-0.35803	1.64522
C	-3.82825	-0.44398	1.71356
H	-4.36588	0.43199	2.05608
H	-1.93987	0.55136	1.92038
N	-0.30036	-1.47566	1.12852
C	0.60066	-0.49700	1.48401
O	0.24021	0.60601	1.89351
C	2.04093	-0.89712	1.37186
C	2.92032	-0.02347	1.90678
C	4.36593	-0.12180	2.07714
C	5.06792	-1.33102	2.21960
C	6.44262	-1.34706	2.40879
C	7.15507	-0.14728	2.45525
C	6.47941	1.06848	2.34266
C	5.10576	1.07252	2.16758
H	4.58763	2.02030	2.07683
H	7.03998	1.99366	2.38098
O	8.51024	-0.10443	2.61737
H	8.86656	-0.99972	2.67007
H	6.96529	-2.29116	2.51408
H	4.53914	-2.27301	2.21357
H	2.48579	0.90582	2.26282
C	2.41077	-2.19264	0.69661
H	2.28189	-3.06057	1.35284
H	1.80365	-2.35832	-0.19730
H	3.44979	-2.17842	0.37065
H	0.09108	-2.35001	0.81289
H	-1.80695	-3.50949	0.53787
H	-4.26394	-3.63668	0.68745
H	-6.17188	0.30331	1.33340
H	-8.49763	-1.26812	2.07142
C	-8.53723	0.88402	2.00479
C	-7.98878	2.03840	1.22995
C	-8.60843	2.92081	0.37139
N	-7.60426	3.70751	-0.07739
N	-6.42386	3.35586	0.44924
N	-6.65372	2.34331	1.24382
H	-7.65617	4.48649	-0.72044
H	-9.63188	3.04297	0.06266

H	-9.62411	0.93166	2.02864
H	-8.17421	0.92634	3.03351
H	-8.91894	-1.19186	-1.82943
H	-6.93538	-3.06406	-1.93639
H	-4.48642	-3.10740	-2.51376
H	-2.76583	0.82004	-1.55391
H	-0.02317	-1.45611	-2.62446
H	2.31614	-0.81786	-2.37525
H	3.90479	-0.42276	-1.38120
O	5.47818	-1.77801	-1.05222
C	5.70032	-2.64622	-2.18590
H	4.96392	-3.44255	-2.09784
H	5.54319	-2.09539	-3.11662
H	6.70789	-3.05981	-2.15817
O	8.17906	-1.93488	-0.57473
H	9.13783	-1.79377	-0.39629
C	9.80351	0.39587	-0.36123
O	10.41474	-0.66182	-0.20241
O	10.42936	1.57842	-0.26106
H	11.36208	1.40841	-0.05664
H	8.30245	2.62083	-0.75918
H	5.86722	2.73209	-1.14904

Cartesian coordinates of the optimized geometry of **8**:

C	6.41730	1.90681	-0.75095
C	7.77523	1.77065	-0.55209
C	8.40732	0.51874	-0.61385
C	7.63555	-0.63072	-0.90505
C	6.26440	-0.49295	-1.13408
C	5.64951	0.76395	-1.03003
N	4.27068	0.76011	-1.22854
C	3.35612	1.73871	-0.97059
O	3.68282	2.83786	-0.48626
C	1.94990	1.40791	-1.26561
C	1.57092	0.29770	-2.03591
C	0.24673	-0.03068	-2.24231
C	-0.75402	0.75465	-1.64934
C	-0.40719	1.89072	-0.89996
C	0.93742	2.24280	-0.73468
O	1.21771	3.37476	-0.05873
H	2.20444	3.46282	-0.07210
O	-1.44316	2.64486	-0.41805
C	-1.41865	3.05170	0.97138
H	-2.46218	3.12589	1.27470
H	-0.92541	4.01713	1.06967
H	-0.90102	2.30473	1.57128
N	-2.11966	0.48868	-1.73476
C	-2.75979	-0.54486	-2.36401
O	-2.18120	-1.40476	-3.02191

C	-4.24001	-0.57882	-2.15100
C	-4.87470	-1.81861	-2.26200
C	-6.22177	-1.95057	-1.95676
C	-6.95166	-0.83275	-1.55330
N	-6.35270	0.42533	-1.53940
C	-4.99738	0.54891	-1.81370
N	-8.32205	-0.98766	-1.18601
C	-8.92484	-0.92489	0.03223
O	-10.14081	-1.07676	0.12990
C	-8.13736	-0.69718	1.34139
N	-6.69555	-0.76480	1.28520
C	-6.00116	-1.92858	1.25055
O	-6.56474	-3.02622	1.22818
C	-4.51491	-1.79178	1.20377
C	-3.76325	-2.85927	0.70077
C	-2.38605	-2.77146	0.59964
C	-1.70978	-1.61451	1.02277
C	-2.45412	-0.54341	1.53507
C	-3.83819	-0.63912	1.61376
H	-4.37775	0.20600	2.02431
H	-1.94955	0.34481	1.87173
N	-0.31405	-1.60952	0.90291
C	0.59559	-0.70818	1.40803
O	0.24495	0.30850	2.00609
C	2.03223	-1.08600	1.20810
C	2.92290	-0.32670	1.88084
C	4.36949	-0.46329	2.01499
C	5.06031	-1.68477	1.93792
C	6.43582	-1.74607	2.11251
C	7.16041	-0.57951	2.36417
C	6.49628	0.64333	2.46873
C	5.12176	0.69029	2.30665
H	4.61271	1.64400	2.38586
H	7.06628	1.54255	2.66420
O	8.51645	-0.57783	2.52595
H	8.86468	-1.47146	2.41909
H	6.94895	-2.69918	2.04928
H	4.52187	-2.60632	1.77080
H	2.49874	0.52546	2.40342
C	2.38635	-2.23910	0.30464
H	2.26462	-3.21128	0.79527
H	1.76437	-2.23936	-0.59432
H	3.42084	-2.16819	-0.02869
H	0.07021	-2.43216	0.46354
H	-1.82149	-3.60321	0.19340
H	-4.27574	-3.75371	0.37219
H	-6.19840	0.11432	1.22190
H	-8.48808	-1.50926	1.98068
C	-8.56974	0.63830	1.99214
C	-8.03296	1.83918	1.28325
C	-8.66034	2.75387	0.46510

N	-7.66757	3.58531	0.07416
N	-6.48722	3.23081	0.60019
N	-6.70537	2.17192	1.33462
H	-7.72884	4.39842	-0.52427
H	-9.68187	2.87084	0.14797
H	-9.65763	0.67286	2.01488
H	-8.21189	0.63536	3.02365
H	-8.95791	-1.27300	-1.92215
H	-6.70111	-2.92064	-1.98244
H	-4.28836	-2.68420	-2.53903
H	-2.67665	1.11280	-1.16640
H	-0.02948	-0.88799	-2.83202
H	2.32130	-0.32113	-2.51067
H	3.91427	-0.16059	-1.44547
O	5.46091	-1.57995	-1.37594
C	5.66564	-2.24438	-2.64306
H	4.90759	-3.02341	-2.69448
H	5.52840	-1.53662	-3.46440
H	6.66161	-2.68383	-2.68670
O	8.16073	-1.86921	-0.95395
H	9.12218	-1.77995	-0.75755
C	9.82943	0.35631	-0.33576
O	10.42045	-0.72409	-0.36867
O	10.47739	1.49057	-0.02994
H	11.40649	1.26961	0.13904
H	8.37108	2.64376	-0.32518
H	5.93683	2.86718	-0.67287
H	-4.55230	1.53571	-1.78166

Cartesian coordinates of the optimized geometry of **9**:

C	-6.37201	2.19510	-0.04604
C	-7.72607	1.94515	-0.11019
C	-8.28957	0.79281	0.46147
C	-7.44786	-0.12553	1.13309
C	-6.07892	0.13878	1.22505
C	-5.53424	1.27934	0.61587
N	-4.15741	1.41765	0.71889
C	-3.32083	2.27481	0.06608
O	-3.67256	3.14324	-0.72670
C	-1.87847	2.04025	0.40448
N	-1.60643	1.13190	1.34863
C	-0.34156	0.86284	1.65610
C	0.73374	1.49424	1.00524
C	0.44070	2.45092	0.02697
C	-0.87930	2.73241	-0.27523
N	2.07630	1.20305	1.25267
C	2.61261	0.30011	2.13788
O	1.93451	-0.36914	2.90928
C	4.09785	0.15841	2.05454

C	4.65126	-1.05692	2.46597
C	6.00445	-1.30971	2.29075
C	6.82091	-0.33617	1.71529
C	6.29859	0.91775	1.40343
C	4.93954	1.15696	1.55017
H	6.95507	1.69058	1.03166
N	8.19781	-0.62340	1.47927
C	8.87979	-0.79188	0.31300
O	10.09552	-0.97096	0.32710
C	8.17925	-0.82103	-1.06414
N	6.73653	-0.89385	-1.08091
C	6.05230	-2.03802	-0.83317
O	6.62900	-3.09712	-0.57015
C	4.56375	-1.92377	-0.84594
C	3.82589	-2.89644	-0.16267
C	2.44857	-2.80894	-0.07426
C	1.75818	-1.75044	-0.68803
C	2.48646	-0.78083	-1.39162
C	3.87239	-0.87265	-1.45658
H	4.40216	-0.10544	-2.00892
H	1.96802	0.03078	-1.87479
N	0.36582	-1.72626	-0.53919
C	-0.56723	-0.98575	-1.23450
O	-0.24283	-0.16148	-2.08640
C	-1.99048	-1.30051	-0.88559
C	-2.91236	-0.83589	-1.75490
C	-4.35849	-1.02763	-1.78787
C	-5.02781	-2.13251	-1.23344
C	-6.40711	-2.25807	-1.32253
C	-7.15757	-1.27340	-1.96784
C	-6.51608	-0.18010	-2.55077
C	-5.13792	-0.07218	-2.46693
H	-4.64713	0.78248	-2.91853
H	-7.10594	0.57787	-3.05001
O	-8.51851	-1.33432	-2.06651
H	-8.84942	-2.11473	-1.60548
H	-6.90371	-3.11727	-0.88524
H	-4.47126	-2.92148	-0.74963
H	-2.52111	-0.21317	-2.55398
C	-2.29644	-2.07536	0.37039
H	-0.00014	-2.43239	0.08170
H	1.89460	-3.56140	0.47579
H	4.35105	-3.70989	0.32005
H	6.23362	-0.02694	-1.22143
H	8.56617	-1.74011	-1.50761
C	8.66023	0.35900	-1.94014
C	8.07860	1.67603	-1.54046
C	8.65608	2.76269	-0.91958
N	7.63864	3.64294	-0.77748
N	6.49050	3.15781	-1.26898
N	6.75393	1.96523	-1.73360

H	7.66145	4.57073	-0.37538
H	9.65837	2.96688	-0.58568
H	9.74723	0.40015	-1.89130
H	8.38032	0.13895	-2.97239
H	8.77585	-0.77642	2.29777
H	6.42302	-2.27385	2.54913
H	4.00008	-1.81932	2.87111
H	2.72425	1.63309	0.60911
H	-0.15803	0.12292	2.41902
H	-3.68149	0.72072	1.28513
O	-5.21914	-0.74568	1.82717
C	-5.32969	-0.84284	3.26293
H	-4.55060	-1.53568	3.57539
H	-5.16134	0.13549	3.72060
H	-6.30940	-1.22686	3.54727
O	-7.90590	-1.26704	1.68142
H	-8.87513	-1.29852	1.50771
C	-9.70786	0.48332	0.33721
O	-10.23862	-0.52693	0.80271
O	-10.42799	1.39223	-0.33876
H	-11.34765	1.08560	-0.36568
H	-8.37391	2.63977	-0.62667
H	-5.94208	3.06683	-0.51060
H	4.55770	2.13584	1.28760
H	-1.14180	3.45770	-1.03251
H	-1.62131	-1.78990	1.17989
H	-2.21255	-3.15915	0.22874
H	-3.30697	-1.86229	0.71592
H	1.24479	2.95458	-0.49700

Cartesian coordinates of the optimized geometry of **10**:

C	-6.28827	2.19905	0.08563
C	-7.65368	2.07118	-0.06185
C	-8.33755	0.92382	0.37023
C	-7.60979	-0.12246	0.98486
C	-6.23079	0.01470	1.15967
C	-5.56523	1.15771	0.69180
N	-4.18620	1.15989	0.88485
C	-3.24360	2.02205	0.38666
O	-3.50434	2.95914	-0.36063
C	-1.84912	1.72000	0.81555
C	-1.51429	1.02422	1.98213
C	-0.18640	0.77493	2.28423
C	0.78333	1.23759	1.38528
N	0.48280	1.93695	0.28115
C	-0.79726	2.16961	0.01351
N	2.15111	1.01965	1.52947
C	2.81117	0.22676	2.43799
O	2.24561	-0.37885	3.34071
C	4.28284	0.12389	2.20677
C	4.91938	-1.04949	2.61899

C	6.25840	-1.26246	2.32482
C	6.97879	-0.28780	1.63384
C	6.37866	0.92795	1.31155
C	5.02924	1.12323	1.57149
H	6.96445	1.70427	0.84181
N	8.34070	-0.53829	1.29468
C	8.93461	-0.74732	0.08738
O	10.14787	-0.93174	0.02091
C	8.14202	-0.80575	-1.23714
N	6.69955	-0.83972	-1.16240
C	5.99910	-1.95474	-0.83996
O	6.55908	-3.01833	-0.56075
C	4.51346	-1.80376	-0.80696
C	3.77399	-2.71559	-0.04645
C	2.39738	-2.60930	0.04559
C	1.71175	-1.59580	-0.64364
C	2.44262	-0.67642	-1.40795
C	3.82726	-0.78560	-1.47712
H	4.36019	-0.06448	-2.08543
H	1.92727	0.10812	-1.93639
N	0.31683	-1.55830	-0.50746
C	-0.60793	-0.94417	-1.32503
O	-0.27253	-0.23304	-2.26971
C	-2.03850	-1.24105	-0.98600
C	-2.94849	-0.75060	-1.85414
C	-4.39673	-0.92378	-1.89859
C	-5.07183	-2.06281	-1.42776
C	-6.45153	-2.17437	-1.52851
C	-7.19488	-1.13996	-2.10004
C	-6.54688	-0.00920	-2.59900
C	-5.16847	0.08416	-2.50678
H	-4.67157	0.96628	-2.89411
H	-7.13220	0.78657	-3.04149
O	-8.55541	-1.18472	-2.20399
H	-8.89061	-2.00260	-1.81708
H	-6.95364	-3.06120	-1.15792
H	-4.51812	-2.88848	-1.00437
H	-2.54277	-0.11950	-2.63925
C	-2.36760	-2.02942	0.25557
H	-0.05076	-2.19265	0.18519
H	1.84078	-3.31800	0.64848
H	4.29501	-3.50143	0.48406
H	6.21159	0.03517	-1.30637
H	8.47802	-1.74651	-1.67699
C	8.59095	0.33964	-2.17451
C	8.07877	1.67936	-1.75603
C	8.72548	2.74616	-1.16977
N	7.74806	3.66096	-0.97639
N	6.55921	3.21524	-1.40470
N	6.75638	2.01333	-1.87884
H	7.82422	4.58781	-0.57865

H	9.75072	2.91555	-0.89050
H	9.67905	0.34828	-2.20724
H	8.22669	0.10902	-3.17761
H	8.97891	-0.67666	2.07004
H	6.73884	-2.19623	2.58708
H	4.34167	-1.81254	3.12303
H	2.68217	1.37783	0.74751
H	-3.85658	0.32628	1.35287
O	-5.47138	-0.98771	1.71160
C	-5.67719	-1.22438	3.12212
H	-4.96109	-1.99472	3.40140
H	-5.48047	-0.31071	3.68872
H	-6.69376	-1.57058	3.30643
O	-8.18651	-1.26647	1.39852
H	-9.14825	-1.19044	1.19752
C	-9.77092	0.75384	0.16533
O	-10.40839	-0.23525	0.53006
O	-10.37557	1.77223	-0.46553
H	-11.31741	1.55647	-0.54941
H	-8.21552	2.86416	-0.53562
H	-5.76743	3.07066	-0.27322
H	4.57897	2.06925	1.29690
H	-1.01462	2.72525	-0.89095
H	-1.75815	-1.70408	1.10311
H	-2.20488	-3.10513	0.12485
H	-3.40834	-1.89033	0.54395
H	-2.27649	0.68715	2.67425
H	0.10404	0.23660	3.17082

Cartesian coordinates of the optimized geometry of **11**:

C	-6.37366	2.17821	0.18941
C	-7.73686	2.00873	0.06793
C	-8.37172	0.82359	0.47248
C	-7.59443	-0.21697	1.03414
C	-6.21752	-0.03831	1.18305
C	-5.60014	1.14131	0.73957
N	-4.21814	1.18185	0.89289
C	-3.31543	2.08758	0.39181
O	-3.63503	3.04176	-0.31103
C	-1.89740	1.80536	0.75249
C	-1.52258	1.08923	1.89434
C	-0.18792	0.82615	2.17621
C	0.80372	1.27828	1.29613
C	0.43544	2.02341	0.16524
C	-0.89523	2.28711	-0.09664
N	2.16943	1.02191	1.46467
C	2.79917	0.25184	2.40791
O	2.21494	-0.32587	3.31911

C	4.27614	0.13208	2.20672
C	4.89325	-1.04912	2.62544
C	6.23398	-1.27718	2.34947
C	6.97538	-0.30946	1.67111
C	6.39521	0.91517	1.34713
C	5.04451	1.12563	1.58903
H	6.99717	1.68628	0.88938
N	8.33751	-0.57618	1.34424
C	8.93808	-0.78931	0.14141
O	10.14987	-0.98714	0.08338
C	8.15578	-0.83544	-1.18995
N	6.71219	-0.85883	-1.12931
C	6.00182	-1.97000	-0.81578
O	6.55226	-3.03582	-0.52605
C	4.51675	-1.81262	-0.80746
C	3.76250	-2.70098	-0.03358
C	2.38552	-2.58732	0.03511
C	1.71290	-1.59254	-0.69385
C	2.45861	-0.70040	-1.47650
C	3.84443	-0.81370	-1.51881
H	4.39041	-0.11418	-2.14066
H	1.95348	0.06389	-2.04357
N	0.31786	-1.54833	-0.57694
C	-0.59618	-0.92287	-1.39636
O	-0.24909	-0.22289	-2.34545
C	-2.03066	-1.19826	-1.05576
C	-2.93722	-0.68323	-1.91235
C	-4.38878	-0.83964	-1.94772
C	-5.06606	-1.99245	-1.51600
C	-6.44717	-2.09417	-1.61116
C	-7.18971	-1.03544	-2.13704
C	-6.53981	0.11226	-2.59284
C	-5.15987	0.19572	-2.50782
H	-4.66171	1.09053	-2.86315
H	-7.12440	0.92791	-2.99864
O	-8.55123	-1.07308	-2.23876
H	-8.88701	-1.90488	-1.88339
H	-6.95046	-2.99322	-1.27299
H	-4.51223	-2.83615	-1.12933
H	-2.52821	-0.04764	-2.69220
C	-2.36699	-1.99158	0.18070
H	-0.06044	-2.15921	0.13064
H	1.81756	-3.27569	0.65081
H	4.27247	-3.47337	0.52657
H	6.23080	0.01917	-1.27723
H	8.48824	-1.77705	-1.63067
C	8.62252	0.30952	-2.11913
C	8.11797	1.65223	-1.70122
C	8.76856	2.71131	-1.10537
N	7.79759	3.63430	-0.91845
N	6.60898	3.20060	-1.35977

N	6.79997	1.99849	-1.83587
H	7.87820	4.55907	-0.51678
H	9.79251	2.87045	-0.81556
H	9.71092	0.30916	-2.14191
H	8.26565	0.08528	-3.12634
H	8.96861	-0.71985	2.12440
H	6.69930	-2.21773	2.61488
H	4.29944	-1.80709	3.11848
H	2.75113	1.34293	0.70493
H	0.08800	0.26898	3.05608
H	-3.84440	0.34220	1.31387
O	-5.41443	-1.03500	1.68180
C	-5.57057	-1.31619	3.09002
H	-4.82866	-2.07681	3.32537
H	-5.37755	-0.41463	3.67707
H	-6.57271	-1.69188	3.29534
O	-8.12210	-1.39400	1.42114
H	-9.08858	-1.34848	1.23497
C	-9.80070	0.60858	0.28515
O	-10.39430	-0.41944	0.61590
O	-10.45537	1.62953	-0.28972
H	-11.38916	1.37989	-0.36878
H	-8.33587	2.79791	-0.36496
H	-5.89085	3.07883	-0.15020
H	4.60921	2.07850	1.31331
H	-1.17534	2.84656	-0.97915
H	-1.76898	-1.65875	1.03380
H	-2.19314	-3.06564	0.05058
H	-3.41119	-1.86123	0.45909
H	1.20021	2.37679	-0.51648
H	-2.26810	0.74136	2.59947

Cartesian coordinates of the optimized geometry of **12**:

C	-6.91654	-1.83857	-0.55770
C	-8.28389	-1.64431	-0.41417
C	-8.85364	-0.43744	-0.81558
N	-8.13103	0.54821	-1.36979
C	-6.82998	0.36690	-1.50808
C	-6.15701	-0.80231	-1.10323
N	-4.77466	-0.83054	-1.28690
C	-3.86517	-1.69369	-0.73827
O	-4.22614	-2.62348	0.00509
C	-2.44475	-1.45020	-1.04249
C	-2.01085	-0.53708	-2.01620
C	-0.67360	-0.28246	-2.23802
C	0.28937	-0.94475	-1.46012
C	-0.11017	-1.88761	-0.49936
C	-1.46846	-2.16519	-0.30449

O	-1.79334	-3.11434	0.59452
H	-2.78212	-3.17486	0.58349
O	0.88941	-2.55256	0.15862
C	0.83969	-2.62731	1.60431
H	1.87596	-2.71279	1.92848
H	0.27156	-3.50213	1.91529
H	0.38910	-1.72221	2.00907
N	1.66359	-0.73970	-1.56309
C	2.34818	0.12853	-2.37146
O	1.80374	0.87631	-3.17831
C	3.83139	0.12948	-2.17593
C	4.52817	1.27295	-2.57530
C	5.89018	1.38923	-2.33761
C	6.57410	0.34905	-1.70885
C	5.90698	-0.83231	-1.38996
C	4.53839	-0.93338	-1.60082
H	6.45836	-1.65853	-0.96556
N	7.96766	0.49415	-1.43634
C	8.63607	0.65636	-0.26188
O	9.86224	0.74247	-0.25523
C	7.91434	0.77557	1.09802
N	6.47812	0.92389	1.08448
C	5.84924	2.09227	0.80554
O	6.47293	3.12216	0.53522
C	4.35733	2.03510	0.80009
C	3.65820	3.02284	0.09824
C	2.27845	2.98228	0.00169
C	1.54814	1.95697	0.62533
C	2.23900	0.97248	1.34479
C	3.62546	1.01712	1.41913
H	4.12190	0.23975	1.98766
H	1.69263	0.18710	1.83683
N	0.15528	1.97923	0.47421
C	-0.79685	1.24459	1.14366
O	-0.49414	0.39412	1.97994
C	-2.21607	1.58330	0.80086
C	-3.14671	0.98714	1.57620
C	-4.59841	1.12846	1.60536
C	-5.30169	2.27121	1.18403
C	-6.68490	2.34255	1.28140
C	-7.40626	1.26699	1.80391
C	-6.73137	0.12907	2.24834
C	-5.35041	0.07281	2.15637
H	-4.83440	-0.81551	2.50180
H	-7.29776	-0.70053	2.65200
O	-8.76626	1.27781	1.91349
H	-9.12590	2.07471	1.50500
H	-7.20608	3.23545	0.95399
H	-4.76974	3.13444	0.81140
H	-2.75296	0.27387	2.29386
C	-2.50655	2.51519	-0.34638

H	-2.39830	3.56978	-0.06939
H	-1.84371	2.31897	-1.19220
H	-3.52342	2.37832	-0.71037
H	-0.18614	2.69390	-0.15028
H	1.75497	3.74586	-0.56261
H	4.21397	3.81122	-0.39184
H	5.93096	0.08502	1.22765
H	8.34395	1.68378	1.52421
C	8.30807	-0.40924	2.01131
C	7.67025	-1.69937	1.60904
C	8.20264	-2.81008	0.99041
N	7.14692	-3.64134	0.83478
N	6.01736	-3.10441	1.31556
N	6.33121	-1.92624	1.78637
H	7.13131	-4.56811	0.43003
H	9.19793	-3.05970	0.66652
H	9.39226	-0.50743	1.99758
H	8.00640	-0.15718	3.02988
H	8.57671	0.57007	-2.24307
H	6.41975	2.29709	-2.59634
H	3.98066	2.08660	-3.03103
H	2.19112	-1.26349	-0.87764
H	-0.35845	0.42601	-2.98475
H	-2.72410	-0.01383	-2.63952
H	-4.39801	-0.02865	-1.76898
C	-10.30248	-0.14634	-0.62120
O	-10.84890	0.89819	-0.89917
O	-10.96659	-1.18975	-0.07956
H	-11.89424	-0.92947	0.02870
H	-8.89843	-2.41985	0.01971
H	-6.44791	-2.75362	-0.23626
H	4.04471	-1.85952	-1.33356
H	-6.26437	1.18419	-1.94700

Cartesian coordinates of the optimized geometry of **13**:

C	-6.90302	-1.88294	-0.50009
C	-8.26286	-1.67408	-0.35880
C	-8.82982	-0.47387	-0.79952
C	-7.98573	0.46157	-1.40201
N	-6.67954	0.27728	-1.55137
C	-6.14397	-0.86131	-1.08691
N	-4.76226	-0.90757	-1.26148
C	-3.84925	-1.76316	-0.70613
O	-4.20310	-2.69128	0.04252
C	-2.43149	-1.51147	-1.01293
C	-2.00617	-0.60153	-1.99366
C	-0.67124	-0.34183	-2.22238
C	0.29803	-0.99311	-1.44271
C	-0.09312	-1.92901	-0.47195

C	-1.44883	-2.21462	-0.27211
O	-1.76506	-3.15904	0.63485
H	-2.75308	-3.22840	0.62515
O	0.91131	-2.57941	0.19338
C	0.86553	-2.62473	1.64048
H	1.90260	-2.70161	1.96412
H	0.29949	-3.49384	1.97077
H	0.41380	-1.71237	2.02763
N	1.67053	-0.78139	-1.55164
C	2.34886	0.07884	-2.37379
O	1.79917	0.81100	-3.19123
C	3.83205	0.09193	-2.17877
C	4.52156	1.23403	-2.59429
C	5.88229	1.36339	-2.35592
C	6.57203	0.33774	-1.70995
C	5.91283	-0.84370	-1.37531
C	4.54545	-0.95760	-1.58729
H	6.46934	-1.65943	-0.93749
N	7.96341	0.49724	-1.43448
C	8.62487	0.68332	-0.25962
O	9.85036	0.77800	-0.24830
C	7.89542	0.81985	1.09464
N	6.45836	0.95896	1.07186
C	5.82371	2.11923	0.77286
O	6.44250	3.14865	0.48968
C	4.33212	2.05352	0.76231
C	3.63048	3.02713	0.04333
C	2.25135	2.97815	-0.05751
C	1.52385	1.95866	0.57895
C	2.21713	0.98824	1.31494
C	3.60313	1.04099	1.39352
H	4.10138	0.27461	1.97522
H	1.67278	0.20748	1.81644
N	0.13126	1.97386	0.42544
C	-0.81996	1.24418	1.10185
O	-0.51704	0.39670	1.94091
C	-2.23890	1.58818	0.76354
C	-3.17030	1.00976	1.55066
C	-4.62006	1.17379	1.58411
C	-5.30081	2.33903	1.18964
C	-6.68235	2.43647	1.29178
C	-7.42377	1.36329	1.79028
C	-6.77056	0.20157	2.20558
C	-5.39112	0.12069	2.11204
H	-4.89243	-0.78510	2.43734
H	-7.35243	-0.62529	2.59221
O	-8.78306	1.39877	1.90965
H	-9.12719	2.22501	1.54911
H	-7.18580	3.34805	0.98847
H	-4.75085	3.19884	0.83487
H	-2.78179	0.29815	2.27293

C	-2.53105	2.50653	-0.39420
H	-2.40116	3.56378	-0.13731
H	-1.88315	2.28475	-1.24557
H	-3.55573	2.37927	-0.73994
H	-0.21199	2.68254	-0.20493
H	1.72612	3.73088	-0.63460
H	4.18394	3.81117	-0.45626
H	5.91575	0.11907	1.22612
H	8.31725	1.73758	1.50799
C	8.29194	-0.34724	2.02934
C	7.66305	-1.64762	1.64644
C	8.20441	-2.76589	1.04960
N	7.15398	-3.60554	0.90341
N	6.01924	-3.06663	1.36952
N	6.32444	-1.87884	1.82143
H	7.14532	-4.53922	0.51467
H	9.20257	-3.01560	0.73476
H	9.37672	-0.43951	2.02177
H	7.98459	-0.07961	3.04225
H	8.57561	0.56398	-2.23963
H	6.40603	2.27098	-2.62707
H	3.96913	2.03712	-3.06263
H	2.20260	-1.29346	-0.86085
H	-0.36228	0.36282	-2.97530
H	-2.72583	-0.08640	-2.61605
H	-4.41388	-0.09043	-1.74190
C	-10.25711	-0.14142	-0.62996
O	-10.77346	0.89875	-0.98735
O	-10.95596	-1.12204	-0.02065
H	-11.87650	-0.82804	0.05971
H	-8.88067	-2.43092	0.10510
H	-6.42837	-2.78414	-0.15079
H	4.05784	-1.88325	-1.30746
H	-8.39242	1.39816	-1.76578

Cartesian coordinates of the optimized geometry of **14**:

C	-6.88337	-1.85409	-0.56688
C	-8.25384	-1.69920	-0.41520
C	-8.89219	-0.51489	-0.79785
C	-8.13159	0.51692	-1.35987
C	-6.76662	0.37449	-1.51337
C	-6.12594	-0.80467	-1.10368
N	-4.73555	-0.85270	-1.27120
C	-3.83113	-1.70587	-0.70831
O	-4.18544	-2.62446	0.05436
C	-2.40853	-1.46940	-1.02101
C	-1.97568	-0.57948	-2.01617
C	-0.63821	-0.33286	-2.24844
C	0.32548	-0.97907	-1.45875

C	-0.07314	-1.89732	-0.47440
C	-1.43143	-2.16852	-0.26997
O	-1.75628	-3.09672	0.65112
H	-2.74646	-3.15518	0.63893
O	0.92744	-2.54618	0.19853
C	0.87513	-2.59312	1.64501
H	1.91156	-2.65756	1.97368
H	0.31846	-3.46957	1.97179
H	0.41011	-1.68728	2.03133
N	1.70032	-0.77904	-1.56959
C	2.38587	0.06421	-2.40200
O	1.84433	0.78674	-3.23375
C	3.86807	0.07254	-2.19860
C	4.56473	1.21039	-2.61366
C	5.92354	1.33673	-2.36301
C	6.60382	0.31201	-1.70570
C	5.93848	-0.86644	-1.37305
C	4.57271	-0.97703	-1.59692
H	6.48855	-1.68173	-0.92624
N	7.99207	0.46996	-1.41407
C	8.63725	0.66370	-0.23163
O	9.86259	0.75768	-0.20377
C	7.88933	0.80919	1.11170
N	6.45240	0.94700	1.07050
C	5.81983	2.10582	0.76190
O	6.44028	3.13391	0.47737
C	4.32817	2.04086	0.74506
C	3.62997	3.00674	0.01238
C	2.25073	2.96076	-0.08855
C	1.51941	1.95282	0.56194
C	2.20940	0.98929	1.30993
C	3.59566	1.03851	1.38837
H	4.09158	0.27856	1.98043
H	1.66221	0.21740	1.82181
N	0.12671	1.97312	0.41068
C	-0.82754	1.25736	1.09772
O	-0.52746	0.41675	1.94486
C	-2.24506	1.60772	0.76043
C	-3.17943	1.02491	1.54114
C	-4.62846	1.19302	1.57851
C	-5.30641	2.36468	1.19819
C	-6.68694	2.46591	1.30827
C	-7.43025	1.39067	1.79991
C	-6.78009	0.22284	2.20075
C	-5.40172	0.13768	2.09907
H	-4.90482	-0.77380	2.41061
H	-7.36378	-0.60695	2.57811
O	-8.78837	1.43155	1.92565
H	-9.13095	2.25622	1.56045
H	-7.18865	3.38184	1.01535
H	-4.75481	3.22622	0.84998

H	-2.79284	0.30512	2.25638
C	-2.53128	2.53686	-0.39010
H	-2.37412	3.58988	-0.13115
H	-1.89853	2.30350	-1.25014
H	-3.56220	2.43513	-0.72450
H	-0.21372	2.67480	-0.22886
H	1.72820	3.70783	-0.67539
H	4.18595	3.78297	-0.49651
H	5.90853	0.10788	1.22484
H	8.30512	1.72999	1.52435
C	8.27443	-0.35157	2.05919
C	7.65095	-1.65454	1.67620
C	8.19974	-2.77563	1.09156
N	7.15145	-3.61653	0.93742
N	6.01109	-3.07575	1.38735
N	6.31046	-1.88550	1.83664
H	7.14769	-4.55228	0.55359
H	9.20163	-3.02655	0.78977
H	9.35923	-0.44327	2.06633
H	7.95369	-0.07764	3.06624
H	8.61509	0.52981	-2.21143
H	6.45263	2.24161	-2.63279
H	4.01902	2.01310	-3.09054
H	2.22741	-1.28167	-0.86818
H	-0.32395	0.35741	-3.01246
H	-2.69006	-0.06957	-2.64932
H	-4.35090	-0.06238	-1.76470
C	-10.33812	-0.29817	-0.59969
O	-10.94248	0.70767	-0.92037
O	-10.95965	-1.34315	-0.00714
H	-11.89730	-1.11512	0.08488
H	-8.83165	-2.50522	0.01616
H	-6.39796	-2.76220	-0.25105
H	4.07898	-1.89963	-1.31759
H	-8.61907	1.43788	-1.65128
H	-6.18067	1.18767	-1.92458

3 NMR Spectra

3.1 NMR spectra of A-Variations (3, 4) and reaction intermediates

(E)-2-methyl-3-(pyridin-2-yl)acrylic acid (18a)

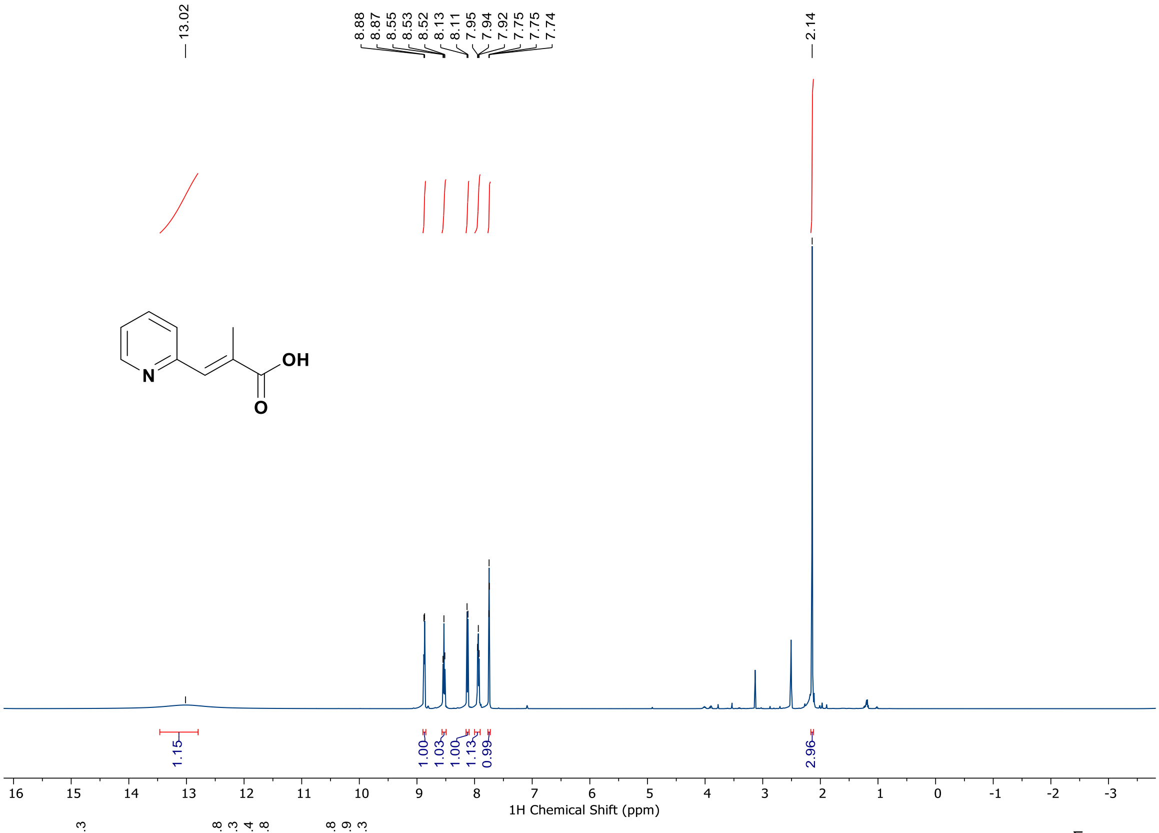

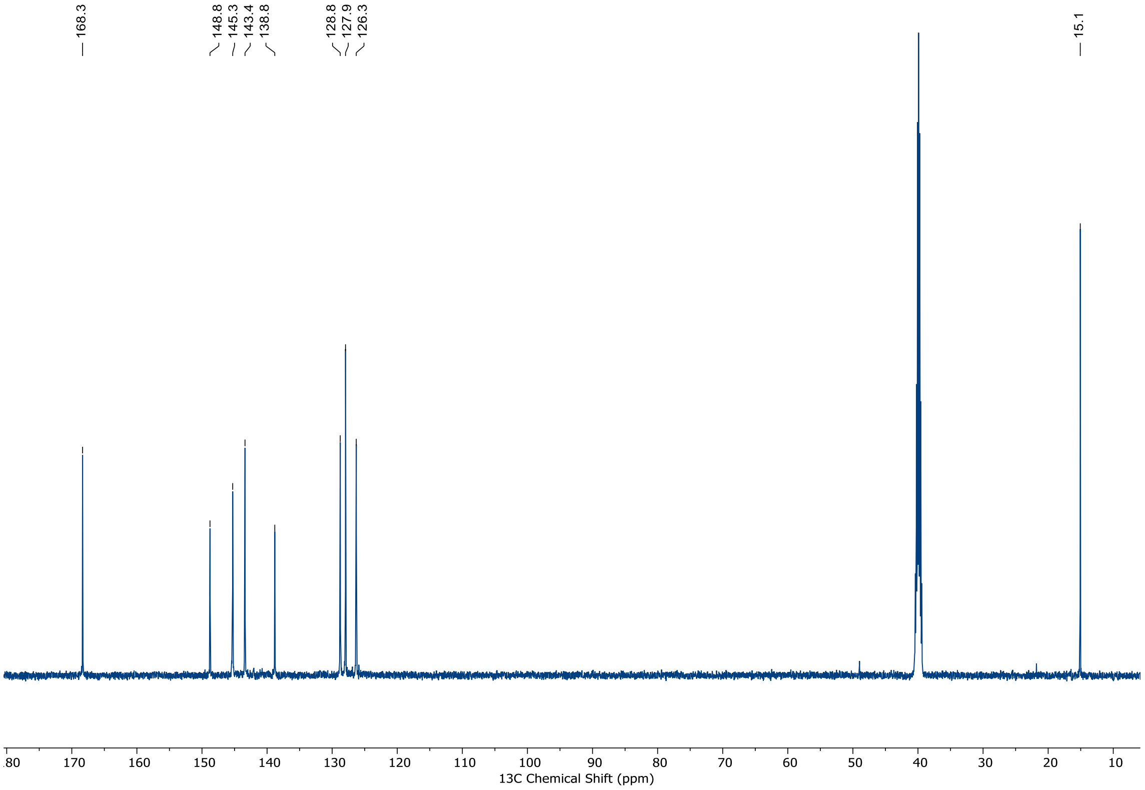

tert-butyl (E)-4-(2-methyl-3-(pyridin-2-yl)acrylamido)benzoate (18a-2)

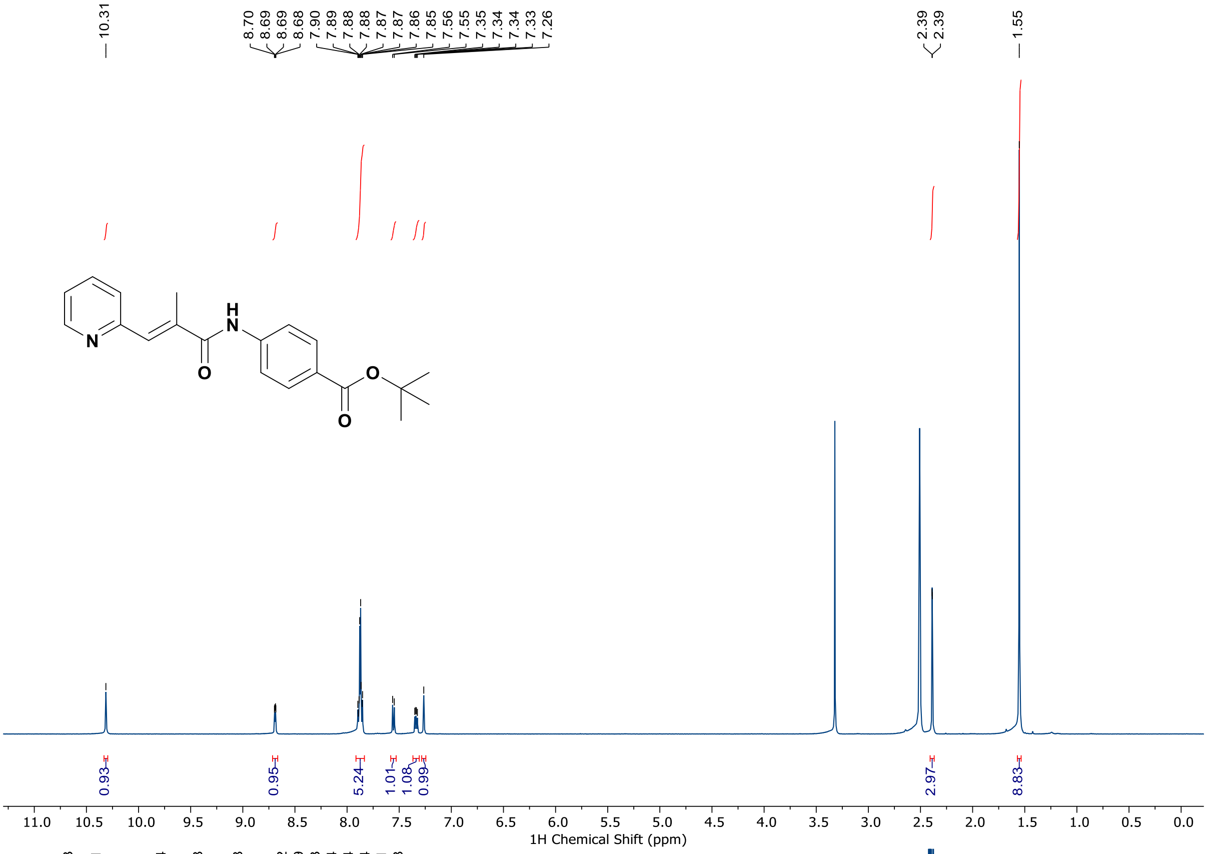

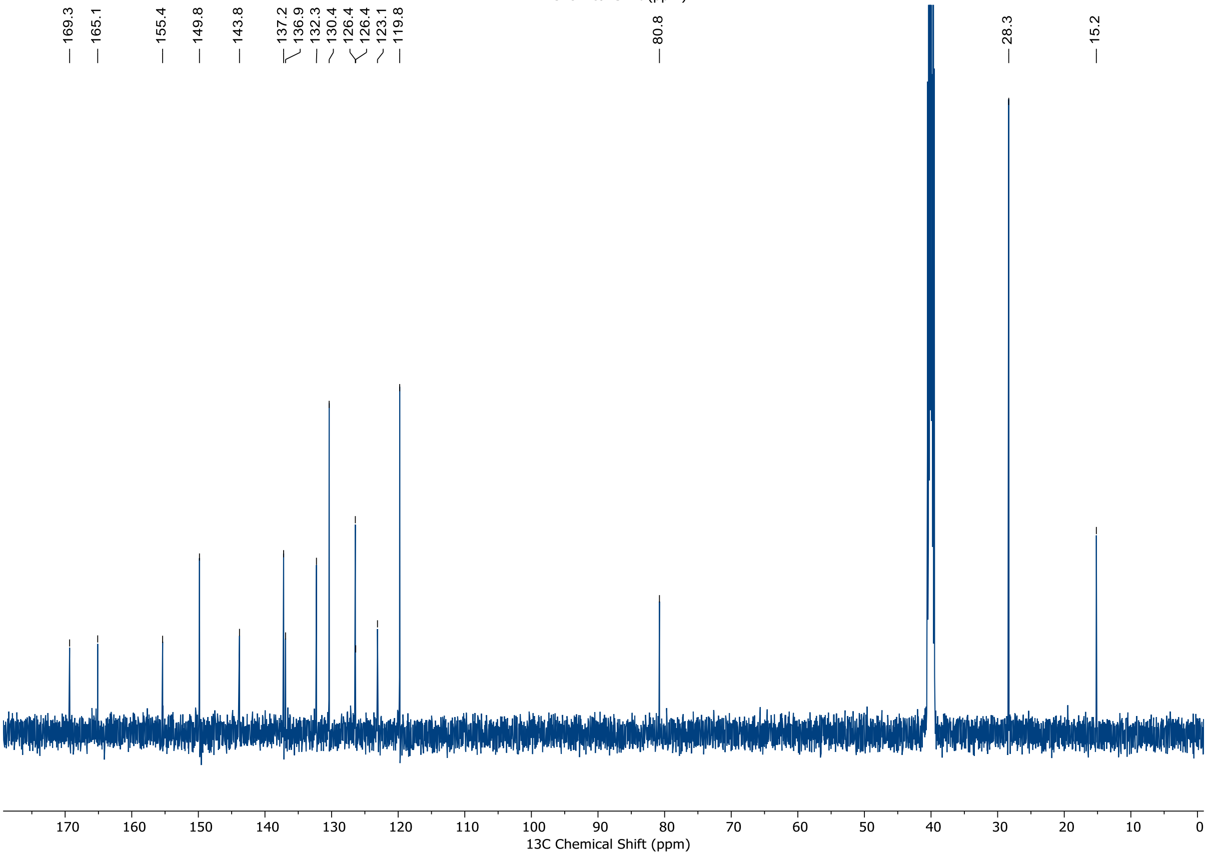

(E)-4-(2-methyl-3-(pyridin-2-yl)acrylamido)benzoic acid (21a)

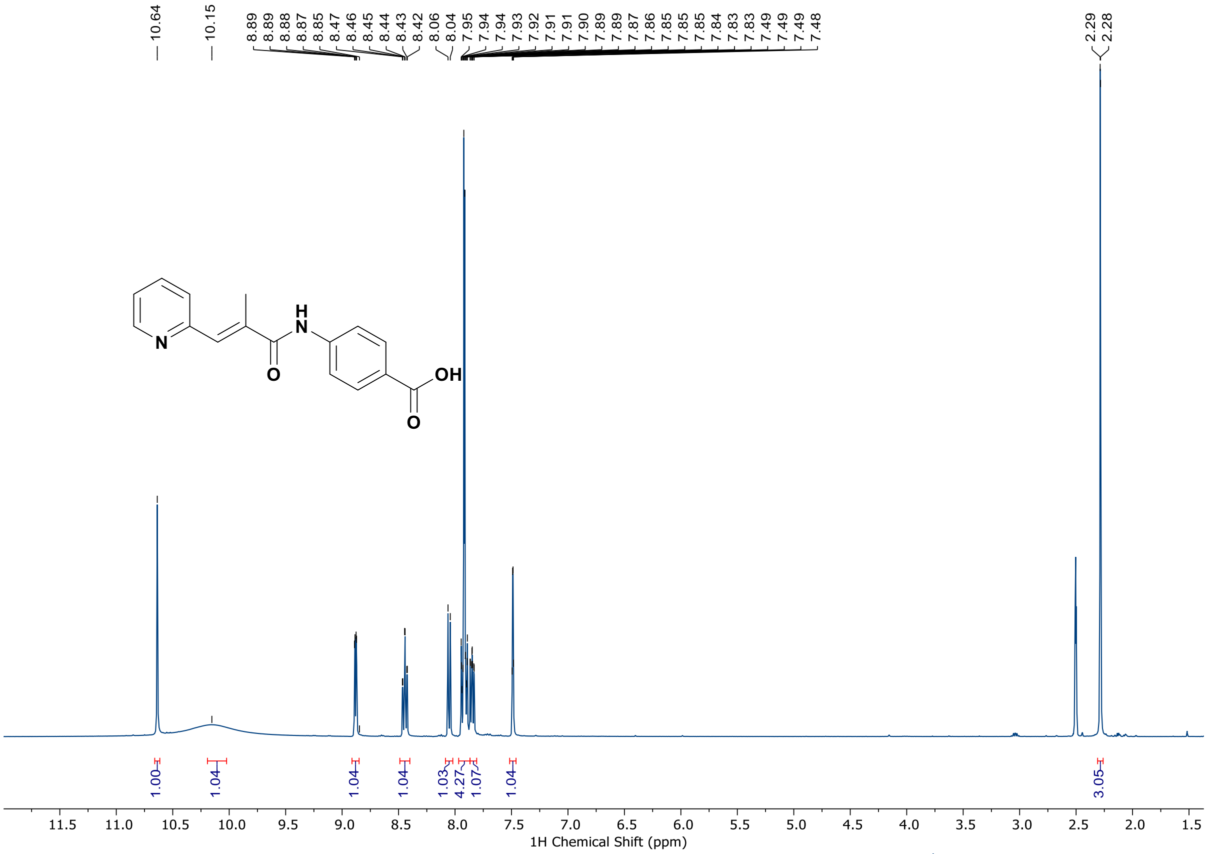

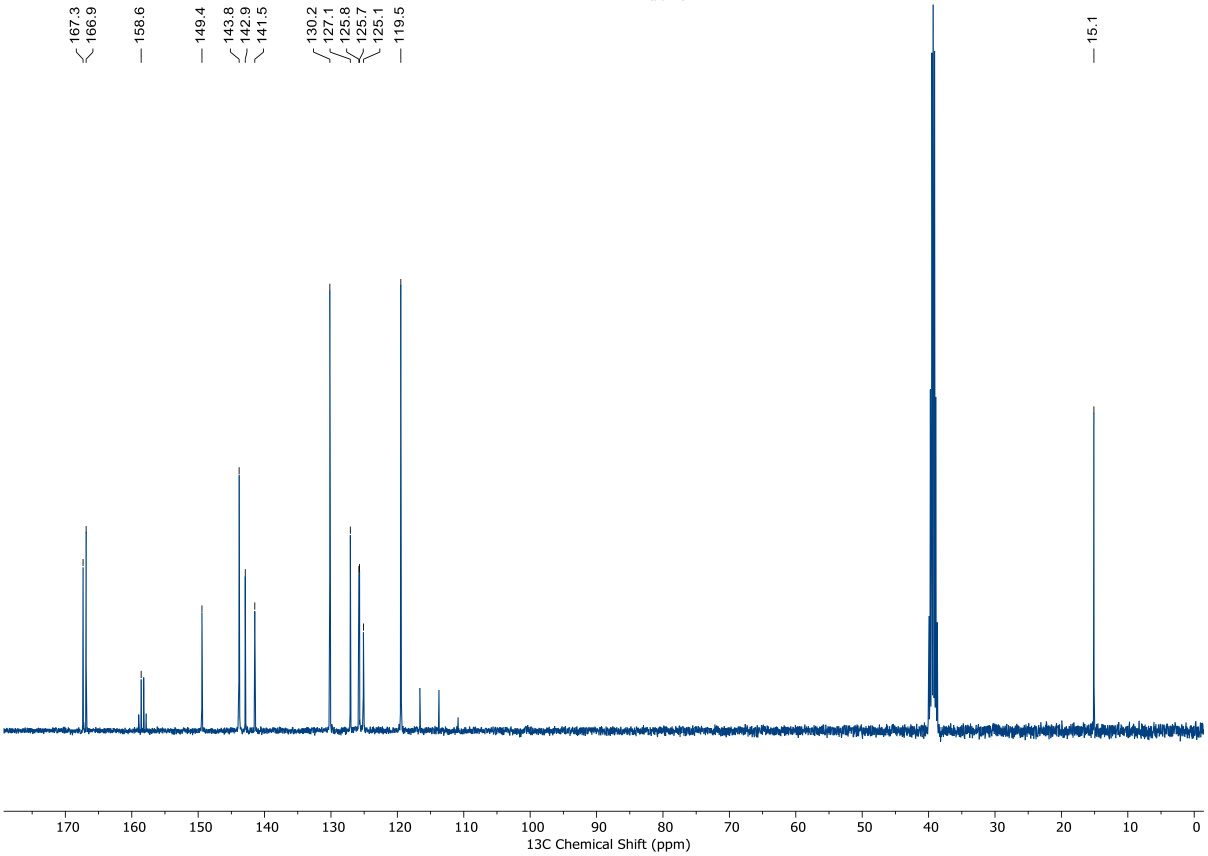

perchlorophenyl (E)-4-(2-methyl-3-(pyridin-2-yl)acrylamido)benzoate (22a)

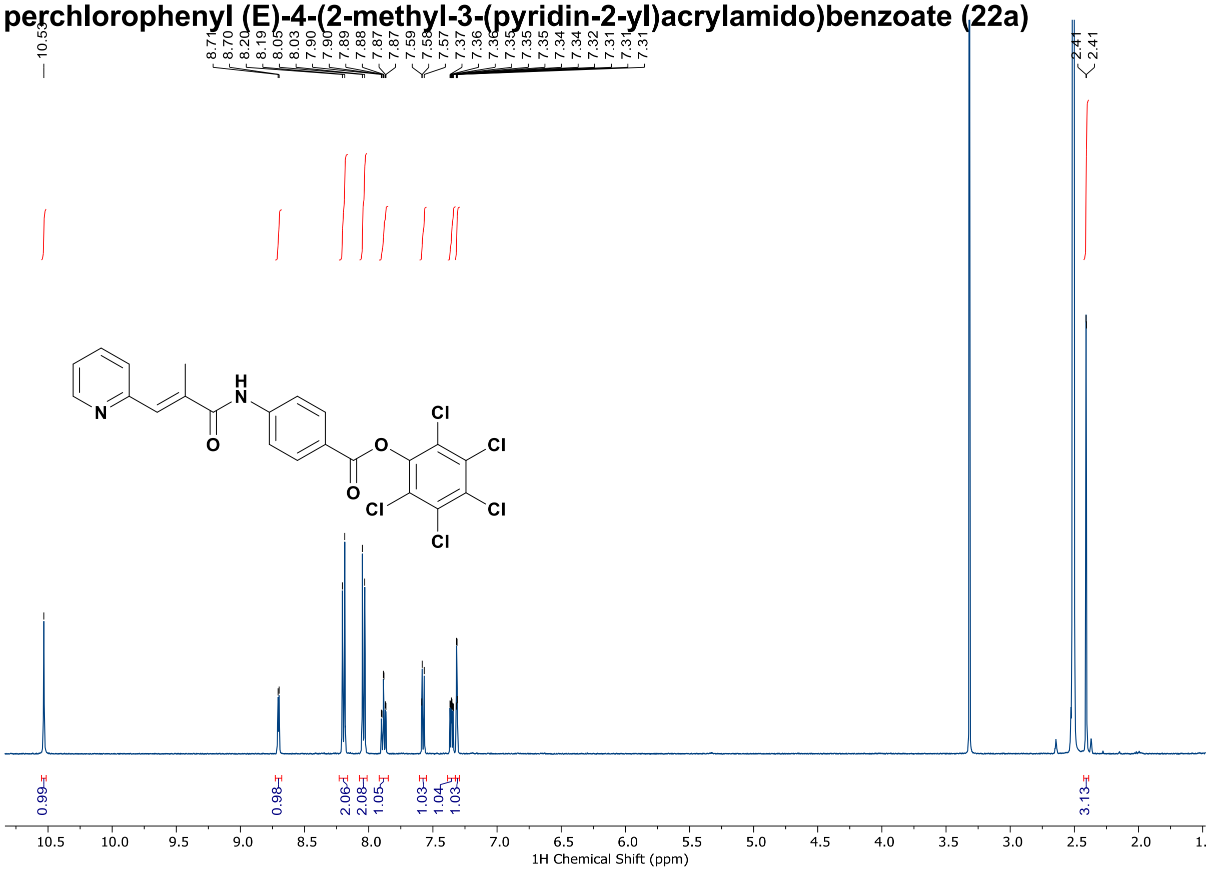

(S)-4-(4-(4-(2-amino-3-(1-((pivaloyloxy)methyl)-1H-1,2,3-triazol-4-yl)propanamido)benzamido)-2-hydroxy-3-methoxybenzamido)-2-hydroxy-3-methoxybenzoic acid (37g)

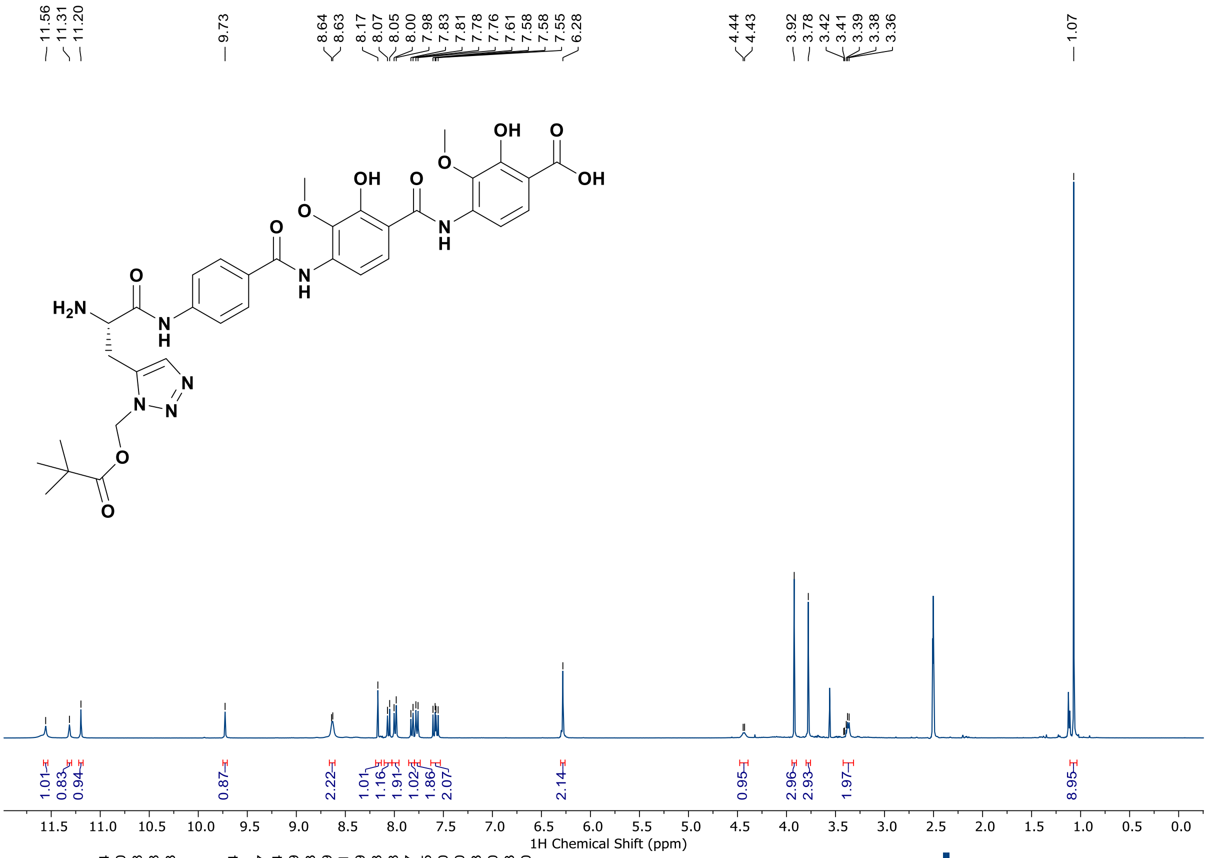

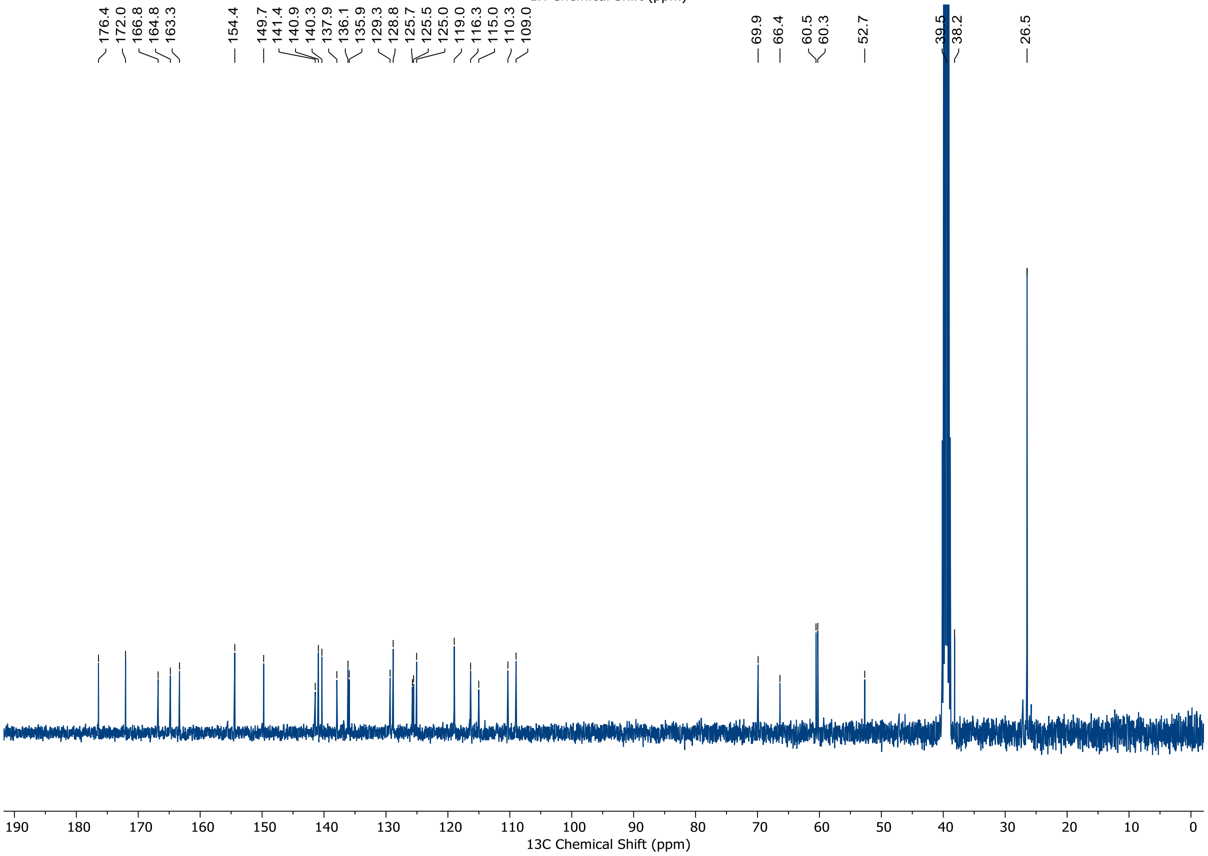

(S)-4-(4-(4-(2-amino-3-(1-((pivaloyloxy)methyl)-1H-1,2,3-triazol-4-yl)propanamido)benzamido)-2-hydroxy-3-methoxybenzamido)-2-hydroxy-3-methoxybenzoic acid (3)

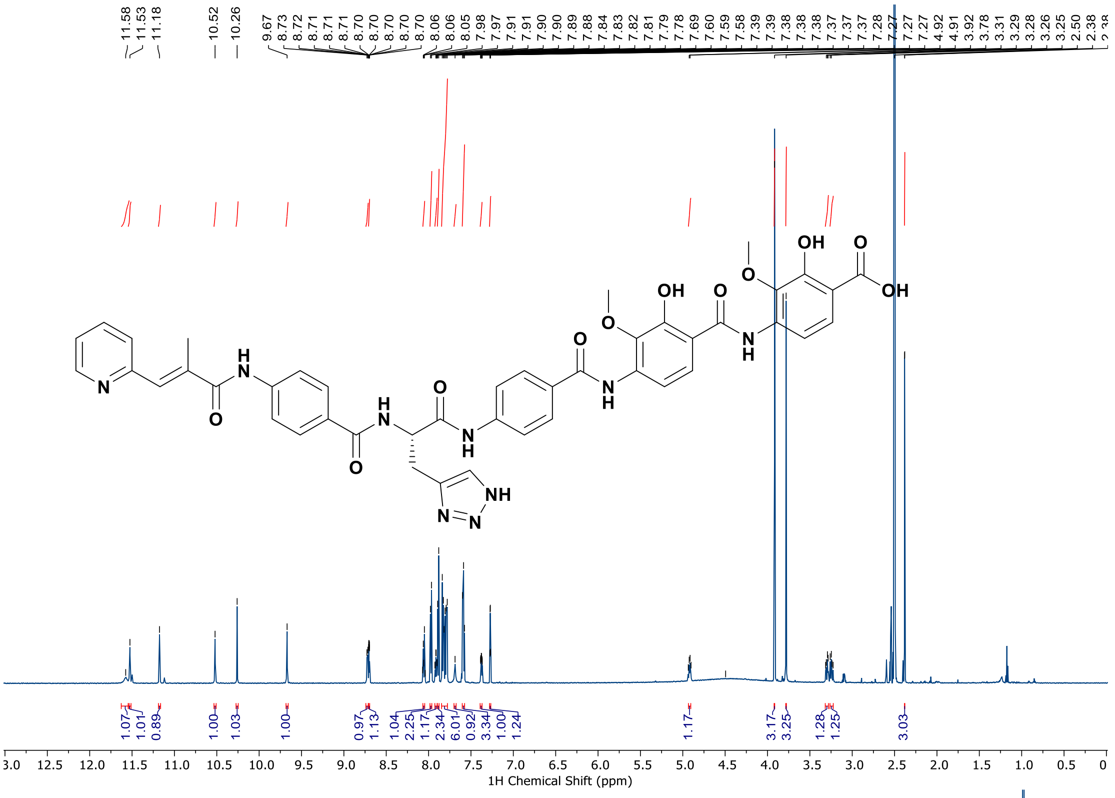

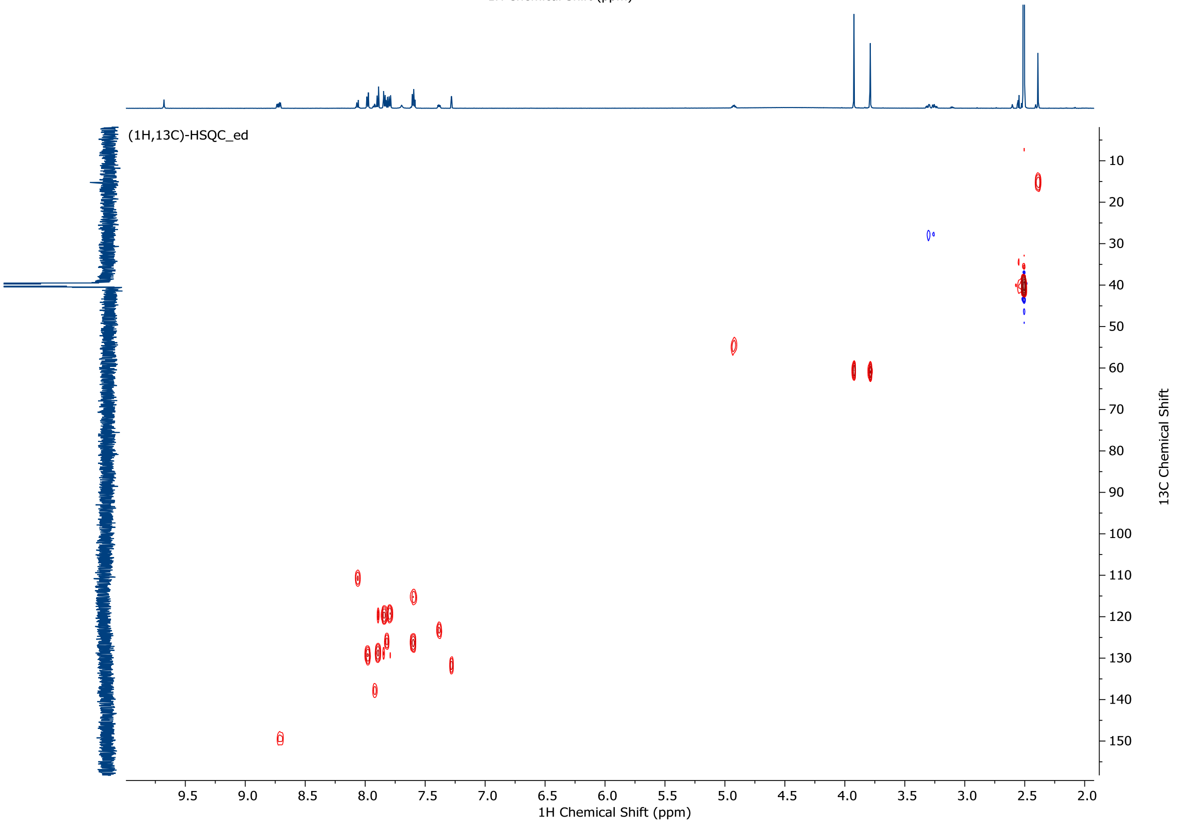

(E)-2-methyl-3-(pyridin-3-yl)acrylic acid (18b)

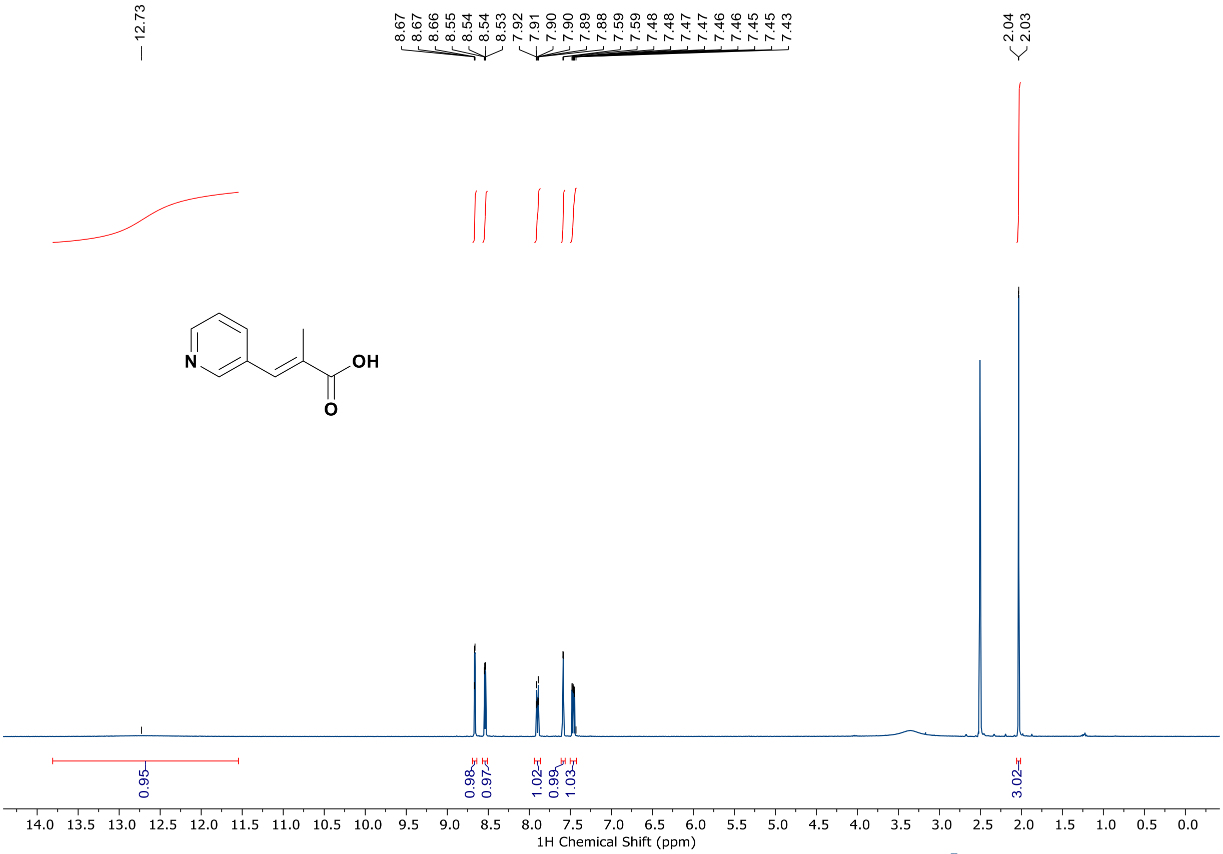

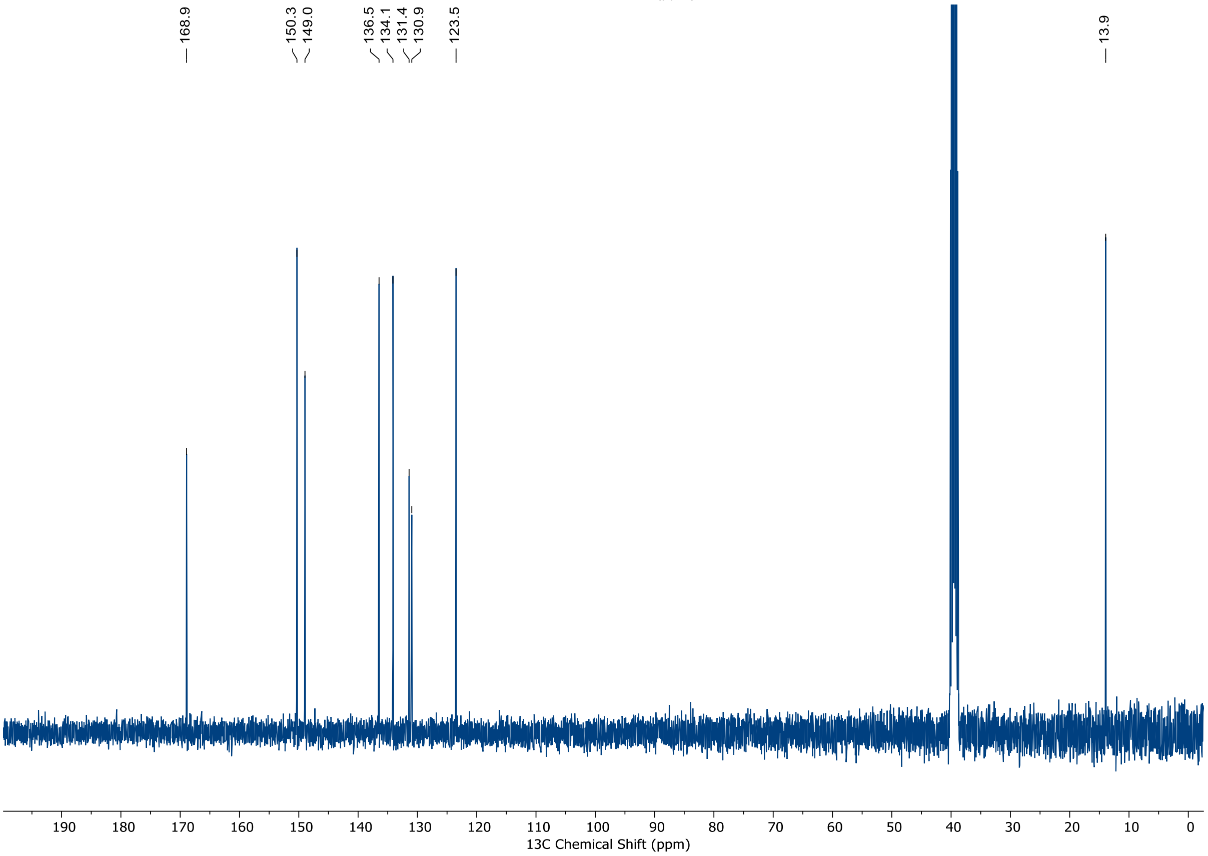

tert-butyl (E)-4-(2-methyl-3-(pyridin-3-yl)acrylamido)benzoate (18b-2)

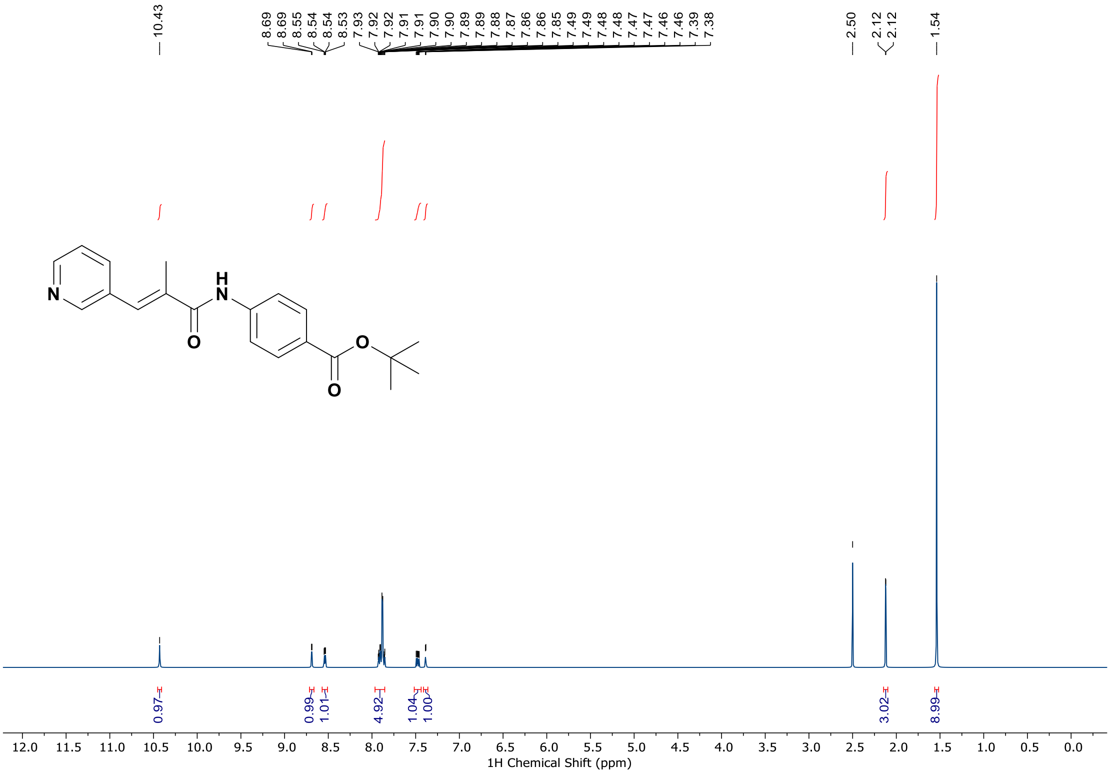

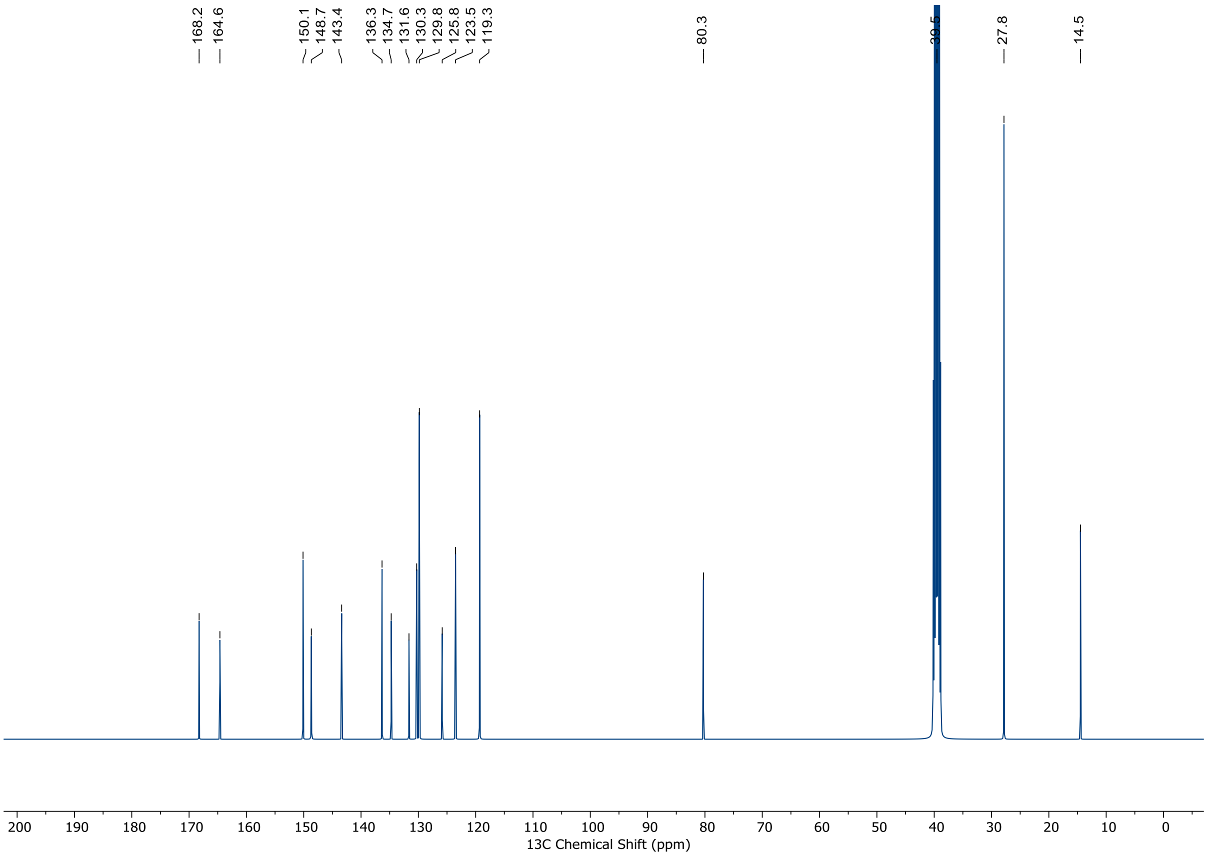

(E)-4-(2-methyl-3-(pyridin-3-yl)acrylamido)benzoic acid (21b)

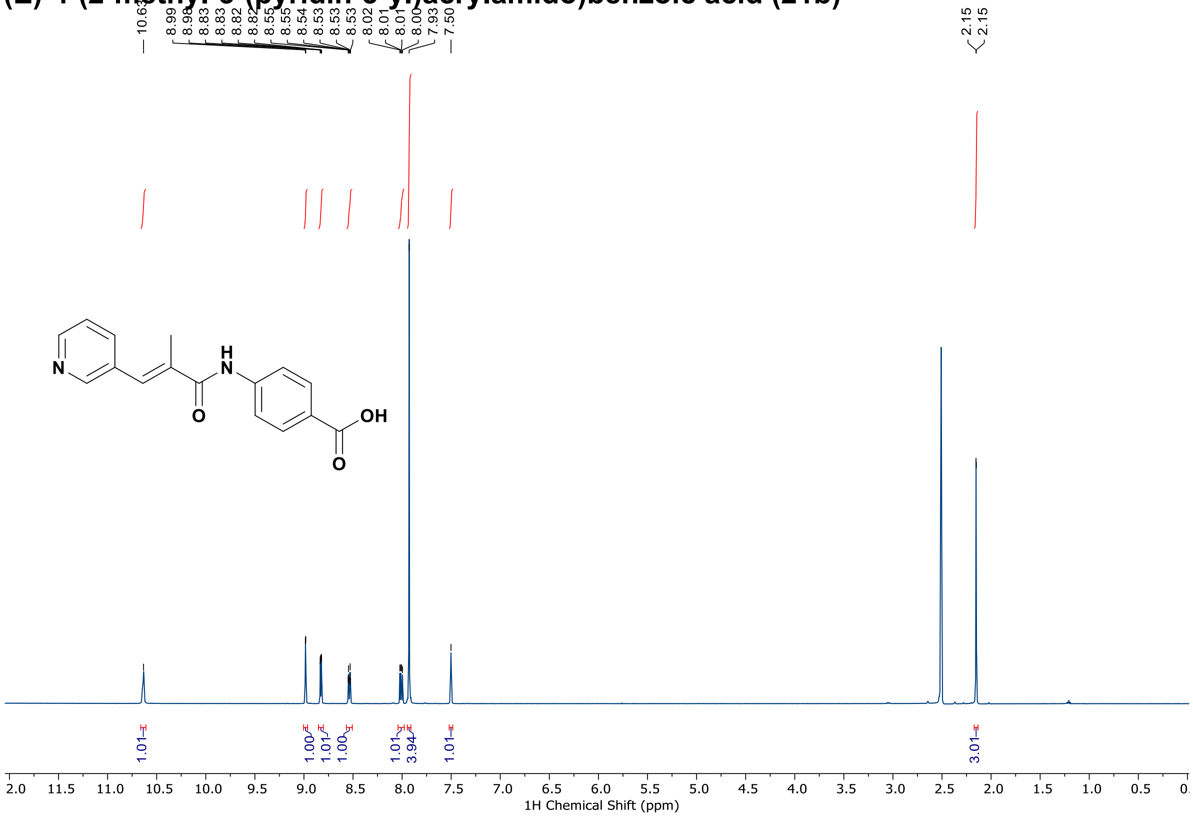

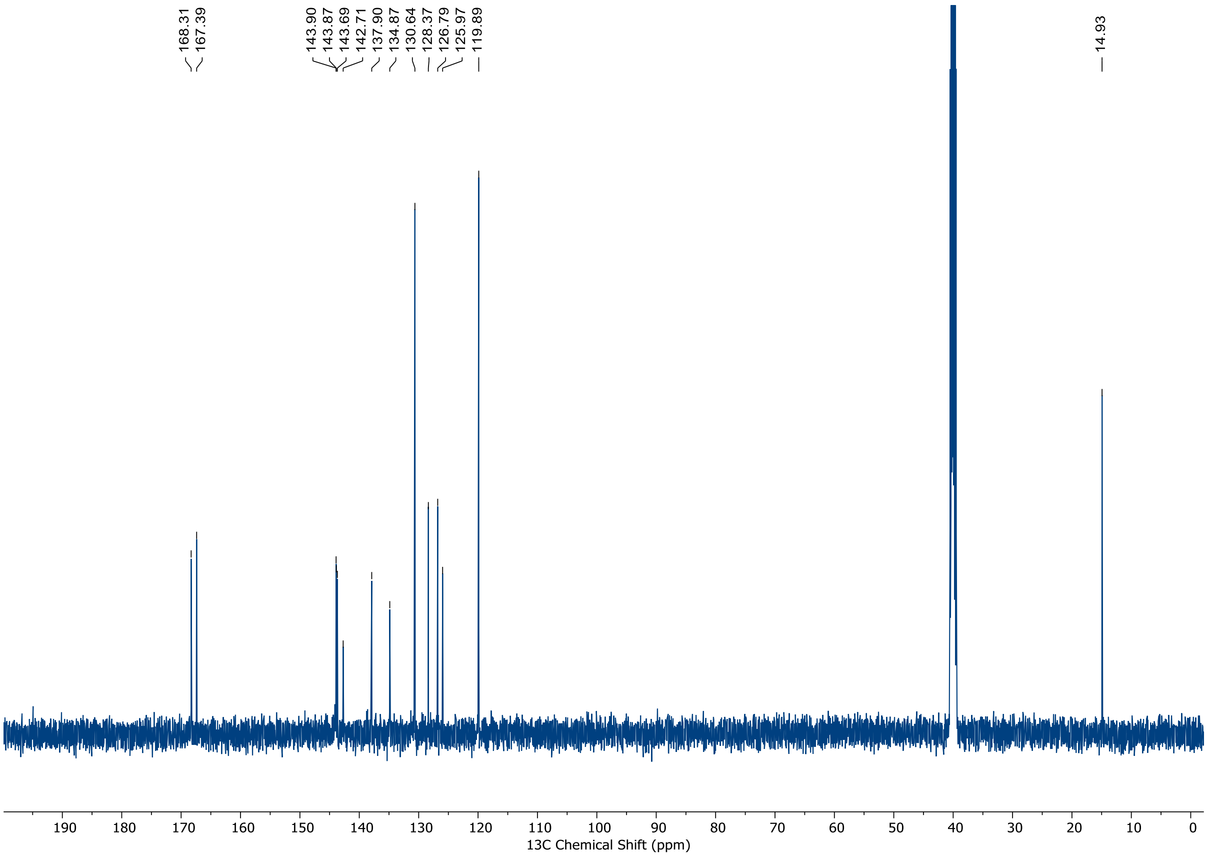

perchlorophenyl (E)-4-(2-methyl-3-(pyridin-3-yl)acrylamido)benzoate (22b)

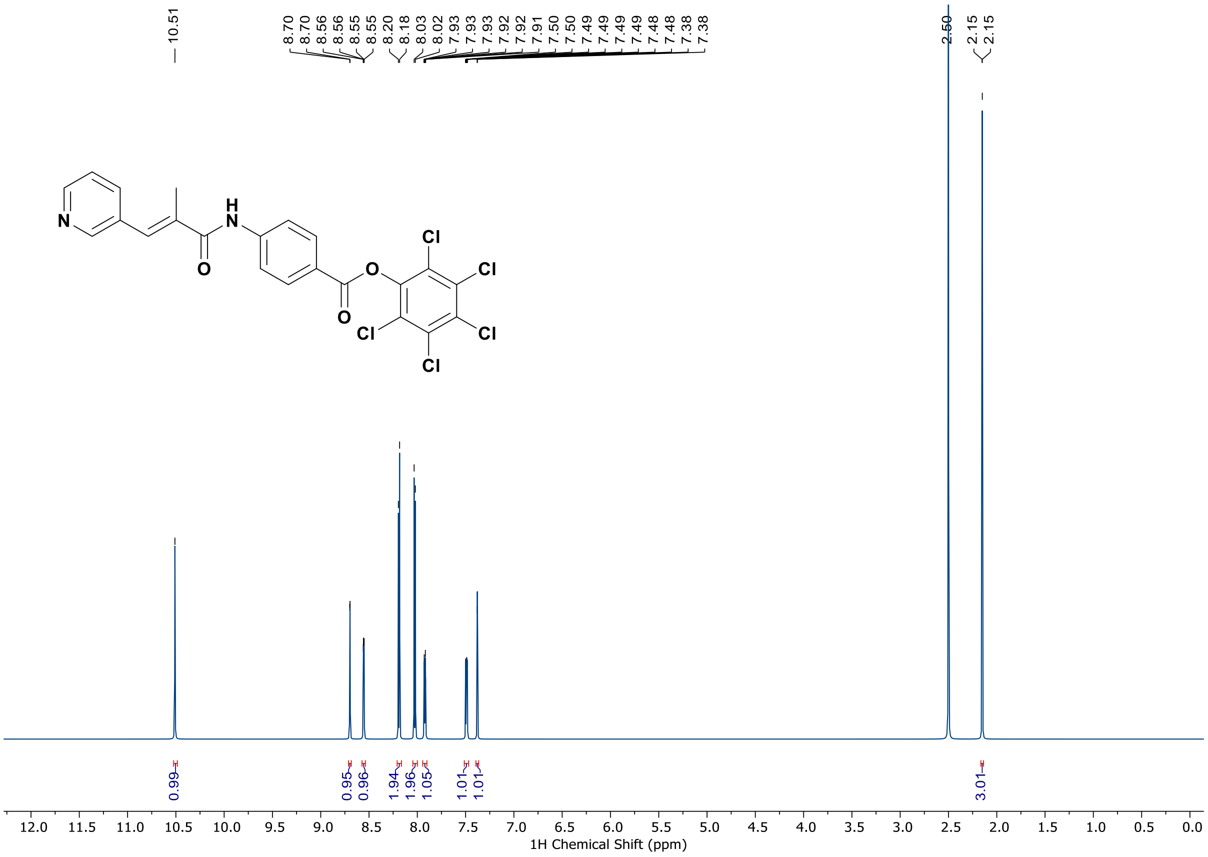

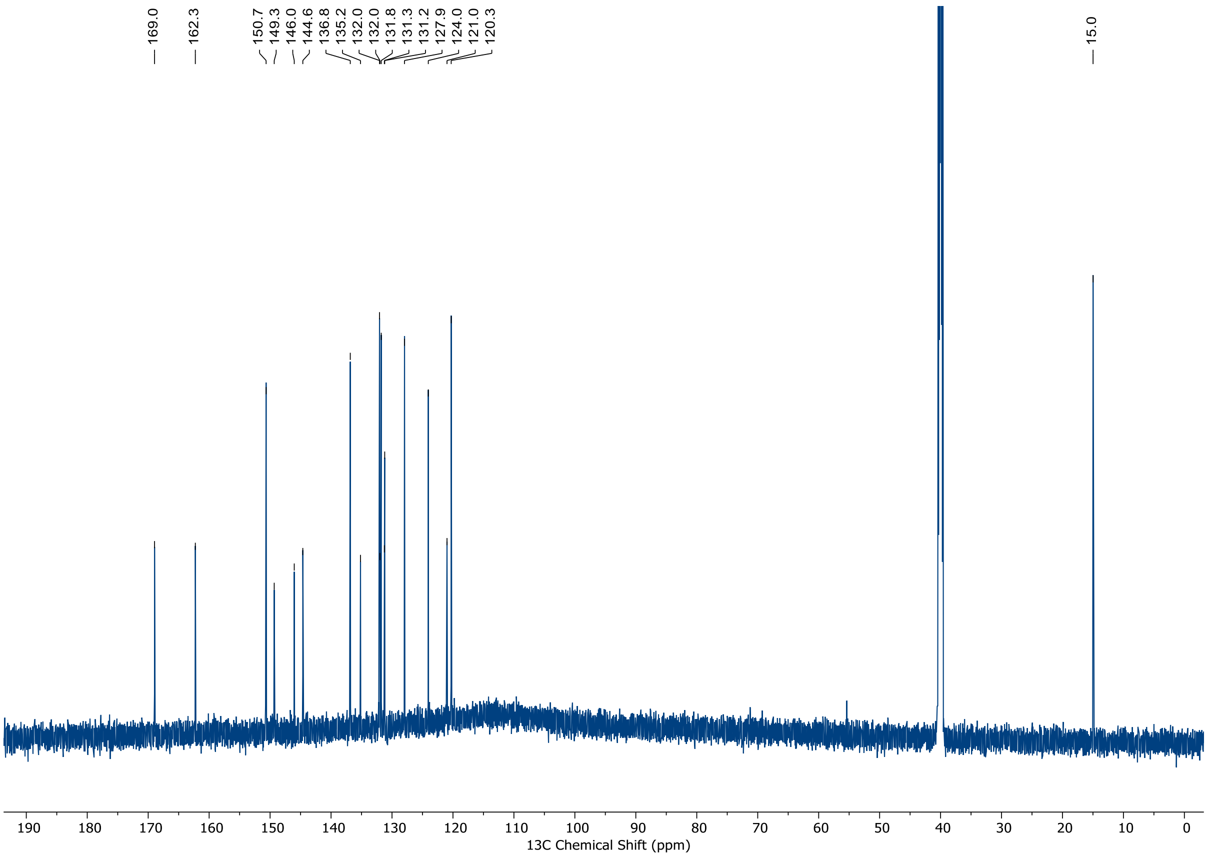

(S,E)-2-hydroxy-4-(2-hydroxy-3-methoxy-4-(4-(2-(4-(2-methyl-3-(pyridin-3-yl)acrylamido)benzamido)-3-(1H-1,2,3-triazol-4-yl)propanamido)benzamido)benzamido)-3-methoxybenzoic acid (4)

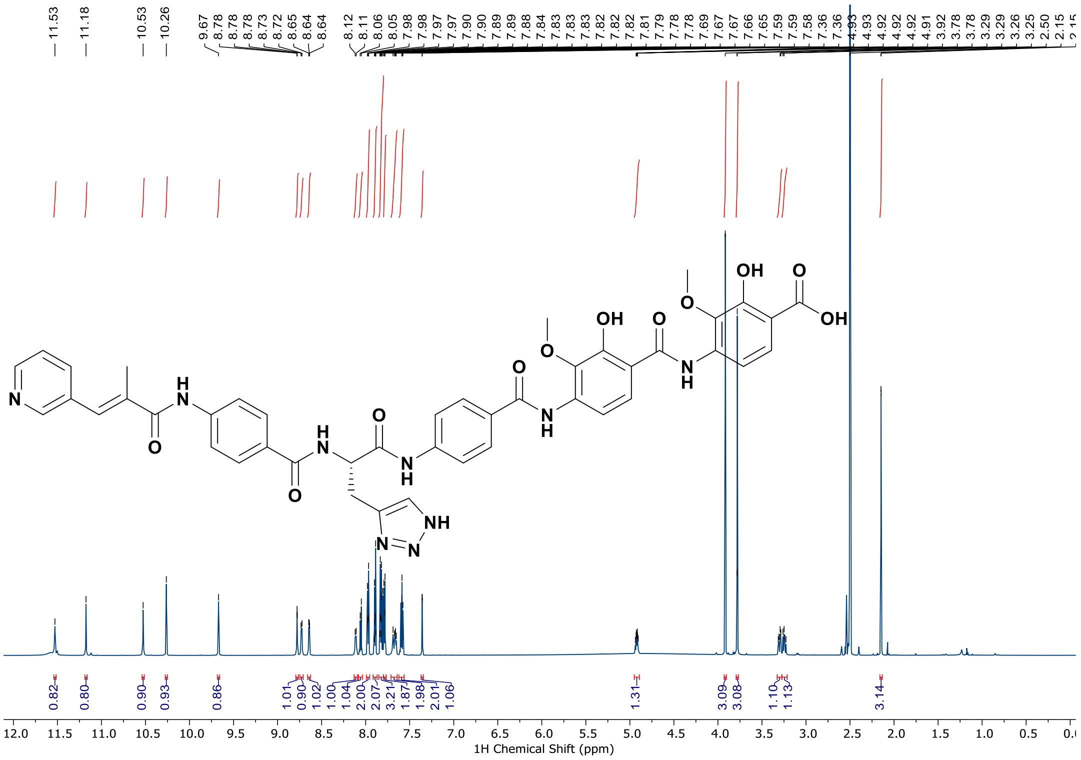

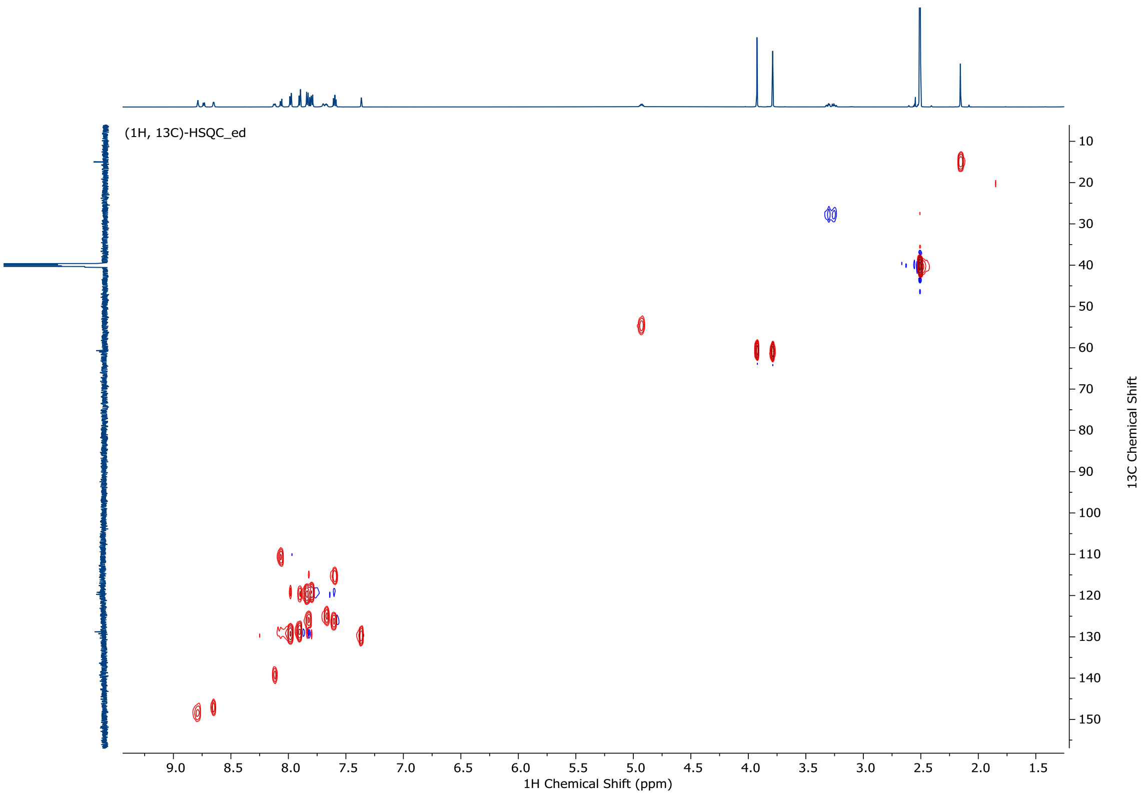

3.2 NMR spectra of B-Variations (5, 6) and reaction intermediates

(E)-3-(4-acetoxyphenyl)-2-methylacrylic acid (19)

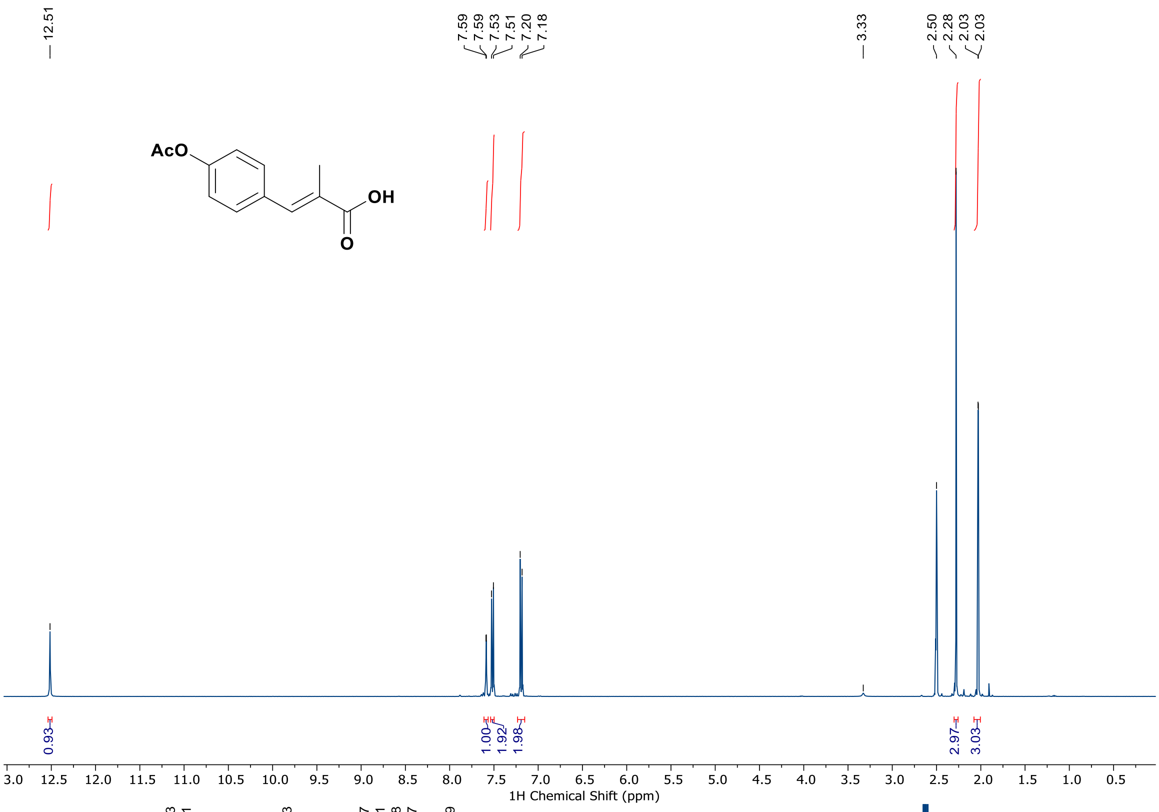

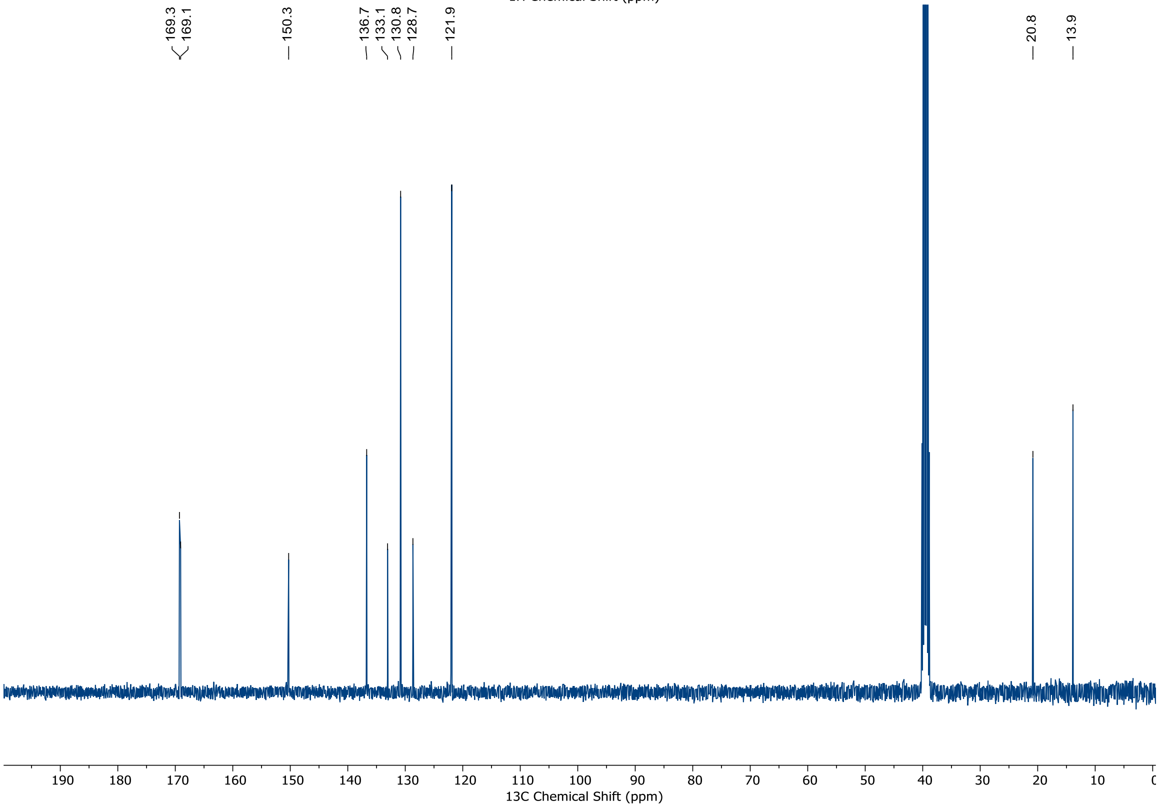

methyl (E)-6-(3-(4-acetoxyphenyl)-2-methylacrylamido)nicotinate (20c-1)

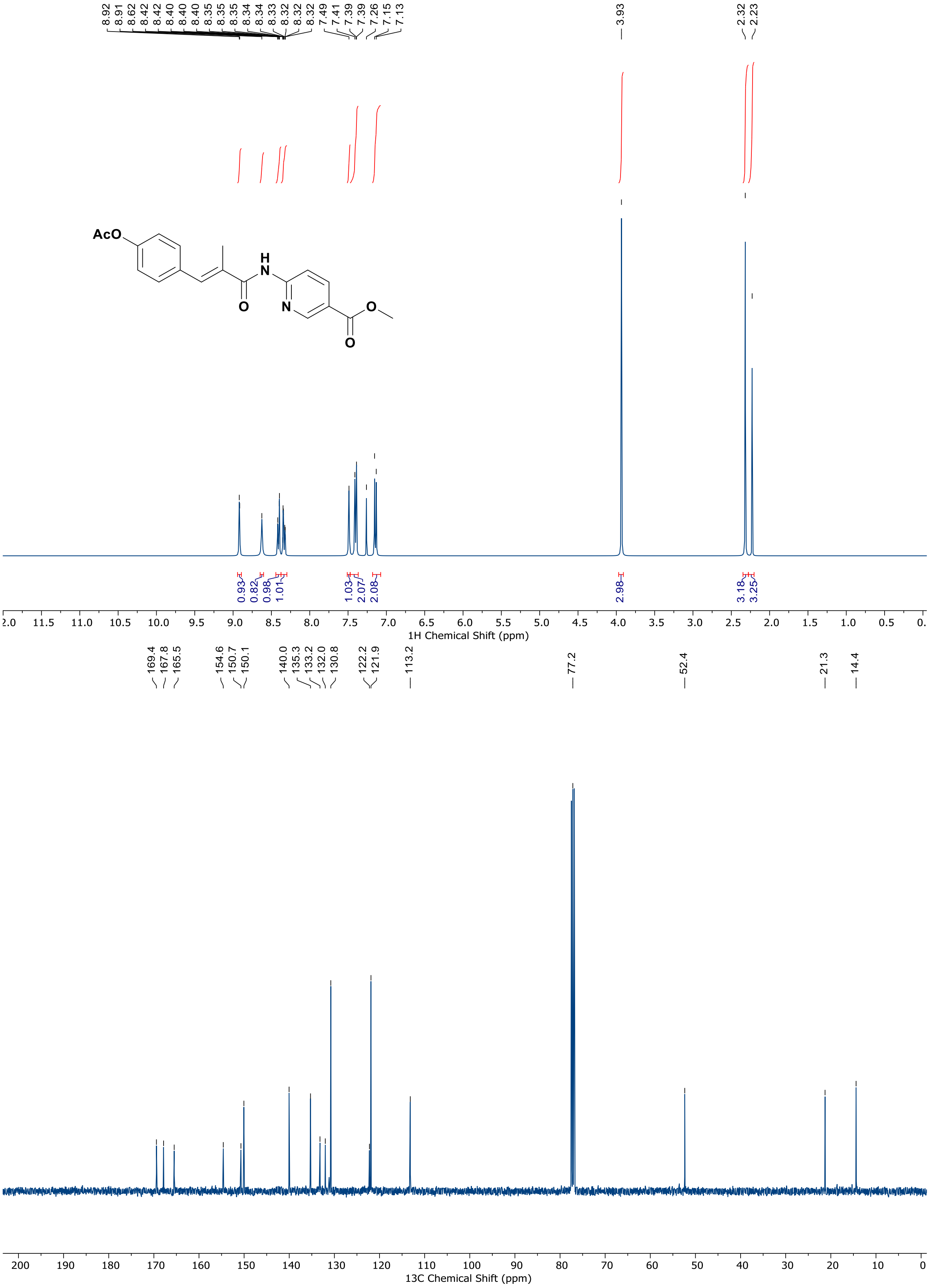

(E)-6-(3-(4-hydroxyphenyl)-2-methylacrylamido)nicotinic acid (20c-2)

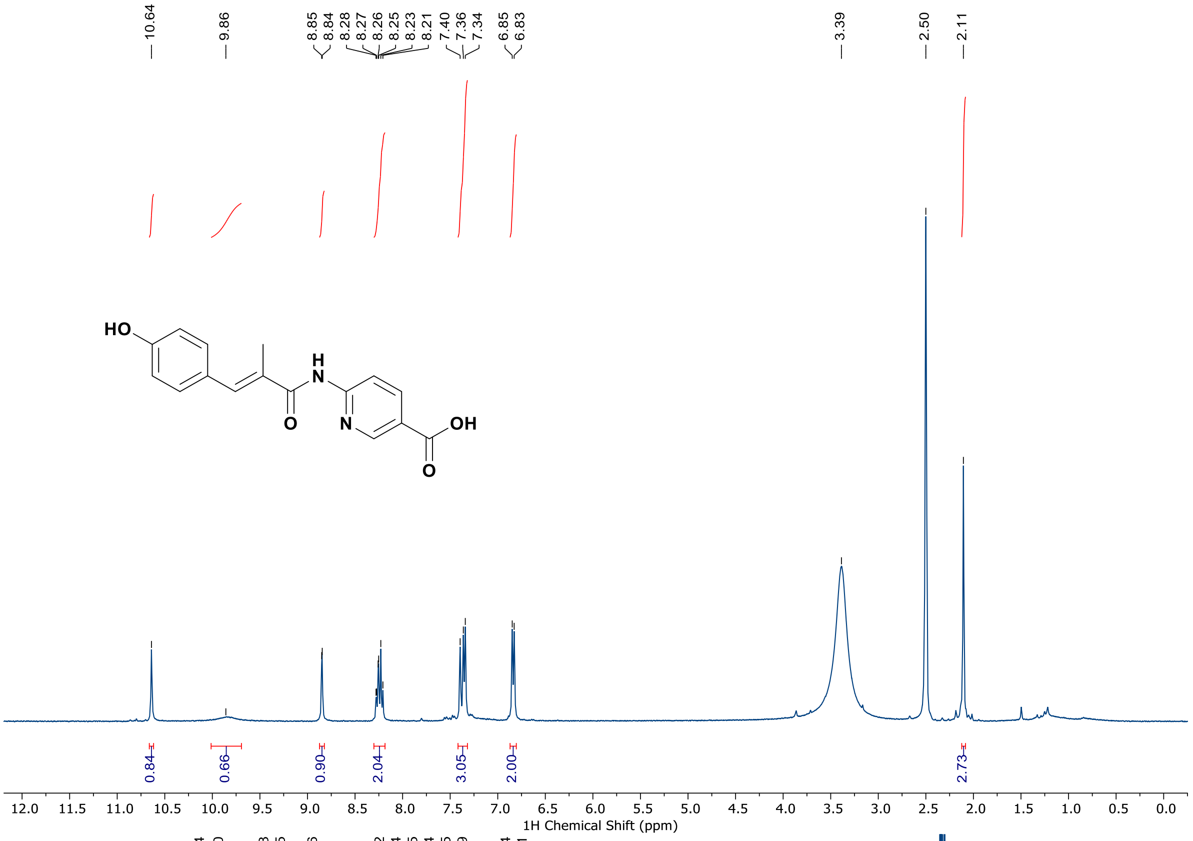

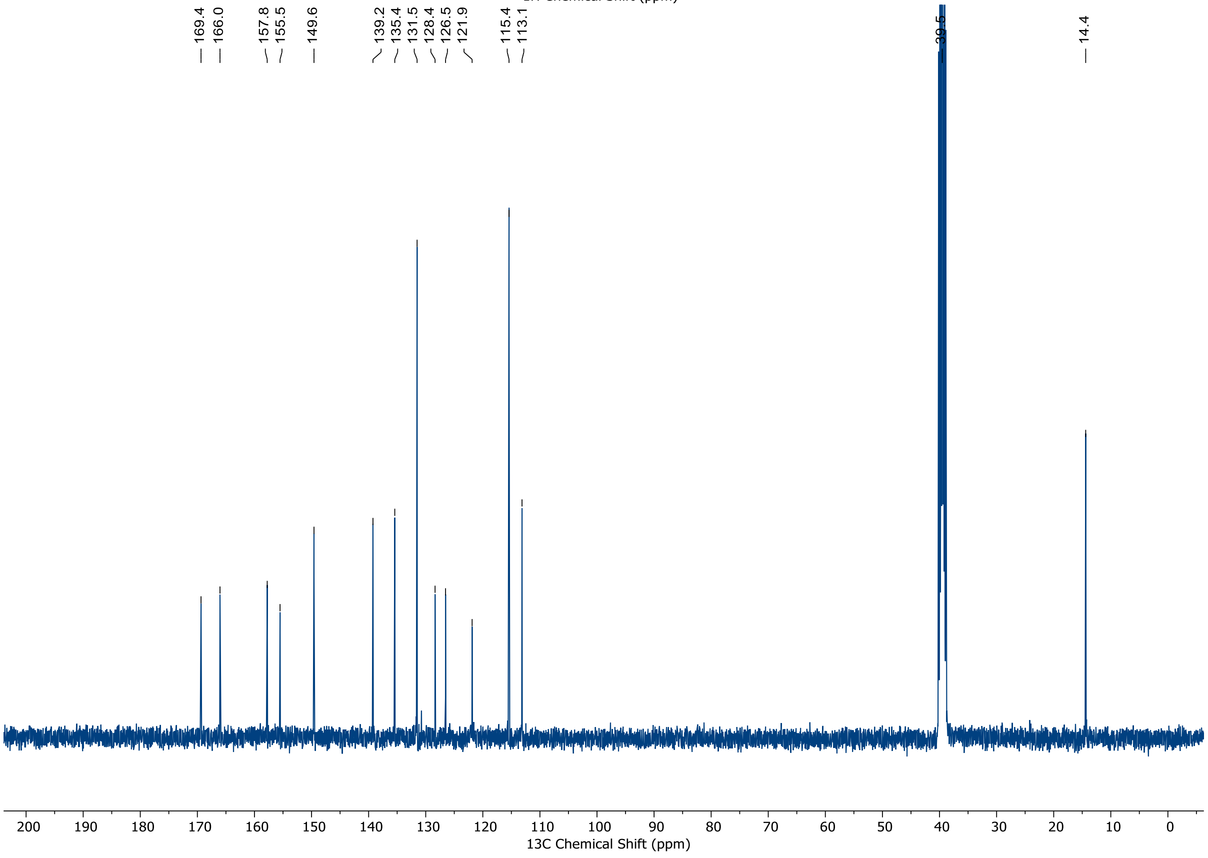

(E)-6-(3-(4-((tert-butoxycarbonyl)oxy)phenyl)-2-methylacrylamido)nicotinic (21c)

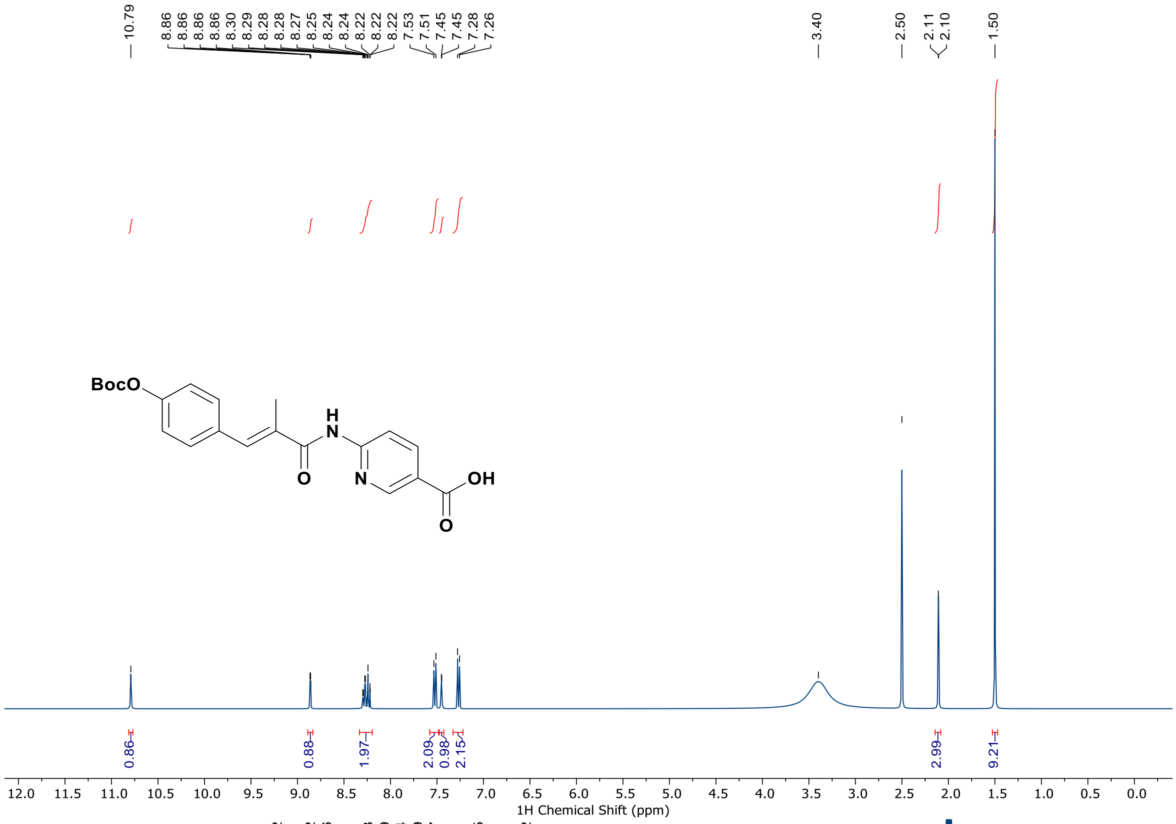

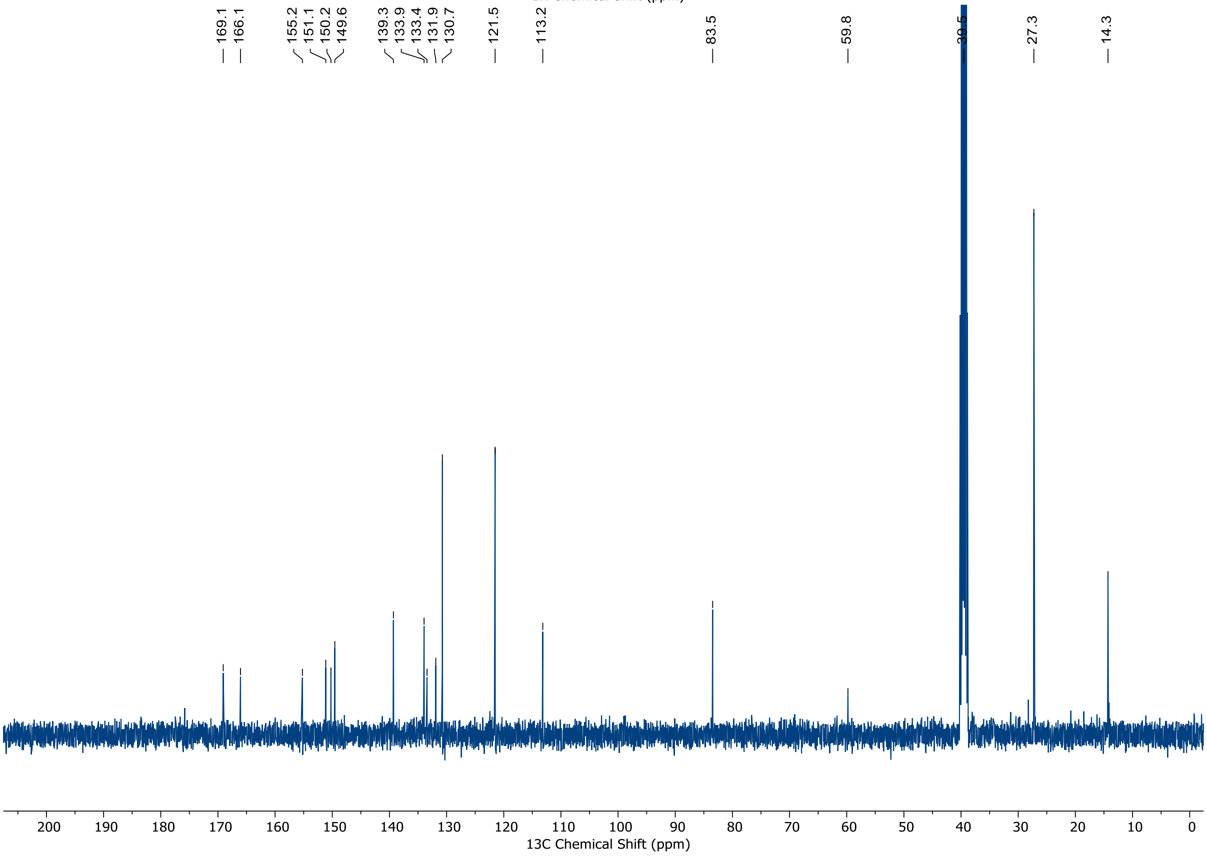

perchlorophenyl (E)-6-(3-(4-((tert-butoxycarbonyl)oxy)phenyl)-2-methylacrylamido)nicotinate (22c)

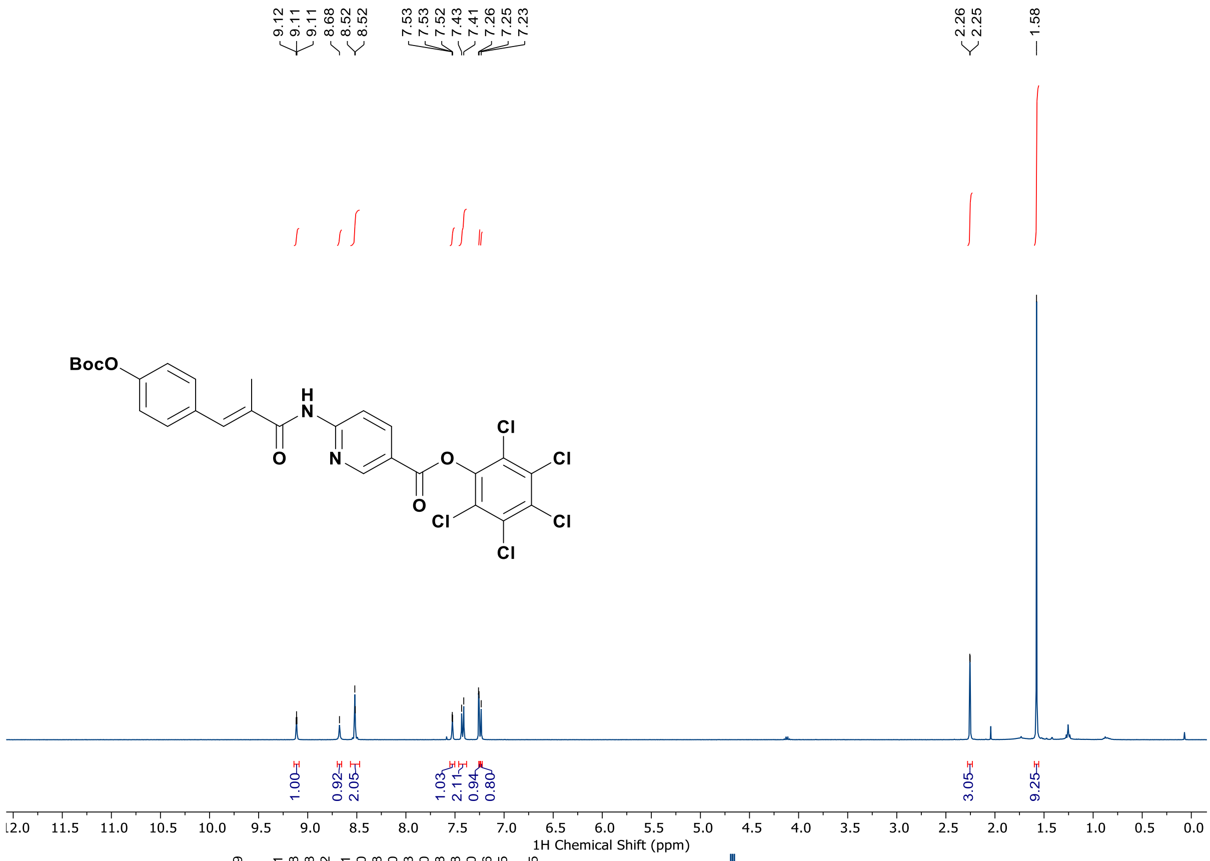

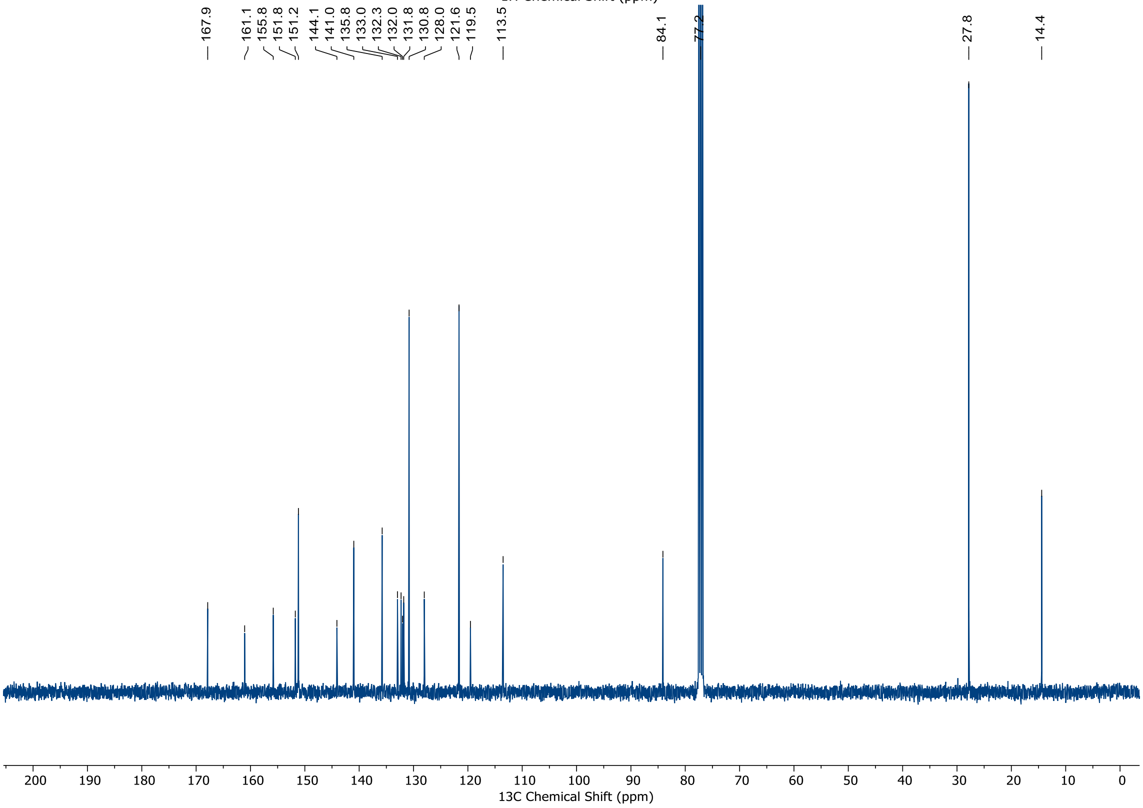

(S,E)-2-hydroxy-4-(2-hydroxy-4-(4-(2-(6-(3-(4-hydroxyphenyl)-2-methylacrylamido)nicotinamido)-3-(1H-1,2,3-triazol-4-yl)propanamido)benzamido)-3-methoxybenzamido)-3-methoxybenzoic acid (5)

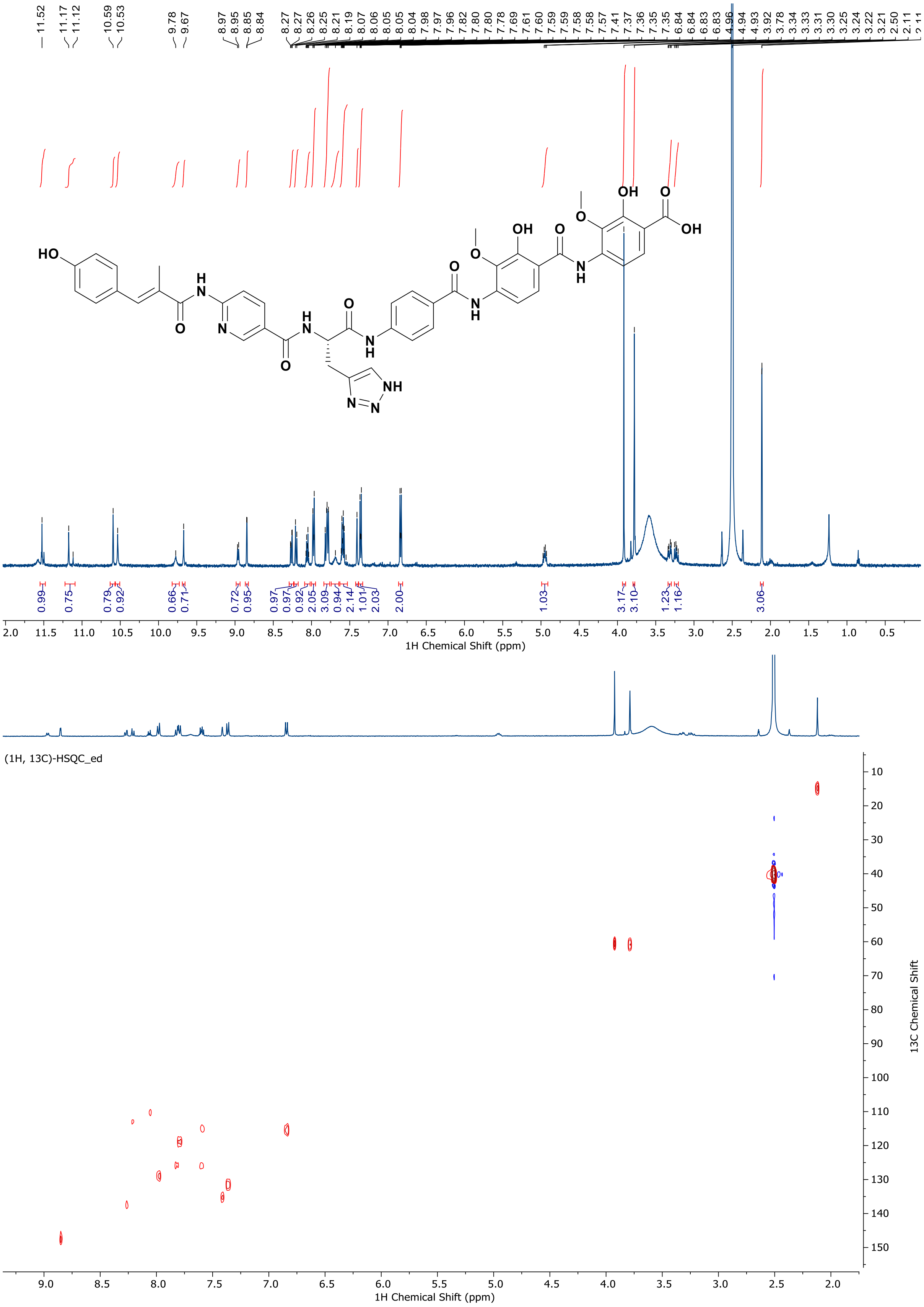

methyl (E)-5-(3-(4-acetoxyphenyl)-2-methylacrylamido)picolinate (20d-1)

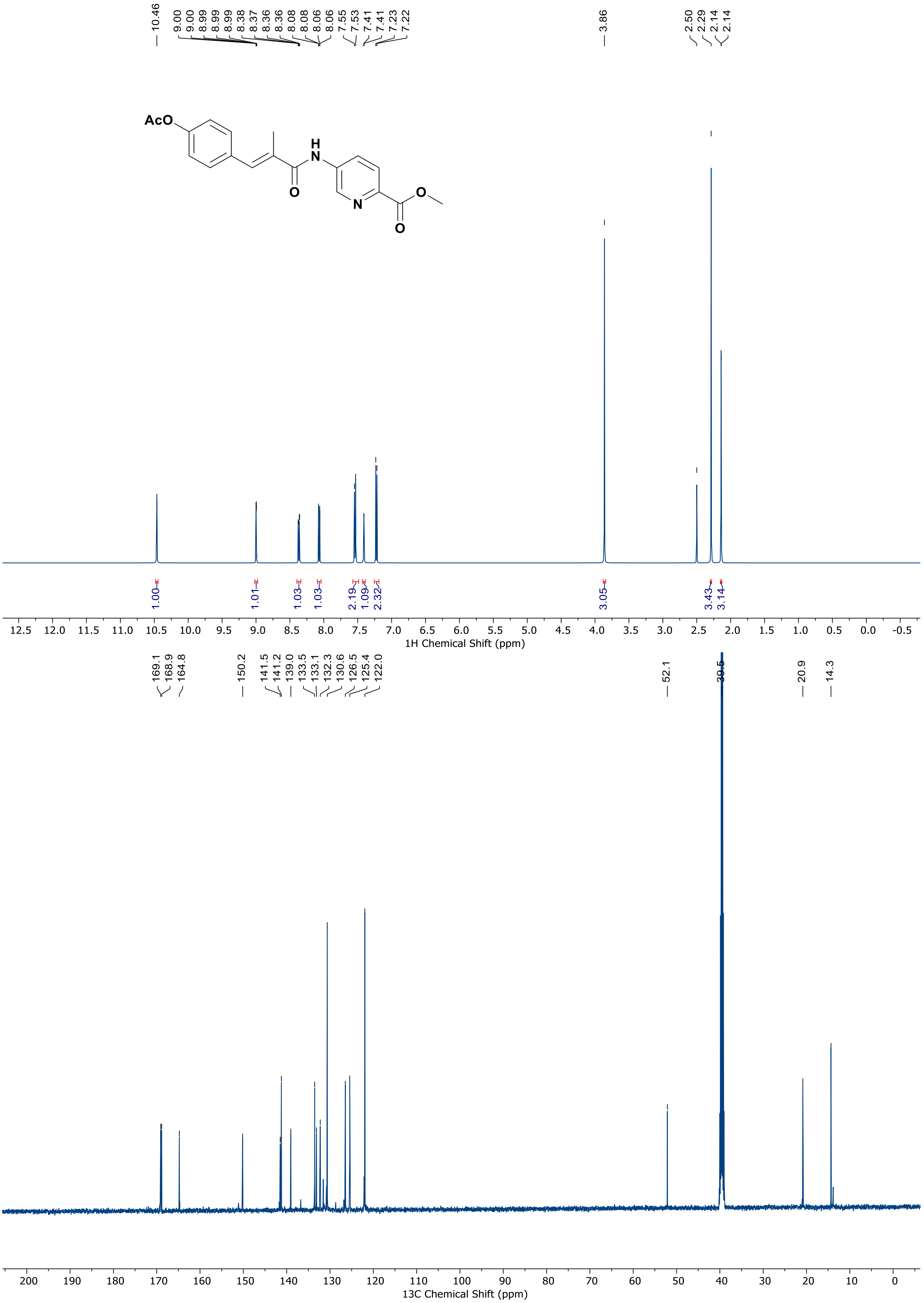

(E)-5-(3-(4-hydroxyphenyl)-2-methylacrylamido)picolinic acid (20d-2)

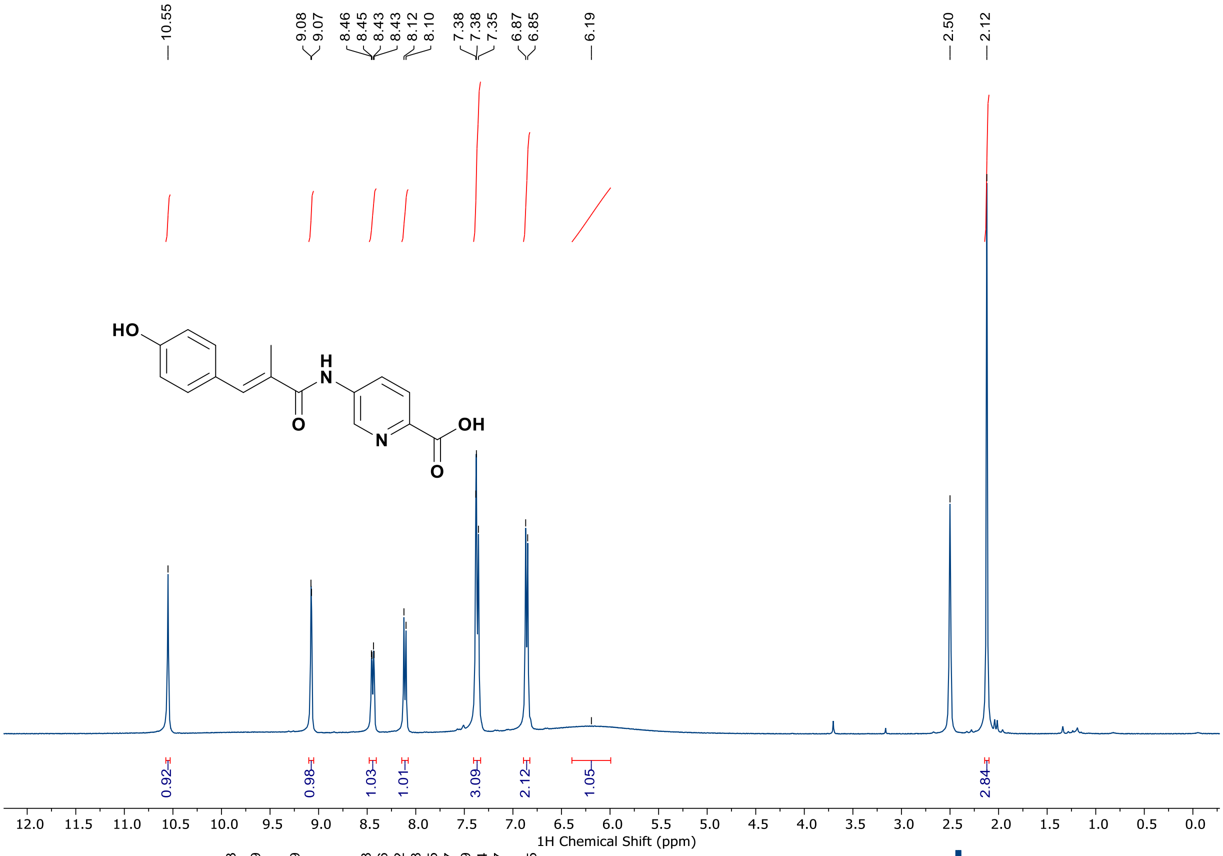

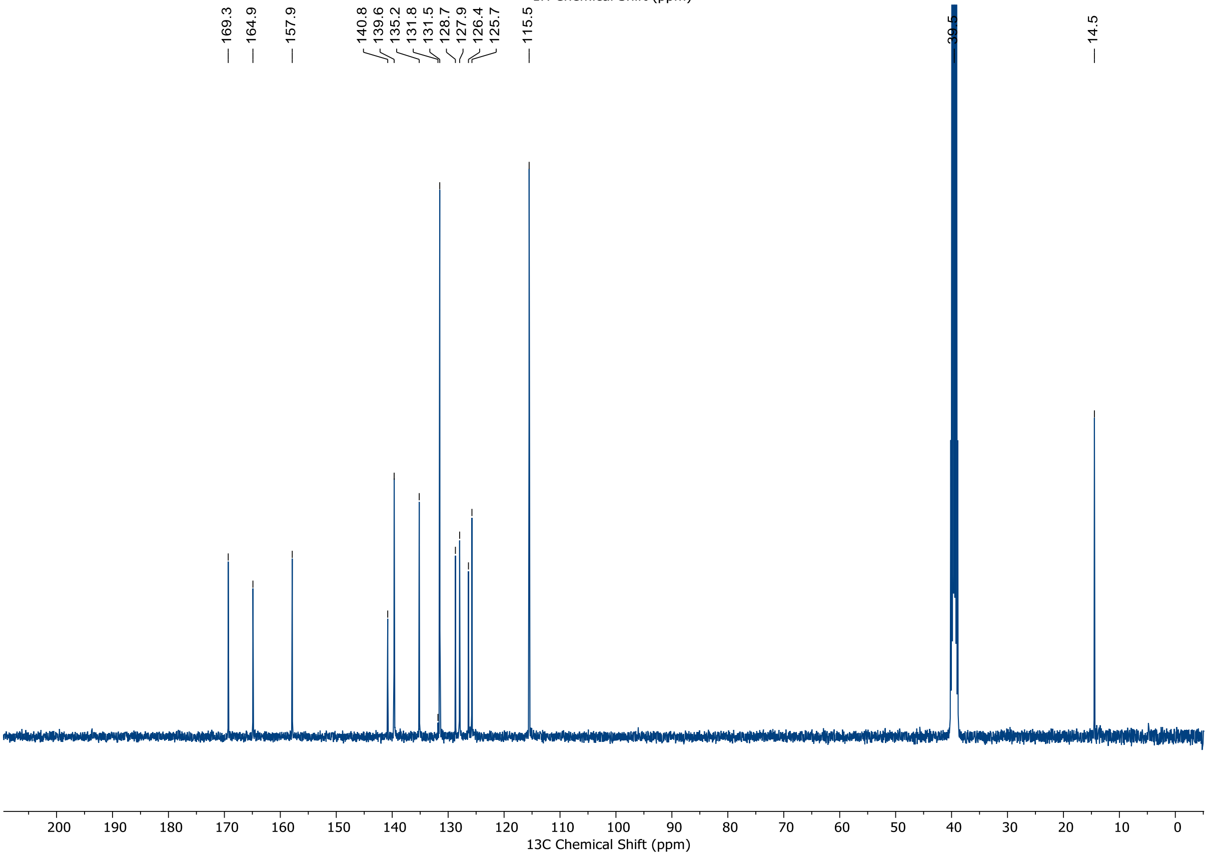

(E)-5-(3-(4-((tert-butoxycarbonyl)oxy)phenyl)-2-methylacrylamido)picolinic acid (21d)

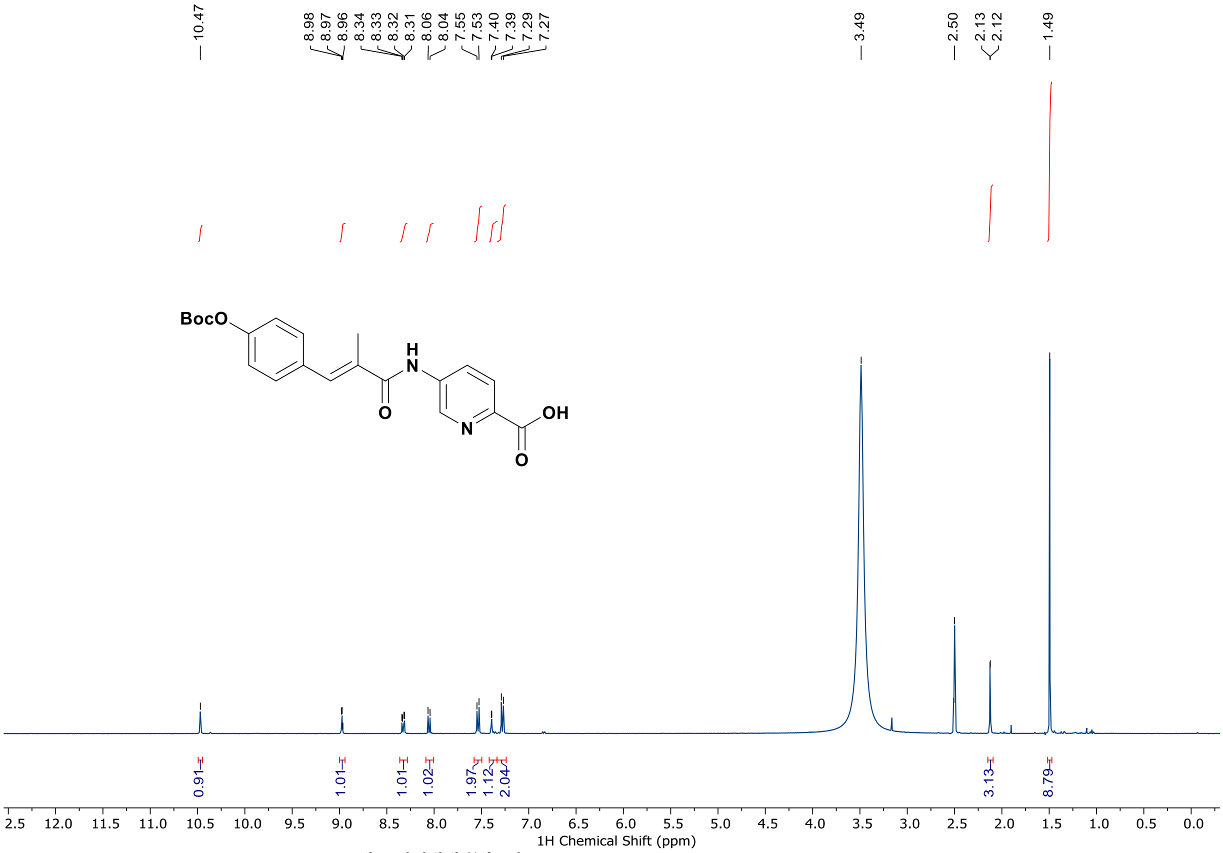

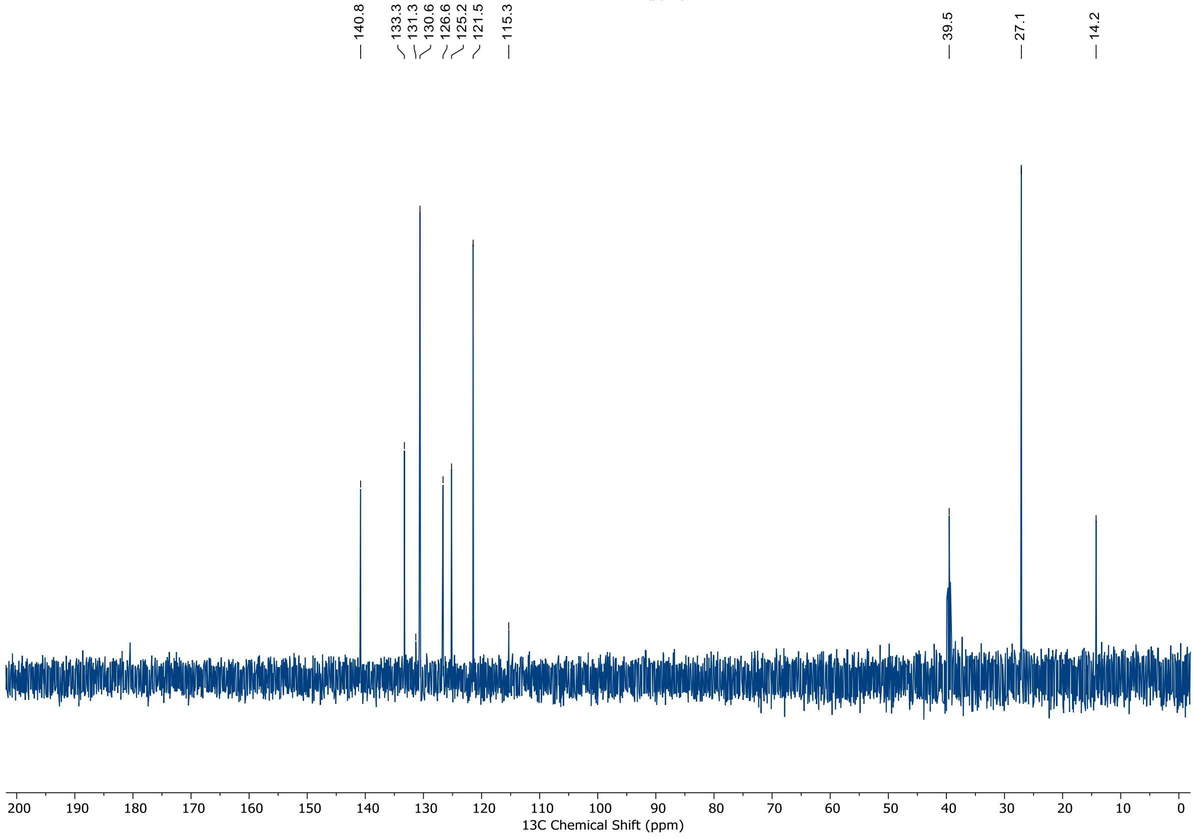

perchlorophenyl (E)-5-(3-(4-((tert-butoxycarbonyl)oxy)phenyl)-2-methylacrylamido)picolinate (22d)

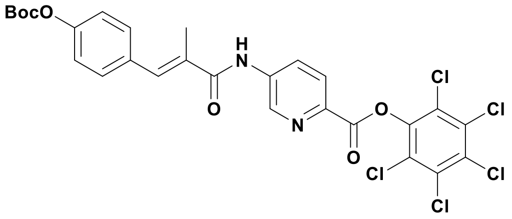

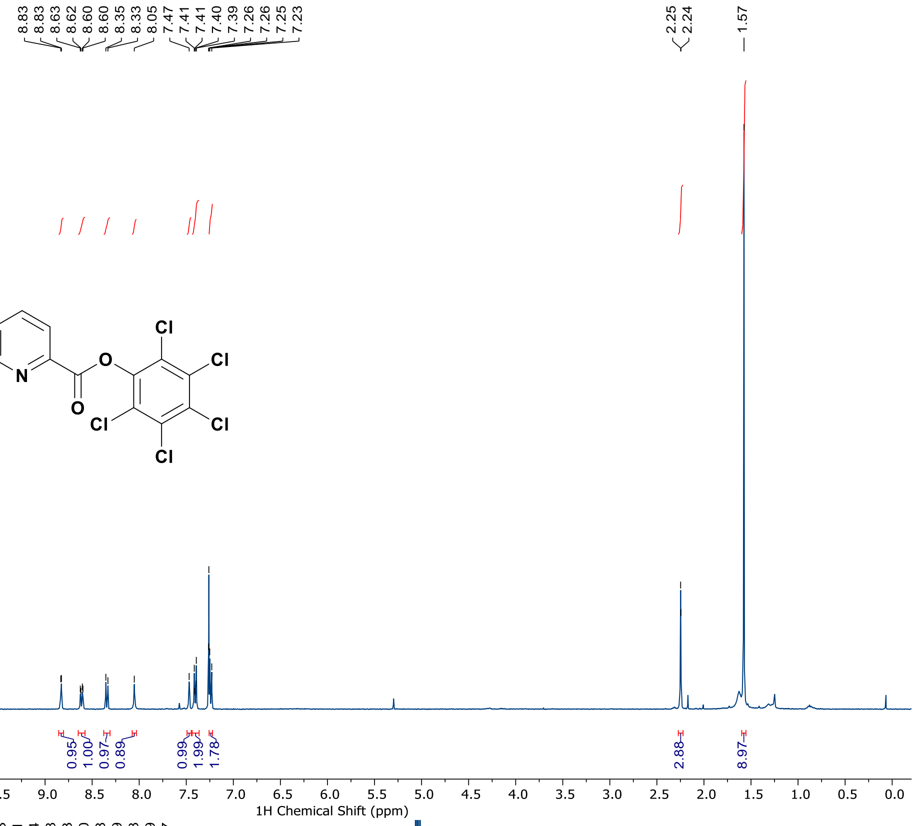

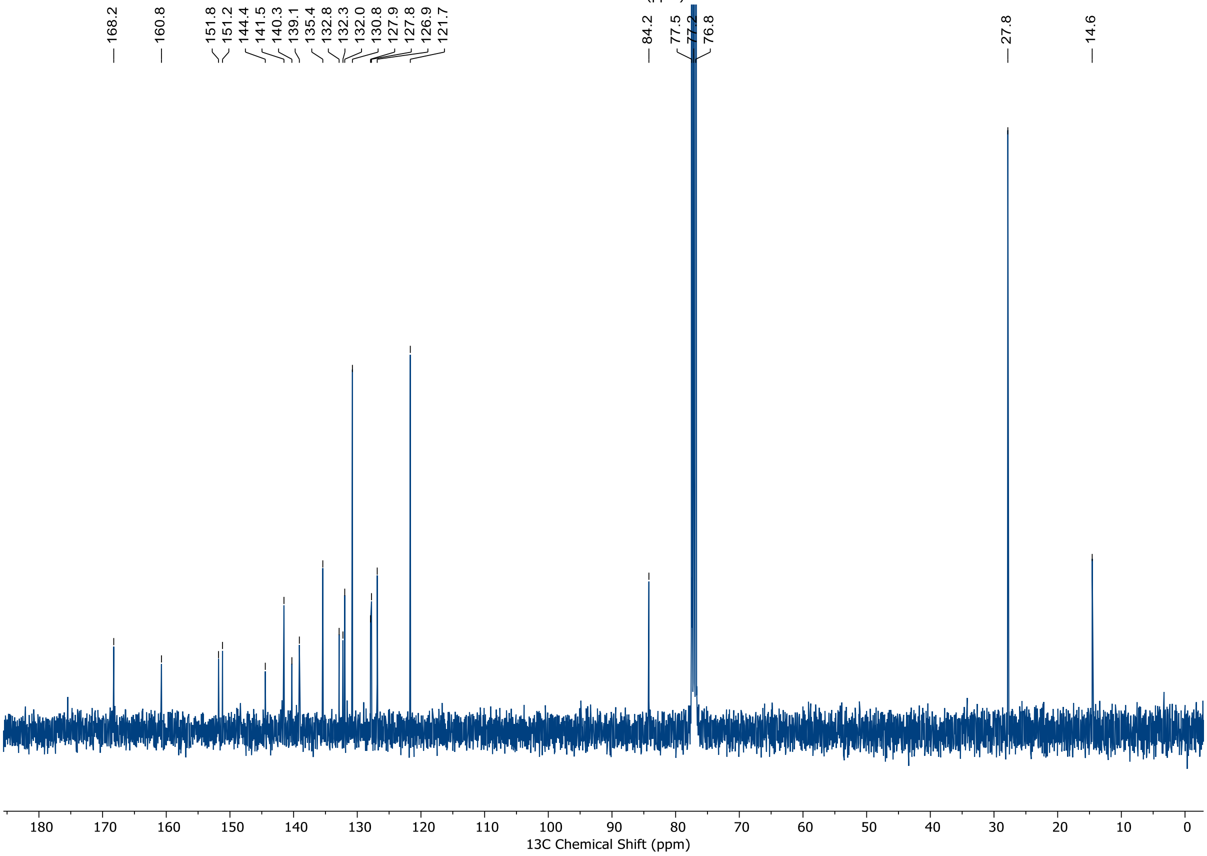

(S,E)-2-hydroxy-4-(2-hydroxy-4-(4-(2-(5-(3-(4-hydroxyphenyl)-2-methylacrylamido)picolinamido)-3-(1H-1,2,3-triazol-4-yl)propanamido)benzamido)-3-methoxybenzamido)-3-methoxybenzoic acid (6)

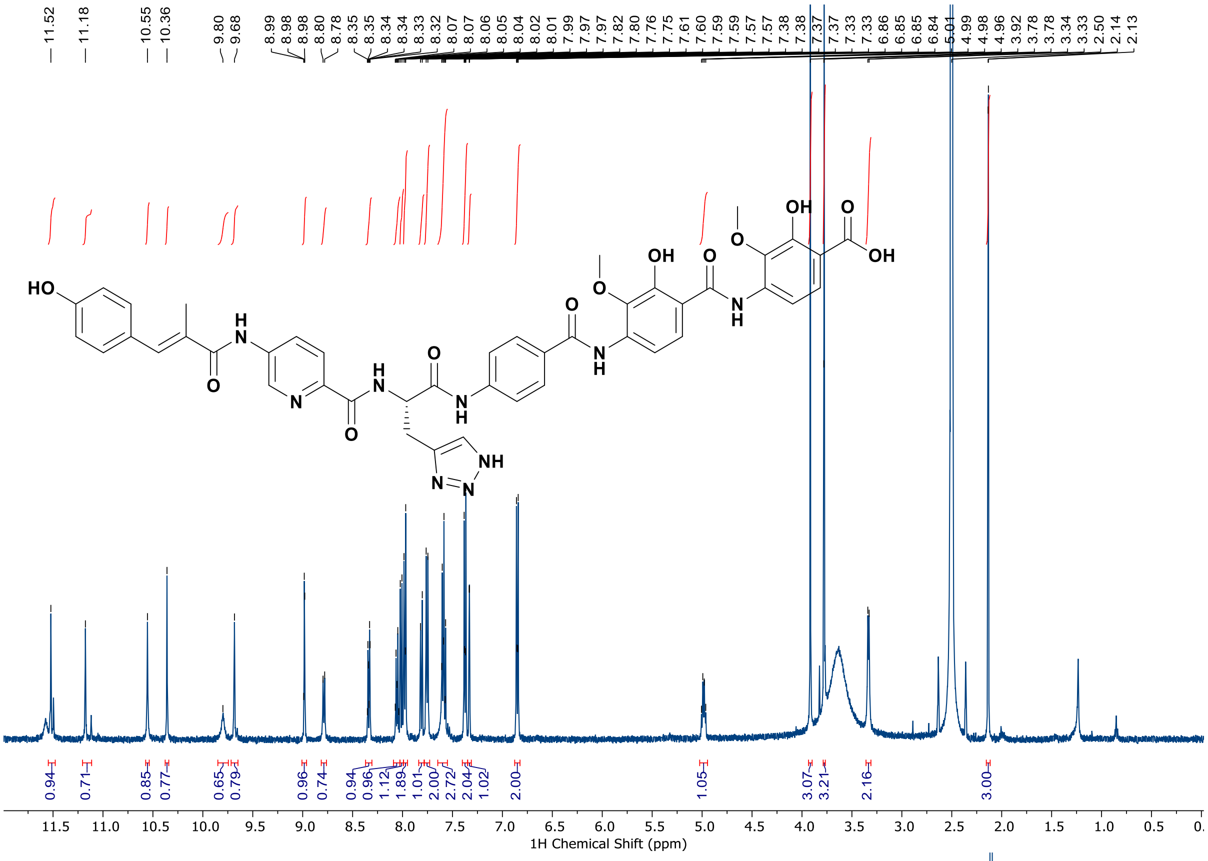

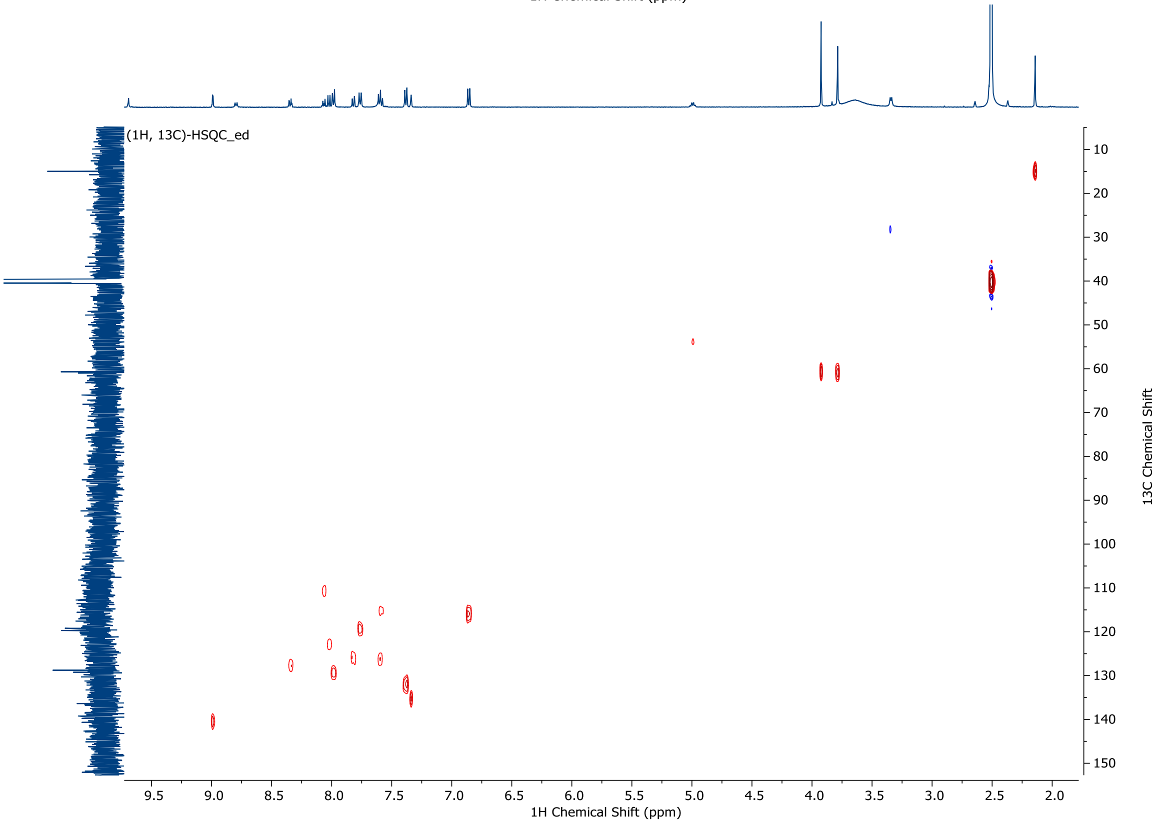

3.3 NMR spectra of F-Variations (12, 13, 15) and reaction intermediates

2-(benzyloxy)-3-methoxy-4-nitrobenzoic acid (23)

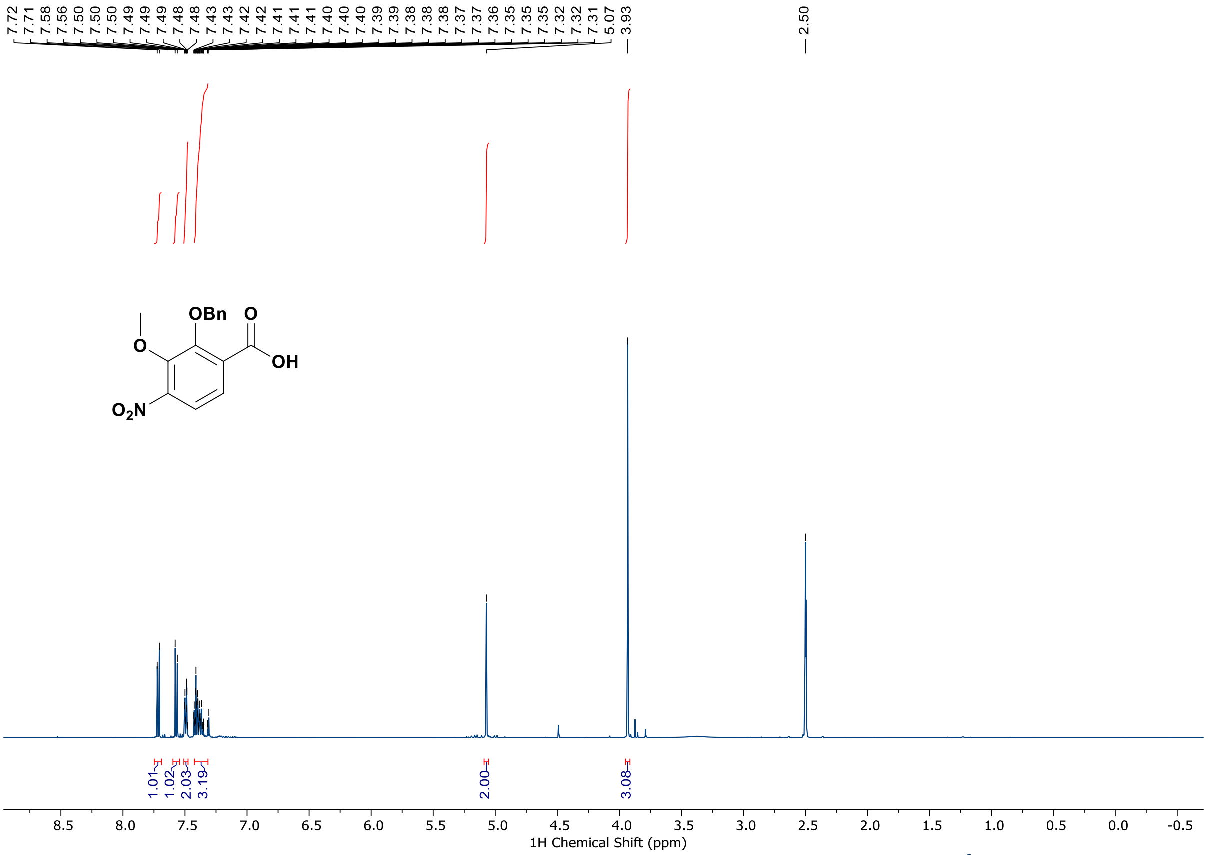

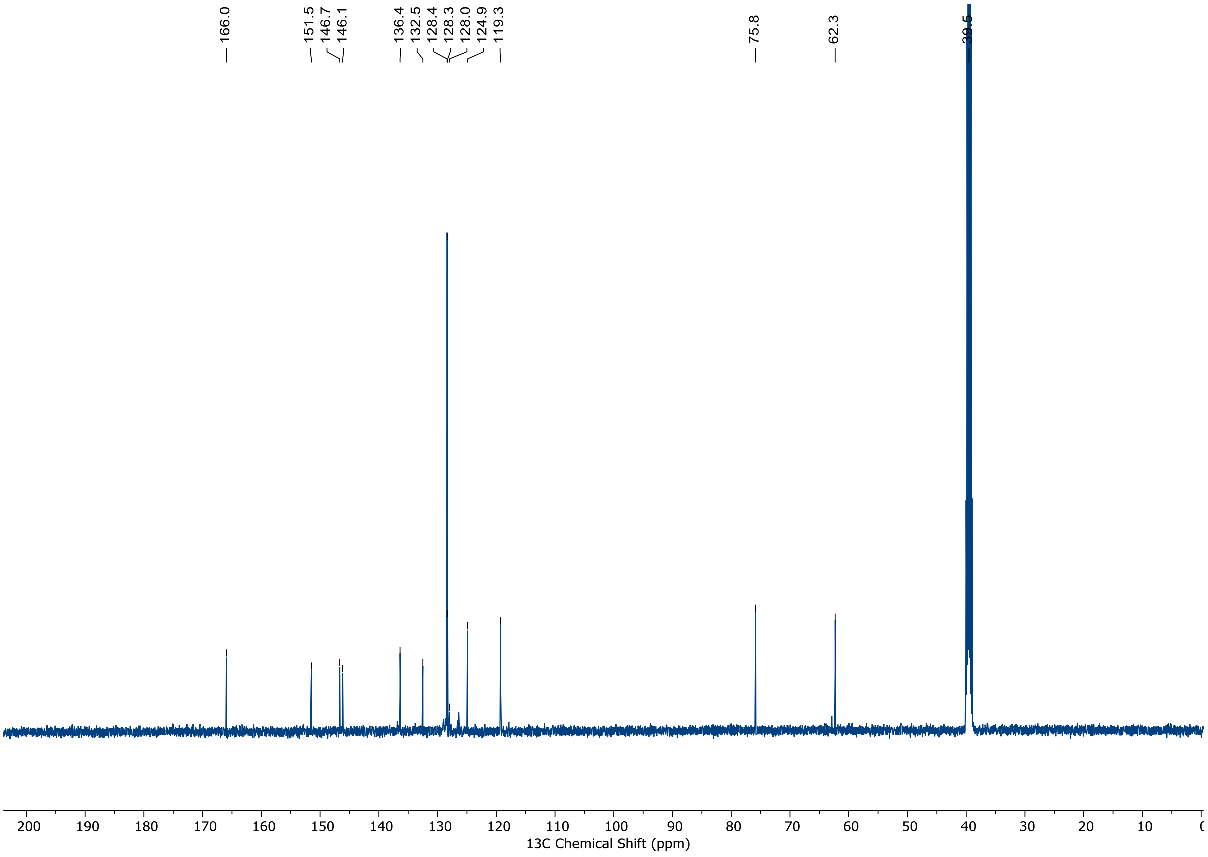

methyl 5-(2-(benzyloxy)-3-methoxy-4-nitrobenzamido)picolinate (24a-1)

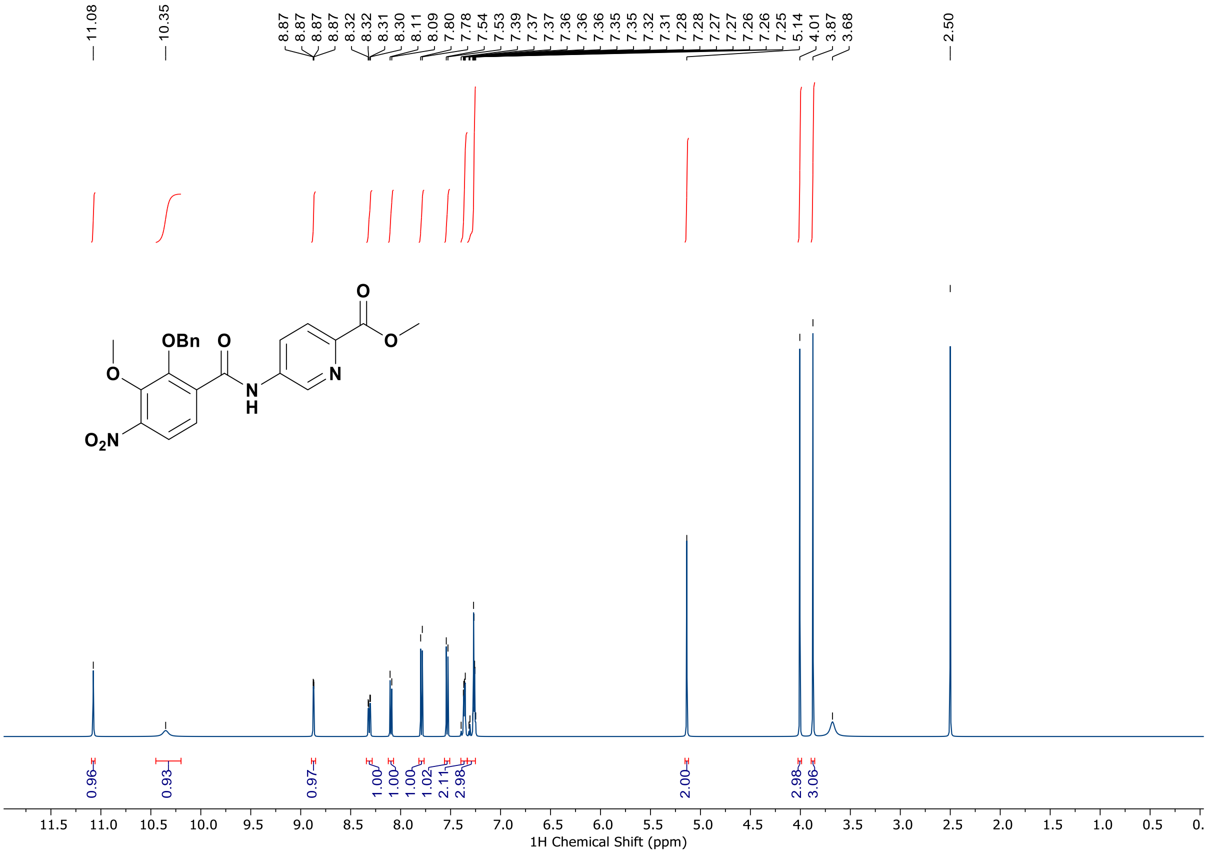

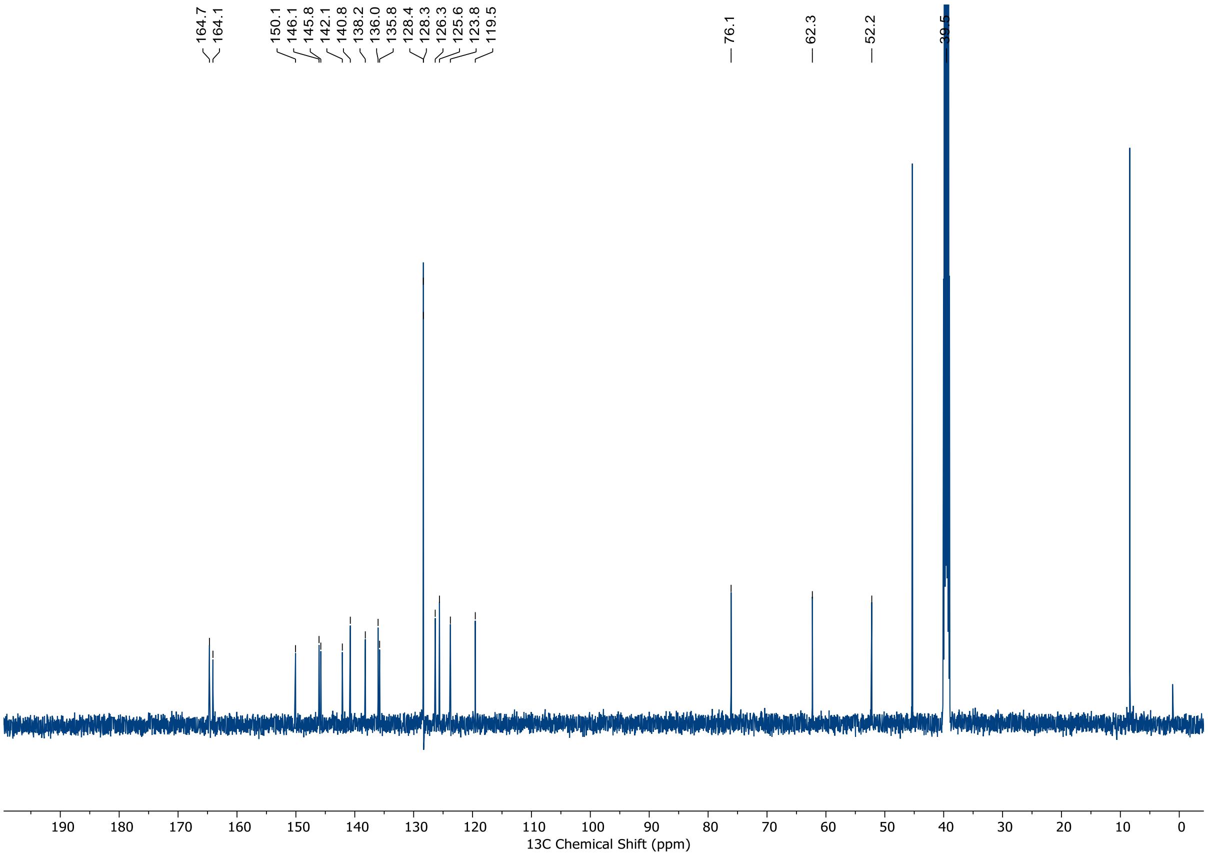

methyl 5-(4-amino-2-(benzyloxy)-3-methoxybenzamido)picolinate (25a)

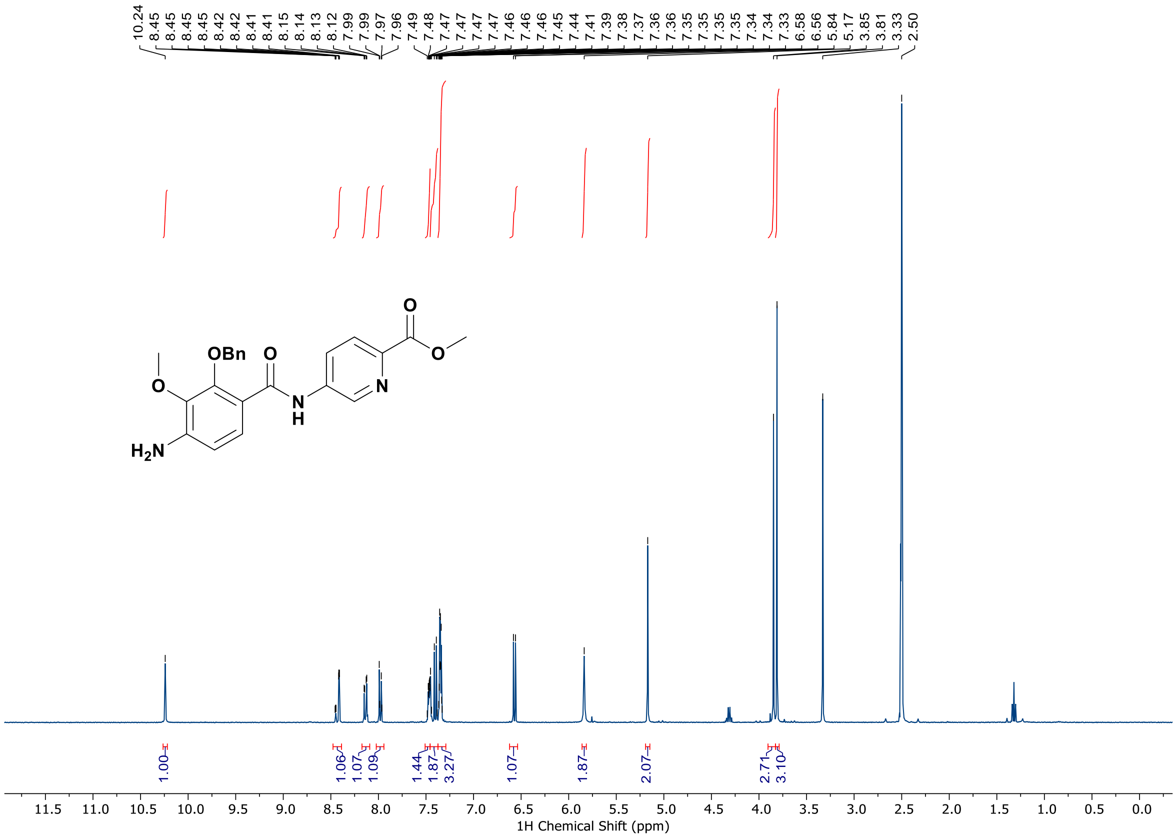

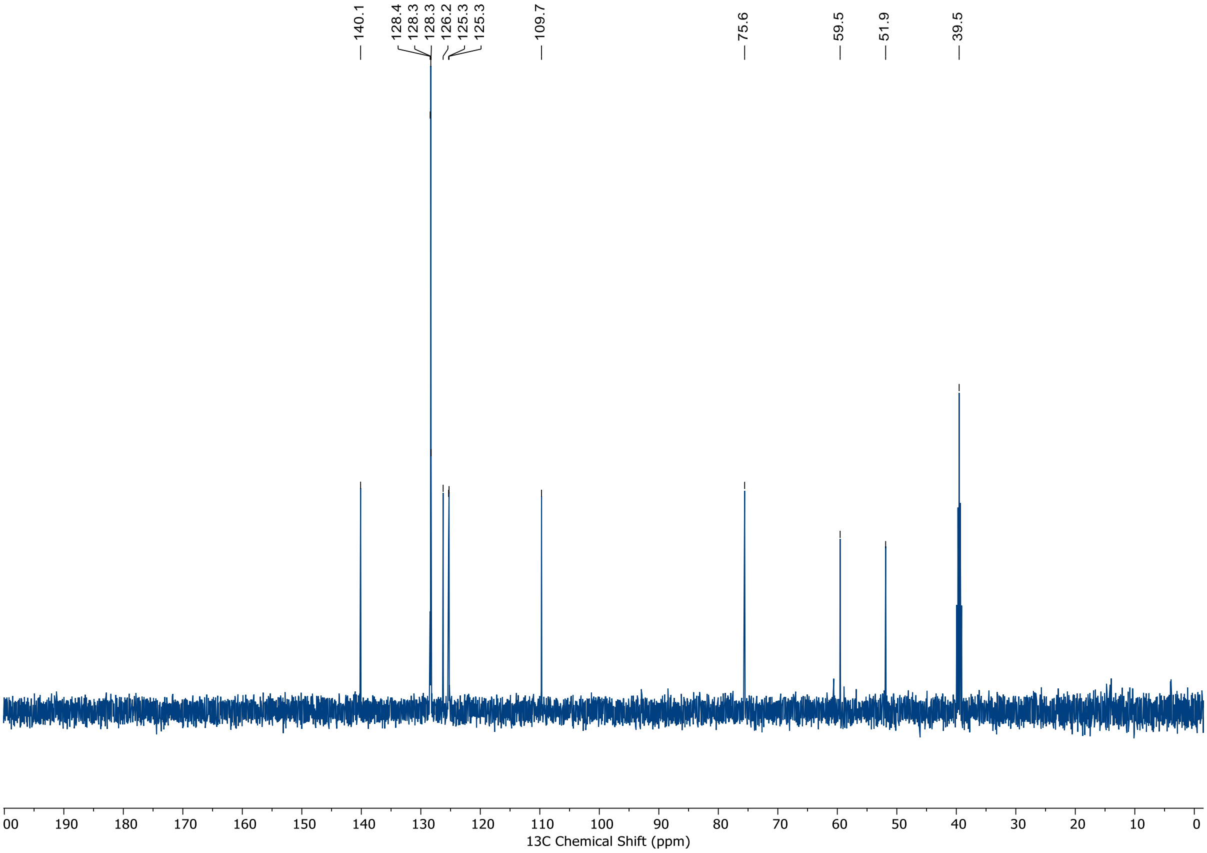

4-amino-2-(benzyloxy)-N-(6-(hydroxymethyl)pyridin-3-yl)-3-methoxybenzamide (26)

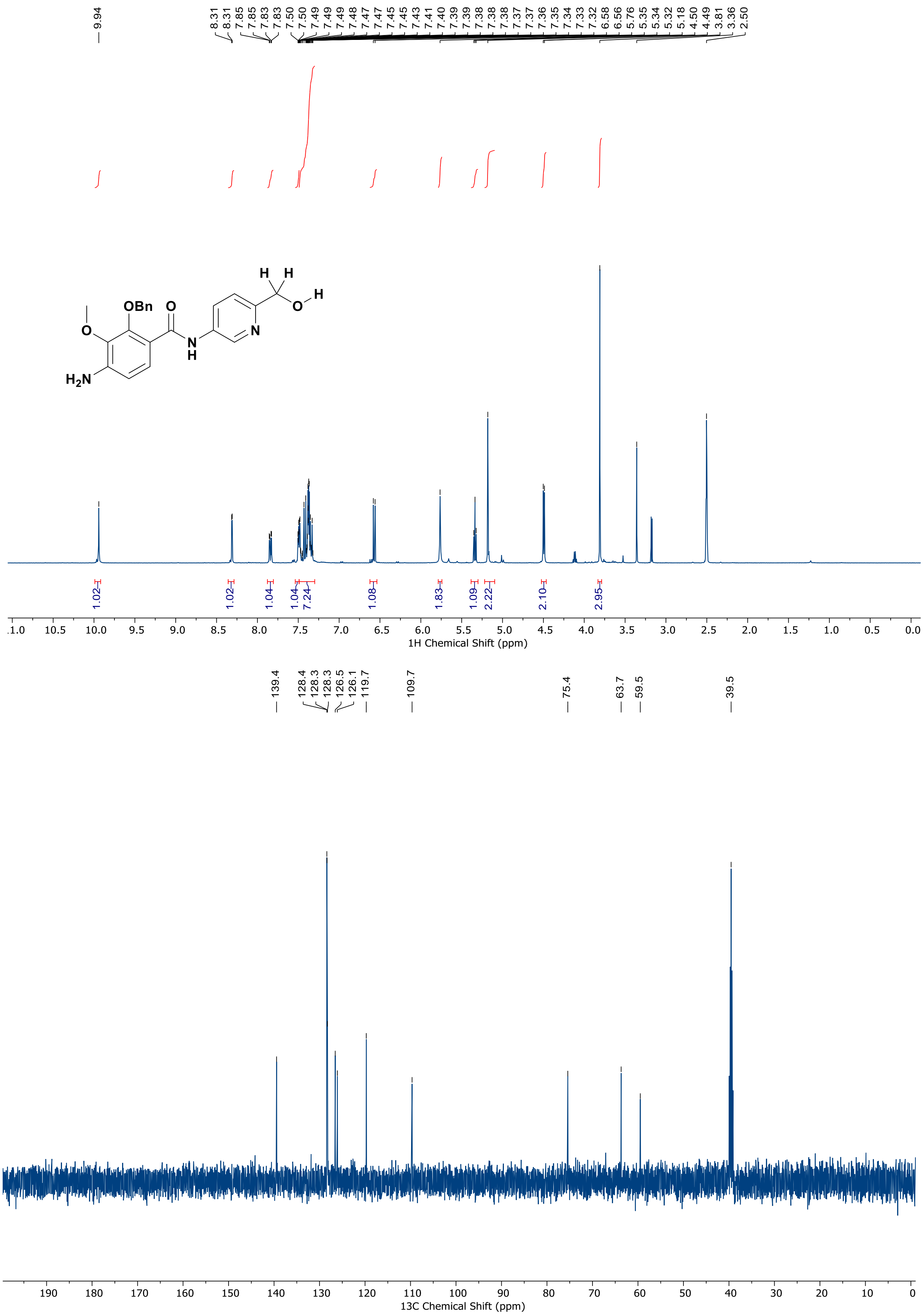

methyl 5-(2-(benzyloxy)-3-methoxy-4-(4-nitrobenzamido)benzamido)picolinate (25a-1)

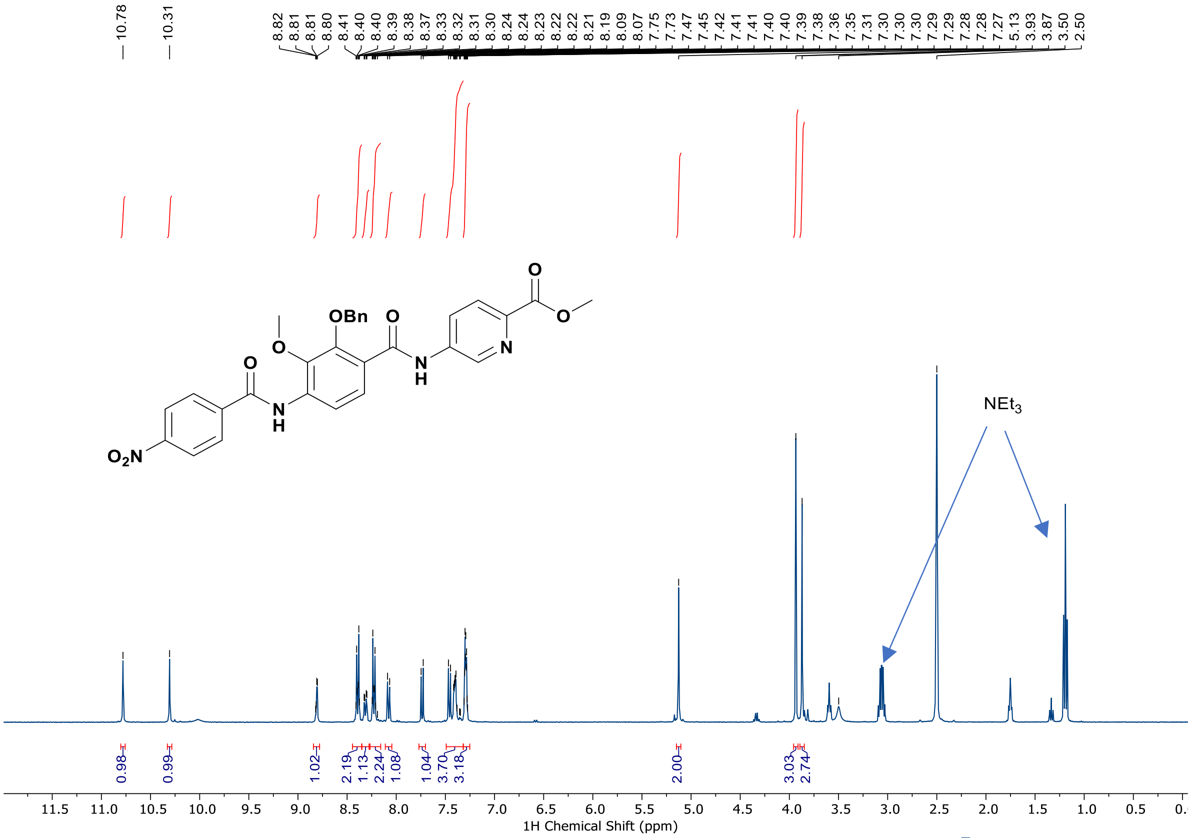

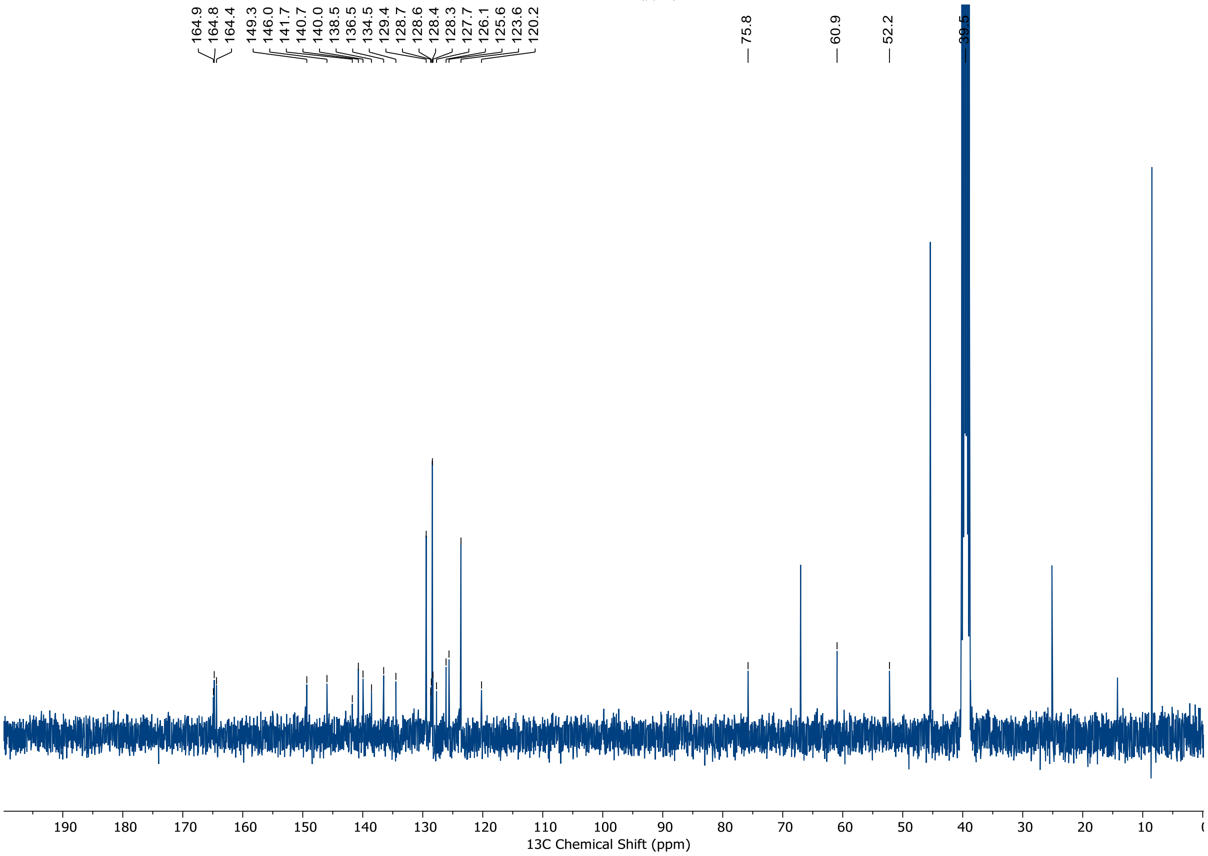

methyl 5-(4-(4-aminobenzamido)-2-(benzyloxy)-3-methoxybenzamido)picolinate (36a)

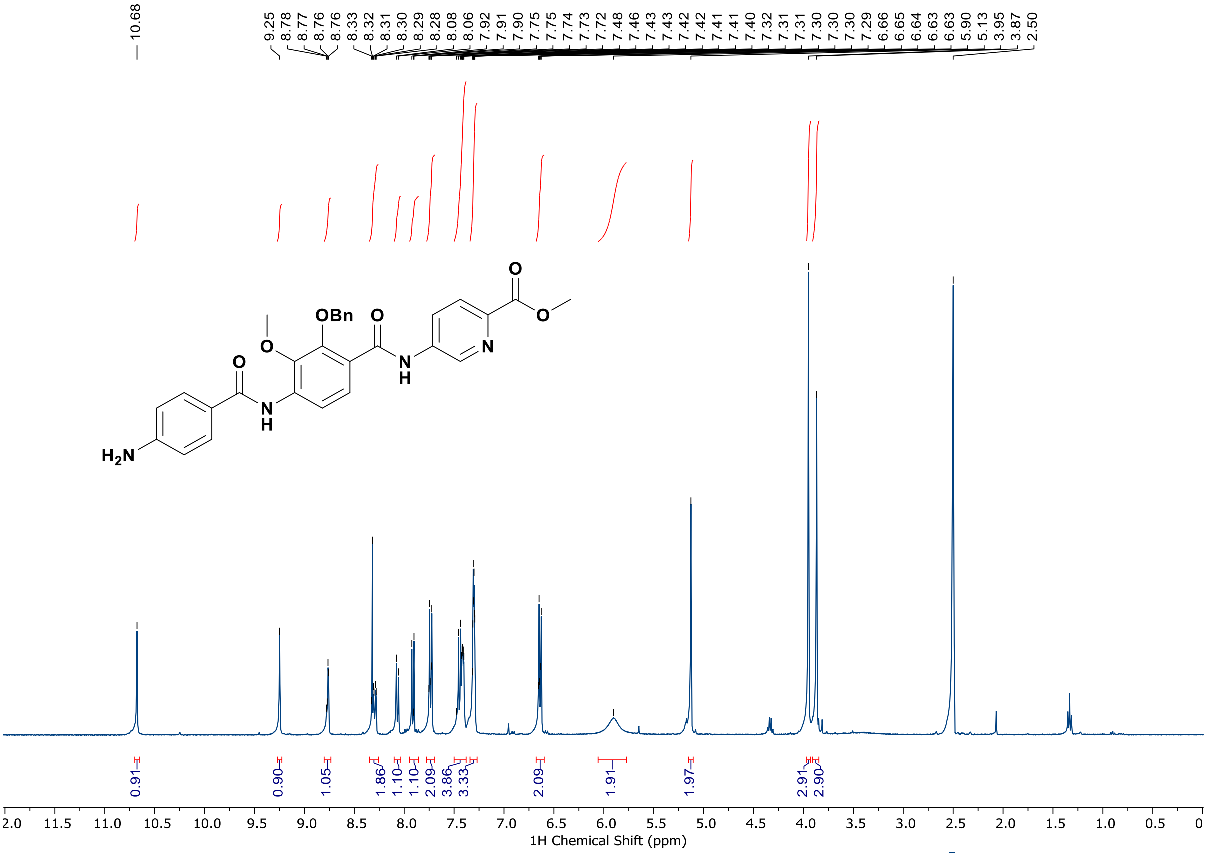

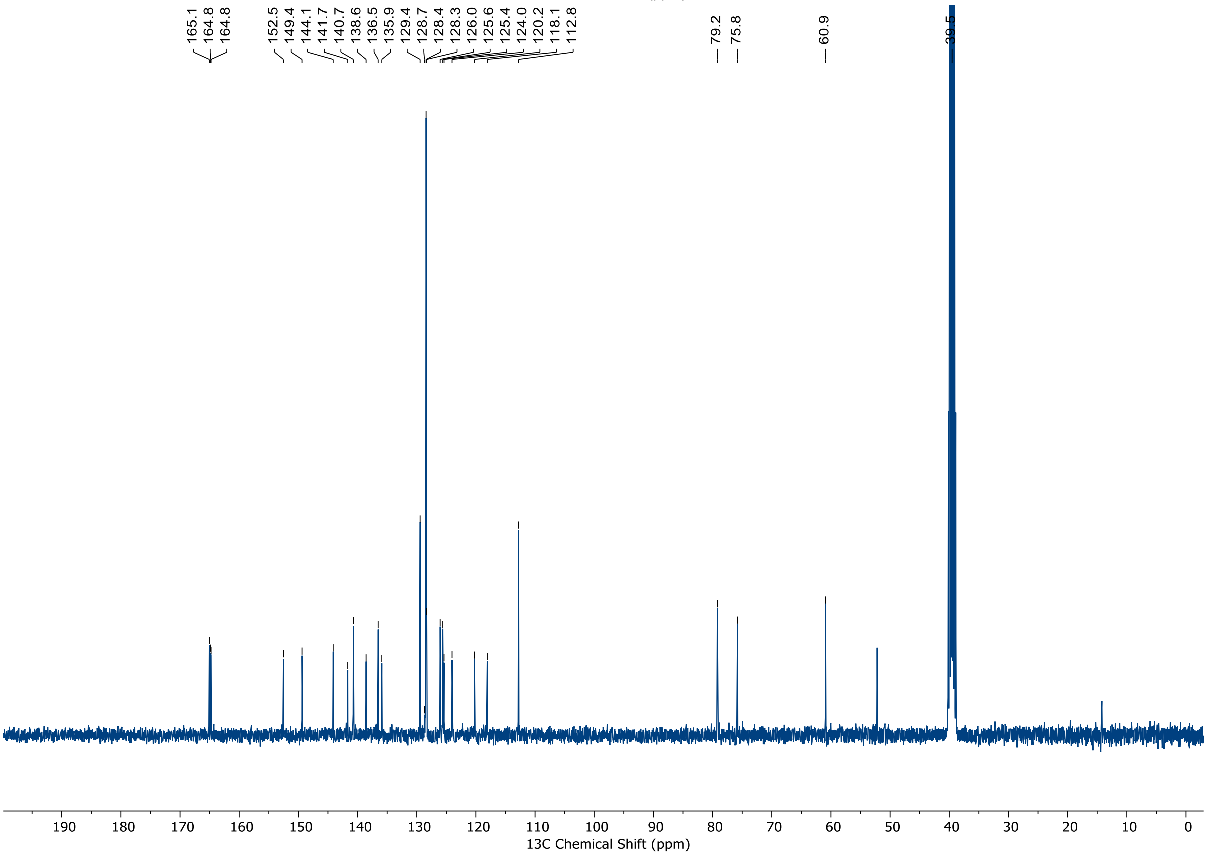

methyl (S)-5-(2-(benzyloxy)-4-(4-(2-((tert-butoxycarbonyl)amino)-3-(1-((pivaloyloxy)methyl)-1H-1,2,3-triazol-4-yl)propanamido)benzamido)-3-methoxybenzamido)picolinate (36a-1)

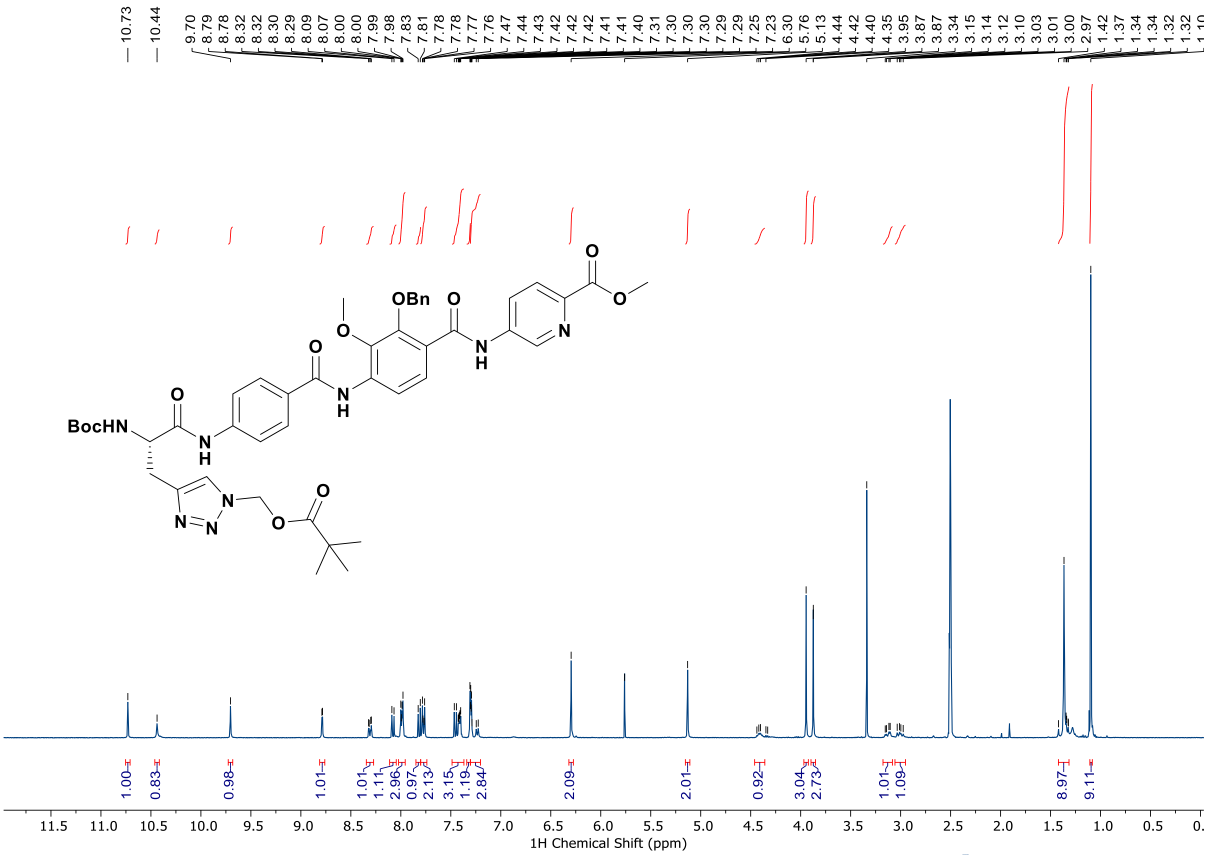

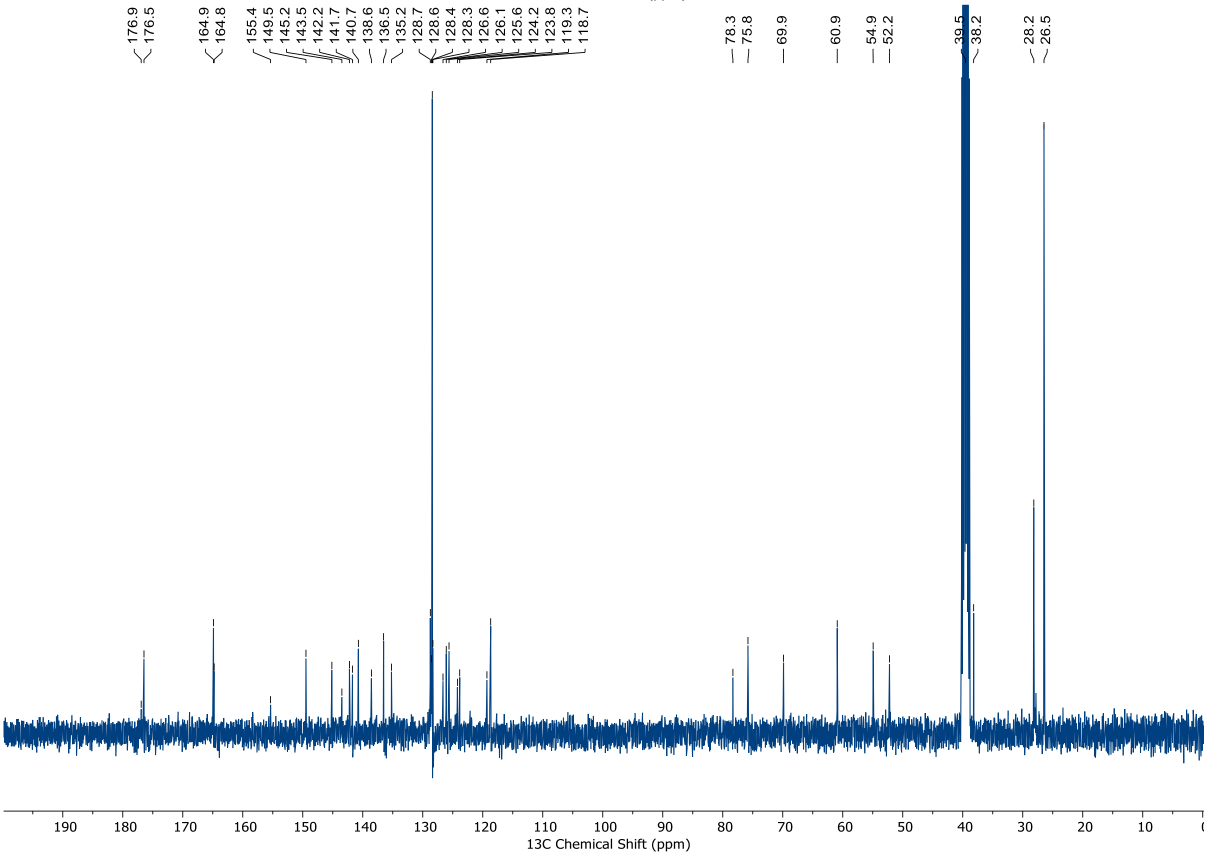

methyl (S)-5-(4-(4-(2-((tert-butoxycarbonyl)amino)-3-(1-((pivaloyloxy)methyl)-1H-1,2,3-triazol-4-yl)propanamido)benzamido)-2-hydroxy-3-methoxybenzamido)picolinate (36a-2)

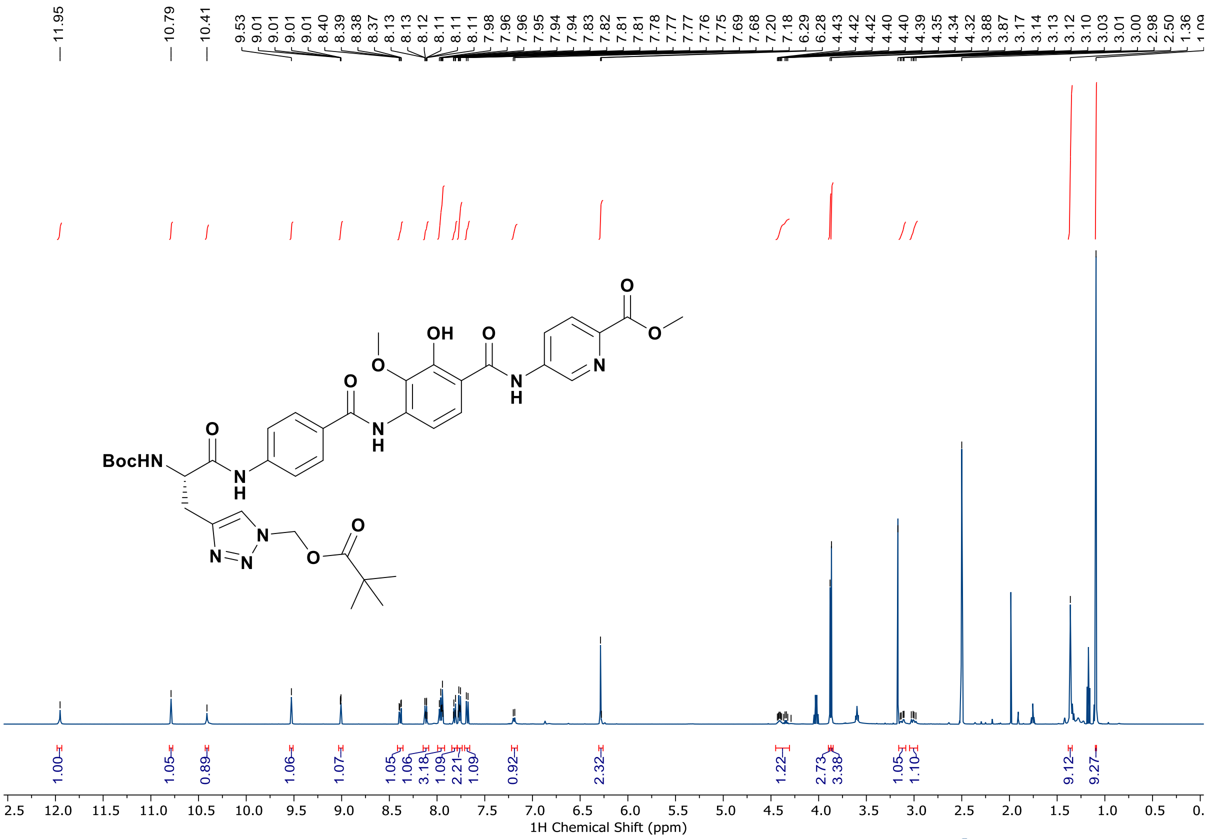

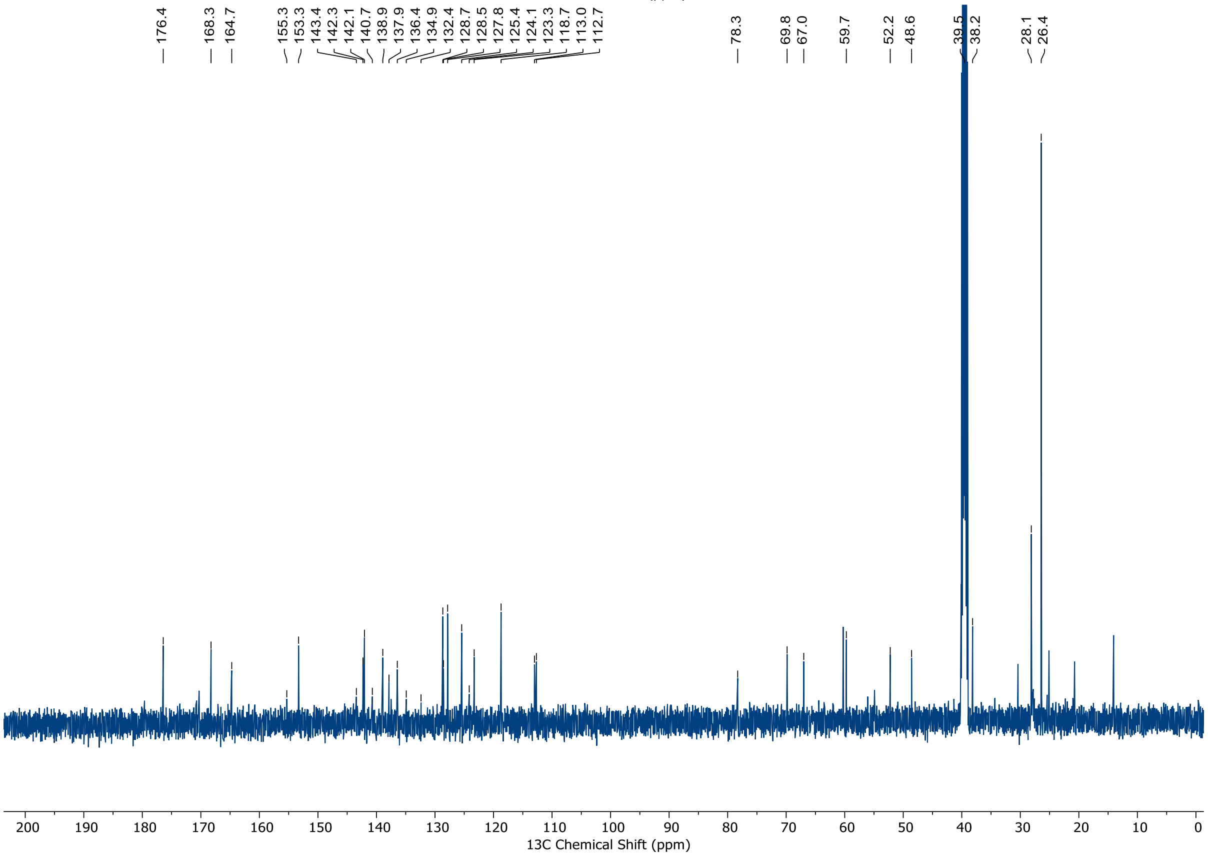

methyl (S)-5-(4-(4-(2-amino-3-(1-((pivaloyloxy)methyl)-1H-1,2,3-triazol-4-yl)propanamido)benzamido)-2-hydroxy-3-methoxybenzamido)picolinate (37a)

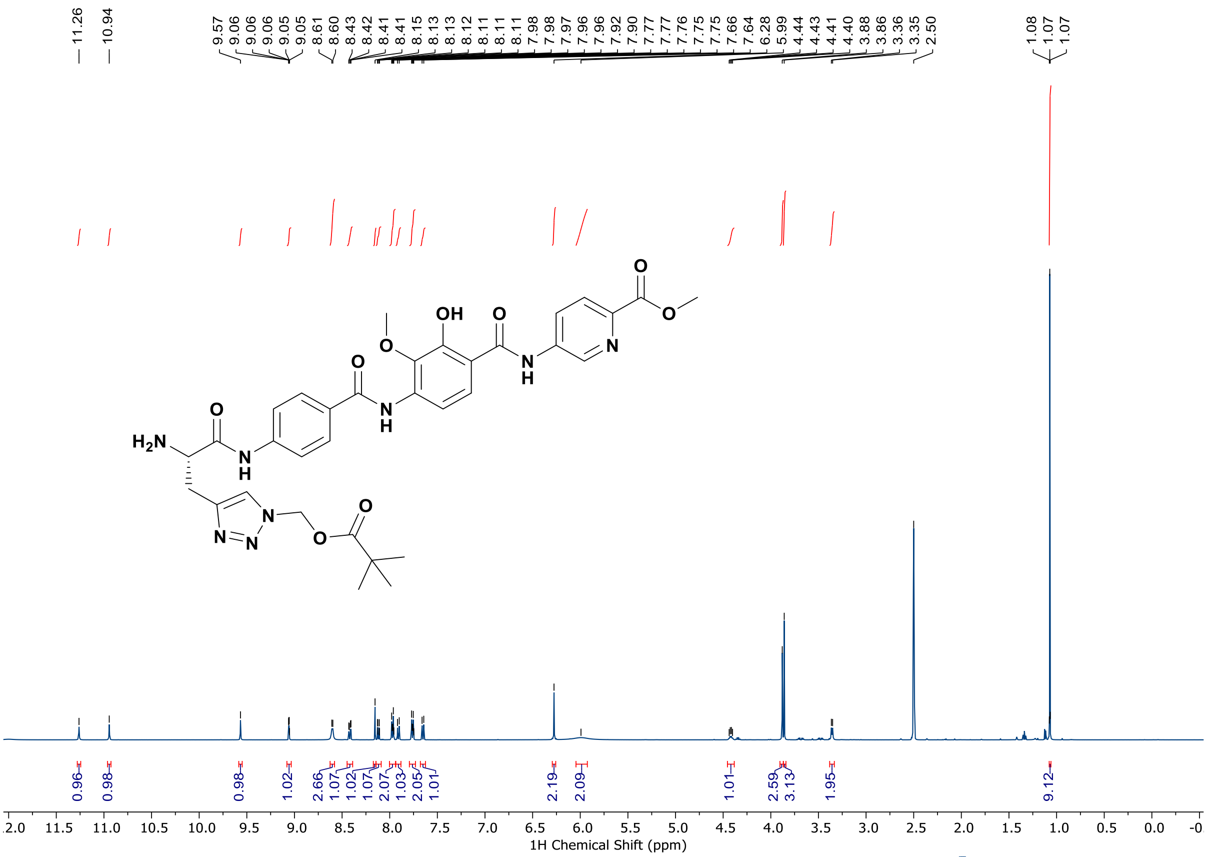

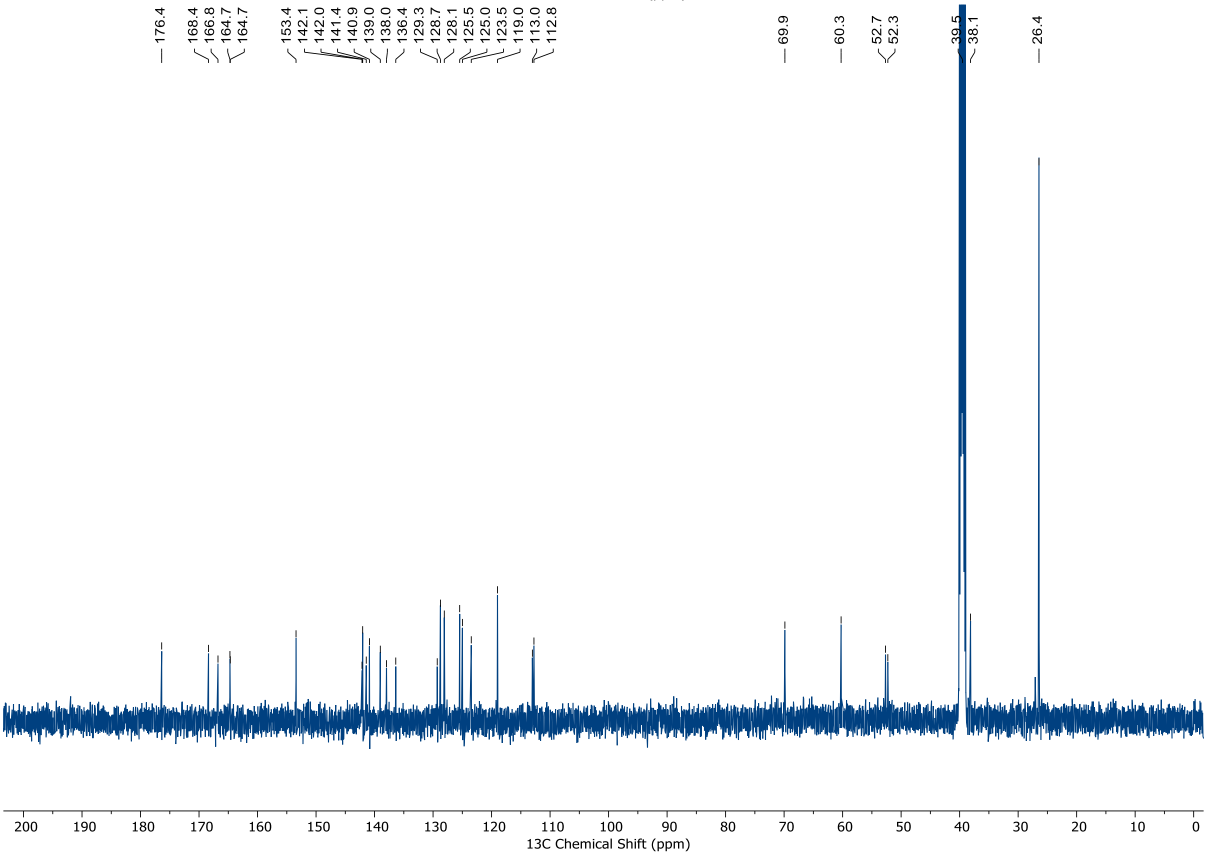

perchlorophenyl (E)-4-(3-(4-hydroxyphenyl)-2-methylacrylamido)benzoate (39)

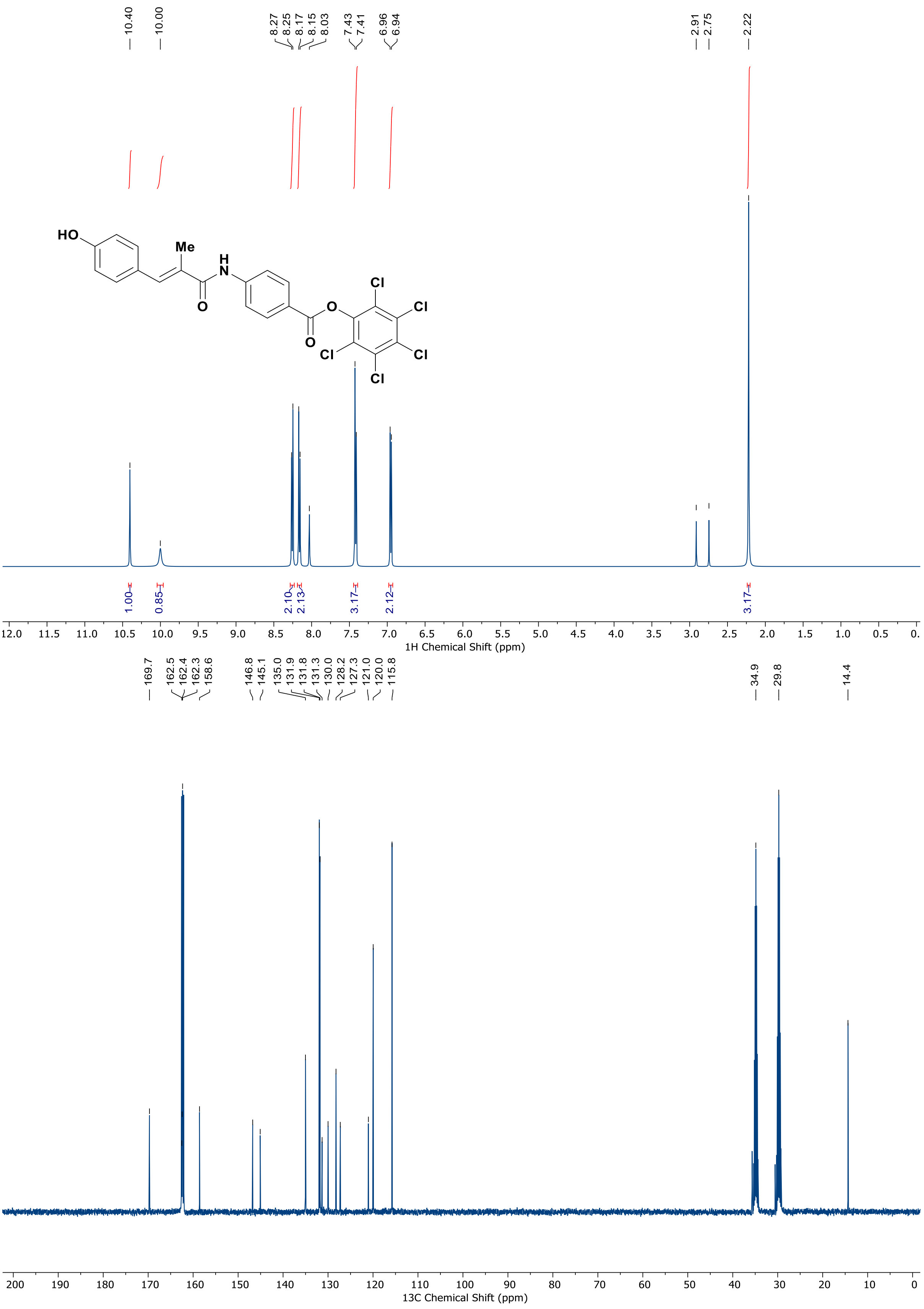

(S,E)-5-(2-hydroxy-4-(4-(2-(4-(3-(4-hydroxyphenyl)-2-methylacrylamido)benzamido)-3-(1H-1,2,3-triazol-4-yl)propanamido)benzamido)-3-methoxybenzamido)picolinic acid (12)

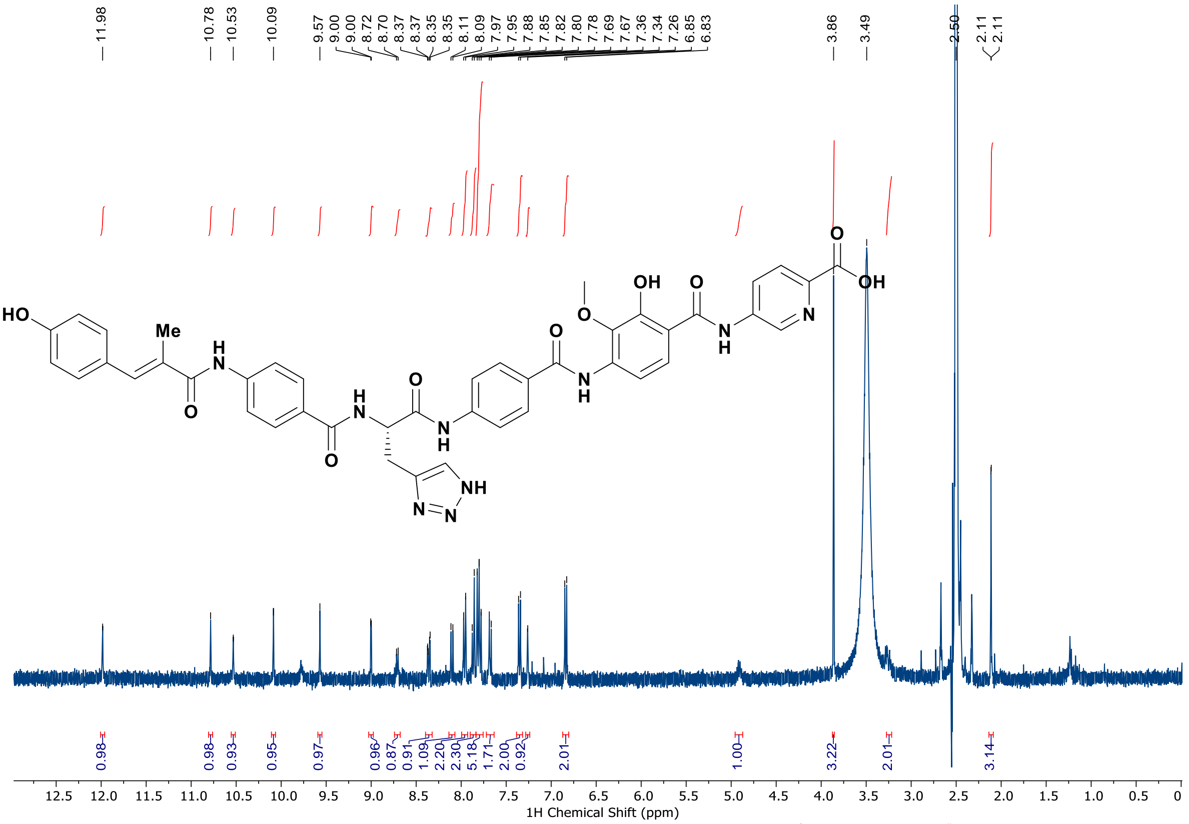

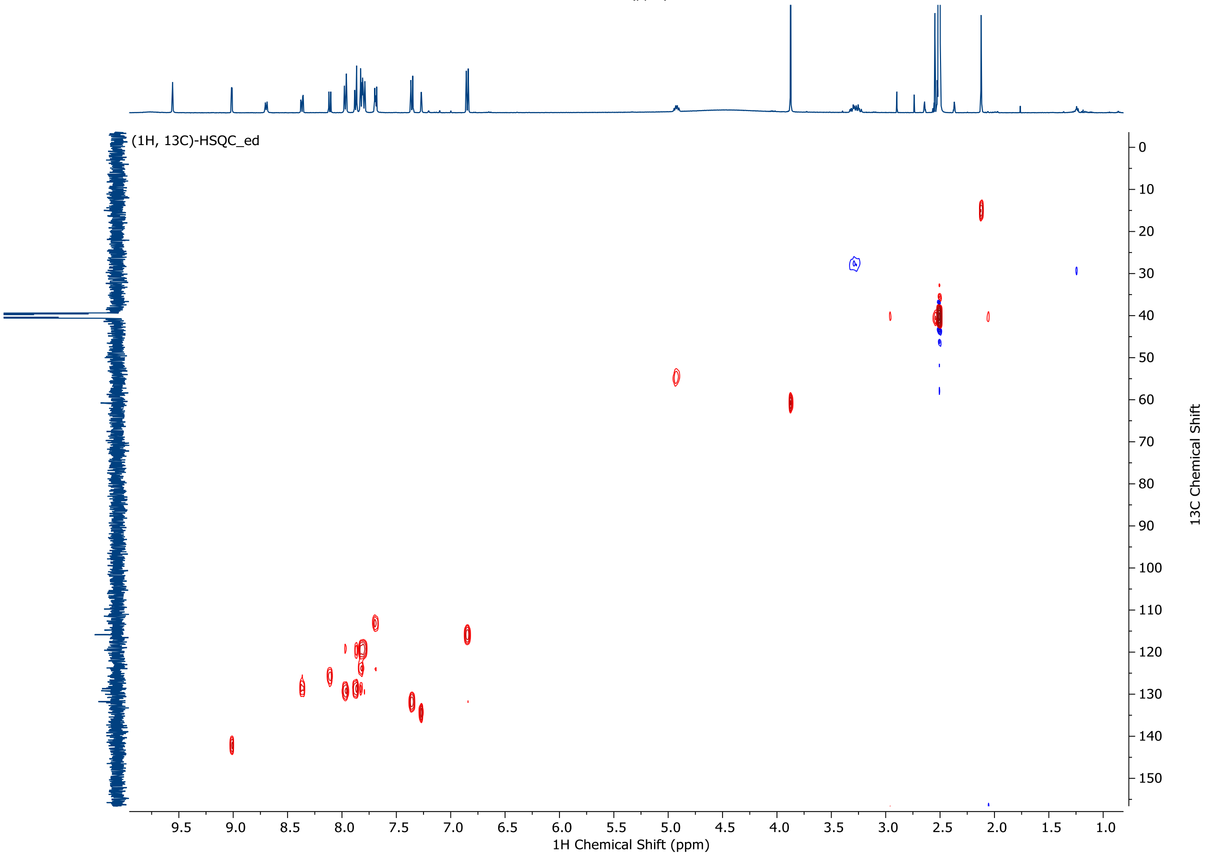

methyl 6-(2-(benzyloxy)-3-methoxy-4-nitrobenzamido)nicotinate (24b-1)

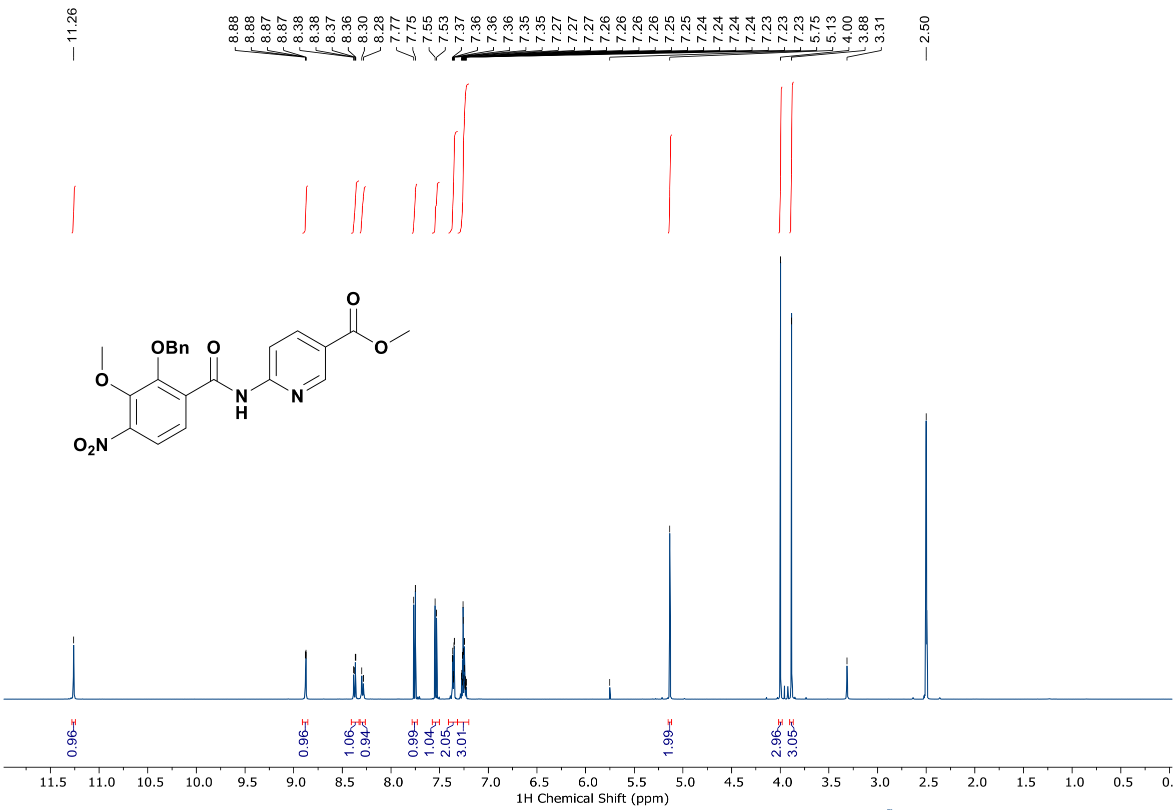

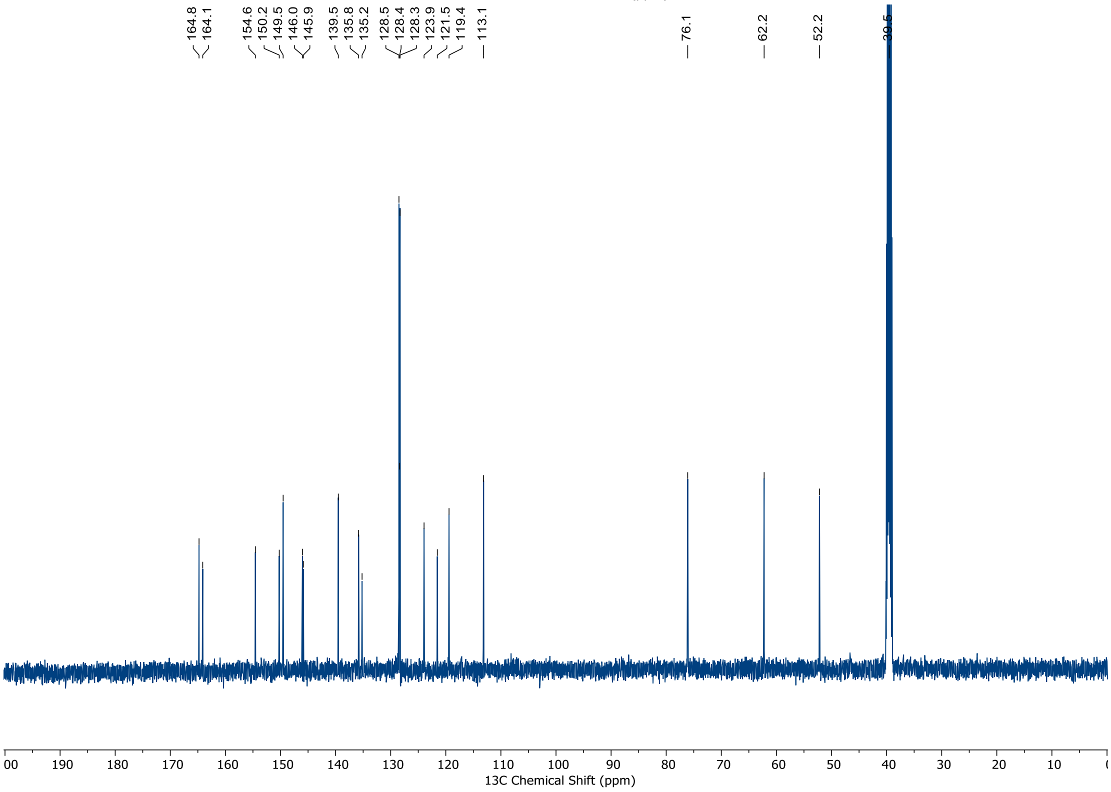

methyl 6-(4-amino-2-(benzyloxy)-3-methoxybenzamido)nicotinate (25b)

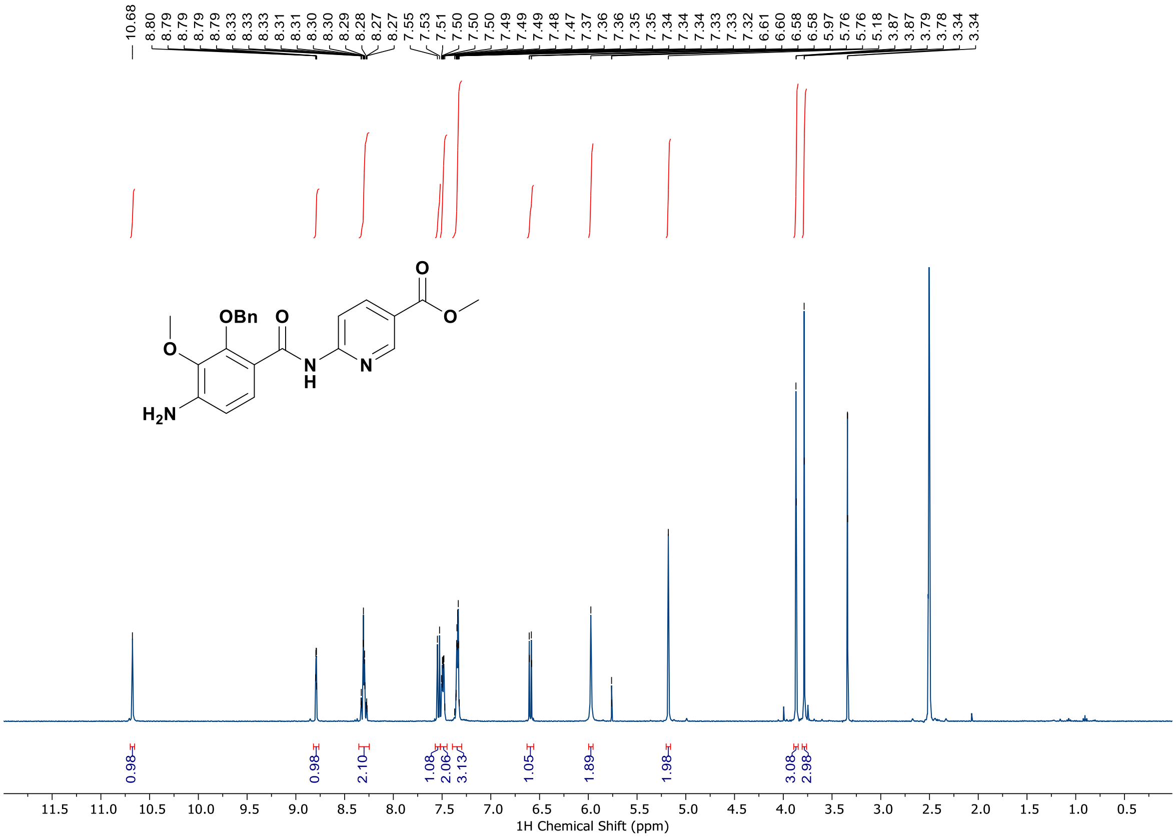

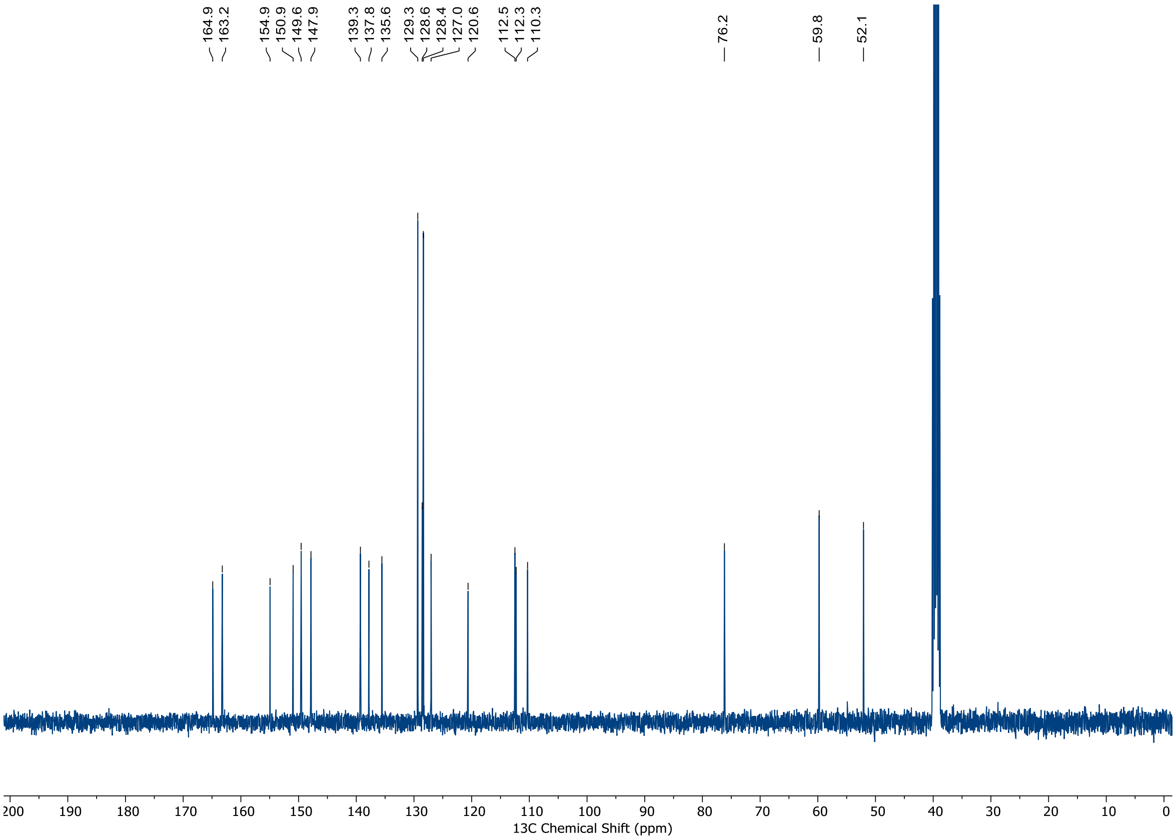

methyl 6-(2-(benzyloxy)-3-methoxy-4-(4-nitrobenzamido)benzamido)nicotinate (25b-1)

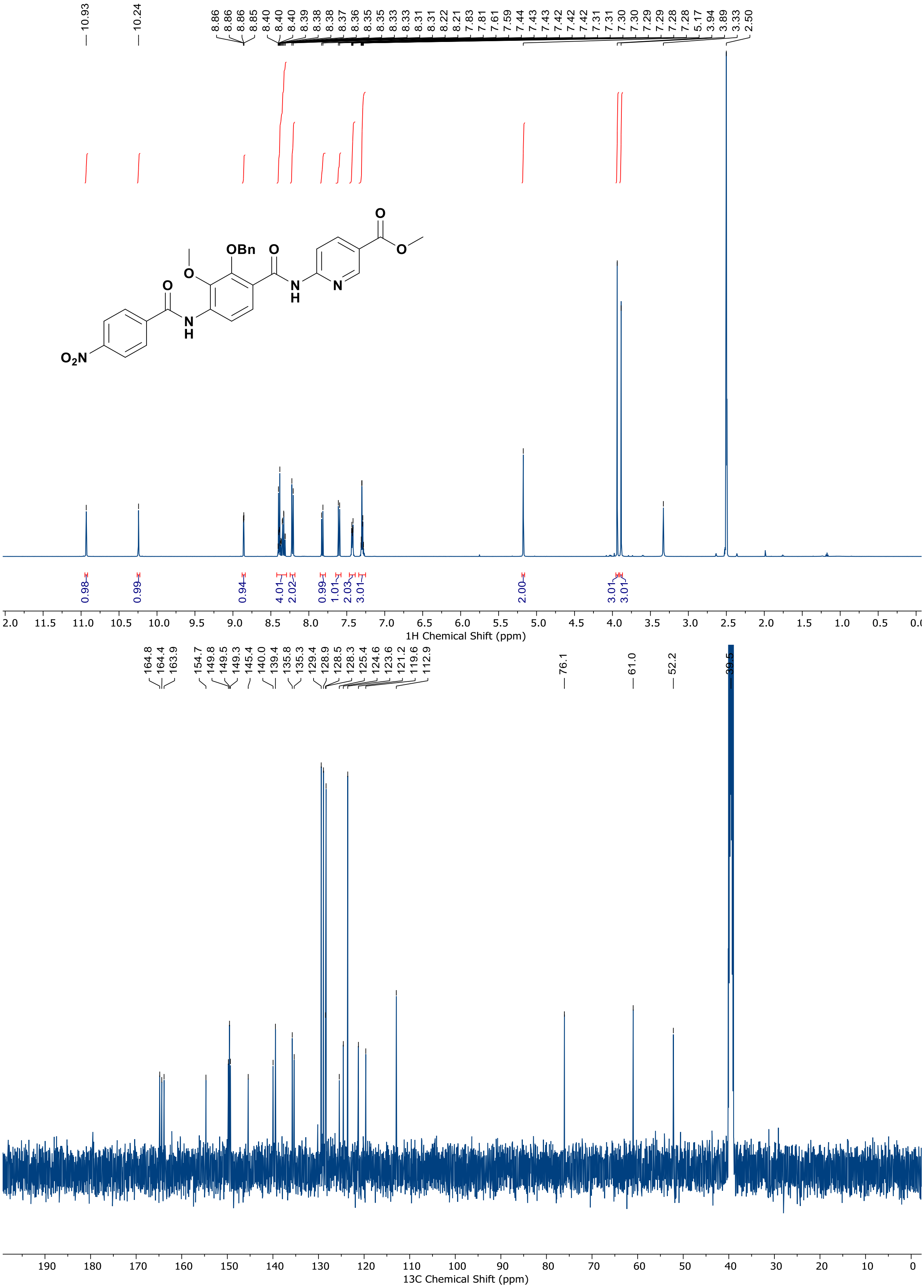

methyl 6-(4-(4-aminobenzamido)-2-(benzyloxy)-3-methoxybenzamido)nicotinate (36b)

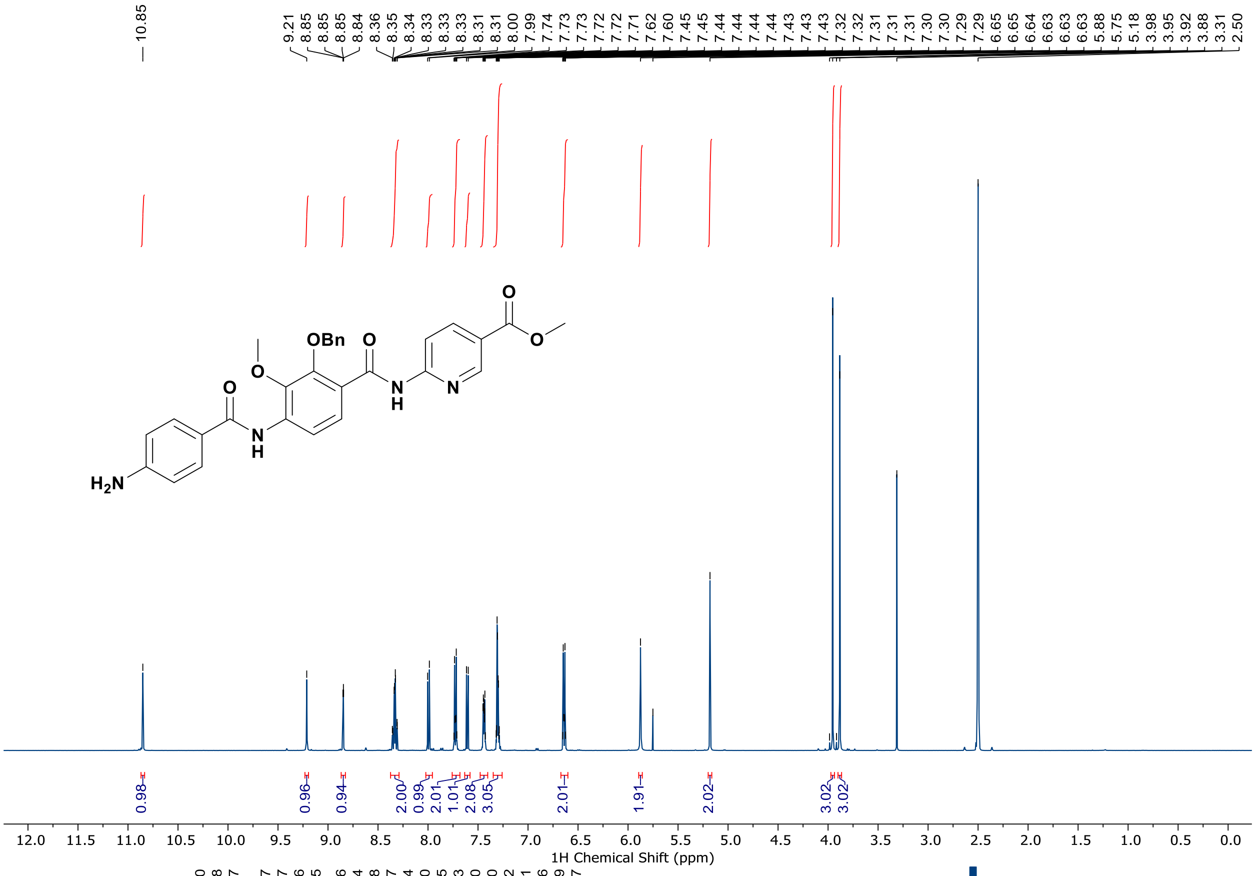

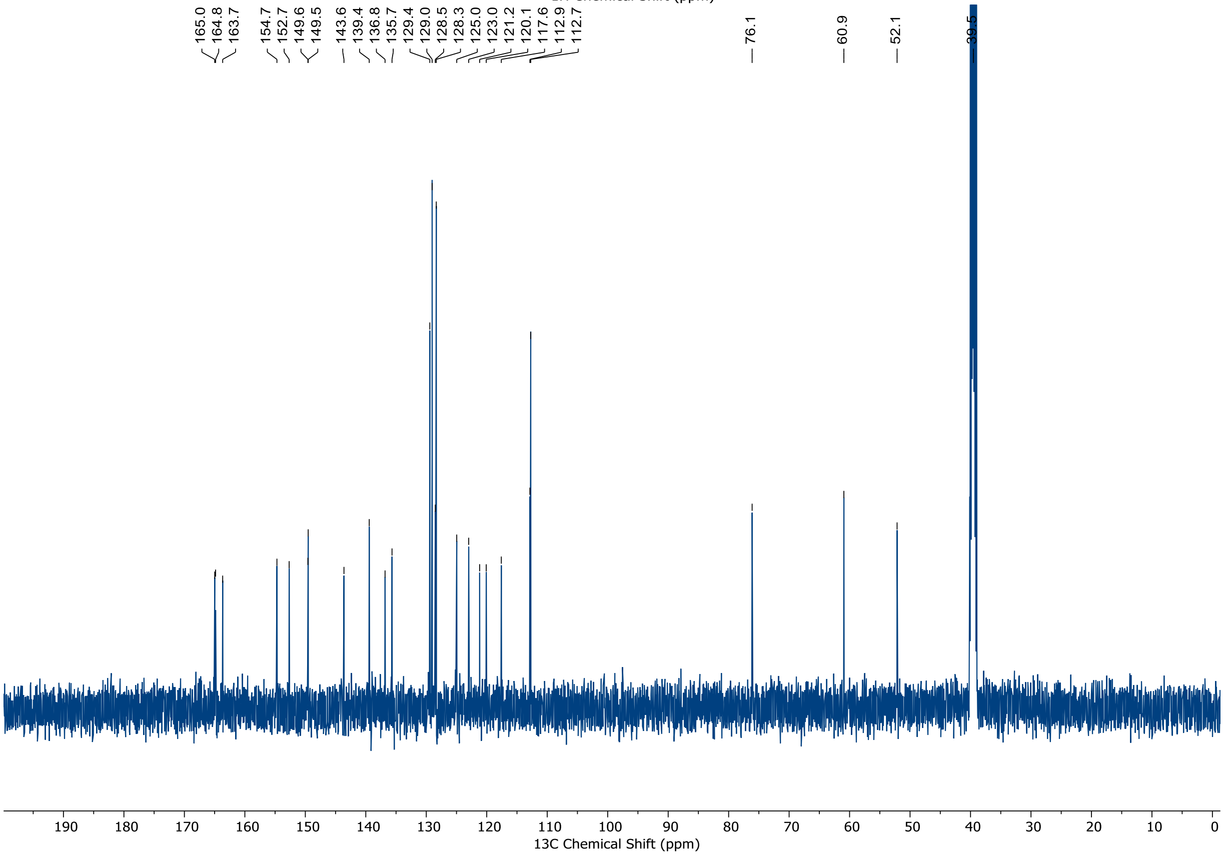

(S)-2-((tert-butoxycarbonyl)amino)-3-(1-((pivaloyloxy)methyl)-1H-1,2,3-triazol-4-yl)propanoic acid (38)

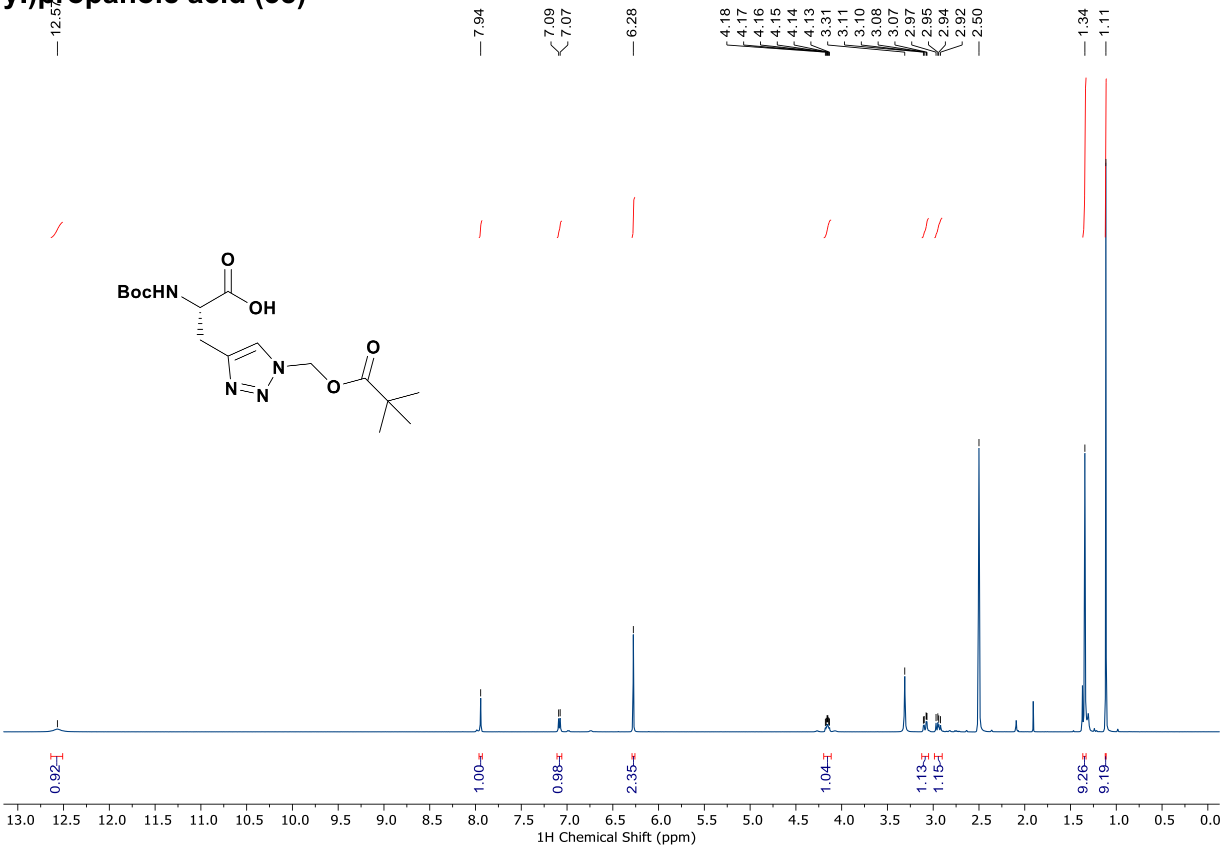

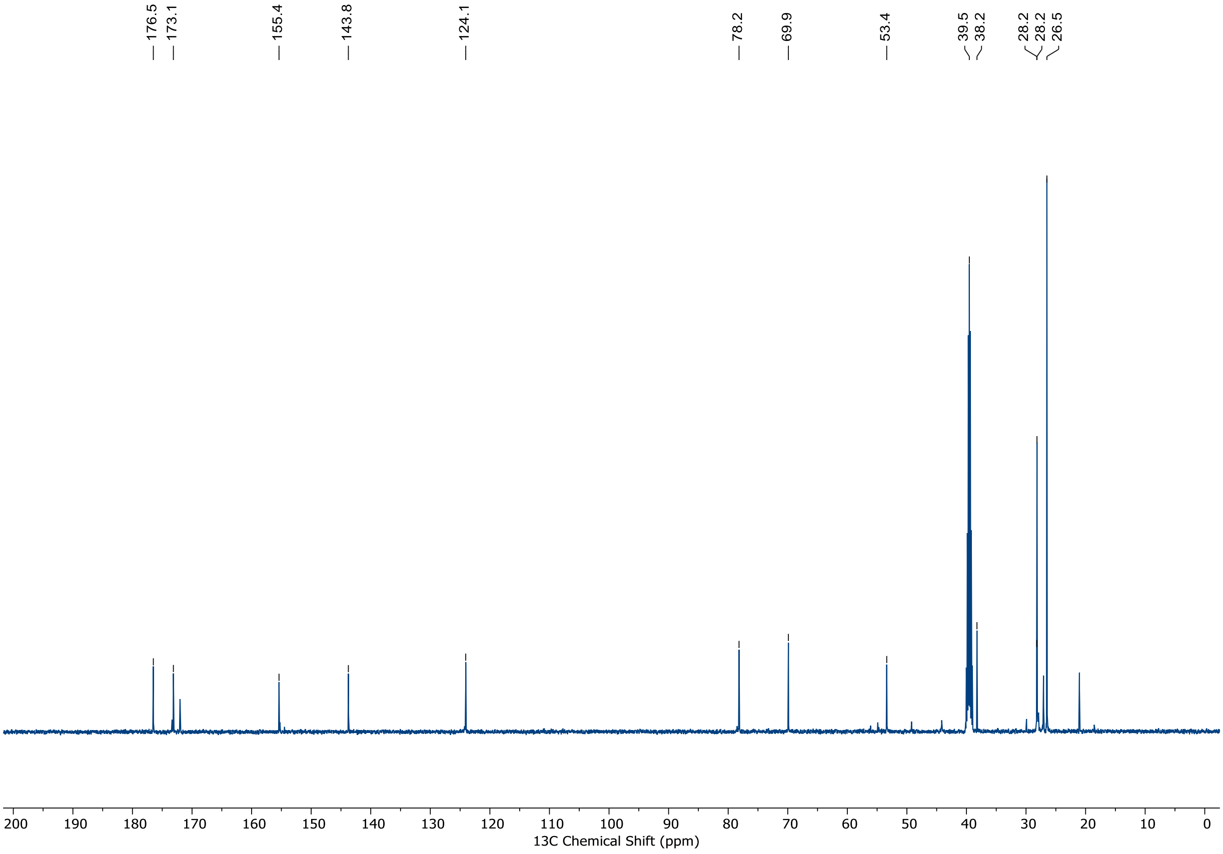

methyl (S)-6-(2-(benzyloxy)-4-(4-(2-((tert-butoxycarbonyl)amino)-3-(1-((pivaloyloxy)methyl)-1H-1,2,3-triazol-4-yl)propanamido)benzamido)-3-methoxybenzamido)nicotinate (36b-1)

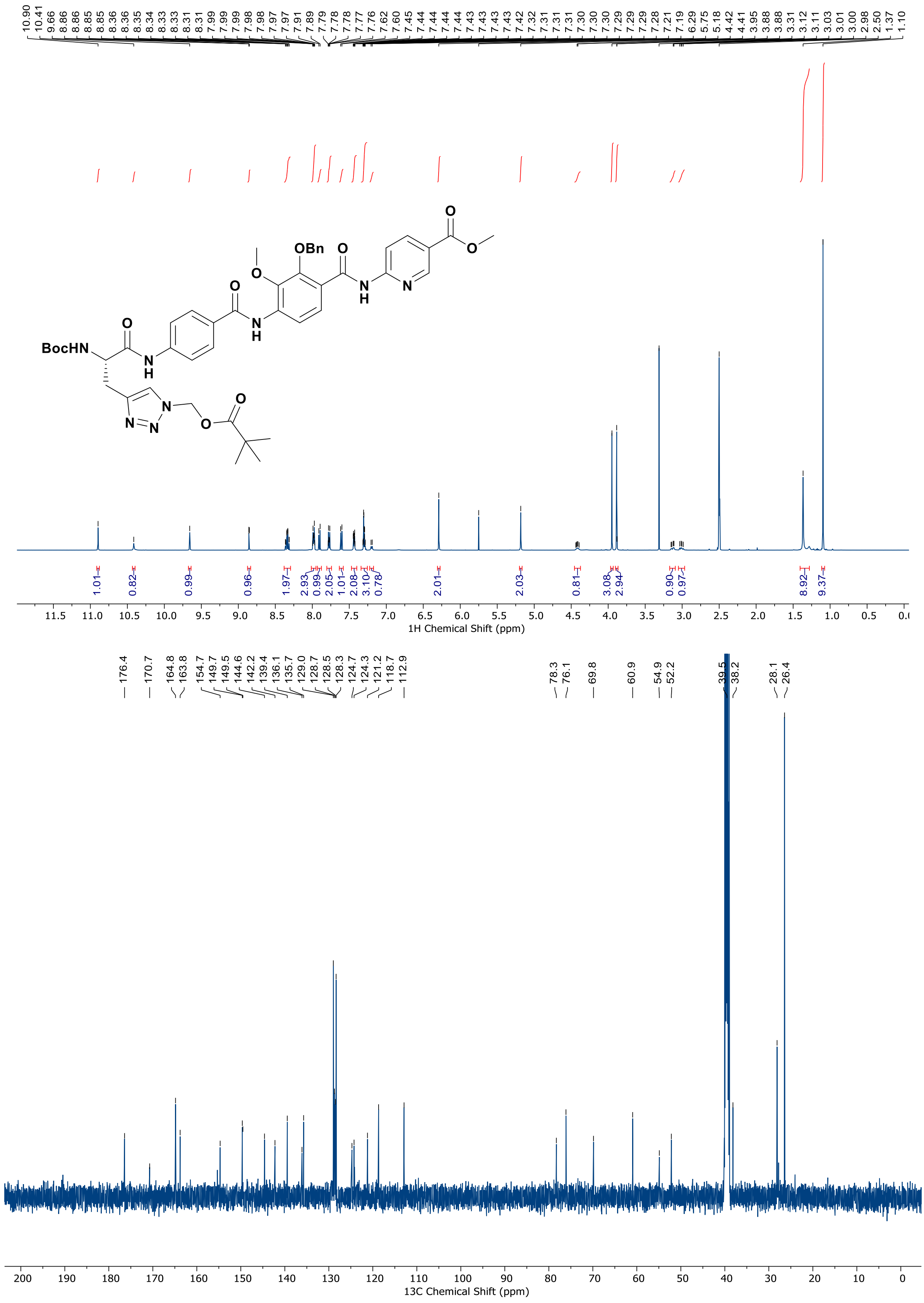

methyl (S)-6-(4-(4-(2-((tert-butoxycarbonyl)amino)-3-(1-((pivaloyloxy)methyl)-1H-1,2,3-triazol-4-yl)propanamido)benzamido)-2-hydroxy-3-methoxybenzamido)nicotinate (36b-2)

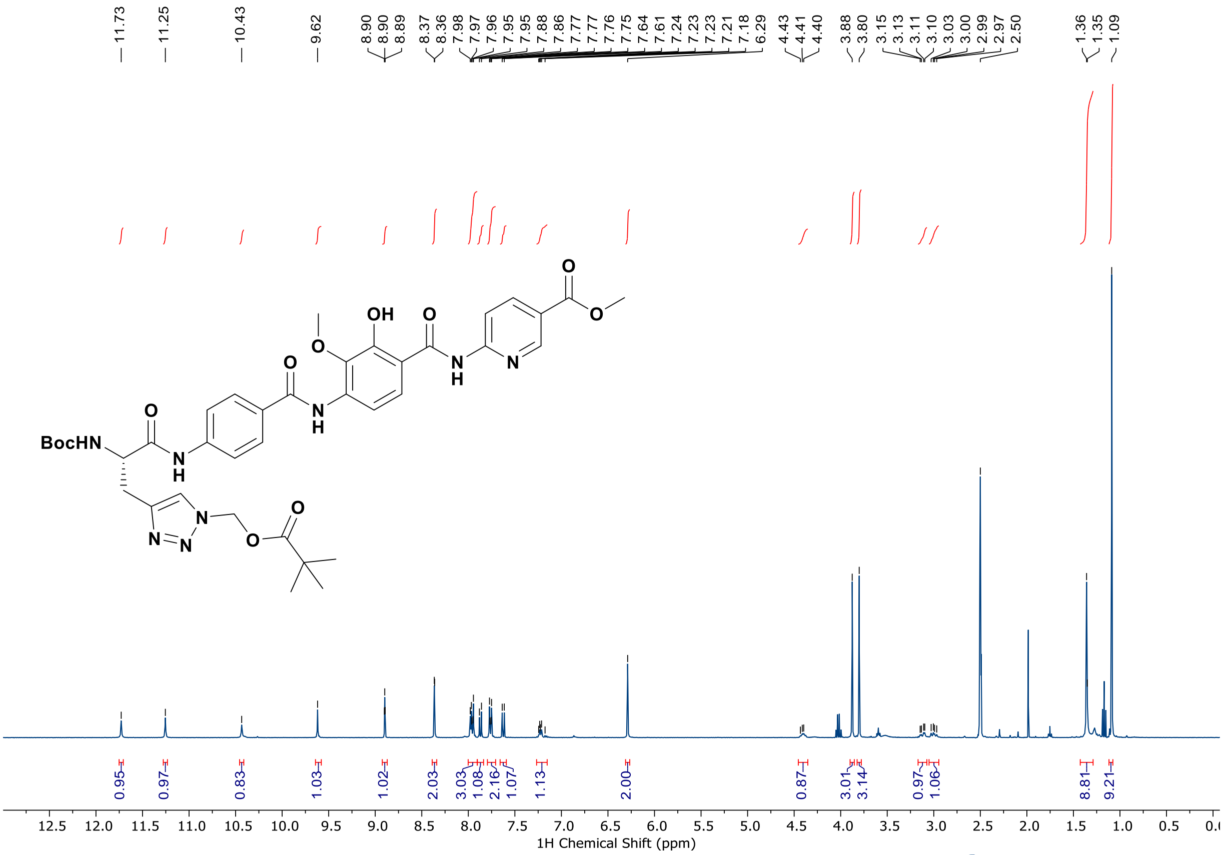

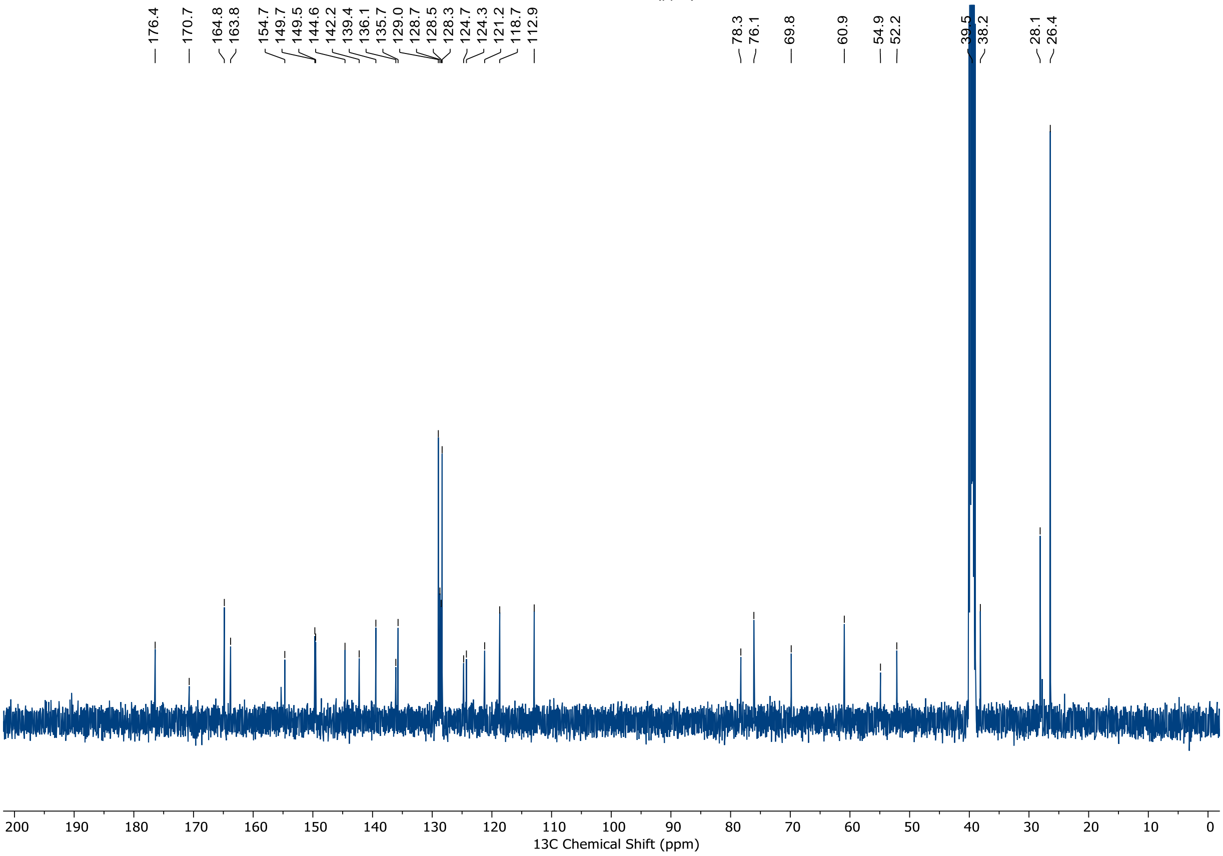

methyl (S)-6-(4-(4-(2-amino-3-(1-((pivaloyloxy)methyl)-1H-1,2,3-triazol-4-yl)propanamido)benzamido)-2-hydroxy-3-methoxybenzamido)nicotinate (37b)

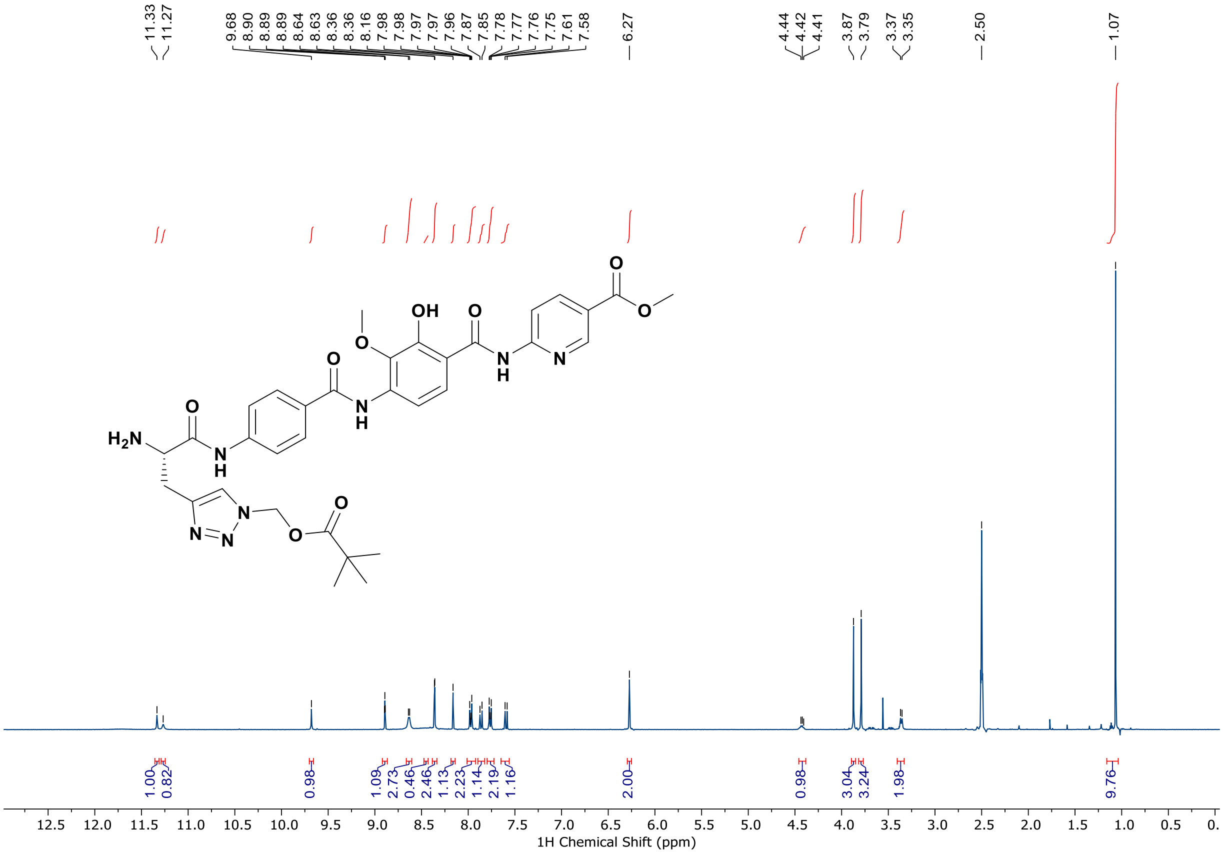

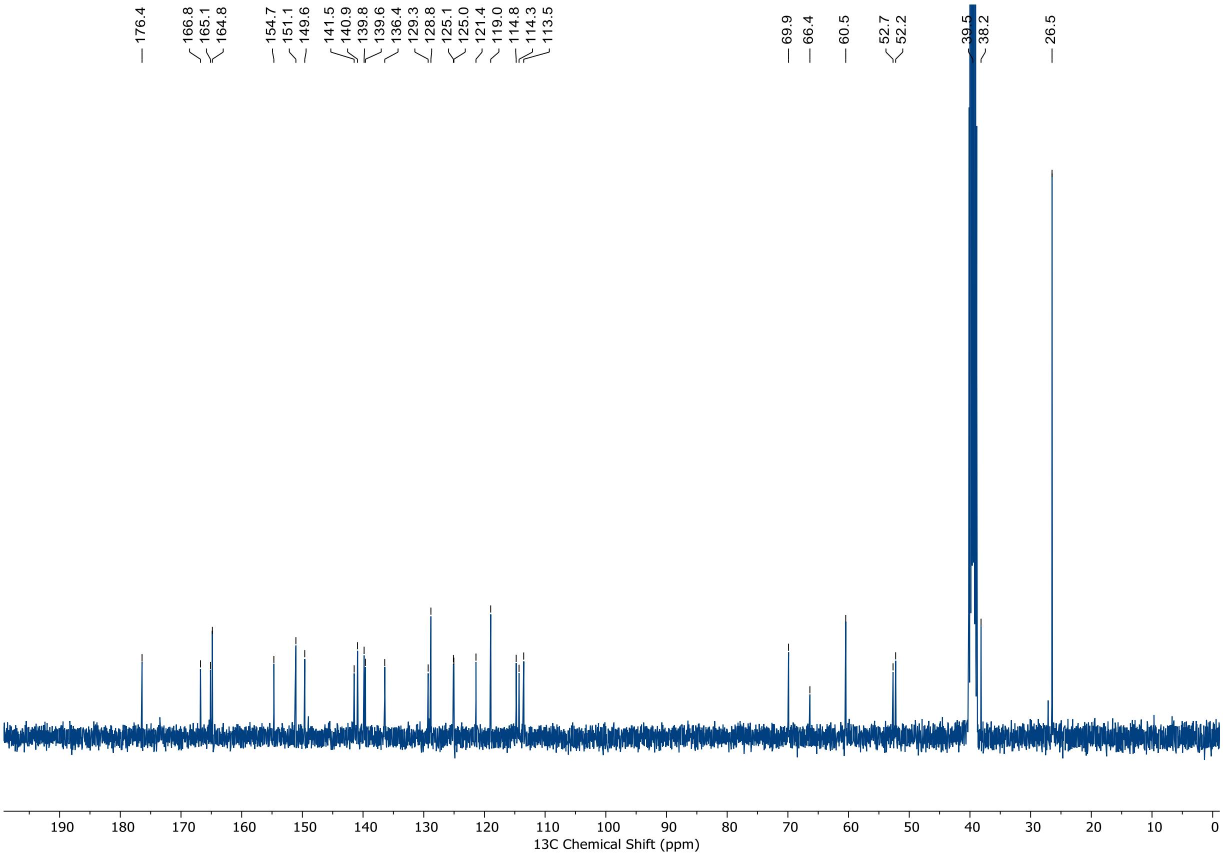

(S,E)-6-(2-hydroxy-4-(4-(2-(4-(3-(4-hydroxyphenyl)-2-methylacrylamido)benzamido)-3-(1H-1,2,3-triazol-4-yl)propanamido)benzamido)-3-methoxybenzamido)nicotinic acid (13)

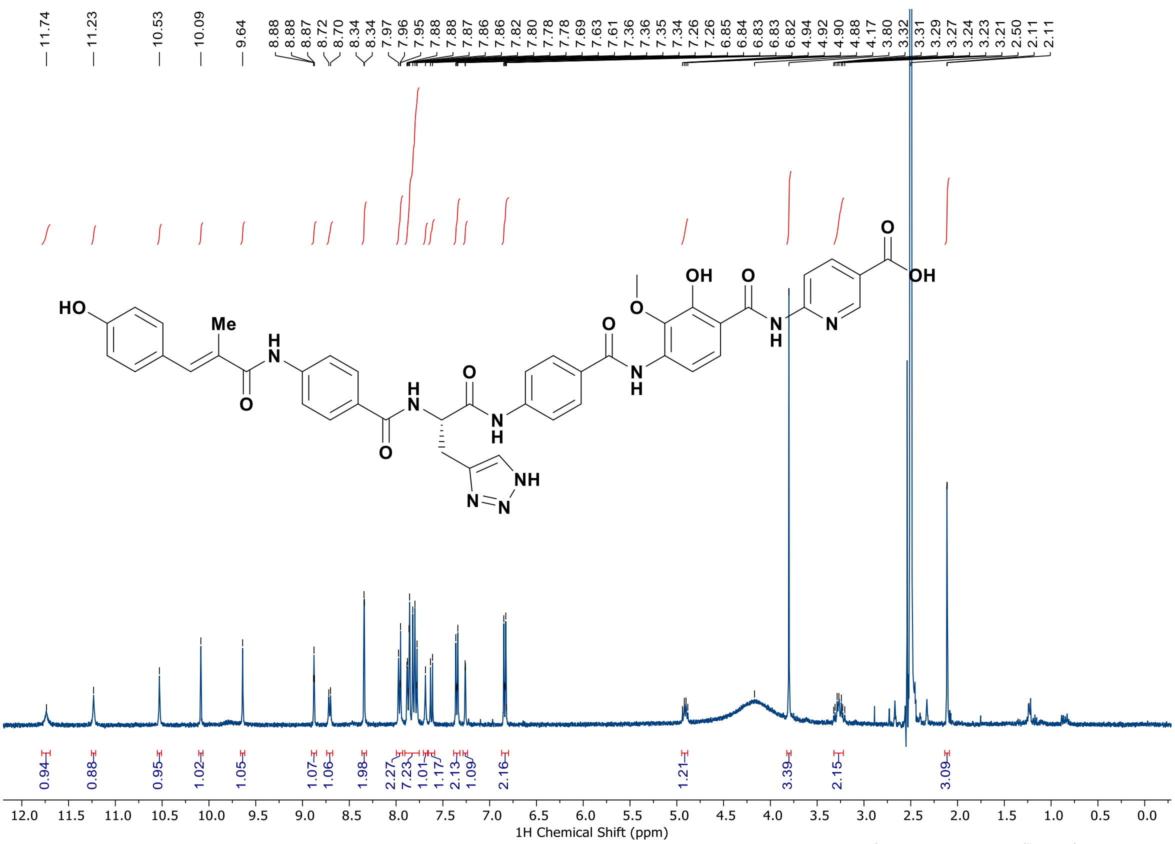

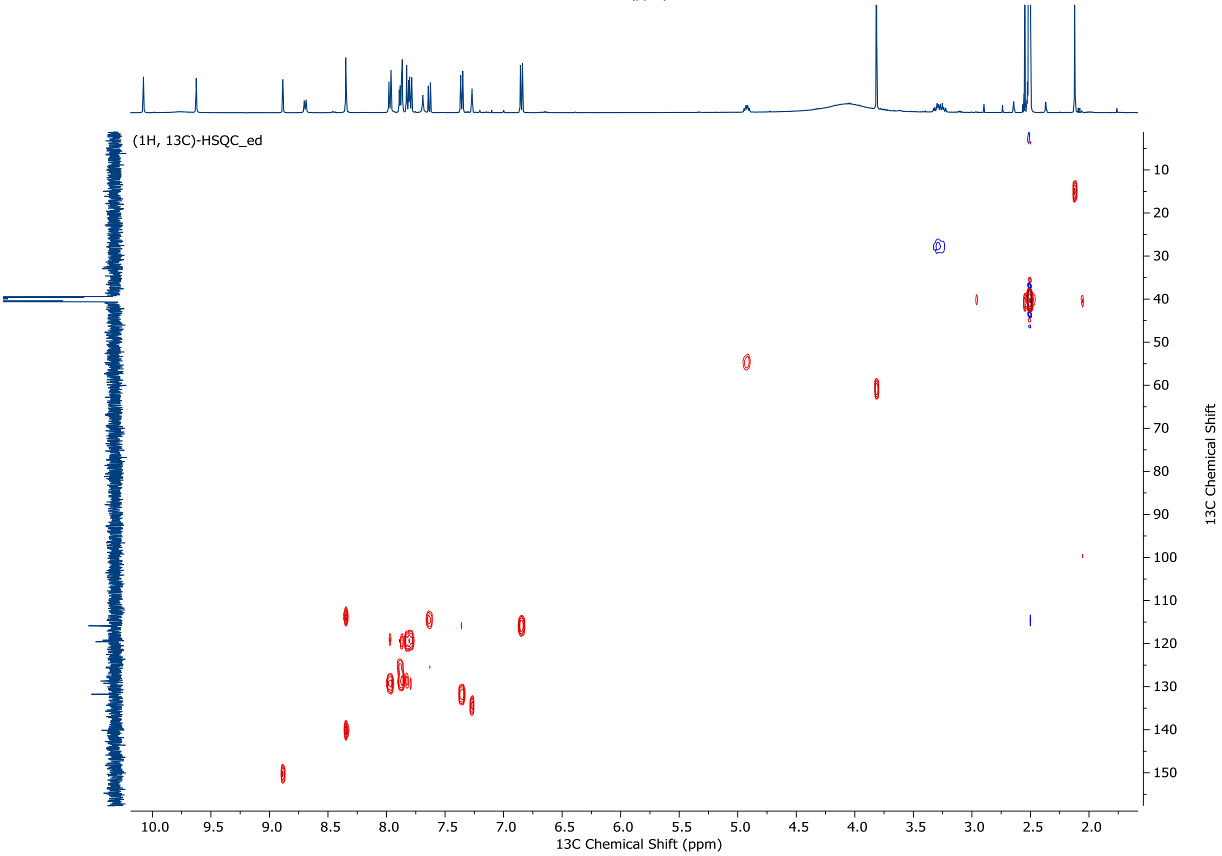

2-(benzyloxy)-3-methoxy-4-nitro-N-phenylbenzamide (24c-1)

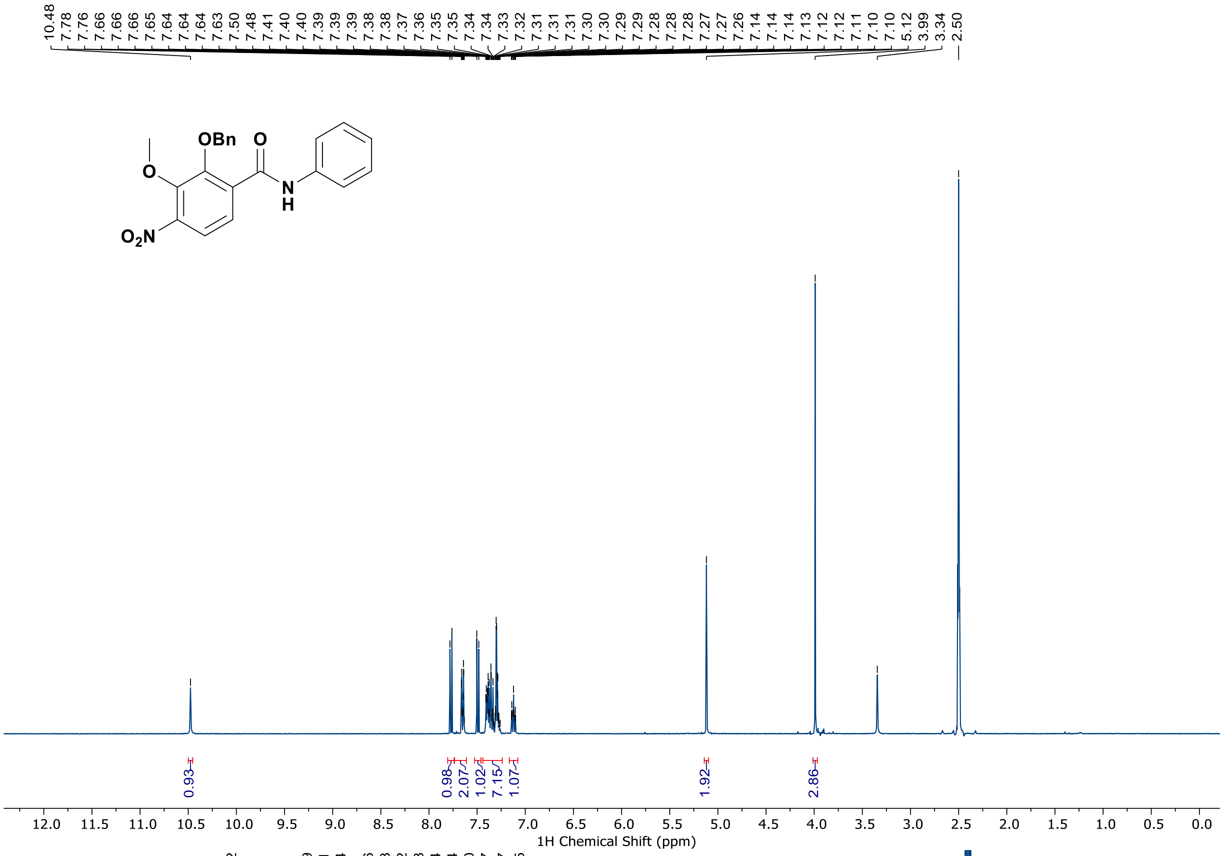

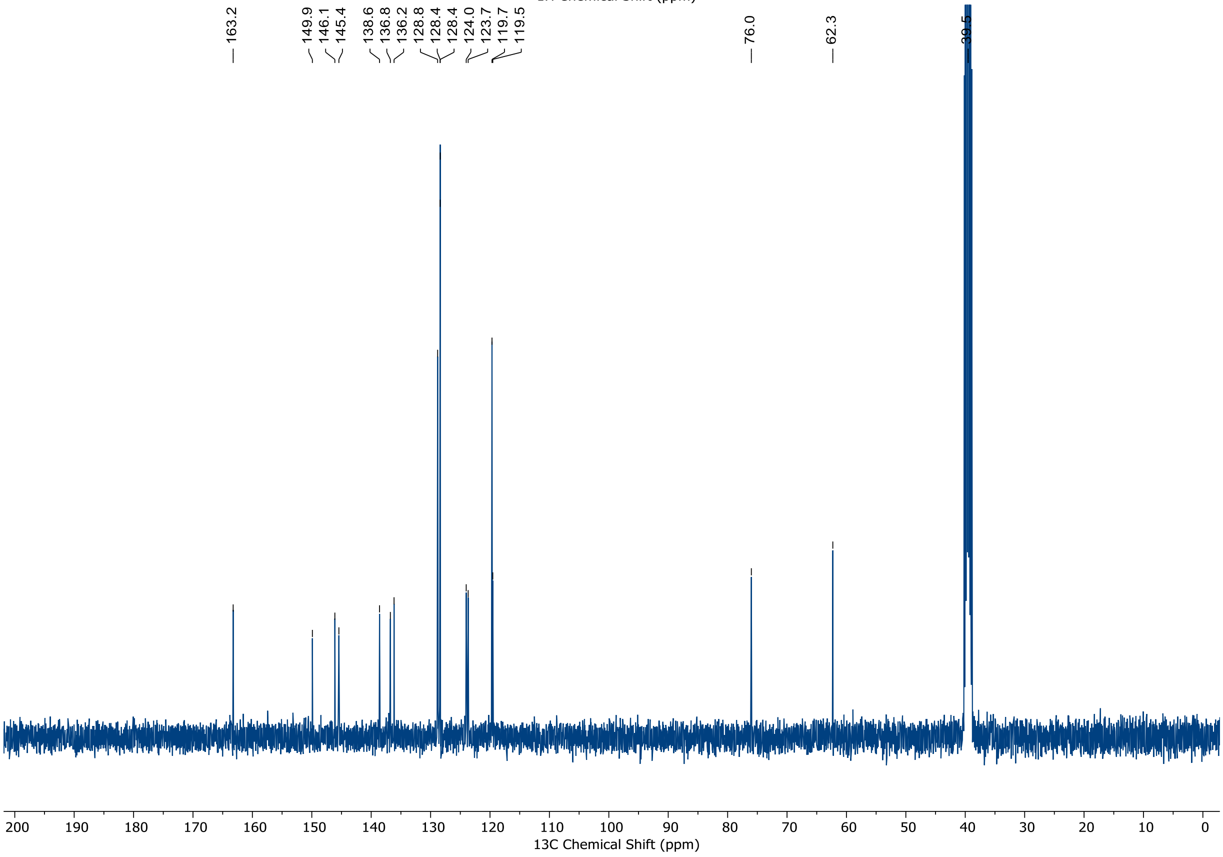

4-amino-2-(benzyloxy)-3-methoxy-N-phenylbenzamide (35a)

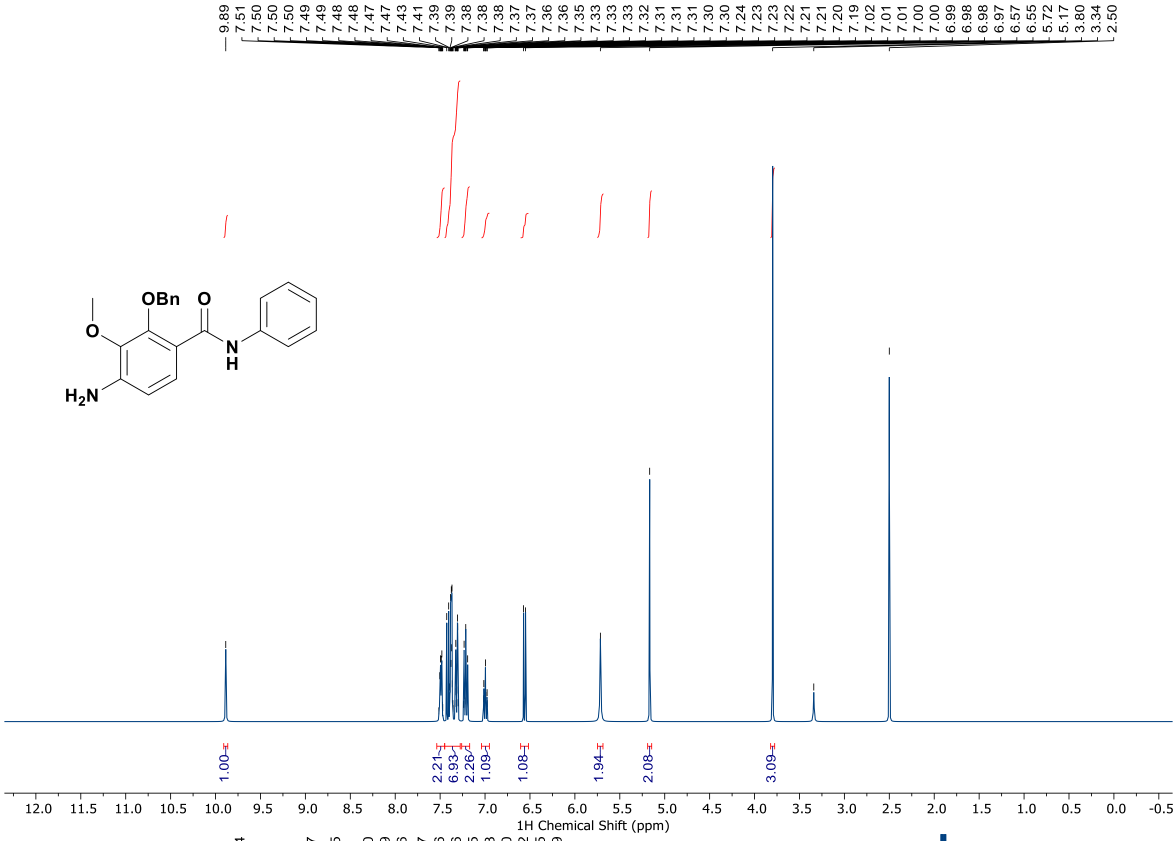

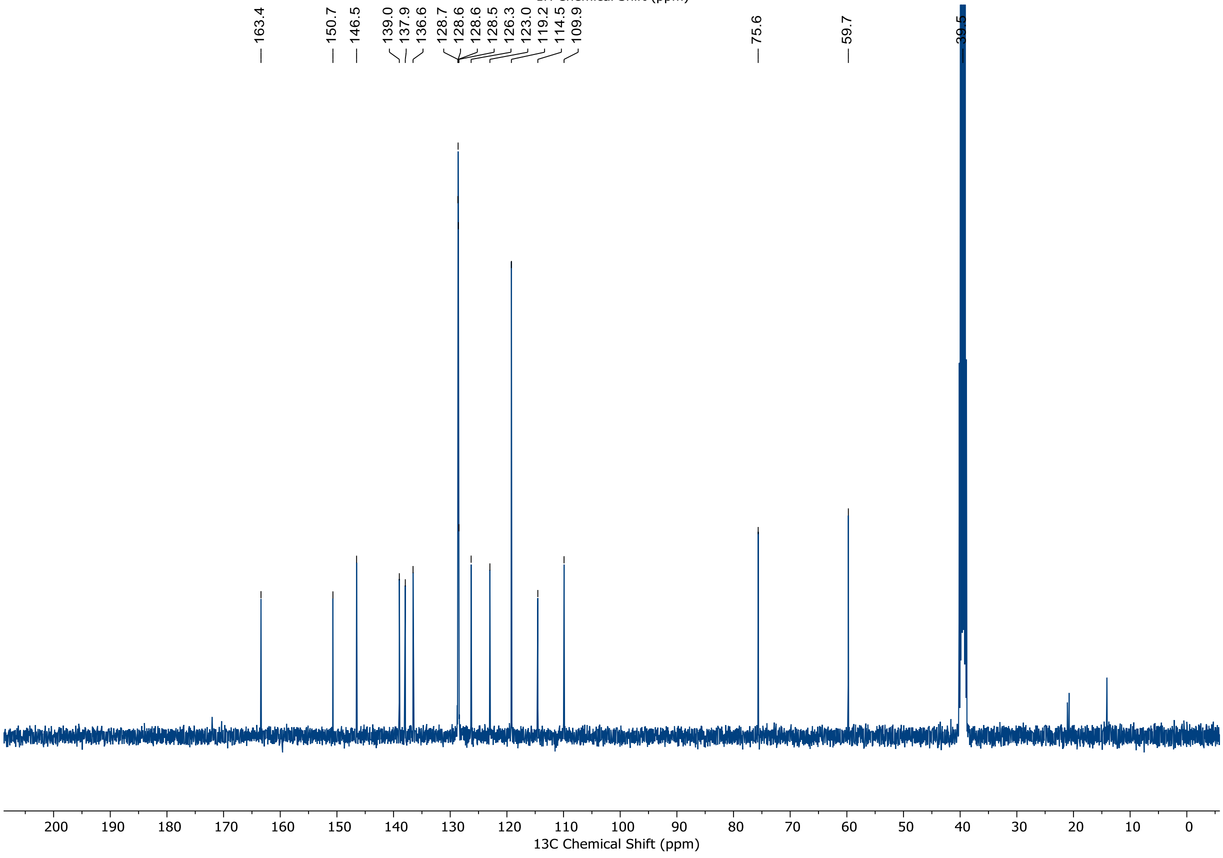

2-(benzyloxy)-3-methoxy-4-(4-nitrobenzamido)-N-phenylbenzamide (35a-1)

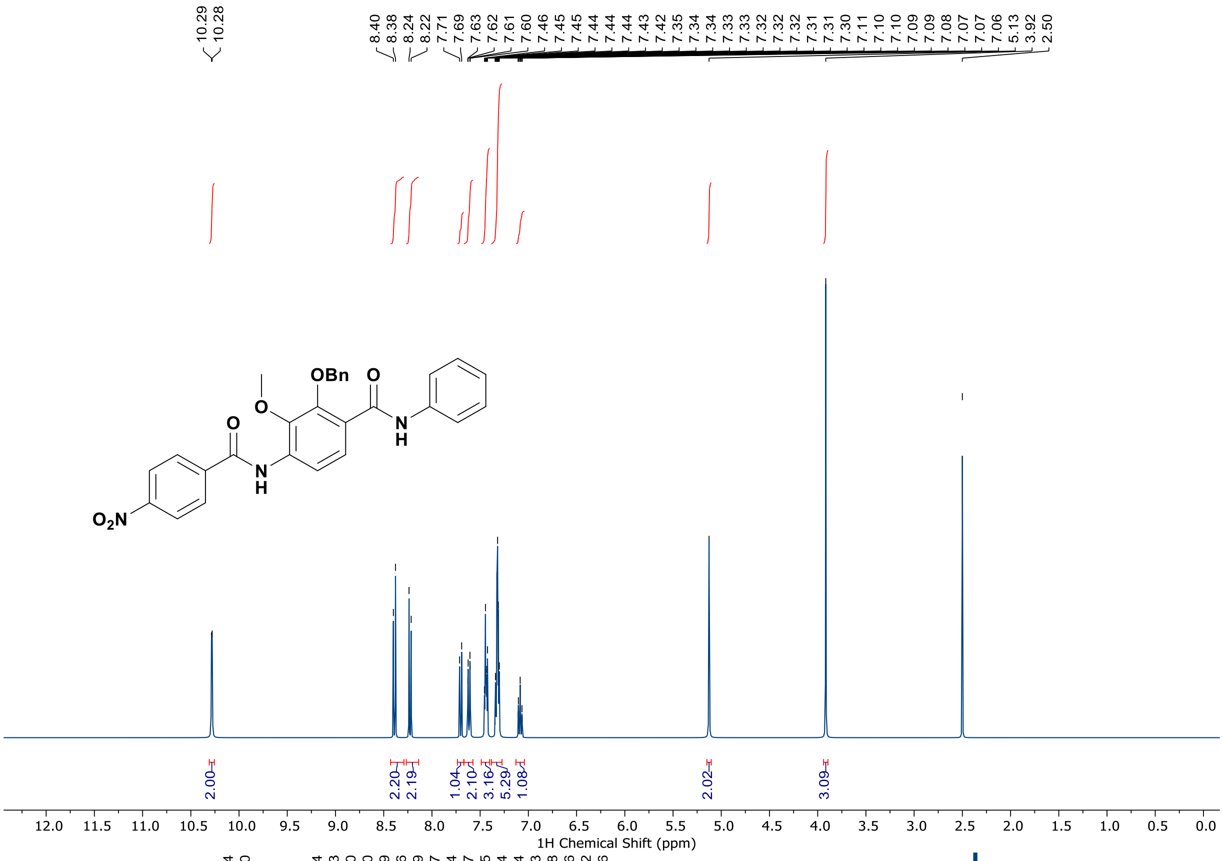

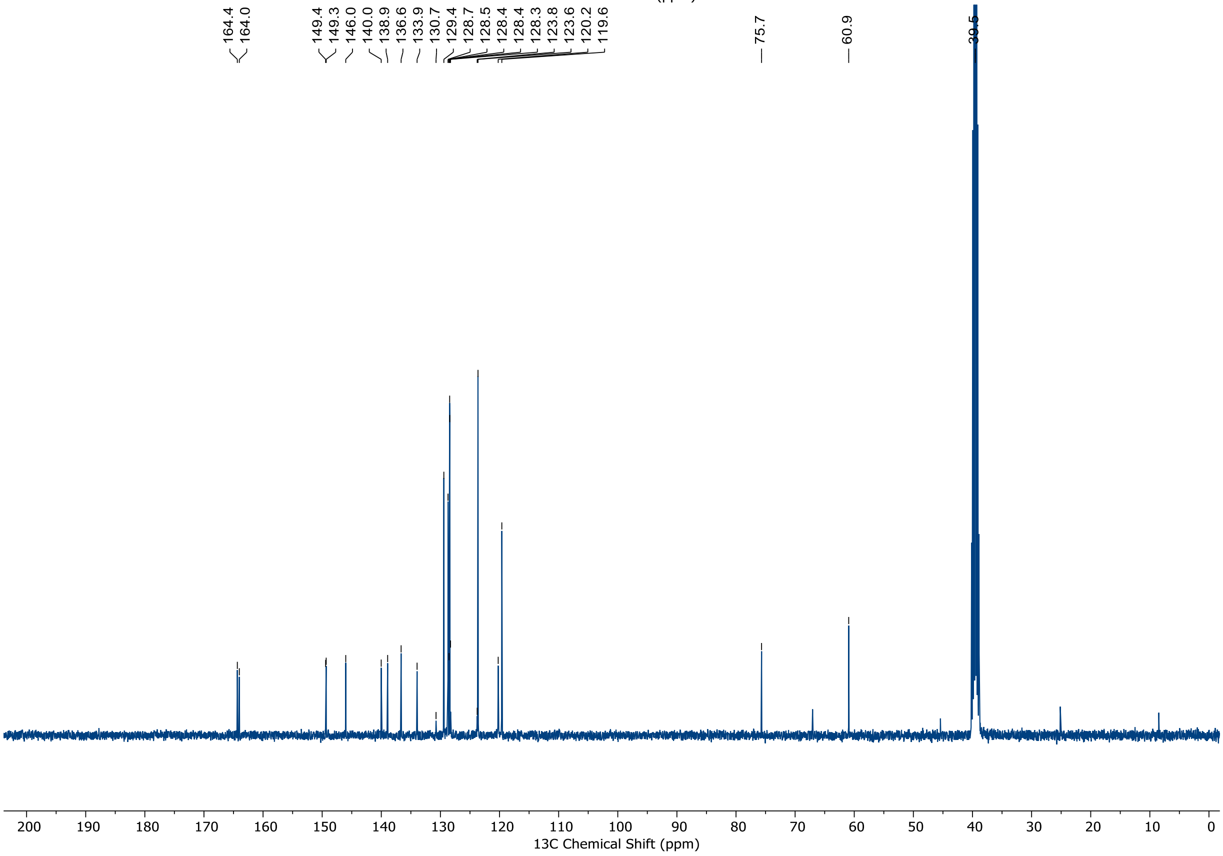

4-(4-aminobenzamido)-2-(benzyloxy)-3-methoxy-N-phenylbenzamide (36e)

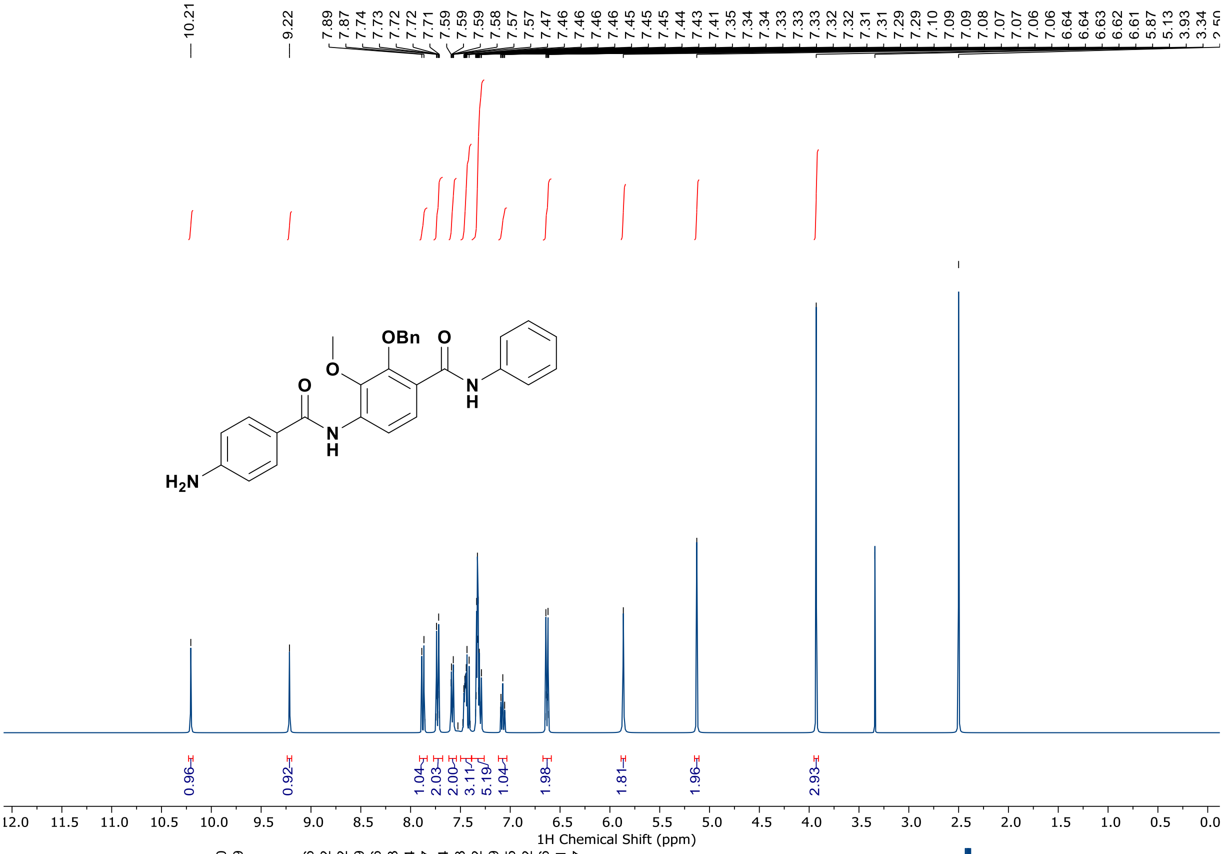

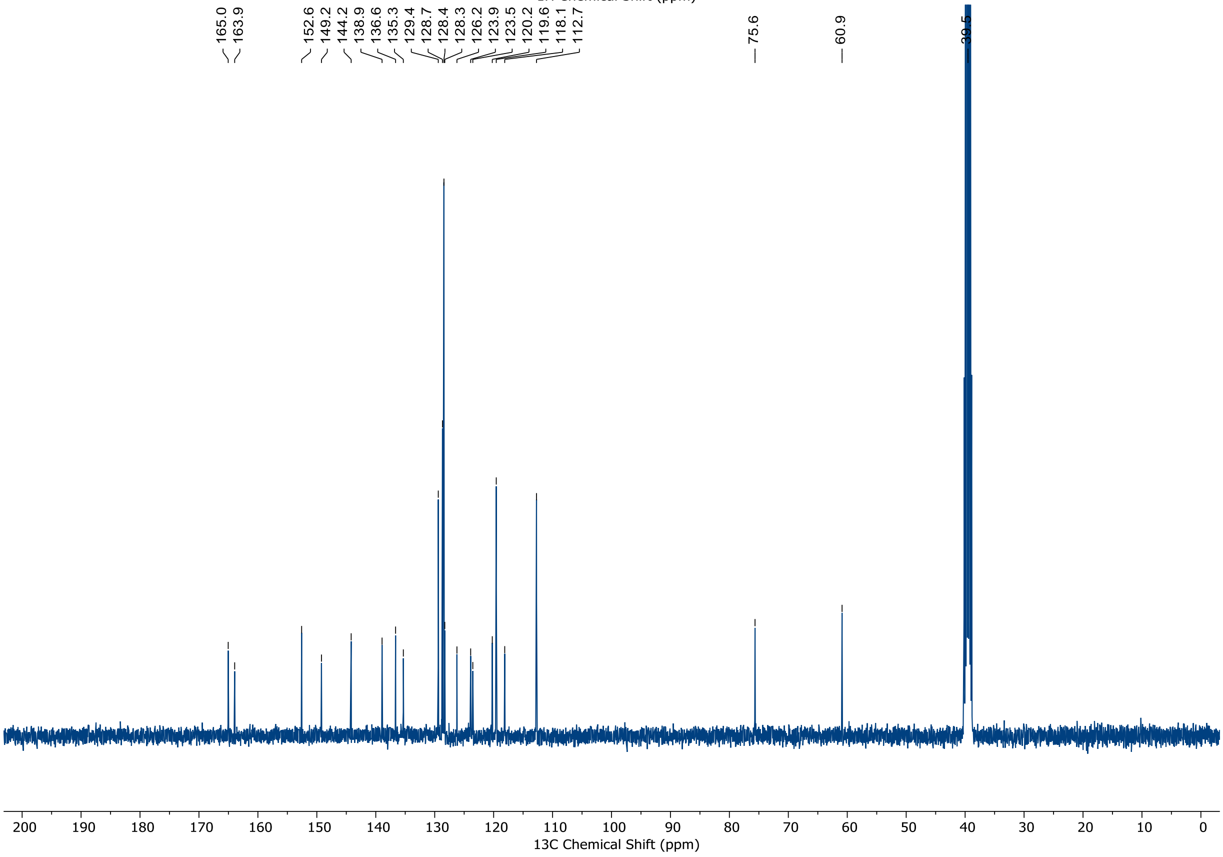

(S)-(4-(3-((4-((3-(benzyloxy)-2-methoxy-4-(phenylcarbamoyl)phenyl)carbamoyl)phenyl)amino)-2-((tert-butoxycarbonyl)amino)-3-oxopropyl)-1H-1,2,3-triazol-1-yl)methyl pivalate (36e-1)

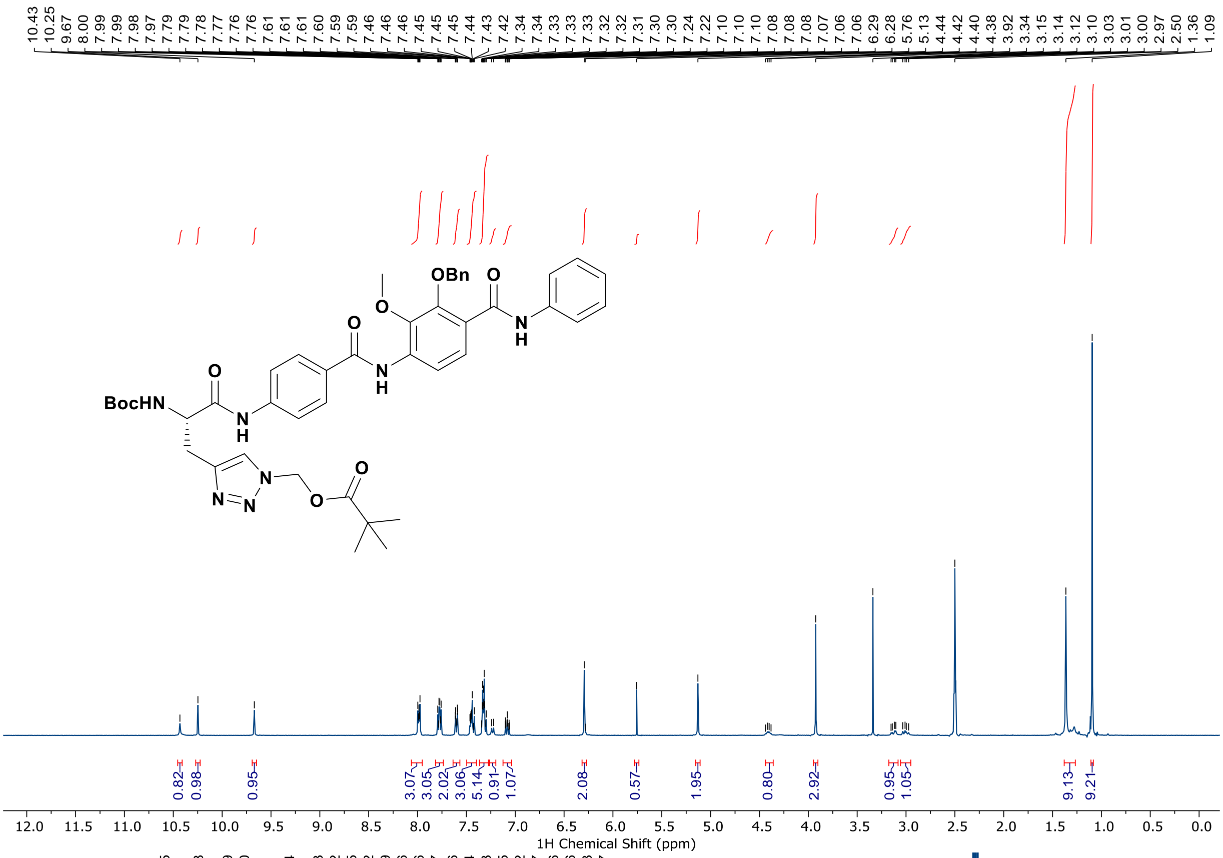

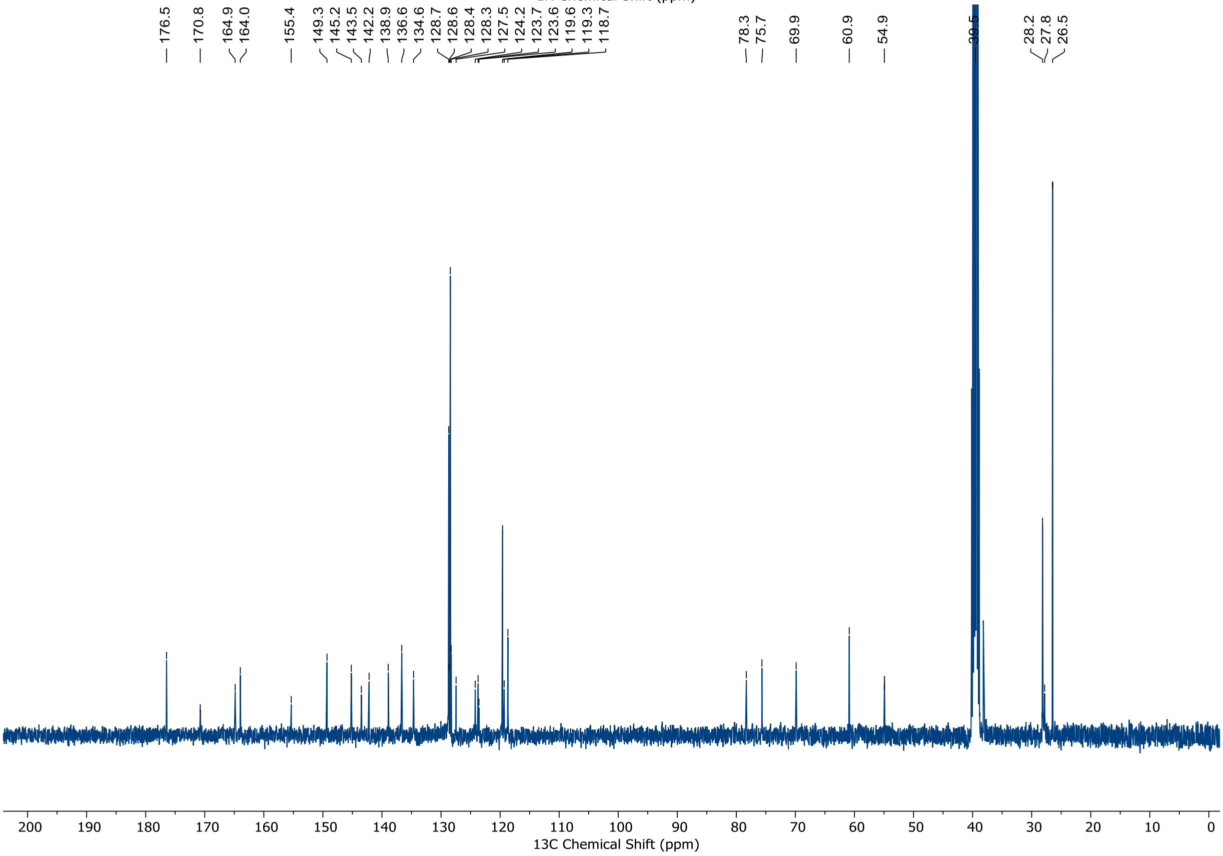

(S)-(4-(2-((tert-butoxycarbonyl)amino)-3-((4-((3-hydroxy-2-methoxy-4-(phenylcarbamoyl)phenyl)carbamoyl)phenyl)amino)-3-oxopropyl)-1H-1,2,3-triazol-1-yl)methyl pivalate (36e-2)

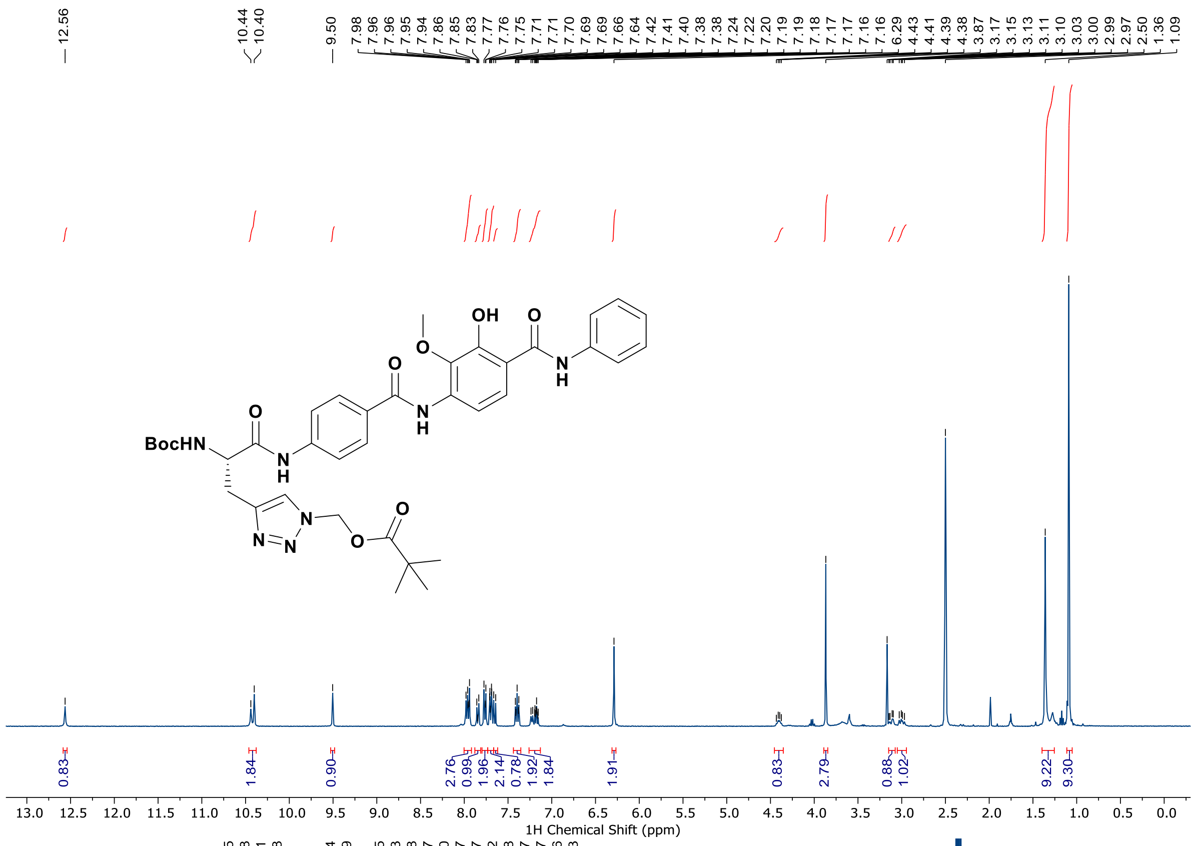

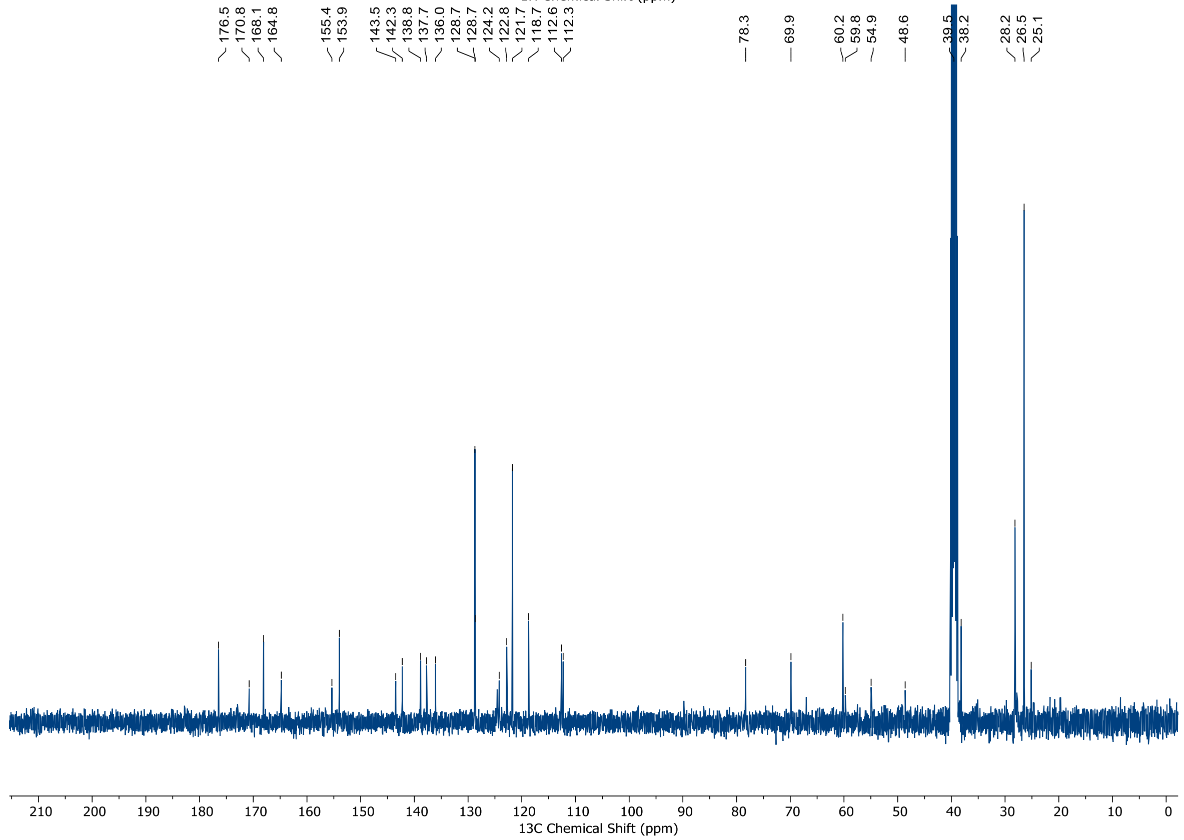

(S)-(4-(2-amino-3-((4-((3-hydroxy-2-methoxy-4-(phenylcarbamoyl)phenyl)carbamoyl)phenyl)amino)-3-oxopropyl)-1H-1,2,3-triazol-1-yl)methyl pivalate (37e)

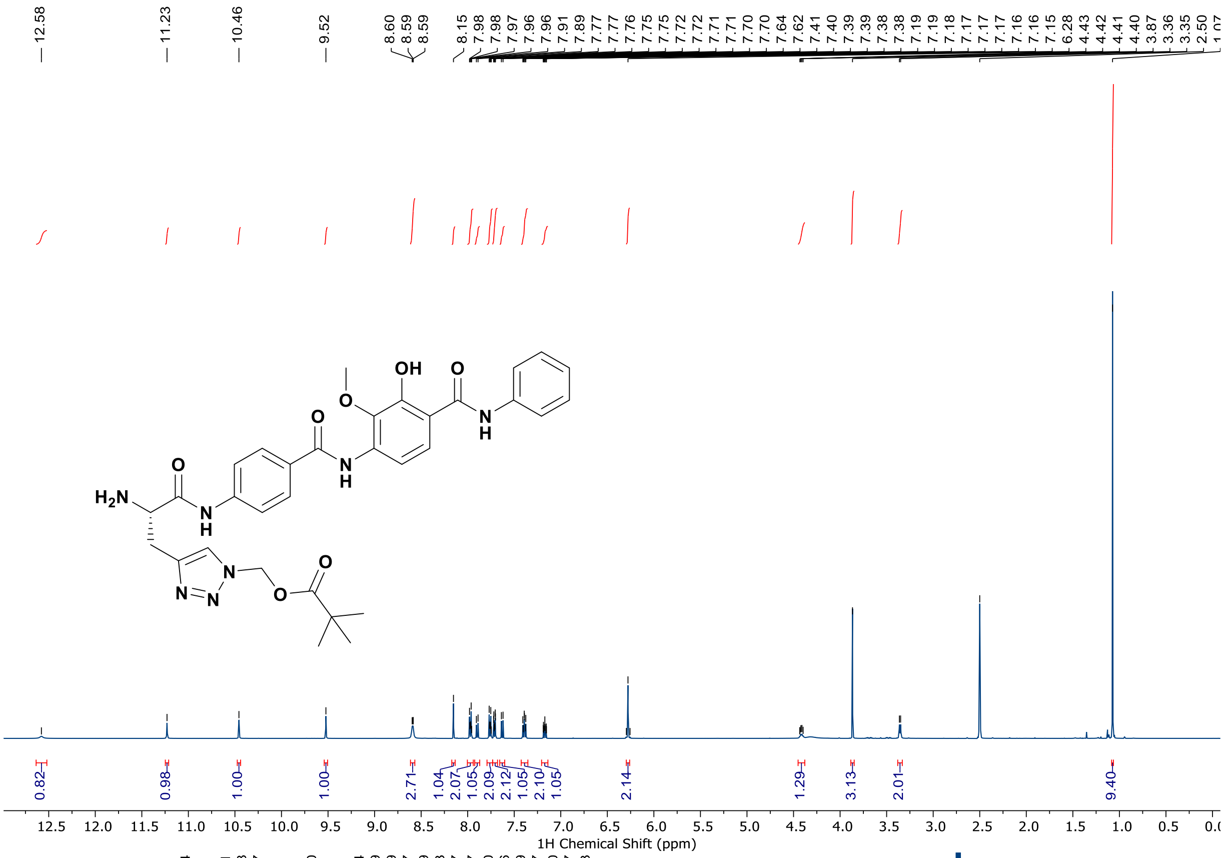

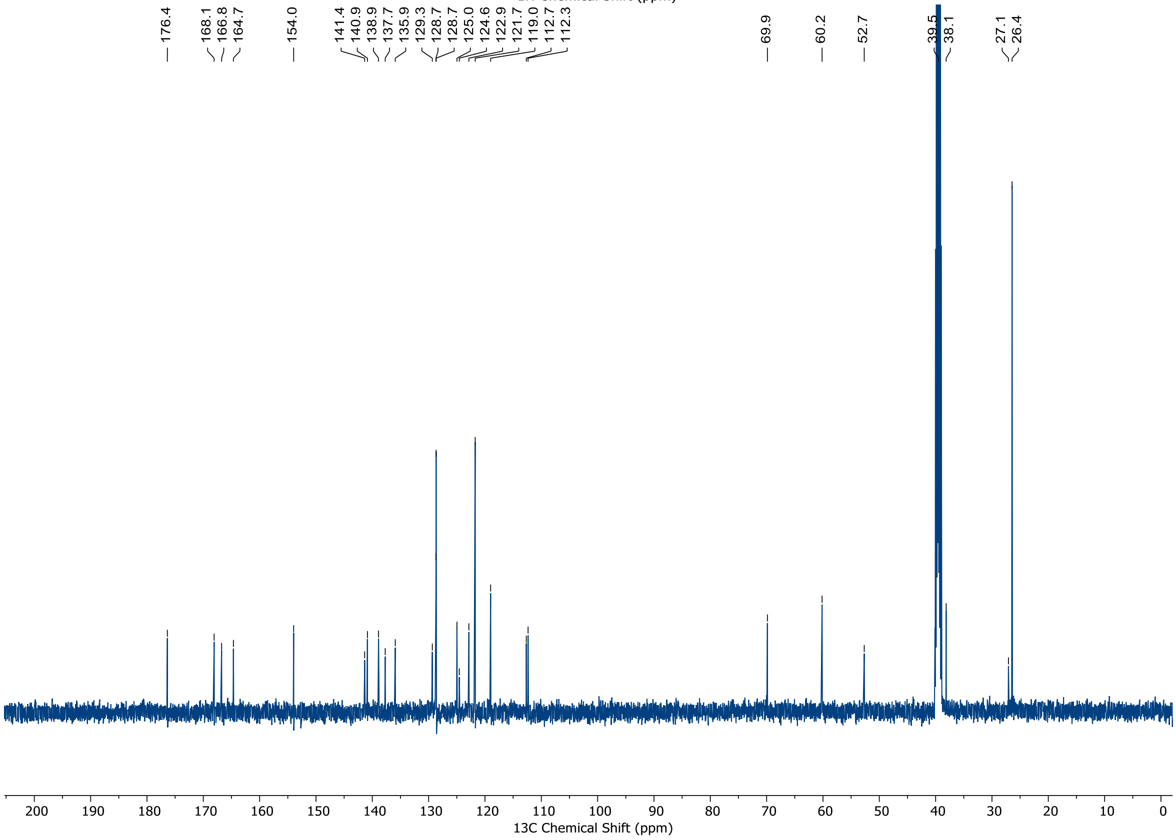

(S,E)-2-hydroxy-4-(4-(2-(4-(3-(4-hydroxyphenyl)-2-methylacrylamido)benzamido)-3-(1H-1,2,3-triazol-4-yl)propanamido)benzamido)-3-methoxy-N-phenylbenzamide (15)

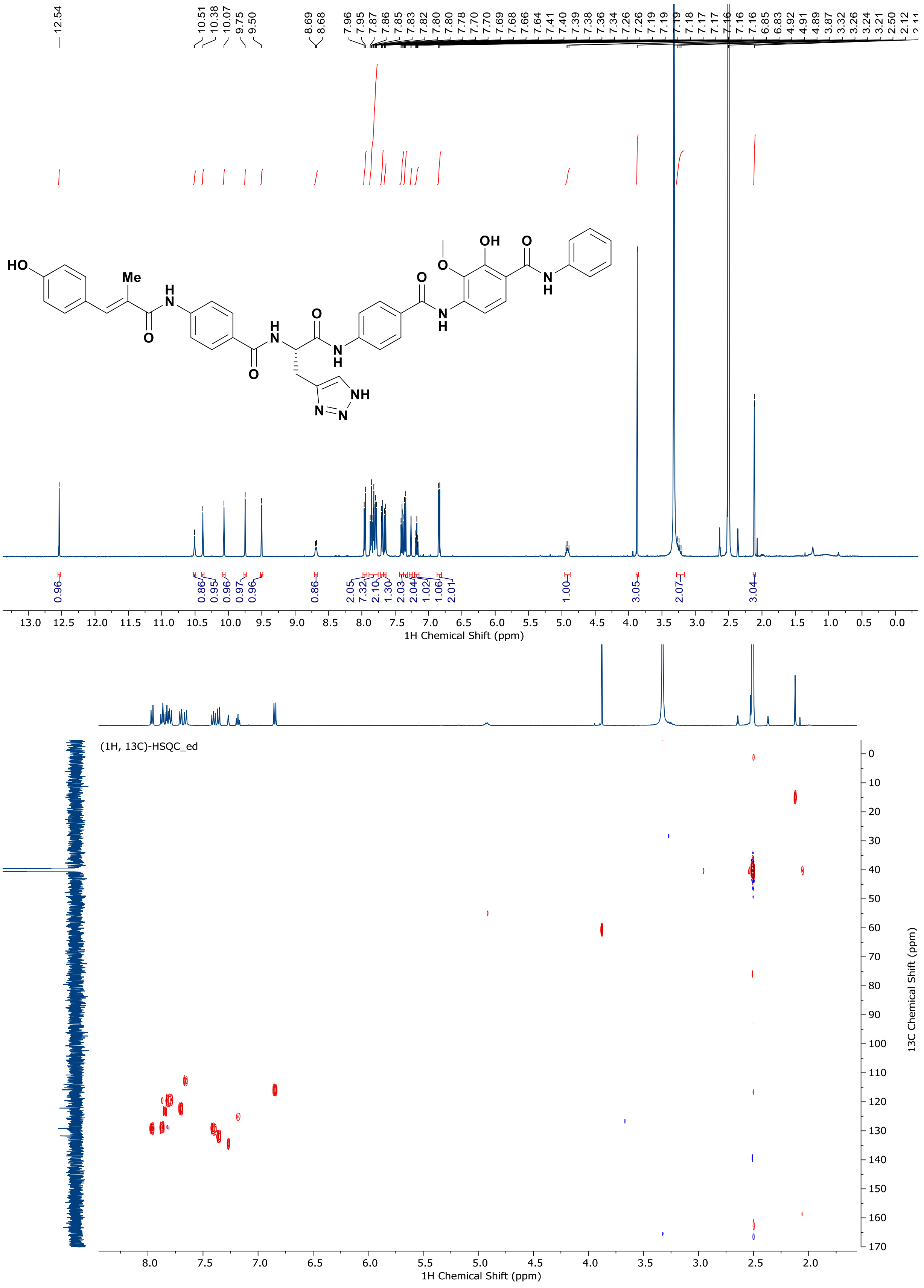

3.4 NMR spectra of E-Variations (9, 10) and reaction intermediates

allyl 2-(allyloxy)-4-amino-3-methoxybenzoate (28a)

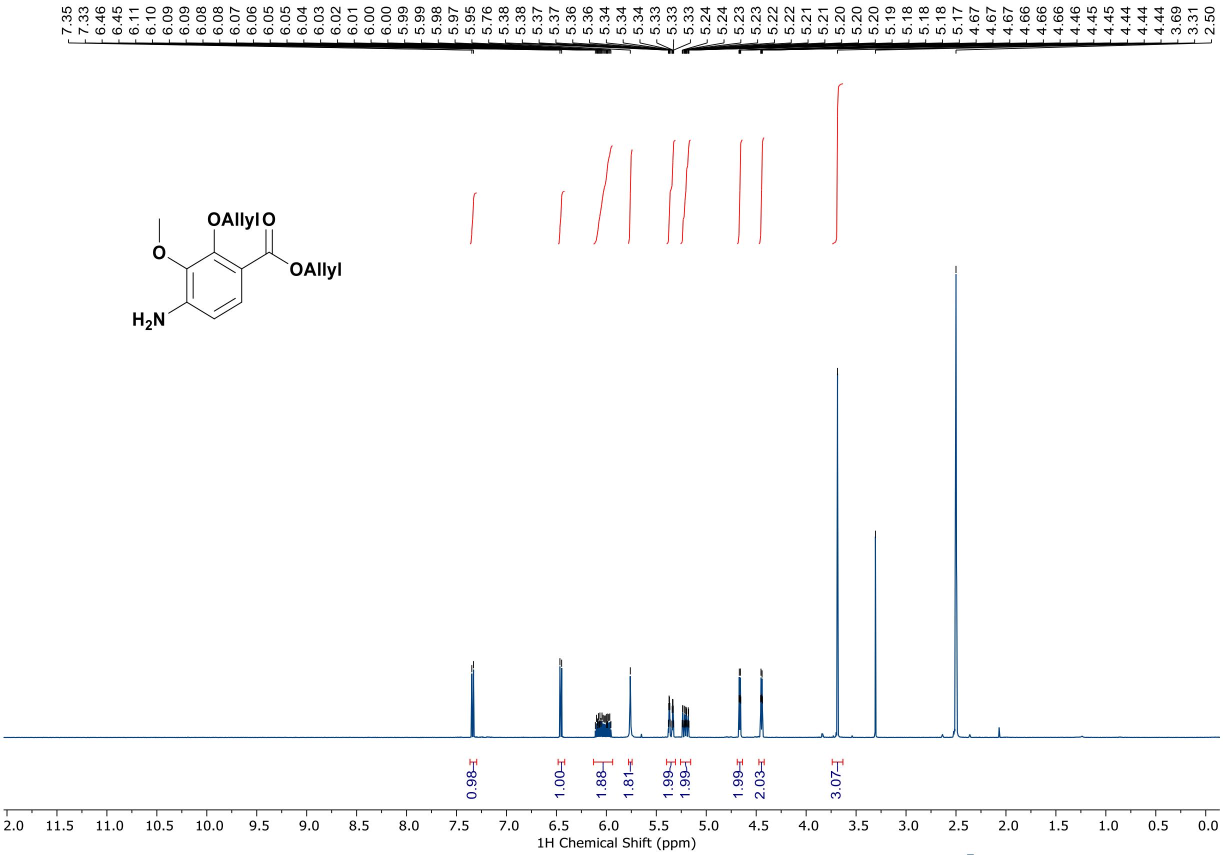

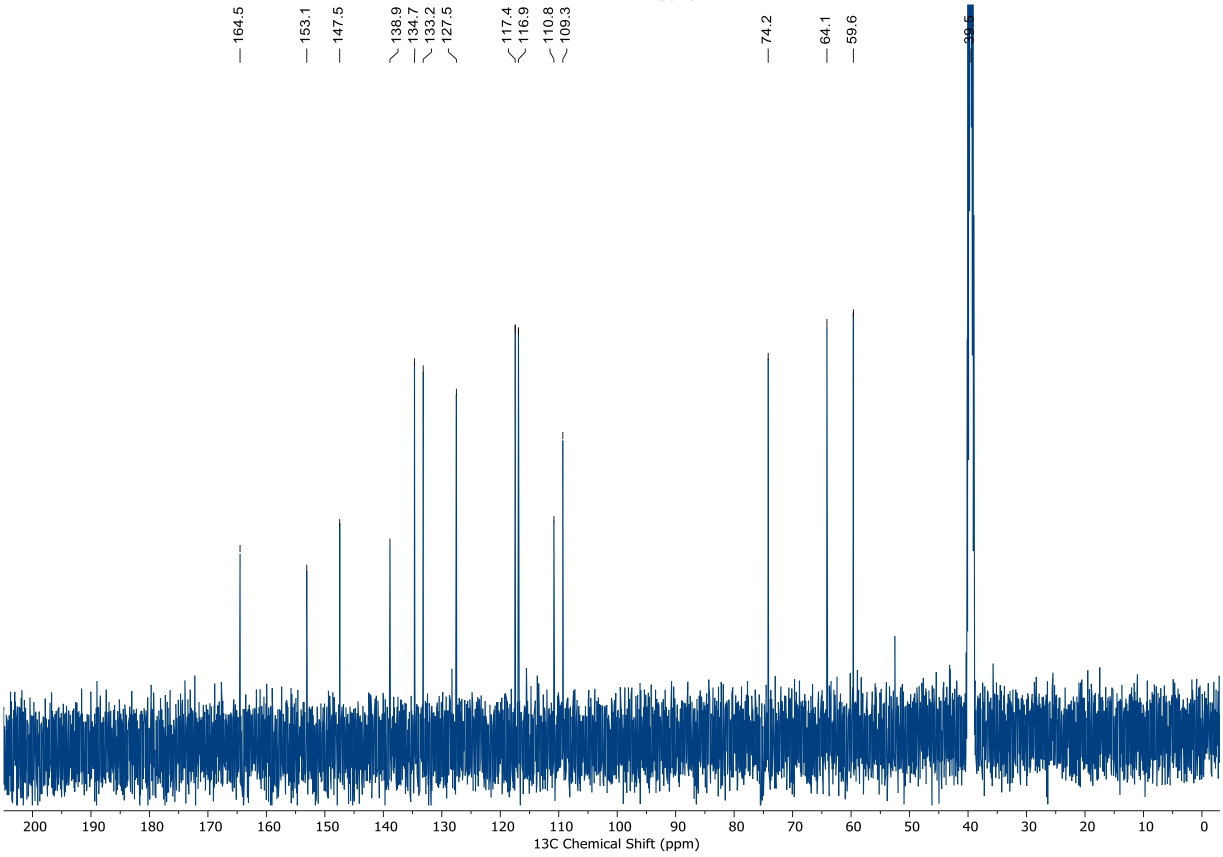

allyl 2-(allyloxy)-3-methoxy-4-(5-nitropicolinamido)benzoate (29a)

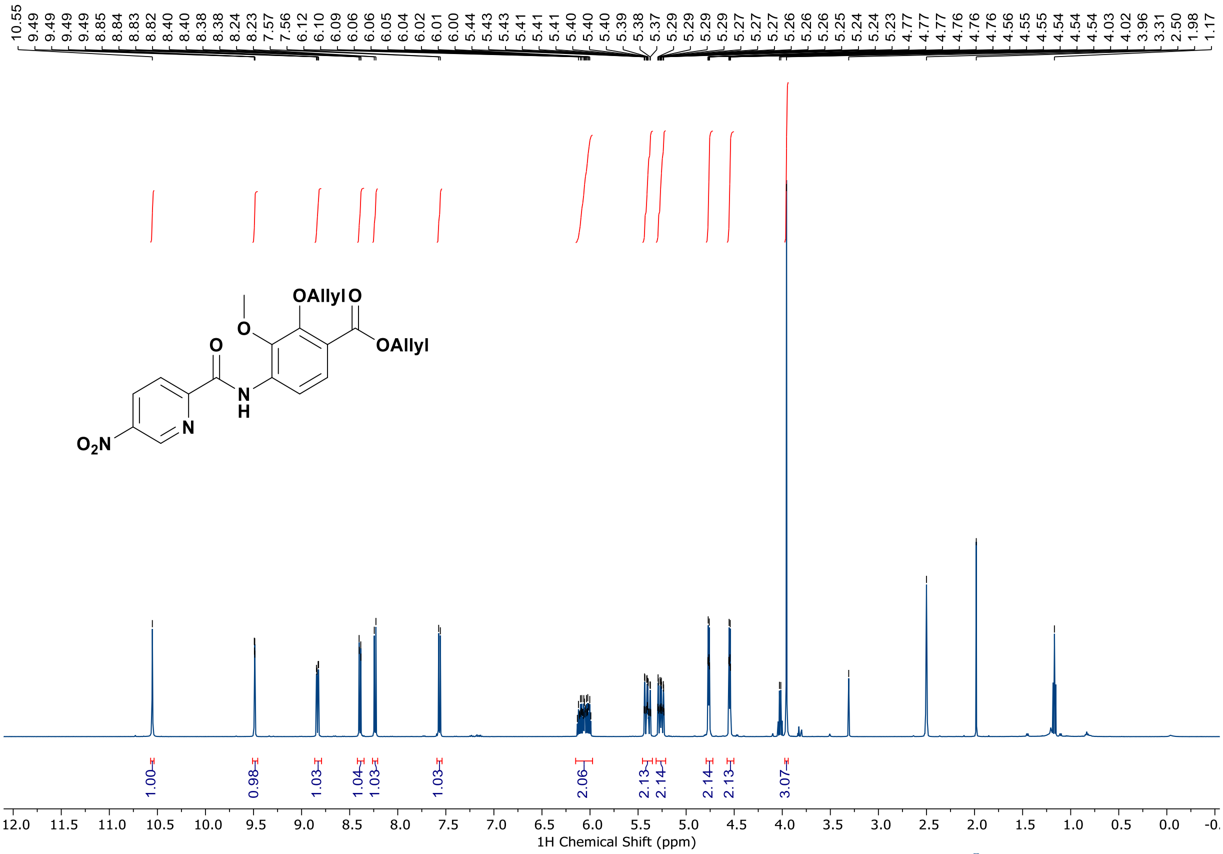

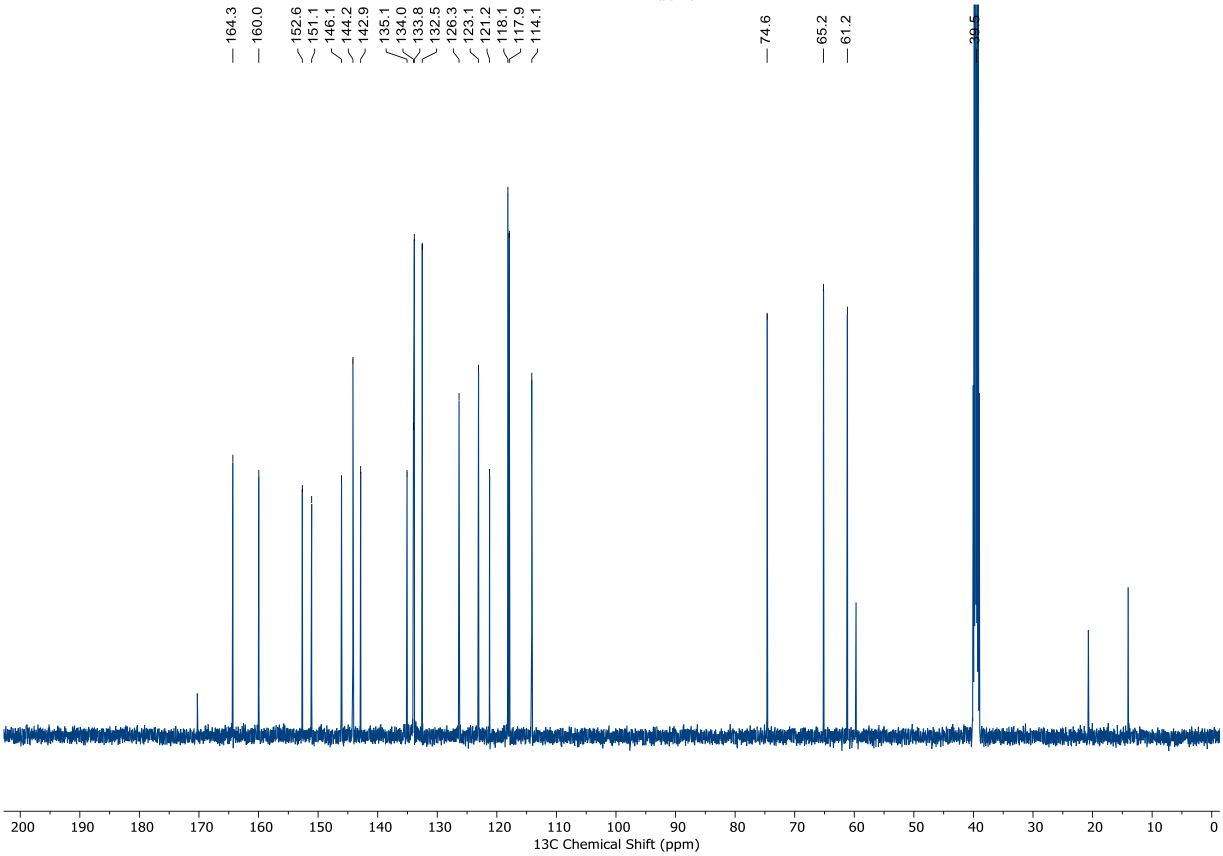

allyl 2-(allyloxy)-4-(5-aminopicolinamido)-3-methoxybenzoate (33a)

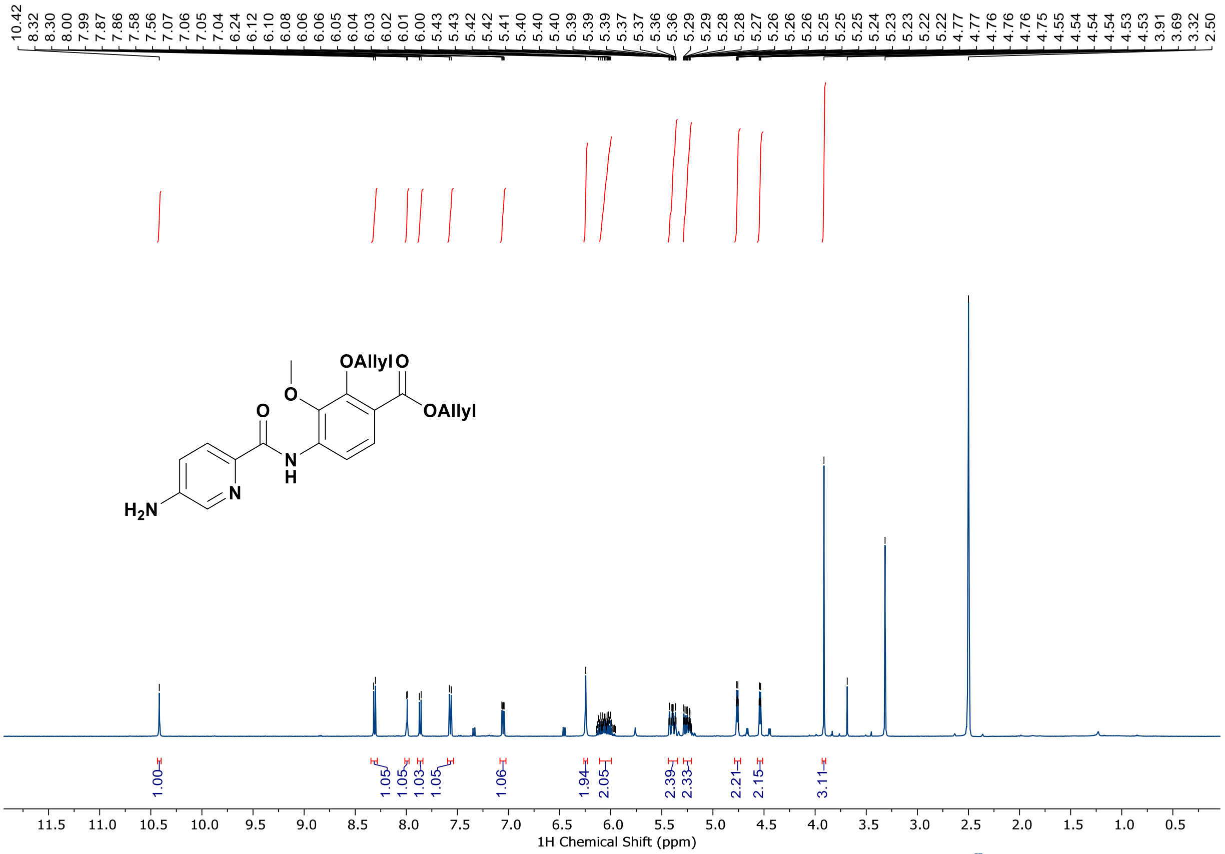

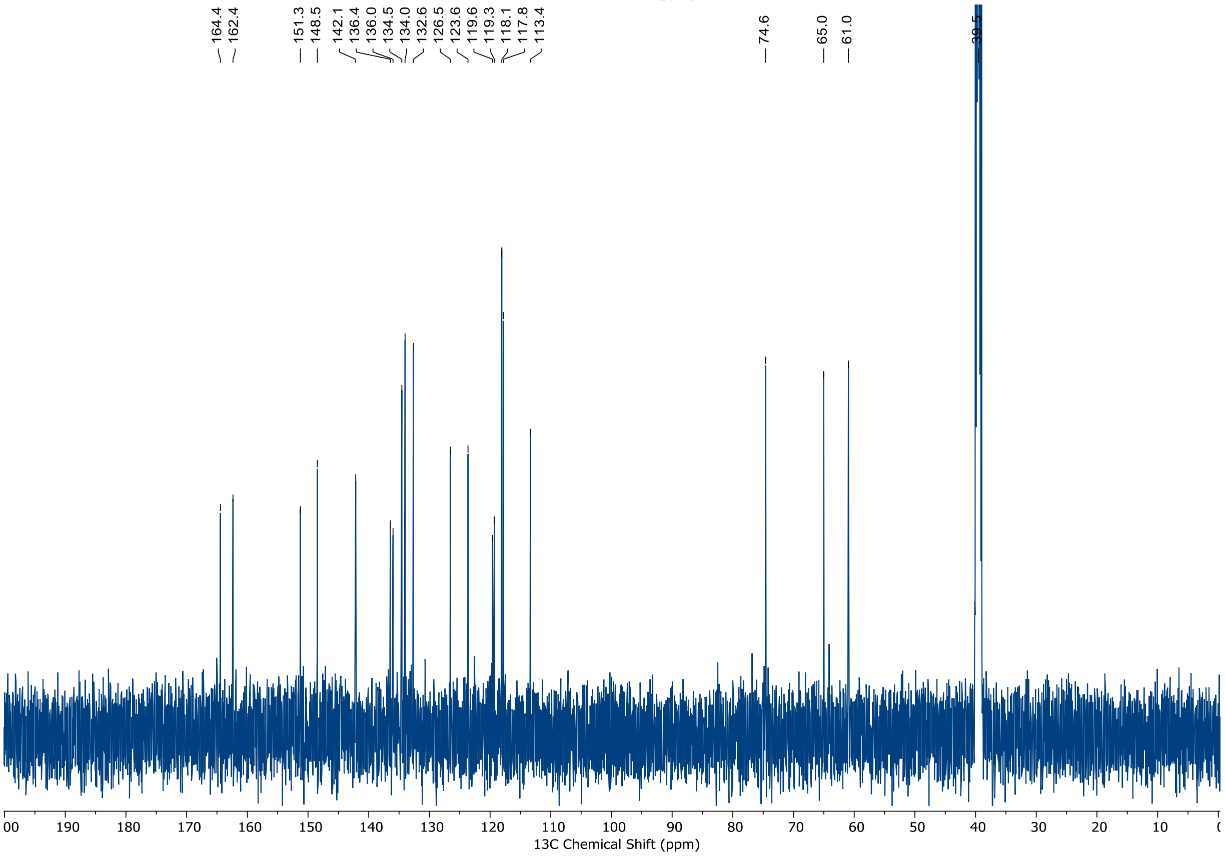

allyl 2-(allyloxy)-3-methoxy-4-(5-(4-nitrobenzamido)picolinamido)benzoate (33a-1)

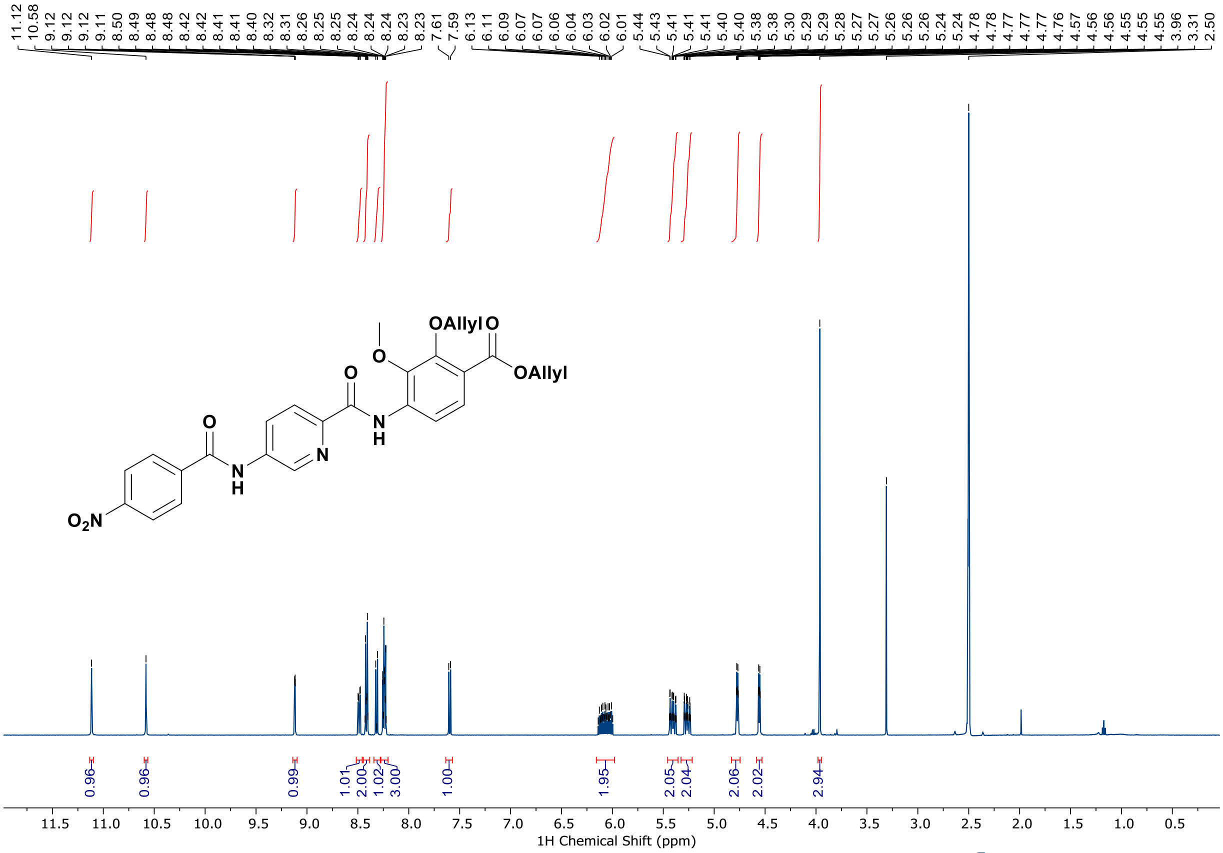

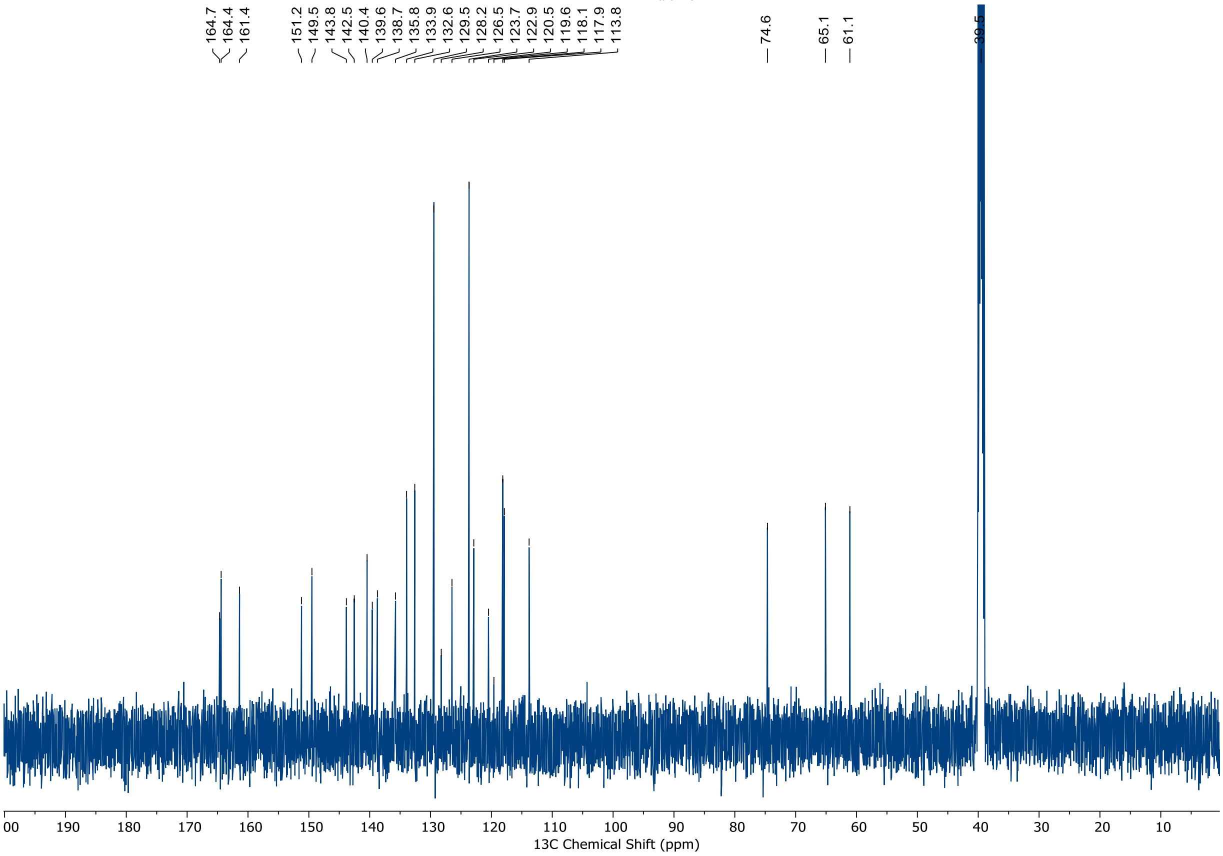

allyl 2-(allyloxy)-4-(5-(4-aminobenzamido)picolinamido)-3-methoxybenzoate (36c)

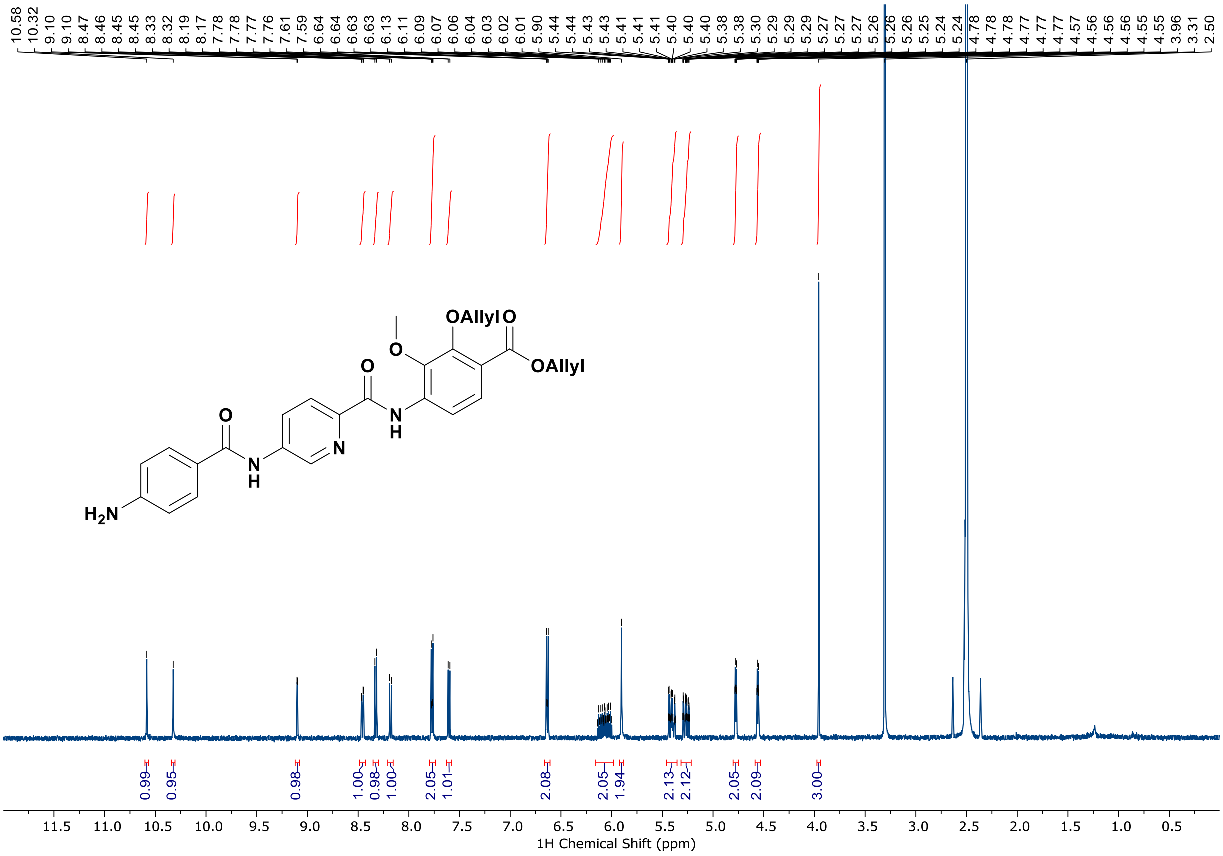

allyl (S)-2-(allyloxy)-4-(5-(4-(2-((tert-butoxycarbonyl)amino)-3-(1-((pivaloyloxy)methyl)-1H-1,2,3-triazol-4-yl)propanamido)benzamido)picolinamido)-3-methoxybenzoate (36c-1)

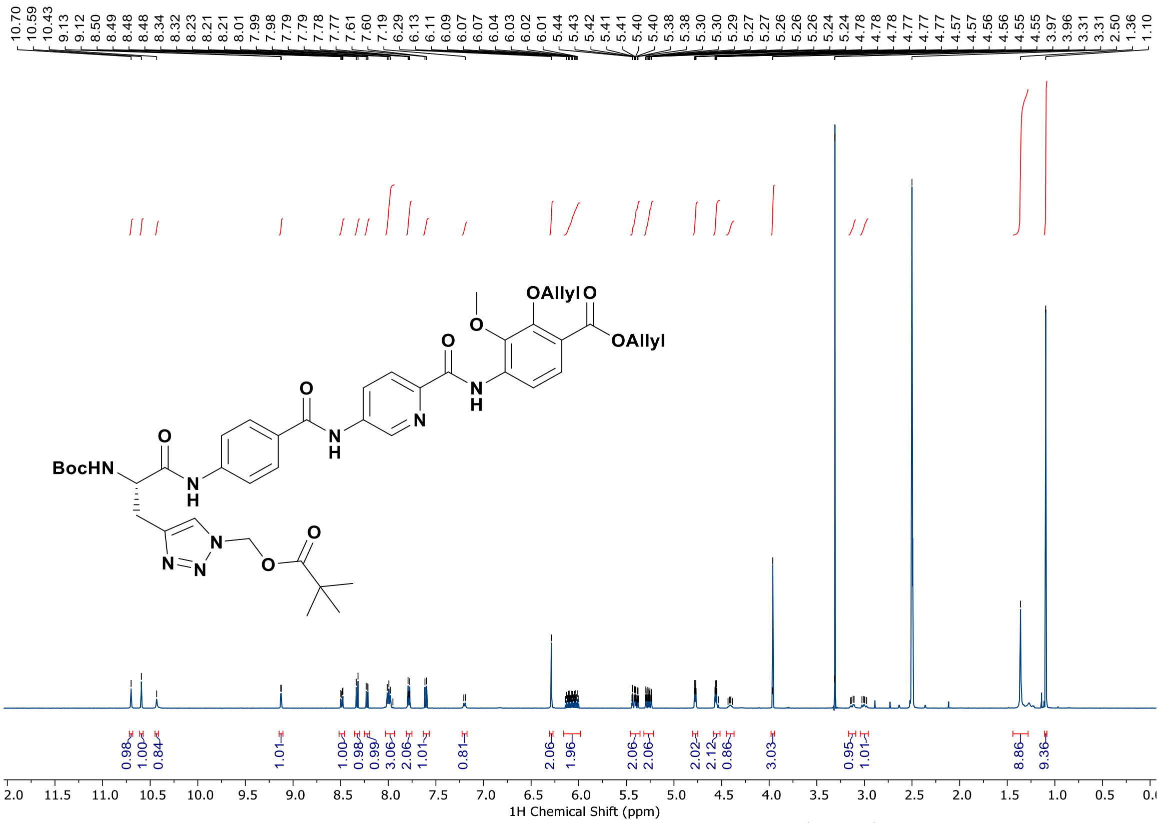

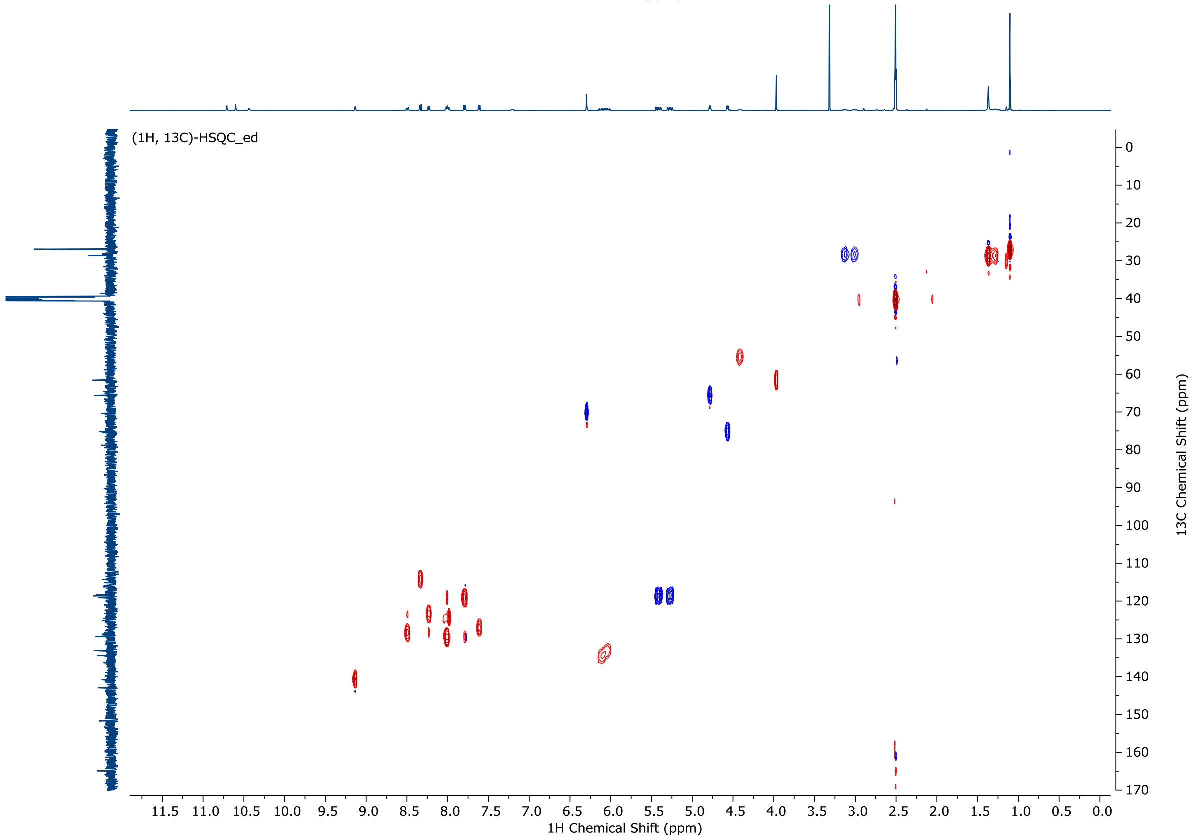

(S)-4-(5-(4-(2-((tert-butoxycarbonyl)amino)-3-(1-((pivaloyloxy)methyl)-1H-1,2,3-triazol-4-yl)propanamido)benzamido)picolinamido)-2-hydroxy-3-methoxybenzoic acid (36c-2)

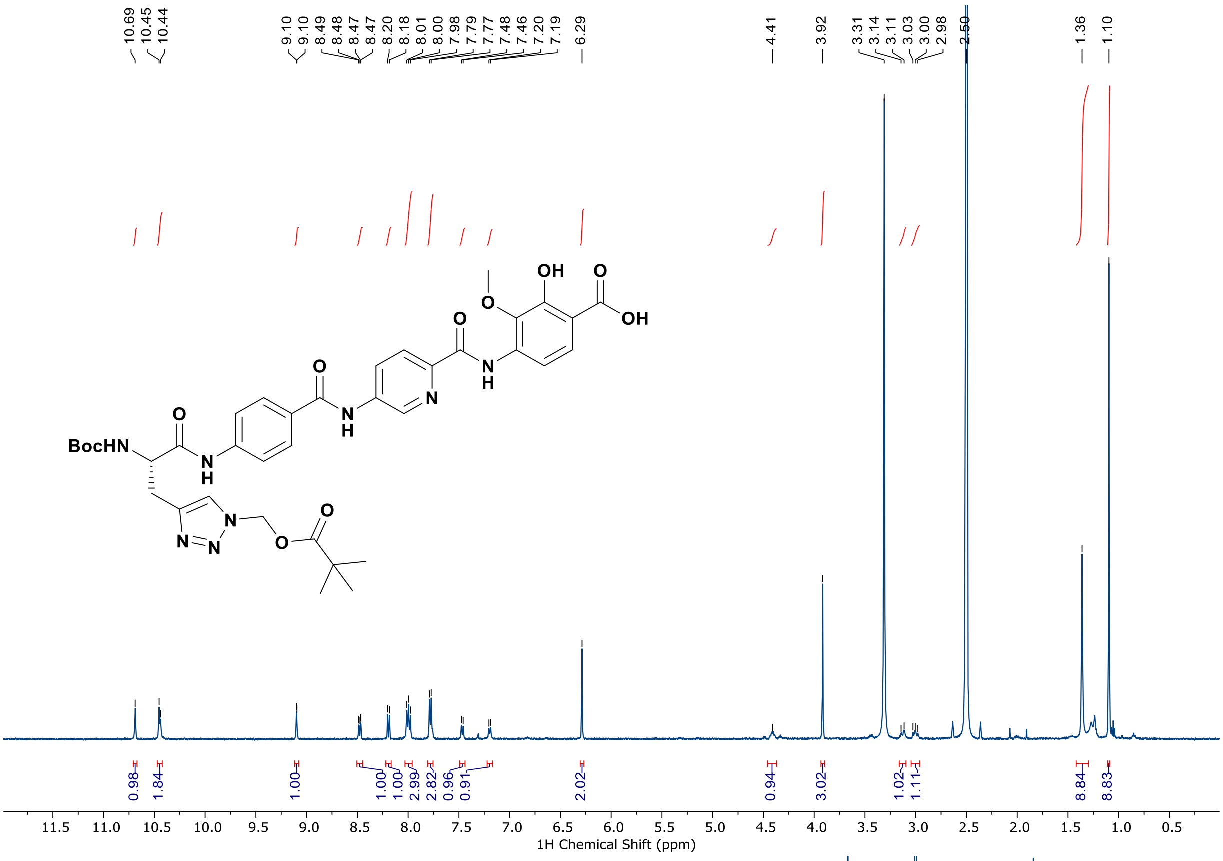

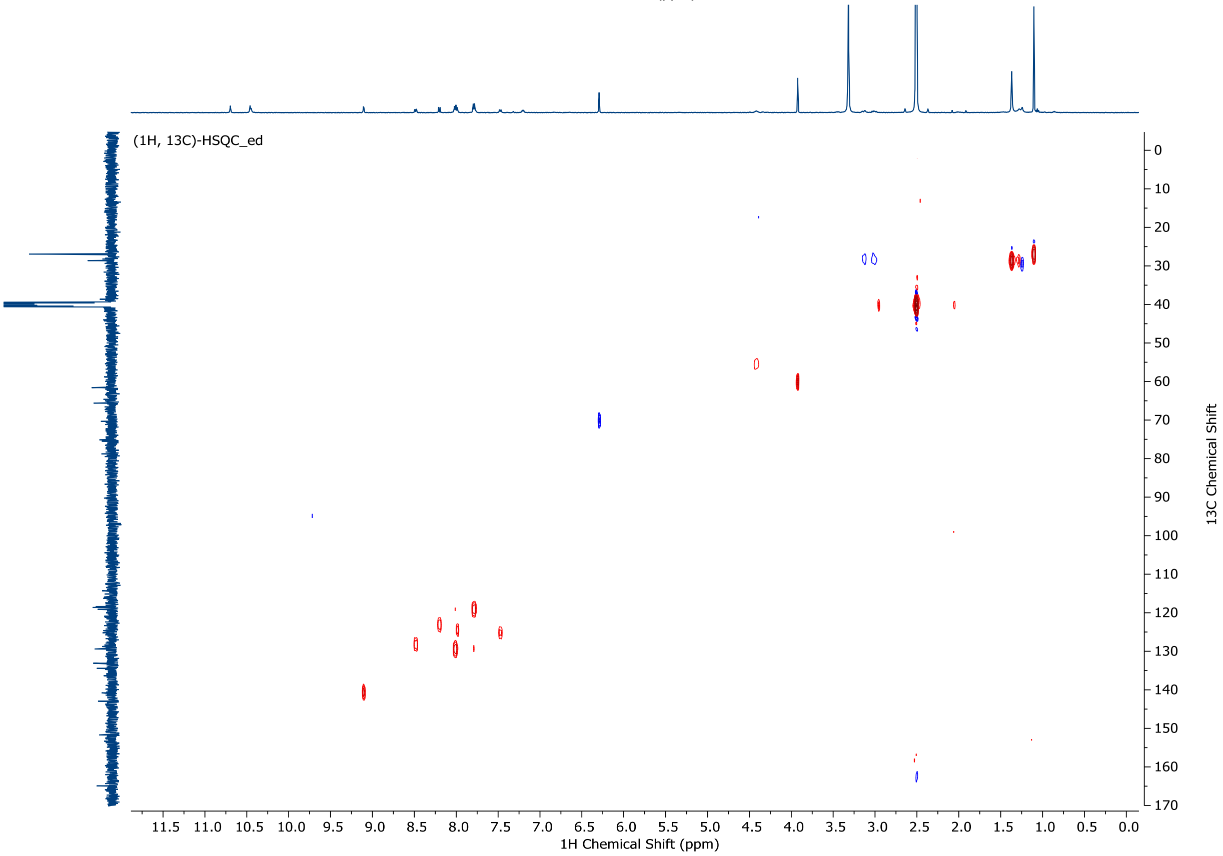

(S,E)-2-hydroxy-4-(5-(4-(2-(4-(3-(4-hydroxyphenyl)-2-methylacrylamido)benzamido)-3-(1H-1,2,3-triazol-4-yl)propanamido)benzamido)picolinamido)-3-methoxybenzoic acid (9)

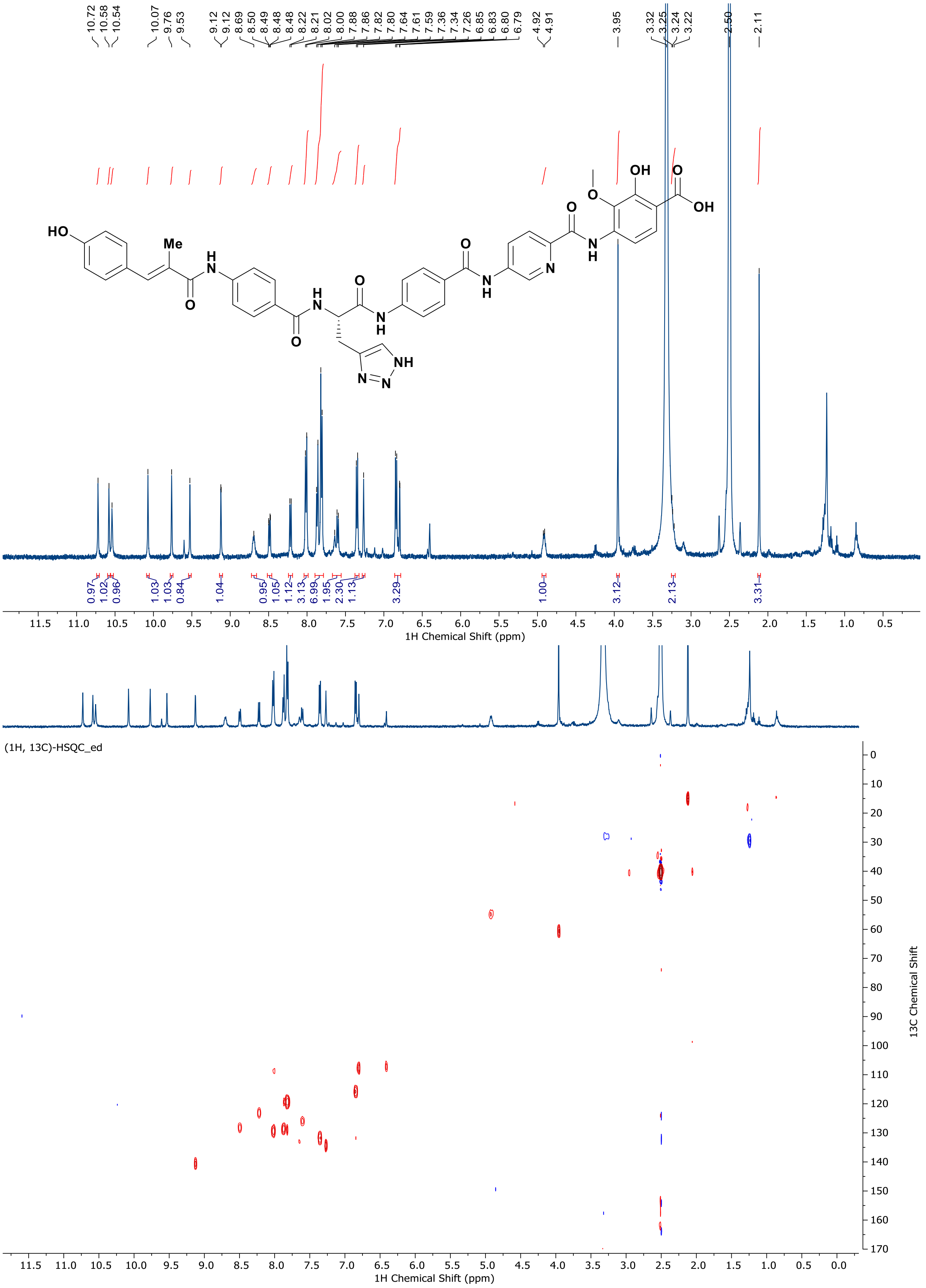

6-((tert-butoxycarbonyl)amino)nicotinic acid (31)

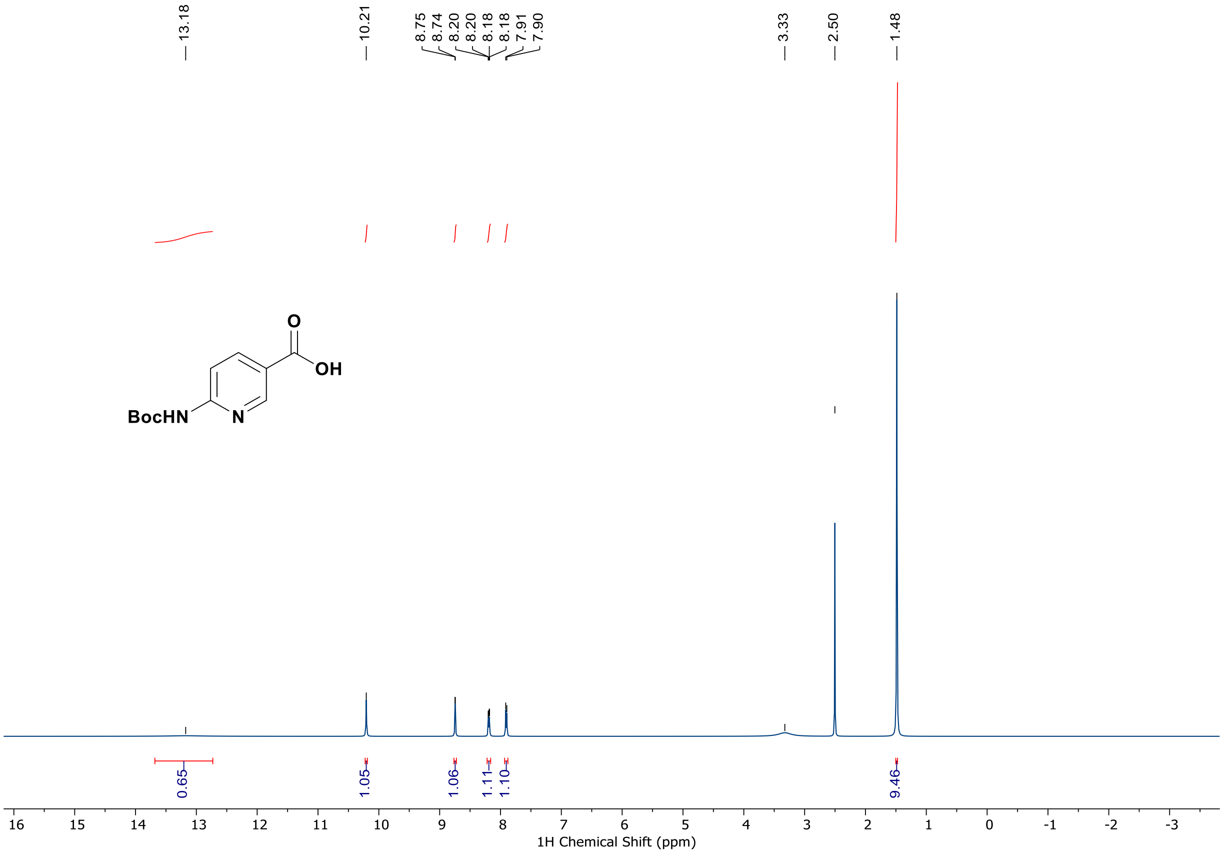

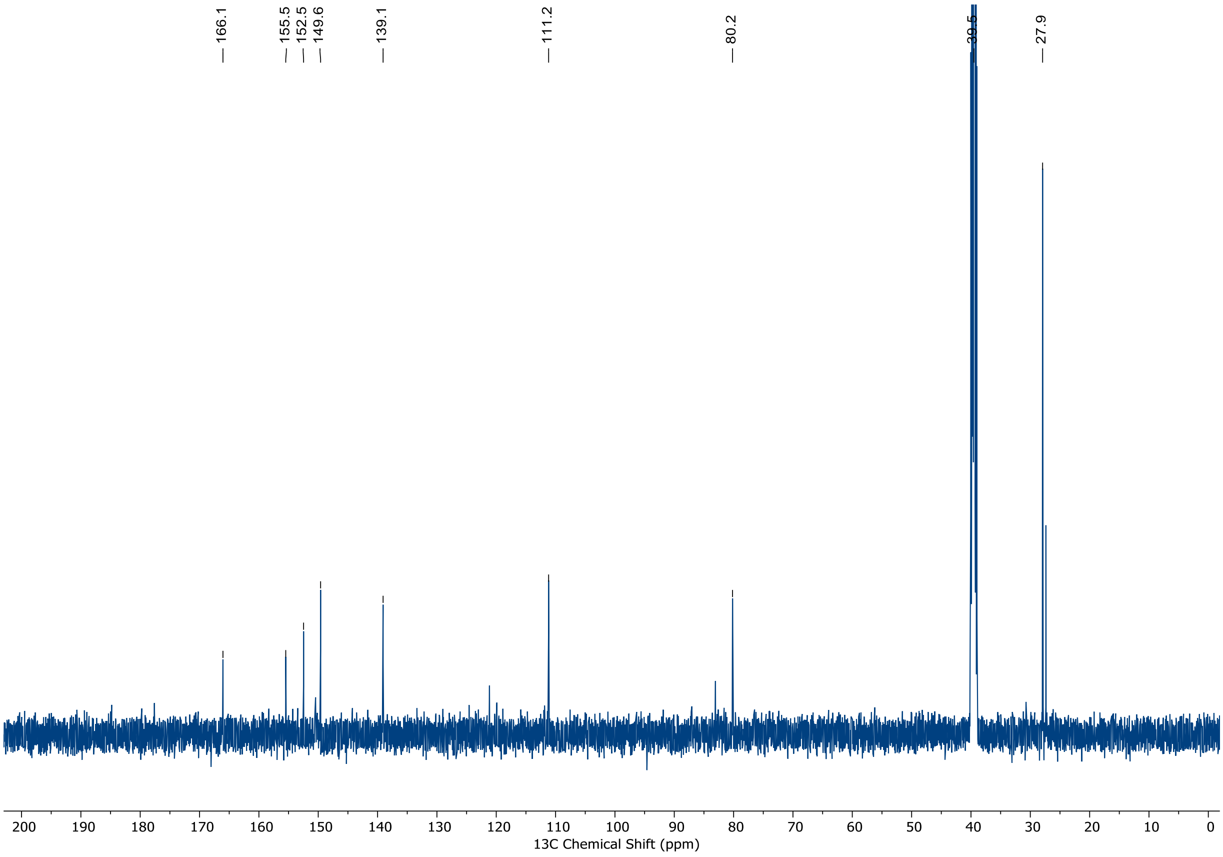

2-hydroxy-3-methoxy-4-nitrobenzoic acid (31-1)[11]

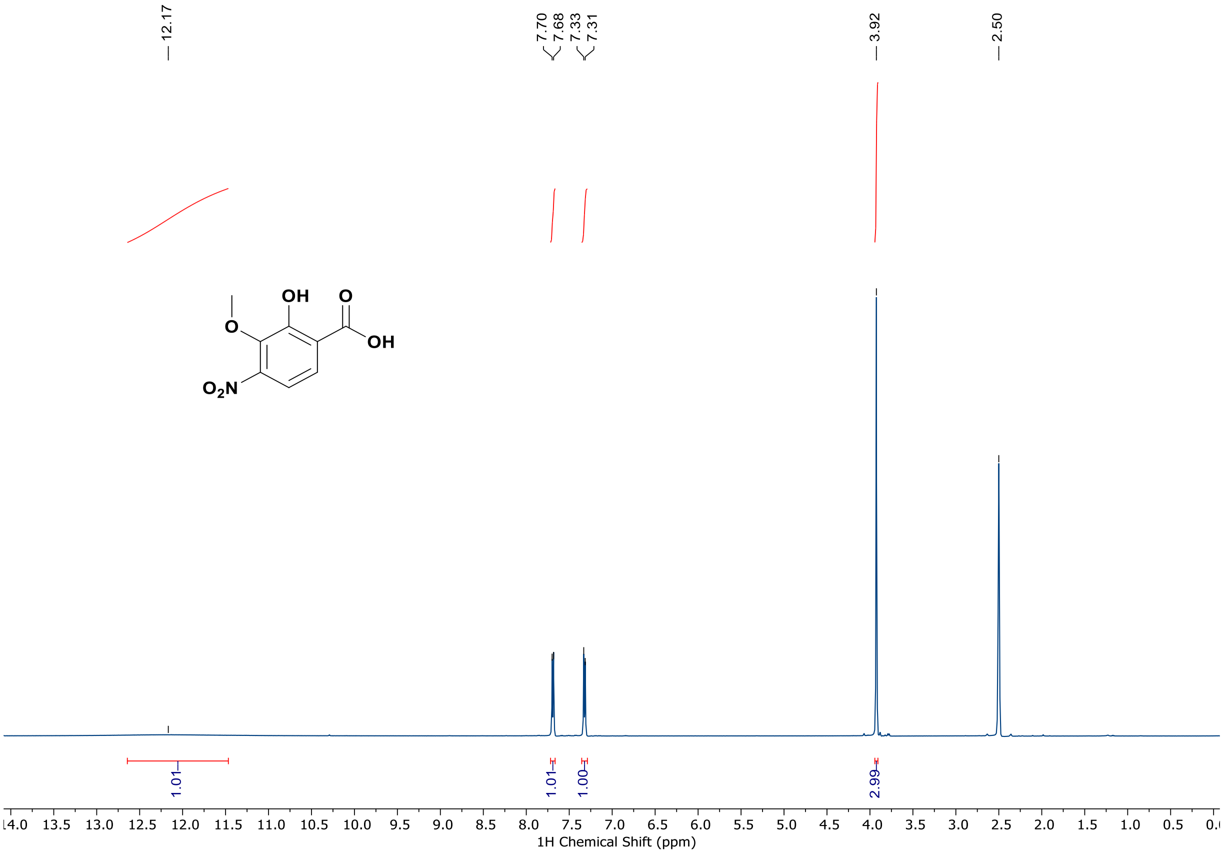

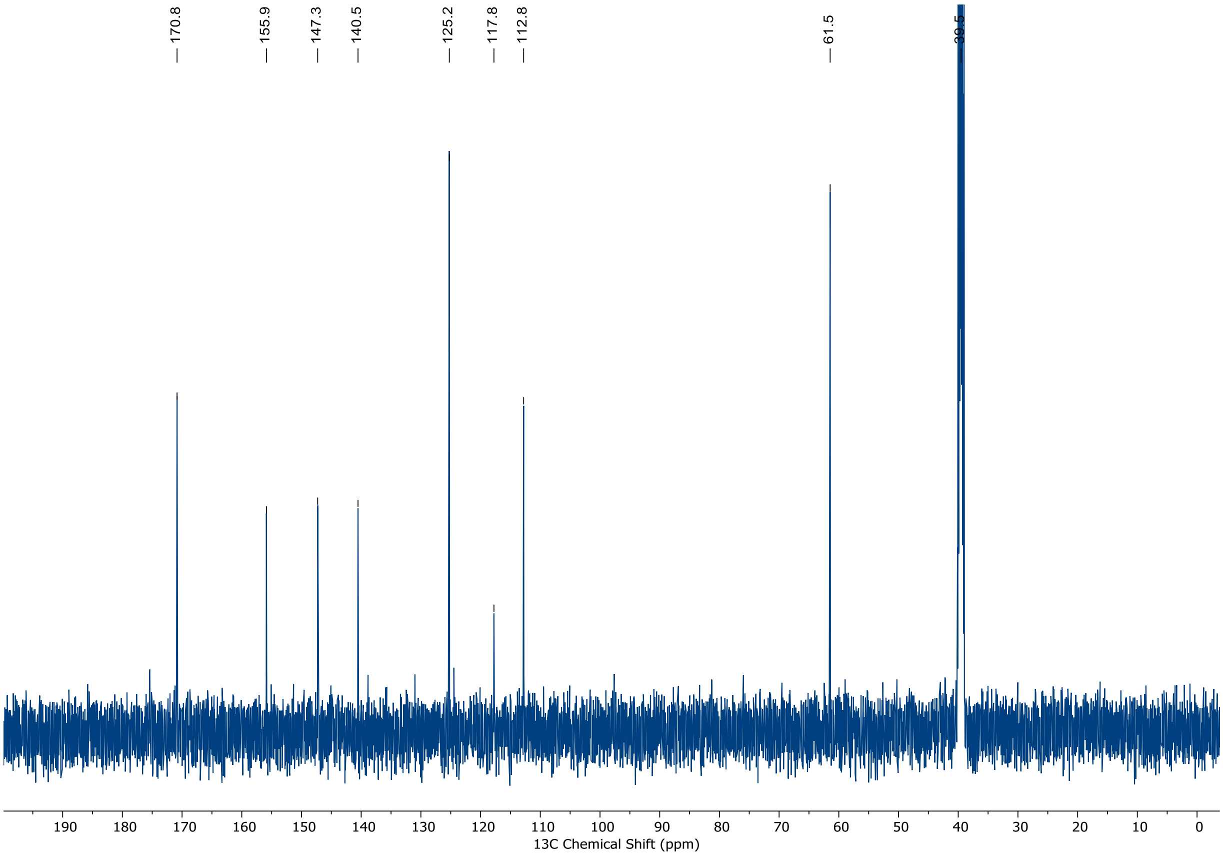

benzyl 4-amino-2-(benzyloxy)-3-methoxybenzoate (31-2)

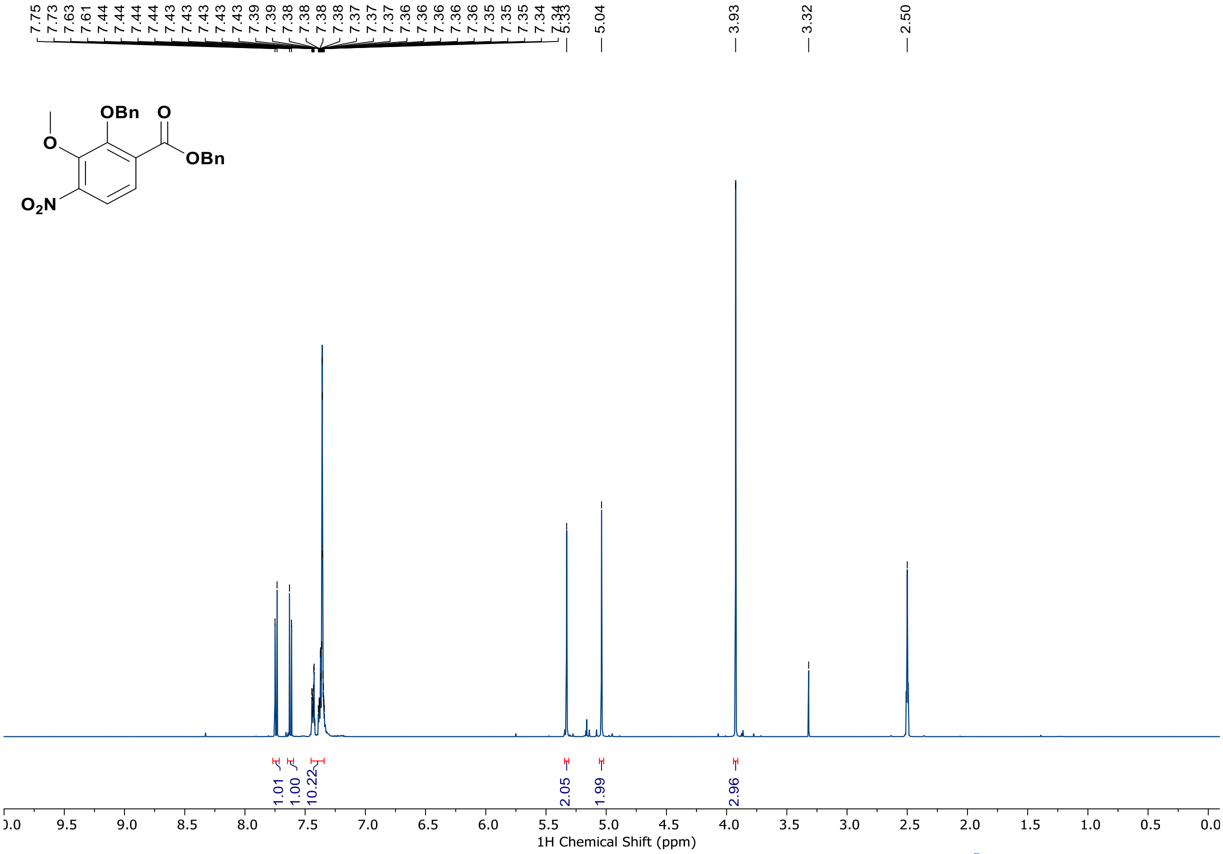

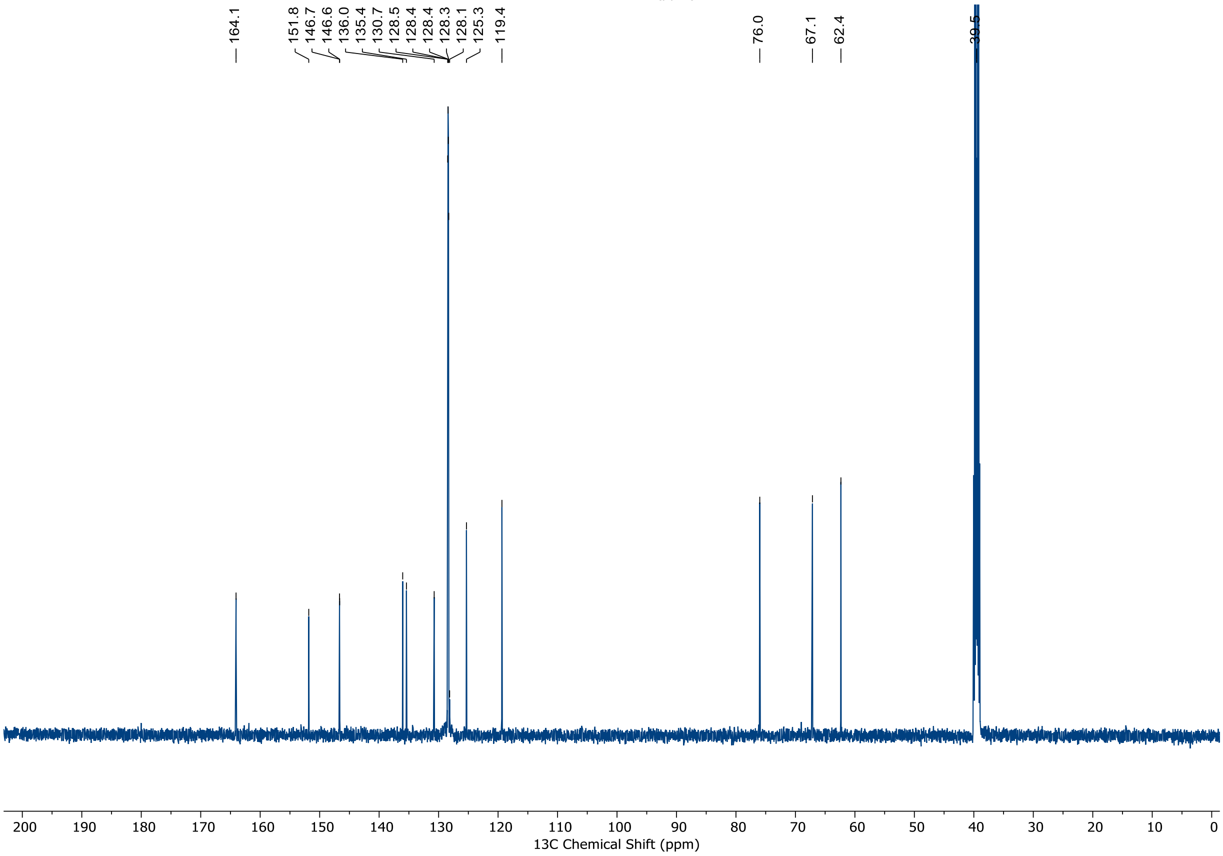

benzyl 4-amino-2-(benzyloxy)-3-methoxybenzoate (28b)

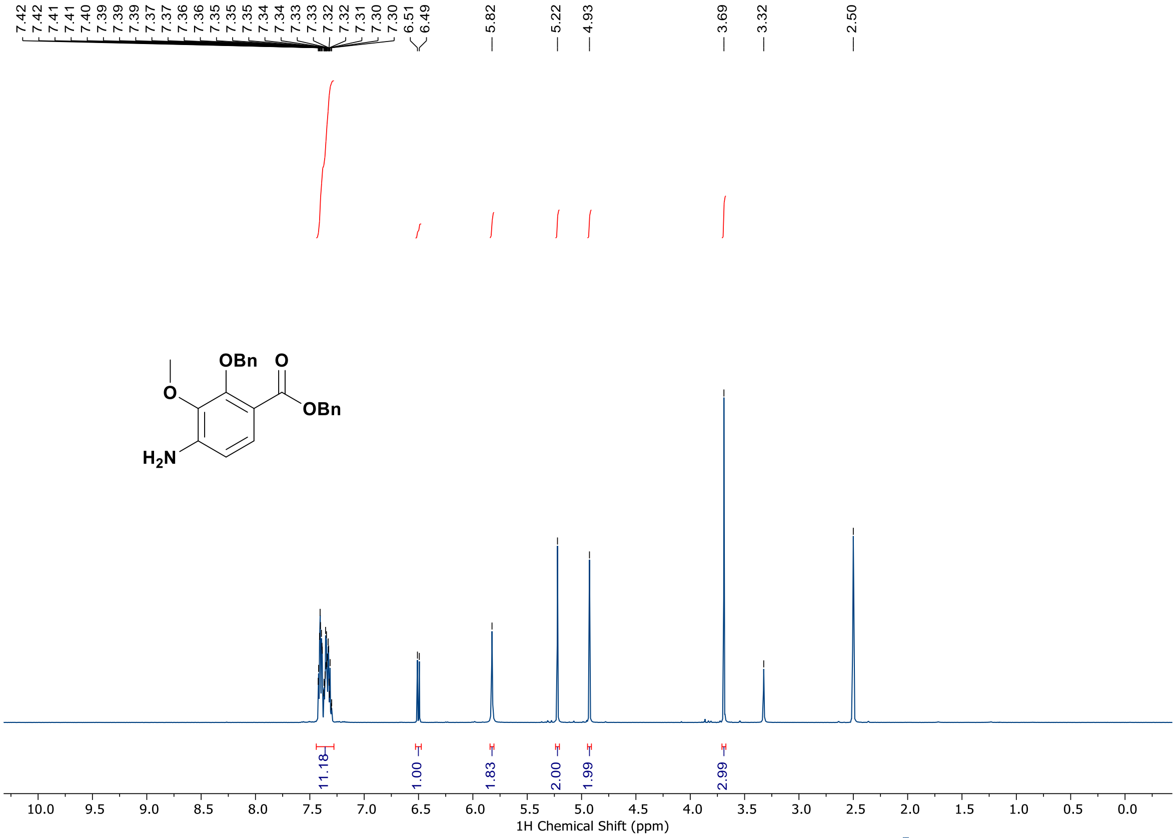

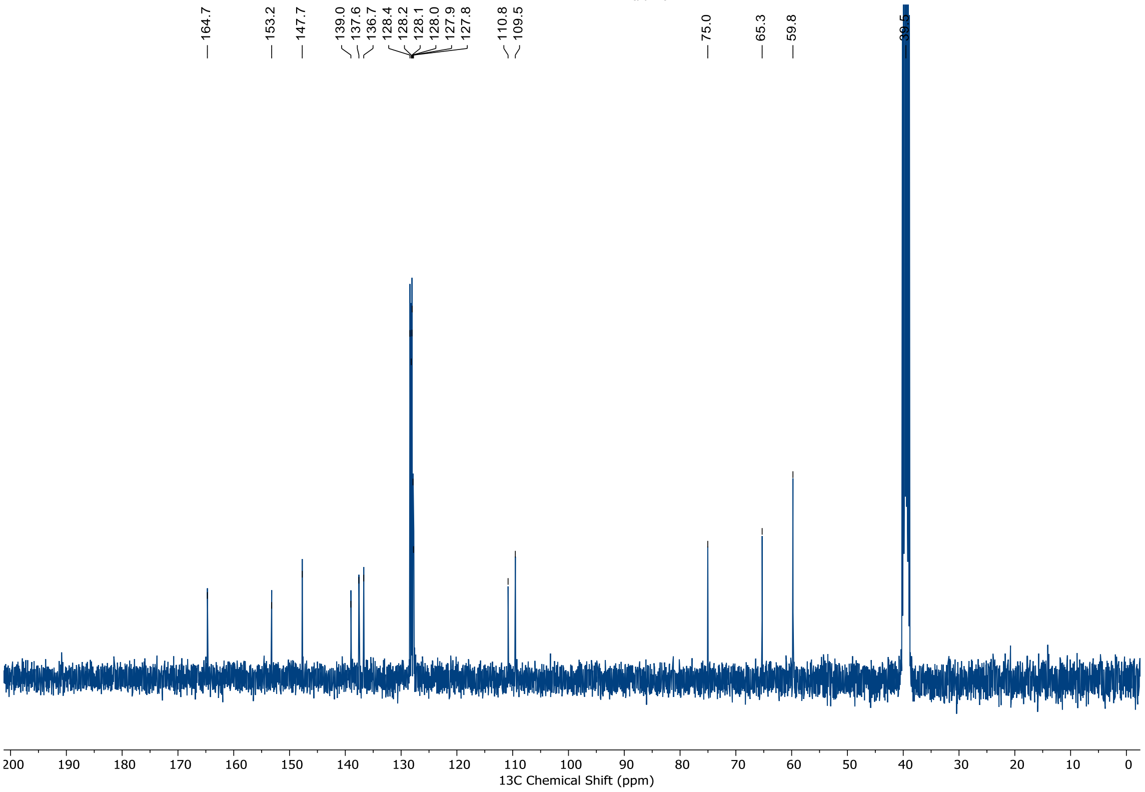

benzyl 2-(benzyloxy)-3-methoxy-4-(6-nitronicotinamido)benzoate (29b)

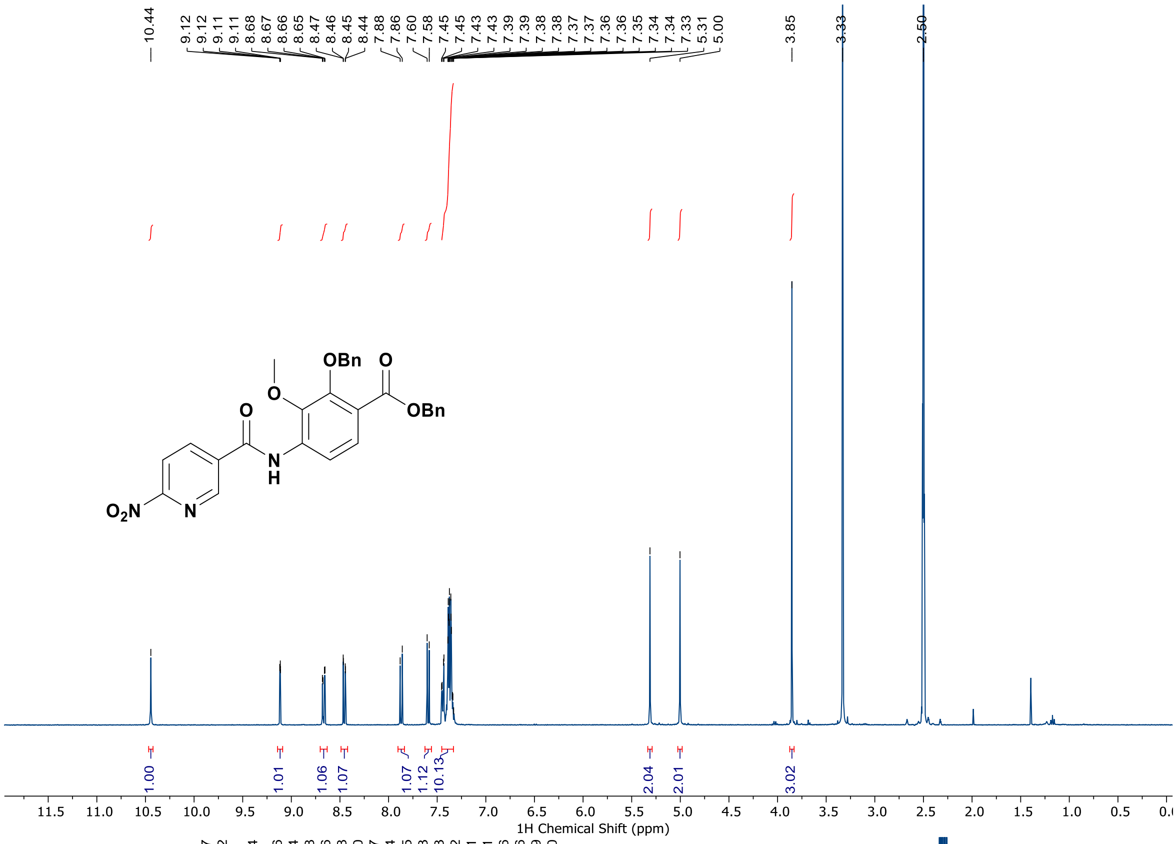

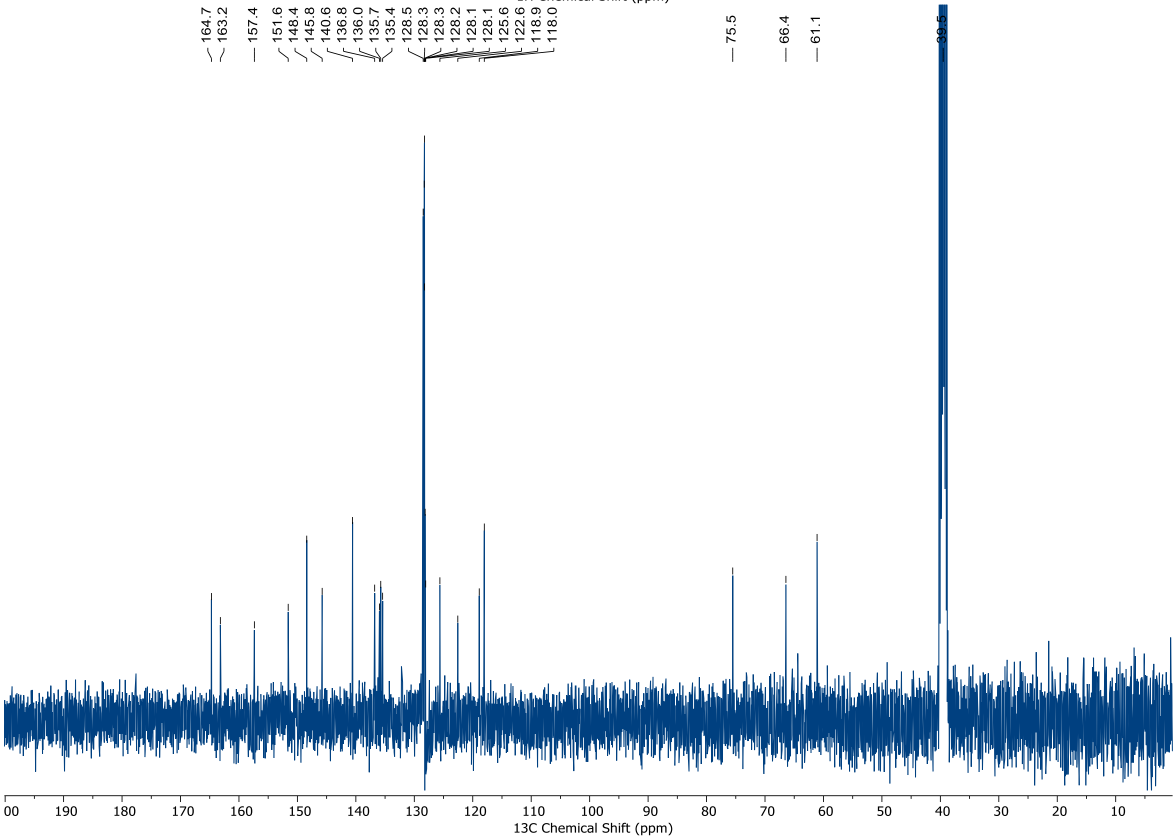

benzyl 2-(benzyloxy)-4-(6-chloronicotinamido)-3-methoxybenzoate (30)

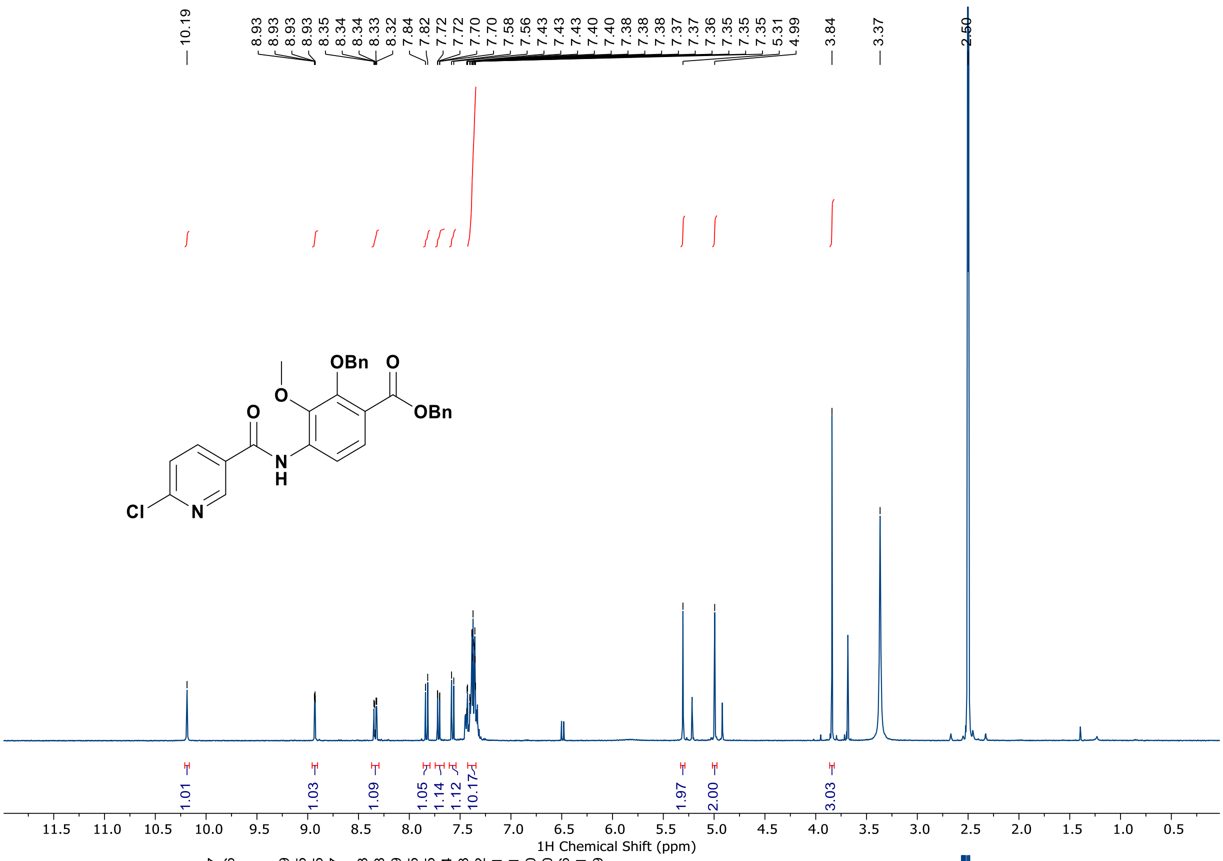

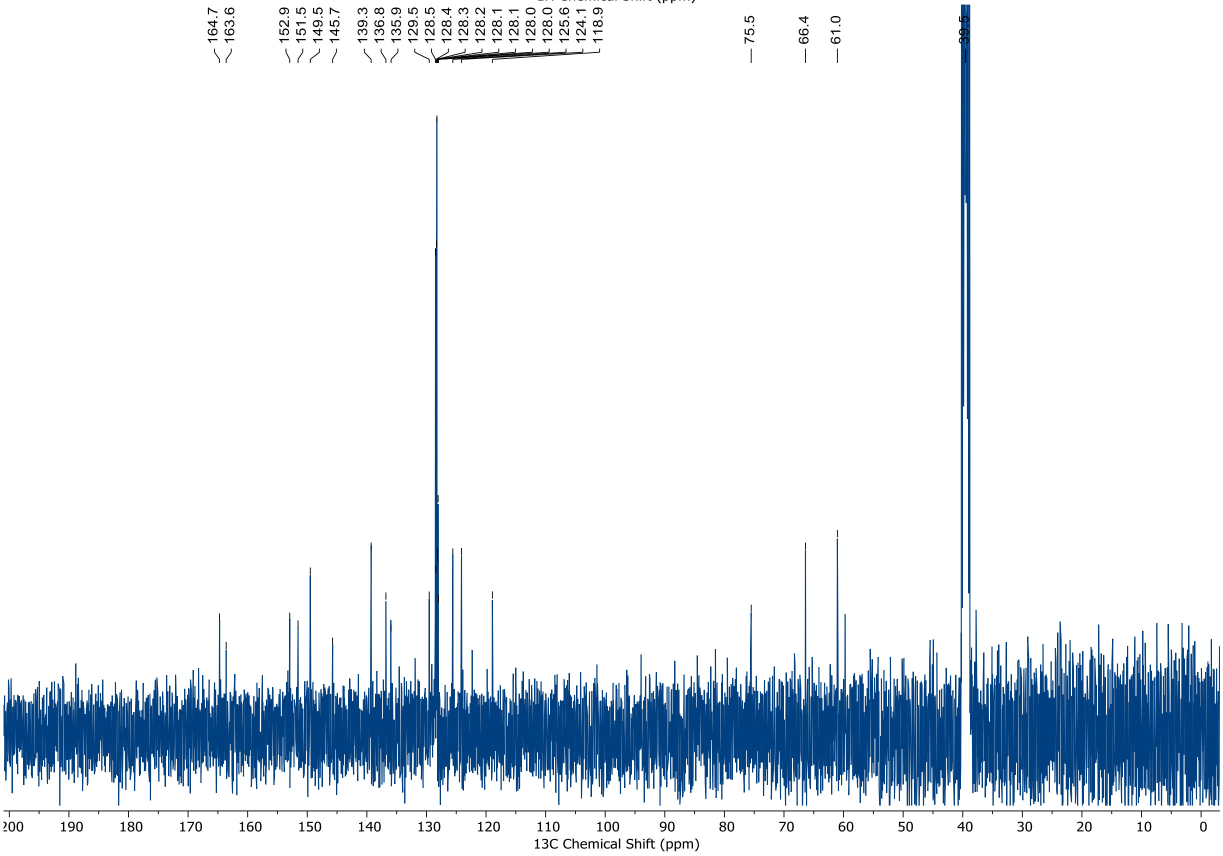

benzyl 2-(benzyloxy)-4-(6-((tert-butoxycarbonyl)amino)nicotinamido)-3-methoxybenzoate (32)

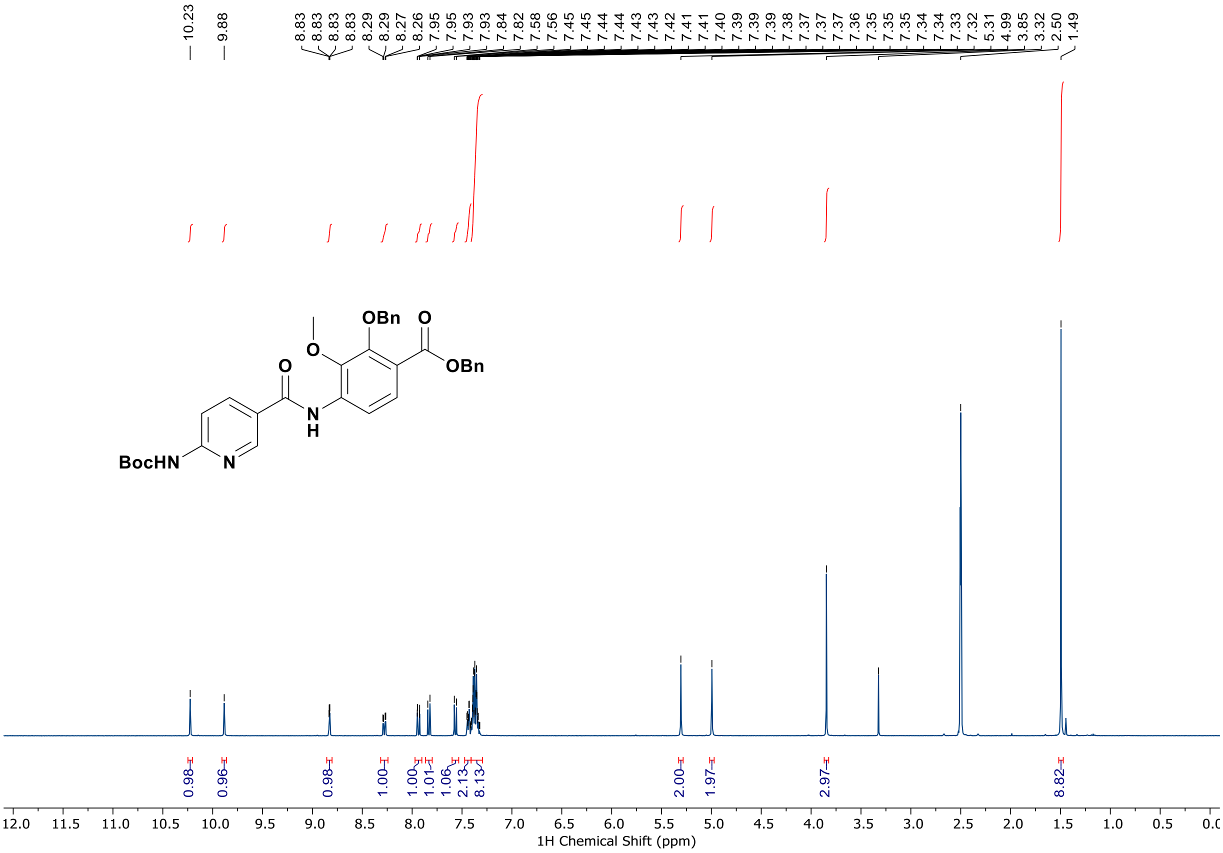

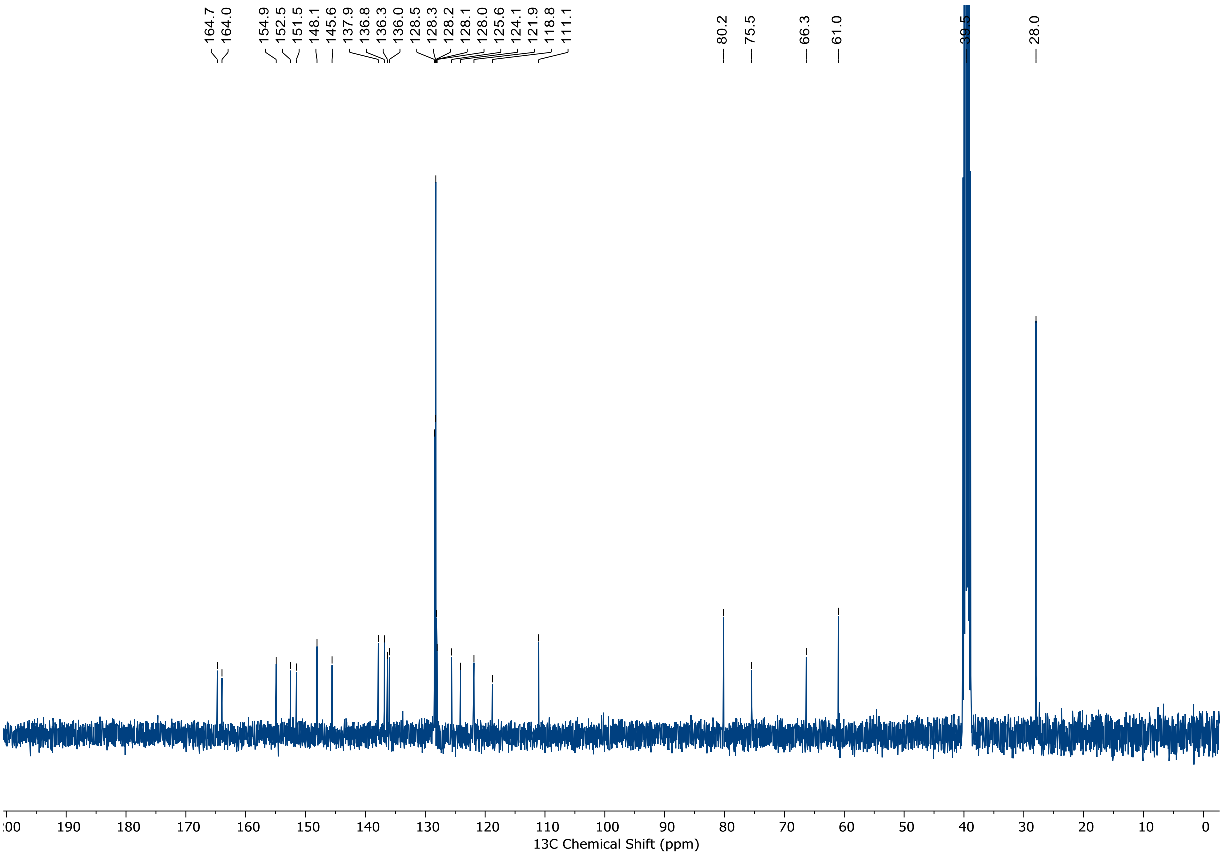

benzyl 4-(6-aminonicotinamido)-2-(benzyloxy)-3-methoxybenzoate (33b)

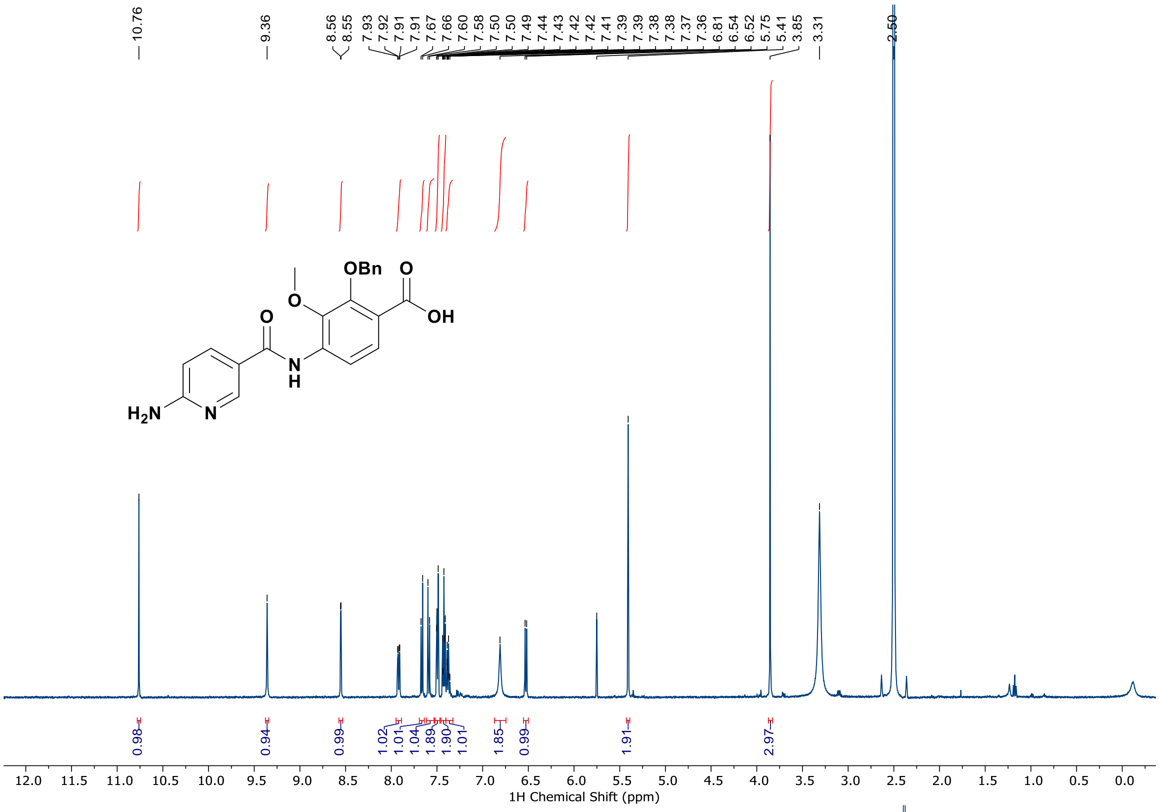

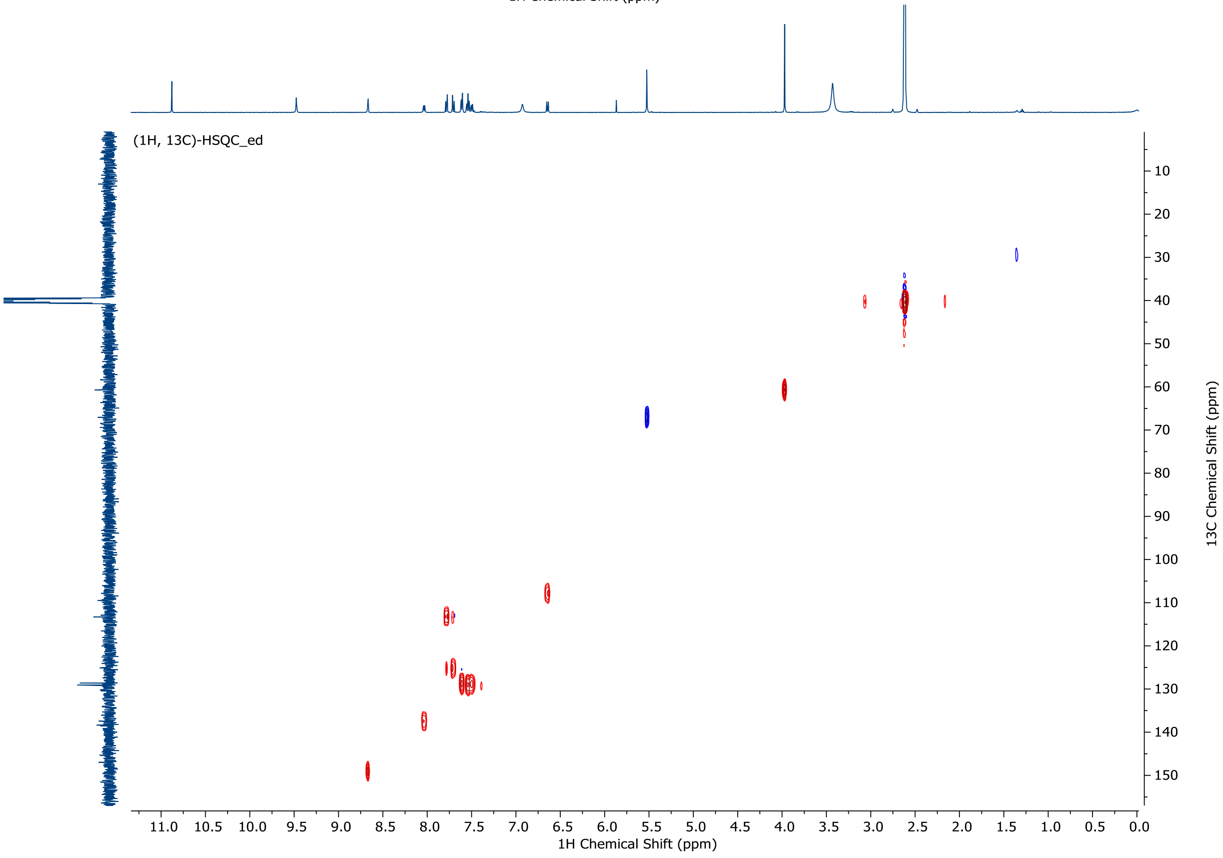

benzyl 4-(6-aminonicotinamido)-2-(benzyloxy)-3-methoxybenzoate (33b)

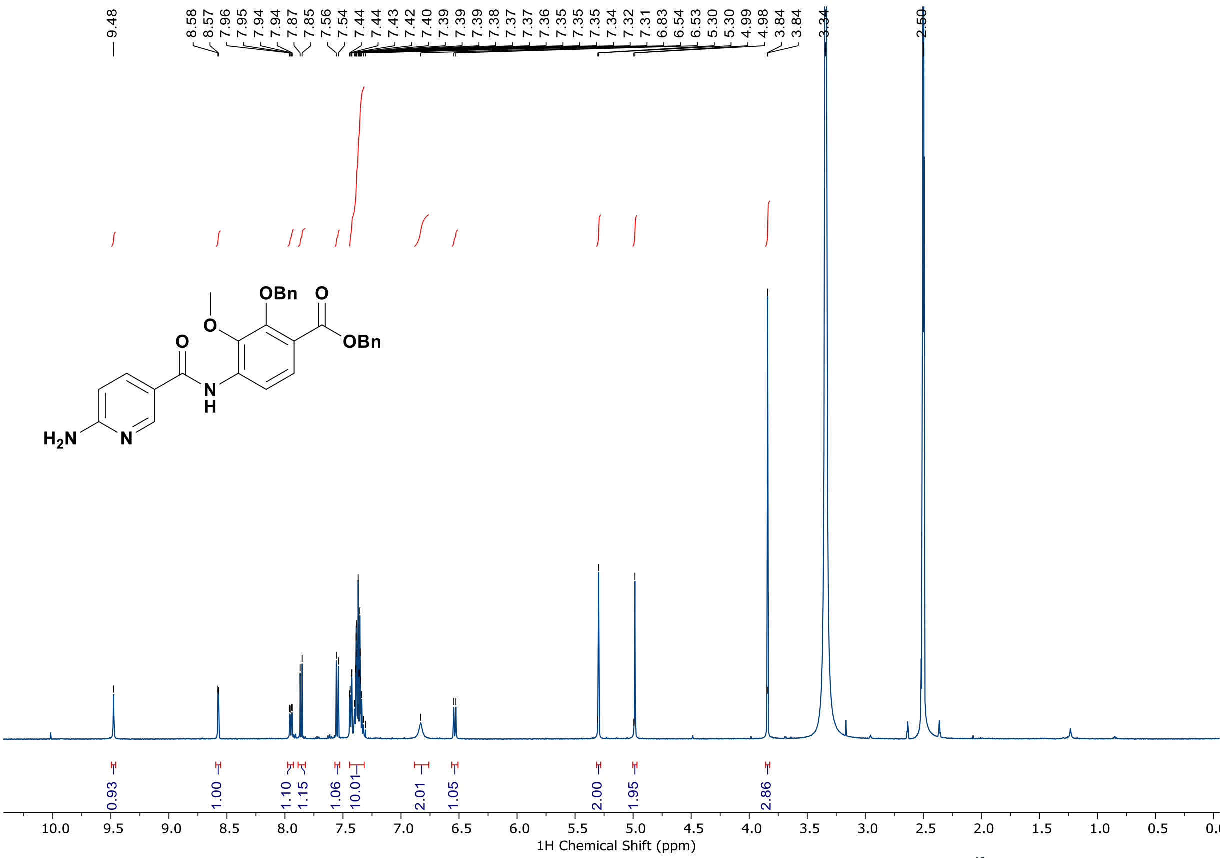

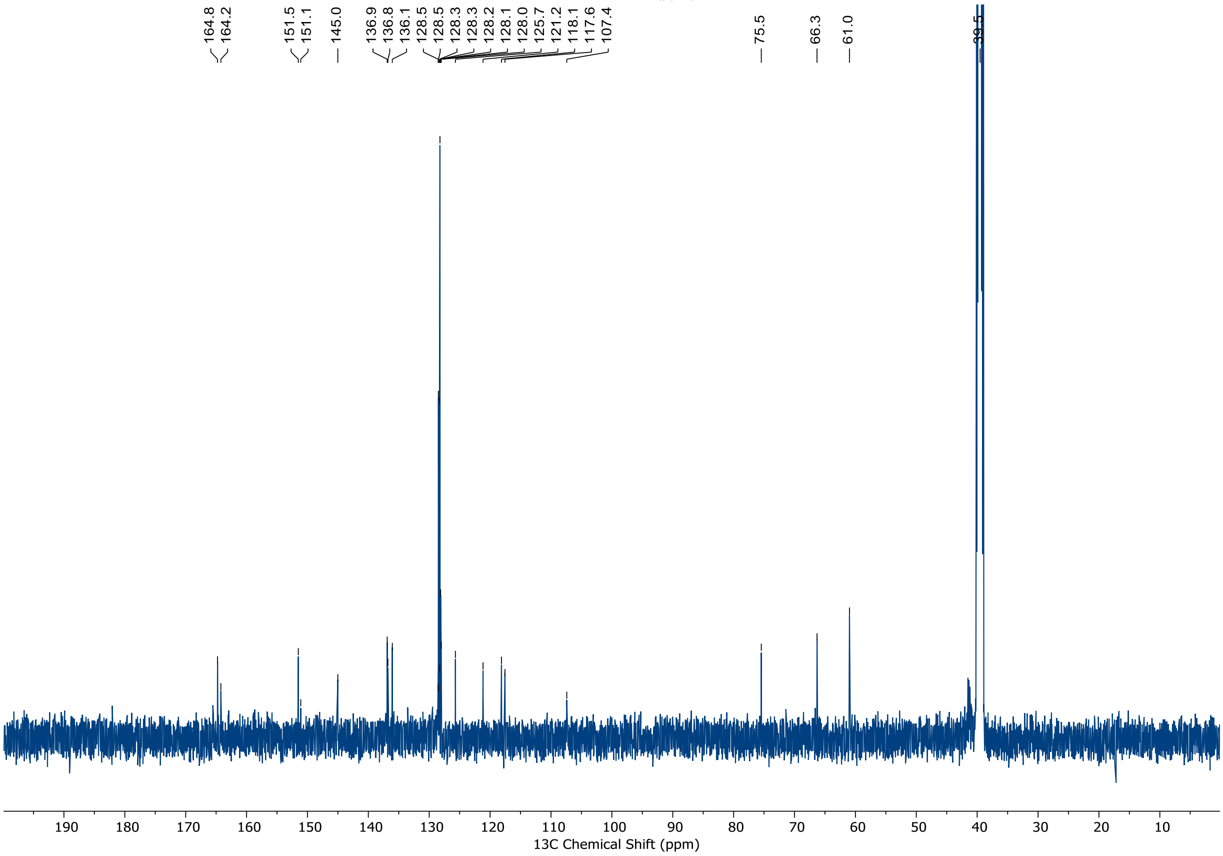

benzyl 2-(benzyloxy)-3-methoxy-4-(6-(4-nitrobenzamido)nicotinamido)benzoate (33b-1)

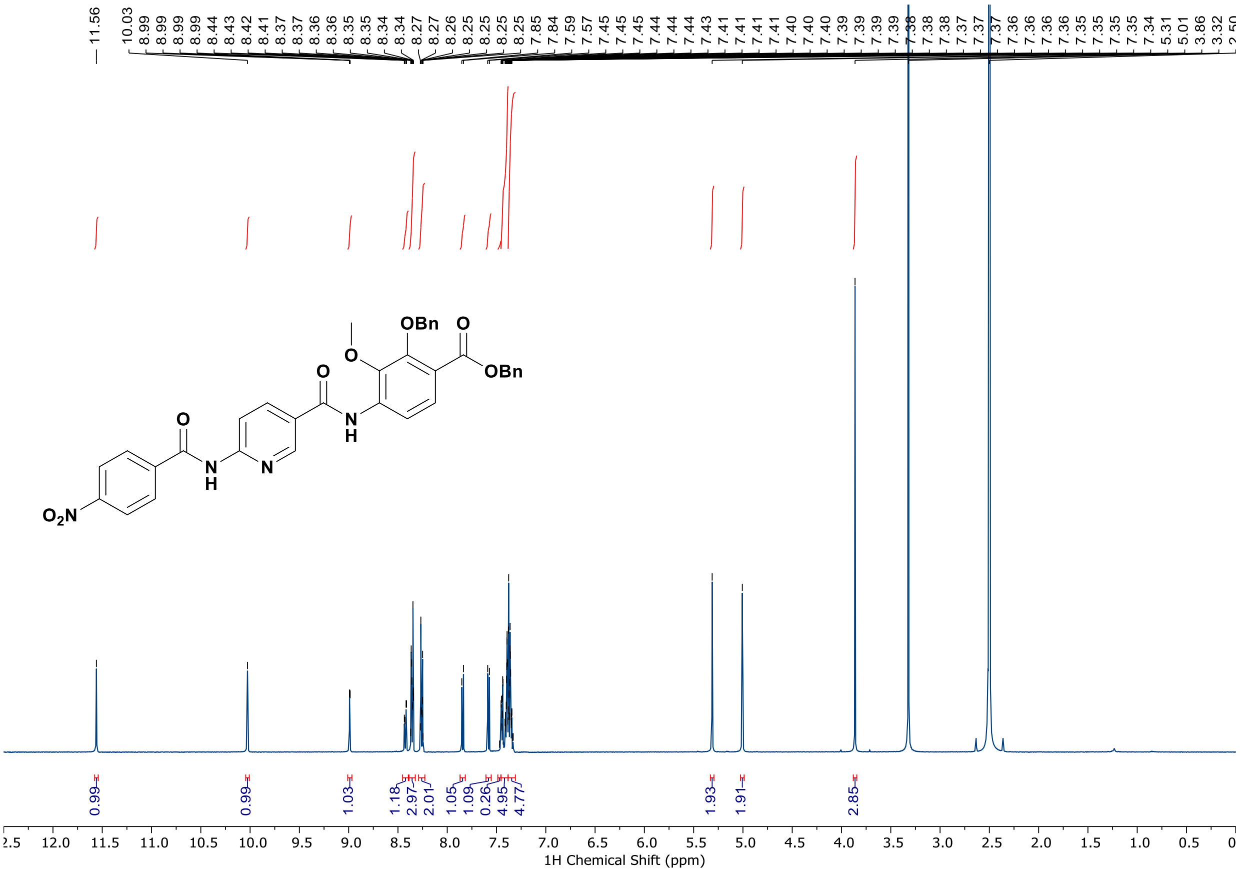

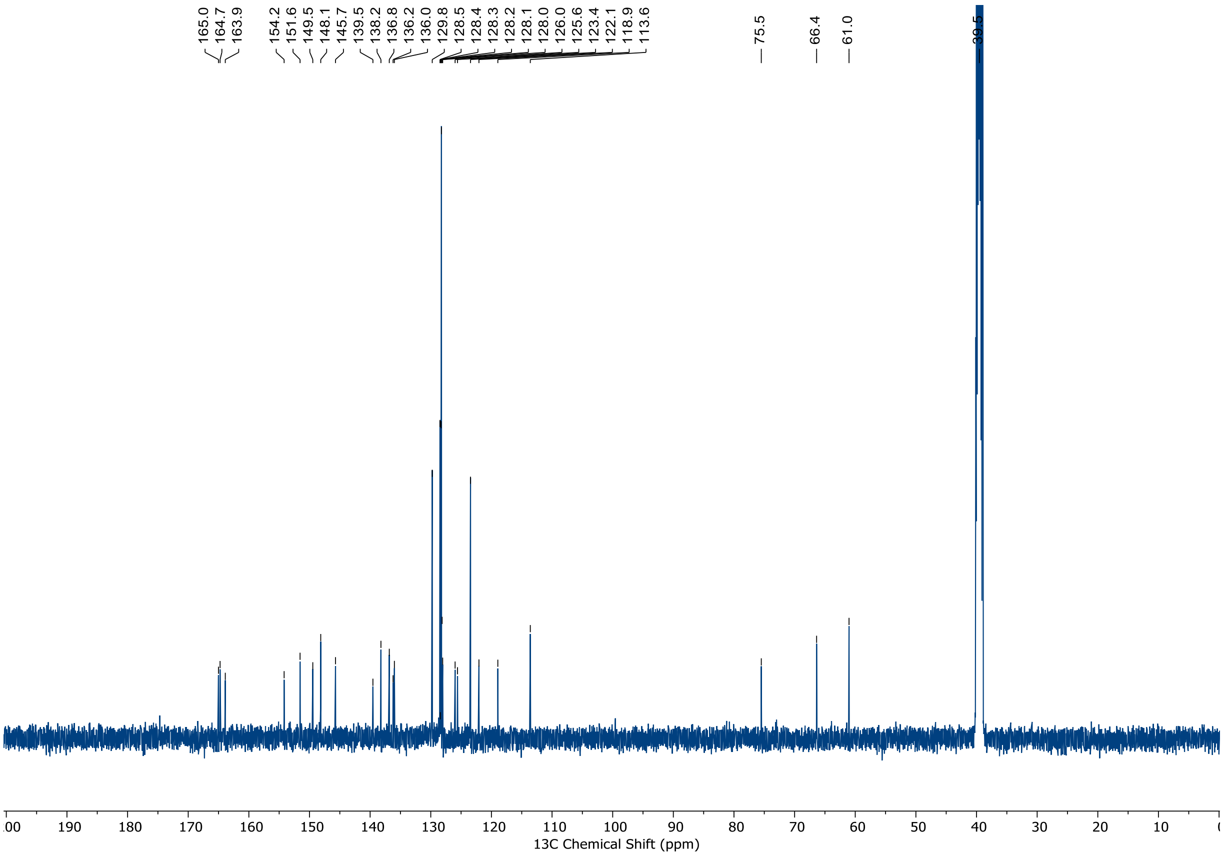

benzyl 2-(benzyloxy)-3-methoxy-4-(6-(4-nitro-N-(4-nitrobenzoyl)benzamido)nicotinamido)benzoate (33b-2)

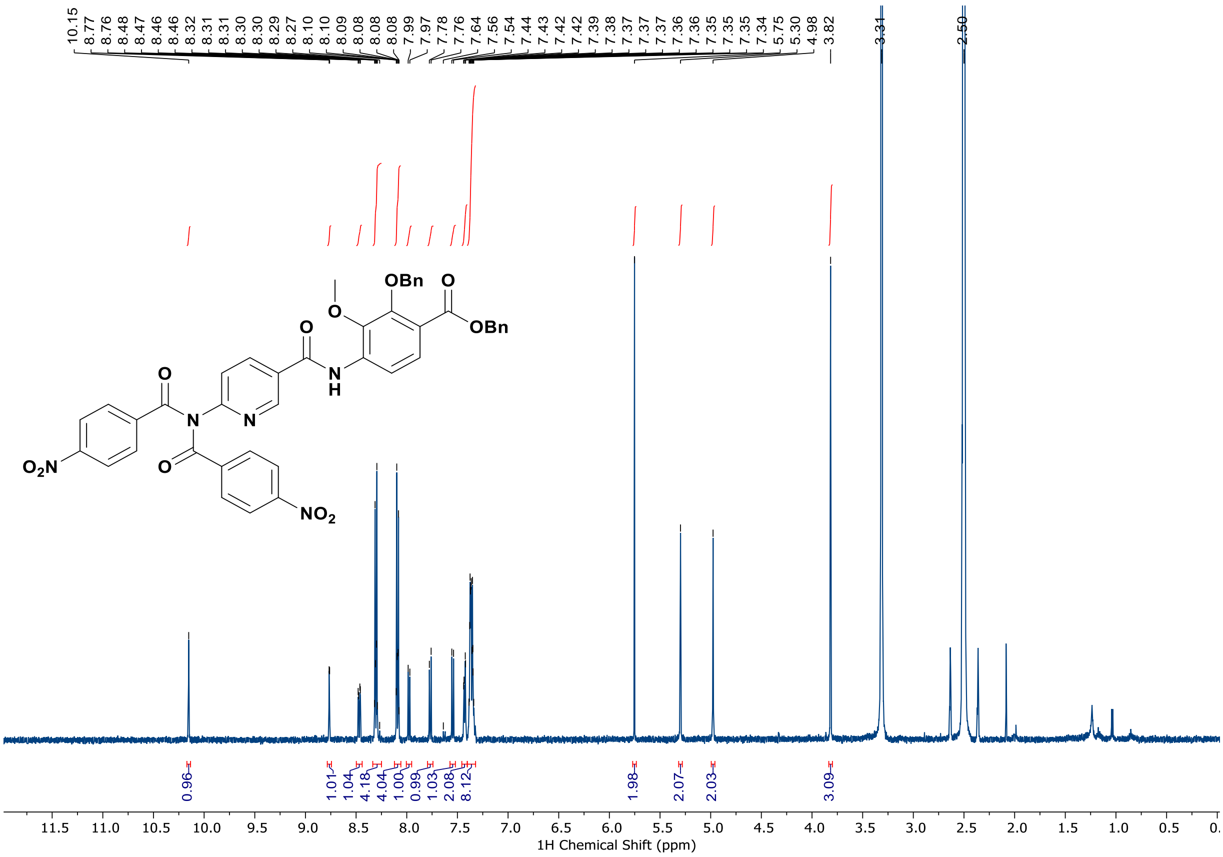

benzyl 4-(6-(4-aminobenzamido)nicotinamido)-2-(benzyloxy)-3-methoxybenzoate (36d)

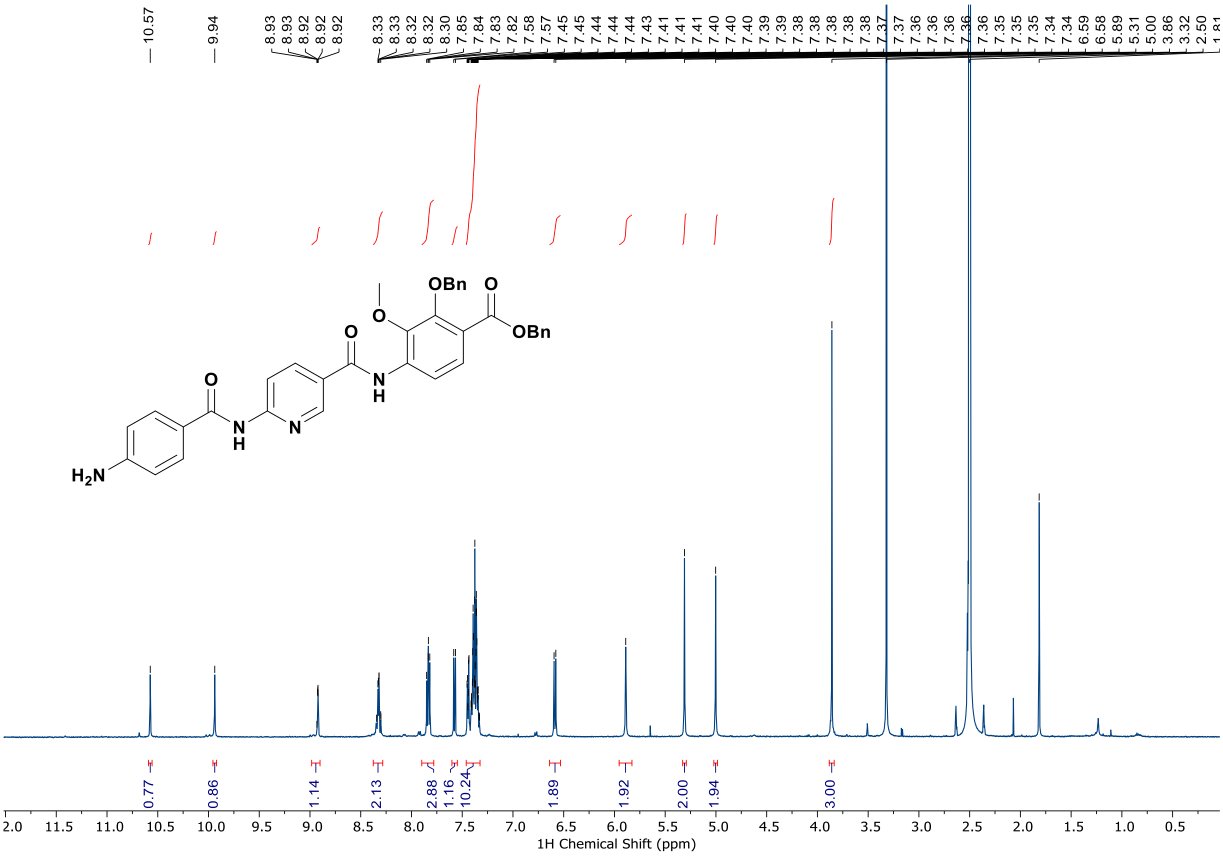

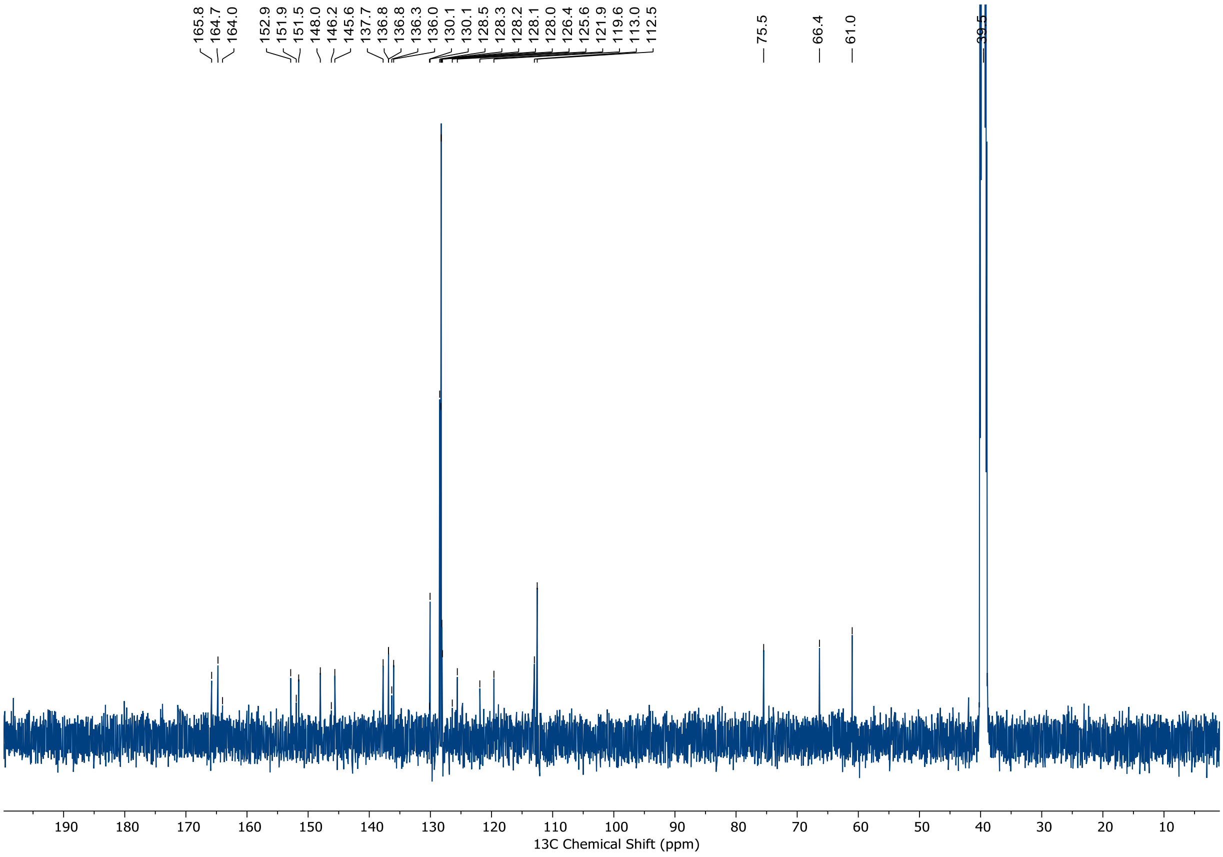

benzyl (S)-2-(benzyloxy)-4-(6-(4-(2-((tert-butoxycarbonyl)amino)-3-(1-((pivaloyloxy)methyl)-1H-1,2,3-triazol-4-yl)propanamido)benzamido)nicotinamido)-3-methoxybenzoate (36d-1)

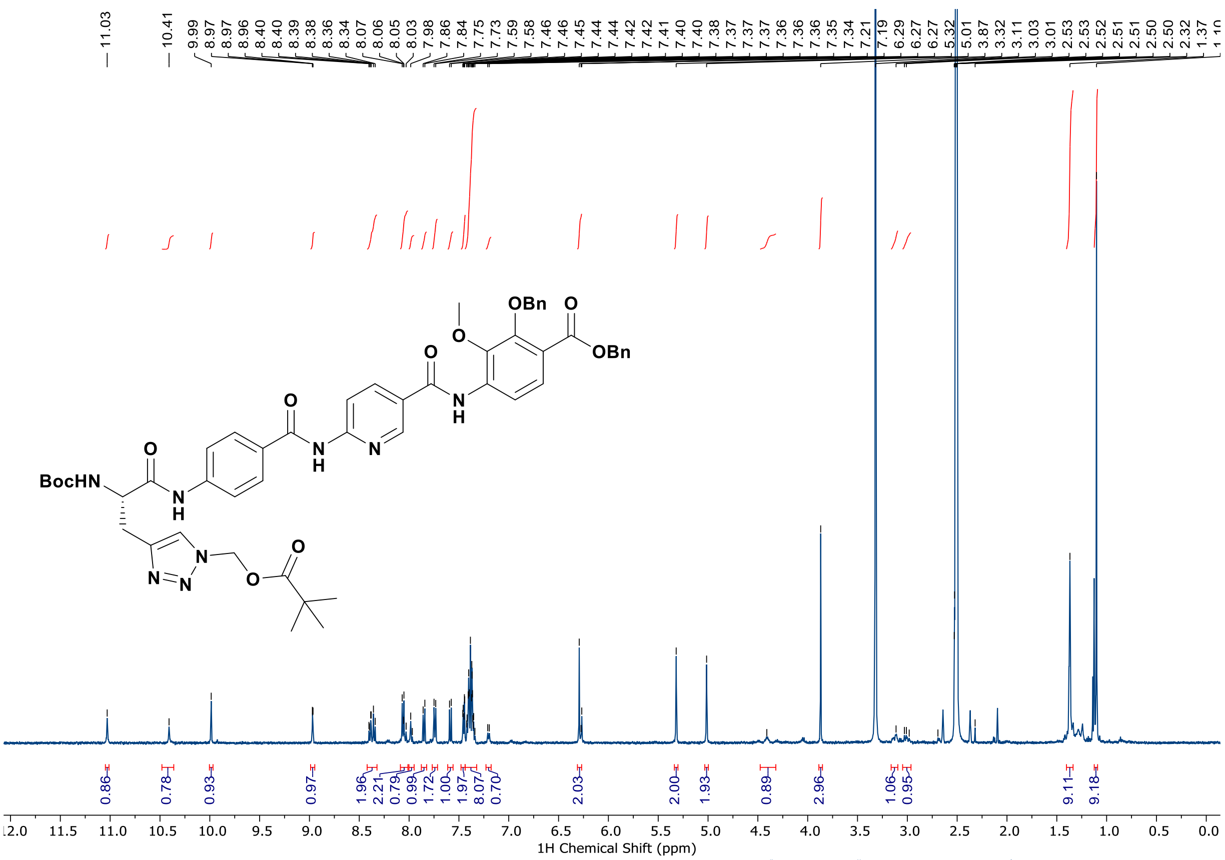

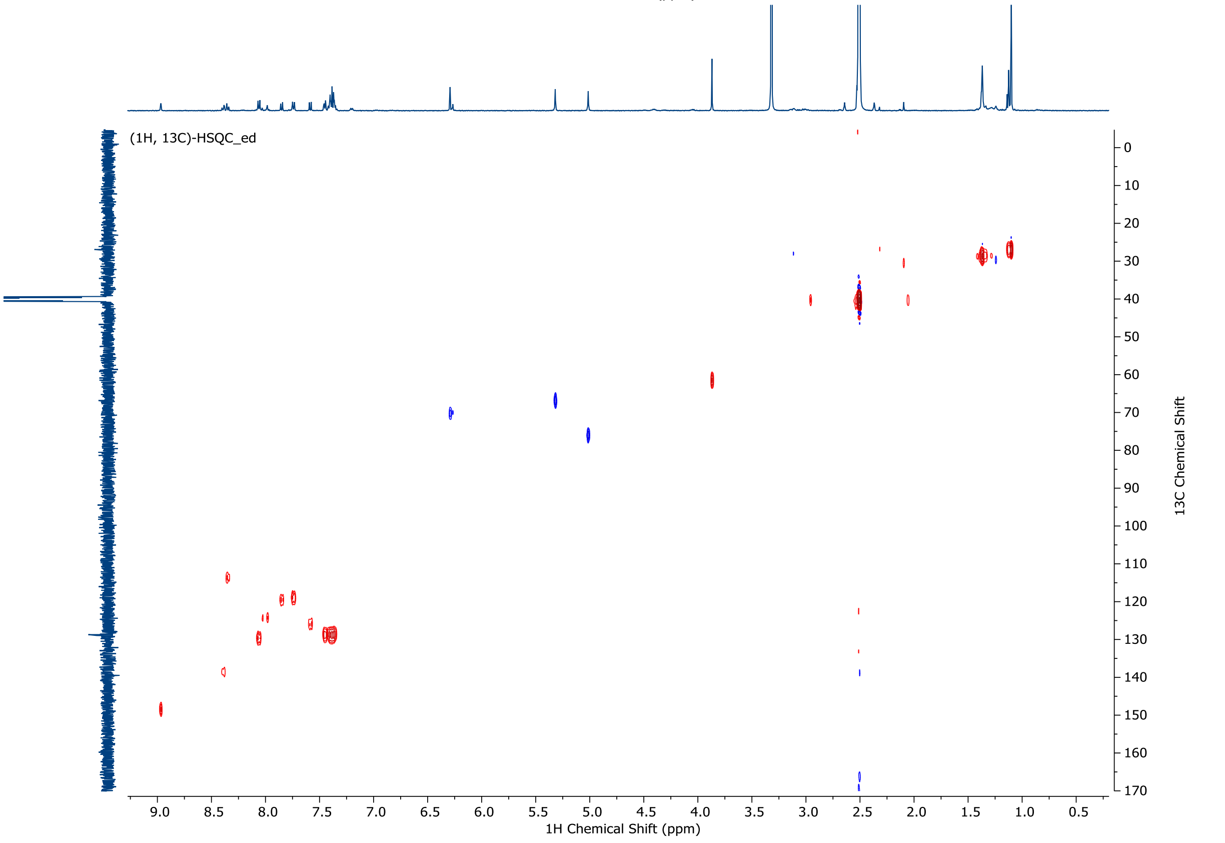

(S)-4-(6-(4-(2-amino-3-(1-((pivaloyloxy)methyl)-1H-1,2,3-triazol-4-yl)propanamido)benzamido)nicotinamido)-2-hydroxy-3-methoxybenzoic acid (37d)

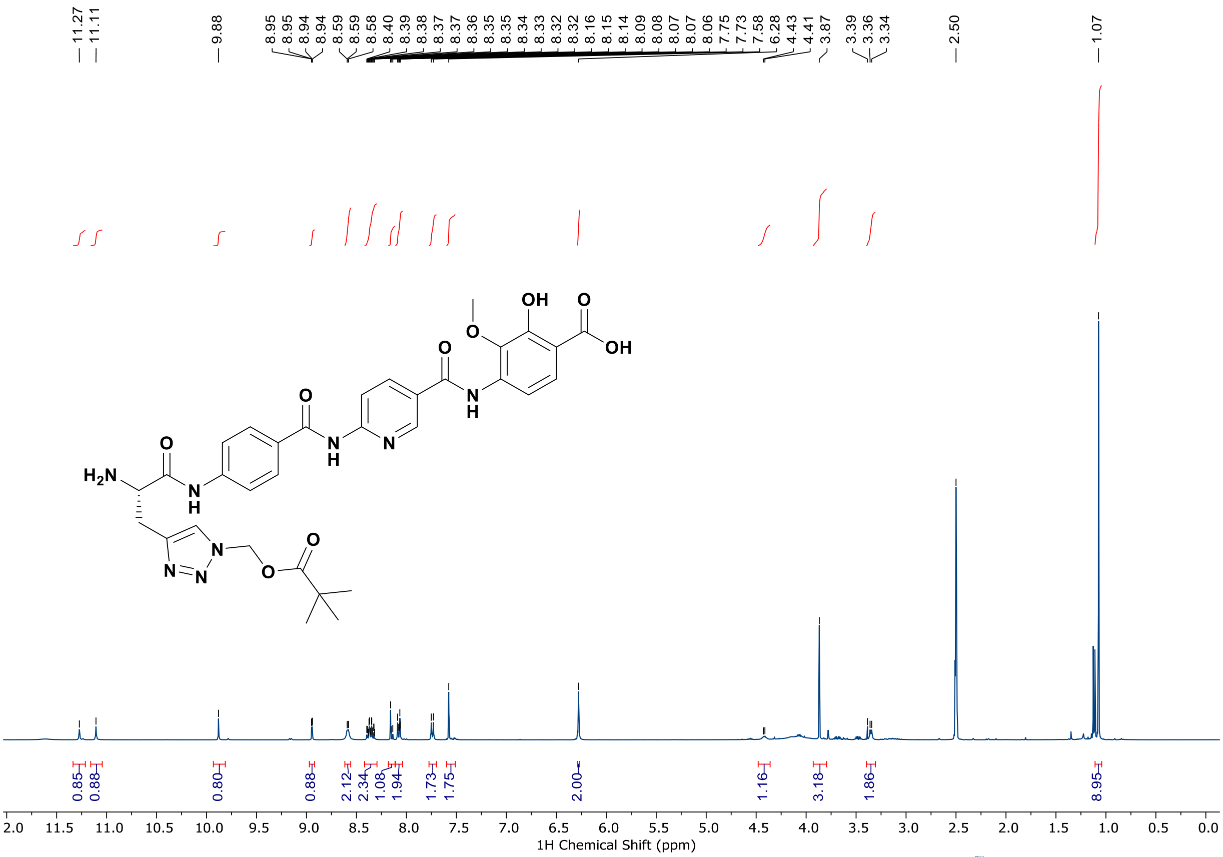

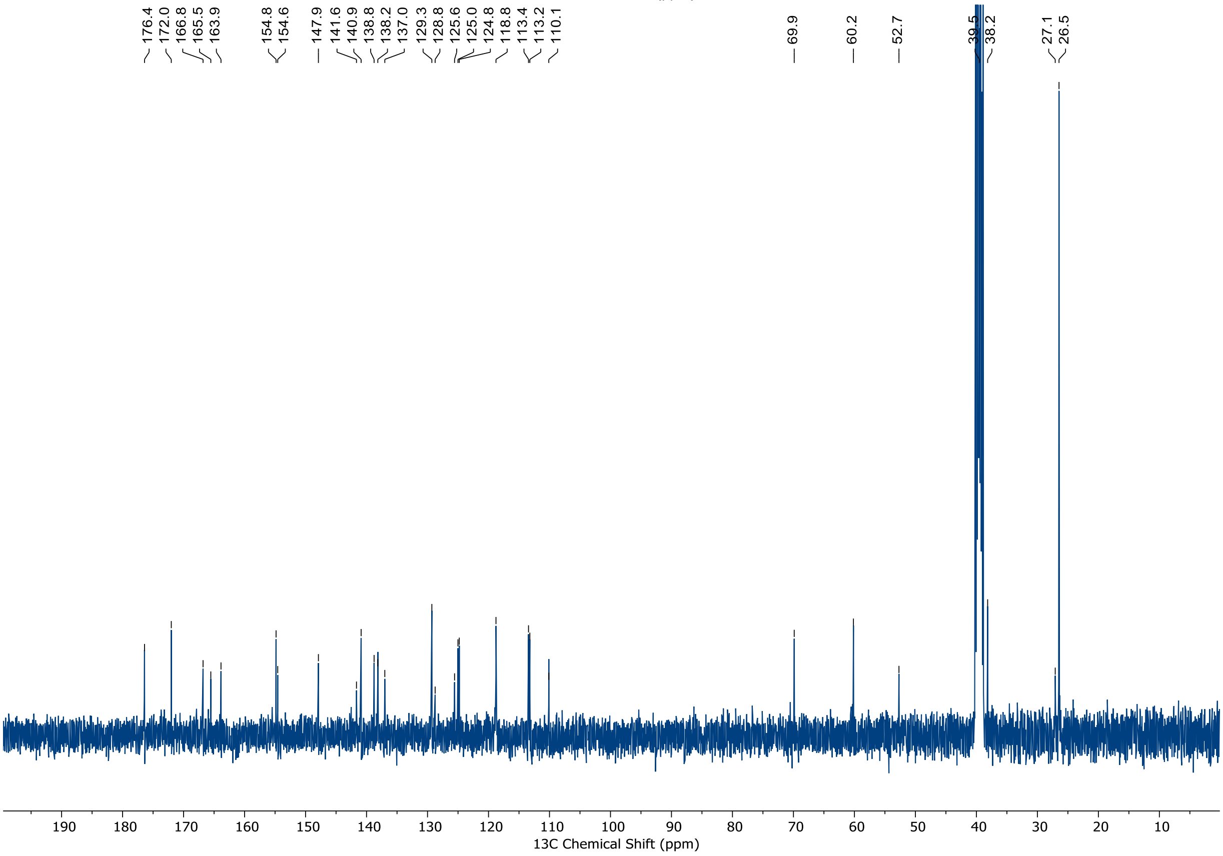

(S,E)-2-hydroxy-4-(6-(4-(2-(4-(3-(4-hydroxyphenyl)-2-methylacrylamido)benzamido)-3-(1H-1,2,3-triazol-4-yl)propanamido)benzamido)nicotinamido)-3-methoxybenzoic acid (10)

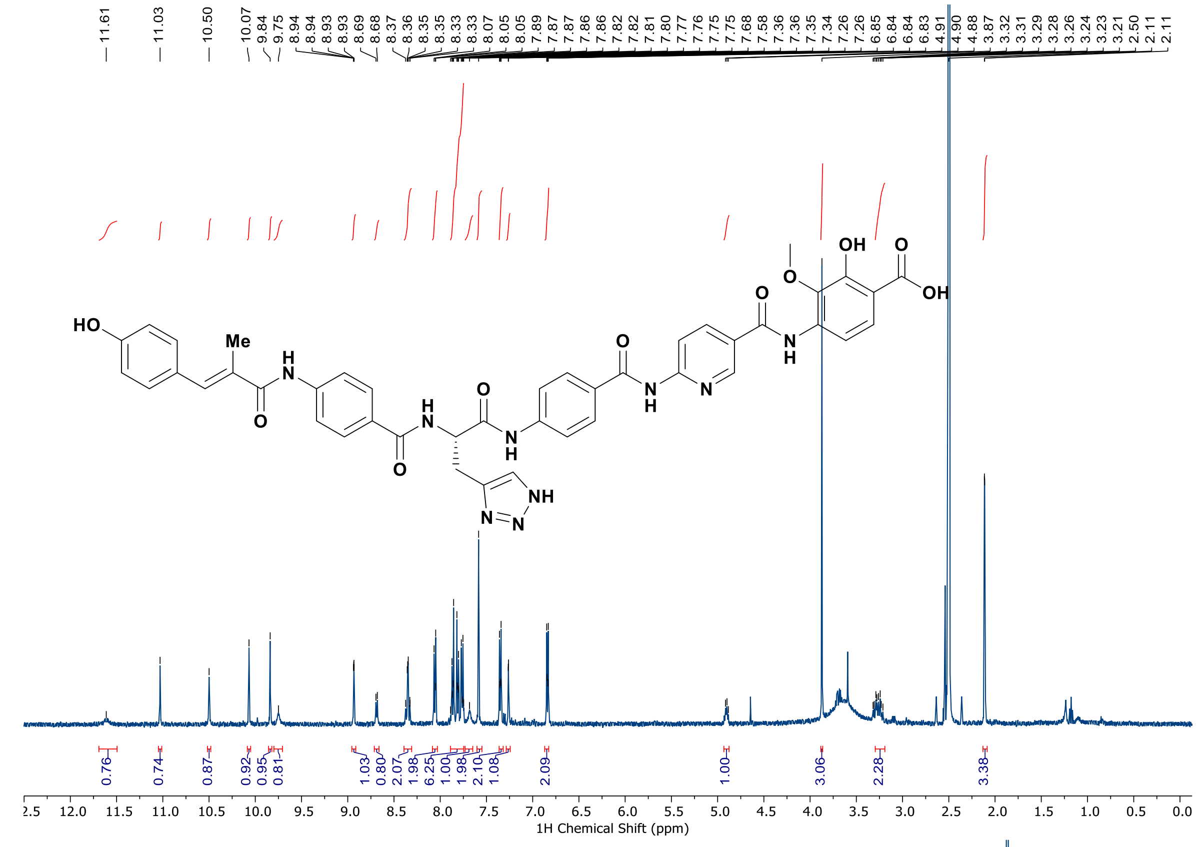

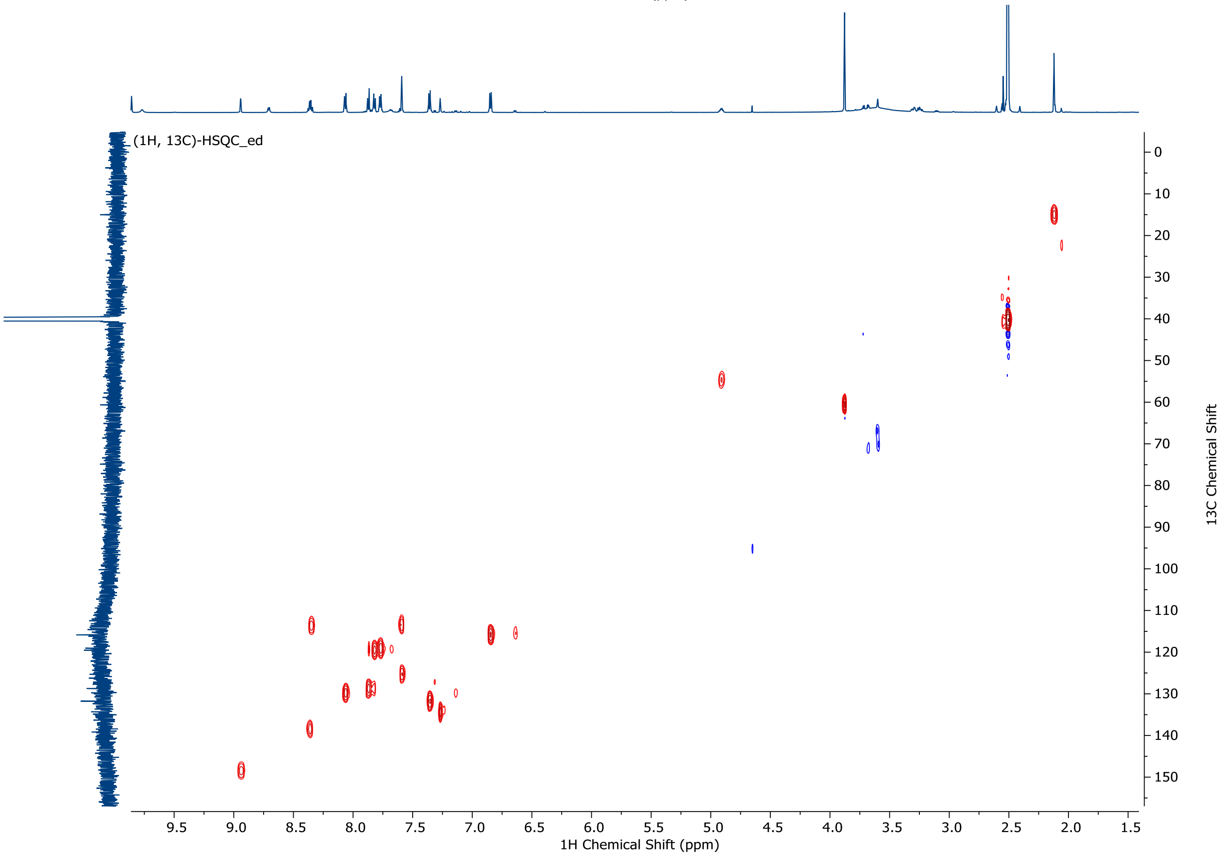

3.5 NMR spectra of D-Variation (7) and reaction intermediates

benzyl 4-(4-amino-2-(benzyloxy)-3-methoxybenzamido)-2-(benzyloxy)-3-methoxybenzoate (40)

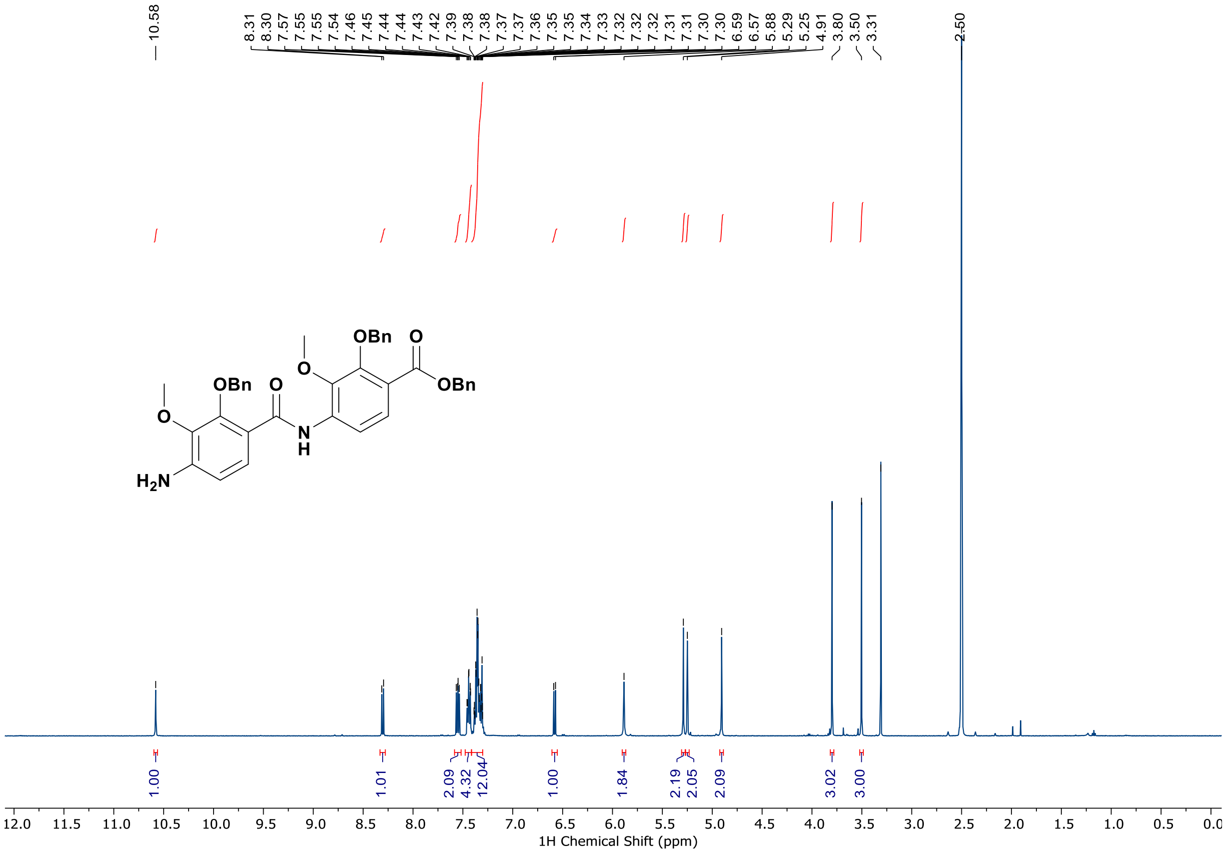

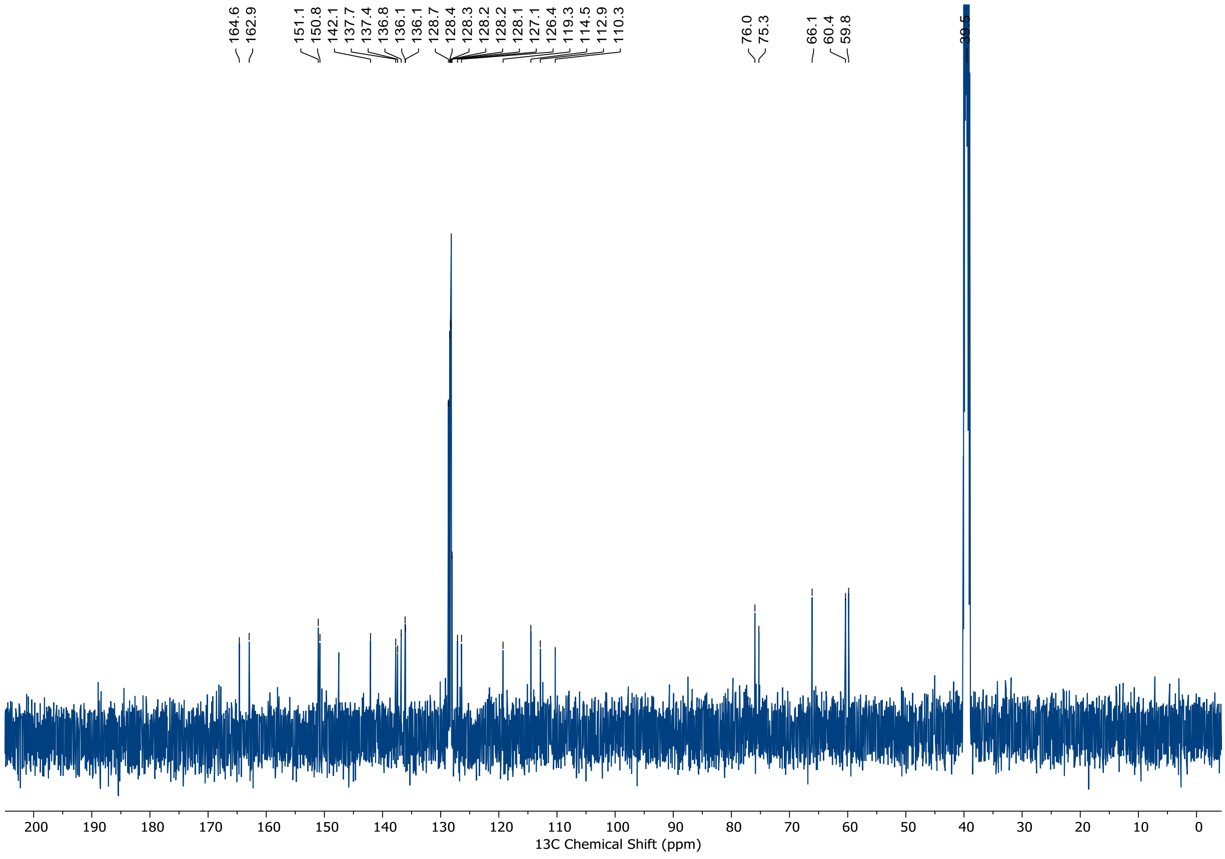

benzyl 2-(benzyloxy)-4-(2-(benzyloxy)-4-(6-((tert-butoxycarbonyl)amino)nicotinamido)-3-methoxybenzamido)-3-methoxybenzoate (40-1)

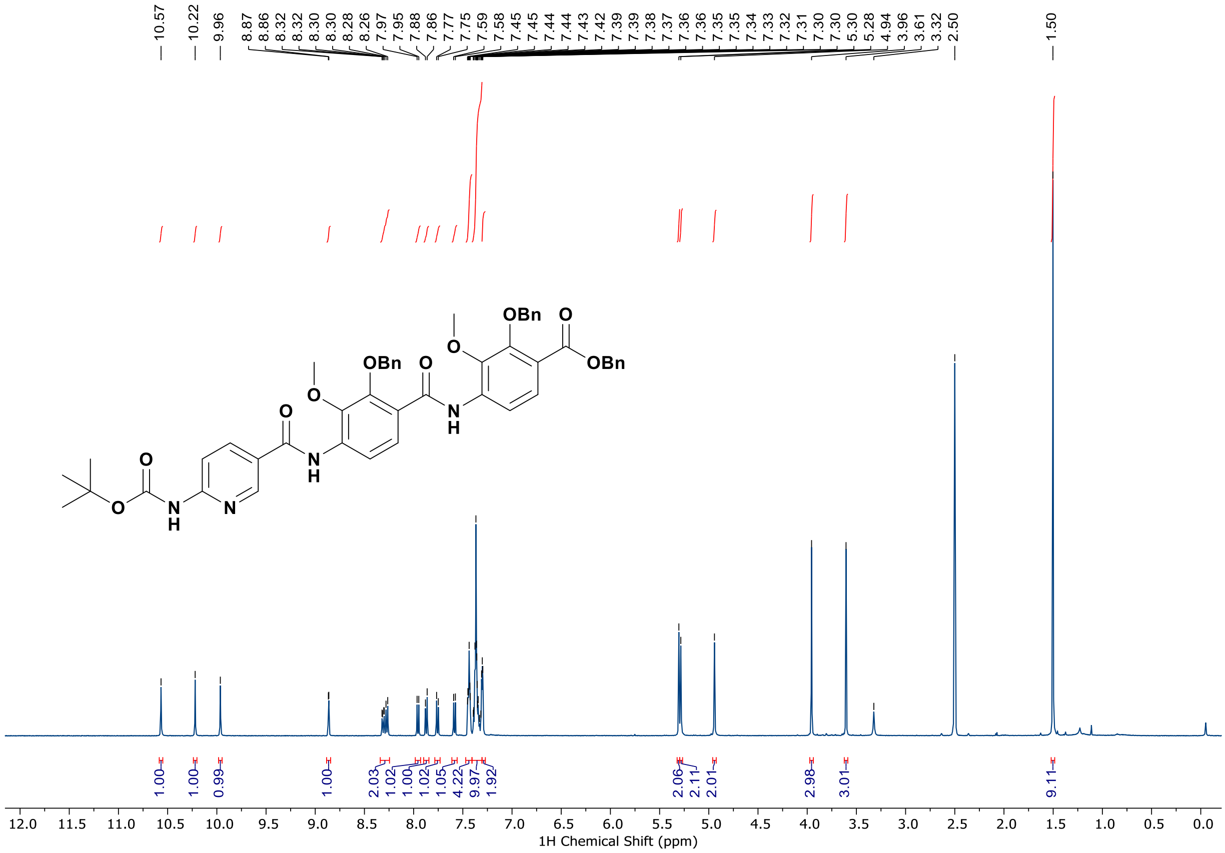

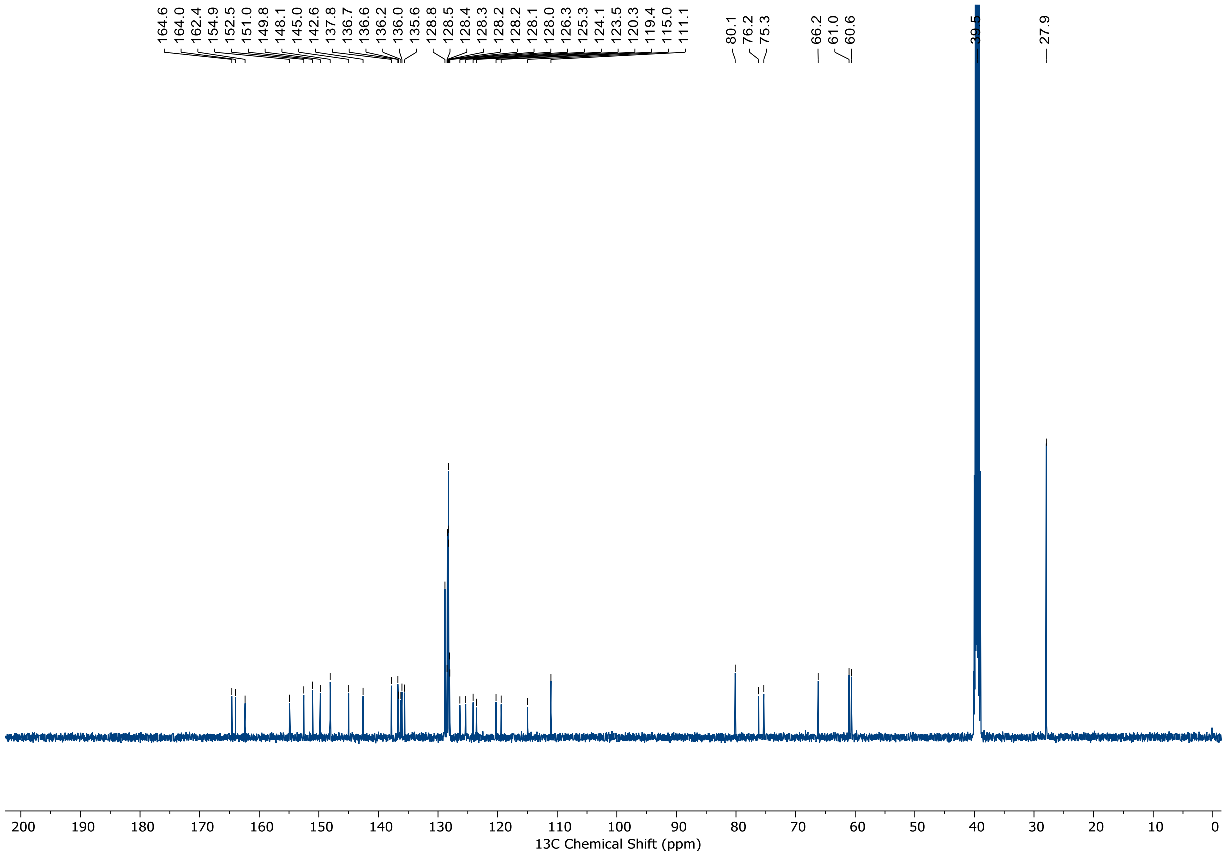

6-(((trichloromethoxy)carbonyl)amino)nicotinic acid (41-1)

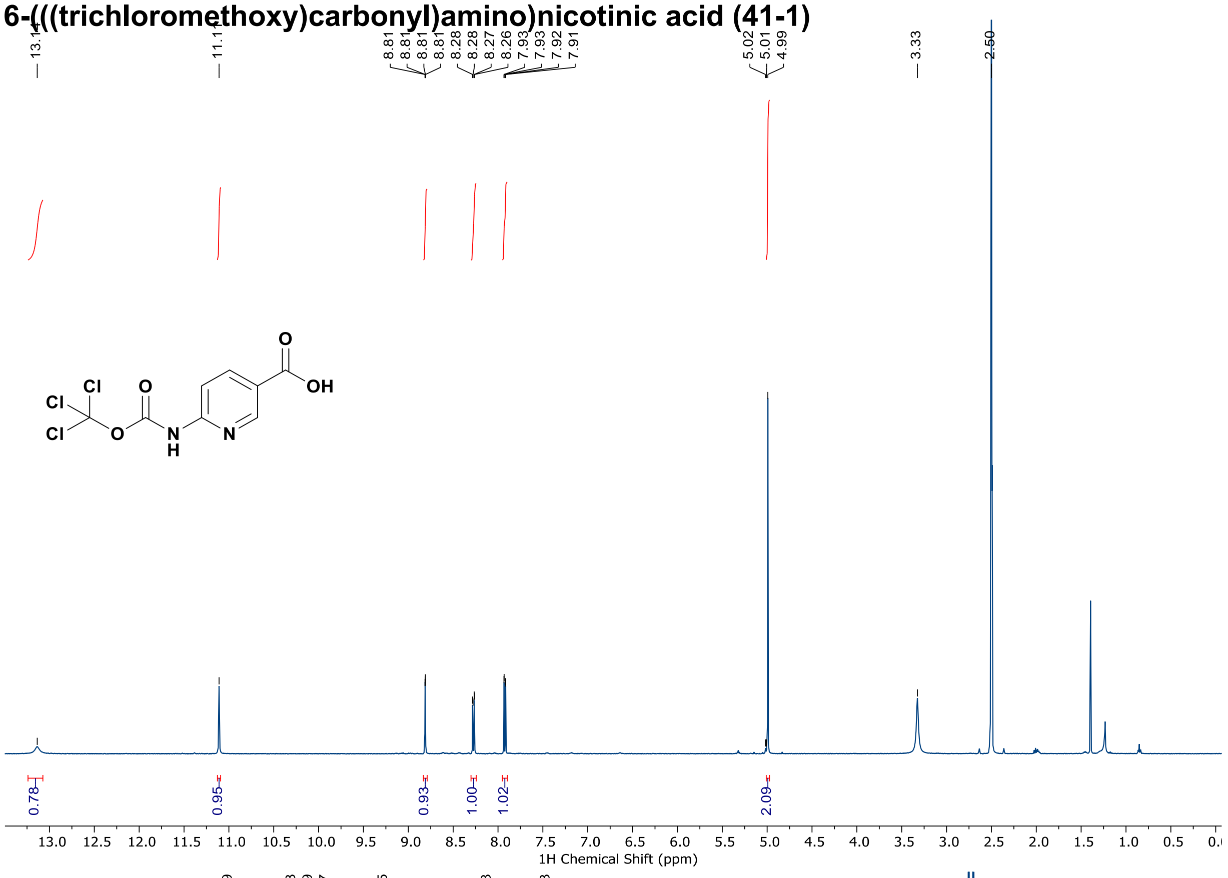

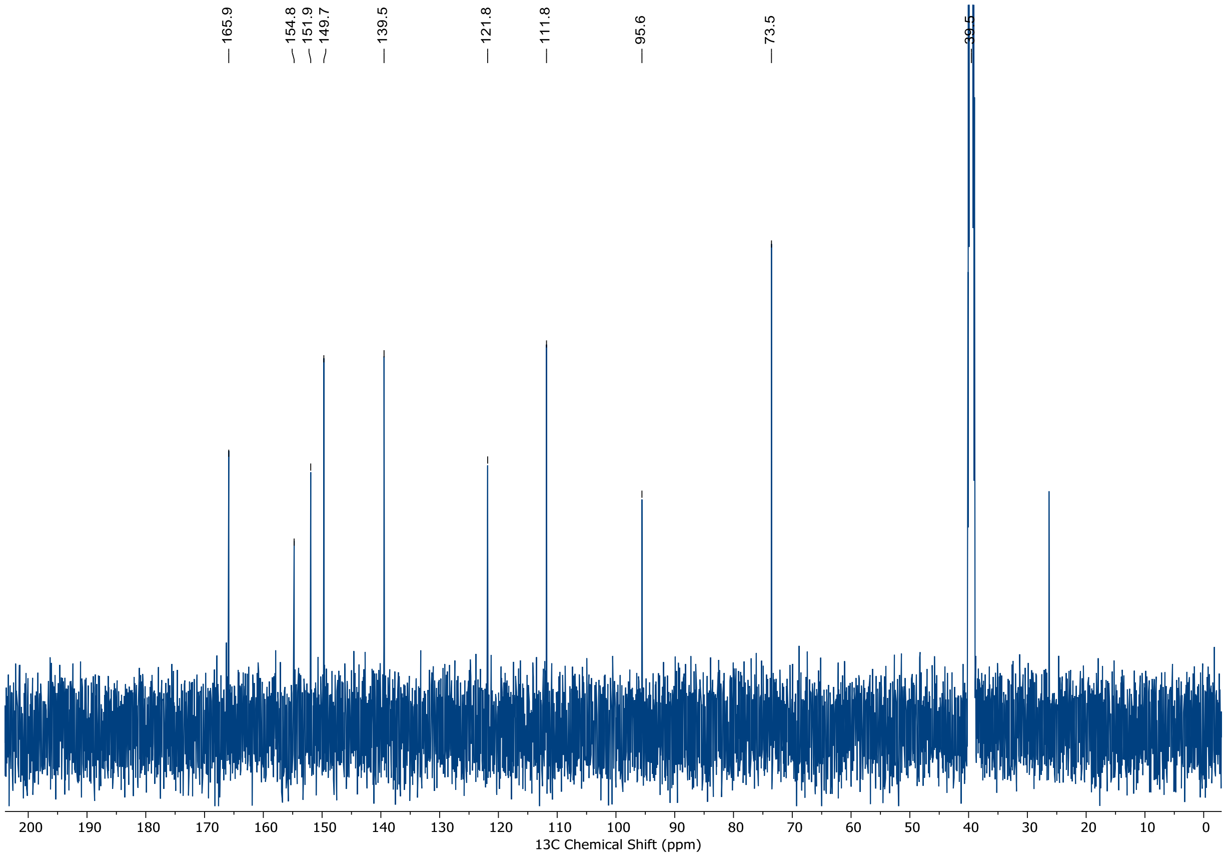

allyl 2-(allyloxy)-4-(2-(allyloxy)-4-amino-3-methoxybenzamido)-3-methoxybenzoate (35b)

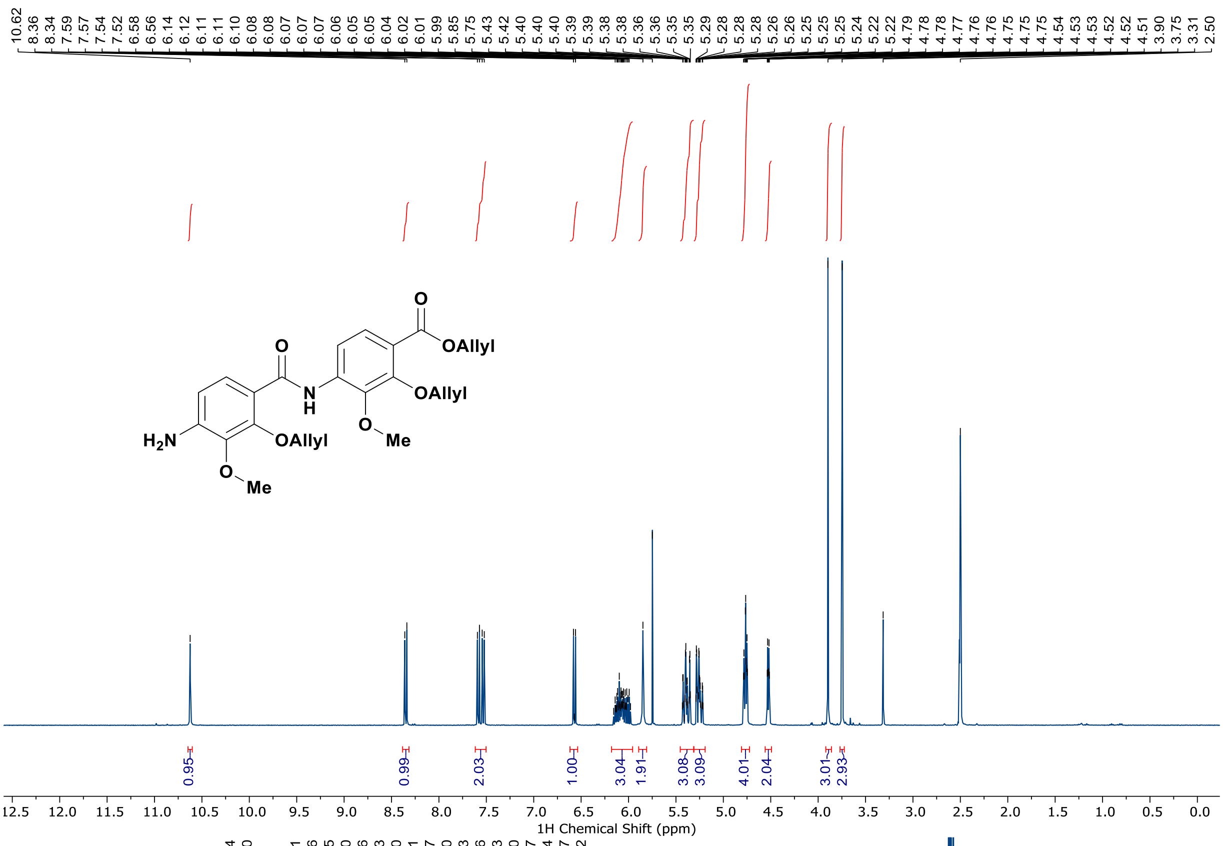

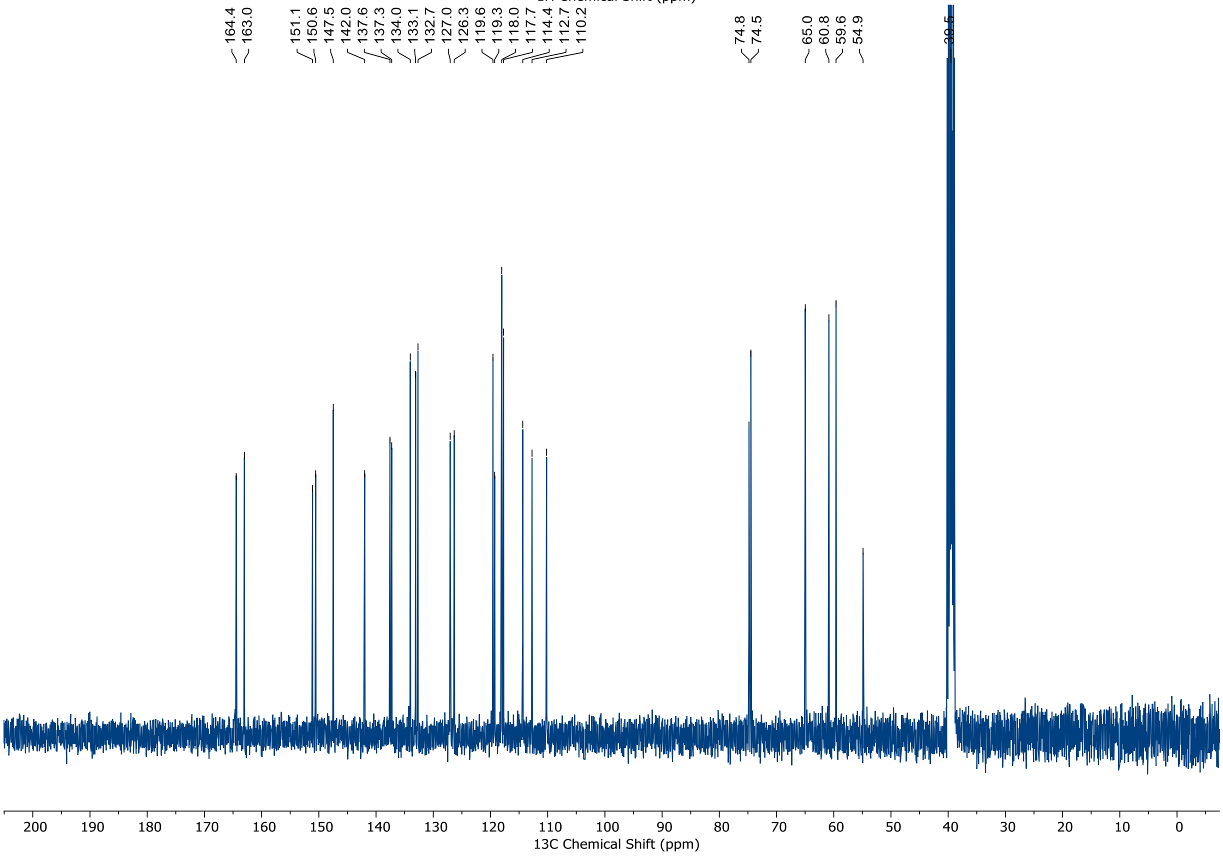

allyl 2-(allyloxy)-4-(2-(allyloxy)-4-(6-aminonicotinamido)-3-methoxybenzamido)-3-methoxybenzoate (41-3)

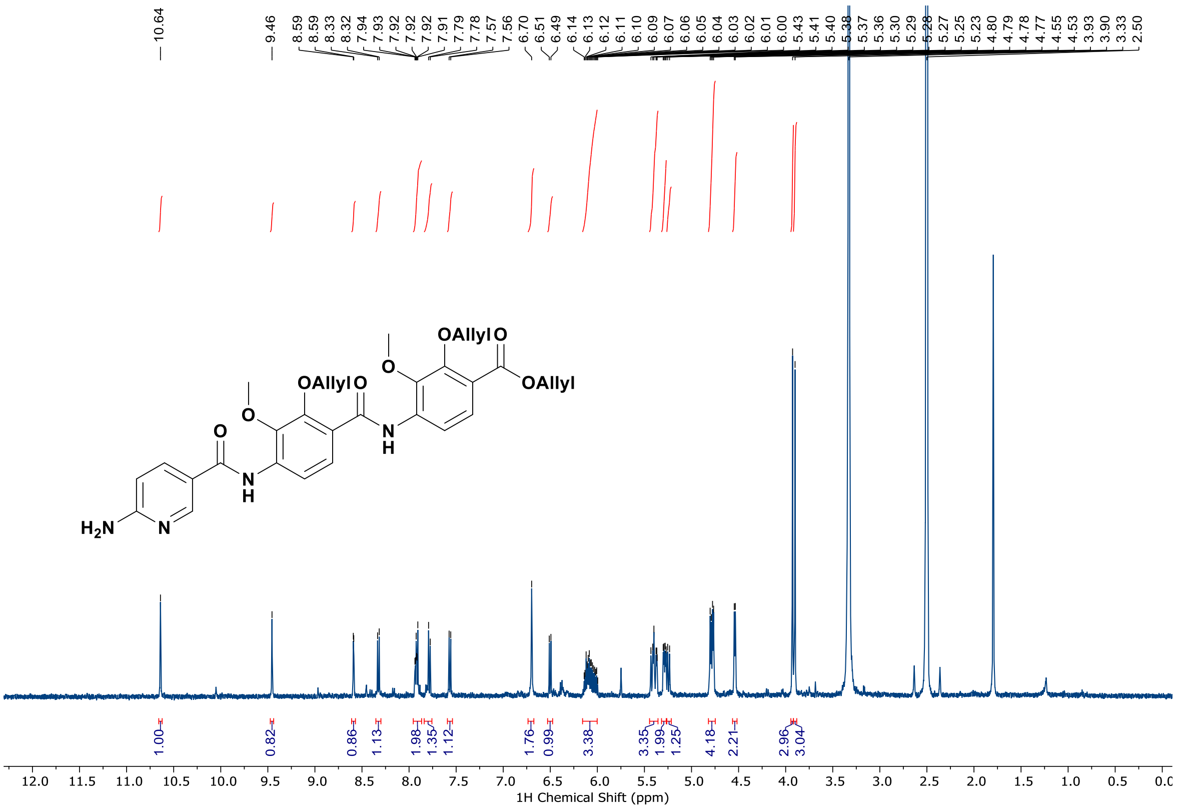

benzyl 4-(4-(6-aminonicotinamido)-2-(benzyloxy)-3-methoxybenzamido)-2-(benzyloxy)-3-methoxybenzoate (42-1)

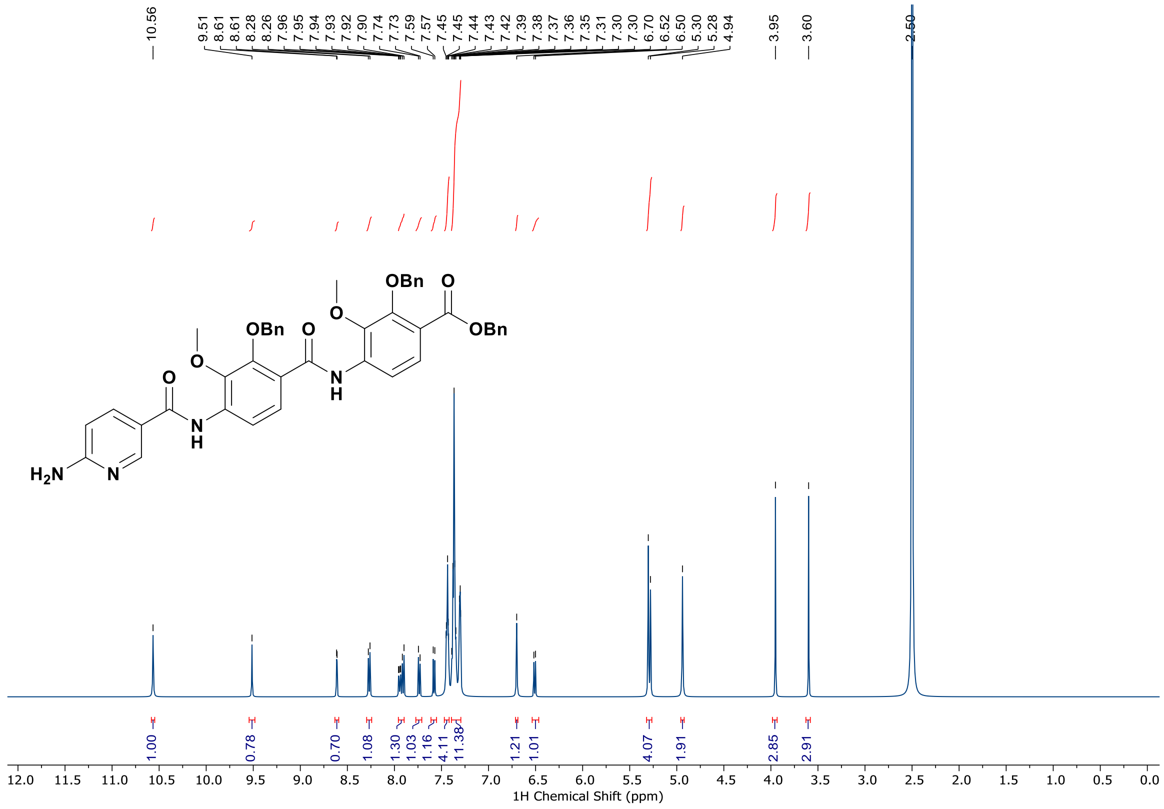

benzyl (S)-2-(benzyloxy)-4-(2-(benzyloxy)-4-(6-(2-((tert-butoxycarbonyl)amino)-3-(1-((pivaloyloxy)methyl)-1H-1,2,3-triazol-4-yl)propanamido)nicotinamido)-3-methoxybenzamido)-3-methoxybenzoate (42-2)

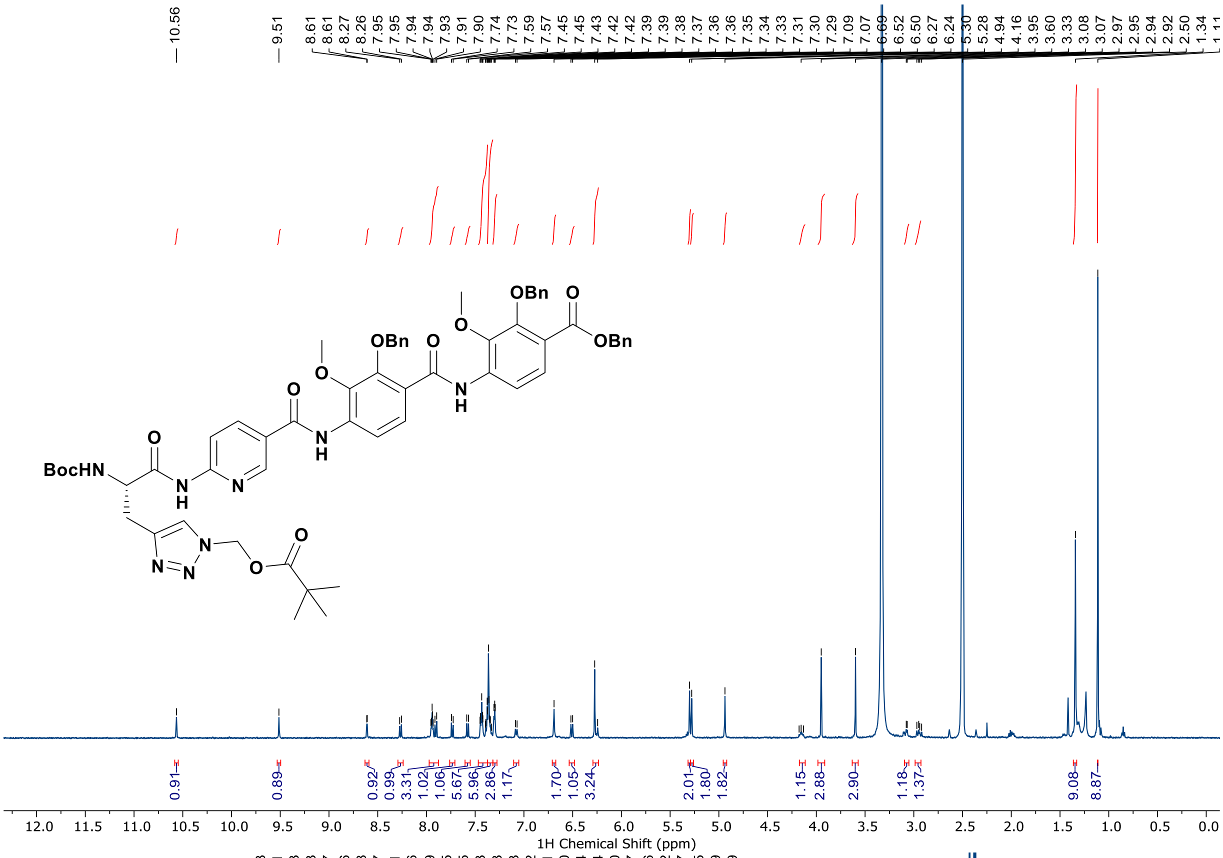

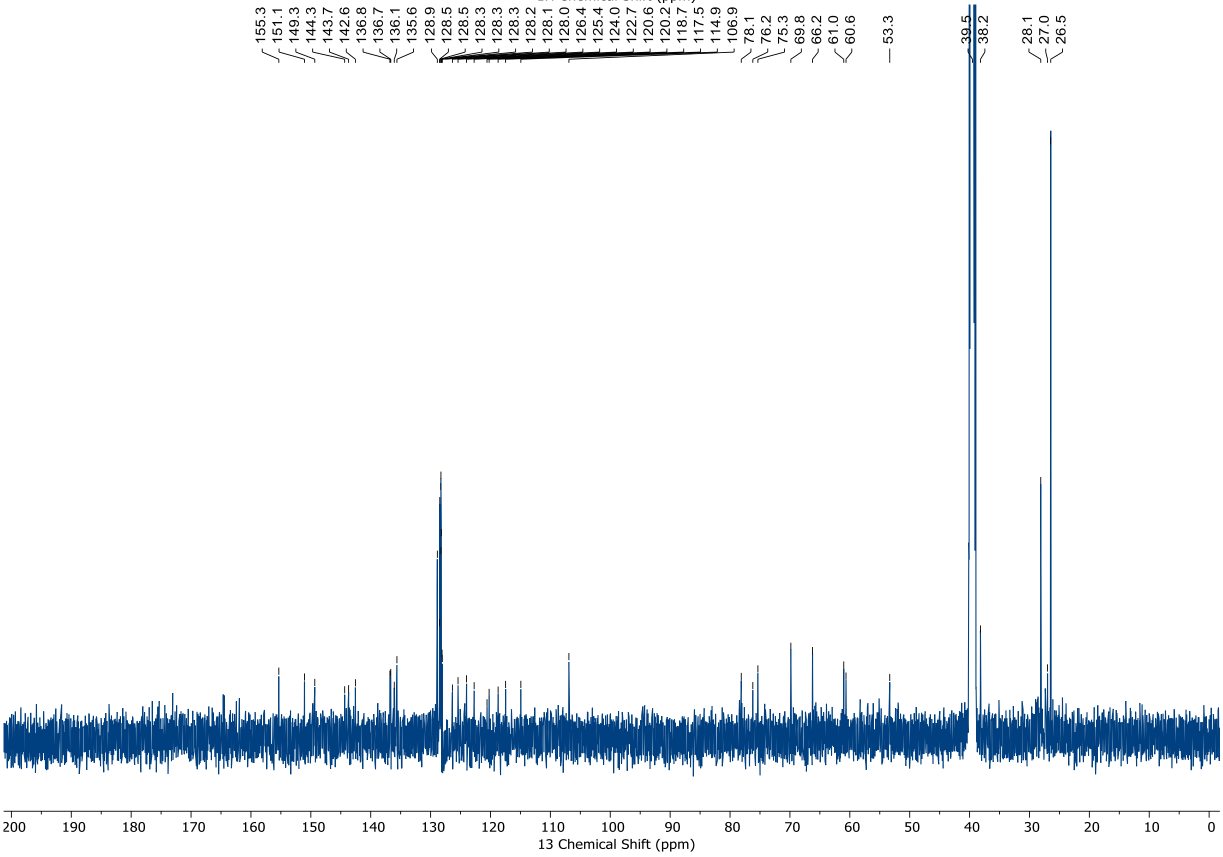

allyl 2-(allyloxy)-4-(2-(allyloxy)-3-methoxy-4-(5-nitropicolinamido)benzamido)-3-methoxybenzoate (35b-1)

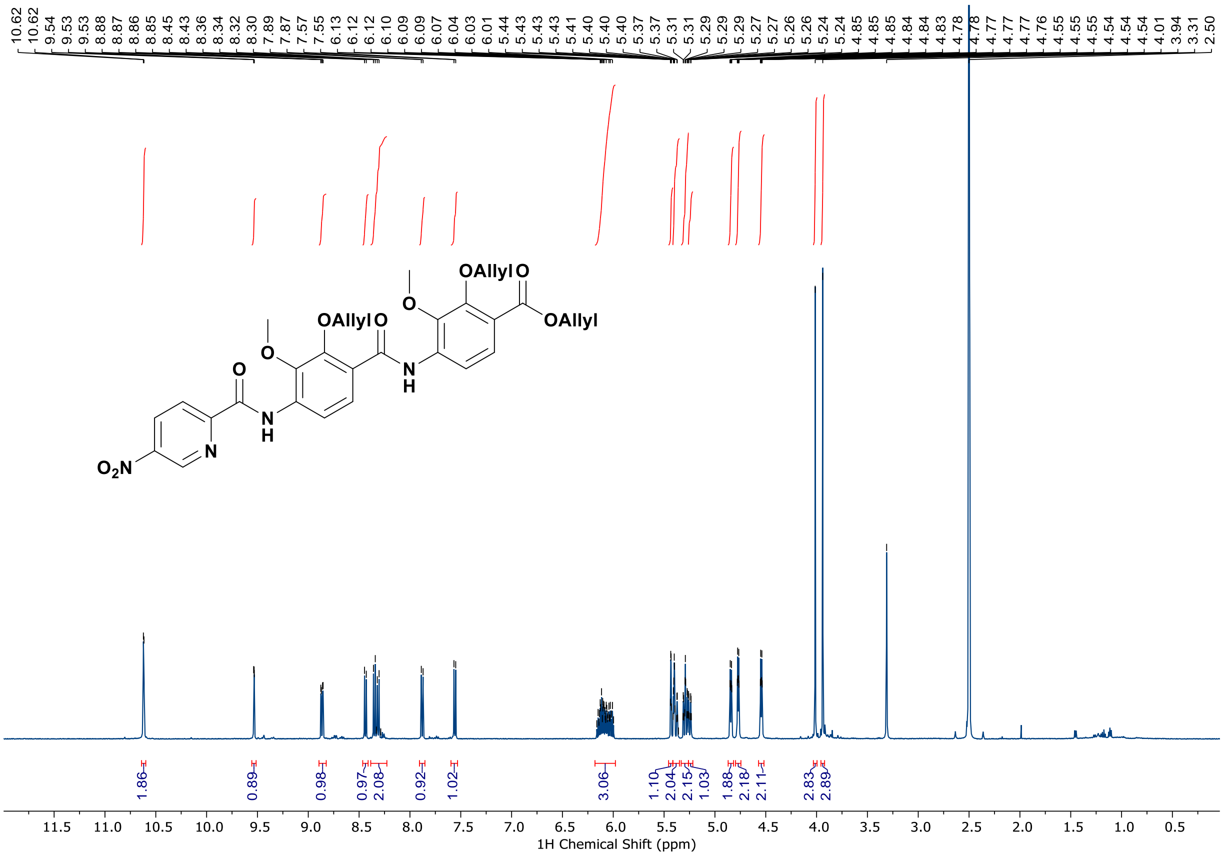

allyl 2-(allyloxy)-4-(2-(allyloxy)-4-(5-aminopicolinamido)-3-methoxybenzamido)-3-methoxybenzoate (36f)

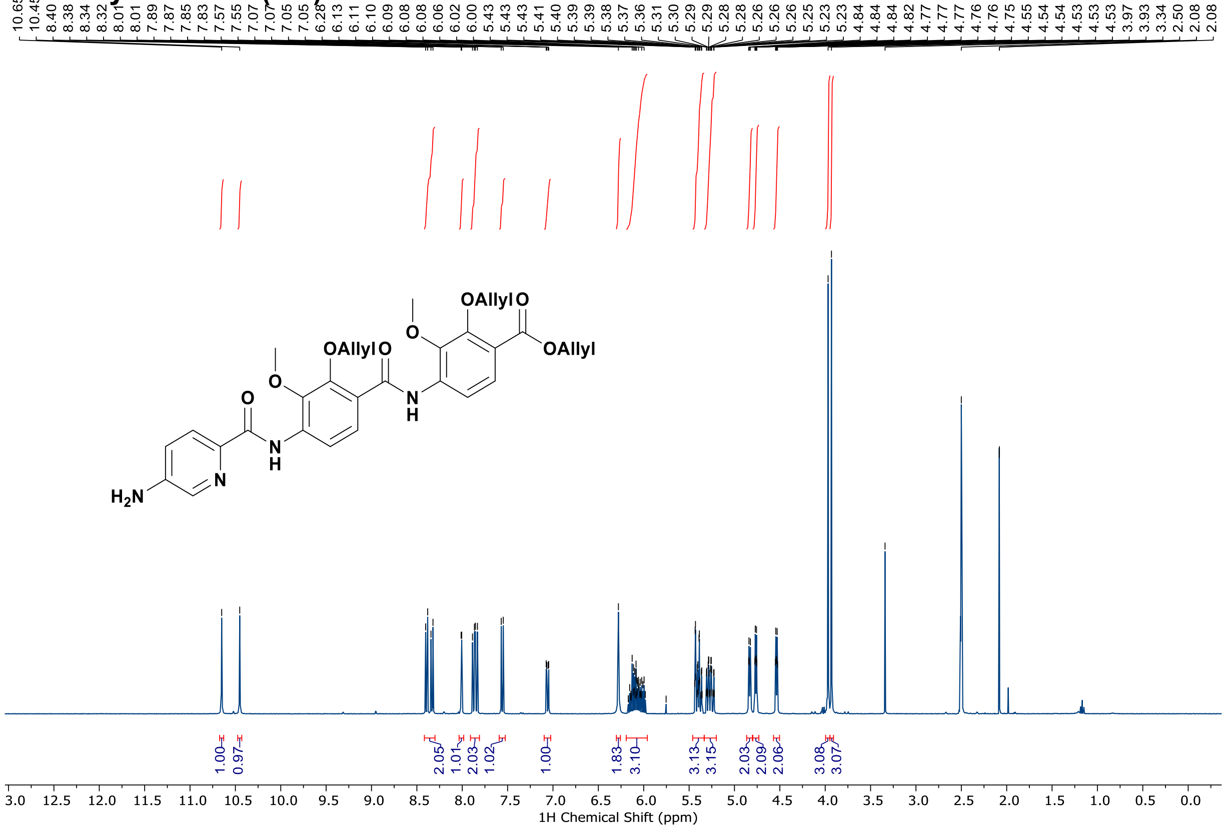

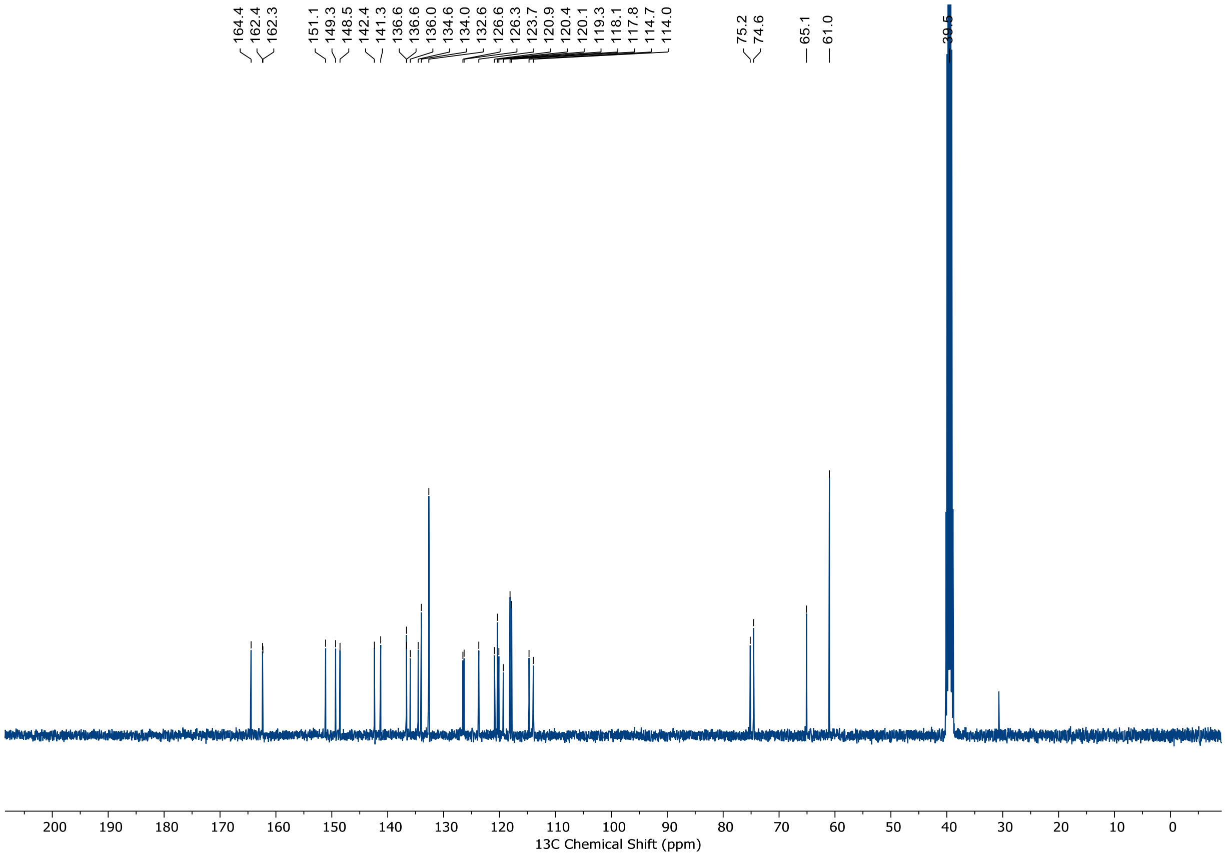

allyl (S)-2-(allyloxy)-4-(2-(allyloxy)-4-(5-(2-((tert-butoxycarbonyl)amino)-3-(1-((pivaloyloxy)methyl)-1H-1,2,3-triazol-4-yl)propanamido)picolinamido)-3-methoxybenzamido)-3-methoxybenzoate (36f-1)

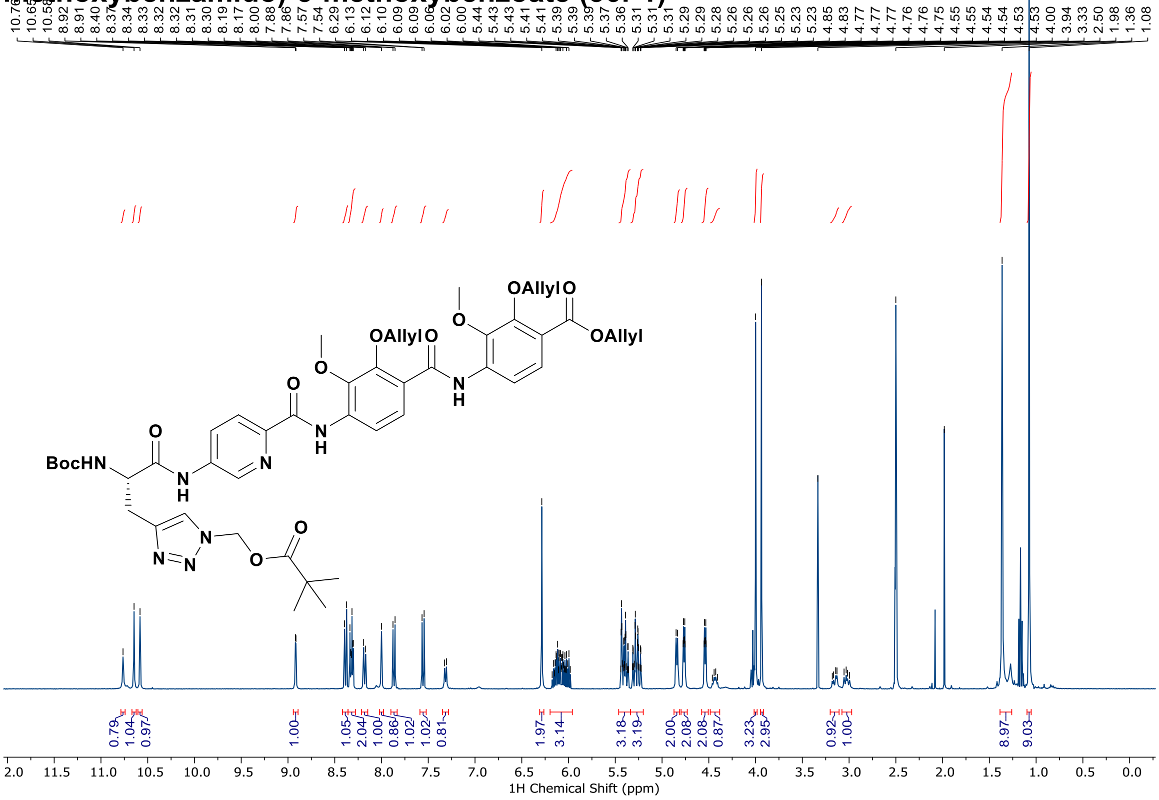

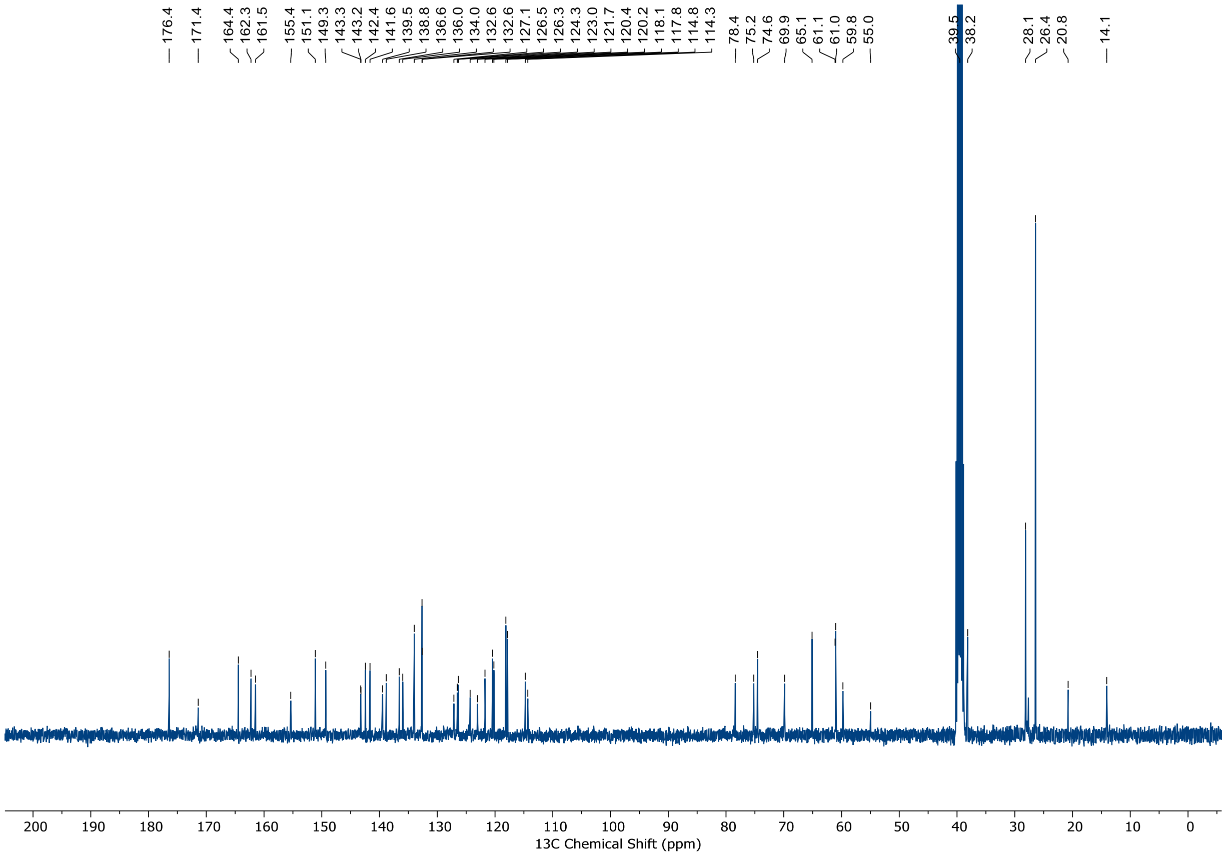

(S)-4-(4-(5-(2-((tert-butoxycarbonyl)amino)-3-(1-((pivaloyloxy)methyl)-1H-1,2,3-triazol-4-yl)propanamido)picolinamido)-2-hydroxy-3-methoxybenzamido)-2-hydroxy-3-methoxybenzoic acid (36f-2)

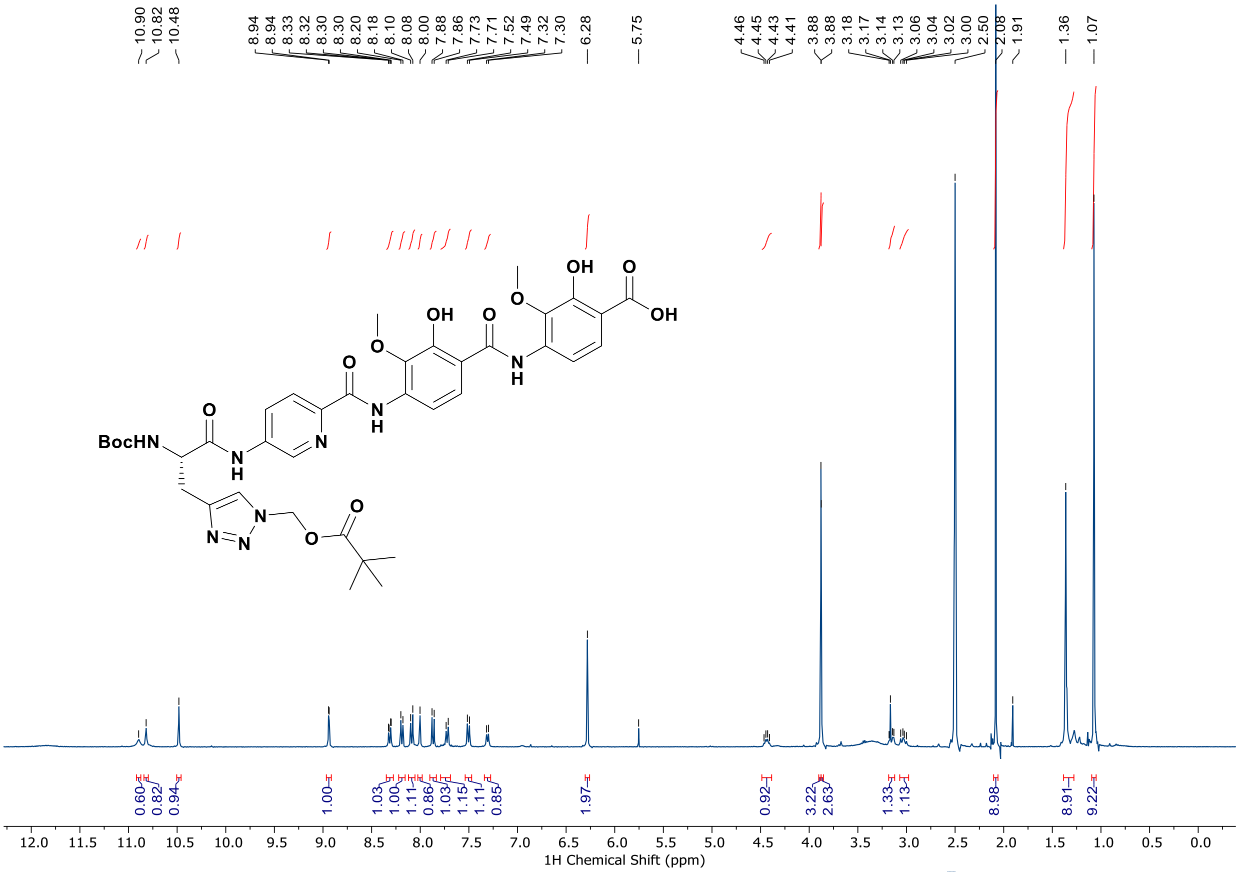

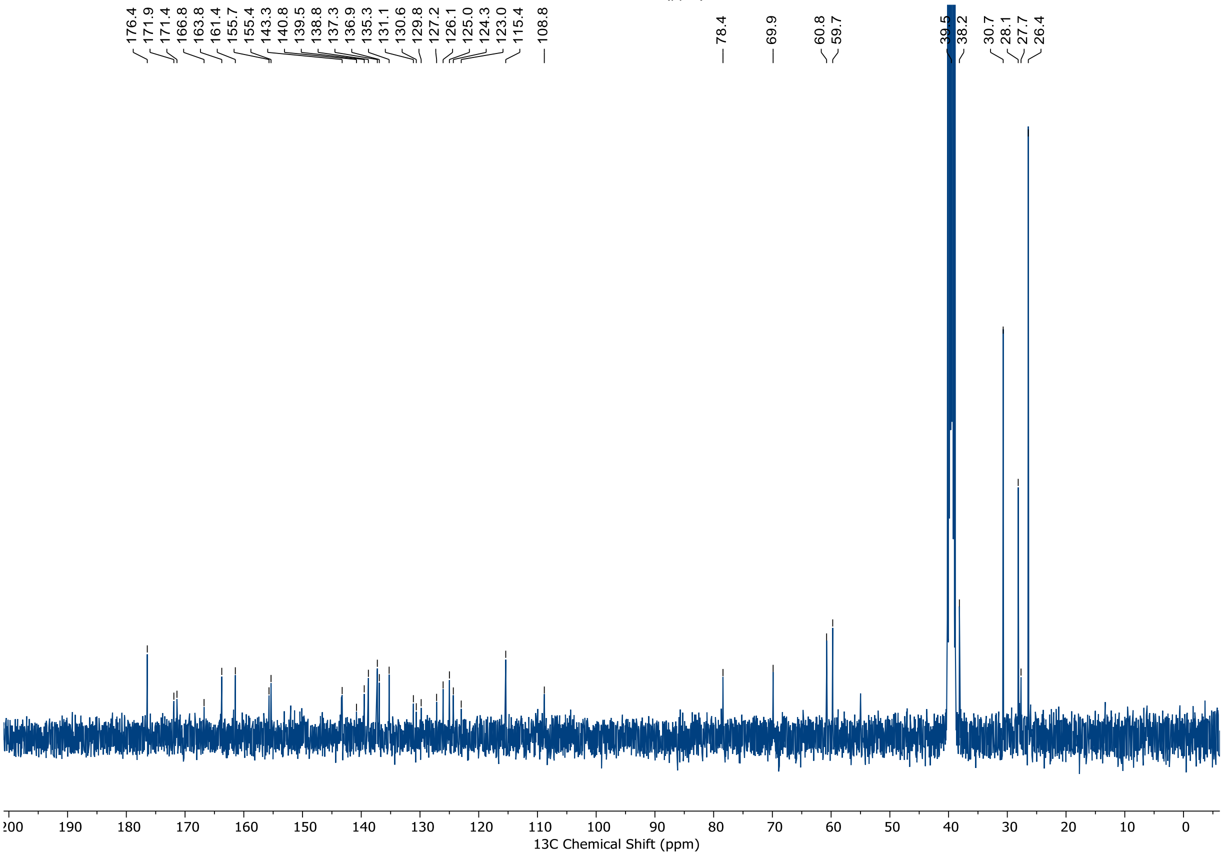

(S)-4-(4-(5-(2-amino-3-(1-((pivaloyloxy)methyl)-1H-1,2,3-triazol-4-yl)propanamido)picolinamido)-2-hydroxy-3-methoxybenzamido)-2-hydroxy-3-methoxybenzoic acid (37)

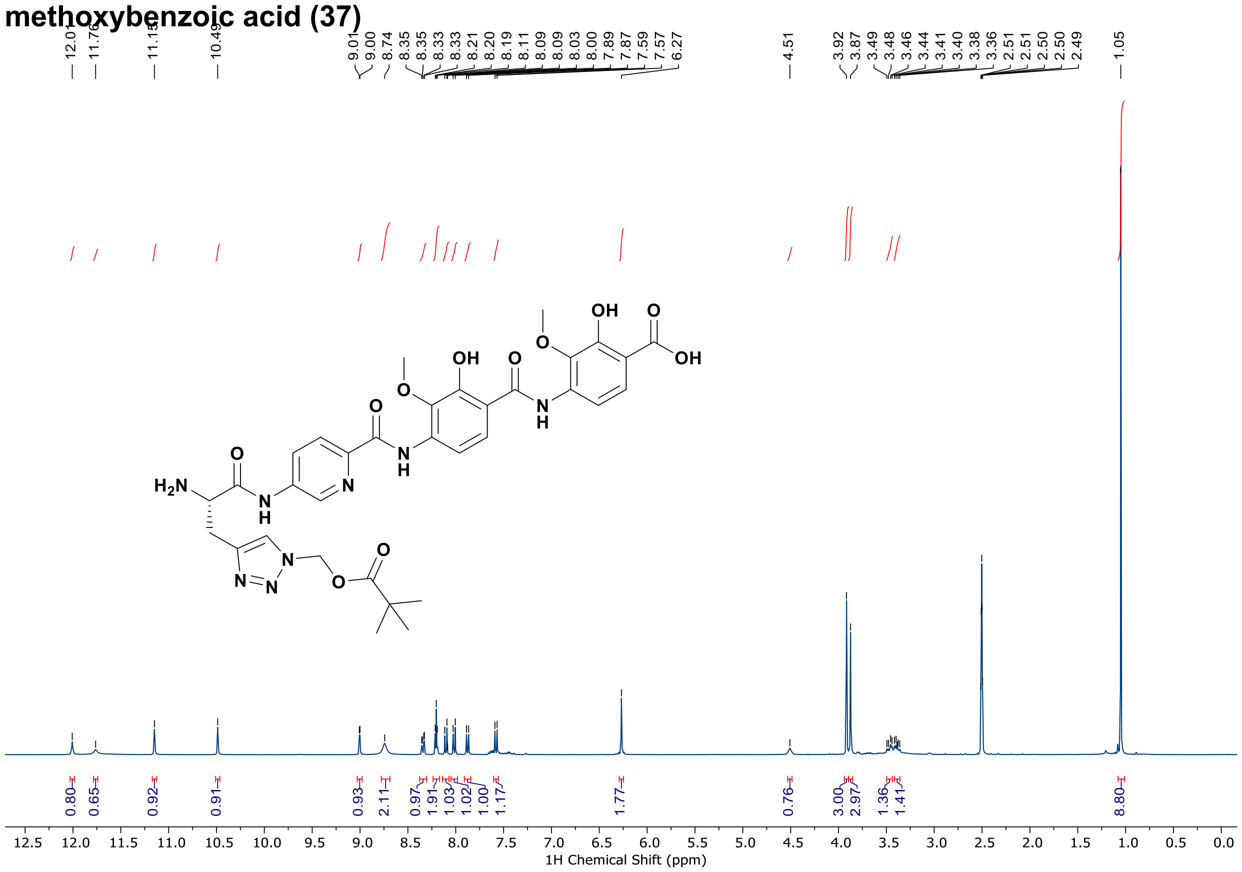

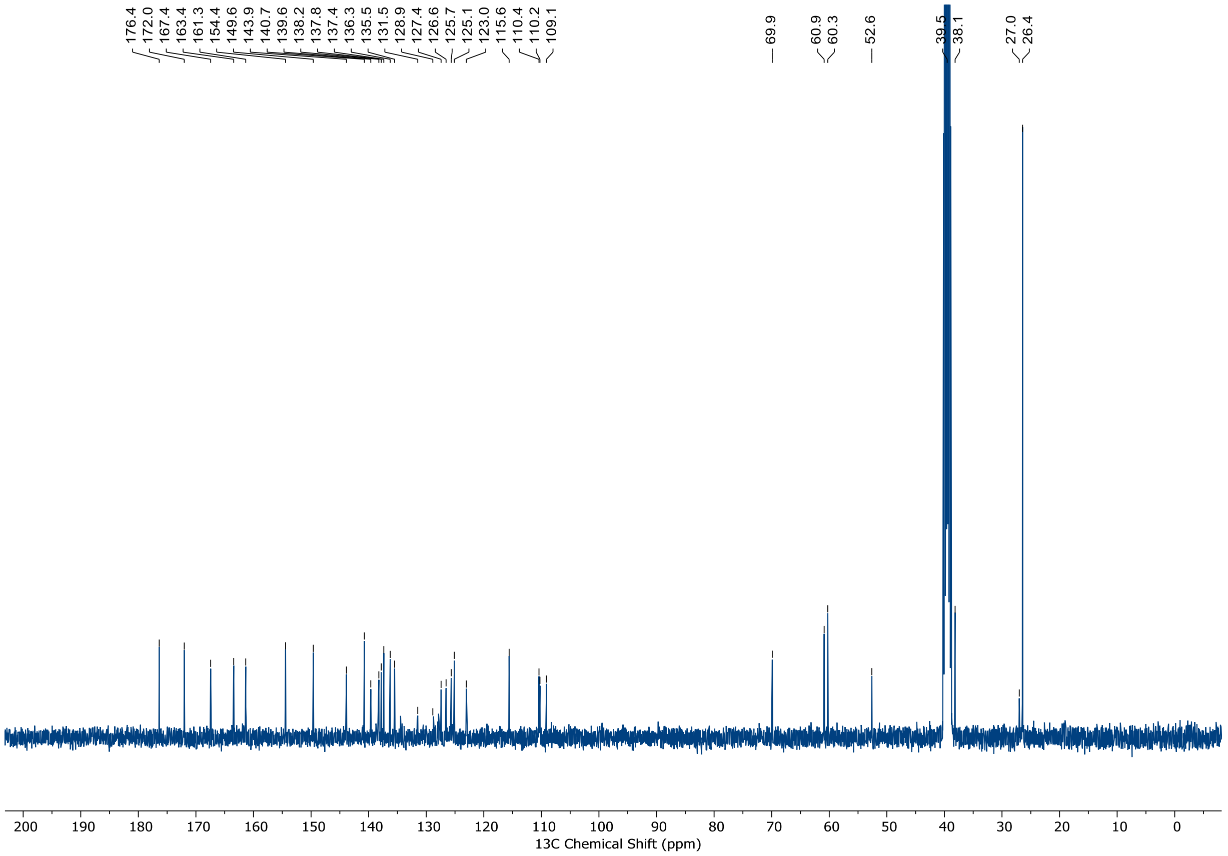

(S,E)-2-hydroxy-4-(2-hydroxy-4-(5-(2-(4-(3-(4-hydroxyphenyl)-2-methylacrylamido)benzamido)-3-(1H-1,2,3-triazol-4-yl)propanamido)picolinamido)-3-methoxybenzamido)-3-methoxybenzoic acid (7)

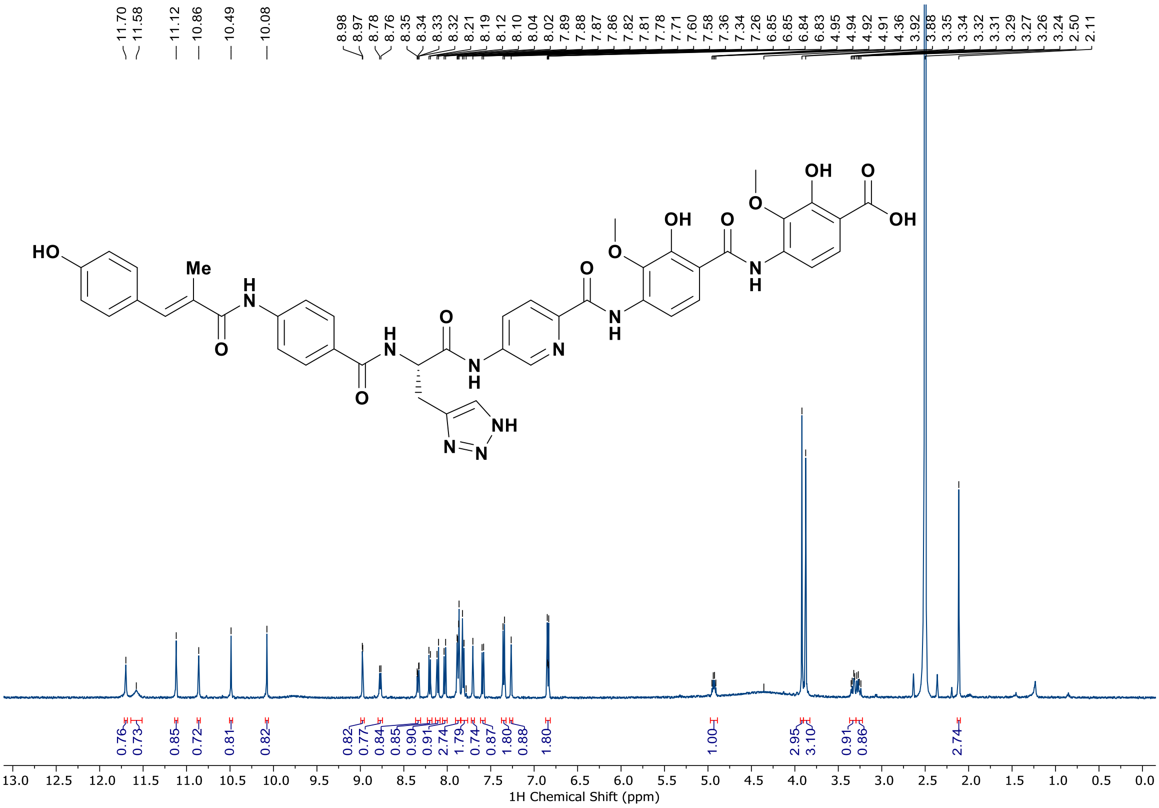

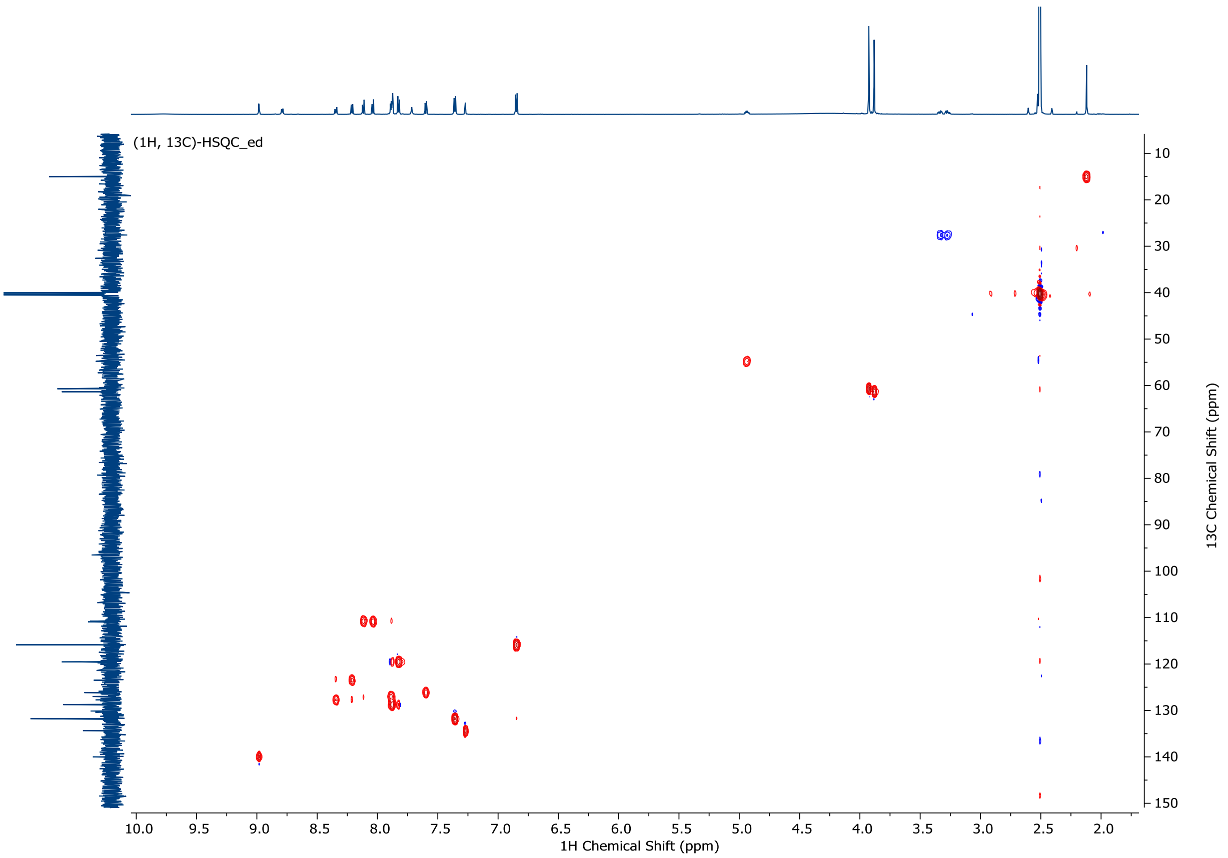

4 REFERENCES

1. M. Melvin P. Weinstein, *Methods for Dilution Antimicrobial Susceptibility Tests for Bacteria That Grow Aerobically, 11th Edition*, CLSI, 2018.
2. P. Heisig and R. Tschorny, *Antimicrob Agents Chemother*, 1994, **38**, 1284-1291.
3. L. Inspiralis, *E. coli Gyrase Supercoiling Assay Protocol*, https://www.inspiralis.com/assets/TechnicalDocuments/E.coli-Gyrase-Supercoiling-Assay-Protocol.pdf, 2021.
4. G. Testolin, K. Cirnski, K. Rox, H. Prochnow, V. Fetz, C. Grandclaudon, T. Mollner, A. Baiyoumy, A. Ritter, C. Leitner, J. Krull, J. Van Den Heuvel, A. Vassort, S. Sordello, M. M. Hamed, W. A. M. Elgaher, J. Herrmann, R. W. Hartmann, R. Müller and M. Brönstrup, *Chemical Science*, 2020, **11**, 1316-1334.
5. S. Baumann, J. Herrmann, R. Raju, H. Steinmetz, K. I. Mohr, S. Hüttel, K. Harmrolfs, M. Stadler and R. Müller, *Angewandte Chemie International Edition*, 2014, **53**, 14605-14609.
6. S. Hüttel, G. Testolin, J. Herrmann, T. Planke, F. Gille, M. Moreno, M. Stadler, M. Brönstrup, A. Kirschning and R. Müller, *Angewandte Chemie International Edition*, 2017, **56**, 12760-12764.
7. Y. Cheng, T. C. Judd, M. D. Bartberger, J. Brown, K. Chen, R. T. Fremeau, D. Hickman, S. A. Hitchcock, B. Jordan, V. Li, P. Lopez, S. W. Louie, Y. Luo, K. Michelsen, T. Nixey, T. S. Powers, C. Rattan, E. A. Sickmier, D. J. St Jean, R. C. Wahl, P. H. Wen and S. Wood, *Journal of Medicinal Chemistry*, 2011, **54**, 5836–5857.
8. I. Behroz, P. Durkin, S. Gratz, M. Seidel, L. Rostock, M. Spinczyk, J. B. Weston and R. D. Sussmuth, *Chemistry*, 2019, DOI: 10.1002/chem.201904752.
9. L. v. Eckardstein, D. Petras, T. Dang, S. Cociancich, S. Sabri, S. Grätz, D. Kerwat, M. Seidel, A. Pesic, P. C. Dorrestein, M. Royer, J. B. Weston and R. D. Süssmuth, *Chemistry – A European Journal*, 2017, **23**, 15316–15321.
10. J. Kretz, D. Kerwat, V. Schubert, S. Grätz, A. Pesic, S. Semsary, S. Cociancich, M. Royer and R. D. Süssmuth, *Angewandte Chemie International Edition*, 2014, **54**, 1969–1973.
11. S. Kishimoto, S. Nishimura, M. Hatano, M. Igarashi and H. Kakeya, *The Journal of Organic Chemistry*, 2015, **80**, 6076-6082.
12. P. C. Chiang and Y. Hu, *Comb Chem High Throughput Screen*, 2009, **12**, 250-257.
13. MarvinSketch, *Journal*, 2021.
14. P. Pracht, F. Bohle and S. Grimme, *Physical Chemistry Chemical Physics*, 2020, **22**, 7169-7192.
15. C. Bannwarth, S. Ehlert and S. Grimme, *Journal of Chemical Theory and Computation*, 2019, **15**, 1652-1671.
16. S. Grimme and C. Bannwarth, *The Journal of Chemical Physics*, 2016, **145**, 054103.
17. A. D. Becke, *The Journal of Chemical Physics*, 1993, **98**, 5648-5652.
18. C. Lee, W. Yang and R. G. Parr, *Physical Review B*, 1988, **37**, 785-789.
19. S. H. Vosko, L. Wilk and M. Nusair, *Canadian Journal of Physics*, 1980, **58**, 1200-1211.
20. P. J. Stephens, F. J. Devlin, C. F. Chabalowski and M. J. Frisch, *The Journal of Physical Chemistry*, 1994, **98**, 11623-11627.
21. T. Clark, J. Chandrasekhar, G. W. Spitznagel and P. V. R. Schleyer, *Journal of Computational Chemistry*, 1983, **4**, 294-301.
22. R. Krishnan, J. S. Binkley, R. Seeger and J. A. Pople, *The Journal of Chemical Physics*, 1980, **72**, 650-654.
23. S. Grimme, J. Antony, S. Ehrlich and H. Krieg, *The Journal of Chemical Physics*, 2010, **132**, 154104.
24. S. Grimme, S. Ehrlich and L. Goerigk, *Journal of Computational Chemistry*, 2011, **32**, 1456-1465.
25. Y. Takano and K. N. Houk, *Journal of Chemical Theory and Computation*, 2005, **1**, 70-77.

26. M. J. Frisch, G. W. Trucks, H. B. Schlegel, G. E. Scuseria, M. A. Robb, J. R. Cheeseman, G. Scalmani, V. Barone, G. A. Petersson, H. Nakatsuji, X. Li, M. Caricato, A. V. Marenich, J. Bloino, B. G. Janesko, R. Gomperts, B. Mennucci, H. P. Hratchian, J. V. Ortiz, A. F. Izmaylov, J. L. Sonnenberg, Williams, F. Ding, F. Lipparini, F. Egidi, J. Goings, B. Peng, A. Petrone, T. Henderson, D. Ranasinghe, V. G. Zakrzewski, J. Gao, N. Rega, G. Zheng, W. Liang, M. Hada, M. Ehara, K. Toyota, R. Fukuda, J. Hasegawa, M. Ishida, T. Nakajima, Y. Honda, O. Kitao, H. Nakai, T. Vreven, K. Throssell, J. A. Montgomery Jr., J. E. Peralta, F. Ogliaro, M. J. Bearpark, J. J. Heyd, E. N. Brothers, K. N. Kudin, V. N. Staroverov, T. A. Keith, R. Kobayashi, J. Normand, K. Raghavachari, A. P. Rendell, J. C. Burant, S. S. Iyengar, J. Tomasi, M. Cossi, J. M. Millam, M. Klene, C. Adamo, R. Cammi, J. W. Ochterski, R. L. Martin, K. Morokuma, O. Farkas, J. B. Foresman and D. J. Fox, *Journal*, 2016.
27. S.A. Ahmed, R. M.Gogal Jr., J. E. Walsh, Journal of Immunological Methods, **1994** *170*, 211-224.
